# Combining photoredox catalysis and hydrogen atom transfer for dearomative functionalization of electron rich heteroarenes†

**DOI:** 10.1039/d3sc00060e

**Published:** 2023-02-28

**Authors:** Peng Ji, Xiang Meng, Jing Chen, Feng Gao, Hang Xu, Wei Wang

**Affiliations:** a Department of Pharmacology and Toxicology, R. Ken Coit College of Pharmacy, University of Arizona USA; b Department of Chemistry and Biochemistry, University of Arizona USA; c University of Arizona Cancer Centre, University of Arizona 1703 E. Mabel Street Tucson AZ 85721-0207 USA wang@pharmacy.arizona.edu

## Abstract

Reductive dearomatization has been a broadly explored means for rapid generation of sp^3^ complexity from simple planar arenes. Breaking the electron rich, stable aromatic systems requires strong reduction conditions. It has been notoriously challenging to dearomatize electron even richer heteroarenes. Herein we report an umpolung strategy enabling dearomatization of such structures under mild conditions. The reversal of the reactivity of these electron rich aromatics *via* photoredox mediated single electron transfer (SET) oxidation generates electrophilic radical cations, which can react with nucleophiles and break the aromatic structure to form a Birch type radical species. A crucial hydrogen atom transfer (HAT) is successfully engineered into the process to efficiently trap the dearomatic radical and minimize the formation of the overwhelmingly favorable, irreversible aromatization products. Particularly, a non-canonical dearomative ring-cleavage of thiophene/furan through selective C(sp^2^)–S bond breaking was first discovered. The preparing power of the protocol has been demonstrated for selective dearomatization and functionalization of various electron rich heteroarenes including thiophenes, furans, benzothiophenes and indoles. Furthermore, the process offers an unrivaled capacity for simultaneously introducing C–N/O/P bonds on these structures as exemplified by various “N”, “O” and “P” centered functional moieties with 96 examples.

## Abstract 2

Engineering organophotoredox catalysis with hydrogen atom transfer directs toward an unfavorable dearomatization process under mild reaction conditions.

## Graphical abstract

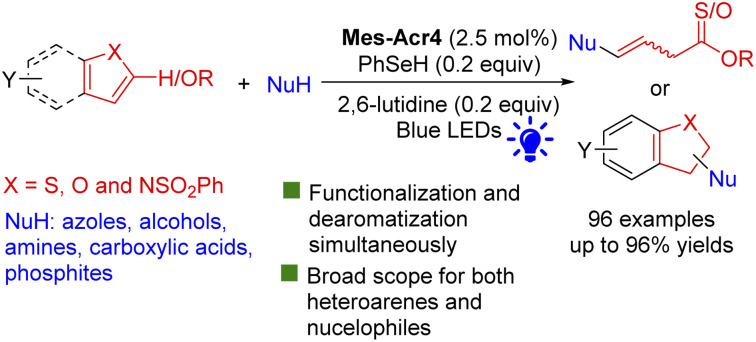

## Introduction

**Scheme 1:**
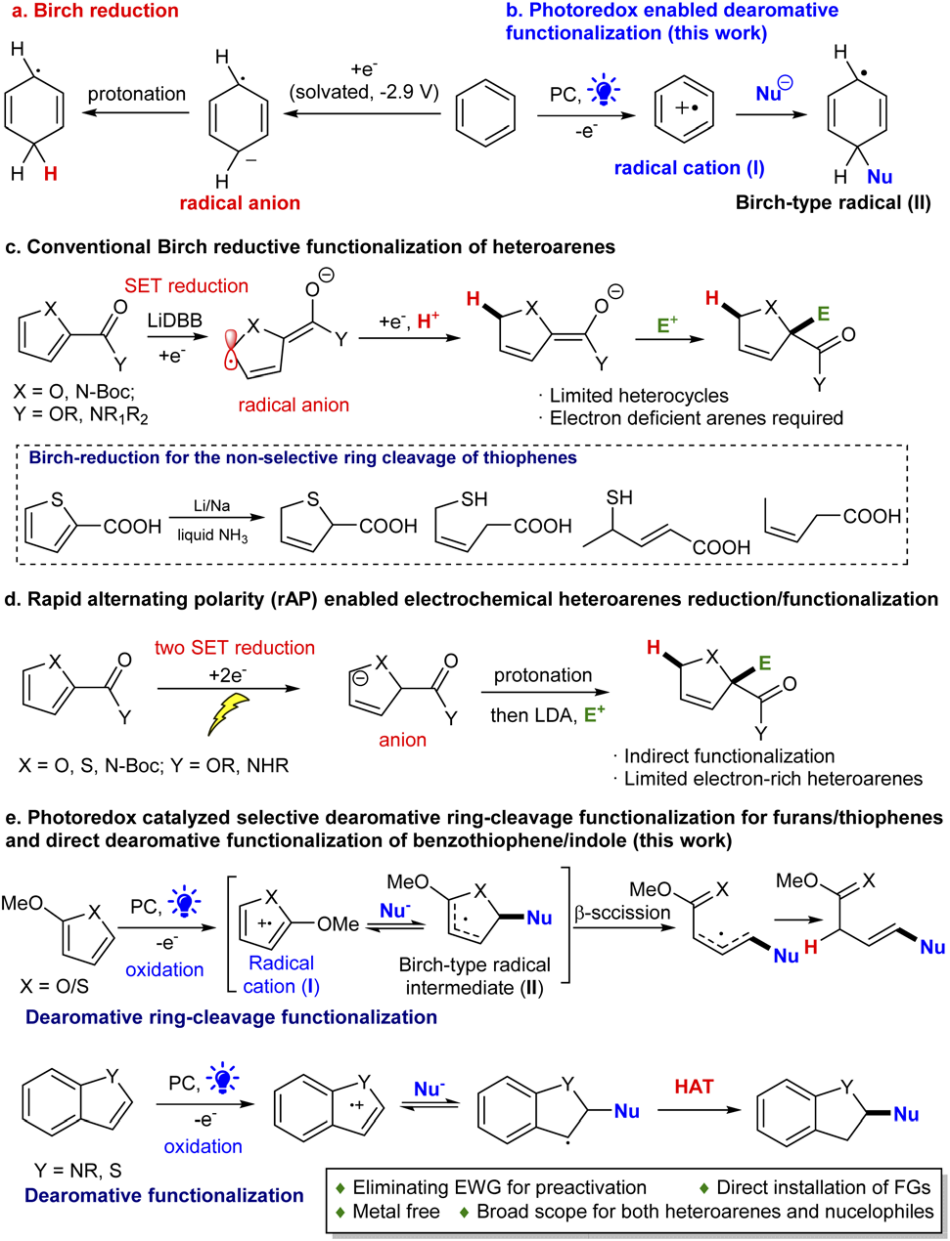
Reductive dearomatization methods.

Reductive dearomatization, a viable strategy for rapid generation of sp^3^ complexity from simple arenes,^1–3^ has been broadly explored in organic synthesis.^4,5^ Among them, Birch reduction is a well-established example of such transformations.^2^ The process is often carried out under harsh reaction conditions since the electron rich nature of aromatic systems makes them difficult to break reductively (Scheme 1a). Recent efforts have made the process more practical and safer. Koide developed an elegant scalable protocol employing lithium/ethylenediamine in THF.^6^ Moreover, new reduction strategies have been developed using electrochemical and photochemical methods by Baran,^7^ König,^8^ and Miyake^9^ under mild reaction conditions. However, the scope of these methods is still largely limited to non-heteroaromatic hydrocarbon substrates. Very limited success has been observed for dearomatizing heteroarenes particularly 5-membered ring systems because of the inherent electron richer nature, metal poisoning effect and ring fragmentation.^10–14^ Donohoe and House reported the first example using LiDBB (lithium di-*tert*-butylbiphenyl) to reduce the electron-deficient furan/pyrroles (Scheme 1c).^15,16^ However, for thiophene rings, the Birch-type reaction usually results in a non-selective, dearomative ring cleavage or direct dearomative reduction product (Scheme 1c).^16^ Recently, Baran disclosed a rapid alternating polarity (rAP) method enabling the selective reduction of five membered ring heteroarenes (Scheme 1d).^17^ It is recognized that electron-withdrawing groups (EWGs) (*e.g.*, 2-carbonyl derived structures) are required for the effective reduction. Moreover, the dearomative functionalization using the Birch reduction has been particularly challenging. It mainly relies on trapping the *in situ* formed carbanions for alkylations.^6,7,17^ Clearly, a strategy which can streamline the dearomatization and simultaneous functionalization of simple heteroarenes can be immediately appreciated by synthetic and medicinal chemists by offering a rapid approach to medicinally valued, heavily functionalized 3-dimensional heterocyclics.^18^

Recently we have achieved the selective dearomative functionalization of benzene in polycyclic arenes;^19^ however, it is challenging to selectively dearomatize a single ring heteroarene, including thiophene/furan, which represents a long-standing issue.^1^ In order to address this issue, we envisioned that integrating photoredox catalysis into hydrogen atom transfer (HAT) could create a new strategy for direct dearomative functionalization of electron-rich heteroarenes (Scheme 1b and e). In light of the electron rich nature of aromatic structures, we conceived that single electron transfer (SET) oxidation of the system by an excited photocatalyst (PC*) could conveniently generate a radical cation (I, Scheme 1b and e).^20^ The reversed reactivity would make the nucleophilic addition more easy and generated an aromatic radical (II), a key species in Birch reduction, which then could undergo direct reduction by PC˙^−^ to give an anion. Subsequent protonation would give the functionalized dearomative product. Alternatively, engineering a new hydrogenation process [*e.g.*, hydrogen atom transfer (HAT)] could trap the radical (II) to directly furnish the functionalized dearomatization product. However, the intrinsic thermodynamic and kinetic favorable aromatization (*e.g.*, formation of V)^21–27^ makes this unprecedented dearomatization particularly difficult. Below, we describe the successful development of a general selective method for the dearomative functionalization of diverse heteroarenes. Moreover, unexpectedly, the dearomative ring-cleavage functionalization of thiophene/furan through selective C(sp^2^)–S/O bond breaking was discovered for the first time. The HAT process is crucial for overcoming the overwhelmingly favourable, irreversible aromatization.

## Results and discussion

Our studies started with the investigation of thiophenes since it has been a challenging target for dearomatization and their visible-light induced dearomatization has not been established,^1,28^ probably due to the high resonance stabilization (29 kcal mol^−1^).^29^ Given the easy oxidation of 2-methoxythiophene (1) (*E*^ox^: 1.44 V *vs.* SCE, Fig. S3†), we assessed the viability of the proposed dearomative functionalization protocol by reacting it with pyrazole (2) as a nucleophile in the presence of a photocatalyst (PC, 2.5 mol%) and a HAT agent (20 mol%) under blue LED irradiation (Table 1). A series of organic photocatalysts (entries 1–7) with oxidation potential larger than 1.44 V were selected for the investigation. Unexpectedly, the commercially available acridinium salts (Mes-Acr1-4) delivered a non-canonical dearomative ring-cleavage product thionoester 3 with two isolable *Z*/*E*-isomers (1 : 1 ratio, entries 1–4) through selective C(sp^2^)–S/O bond breaking, which is rarely achieved using the traditional ionic method.^16^Mes-Acr4 turned out to be the optimal one. Among 13 different hydrogen atom donors probed, benzeneselenol was proved to be the best one (entries 1, 8, and 9, and Table S2†). The solvent played the vital role in the reaction (Table S3†). Importantly, low concentrations improved the chemical transformation dramatically (entries 1 and 10–12). Finally, control experiments (entries 13–16) indicated that the base, HAT catalyst, and PC are essential for the reactions. It is noted that without a HAT agent, the dearomatization product with 23% yield was obtained (entry 13). This suggests that both pathways (paths a and b, Scheme 1c) are in operation, but the HAT pathway is kinetically favored presumably because the HAT can facilitate the process. Taken together, the optimal protocol has been established as follows: a mixture of 2-methoxythiophene (1.5 equiv.) and pyrazole (1.0 equiv.) in the presence of Mes-Acr4 (2.5 mol%), PhSeH (0.2 equiv.), and 2,6-lutidine (0.2 equiv.) in DCM (0.05 M) is under the irradiation of 40 W blue LEDs for 48 h giving the best reaction efficacy (77% yield, entry 1).

**Table 1:** Reaction condition optimization

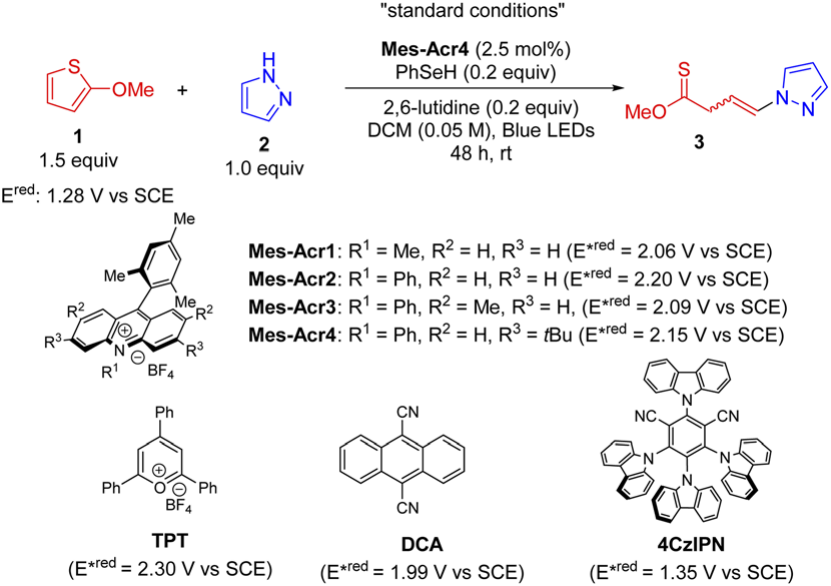		
Entry	Variation from the “standard conditions^a^”	Yield^b^ (%)
1	None	81 (77)^c^
2	Mes-Acr1 instead of Mes-Acr4	50
3	Mes-Acr2 instead of Mes-Acr4	42
4	Mes-Acr3 instead of Mes-Acr4	58
5	TPT instead of Mes-Acr4	Trace
6	DCA instead of Mes-Acr4	Trace
7	4CzIPN instead of Mes-Acr4	Trace
8	PhSH instead of PhSeH	56
9	4-NO_2_PhSH instead of PhSeH	38
10	DCM (0.025 M)	82
11	DCM (0.1 M)	60
12	DCM (0.2 M)	30
13	Without 2,6-lutidine	68
14	Without PhSeH	23
15	Without Mes-Acr4	Trace
16	No light	Trace

a Standard conditions: a mixture of 2-methoxythiophene (1.5 equiv.), pyrazole (1.0 equiv.), Mes-Acr4 (2.5 mol%), PhSeH (0.2 equiv.), and 2,6-lutidine (0.2 equiv.) in DCM (0.05 M) under the irradiation of 40 W blue LEDs for 48 hours at room temperature.

b Yield is determined by crude ^1^H NMR using 1,3,5-trimethoxybenzene as the internal reference.

c Isolated yield.

**Scheme 2:**
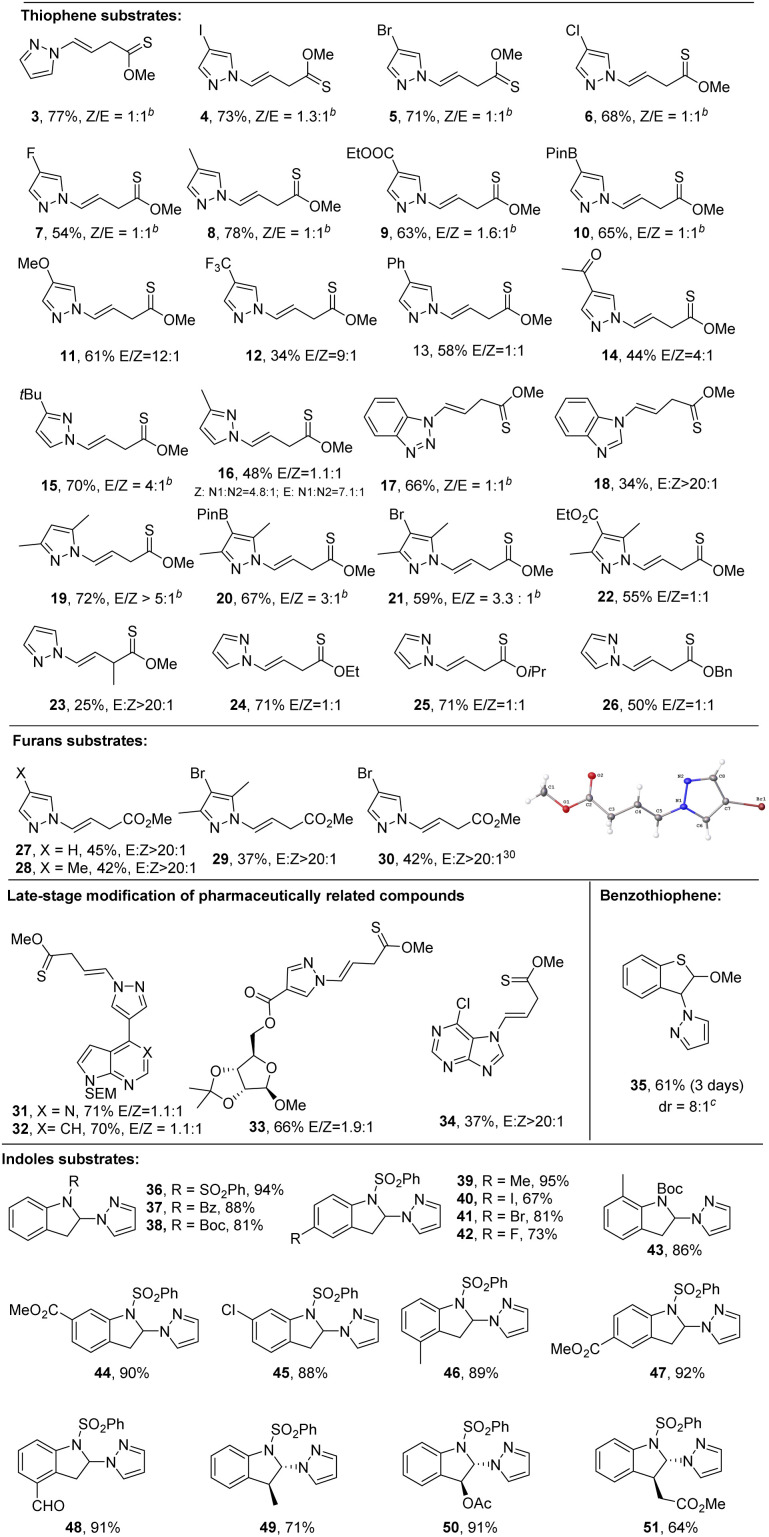
Scope of heteroarenes. ^*a*^ Reaction conditions, unless specified, see Table 1, entry 1 and ESI.† Yields are isolated yields. ^*b*^*E*/*Z* ratio was determined by crude ^1^H NMR. ^*c*^ Diastereoselectivity (dr) was determined by ^1^H NMR.

Having established the optimal reactions conditions, we explored the scope of the process with various heteroarenes including thiophenes, furans, benzothiophenes, and indoles (Scheme 2). The dearomative ring-cleavage functionalization of thiophenes using diverse pyrazoles was first investigated. Both electron donating and withdrawing groups are tolerated under the mild reaction conditions with moderate to good yield. They include iodide (4, 73%), bromide (5, 71%), chloride (6, 68%), fluoride (7, 54%), methyl (8, 78%; 16, 48%), *tert*-butyl (15, 70%), ester (9, 63%), boronic acid (10, 65%), methoxy (11, 61%), trifluoromethyl (12, 34%), phenyl (13, 58), ketone (14, 44%), disubstituted (19, 72%), trisubstituted (20, 67%; 21, 59%; 22, 55%), benzotriazole (17, 66%) and benzimidazole (18, 34%). Notably, boronic acid (10) can serve as a viable handle for cross coupling reactions in further synthetic elaboration. It appears that the Z/*E* selectivity is affected by the steric hindrance. A relatively high rr (>3 : 1) is observed with congested pyrazoles (15 and 19–21). In addition to monosubstituted thiophene, 2-methoxy, 3-methylthiophene (23, 25%) could also be dearomatized with reasonable yield. Notably exclusive *E*-configuration was formed in 23. Although low *Z*/*E*-selectivity was observed with thiophenes, 2-methoxyfuran (27–30) worked smoothly and delivered single *Z*-conformation.^30^ The simple 2-methoxythiophene could undergo the late-stage modification of complex molecules including pharmaceutically related heteroarenes (31, 71%; 32, 70%), ribose (33, 66%), and purine (34, 37%). As to polycyclic heteroarenes, benzothiophene (35) could selectively undergo dearomative non-cleavage hydroamination in the five-member ring at the 3-position, yielding 61% product with *trans* : *cis* = 8 : 1. Similarly, the simple indoles could selectively be dearomatized in the 2-position of the five member ring. Among three protecting groups (36–38), the sulfonamide delivered an excellent yield (94%). Diverse functional groups in the phenyl ring of indoles could be tolerated, such as methyl (39, 95%; 43, 86%; 46, 89%), halogens (40–42, 67–81%), ester (44, 92%), and aldehyde (48, 91%). Additionally, *trans*-products (49, 71%, 50, 91% and 51, 64%) were produced with 3-substituted indoles in high yields.

**Scheme 3:**
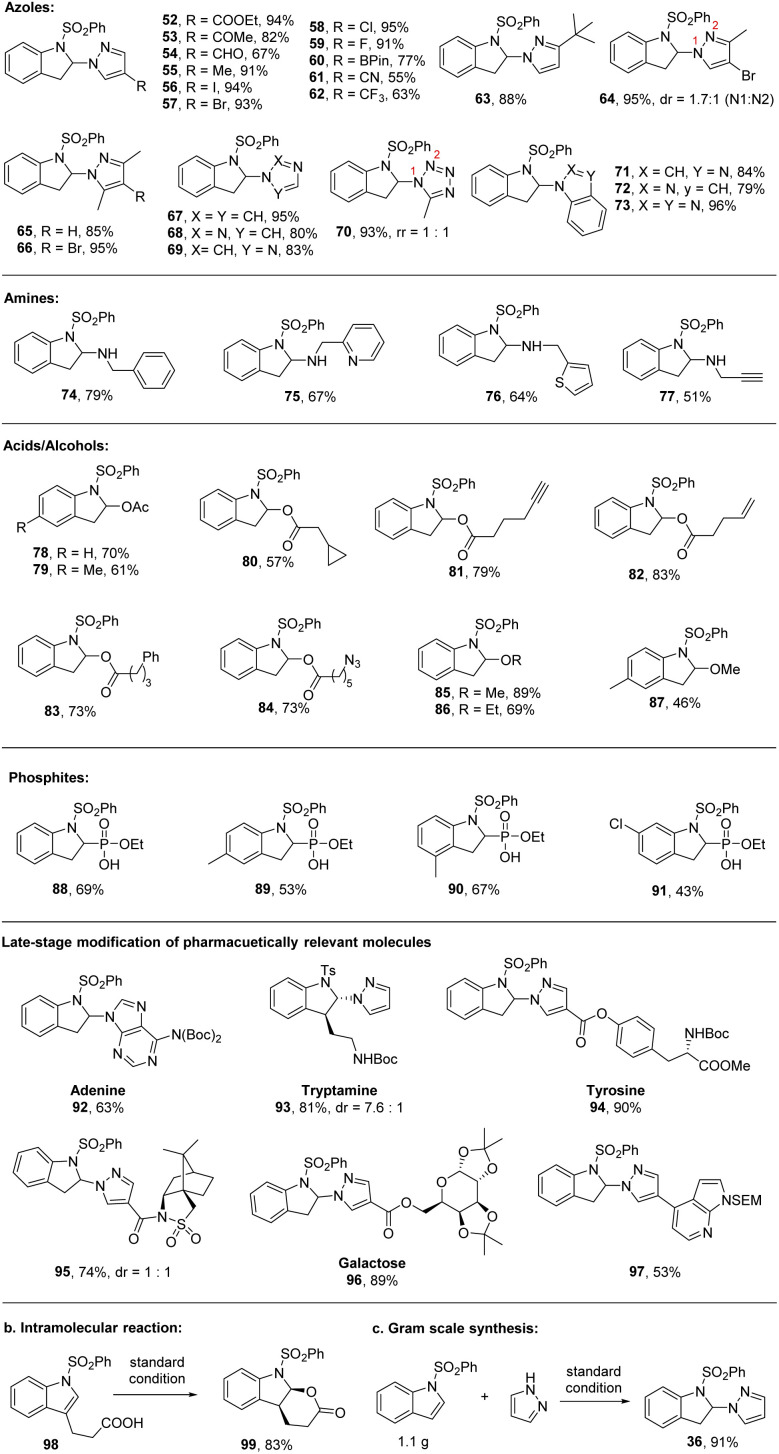
Scope of nucleophiles. Reaction conditions, unless specified, see Table 1, entry 1 and ESI.† Yields are isolated yields.

Visible light-mediated photochemical dearomatization of indoles has been reported recently.^31^ These methods are limited to intramolecular and [4 + 2]/[2 + 2] cycloaddition processes.^32–44^ In contrast, intermolecular dearomative functionalization remains underexplored.^45–47^ Herein, we found that the intermolecular photoredox catalyzed dearomatization strategy shows a broader scope with a wide array of nucleophiles including amines, various azoles, carboxylates, alcohols, and phosphites. As shown in Scheme 3, a number of functional groups tethered to pyrazoles can be tolerated including ester (52, 94%), ketone (53, 82%), aldehyde (54, 67%), methyl (55, 91%), halogen (56–59, 91–95%), boronic acid (60, 77%), nitrile (61, 55%), trifluoromethyl (62, 63%), *t*-butyl (63, 88%), disubstituted (64, 95%; 65, 85%), and trisubstituted (66, 95%). Aside from pyrazoles, other common azoles, including imidazole (67, 95%), 1,2,3-triazole (68, 80%), 1,2,4-triazole (69, 83%), tetrazole (70, 93%), benzoimidazole (71, 84%), indazole (72, 79%), and benzotriazole (73, 96%), are also amenable in good to excellent yield. Amines such as benzylamine (74, 79%), 2-picolylamine (75, 67%), 2-aminomethyl)thiophene (76, 64%), and propargylamine (77, 51%) are also viable nucleophiles for direct functional dearomatization. Furthermore, remarkably, the less nucleophilic O-nucleophiles including carboxylic acids (78–84) and alcohols (85–87) could also work smoothly. These transformations are compatible with a number of functional groups such as cyclopropyl (80, 57%), alkyne (81, 79%), alkene (82, 83%), phenyl (83, 73%), azide (84, 73%), methanol (85, 89%; 87, 46%), and ethanol (86, 69%). Moreover, triethyl phosphite (88–91) was a validated nucleophilic agent, giving 2,3-dihydro-1*H*-indol-2-ylphosphonic acid in good yields (75–78%). The mild reaction conditions also enabled the late-stage modification of pharmaceutically relevant molecules such as aldenine (92, 63%), tryptamine (93, 81%), tyrosine (94, 90%), Oppolzer's camphorsultam (95, 74%), galactose (96, 89%), and 4-(1*H*-pyrazol-4-yl)-7*H*-pyrrolo[2,3-*d*]pyrimidine (97, 53%). In addition to intermolecular dearomatization, the intramolecular reaction could also work efficiently under the standard conditions, delivering the cyclized product (99, 83%, Scheme 3b). The reaction can be scaled up without loss of yield (Scheme 3c). These results clearly demonstrate the mildness, high regioselectivity, and broad generality of the newly developed synthetic manifold.

**Scheme 4:**
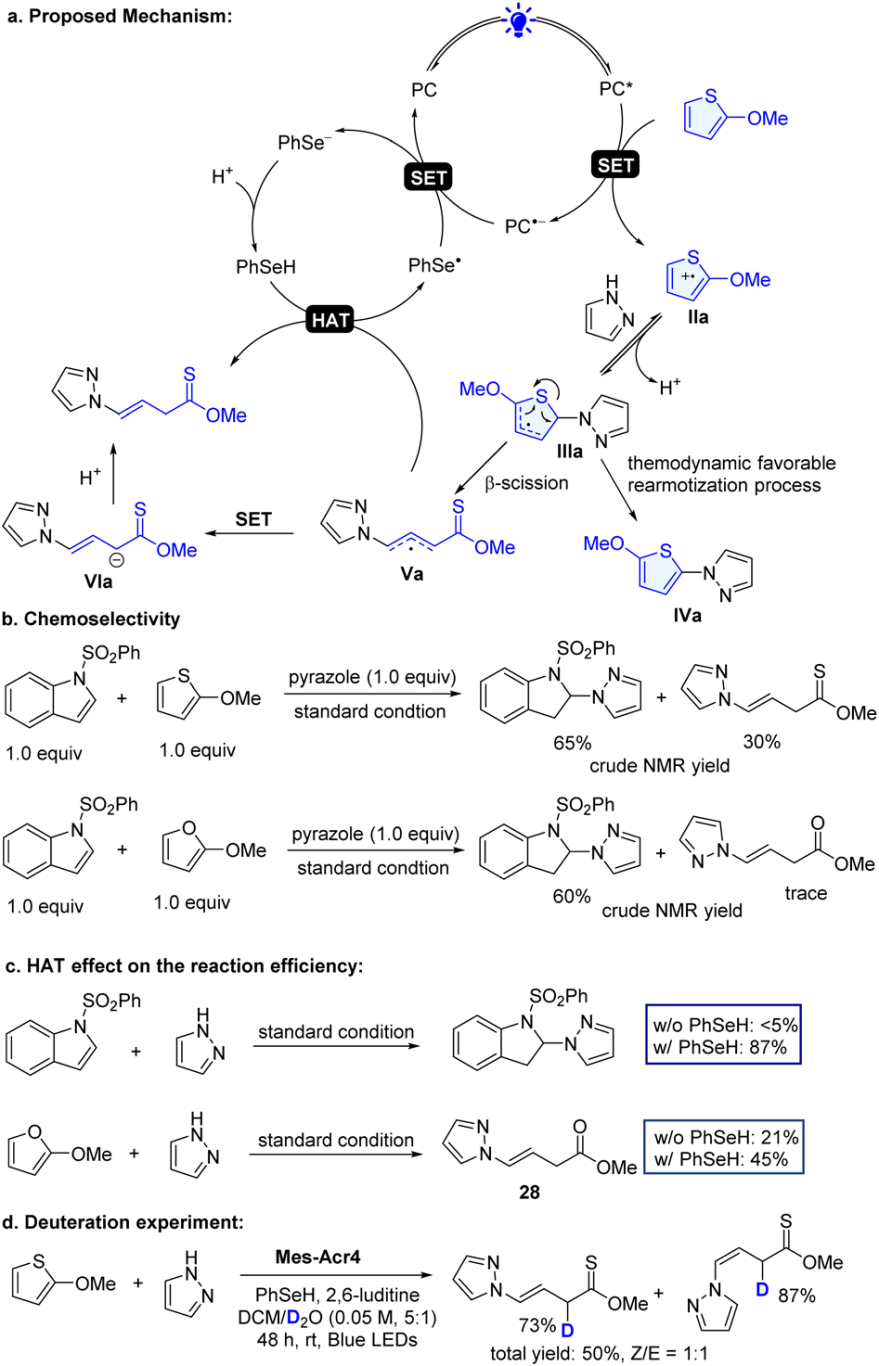
Proposed mechanism and designed experiments for mechanistic studies.

In the photoredox dearomative functionalization reaction, one of the important steps is the selective SET oxidation of heteroarenes. Although visible light mediated SET oxidation of arenes including indoles in organic transformations has been reported,^21–27,31,48–56^ this study represents the first example of oxidation of thiophenes, furans and benzothiophenes. The excited state PC^+^* Mes-Acr4* possesses strong oxidizing power (*E*^red^* = 2.15 V)^57^ and can oxidize these substrates (2-methoxythiophene: *E*^ox^ = 1.44 V; 2-methoxyfuran: *E*^ox^ = 1.28 V, and *N*-SO_2_Ph indole: *E*^ox^ = 1.90 V *vs.* SCE, Fig. S1–S3†) in principle (Scheme 4a using 2-methoxythiophene as an illustration example). We found that although the indole had high oxidation potential, the reaction occurred with it favorably in the mixture of indole and 2-methoxythiophene (Scheme 4b). A similar trend was observed with 2-methoxyfuran. Furans and thiophenes have higher resonance stabilization than indoles.^29^

Additional energy is needed to cleave the heteroarene ring bond, which was usually achieved *via* transitional-metal-catalyzed C–S insertion.^58^ These factors may pose a challenge for dearomative ring-opening functionalization of simple thiophenes and furans with photoredox catalysis. The electrophilic radical cation IIa reacts with pyrazole followed by subsequent deprotonation to form the radical IIIa (Scheme 4a), which readily undergoes the ring-opening β-scission and generate radical Va. The radical Va can abstract a hydrogen atom from PhSeH or undergoes the SET process, providing a non-classic dearomative ring-cleavage product. On the other hand, the radical IIIa can undergo thermodynamically favorable rearomatization^20–27^ to give functionalized aromatic product IVa. However, the application of the photoredox C–H functionalization of these heteroarenes has not been reported. In our effort, we further challenged the chemistry by redirecting to an overwhelmingly unfavorable dearomatized product. We found that the incorporation of a new HAT process can significantly inhibit the aromatized process and facilitate the dearomative product (Table 1, entries 1 and 14). The HAT agents are crucial for the process. Without it, only a small amount of product was formed (entry 14 in Table 1). In addition, the choice of HAT agents is also important. Selenol with a higher H-atom transfer rate (*K*_20_ = 1.3 × 10^9^ M^−1^ s^−1^*vs.* PhSH: *K*_20_ = 9.0 × 10^7^ M^−1^ s^−1^)^59–61^ gives the desired product in a better yield (entries 8 and 14 in Table 1). Furthermore, control experiments further validated the vital role of the HAT agent. In the absence of PhSeH, the standard conditions failed to deliver the dearomatized indole product (Scheme 4c). The lack of the HAT agent also significantly compromises the reaction efficacy in the reaction of 2-methoxyfuran. Finally, the deuteration experiment consolidates the HAT process engaged in the reaction (Scheme 4d). Taken together, these studies showed that the HAT is an important factor contributing to the success of the new dearomatization process.

## Conclusions

In summary, we have developed a conceptually different approach for the dearomatization of challenging electron rich heteroarenes. Distinct from the widely used reductive dearomatization methods, we have combined photoredox catalysis and HAT chemistry for dearomatization and simultaneous functionalization of heteroarenes. Moreover, the success of the transformation relies on implementing a HAT, which directs towards dearomatization rather than the thermo-dynamically favorable aromatization. Especially, we first discovered the photoredox catalyzed dearomative ring-cleavage functionalization of thiophenes and furans. The mild protocol enables broad substrate scope of heteroarenes such as thiophenes, furans, benzothiophenes, and indoles and nucleophiles including pharmaceutically valued azoles, amines, carboxylic acids, alcohols, and phosphites amenable to the process and the late-stage modification of pharmaceutically valued molecules. It is expected that high efficiency of this methodology will be appreciated by organic chemists and medicinal chemists to rapidly access a library of heavily functionalized, biologically valued heterocycles.

## Author contributions

P. J., X. M., J. C., F. G., and H. X. conducted and analyzed the synthetic experiments. W. W. planned, designed and directed the project. P. J. and W. W. wrote the manuscript.

## Conflicts of interest

There are no conflicts to declare.

## Supplementary Material

SC-014-D3SC00060E-s001

SC-014-D3SC00060E-s002
