# Supplementary material for: Combining photoredox catalysis and hydrogen atom transfer for dearomative functionalization of electron rich heteroarenes
Source: Chem Sci. 2023 Feb 28;14(12):3332–7. doi: 10.1039/d3sc00060e (PMC10034082; doi:10.1039/d3sc00060e)
Supplement: SC-014-D3SC00060E-s001 [file SC-014-D3SC00060E-s001.pdf]

Electronic Supplementary Material (ESI) for Chemical Science.
This journal is © The Royal Society of Chemistry 2023

Supplementary Materials

Combining Photoredox Catalysis and Hydrogen Atom Transfer for Dearomative Functionalization of Electron Rich Heteroarenes

Peng Ji,[1] Xiang Meng,[1] Jing Chen,[1] Feng Gao,[1] Hang Xu,[1] and Wei Wang[1,2,3*]

[1]*Department of Pharmacology and Toxicology, R. Ken Coit College of Pharmacy*
[2]*Chemistry and Biochemistry, and* [3]*University of Arizona Cancer Center*
University of Arizona, Tucson, AZ 85721-0207, USA

Table of Contents

1. General Information S2
2. General Procedures and Optimization of Reaction Conditions S2
3. Measurement of redox potential S9
4. Compounds Characterization Data S11
5. Spectral Data S50
6. Single crystal X-ray diffraction data of **30** S171
7. References S177

1. General Information

All commercially available reagents were purchased from Sigma Aldrich, Ark Pharm Inc., Matrix Chemical, Combi-Blocks, AKSci, Alfa Aesar, Acros, Ambeed or TCI America, and used as received unless otherwise noted. Merck 60 silica gel was used for chromatography, and Whatman silica gel plates with a fluorescence F254 indicator were used for thin-layer chromatography (TLC) analysis. **^{1}H** and **^{13}C NMR** spectra were recorded on Bruker Avance 500 MHz or Varian 400 MHz. Chemical shifts in **^{1}H NMR** spectra are reported in parts permillion (ppm) relative to residual chloroform (7.26 ppm) or dimethyl sulfoxide (2.50 ppm) as internal standards. ^{1}H NMR data are reported as follows: chemical shift (δ ppm), multiplicity (s = singlet, d = doublet, quint = quintet, sext = sextet, m = multiplet, br = broad), coupling constant in Hertz (Hz) and hydrogen numbers based on integration intensities. **^{13}C NMR** chemical shifts are reported in ppm relative to the central peak of $CDCl_3$ (77.16 ppm) or DMSO-d_6 (39.52 ppm) as internal standards. High-resolution mass spectrometry was performed in Analytical and Biological Mass Spectrometry Center. Both blue LED strip and 40 W Kessil Blue LEDs were purchased from Amazon. Cyclic voltammetry was performed at 25 °C on a CH Instrument CHI604xD electrochemical analyzer using a glassy carbon working electrode, a platinum wire counter electrode, and the Ag/AgCl reference electrode calibrated using ferrocene redox couple (4.8 V below vacuum). HPLC analyses with chiral stationary phase were carried out using a SHIMADZU HPLC system with SPD-20A detector. Samples are purified by flash column chromatography on Teledyne ISCO CombiFlash NextGen300+.

2. General Procedures and Optimization of Reaction Conditions

2.1 General procedure A (3-34)

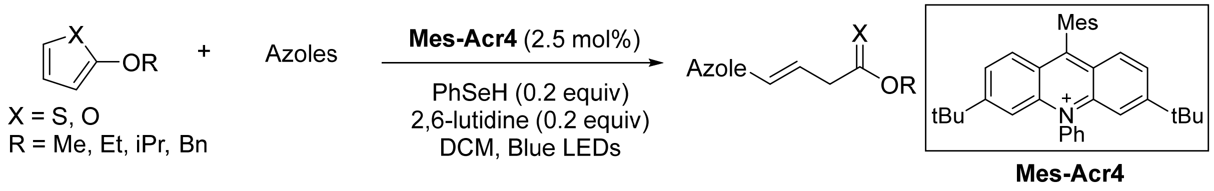

To an oven-dried 20 mL-Schlenk tube equipped with a stir bar, was added nucleophiles (0.2 mmol), arenes (0.3 mmol), PhSeH (0.04 mmol), **Mes-Acr-4** (0.005 mmol), 2,6-lutidine (0.04 mmol) as the base, will be added. The tube was evacuated and back-filled with N_2 for three times, then sealed with rubber stopper and parafilm. Subsequently, the degassed dichloromethane (4 mL) was added. The reaction was irradiated by the two 40 W Kessil Blue LEDs, cooling by the electronic fan (Figure S1). After the completion of reactions, the resulted solution was purified by flash column chromatography on silica gel eluting with hexane/ethyl acetate or DCM/ethyl acetate in the indicated ratio.

2.2 General procedure B (36-73)

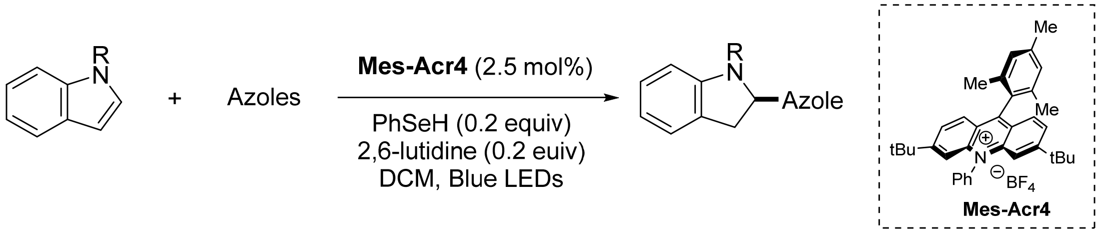

To an oven-dried 20 mL-Schlenk tube equipped with a stir bar, was added azoles (0.24 mmol), indole (0.2 mmol), PhSeH (0.04 mmol), **Mes-Acr-4** (0.005 mmol), 2,6-lutidine (0.04 mmol) as the base, will be added. The tube was evacuated and back-filled with N_2 for three times, then sealed with rubber stopper and parafilm. Subsequently, the degassed dichloromethane (4 mL) was added. The reaction was irradiated by the two 40 W Kessil Blue LEDs, cooling by the electronic fan (Figure S1). After the completion of reactions, the resulted solution was purified by flash column chromatography on silica gel eluting with hexane/ethyl acetate or DCM/ethyl acetate in the indicated ratio.

2.3 General procedure C (74-71, 88-91)

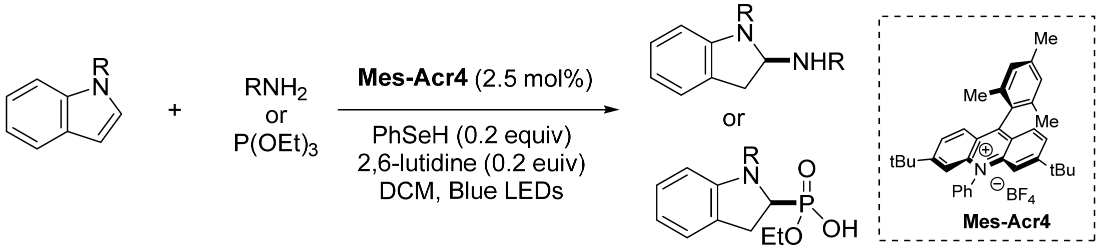

To an oven-dried 20 mL-Schlenk tube equipped with a stir bar, was added amine or $P(OEt)_3$ (0.4 mmol), indole (0.2 mmol), PhSeH (0.04 mmol), **Mes-Acr-4** (0.005 mmol), 2,6-lutidine (0.04 mmol) as the base, will be added. The tube was evacuated and back-filled with N_2 for three times, then sealed with rubber stopper and parafilm. Subsequently, the degassed dichloromethane (4 mL) was added. The reaction was irradiated by the two 40 W Kessil Blue LEDs, cooling by the electronic fan (Figure S1). After the completion of reactions, the resulted solution was purified by flash column chromatography on silica gel eluting with hexane/ethyl acetate or DCM/ethyl acetate in the indicated ratio.

2.4 General procedure D (78-87)

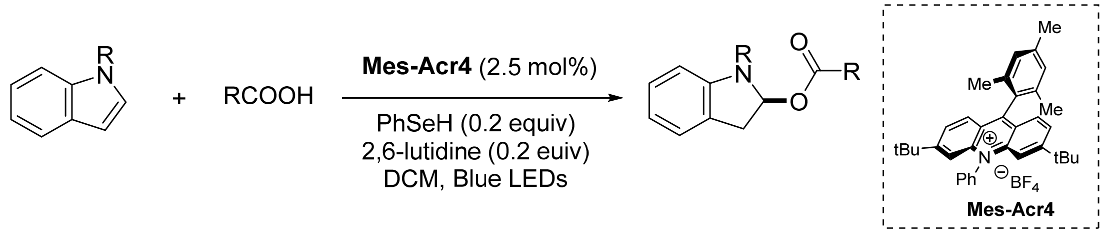

To an oven-dried 20 mL-Schlenk tube equipped with a stir bar, was added carboxylic acids (0.6 mmol) or acetic acid (5.0 equiv), indole (0.2 mmol), PhSeH (0.04 mmol), **Mes-Acr-4** (0.005 mmol), 2,6-lutidine (0.04 mmol) as the base, will be added. The tube was evacuated and back-filled with N_2 for three times, then sealed with rubber stopper and parafilm. Subsequently, the degassed dichloromethane (4 mL) was added. The reaction was irradiated by the two 40 W Kessil Blue LEDs, cooling by the electronic fan (Figure S1). After the completion of reactions, the resulted solution was purified by flash column chromatography on silica gel eluting with hexane/ethyl acetate or DCM/ethyl acetate in the indicated ratio.

2.5 General procedure E (85-87)

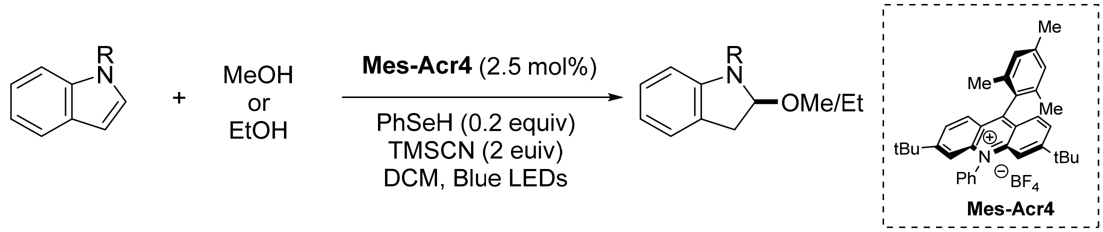

To an oven-dried 20 mL-Schlenk tube equipped with a stir bar, was added indole (0.2 mmol), PhSeH (0.04 mmol), **Mes-Acr-4** (0.005 mmol), TMSCN (0.4 mmol). The tube was evacuated and back-filled with N_2 for three times, then sealed with rubber stopper and parafilm. Subsequently, the degassed MeOH or EtOH (4 mL) was added. The reaction was irradiated by the two 40 W Kessil Blue LEDs for 48 hours, cooling by the electronic fan (Figure S1). After the completion of reactions, the resulted solution was purified by flash column chromatography on silica gel eluting with hexane/ethyl acetate or DCM/ethyl acetate in the indicated ratio.

2.6 Intramolecular reaction

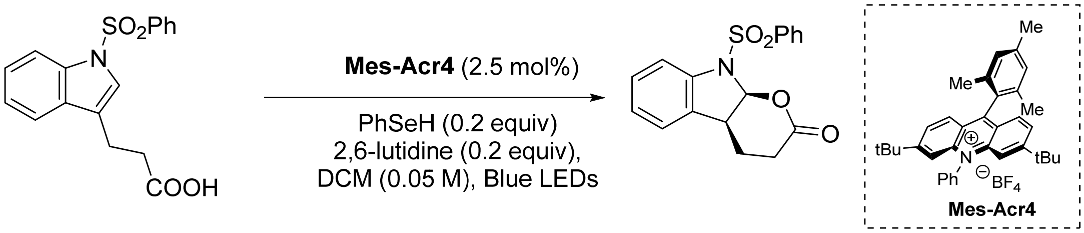

To an oven-dried 20 mL-Schlenk tube equipped with a stir bar, was added indole acid (0.2 mmol), PhSeH (0.04 mmol), **Mes-Acr-4** (0.005 mmol), 2,6-lutidine (0.04 mmol). The tube was evacuated and back-filled with N_2 for three times, then sealed with rubber stopper and parafilm. Subsequently, the degassed DCM (4 mL) was added. The reaction was irradiated by the two 40 W Kessil Blue LEDs for 48 hours, cooling by the electronic fan (Figure S1). After the completion of reactions, the resulted solution was purified by flash column chromatography on silica gel eluting with hexane/ethyl acetate or DCM/ethyl acetate in the indicated ratio.

2.7 Large scale synthesis 36

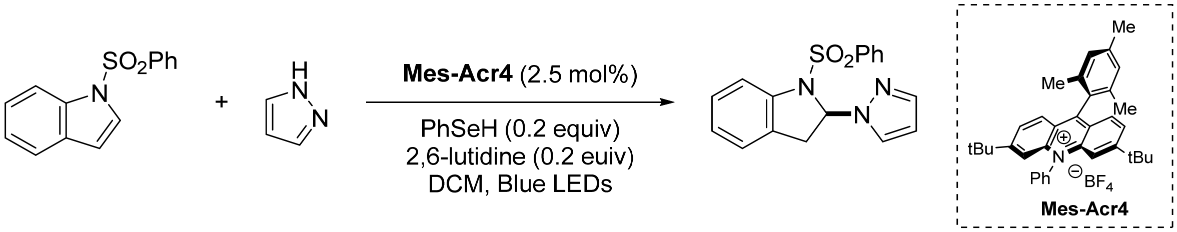

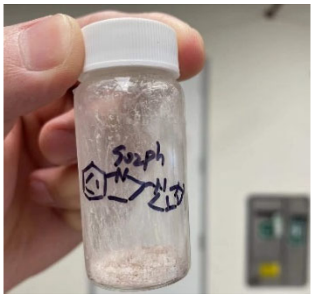

To an oven-dried 20 mL-Schlenk tube equipped with a stir bar, was added pyrazole (349 mg, 5.13 mmol) (5.0 equiv), indole (1.1 g, 4.28 mmmol), PhSeH (91 uL, 0.86 mmol), **Mes-Acr-4** (61 mg, 0.11 mmol), 2,6-lutidine (66 uL, 0.11 mmol) as the base, will be added. The tube was evacuated and back-filled with N_2 for three times, then sealed with rubber stopper and parafilm. Subsequently, the degassed dichloromethane (4 mL) was added. The reaction was irradiated by the two 40 W Kessil Blue LEDs, cooling by the electronic fan. After the completion of reactions, the resulted solution was purified by flash column chromatography on silica gel eluting with hexane/ethyl acetate (5:1) in the indicated ratio.

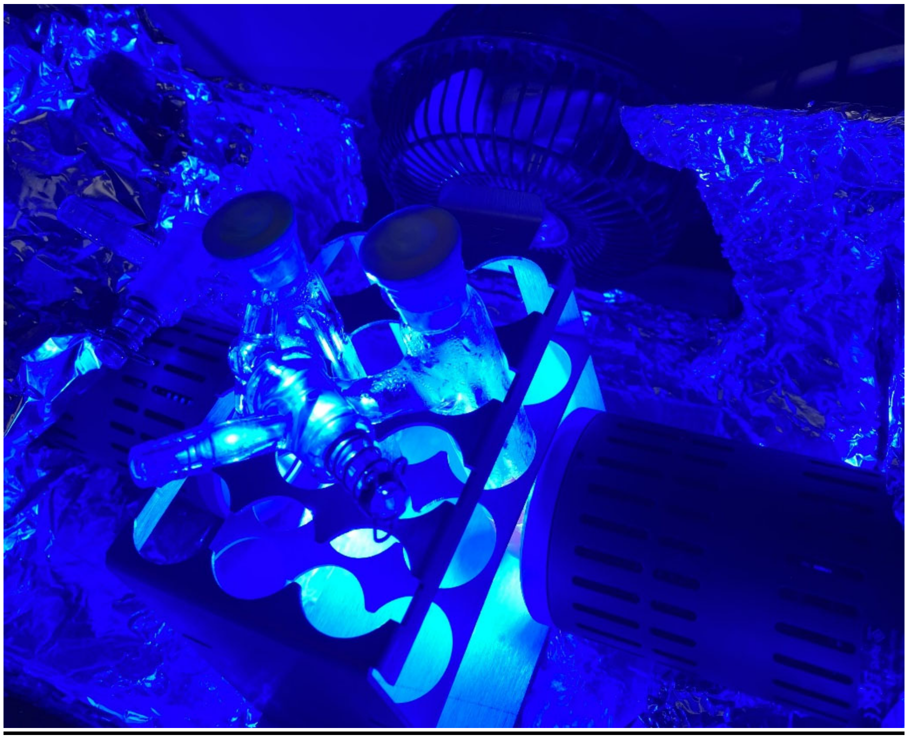

Figure S1. Reaction setup

2.8 reaction conditions optimization

Table S1. Optimization of different photocatalyst.

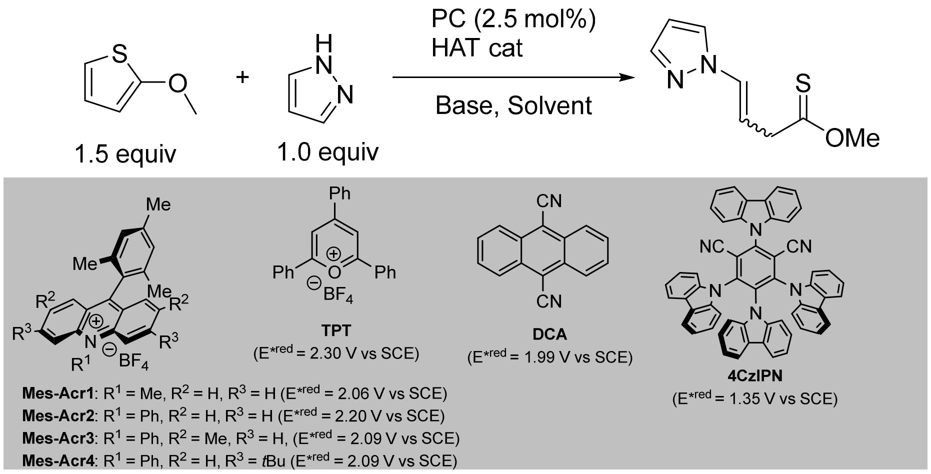

PC (2.5 mol%)	Base (0.2 equiv)	HAT cat (0.2 equiv)	Solvent	Con (M)	Yield	Z/E
**Mes-Acr2**	2,6-lutidine	PhSeH	DCM	**0.05**	**42**	**1:1**
**Mes-Acr4**	2,6-lutidine	PhSeH	DCM	**0.05**	**81**	**1:1**
**Mes-Acr3**	2,6-lutidine	PhSeH	DCM	**0.05**	**58**	**1:1**

**Mes-Acr1**	2,6-lutidine	PhSeH	DCM	**0.05**	**50**	**1:1**
**4CzIPN**	2,6-lutidine	PhSeH	DCM	**0.05**	**trace**	**-**
**DCA**	2,6-lutidine	PhSeH	DCM	**0.05**	**trace**	**-**
**TPT**	2,6-lutidine	PhSeH	DCM	**0.05**	**trace**	**-**

Table S2. Optimization of HAT agents

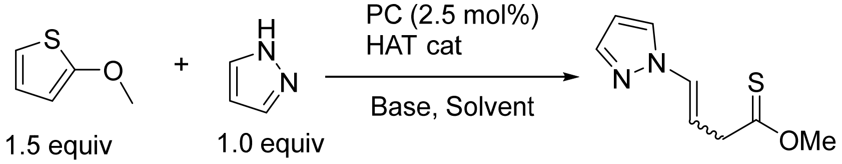

PC (2.5 mol%)	Base (0.2 equiv)	HAT cat (0.2 equiv)	Solvent	Con (M)	Yield	E/Z
**Mes-Acr4**	2,6-lutidine	PhSeH	DCM	0.05	81	1:1
**Mes-Acr4**	2,6-lutidine	Ph_3SiSH	DCM	0.05	60	2:1
**Mes-Acr4**	2,6-lutidine	iPr_3SiSH	DCM	0.05	57	1.2:1
**Mes-Acr4**	2,6-lutidine	PhSH	DCM	0.05	56	2.7:1
**Mes-Acr4**	2,6-lutidine	TRIPSH	DCM	0.05	54	1:1
**Mes-Acr4**	2,6-lutidine	4-NO_2PhSH	DCM	0.05	38	6.6:1
**Mes-Acr4**	2,6-lutidine	4-MeOPhSH	DCM	0.05	63	1:1
**Mes-Acr4**	2,6-lutidine	Cysteine	DCM	0.05	55	1.6:1
**Mes-Acr4**	2,6-lutidine	3,6-diCl-1,2-PhSH	DCM	0.05	53	1.2:1
**Mes-Acr4**	2,6-lutidine	2-NH_2PhSe	DCM	0.05	33	2.9:1
**Mes-Acr4**	2,6-lutidine	$SHCH_2COOMe$	DCM	0.05	56	2.7:1
**Mes-Acr4**	2,6-lutidine	Adman-SH	DCM	0.05	63	1:1
**Mes-Acr4**	2,6-lutidine	PhCOSH	DCM	0.05	56	2.1:1

Table S3. Optimization of Solvent.

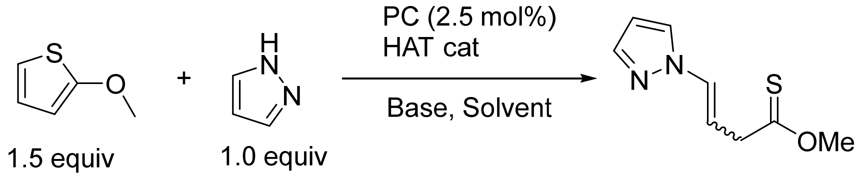

PC (2.5 mol%)	Base (0.2 equiv)	HAT cat (0.2 equiv)	Solvent	Con (M)	Yield
**Mes-Acr4**	2,6-lutidine	PhSeH	DCE	0.05	63
**Mes-Acr4**	2,6-lutidine	PhSeH	DMF	0.05	trace
**Mes-Acr4**	2,6-lutidine	PhSeH	ACN	0.05	16
**Mes-Acr4**	2,6-lutidine	PhSeH	$PhCF_3$	0.05	57

Table S4. Optimization of concentration and control studies.

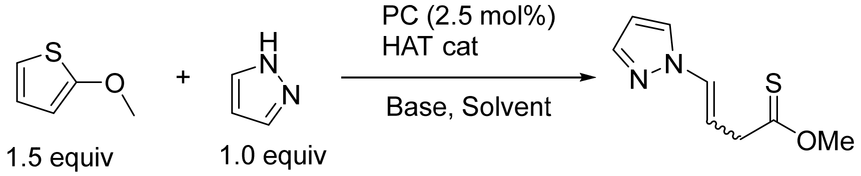

PC (2.5 mol%)	Base (0.2 equiv)	HAT cat (0.2 eq)	Solvent	Con (M)	Yield	E/Z
**Mes-Acr4**	2,6-lutidine	PhSeH	DCM	0.05	81	1:1
**Mes-Acr4**	2,6-lutidine	PhSeH	DCM	0.1	60	3:1
**Mes-Acr4**	2,6-lutidine	PhSeH	DCM	0.2	30	2:1
**Mes-Acr4**	none	PhSeH	DCM	0.05	68	1:1.4
**Mes-Acr4**	2,6-lutidine	none	DCM	0.05	23	1:1
**Mes-Acr4**	2,6-lutidine	PhSeH	DCM	0.05	trace	No light
**Mes-Acr4**	2,6-lutidine	PhSeH	DCM	0.05	trace	No PC

3. Measurement of redox potential

Procedure for measurement of the redox potential:

Voltammetric measurements were recorded on a CH Instruments: Model 600E Series Electrochemical Analyzer using a standard three electrodes setup in dry and degassed MeCN (10 mL), with ferrocene as an internal reference ($E^0_{1/2}$ = + 0.40 V vs SCE) and nBu$_4$NPF$_6$ as the electrolyte (0.10 mmol). Cyclic voltammograms were recorded at a scan rate of 0.2 V/s.

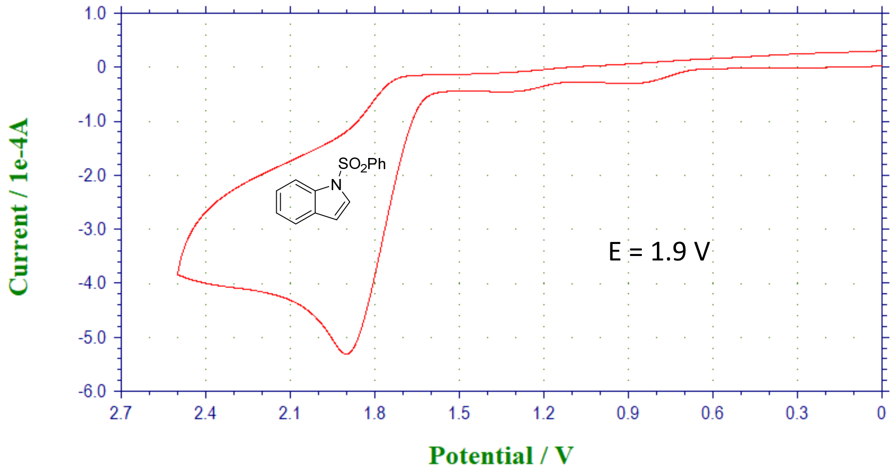

Figure S2. Redox potential of indole.

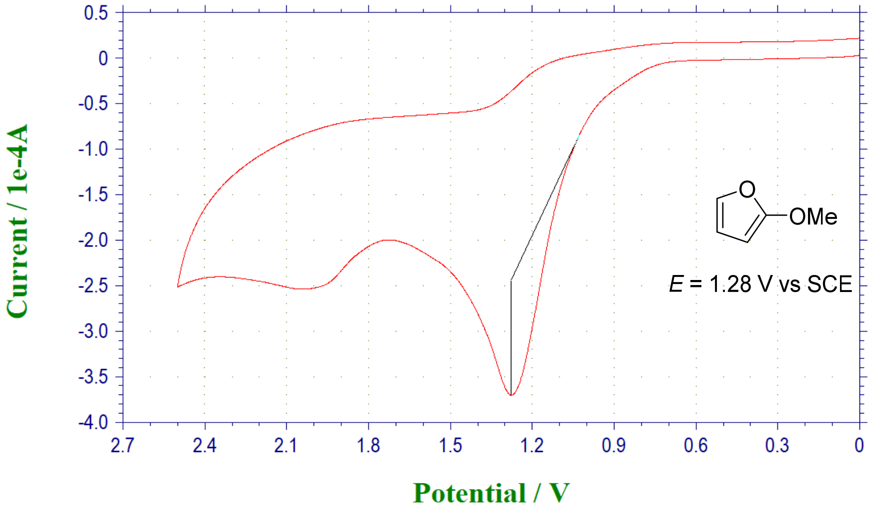

Figure S3. Redox potential of furan.

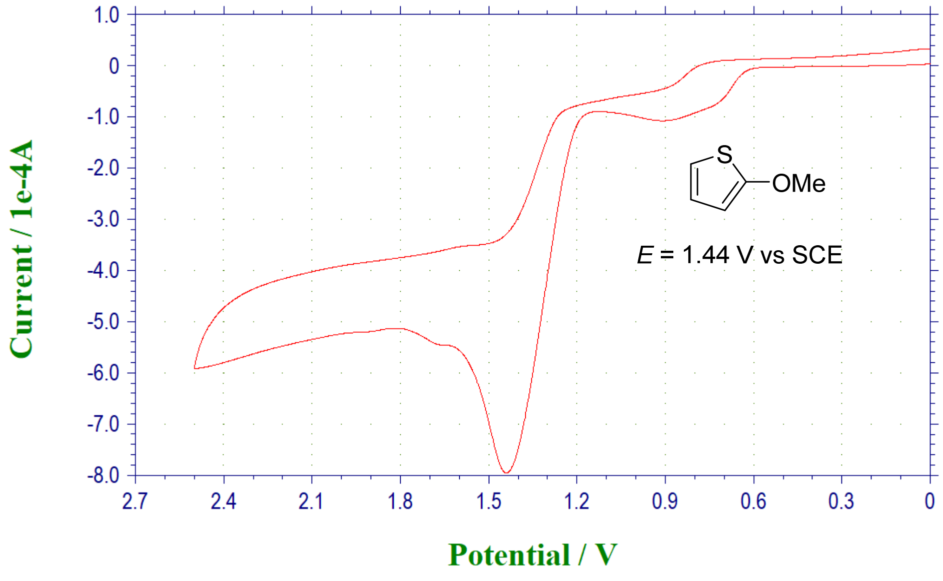

Figure S4. Redox potential of 2-methoxylthiophene.

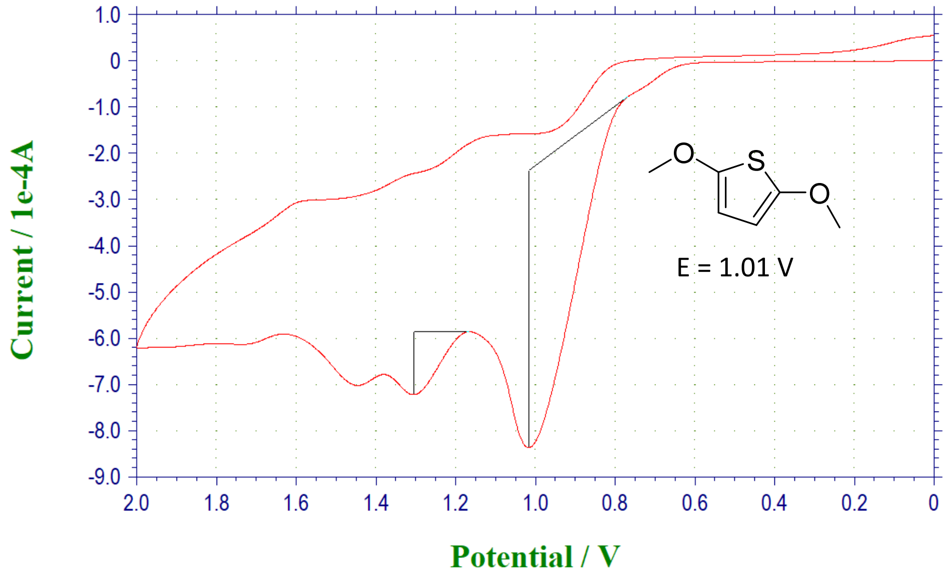

Figure S5. Redox potential of 2,5-dimethoxylthiophene.

4. Compounds Characterization Data

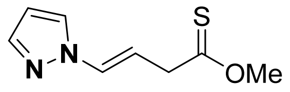

***O*-methyl (*E*)-4-(1*H*-pyrazol-1-yl)but-3-enethioate (3)**: The title compound was prepared according to the general procedure A. The crude residue was purified by column chromatography on silica gel with an eluent of hexanes/ethyl acetate (2:1) to furnish the pure compound as a yellow oil in 76% yield.

^{1}H NMR (400 MHz, $CDCl_3$) δ 7.60 (d, *J* = 1.8 Hz, 1H), 7.58 (d, *J* = 2.4 Hz, 1H), 6.94 (dt, *J* = 14.1, 1.4 Hz, 1H), 6.34 (t, *J* = 2.1 Hz, 1H), 6.25 (dt, *J* = 14.1, 7.6 Hz, 1H), 4.10 (s, 3H), 3.59 (dd, *J* = 7.6, 1.4 Hz, 2H).

^{13}C NMR (101 MHz, $CDCl_3$) δ 220.61, 140.85, 129.41, 127.56, 111.38, 107.00, 59.29, 46.68.

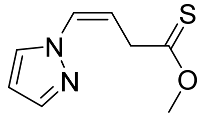

***O*-methyl (*Z*)-4-(1*H*-pyrazol-1-yl)but-3-enethioate (3)**:

^{1}H NMR (400 MHz, $CDCl_3$) δ 7.62 (d, J = 1.8 Hz, 1H), 7.56 (d, J = 2.6 Hz, 1H), 6.85 (dt, J = 9.4, 1.9 Hz, 1H), 6.33 (t, J = 2.1 Hz, 1H), 5.61 (dt, J = 9.4, 7.1 Hz, 1H), 4.12 (s, 3H), 4.09 (dd, J = 7.1, 1.8 Hz, 2H).

^{13}C NMR (101 MHz, $CDCl_3$) δ 221.55, 140.95, 130.53, 126.56, 113.79, 106.46, 59.32, 44.10.

HRMS (ESI) m/z $[M + H]^+$ calculated for $C_8H_{10}N_2OS$: 183.0587, found 183.0585.

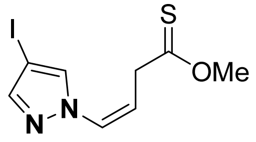

***O*-methyl (*Z*)-4-(4-iodo-1*H*-pyrazol-1-yl)but-3-enethioate (4)**: The title compound was prepared according to the general procedure A. The crude residue was purified by column chromatography on silica gel with an eluent of hexanes/ethyl acetate (2:1) to furnish the pure compound as a yellow oil in 76% yield.

^{1}H NMR (500 MHz, $CDCl_3$) δ 7.611 (s, 1H), 7.607 (s, 1H), 6.792 (dd, *J* = 9.3, 2.0 Hz, 1H), 5.647 (dt, *J* = 9.5, 7.2 Hz, 1H), 4.114 (s, 3H), 4.008 (dd, *J* = 7.2, 1.8 Hz, 2H).

^{13}C NMR (126 MHz, $CDCl_3$) δ 219.35, 145.80, 134.67, 125.97, 115.21, 59.36, 43.90, 29.55.

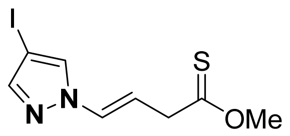

***O*-methyl (*E*)-4-(4-iodo-1*H*-pyrazol-1-yl)but-3-enethioate (4)**:

^{1}H NMR (500 MHz, $CDCl_3$) δ 7.65 (s, 2H), 7.58 (s, 1H), 6.88 (d, *J* = 14.1 Hz, 1H), 6.23 (dt, *J* = 14.1, 7.6 Hz, 2H), 4.10 (s, 5H), 3.57 (dd, *J* = 7.5, 1.4 Hz, 4H).

^{13}C NMR (126 MHz, $CDCl_3$) δ 220.09, 145.64, 131.78, 128.80, 112.51, 59.29, 46.31, 29.70.

HRMS (ESI) m/z $[M + H]^+$ calculated for $C_8H_9IN_2OS$: 308.9553, found 308.9552.

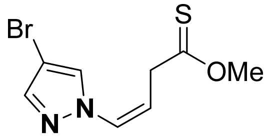

***O*-methyl (*Z*)-4-(4-bromo-1*H*-pyrazol-1-yl)but-3-enethioate (5):**

^{1}H NMR (500 MHz, $CDCl_3$) δ 7.59 (s, 1H), 7.57 (s, 1H), 6.77 (dt, *J* = 9.4, 1.9 Hz, 1H), 5.66 (dt, *J* = 9.3, 7.2 Hz, 1H), 4.11 (s, 3H), 4.00 (dd, *J* = 7.2, 1.8 Hz, 2H).

^{13}C NMR (126 MHz, $CDCl_3$) δ 220.82, 141.37, 130.31, 126.16, 115.21, 59.39, 43.84, 29.85.

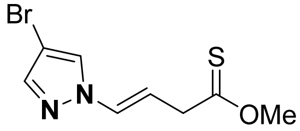

***O*-methyl (*E*)-4-(4-bromo-1*H*-pyrazol-1-yl)but-3-enethioate (5):**

^{1}H NMR (500 MHz, $CDCl_3$) δ 7.63 (s, 1H), 7.54 (s, 1H), 6.97 – 6.71 (m, 1H), 6.22 (dt, *J* = 14.6, 7.6 Hz, 1H), 4.10 (s, 3H), 3.57 (dd, *J* = 7.6, 1.4 Hz, 2H).

^{13}C NMR (126 MHz, $CDCl_3$) δ 220.23, 141.38, 129.16, 127.54, 112.53, 59.46, 46.43, 29.85.

HRMS (ESI) m/z $[M + H]^+$ calculated for $C_8H_9BrN_2OS$: 260.9692, found 260.9690.

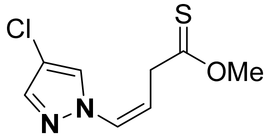

***O*-methyl (*Z*)-4-(4-chloro-1*H*-pyrazol-1-yl)but-3-enethioate (6):**

^{1}H NMR (500 MHz, $CDCl_3$) δ 7.57 (s, 1H), 7.53 (s, 1H), 6.74 (d, *J* = 9.3 Hz, 1H), 5.66 (dt, *J* = 9.3, 7.2 Hz, 1H), 4.11 (s, 3H), 4.00 (dd, *J* = 7.2, 1.8 Hz, 2H).

^{13}C NMR (126 MHz, $CDCl_3$) δ 220.84, 139.31, 128.10, 126.26, 115.11, 59.39, 43.83, 33.28.

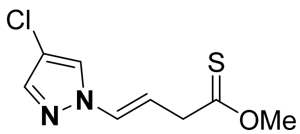

***O*-methyl (*E*)-4-(4-chloro-1*H*-pyrazol-1-yl)but-3-enethioate (6):**

^{1}H NMR (500 MHz, $CDCl_3$) δ 7.60 (s, 1H), 7.51 (s, 1H), 6.84 (d, *J* = 14.2 Hz, 1H), 6.20 (dt, *J* = 14.1, 7.6 Hz, 1H), 4.10 (s, 4H), 3.56 (dd, *J* = 7.6, 1.4 Hz, 3H).

^{13}C NMR (126 MHz, $CDCl_3$) δ 220.25, 139.33, 129.31, 125.28, 112.40, 59.45, 46.43, 31.07.

HRMS (ESI) m/z $[M + H]^+$ calculated for $C_8H_9ClN_2OS$: 217.0197, found 217.0194.

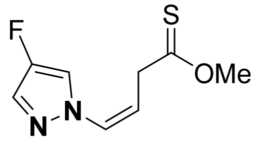

***O*-methyl (*Z*)-4-(4-fluoro-1*H*-pyrazol-1-yl)but-3-enethioate (7):**

^{1}H NMR (500 MHz, $CDCl_3$) δ 7.48 (d, *J* = 4.7 Hz, 1H), 7.47 (d, *J* = 3.8 Hz, 1H), 6.69 (dt, *J* = 9.4, 1.9 Hz, 1H), 5.63 (dt, *J* = 9.4, 7.2 Hz, 1H), 4.11 (s, 3H), 3.99 (dd, *J* = 7.2, 1.8 Hz, 2H).

^{13}C NMR (126 MHz, $CDCl_3$) δ 220.95, 150.26 (d, *J* = 248.2 Hz), 128.38 (d, *J* = 13.8 Hz), 126.74, 116.17 (d, *J* = 27.9 Hz), 114.49, 59.36, 43.79.

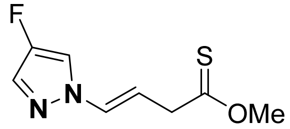

***O*-methyl (*E*)-4-(4-fluoro-1*H*-pyrazol-1-yl)but-3-enethioate (7):**

^{1}H NMR (500 MHz, $CDCl_3$) δ 7.50 (d, *J* = 4.7 Hz, 1H), 7.44 (d, *J* = 4.1 Hz, 1H), 6.81 (dd, *J* = 14.3, 1.3 Hz, 1H), 6.11 (dt, *J* = 14.2, 7.5 Hz, 1H), 4.10 (s, 3H), 3.56 (dd, *J* = 7.5, 1.4 Hz, 2H).

^{13}C NMR (126 MHz, $CDCl_3$) δ 220.40, 150.67 (d, *J* = 249.2 Hz), 130.05, 128.38 (d, *J* = 13.8 Hz), 113.17 (d, *J* = 28.1 Hz), 111.52, 59.44, 46.43.

HRMS (ESI) m/z $[M + H]^+$ calculated for $C_8H_9ClN_2OS$: 217.0197, found 217.0194.

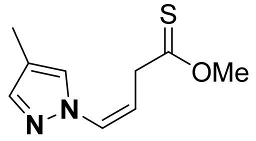

***O*-methyl (*Z*)-4-(4-methyl-1*H*-pyrazol-1-yl)but-3-enethioate (8)**:

^{1}H NMR (500 MHz, $CDCl_3$) δ 7.42 (s, 1H), 7.32 (t, *J* = 0.9 Hz, 1H), 6.76 (dt, *J* = 9.4, 1.8 Hz, 1H), 5.51 (dt, *J* = 9.4, 7.1 Hz, 1H), 4.11 (s, 3H), 4.06 (dd, *J* = 7.2, 1.8 Hz, 2H), 2.12 – 1.91 (m, 3H).

^{13}C NMR (126 MHz, $CDCl_3$) δ 221.73, 141.70, 129.06, 126.61, 112.48, 59.29, 44.11, 31.06, 8.86.

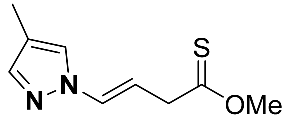

***O*-methyl (*E*)-4-(4-methyl-1*H*-pyrazol-1-yl)but-3-enethioate (8)**:

^{1}H NMR (500 MHz, $CDCl_3$) δ 7.40 (s, 2H), 7.38 – 7.36 (m, 2H), 6.87 (d, *J* = 14.2 Hz, 1H), 6.11 (dt, *J* = 14.2, 7.6 Hz, 2H), 4.09 (s, 6H), 3.56 (dd, *J* = 7.5, 1.4 Hz, 4H), 2.08 (s, 6H).

^{13}C NMR (126 MHz, $CDCl_3$) δ 221.03, 141.69, 129.74, 126.09, 110.16, 59.40, 46.85, 29.84, 8.98.

HRMS (ESI) m/z $[M + H]^+$ calculated for $C_9H_{12}N_2OS$: 197.0743, found 197.0743.

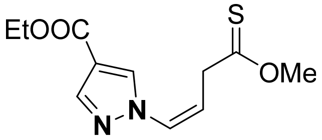

Ethyl (*Z*)-1-(4-methoxy-4-thioxobut-1-en-1-yl)-1*H*-pyrazole-4-carboxylate (9):

^{1}H NMR (500 MHz, $CDCl_3$) δ 7.99 (d, *J* = 14.3 Hz, 2H), 6.80 (dt, *J* = 9.4, 1.9 Hz, 1H), 5.77 (dt, *J* = 9.4, 7.1 Hz, 1H), 4.29 (q, *J* = 7.1 Hz, 2H), 4.10 (s, 3H), 4.03 (dd, *J* = 7.1, 1.9 Hz, 2H), 1.34 (t, *J* = 7.1 Hz, 3H).

^{13}C NMR (126 MHz, $CDCl_3$) δ 220.62, 162.77, 142.01, 133.47, 125.88, 116.80, 115.83, 60.51, 59.33, 43.79, 14.47.

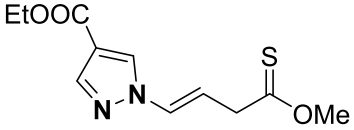

Ethyl (*E*)-1-(4-methoxy-4-thioxobut-1-en-1-yl)-1*H*-pyrazole-4-carboxylate (9):

^{1}H NMR (500 MHz, $CDCl_3$) δ 8.04 (s, 1H), 7.97 (s, 1H), 6.89 (dt, *J* = 14.0, 1.5 Hz, 1H), 6.39 (dt, *J* = 14.1, 7.5 Hz, 1H), 4.30 (q, *J* = 7.1 Hz, 2H), 4.10 (s, 3H), 3.59 (dd, *J* = 7.6, 1.4 Hz, 2H), 1.34 (t, *J* = 7.1 Hz, 3H).

^{13}C NMR (126 MHz, $CDCl_3$) δ 219.88, 162.73, 142.13, 130.77, 128.79, 116.40, 114.58, 60.54, 59.45, 46.45, 14.48.

HRMS (ESI) m/z $[M + H]^+$ calculated for $C_{11}H_{14}N_2O_3S$: 255.0798, found 255.0797.

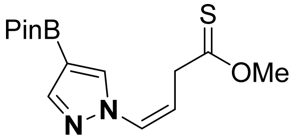

***O*-methyl (*Z*)-4-(4-(4,4,5,5-tetramethyl-1,3,2-dioxaborolan-2-yl)-1*H*-pyrazol-1-yl)but-3-enethioate (10):**

^{1}H NMR (500 MHz, $CDCl_3$) δ 7.86 (s, 1H), 7.79 (s, 1H), 6.83 (d, *J* = 9.3 Hz, 1H), 5.89 – 5.51 (m, 1H), 4.11 (s, 3H), 4.09 (dd, *J* = 6.9, 2.0 Hz, 3H), 1.32 (s, 14H).

^{13}C NMR (126 MHz, $CDCl_3$) δ 146.62, 137.39, 126.09, 114.84, 83.65, 59.30, 44.02, 24.93.

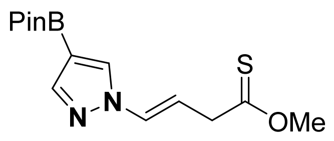

***O*-methyl (*E*)-4-(4-(4,4,5,5-tetramethyl-1,3,2-dioxaborolan-2-yl)-1*H*-pyrazol-1-yl)but-3-enethioate (10):**

^{1}H NMR (500 MHz, $CDCl_3$) δ 7.87 (s, 1H), 7.84 (s, 1H), 6.92 (d, *J* = 14.1 Hz, 1H), 6.30 (dt, *J* = 14.5, 7.6 Hz, 1H), 4.09 (s, 3H), 3.66 – 3.50 (m, 2H), 1.32 (s, 12H).

^{13}C NMR (126 MHz, $CDCl_3$) δ 220.51, 146.68, 134.64, 129.07, 112.72, 83.66, 59.42, 46.77, 24.93.

HRMS (ESI) m/z $[M + H]^+$ calculated for $C_{14}H_{21}BN_2O_3S$: 309.1439, found 309.1439.

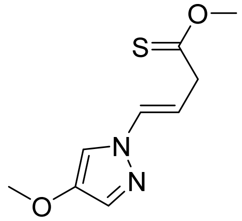

***O*-methyl (*E*)-4-(4-methoxy-1*H*-pyrazol-1-yl)but-3-enethioate (11):**

^{1}H NMR (500 MHz, $CDCl_3$) δ 7.34 (s, 1H), 7.27 (s, 1H), 6.82 (dt, *J* = 14.2, 1.4 Hz, 2H), 6.00 (dt, *J* = 14.3, 7.5 Hz, 2H), 4.10 (s, 5H), 3.76 (s, 5H), 3.55 (dd, *J* = 7.5, 1.4 Hz, 3H).

^{13}C NMR (101 MHz, $CDCl_3$) δ 220.96, 148.12, 130.37, 129.33, 110.79, 109.41, 59.38, 58.88, 46.63.

HRMS (ESI) m/z $[M + H]^+$ calculated for $C_9H_{12}N_2O_2S$: 213.0692, found 213.0692.

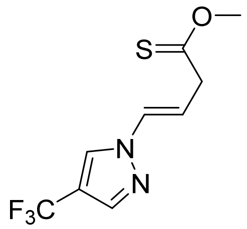

***O*-methyl (*E*)-4-(4-(trifluoromethyl)-1*H*-pyrazol-1-yl)but-3-enethioate (12):**

^{1}H NMR (500 MHz, $CDCl_3$) δ 7.86 (s, 1H), 7.78 (s, 1H), 6.90 (dt, 1H), 6.39 (dt, J = 14.1, 7.6 Hz, 1H), 4.11 (s, 3H), 3.59 (dd, J = 7.6, 1.4 Hz, 2H).

^{13}C NMR (126 MHz, $CDCl_3$) δ 219.62, 137.97 (q, *J* = 2.7 Hz), 128.54, 126.80 (d, *J* = 3.9 Hz), 125.05 – 118.32 (m), 115.03, 114.76, 59.34, 46.18.

HRMS (ESI) m/z $[M + H]^+$ calculated for $C_9H_9F_3N_2OS$: 251.0460, found 251.0460.

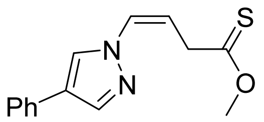

***O*-methyl (*E*)-4-(4-phenyl-1*H*-pyrazol-1-yl)but-3-enethioate (13):**

^{1}H NMR (400 MHz, $CDCl_3$) δ 7.90 (s, 1H), 7.82 (s, 1H), 7.48 (d, J = 6.5 Hz, 2H), 7.38 (d, *J* = 6.9 Hz, 2H), 7.25 (s, 1H), 6.86 (t, J = 7.2 Hz, 1H), 5.64 (t, *J* = 7.3 Hz, 1H), 4.44 – 3.98 (m, 5H).

^{13}C NMR (101 MHz, $CDCl_3$) δ 221.38, 138.66, 131.90, 129.05, 127.04, 126.96, 126.49, 125.82, 123.69, 113.99, 59.38, 44.04.

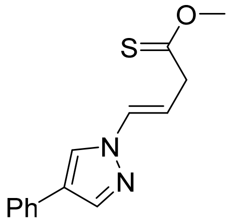

***O*-methyl (*Z*)-4-(4-phenyl-1H-pyrazol-1-yl)but-3-enethioate (13):**

^{1}H NMR (400 MHz, $CDCl_3$) δ 7.87 (s, 1H), 7.82 (s, 1H), 7.49 (s, 2H), 7.37 (d, *J* = 7.5 Hz, 2H), 7.27 – 7.18 (m, 1H), 6.94 (dt, *J* = 14.4, 4.0 Hz, 1H), 6.26 (dt, *J* = 13.6, 6.7 Hz, 1H), 4.10 (dd, *J* = 5.2, 2.6 Hz, 3H), 3.60 (d, *J* = 6.7 Hz, 2H).

^{13}C NMR (101 MHz, $CDCl_3$) δ 220.66, 138.67, 131.90, 129.48, 129.05, 127.00, 125.79, 124.41, 123.99, 111.60, 59.46, 46.71.

HRMS (ESI) m/z $[M + H]^+$ calculated for $C_{14}H_{14}N_2OS$: 259.0900, found 259.0898.

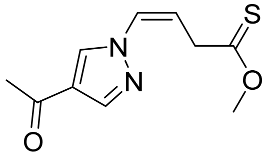

***O*-methyl (*Z*)-4-(4-acetyl-1*H*-pyrazol-1-yl)but-3-enethioate (14):**

^{1}H NMR (500 MHz, $CDCl_3$) δ 8.02 (s, 1H), 7.98 (s, 1H), 6.81 (dt, *J* = 9.3, 1.9 Hz, 1H), 5.78 (dt, *J* = 9.4, 7.2 Hz, 1H), 4.10 (s, 3H), 4.01 (dd, *J* = 7.3, 1.8 Hz, 2H), 2.43 (s, 3H).

^{13}C NMR (126 MHz, $CDCl_3$) δ 220.38, 191.84, 141.25, 132.55, 125.82, 124.37, 117.20, 59.29, 43.73, 28.06.

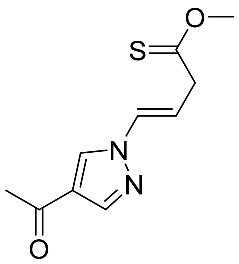

***O*-methyl (*E*)-4-(4-acetyl-1*H*-pyrazol-1-yl)but-3-enethioate (14)**

^{1}H NMR (500 MHz, $CDCl_3$) δ 8.03 (s, 1H), 7.97 (s, 1H), 6.90 (dt, *J* = 14.0, 1.4 Hz, 1H), 6.42 (dt, *J* = 14.0, 7.6 Hz, 1H), 4.10 (s, 3H), 3.59 (dd, *J* = 7.6, 1.4 Hz, 2H), 2.44 (s, 3H).

^{13}C NMR (126 MHz, $CDCl_3$) δ 219.65, 191.78, 141.35, 129.75, 128.56, 124.92, 115.05, 59.35, 46.28, 28.01.

HRMS (ESI) m/z $[M + H]^+$ calculated for $C_{10}H_{12}N_2O_2S$: 225.0692, found 225.0691.

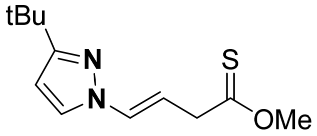

***O*-methyl (*E*)-4-(3-(tert-butyl)-1*H*-pyrazol-1-yl)but-3-enethioate (15):**

^{1}H NMR (500 MHz, $CDCl_3$) δ 7.44 (dd, *J* = 28.4, 2.5 Hz, 1H), 6.99 – 6.63 (m, 1H), 6.18 (dd, *J* = 11.8, 2.5 Hz, 1H), 6.15 – 5.41 (m, 1H), 4.10 (d, *J* = 8.6 Hz, 3H), 4.39 – 3.36 (m, 2H), 1.31 (s, 9H).

^{13}C NMR (126 MHz, $CDCl_3$) δ 221.70 (d, *J* = 134.7 Hz), 163.68, 130.34 (d, *J* = 92.2 Hz), 127.15 (d, *J* = 152.3 Hz), 110.88 (d, *J* = 240.8 Hz), 103.39 (d, *J* = 113.0 Hz), 59.29 (d, *J* = 21.9 Hz), 45.66 (d, *J* = 336.4 Hz), 32.69 – 29.52 (m), 30.51 (d, *J* = 6.8 Hz).

HRMS (ESI) m/z $[M + H]^+$ calculated for $C_{12}H_{18}N_2OS$: 239.1213, found 239.1211.

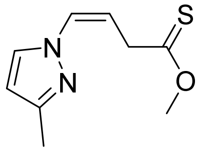

***O*-methyl (*Z*)-4-(3-methyl-1*H*-pyrazol-1-yl)but-3-enethioate (16):**

^{1}H NMR (400 MHz, $CDCl_3$) δ 7.45 (d, *J* = 2.4 Hz, 1H), 6.76 (dt, *J* = 9.4, 1.8 Hz, 1H), 6.10 (d, *J* = 2.3 Hz, 1H), 5.50 (dt, *J* = 9.4, 7.1 Hz, 1H), 4.11 (s, 3H), 4.07 (dd, *J* = 7.2, 1.8 Hz, 2H), 2.30 (s, 4H).

^{13}C NMR (101 MHz, $CDCl_3$) δ 221.61, 150.25, 131.05, 126.46, 112.32, 106.23, 59.12, 44.08, 13.68.

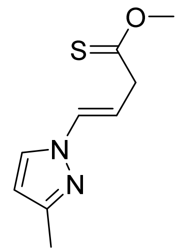

O-methyl (E)-4-(3-methyl-1H-pyrazol-1-yl)but-3-enethioate (16):

^{1}H NMR (400 MHz, $CDCl_3$) δ 7.44 (d, J = 2.4 Hz, 1H), 6.84 (dt, *J* = 14.1, 1.4 Hz, 1H), 6.16 (dt, *J* = 14.1, 7.6 Hz, 1H), 6.11 (d, *J* = 2.3 Hz, 1H), 4.09 (s, 3H), 3.56 (dd, *J* = 7.6, 1.4 Hz, 2H), 2.30 (s, 3H).

^{13}C NMR (101 MHz, $CDCl_3$) δ 220.85, 150.45, 129.25, 128.50, 110.07, 106.89, 59.24, 46.83, 13.63.

HRMS (ESI) m/z $[M + H]^+$ calculated for $C_9H_{12}N_2OS$: 197.0743, found 197.0743.

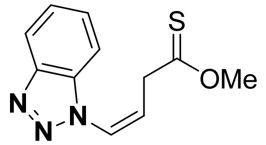

O-methyl (Z)-4-(1H-benzo[d][1,2,3]triazol-1-yl)but-3-enethioate (17):

^{1}H NMR (500 MHz, $CDCl_3$) δ 8.09 (d, *J* = 8.3 Hz, 1H), 7.64 – 7.49 (m, 2H), 7.42 (ddd, *J* = 8.2, 4.9, 3.1 Hz, 1H), 7.16 (dt, *J* = 9.1, 2.0 Hz, 1H), 6.21 (dt, *J* = 9.1, 7.0 Hz, 1H), 4.11 (s, 3H), 4.08 (dd, *J* = 6.9, 1.9 Hz, 2H).

^{13}C NMR (126 MHz, $CDCl_3$) δ 220.48, 145.30, 132.85, 128.27, 124.66, 120.99, 120.59, 120.30, 109.58, 59.34, 44.28.

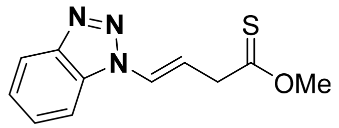

O-methyl (E)-4-(1H-benzo[d][1,2,3]triazol-1-yl)but-3-enethioate (17):

^{1}H NMR (500 MHz, $CDCl_3$) δ 8.09 (d, *J* = 8.3 Hz, 1H), 7.74 (d, *J* = 8.3 Hz, 1H), 7.56 (ddd, *J* = 8.3, 6.8, 0.9 Hz, 1H), 7.50 – 7.36 (m, 2H), 6.71 (dt, *J* = 14.7, 7.5 Hz, 1H), 4.15 (s, 2H), 3.73 (dd, *J* = 7.6, 1.4 Hz, 2H).

^{13}C NMR (126 MHz, $CDCl_3$) δ 219.87, 146.41, 131.61, 128.45, 125.53, 124.70, 120.50, 116.55, 110.39, 59.54, 46.92.

HRMS (ESI) m/z $[M + H]^+$ calculated for $C_{11}H_{11}N_3OS$: 234.0496, found 234.0493.

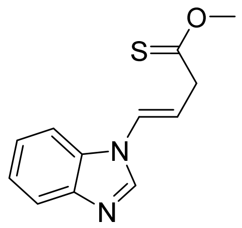

O-methyl (E)-4-(1H-benzo[d]imidazol-1-yl)but-3-enethioate (18):

1H NMR (500 MHz, $CDCl_3$) δ 8.69 (s, 1H), 7.95 – 7.82 (m, 1H), 7.74 – 7.56 (m, 1H), 7.49 – 7.34 (m, 2H), 7.05 (dt, *J* = 14.3, 1.4 Hz, 1H), 6.44 (dt, *J* = 14.6, 7.5 Hz, 1H), 4.15 (s, 3H), 3.69 (dd, *J* = 7.5, 1.3 Hz, 2H).

13C NMR (126 MHz, $CDCl_3$) δ 218.46, 140.13, 133.67, 124.99, 124.45, 123.68, 119.35, 118.62, 117.50, 111.09, 59.43, 46.42.

HRMS (ESI) m/z $[M + H]^+$ calculated for $C_{12}H_{12}N_2OS$: 233.0743, found 233.0743

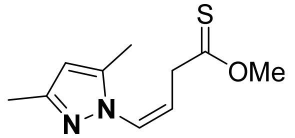

O-methyl (Z)-4-(3,5-dimethyl-1H-pyrazol-1-yl)but-3-enethioate (19):

^{1}H NMR (500 MHz, $CDCl_3$) δ 6.61 (d, *J* = 9.3 Hz, 1H), 5.83 (s, 1H), 5.58 (dt, *J* = 9.3, 6.8 Hz, 1H), 4.16 (dd, *J* = 6.8, 1.8 Hz, 3H), 4.10 (s, 4H), 2.23 (d, *J* = 4.1 Hz, 8H).

^{13}C NMR (126 MHz, $CDCl_3$) δ 222.77, 149.35, 140.01, 123.11, 114.09, 105.90, 59.16, 44.11, 13.86, 11.31.

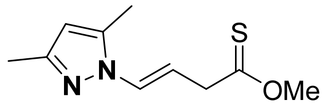

O-methyl (E)-4-(3,5-dimethyl-1H-pyrazol-1-yl)but-3-enethioate (19):

^{1}H NMR (500 MHz, $CDCl_3$) δ 6.74 (dd, *J* = 13.7, 1.6 Hz, 1H), 6.29 (dt, *J* = 14.4, 7.6 Hz, 1H), 5.84 (s, 1H), 4.08 (s, 3H), 3.58 (d, *J* = 7.7 Hz, 2H), 2.26 (s, 3H), 2.24 (s, 3H).

^{13}C NMR (126 MHz, $CDCl_3$) δ 221.25, 149.98, 139.11, 125.97, 110.92, 106.77, 59.41, 47.59, 13.77, 11.11.

HRMS (ESI) m/z $[M + H]^+$ calculated for $C_{10}H_{14}N_2OS$: 211.0900, found 211.0897.

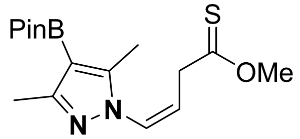

***O*-methyl (*Z*)-4-(3,5-dimethyl-4-(4,4,5,5-tetramethyl-1,3,2-dioxaborolan-2-yl)-1*H*-pyrazol-1-yl)but-3-enethioate (20):**

^{1}H NMR (500 MHz, $CDCl_3$) δ 6.63 (d, *J* = 9.3 Hz, 1H), 5.64 (dt, *J* = 9.3, 6.8 Hz, 1H), 4.11 (dd, *J* = 7.1, 2.1 Hz, 2H), 4.09 (s, 3H), 2.40 (s, 3H), 2.34 (s, 3H), 1.29 (s, 12H).

^{13}C NMR (126 MHz, $CDCl_3$) δ 222.47, 155.95, 148.25, 123.20, 115.73, 82.77, 59.13, 44.12, 25.02, 14.33, 11.50.

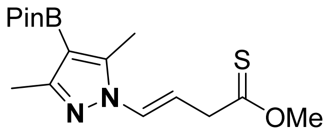

***O*-methyl (E)-4-(3,5-dimethyl-4-(4,4,5,5-tetramethyl-1,3,2-dioxaborolan-2-yl)-1*H*-pyrazol-1-yl)but-3-enethioate (20):**

^{1}H NMR (500 MHz, $CDCl_3$) δ 6.83 – 6.66 (m, 1H), 6.34 (dt, *J* = 13.7, 7.7 Hz, 1H), 4.07 (s, 3H), 3.58 (dd, *J* = 7.6, 1.4 Hz, 2H), 2.43 (s, 3H), 2.35 (s, 3H), 1.28 (s, 12H).

^{13}C NMR (126 MHz, $CDCl_3$) δ 220.98, 156.56, 147.08, 125.53, 111.97, 82.65, 59.29, 47.54, 24.89, 14.14, 11.10.

HRMS (ESI) m/z $[M + H]^+$ calculated for $C_{16}H_{25}BN_2O_3S$: 337.1752, found 337.1750.

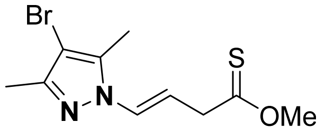

***O*-methyl (*E*)-4-(4-bromo-3,5-dimethyl-1*H*-pyrazol-1-yl)but-3-enethioate (21):**

^{1}H NMR (500 MHz, $CDCl_3$) δ 6.74 (dt, *J* = 13.7, 1.3 Hz, 1H), 6.33 (dt, *J* = 13.7, 7.7 Hz, 1H), 4.09 (s, 3H), 3.58 (dd, *J* = 7.6, 1.4 Hz, 2H), 2.28 (s, 3H), 2.24 (s, 3H).

^{13}C NMR (126 MHz, $CDCl_3$) δ 220.77, 148.39, 137.05, 126.20, 112.32, 96.24, 59.45, 47.24, 12.58, 10.46.

HRMS (ESI) m/z $[M + H]^+$ calculated for $C_{10}H_{13}BrN_2OS$: 289.0005, found 289.0003.

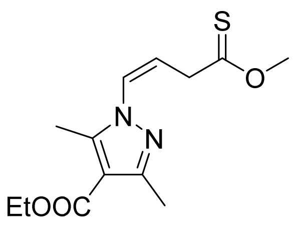

Ethyl (*Z*)-1-(4-methoxy-4-thioxobut-1-en-1-yl)-3,5-dimethyl-1*H*-pyrazole-4-carboxylate (22):

^{1}H NMR (400 MHz, $CDCl_3$) δ 6.66 (dt, *J* = 9.1, 1.8 Hz, 1H), 5.84 (dt, *J* = 9.1, 6.9 Hz, 1H), 4.29 (q, *J* = 7.1 Hz, 2H), 4.10 (s, 3H), 4.01 (dd, *J* = 6.9, 1.8 Hz, 2H), 2.51 (s, 3H), 2.43 (s, 3H), 1.36 (t, *J* = 7.1 Hz, 3H).

^{13}C NMR (101 MHz, $CDCl_3$) δ 221.47, 164.56, 151.62, 144.75, 123.02, 119.13, 110.25, 59.91, 59.20, 44.01, 14.63, 14.53, 11.38.

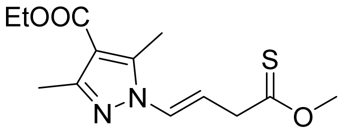

Ethyl (*E*)-1-(4-methoxy-4-thioxobut-1-en-1-yl)-3,5-dimethyl-1*H*-pyrazole-4-carboxylate (22):

^{1}H NMR (400 MHz, $CDCl_3$) δ 6.79 (dt, *J* = 13.7, 1.4 Hz, 1H), 6.47 (dt, *J* = 13.7, 7.7 Hz, 1H), 4.29 (q, *J* = 7.1 Hz, 2H), 4.10 (s, 3H), 3.61 (dd, *J* = 7.6, 1.4 Hz, 2H), 2.56 (s, 3H), 2.44 (s, 3H), 1.36 (t, *J* = 7.1 Hz, 3H).

^{13}C NMR (101 MHz, $CDCl_3$) δ 220.28, 164.29, 152.11, 143.42, 125.02, 114.77, 110.85, 59.77, 59.31, 47.16, 14.47, 14.35, 10.85.

HRMS (ESI) m/z $[M + H]^+$ calculated for $C_{13}H_{18}BrN_2O_3S$: 283.1111, found 283.1109.

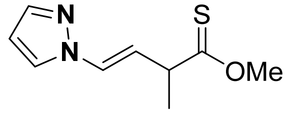

***O*-methyl (*E*)-2-methyl-4-(1*H*-pyrazol-1-yl)but-3-enethioate (23):**

^{1}H NMR (400 MHz, $CDCl_3$) δ 7.59 (d, *J* = 1.8 Hz, 1H), 7.56 (dd, *J* = 2.5, 0.7 Hz, 1H), 6.93 (dd, *J* = 14.2, 1.0 Hz, 1H), 6.33 (dd, *J* = 2.5, 1.8 Hz, 1H), 6.20 (dd, *J* = 14.2, 8.6 Hz, 1H), 4.09 (s, 3H), 3.76 – 3.53 (m, 1H), 1.45 (d, *J* = 7.0 Hz, 3H).

^{13}C NMR (101 MHz, $CDCl_3$) δ 225.56, 140.92, 127.90, 127.70, 118.23, 107.10, 59.33, 50.88, 20.74.

HRMS (ESI) m/z $[M + H]^+$ calculated for $C_9H_{12}N_2OS$: 197.0743, found 197.0741.

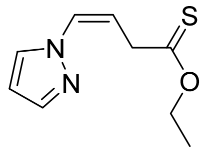

***O*-ethyl (*Z*)-4-(1*H*-pyrazol-1-yl)but-3-enethioate (24):**

^{1}H NMR (400 MHz, $CDCl_3$) δ 7.63 (d, *J* = 1.8 Hz, 1H), 7.58 (dd, *J* = 2.4, 0.7 Hz, 1H), 6.85 (dt, *J* = 9.4, 1.9 Hz, 1H), 6.33 (dd, *J* = 2.5, 1.8 Hz, 1H), 5.63 (dt, *J* = 9.4, 7.1 Hz, 1H), 4.54 (t, *J* = 7.1 Hz, 2H), 4.04 (dd, *J* = 7.1, 1.8 Hz, 2H), 1.41 (t, *J* = 7.1 Hz, 3H).

^{13}C NMR (101 MHz, $CDCl_3$) δ 220.58, 140.73, 130.31, 126.42, 113.81, 106.28, 68.53, 44.32, 13.57.

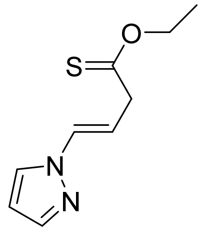

_O_-ethyl (_E_)-4-(1_H_-pyrazol-1-yl)but-3-enethioate (24):

^{1}H NMR (500 MHz, $CDCl_3$) δ 7.58 (d, J = 1.8 Hz, 1H), 7.56 (d, J = 2.5 Hz, 1H), 6.92 (dt, J = 14.1, 1.5 Hz, 1H), 6.32 (t, J = 2.1 Hz, 1H), 6.23 (dt, J = 14.1, 7.6 Hz, 1H), 4.51 (q, J = 7.1 Hz, 2H), 3.54 (dd, J = 7.6, 1.4 Hz, 2H), 1.39 (t, J = 7.1 Hz, 3H).

^{13}C NMR (126 MHz, $CDCl_3$) δ 219.97, 140.81, 129.34, 127.54, 111.53, 106.98, 68.74, 47.07, 13.60.

HRMS (ESI) m/z $[M + H]^+$ calculated for $C_9H_{12}N_2OS$: 197.0743, found 197.0743.

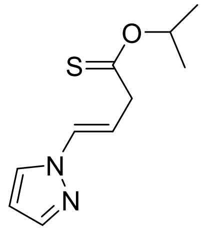

_O_-isopropyl (_E_)-4-(1_H_-pyrazol-1-yl)but-3-enethioate (25):

^{1}H NMR (500 MHz, $CDCl_3$) δ 7.59 (d, J = 1.7 Hz, 1H), 7.58 (d, J = 2.5 Hz, 1H), 6.94 (dt, J = 14.1, 1.5 Hz, 1H), 6.34 (t, J = 2.1 Hz, 1H), 6.23 (dt, J = 14.0, 7.5 Hz, 1H), 5.63 (p, J = 6.2 Hz, 1H), 3.51 (dd, J = 7.5, 1.4 Hz, 2H), 1.36 (d, J = 6.3 Hz, 7H).

^{13}C NMR (126 MHz, $CDCl_3$) δ 219.31, 140.89, 129.39, 127.62, 111.75, 107.09, 76.04, 47.68, 21.15.

HRMS (ESI) m/z $[M + H]^+$ calculated for $C_{10}H_{14}N_2OS$: 211.0900, found 211.0899.

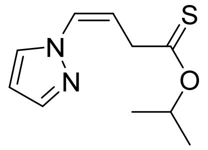

_O_-isopropyl (_Z_)-4-(1_H_-pyrazol-1-yl)but-3-enethioate (25):

1H NMR (500 MHz, CDCl3) δ 7.62 (d, J = 1.8 Hz, 2H), 7.59 (d, J = 2.5 Hz, 2H), 6.85 (dt, J = 9.4, 1.9 Hz, 2H), 6.33 (t, J = 2.1 Hz, 2H), 5.67 (p, J = 6.2 Hz, 2H), 5.62 (dt, J = 9.4, 7.1 Hz, 2H), 3.97 (dd, J = 7.1, 1.9 Hz, 4H), 1.36 (d, J = 6.2 Hz, 12H).

13C NMR (126 MHz, CDCl3) δ 219.87, 140.85, 130.44, 126.56, 113.98, 106.43, 75.84, 45.00, 21.16.

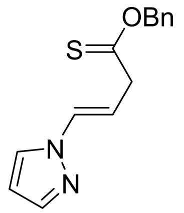

***O*-benzyl (E)-4-(1H-pyrazol-1-yl)but-3-enethioate (26):**

^{1}H NMR (400 MHz, $CDCl_3$) δ 7.60 (d, *J* = 1.8 Hz, 1H), 7.57 (dd, *J* = 2.4, 0.7 Hz, 1H), 7.47 – 7.33 (m, 5H), 6.95 (dt, *J* = 14.2, 1.4 Hz, 1H), 6.34 (dd, *J* = 2.5, 1.8 Hz, 1H), 6.27 (dt, J = 14.1, 7.6 Hz, 1H), 5.51 (s, 2H), 3.61 (dd, J = 7.6, 1.4 Hz, 2H).

^{13}C NMR (101 MHz, $CDCl_3$) δ 219.56, 140.82, 134.82, 129.50, 128.66, 128.62, 128.50, 127.49, 111.42, 106.98, 74.20, 46.88.

HRMS (ESI) m/z $[M + H]^+$ calculated for $C_{14}H_{14}N_2OS$: 259.0900, found 259.0898.

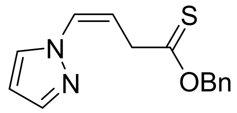

***O*-benzyl (Z)-4-(1H-pyrazol-1-yl)but-3-enethioate (26):**

^{1}H NMR (400 MHz, $CDCl_3$) δ 7.61 (d, J = 1.8 Hz, 1H), 7.55 (d, J = 2.4 Hz, 1H), 7.43 – 7.33 (m, 5H), 6.85 (dt, J = 9.4, 1.8 Hz, 1H), 6.32 (t, J = 2.2 Hz, 1H), 5.64 (dt, J = 9.4, 7.1 Hz, 1H), 5.52 (s, 2H), 4.11 (dd, J = 7.1, 1.9 Hz, 2H).

^{13}C NMR (101 MHz, $CDCl_3$) δ 220.25, 140.77, 134.93, 130.33, 128.62, 128.54, 128.42, 126.47, 113.54, 106.29, 74.05, 44.26.

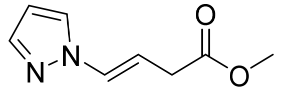

Methyl (E)-4-(1H-pyrazol-1-yl)but-3-enoate (27): The title compound was prepared according to the general procedure D. The crude residue was purified by column chromatography on silica gel with an eluent of hexanes/ethyl acetate (2:1) to furnish the pure compound as a yellow oil in 76% yield.

^{1}H NMR (500 MHz, $CDCl_3$) δ 7.59 (d, *J* = 1.8 Hz, 1H), 7.58 (d, *J* = 2.4 Hz, 1H), 6.97 (d, *J* = 14.2 Hz, 1H), 6.34 (t, *J* = 2.1 Hz, 1H), 6.15 (dt, *J* = 14.2, 7.5 Hz, 1H), 3.72 (s, 3H), 3.21 (dd, *J* = 7.5, 1.5 Hz, 2H).

^{13}C NMR (126 MHz, $CDCl_3$) δ 171.68, 140.97, 130.08, 127.60, 109.11, 107.18, 52.23, 35.00.

HRMS (ESI) m/z $[M + H]^+$ calculated for $C_8H_{10}N_2O_2$: 167.0815, found 167.0814.

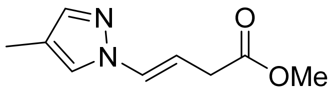

Methyl (E)-4-(4-methyl-1H-pyrazol-1-yl)but-3-enoate (28):

^{1}H NMR (500 MHz, $CDCl_3$) δ 7.36 (d, *J* = 13.5 Hz, 3H), 6.93 – 6.76 (m, 1H), 6.00 (dt, *J* = 14.6, 7.5 Hz, 1H), 3.70 (s, 4H), 3.17 (dd, *J* = 7.5, 1.5 Hz, 3H), 2.07 (s, 4H).

^{13}C NMR (126 MHz, $CDCl_3$) δ 171.74, 141.63, 130.22, 125.96, 117.82, 107.69, 52.12, 34.98, 8.92.

HRMS (ESI) m/z $[M + H]^+$ calculated for $C_9H_{12}N_2O_2$: 181.0972, found 181.0971.

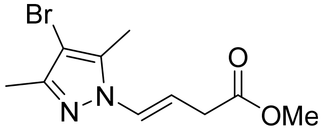

methyl (E)-4-(4-bromo-3,5-dimethyl-1H-pyrazol-1-yl)but-3-enoate (29):

^{1}H NMR (500 MHz, $CDCl_3$) δ 6.77 (d, *J* = 13.8 Hz, 1H), 6.25 (dt, *J* = 13.7, 7.6 Hz, 2H), 3.71 (s, 5H), 3.19 (dd, *J* = 7.6, 1.4 Hz, 3H), 2.27 (s, 5H), 2.24 (s, 5H).

^{13}C NMR (126 MHz, $CDCl_3$) δ 171.62, 148.40, 136.98, 126.67, 110.15, 96.25, 52.19, 35.27, 12.55, 10.41.

HRMS (ESI) m/z $[M + H]^+$ calculated for $C_{10}H_{13}BrN_2O_2$: 273.0233, found 273.0231.

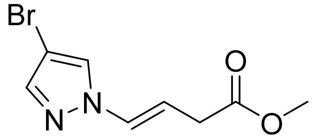

4-bromo-1-(5-methoxy-2,3-dihydrofuran-2-yl)-1H-pyrazole (30):

^{1}H NMR (500 MHz, $CDCl_3$) δ 7.61 (s, 1H), 7.53 (s, 1H), 6.89 (dt, *J* = 14.3, 1.5 Hz, 1H), 6.11 (dt, *J* = 14.2, 7.5 Hz, 1H), 3.72 (s, 3H), 3.20 (dd, *J* = 7.5, 1.5 Hz, 2H).

^{13}C NMR (126 MHz, $CDCl_3$) δ 171.23, 141.24, 129.57, 127.35, 110.00, 95.19, 52.15, 34.62.

HRMS (ESI) m/z $[M + H]^+$ calculated for $C_8H_9BrN_2O_2$: 244.9920, found 244.9920.

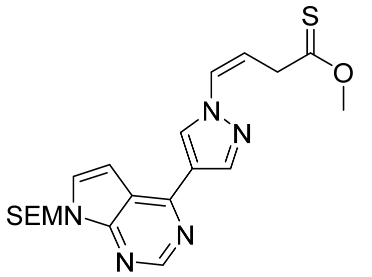

***O*-methyl (Z)-4-(4-(7-((2-(trimethylsilyl)ethoxy)methyl)-7*H*-pyrrolo[2,3-d]pyrimidin-4-yl)-1*H*-pyrazol-1-yl)but-3-enethioate (31):**

^{1}H NMR (400 MHz, $CDCl_3$) δ 8.85 (s, 1H), 8.38 (s, 1H), 8.37 (s, 1H), 7.40 (d, *J* = 3.7 Hz, 1H), 6.93 (dt, *J* = 9.4, 1.8 Hz, 1H), 6.80 (d, *J* = 3.8 Hz, 1H), 5.77 (dt, *J* = 9.3, 7.3 Hz, 1H), 5.67 (s, 2H), 4.14 (s, 3H), 4.16 – 4.10 (m, 2H), 3.61 – 3.48 (m, 2H), 1.05 – 0.69 (m, 2H), -0.06 (s, 9H).

^{13}C NMR (101 MHz, $CDCl_3$) δ 220.82, 152.23, 151.90, 150.53, 140.59, 130.73, 128.47, 126.24, 122.15, 115.71, 114.34, 100.74, 72.80, 66.59, 59.24, 43.95, 17.74, -1.46.

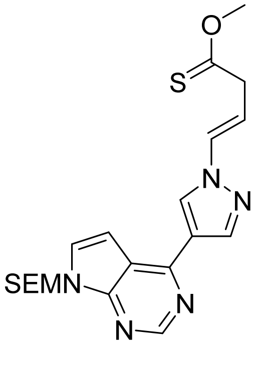

***O*-methyl (*E*)-4-(4-(7-((2-(trimethylsilyl)ethoxy)methyl)-7*H*-pyrrolo[2,3-d]pyrimidin-4-yl)-1*H*-pyrazol-1-yl)but-3-enethioate (31):**

^{1}H NMR (400 MHz, $CDCl_3$) δ 8.85 (s, 1H), 8.39 (s, 1H), 8.33 (s, 1H), 7.40 (d, J = 3.7 Hz, 1H), 7.01 (dt, *J* = 14.2, 1.5 Hz, 1H), 6.79 (d, *J* = 3.7 Hz, 1H), 6.43 (dt, *J* = 14.1, 7.6 Hz, 1H), 5.67 (s, 2H), 4.12 (s, 3H), 3.63 (dd, *J* = 7.6, 1.4 Hz, 2H), 3.60 – 3.42 (m, 2H), 1.06 – 0.77 (m, 2H), -0.06 (s, 9H).

^{13}C NMR (101 MHz, $CDCl_3$) δ 220.02, 152.25, 151.88, 150.47, 140.58, 129.04, 128.49, 127.84, 122.81, 114.31, 113.47, 100.74, 72.80, 66.59, 59.31, 46.51, 17.74, -1.46.

HRMS (ESI) m/z $[M + H]^{+}$ calculated for $C_{20}H_{27}N_5O_2SSi$: 430.1728, found 430.1727.

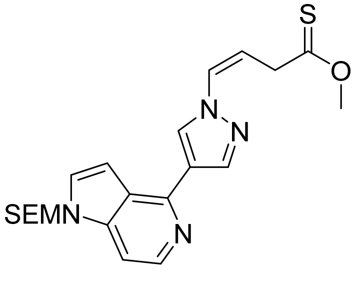

***O*-methyl (*Z*)-4-(4-(1-((2-(trimethylsilyl)ethoxy)methyl)-1*H*-pyrrolo[3,2-c]pyridin-4-yl)-1*H*-pyrazol-1-yl)but-3-enethioate (32):**

^{1}H NMR (500 MHz, $CDCl_3$) δ 8.33 (d, *J* = 5.0 Hz, 1H), 8.12 (s, 1H), 8.10 (s, 1H), 7.41 (d, *J* = 3.6 Hz, 1H), 7.19 (d, *J* = 5.0 Hz, 1H), 6.93 (dt, *J* = 9.4, 1.8 Hz, 1H), 6.72 (d, *J* = 3.7 Hz, 1H), 5.72 (d, *J* = 13.0 Hz, 3H), 4.14 (s, 2H), 4.13 (dd, *J* = 7.3, 1.8 Hz, 2H), 3.77 – 3.36 (m, 2H), 1.04 – 0.69 (m, 2H), -0.06 (s, 9H).

^{13}C NMR (126 MHz, $CDCl_3$) δ 222.40, 150.33, 144.96, 141.24, 133.80, 130.33, 129.51, 127.83, 122.34, 118.91, 116.34, 115.22, 101.74, 74.46, 67.68, 60.72, 45.43, 19.22, -0.00.

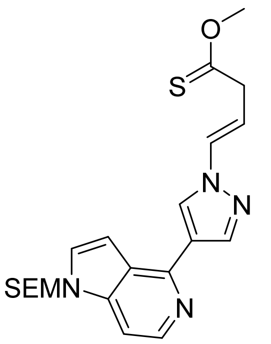

***O*-methyl (*E*)-4-(4-(1-((2-(trimethylsilyl)ethoxy)methyl)-1*H*-pyrrolo[3,2-c]pyridin-4-yl)-1*H*-pyrazol-1-yl)but-3-enethioate (32):**

1H NMR (500 MHz, CDCl3) δ 8.32 (d, J = 5.0 Hz, 1H), 8.09 (s, 1H), 8.06 (s, 1H), 7.41 (d, J = 3.6 Hz, 1H), 7.18 (s, 1H), 7.01 (d, J = 14.1 Hz, 1H), 6.71 (d, J = 3.6 Hz, 1H), 6.37 (dt, J = 14.6, 7.5 Hz, 1H), 5.70 (s, 2H), 4.12 (s, 3H), 3.63 (d, J = 7.5 Hz, 2H), 3.56 (t, J = 8.2 Hz, 2H), 0.92 (t, J = 8.2 Hz, 2H), -0.07 (s, 9H).

13C NMR (126 MHz, CDCl3) δ 221.71, 150.33, 144.96, 141.30, 133.79, 130.63, 129.51, 127.37, 123.04, 118.90, 115.23, 113.99, 101.73, 74.46, 67.67, 60.79, 48.00, 19.22, 0.00.

HRMS (ESI) m/z $[M + H]^+$ calculated for $C_{21}H_{28}N_4O_2SSi$: 429.1775, found 429.1775.

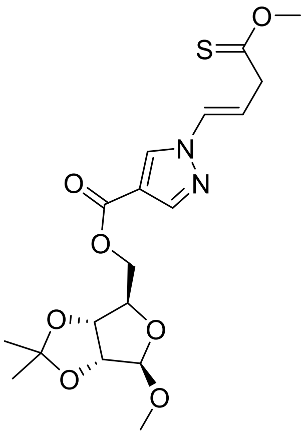

((3a*R*,4*R*,6*R*,6a*R*)-6-methoxy-2,2-dimethyltetrahydrofuro[3,4-d][1,3]dioxol-4-yl)methyl 1-((*E*)-4-methoxy-4-thioxobut-1-en-1-yl)-1*H*-pyrazole-4-carboxylate (33):

^1H NMR (400 MHz, $CDCl_3$) δ 8.08 (d, J = 0.6 Hz, 1H), 8.00 (s, 1H), 6.90 (dt, J = 14.1, 1.5 Hz, 1H), 6.41 (dt, J = 14.0, 7.6 Hz, 1H), 5.00 (s, 1H), 4.72 (dd, J = 6.0, 1.0 Hz, 1H), 4.63 (d, J = 5.9 Hz, 1H), 4.47 (td, J = 6.8, 1.1 Hz, 1H), 4.28 (dd, J = 6.8, 0.7 Hz, 2H), 4.10 (s, 3H), 3.59 (dd, J = 7.6, 1.4 Hz, 2H), 3.32 (s, 3H), 1.49 (d, J = 0.7 Hz, 4H), 1.33 (d, J = 0.7 Hz, 4H).

^{13}C NMR (101 MHz, $CDCl_3$) δ 219.63, 162.03, 142.14, 130.86, 128.60, 115.52, 114.81, 112.63, 109.51, 85.30, 84.36, 81.91, 64.62, 59.31, 54.94, 46.30, 26.47, 25.03.

HRMS (ESI) m/z $[M + H]^+$ calculated for $C_{18}H_{24}N_2O_7S$: 413.1377, found 413.1377.

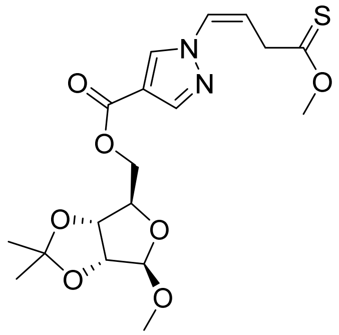

((3a*R*,4*R*,6*R*,6a*R*)-6-methoxy-2,2-dimethyltetrahydrofuro[3,4-d][1,3]dioxol-4-yl)methyl 1-((*Z*)-4-methoxy-4-thioxobut-1-en-1-yl)-1*H*-pyrazole-4-carboxylate (33):

^{1}H NMR (400 MHz, $CDCl_3$) δ 8.06 (s, 1H), 8.03 (s, 1H), 6.81 (dt, *J* = 9.2, 1.9 Hz, 1H), 5.79 (dt, *J* = 9.4, 7.2 Hz, 1H), 5.01 (s, 1H), 4.77 – 4.68 (m, 1H), 4.63 (d, *J* = 5.9 Hz, 1H), 4.47 (d, *J* = 1.1 Hz, 1H), 4.29 (dd, *J* = 6.7, 1.9 Hz, 2H), 4.12 (s, 3H), 4.04 (dd, *J* = 7.2, 1.8 Hz, 2H), 3.33 (s, 3H), 1.50 (d, *J* = 0.7 Hz, 3H), 1.39 – 1.19 (m, 3H).

^{13}C NMR (101 MHz, $CDCl_3$) δ 220.40, 162.08, 142.05, 133.58, 125.73, 117.06, 114.99, 112.62, 109.52, 85.32, 84.37, 81.91, 64.60, 59.21, 54.93, 43.69, 26.47, 25.02.

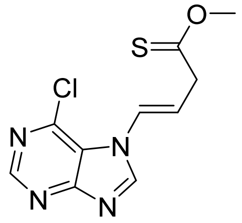

***O*-methyl (*E*)-4-(6-chloro-7*H*-purin-7-yl)but-3-enethioate (34):**

^{1}H NMR (400 MHz, $CDCl_3$) δ 8.80 (s, 1H), 8.32 (s, 1H), 7.12 (dt, *J* = 14.4, 1.4 Hz, 1H), 6.74 (dt, *J* = 14.4, 7.5 Hz, 1H), 4.14 (s, 3H), 3.68 (dd, *J* = 7.5, 1.4 Hz, 2H).

^{13}C NMR (101 MHz, $CDCl_3$) δ 219.17, 152.56, 151.53, 150.75, 142.55, 132.03, 122.02, 118.09, 59.38, 46.57.

HRMS (ESI) m/z $[M + H]^+$ calculated for $C_{10}H_9ClN_4OS$: 269.0258, found 269.0258.

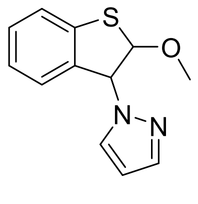

1-(2-methoxy-2,3-dihydrobenzo[b]thiophen-3-yl)-1H-pyrazole (35):

^{1}H NMR (500 MHz, $CDCl_3$) δ 7.62 (d, *J* = 1.8 Hz, 1H), 7.56 (d, *J* = 2.5 Hz, 1H), 7.35 – 7.27 (m, 2H), 7.12 (td, *J* = 7.4, 1.5 Hz, 1H), 6.93 (d, *J* = 7.6 Hz, 1H), 6.35 (d, *J* = 2.3 Hz, 1H), 6.25 (d, *J* = 5.0 Hz, 1H), 5.33 (d, *J* = 4.8 Hz, 1H), 3.27 (s, 3H).

^{13}C NMR (126 MHz, $CDCl_3$) δ 139.51, 137.24, 136.47, 130.92, 129.46, 125.36, 124.99, 123.90, 106.24, 91.96, 72.19, 57.15.

HRMS (ESI) m/z $[M + H]^+$ calculated for $C_{12}H_{12}N_2OS$: 233.0743, found 233.0743.

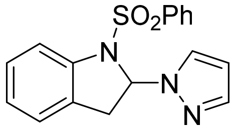

1-(phenylsulfonyl)-2-(1*H*-pyrazol-1-yl)indoline (36):

^{1}H NMR (500 MHz, $CDCl_3$) δ 7.56 (d, *J* = 2.5 Hz, 1H), 7.51 – 7.44 (m, 2H), 7.43 – 7.33 (m, 3H), 7.32 – 7.22 (m, 2H), 7.15 – 7.11 (m, 1H), 7.08 (dd, *J* = 7.5, 1.3 Hz, 1H), 6.97 (td, *J* = 7.5, 1.0 Hz, 1H), 6.46 (dd, *J* = 8.8, 2.2 Hz, 1H), 6.11 (t, *J* = 2.1 Hz, 1H), 3.42 (dd, *J* = 16.9, 2.2 Hz, 1H), 3.33 (dd, *J* = 16.9, 8.8 Hz, 1H).

^{13}C NMR (126 MHz, $CDCl_3$) δ 140.70, 140.48, 138.69, 133.40, 129.39, 129.24, 129.19, 128.21, 126.85, 125.10, 124.72, 114.95, 106.28, 75.61, 36.00.

HRMS (ESI) m/z $[M + H]^+$ calculated for $C_{17}H_{15}N_3O_2S$: 181.0972, found 181.0971.

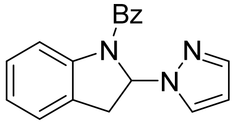

(2-(1*H*-pyrazol-1-yl)indolin-1-yl)(phenyl)methanone (37):

^{1}H NMR (500 MHz, $CDCl_3$) δ 7.53 – 7.07 (m, 8H), 7.07 – 6.82 (m, 2H), 6.67 – 6.41 (m, 1H), 6.05 (d, *J* = 2.3 Hz, 1H), 3.58 (dd, *J* = 16.7, 8.4 Hz, 1H), 3.26 (d, *J* = 16.0 Hz, 1H).

^{13}C NMR (126 MHz, $CDCl_3$) δ 169.62, 140.27, 135.57, 130.88, 129.52, 128.70, 128.38, 127.69, 127.10, 125.16, 124.75, 116.63, 105.91, 75.02, 36.49.

HRMS (ESI) m/z $[M + H]^+$ calculated for $C_{18}H_{15}N_3O$: 290.1288, found 290.1285.

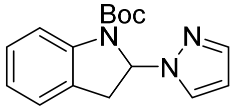

***tert*-butyl 2-(1*H*-pyrazol-1-yl)indoline-1-carboxylate (38):**

^{1}H NMR (500 MHz, DMSO-d_6) δ 7.76 (d, *J* = 2.4 Hz, 1H), 7.42 (d, *J* = 1.7 Hz, 1H), 7.26 (d, *J* = 7.4 Hz, 1H), 7.20 (td, *J* = 7.7, 1.3 Hz, 1H), 7.01 (td, *J* = 7.5, 1.1 Hz, 1H), 6.62 (dd, *J* = 9.5, 2.1 Hz, 1H), 6.23 (t, *J* = 2.1 Hz, 1H), 3.69 (dd, *J* = 17.1, 9.4 Hz, 1H), 3.21 (d, *J* = 17.1 Hz, 1H), 1.39 (s, 10H).

^{13}C NMR (126 MHz, DMSO-d_6) δ 151.12, 142.37, 139.76, 129.91, 129.07, 127.20, 124.50, 122.79, 114.05, 104.67, 81.04, 72.89, 33.58, 27.80.

HRMS (ESI) m/z $[M + H]^+$ calculated for $C_{16}H_{19}N_3O_2$: 286.1550, found 286.1550.

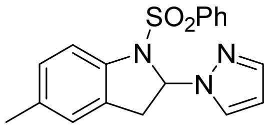

5-methyl-1-(phenylsulfonyl)-2-(1*H*-pyrazol-1-yl)indoline (39):

^1H NMR (500 MHz, $CDCl_3$) δ 7.67 (d, J = 2.7 Hz, 1H), 7.57 (d, J = 7.8 Hz, 2H), 7.54 – 7.43 (m, 2H), 7.37 (q, J = 7.6 Hz, 3H), 7.08 – 6.86 (m, 2H), 6.51 (d, J = 8.9 Hz, 1H), 6.21 (d, J = 2.4 Hz, 1H), 3.47 (d, J = 16.8 Hz, 1H), 3.34 (dd, J = 16.8, 8.8 Hz, 1H), 2.28 (s, 3H).

^{13}C NMR (126 MHz, $CDCl_3$) δ 140.64, 138.55, 138.06, 134.62, 133.35, 129.65, 129.16, 129.14, 128.76, 126.85, 125.73, 115.01, 106.24, 75.81, 35.87, 21.08.

HRMS (ESI) m/z $[M + H]^+$ calculated for $C_{18}H_{17}N_3O_2S$: 340.1114, found 340.1113.

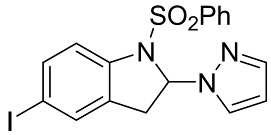

5-iodo-1-(phenylsulfonyl)-2-(1*H*-pyrazol-1-yl)indoline (40):

^1H NMR (500 MHz, $CDCl_3$) δ 7.67 (s, 1H), 7.55 – 7.48 (m, 5H), 7.45 (s, 1H), 7.38 (t, J = 7.8 Hz, 2H), 7.21 (d, J = 8.4 Hz, 1H), 6.52 (d, J = 8.9 Hz, 1H), 6.22 (s, 1H), 3.52 (d, J = 17.0 Hz, 1H), 3.43 (dd, J = 17.2, 8.9 Hz, 1H).

^{13}C NMR (126 MHz, $CDCl_3$) δ 140.96, 140.36, 138.35, 137.02, 133.90, 133.56, 131.87, 129.54, 129.24, 126.71, 116.43, 106.32, 87.82, 75.29, 35.38.

HRMS (ESI) m/z $[M + H]^+$ calculated for $C_{17}H_{14}IN_3O_2S$: 451.9924, found 451.9922.

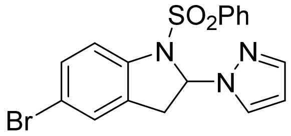

5-bromo-1-(phenylsulfonyl)-2-(1*H*-pyrazol-1-yl)indoline (41):

^1H NMR (500 MHz, $CDCl_3$) δ 7.60 (s, 1H), 7.46 (d, J = 8.0 Hz, 3H), 7.38 (s, 1H), 7.35 – 7.13 (m, 5H), 6.47 (d, J = 8.9 Hz, 1H), 6.15 (s, 1H), 3.47 (d, J = 17.1 Hz, 1H), 3.35 (dd, J = 17.3, 8.9 Hz, 1H).

^{13}C NMR (126 MHz, $CDCl_3$) δ 141.00, 139.72, 138.43, 133.62, 131.75, 131.14, 129.58, 129.31, 128.20, 126.79, 117.47, 116.13, 106.40, 75.57, 35.57.

HRMS (ESI) m/z $[M + H]^+$ calculated for $C_{17}H_{14}BrN_3O_2S$: 404.0063, found 404.0061.

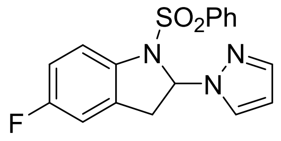

5-fluoro-1-(phenylsulfonyl)-2-(1*H*-pyrazol-1-yl)indoline (42):

^{1}H NMR (500 MHz, $CDCl_3$) δ 7.70 (s, 1H), 7.55 (t, J = 8.3 Hz, 3H), 7.49 – 7.22 (m, 4H), 7.00 – 6.77 (m, 2H), 6.53 (d, J = 8.9 Hz, 1H), 6.24 (s, 1H), 3.54 (d, J = 17.1 Hz, 1H), 3.35 (dd, J = 17.3, 9.0 Hz, 1H).

^{13}C NMR (126 MHz, $CDCl_3$) δ 161.34, 159.41, 140.91, 138.29, 136.50, 133.61, 131.87 (d, J = 8.8 Hz), 129.45, 129.31, 126.86, 116.30 (d, J = 8.6 Hz), 114.84 (d, J = 23.5 Hz), 112.53 (d, J = 24.7 Hz), 76.01, 35.73.

HRMS (ESI) m/z $[M + H]^+$ calculated for $C_{17}H_{14}FN_3O_2S$: 344.0864, found 344.0861.

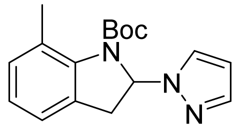

***tert*-butyl 7-methyl-2-(1*H*-pyrazol-1-yl)indoline-1-carboxylate (43):**

^{1}H NMR (500 MHz, DMSO-d_6) δ 7.69 (d, J = 2.4 Hz, 1H), 7.41 (d, J = 1.7 Hz, 1H), 7.12 (dd, J = 6.3, 2.5 Hz, 1H), 7.07 – 6.92 (m, 2H), 6.67 (dd, J = 8.3, 1.3 Hz, 1H), 6.24 (t, J = 2.1 Hz, 1H), 3.78 (dd, J = 16.7, 8.3 Hz, 1H), 3.32 (d, J = 16.8 Hz, 1H), 2.18 (s, 3H), 1.51 (s, 9H).

^{13}C NMR (126 MHz, DMSO) δ 152.24, 139.99, 139.48, 131.88, 129.65, 128.78, 126.67, 124.51, 121.75, 105.40, 81.45, 75.63, 35.71, 27.82, 19.83.

HRMS (ESI) m/z $[M + H]^+$ calculated for $C_{17}H_{21}N_3O_2$: 300.1707, found 300.1705.

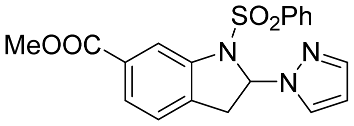

methyl 1-(phenylsulfonyl)-2-(1*H*-pyrazol-1-yl)indoline-6-carboxylate (44):

^{1}H NMR (500 MHz, $CDCl_3$) δ 7.97 (d, J = 3.2 Hz, 1H), 7.68 (dd, J = 7.9, 3.1 Hz, 1H), 7.56 (d, J = 3.2 Hz, 1H), 7.50 – 7.36 (m, 3H), 7.33 (d, J = 3.0 Hz, 1H), 7.29 – 7.21 (m, 2H), 7.18 – 7.01 (m, 1H), 6.51 (dd, J = 9.2, 3.2 Hz, 1H), 6.11 (t, J = 2.6 Hz, 1H), 3.80 (d, J = 2.6 Hz, 3H), 3.48 (d, J = 17.3 Hz, 1H), 3.43 – 3.31 (m, 1H).

^{13}C NMR (126 MHz, $CDCl_3$) δ 166.58, 141.00, 140.90, 138.53, 134.72, 133.59, 130.57, 129.61, 129.28, 126.84, 126.37, 124.91, 115.28, 106.36, 75.58, 52.39, 35.99.

HRMS (ESI) m/z $[M + H]^+$ calculated for $C_{19}H_{17}N_3O_4S$: 384.1013, found 384.1010.

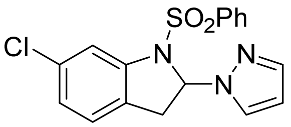

6-chloro-1-(phenylsulfonyl)-2-(1*H*-pyrazol-1-yl)indoline (45):

^{1}H NMR (500 MHz, $CDCl_3$) δ 7.65 (s, 1H), 7.53 (d, *J* = 8.0 Hz, 3H), 7.44 (d, *J* = 3.8 Hz, 2H), 7.38 (t, *J* = 7.6 Hz, 2H), 7.10 (d, *J* = 8.0 Hz, 1H), 7.03 (d, *J* = 8.1 Hz, 1H), 6.58 (d, *J* = 8.2 Hz, 1H), 6.21 (s, 1H), 3.74 – 3.04 (m, 2H).

^{13}C NMR (126 MHz, $CDCl_3$) δ 141.65, 141.01, 138.50, 133.90, 133.64, 129.56, 129.32, 127.79, 126.77, 125.79, 124.59, 114.99, 106.37, 75.88, 35.53.

HRMS (ESI) m/z $[M + H]^+$ calculated for $C_{17}H_{14}ClN_3O_2S$: 360.0568, found 360.0566.

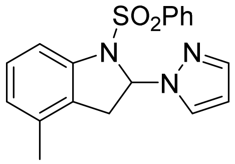

4-methyl-1-(phenylsulfonyl)-2-(1*H*-pyrazol-1-yl)indoline (46):

^{1}H NMR (500 MHz, $CDCl_3$) δ 7.57 (d, *J* = 2.5 Hz, 1H), 7.50 – 7.45 (m, 2H), 7.43 – 7.32 (m, 2H), 7.32 – 7.19 (m, 3H), 7.04 (t, *J* = 7.9 Hz, 1H), 6.78 (d, *J* = 7.6 Hz, 1H), 6.49 (dd, *J* = 9.0, 2.4 Hz, 1H), 6.12 (t, *J* = 2.1 Hz, 1H), 3.34 (dd, *J* = 16.9, 2.4 Hz, 1H), 3.25 (dd, *J* = 17.0, 9.0 Hz, 1H), 2.08 (s, 3H).

^{13}C NMR (126 MHz, $CDCl_3$) δ 140.71, 140.16, 138.66, 134.75, 133.36, 129.26, 129.16, 128.33, 127.92, 126.87, 125.72, 112.20, 106.19, 75.56, 35.19, 18.80.

HRMS (ESI) m/z $[M + H]^+$ calculated for $C_{18}H_{17}N_3O_2S$: 340.1114, found 340.1111.

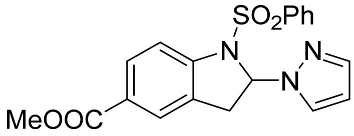

methyl 1-(phenylsulfonyl)-2-(1*H*-pyrazol-1-yl)indoline-5-carboxylate (47):

^{1}H NMR (500 MHz, $CDCl_3$) δ 7.85 (dd, *J* = 8.5, 1.8 Hz, 1H), 7.79 (d, *J* = 1.7 Hz, 1H), 7.55 (d, *J* = 2.5 Hz, 1H), 7.47 – 7.38 (m, 3H), 7.37 – 7.31 (m, 2H), 7.26 (t, *J* = 7.8 Hz, 2H), 6.56 (t, *J* = 5.8 Hz, 1H), 6.12 (t, *J* = 2.1 Hz, 1H), 3.78 (s, 3H), 3.48 (d, *J* = 5.7 Hz, 2H).

^{13}C NMR (126 MHz, $CDCl_3$) δ 166.54, 144.47, 141.07, 138.61, 133.61, 130.58, 129.74, 129.34, 129.23, 126.74, 126.52, 126.20, 113.40, 106.33, 75.69, 52.15, 35.63.

HRMS (ESI) m/z $[M + H]^+$ calculated for $C_{19}H_{17}N_3O_4S$: 384.1013, found 384.1012.

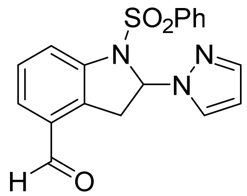

1-(phenylsulfonyl)-2-(1*H*-pyrazol-1-yl)indoline-4-carbaldehyde (48):

^{1}H NMR (500 MHz, $CDCl_3$) δ 10.04 (s, 1H), 7.68 (d, *J* = 8.0 Hz, 2H), 7.52 (dd, *J* = 14.4, 7.7 Hz, 4H), 7.48 – 7.41 (m, 2H), 7.37 (t, *J* = 7.8 Hz, 2H), 6.66 (d, *J* = 9.3 Hz, 1H), 6.23 (d, *J* = 2.8 Hz, 1H), 4.02 (d, *J* = 18.7 Hz, 1H), 3.76 (dd, *J* = 18.8, 9.4 Hz, 1H).

^{13}C NMR (126 MHz, $CDCl_3$) δ 192.11, 142.19, 141.26, 138.64, 133.67, 132.58, 130.47, 129.94, 129.33, 129.03, 128.34, 126.81, 119.39, 106.28, 75.94, 36.12.

HRMS (ESI) m/z $[M + H]^+$ calculated for $C_{18}H_{15}N_3O_3S$: 354.0907, found 354.0905.

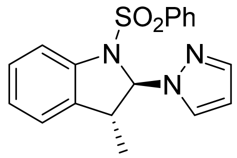

***trans*-3-methyl-1-(phenylsulfonyl)-2-(1*H*-pyrazol-1-yl)indoline (49):**

^{1}H NMR (500 MHz, $CDCl_3$) δ 7.76 (dd, *J* = 17.0, 8.0 Hz, 3H), 7.66 (t, *J* = 7.5 Hz, 1H), 7.52 (dd, *J* = 17.0, 9.3 Hz, 3H), 7.45 (dd, *J* = 11.4, 5.4 Hz, 1H), 7.37 – 7.18 (m, 3H), 6.74 (d, *J* = 8.2 Hz, 1H), 6.27 (t, *J* = 2.0 Hz, 1H), 3.81 (p, *J* = 7.6 Hz, 1H), 1.07 (d, *J* = 7.1 Hz, 3H).

^{13}C NMR (126 MHz, $CDCl_3$) δ 140.77, 139.61, 138.74, 133.67, 133.29, 129.12, 128.41, 128.32, 126.92, 124.59, 123.84, 113.88, 106.15, 79.70, 41.31, 10.53.

HRMS (ESI) m/z $[M + H]^+$ calculated for $C_{18}H_{17}N_3O_2S$: 340.1114, found 340.1113.

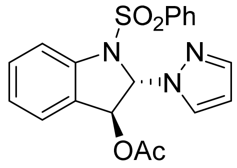

***trans*-1-(phenylsulfonyl)-2-(1*H*-pyrazol-1-yl)indolin-3-yl acetate (50):**

^{1}H NMR (500 MHz, $CDCl_3$) δ 7.50 (d, *J* = 7.9 Hz, 3H), 7.39 (t, *J* = 7.8 Hz, 1H), 7.25 (q, *J* = 9.8, 8.6 Hz, 6H), 7.13 (d, *J* = 7.7 Hz, 1H), 7.01 (dt, *J* = 7.6, 3.7 Hz, 1H), 6.58 (dd, *J* = 7.6, 2.6 Hz, 1H), 6.12 (d, *J* = 7.6 Hz, 1H), 6.03 (s, 1H), 1.64 (d, *J* = 2.7 Hz, 3H).

^{13}C NMR (126 MHz, $CDCl_3$) δ 170.45, 140.65, 140.38, 138.02, 133.59, 130.68, 130.05, 129.21, 127.06, 126.72, 125.39, 124.72, 114.19, 106.00, 76.16, 72.68, 19.96.

HRMS (ESI) m/z $[M + H]^+$ calculated for $C_{19}H_{17}N_3O_4S$: 384.1013, found 384.1012.

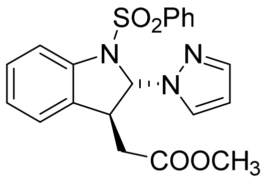

methyl 2-1-(phenylsulfonyl)-2-(1*H*-pyrazol-1-yl)indolin-3-yl)acetate (51):

^{1}H NMR (500 MHz, $CDCl_3$) δ 7.57 – 7.31 (m, 5H), 7.30 – 7.12 (m, 6H), 6.98 (q, *J* = 8.6, 7.9 Hz, 2H), 6.78 – 6.51 (m, 1H), 6.03 (s, 1H), 4.30 – 3.82 (m, 1H), 3.53 (d, *J* = 2.4 Hz, 3H), 2.61 (dd, *J* = 17.5, 5.8 Hz, 1H), 2.25 – 1.95 (m, 1H).

^{13}C NMR (126 MHz, $CDCl_3$) δ 172.38, 140.95, 140.78, 138.85, 133.28, 131.11, 130.26, 129.08, 128.76, 126.85, 124.32, 123.37, 113.71, 105.74, 77.94, 52.05, 42.53, 31.36.

HRMS (ESI) m/z $[M + H]^+$ calculated for $C_{20}H_{19}N_3O_4S$: 398.1169, found 398.1168.

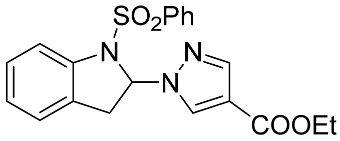

Ethyl 1-(1-(phenylsulfonyl)indolin-2-yl)-1H-pyrazole-4-carboxylate (52):

^{1}H NMR (500 MHz, $CDCl_3$) δ 8.13 (d, *J* = 0.6 Hz, 1H), 7.84 (d, *J* = 0.6 Hz, 1H), 7.66 – 7.61 (m, 2H), 7.59 (d, *J* = 8.1 Hz, 1H), 7.56 – 7.49 (m, 1H), 7.43 – 7.35 (m, 2H), 7.32 – 7.25 (m, 1H), 7.17 (dd, *J* = 7.5, 1.4 Hz, 1H), 7.10 (td, *J* = 7.5, 1.0 Hz, 1H), 6.48 (dd, *J* = 8.5, 2.3 Hz, 1H), 4.27 (qd, *J* = 7.1, 1.6 Hz, 2H), 3.71 – 3.23 (m, 2H), 1.33 (t, *J* = 7.1 Hz, 3H).

^{13}C NMR (126 MHz, $CDCl_3$) δ 162.79, 142.10, 140.37, 138.26, 133.73, 132.17, 129.35, 128.98, 128.54, 126.87, 125.25, 125.23, 115.76, 115.49, 76.18, 60.42, 36.14, 14.52.

HRMS (ESI) m/z $[M + H]^+$ calculated for $C_{19}H_{17}N_3O_4S$: 384.1013, found 384.1012.

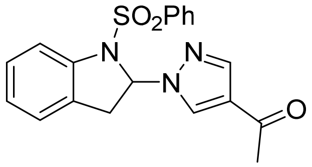

1-(1-(1-(phenylsulfonyl)indolin-2-yl)-1*H*-pyrazol-4-yl)ethan-1-one (53):

^{1}H NMR (500 MHz, $CDCl_3$) δ 8.00 (s, 1H), 7.71 (s, 1H), 7.61 – 7.50 (m, 2H), 7.47 (d, *J* = 8.1 Hz, 1H), 7.44 – 7.37 (m, 1H), 7.32 – 7.22 (m, 2H), 7.20 – 7.11 (m, 1H), 7.04 (d, *J* = 8.0 Hz, 1H), 6.97 (td, *J* = 7.5, 0.9 Hz, 1H), 6.36 (dd, *J* = 8.6, 2.2 Hz, 1H), 3.46 – 3.18 (m, 2H), 2.25 (s, 3H).

^{13}C NMR (126 MHz, $CDCl_3$) δ 191.94, 141.21, 140.27, 138.12, 133.77, 131.41, 129.35, 128.95, 128.54, 126.81, 125.31, 125.26, 124.47, 115.52, 76.23, 36.04, 28.06.

HRMS (ESI) m/z $[M + H]^+$ calculated for $C_{19}H_{17}N_3O_3S$: 368.1063, found 368.1061.

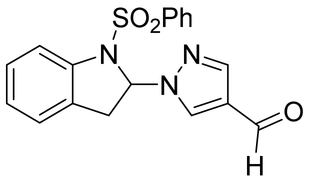

1-(1-(phenylsulfonyl)indolin-2-yl)-1*H*-pyrazole-4-carbaldehyde (54):

^{1}H NMR (500 MHz, $CDCl_3$) δ 9.67 (s, 1H), 8.08 (s, 1H), 7.77 (s, 1H), 7.57 – 7.50 (m, 2H), 7.47 (d, *J* = 8.1 Hz, 1H), 7.45 – 7.38 (m, 1H), 7.26 (t, *J* = 8.0 Hz, 2H), 7.19 – 7.09 (m, 1H), 7.03 (d, *J* = 8.0 Hz, 1H), 6.97 (td, *J* = 7.5, 1.0 Hz, 1H), 6.37 (dd, *J* = 8.6, 2.1 Hz, 1H), 3.35 (dd, *J* = 17.2, 2.1 Hz, 1H), 3.27 (dd, *J* = 17.1, 8.6 Hz, 1H).

^{13}C NMR (126 MHz, $CDCl_3$) δ 184.02, 141.11, 140.21, 138.05, 133.87, 132.89, 129.42, 128.88, 128.61, 126.83, 125.41, 125.31, 124.54, 115.56, 76.36, 36.06.

HRMS (ESI) m/z $[M + H]^+$ calculated for $C_{18}H_{15}N_3O_3S$: 354.0907, found 354.0905.

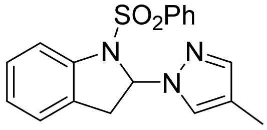

2-(4-methyl-1*H*-pyrazol-1-yl)-1-(phenylsulfonyl)indoline (55):

^{1}H NMR (500 MHz, $CDCl_3$) δ 7.58 (dd, *J* = 8.5, 1.3 Hz, 2H), 7.55 – 7.47 (m, 2H), 7.44 – 7.32 (m, 3H), 7.25 – 7.20 (m, 2H), 7.18 (d, *J* = 7.5 Hz, 1H), 7.11 – 7.02 (m, 1H), 6.47 (dd, *J* = 8.8, 2.2 Hz, 1H), 3.73 – 3.21 (m, 2H), 1.99 (s, 3H).

^{13}C NMR (126 MHz, $CDCl_3$) δ 141.18, 140.59, 138.80, 133.36, 129.51, 129.14, 128.25, 127.67, 126.92, 125.15, 124.73, 116.98, 115.06, 75.67, 35.96, 8.92.

HRMS (ESI) m/z $[M + H]^+$ calculated for $C_{18}H_{17}N_3O_2S$: 340.1114, found 340.1114.

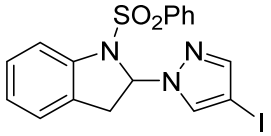

2-(4-iodo-1*H*-pyrazol-1-yl)-1-(phenylsulfonyl)indoline (56):

^{1}H NMR (500 MHz, $CDCl_3$) δ 7.51 – 7.35 (m, 5H), 7.30 – 7.20 (m, 3H), 7.18 – 7.08 (m, 1H), 7.03 (dd, *J* = 7.6, 1.4 Hz, 1H), 6.94 (td, *J* = 7.5, 1.0 Hz, 1H), 6.35 (dd, *J* = 8.3, 2.5 Hz, 1H), 3.37 – 3.12 (m, 2H).

^{13}C NMR (126 MHz, $CDCl_3$) δ 145.33, 140.35, 138.33, 133.61, 133.28, 129.23, 128.99, 128.40, 126.73, 125.18, 124.95, 115.09, 75.95, 57.57, 35.89.

HRMS (ESI) m/z $[M + H]^+$ calculated for $C_{17}H_{14}IN_3O_2S$: 451.9924, found 451.9922.

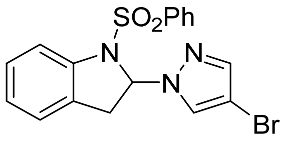

2-(4-bromo-1*H*-pyrazol-1-yl)-1-(phenylsulfonyl)indoline (57):

^{1}H NMR (500 MHz, $CDCl_3$) δ 7.58 – 7.49 (m, 3H), 7.48 – 7.38 (m, 2H), 7.32 – 7.23 (m, 2H), 7.21 – 7.11 (m, 1H), 7.06 (dd, *J* = 7.2, 1.4 Hz, 1H), 6.97 (td, *J* = 7.5, 1.0 Hz, 1H), 6.35 (dd, *J* = 8.6, 2.1 Hz, 1H), 3.36 (dd, *J* = 17.1, 2.2 Hz, 1H), 3.28 (dd, *J* = 17.1, 8.6 Hz, 1H).

^{13}C NMR (126 MHz, $CDCl_3$) δ 140.91, 140.32, 138.31, 133.62, 129.24, 129.04, 129.02, 128.40, 126.76, 125.17, 125.00, 115.19, 94.25, 76.17, 35.79.

HRMS (ESI) m/z $[M + H]^+$ calculated for $C_{17}H_{14}BrN_3O_2S$: 404.0063, found 404.0062.

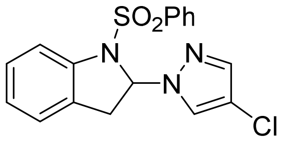

2-(4-chloro-1*H*-pyrazol-1-yl)-1-(phenylsulfonyl)indoline (58):

^{1}H NMR (500 MHz, $CDCl_3$) δ 7.61 – 7.38 (m, 5H), 7.38 – 7.23 (m, 3H), 7.22 – 7.14 (m, 1H), 7.09 (d, *J* = 7.5 Hz, 1H), 7.00 (t, *J* = 7.5 Hz, 1H), 6.35 (dd, *J* = 8.6, 2.1 Hz, 1H), 3.39 (dd, *J* = 17.1, 2.0 Hz, 1H), 3.29 (dd, *J* = 17.0, 8.6 Hz, 1H).

^{13}C NMR (126 MHz, $CDCl_3$) δ 140.34, 138.88, 138.36, 133.64, 129.27, 129.08, 128.44, 126.87, 126.80, 125.18, 125.04, 115.27, 111.06, 76.27, 35.76.

HRMS (ESI) m/z $[M + H]^+$ calculated for $C_{17}H_{14}ClN_3O_2S$: 360.0568, found 360.0566.

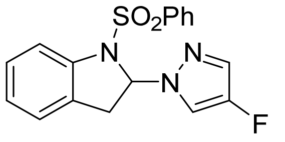

2-(4-fluoro-1*H*-pyrazol-1-yl)-1-(phenylsulfonyl)indoline (59):

^{1}H NMR (500 MHz, $CDCl_3$) δ 7.51 (dd, *J* = 8.1, 1.5 Hz, 2H), 7.46 – 7.34 (m, 3H), 7.26 (t, *J* = 7.8 Hz, 2H), 7.20 – 7.09 (m, 2H), 7.04 (d, *J* = 7.5 Hz, 1H), 6.95 (t, *J* = 7.5 Hz, 1H), 6.27 (dd, *J* = 8.9, 2.0 Hz, 1H), 3.36 (dd, *J* = 17.1, 2.1 Hz, 1H), 3.23 (dd, *J* = 17.0, 8.8 Hz, 1H).

^{13}C NMR (126 MHz, $CDCl_3$) δ 150.90, 148.93, 140.34, 138.43, 133.59, 129.25, 128.35, 127.69 (d, *J* = 14.1 Hz), 126.82, 125.12, 125.01, 115.36, 114.88 (d, *J* = 28.0 Hz), 76.59, 35.48.

HRMS (ESI) m/z $[M + H]^+$ calculated for $C_{17}H_{14}FN_3O_2S$: 344.0864, found 344.0863.

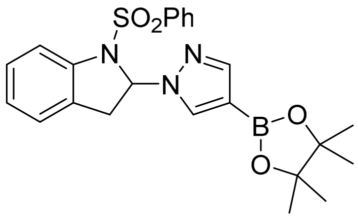

1-(phenylsulfonyl)-2-(4-(4,4,5,5-tetramethyl-1,3,2-dioxaborolan-2-yl)-1*H*-pyrazol-1-yl)indoline (60):

^{1}H NMR (500 MHz, $CDCl_3$) δ 7.81 (s, 1H), 7.62 (s, 1H), 7.52 – 7.48 (m, 2H), 7.46 (d, *J* = 8.1 Hz, 1H), 7.43 – 7.37 (m, 1H), 7.26 (t, *J* = 7.9 Hz, 2H), 7.16 (dd, *J* = 8.1, 6.8 Hz, 1H), 7.06 (dd, *J* = 7.4, 1.4 Hz, 1H), 6.98 (td, *J* = 7.5, 1.0 Hz, 1H), 6.45 (t, *J* = 5.5 Hz, 1H), 3.32 (d, *J* = 5.5 Hz, 2H), 1.20 (s, 12H).

^{13}C NMR (126 MHz, $CDCl_3$) δ 146.47, 140.57, 138.52, 135.59, 133.44, 129.19, 129.15, 128.33, 126.87, 125.22, 124.89, 115.24, 83.49, 75.69, 36.40, 24.98, 24.94, 24.92, 24.82.

HRMS (ESI) m/z $[M + H]^+$ calculated for $C_{23}H_{26}BN_3O_4S$: 452.1810, found 452.1809.

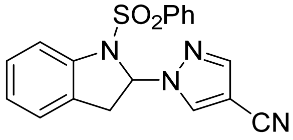

1-(1-(phenylsulfonyl)indolin-2-yl)-1*H*-pyrazole-4-carbonitrile (61):

^{1}H NMR (500 MHz, $CDCl_3$) δ 7.81 (s, 1H), 7.62 (s, 1H), 7.40 (s, 1H), 7.32 (d, *J* = 7.6 Hz, 2H), 7.29 – 7.19 (m, 1H), 7.09 (t, *J* = 7.8 Hz, 2H), 6.96 (t, *J* = 7.8 Hz, 1H), 6.84 (d, *J* = 7.5 Hz, 1H), 6.78 (t, *J* = 7.5 Hz, 1H), 6.17 (dd, *J* = 8.6, 2.2 Hz, 1H), 3.22 – 2.98 (m, 2H).

^{13}C NMR (126 MHz, $CDCl_3$) δ 143.08, 139.95, 138.18, 137.71, 134.25, 134.03, 129.48, 128.68, 126.76, 125.52, 125.29, 115.52, 113.07, 92.99, 76.42, 35.91.

HRMS (ESI) m/z $[M + H]^+$ calculated for $C_{18}H_{14}N_4O_2S$: 351.0910, found 351.0909.

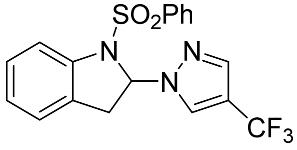

1-(phenylsulfonyl)-2-(4-(trifluoromethyl)-1*H*-pyrazol-1-yl)indoline (62):

^{1}H NMR (500 MHz, $CDCl_3$) δ 7.81 (s, 1H), 7.58 – 7.35 (m, 5H), 7.27 (d, *J* = 8.2 Hz, 2H), 7.15 (q, *J* = 7.5, 6.4 Hz, 1H), 7.06 (d, *J* = 7.5 Hz, 1H), 7.02 – 6.87 (m, 1H), 6.57 – 6.06 (m, 1H), 3.52 – 3.14 (m, 2H).

^{13}C NMR (126 MHz, $CDCl_3$) δ 140.33, 138.29, 137.98 (d, *J* = 2.7 Hz), 133.79, 129.37, 128.89, 128.59, 128.40 (d, *J* = 3.7 Hz), 126.76, 125.25 (d, *J* = 4.8 Hz), 122.41 (q, *J* = 266.2 Hz), 115.35, 114.30 (q, *J* = 38.1 Hz), 76.21, 36.06.

HRMS (ESI) m/z $[M + H]^+$ calculated for $C_{18}H_{14}F_3N_3O_2S$: 394.0832, found 394.0831.

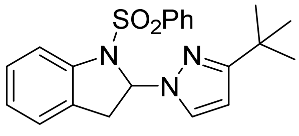

2-(3-(*tert*-butyl)-1*H*-pyrazol-1-yl)-1-(phenylsulfonyl)indoline (63):

^{1}H NMR (500 MHz, $CDCl_3$) δ 7.57 (d, J = 7.8 Hz, 2H), 7.50 (dd, J = 9.8, 7.5 Hz, 2H), 7.42 (t, J = 2.0 Hz, 1H), 7.38 – 7.31 (m, 2H), 7.23 (t, J = 7.8 Hz, 1H), 7.15 (d, J = 7.5 Hz, 1H), 7.05 (t, J = 7.4 Hz, 1H), 6.54 (dt, J = 8.6, 2.1 Hz, 1H), 6.01 (t, J = 2.0 Hz, 1H), 3.46 (d, J = 16.8 Hz, 1H), 3.38 (dd, J = 17.0, 8.7 Hz, 1H), 1.24 (d, J = 1.8 Hz, 9H).

^{13}C NMR (126 MHz, $CDCl_3$) δ 162.76, 140.70, 138.87, 133.30, 129.69, 129.13, 128.61, 128.03, 126.90, 125.18, 124.52, 115.00, 102.69, 75.91, 36.45, 32.27, 30.62.

HRMS (ESI) m/z $[M + H]^+$ calculated for $C_{21}H_{23}N_3O_2S$: 382.1583, found 392.1582.

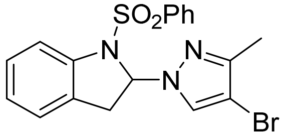

2-(4-bromo-3-methyl-1*H*-pyrazol-1-yl)-1-(phenylsulfonyl)indoline (64):

^{1}H NMR (500 MHz, $CDCl_3$) δ 7.63 (dtd, J = 54.0, 11.1, 9.9, 5.3 Hz, 5H), 7.43 (dt, J = 8.3, 5.0 Hz, 2H), 7.32 (q, J = 9.9 Hz, 1H), 7.29 – 7.19 (m, 1H), 7.14 (td, J = 7.5, 3.0 Hz, 1H), 6.52 (dd, J = 66.8, 8.9 Hz, 1H), 3.98 – 3.10 (m, 2H), 2.32 (dd, J = 148.6, 3.3 Hz, 3H).

^{13}C NMR (126 MHz, $CDCl_3$) δ 148.38, 140.71 (d, J = 45.1 Hz), 139.58 (d, J = 210.1 Hz), 138.08 (d, J = 72.2 Hz), 133.44 (d, J = 21.1 Hz), 129.13 (d, J = 7.3 Hz), 128.98 (d, J = 16.3 Hz), 128.26 (d, J = 20.5 Hz), 126.69 (d, J = 25.7 Hz), 124.79 (d, J = 14.5 Hz), 124.78 (d, J = 119.9 Hz), 114.60 (d, J = 146.3 Hz), 94.78 (d, J = 63.7 Hz), 74.63 (d, J = 385.2 Hz), 36.16 (d, J = 52.5 Hz), 10.94 (d, J = 276.0 Hz).

HRMS (ESI) m/z $[M + H]^+$ calculated for $C_{18}H_{16}BrN_3O_2S$: 418.0219, found 418.0217.

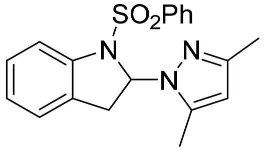

2-(3,5-dimethyl-1*H*-pyrazol-1-yl)-1-(phenylsulfonyl)indoline (65):

^{1}H NMR (500 MHz, $CDCl_3$) δ 7.61 – 7.50 (m, 2H), 7.46 (dd, J = 7.8, 6.4 Hz, 2H), 7.33 (t, J = 7.9 Hz, 2H), 7.25 – 7.18 (m, 1H), 7.16 (dd, J = 7.5, 1.4 Hz, 1H), 7.03 (td, J = 7.5, 1.0 Hz, 1H), 6.48 (dd, J = 9.8, 3.1 Hz, 1H), 5.70 (s, 1H), 3.52 (dd, J = 16.8, 9.8 Hz, 1H), 3.34 (dd, J = 16.8, 3.1 Hz, 1H), 2.36 (s, 3H), 1.99 (s, 3H).

^{13}C NMR (126 MHz, $CDCl_3$) δ 149.15, 141.13, 140.12, 139.09, 132.99, 129.05, 128.81, 127.87, 126.83, 124.81, 123.73, 113.76, 106.06, 72.20, 36.45, 13.68, 11.10.

HRMS (ESI) m/z $[M + H]^+$ calculated for $C_{19}H_{19}N_3O_2S$: 354.1271, found 354.1269.

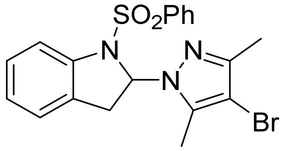

2-(4-bromo-3,5-dimethyl-1*H*-pyrazol-1-yl)-1-(phenylsulfonyl)indoline (66):

^1H NMR (500 MHz, $CDCl_3$) δ 7.43 (dt, *J* = 20.8, 7.9 Hz, 4H), 7.26 (t, *J* = 7.8 Hz, 2H), 7.17 (t, *J* = 7.8 Hz, 1H), 7.09 (d, *J* = 7.4 Hz, 1H), 6.97 (t, *J* = 7.5 Hz, 1H), 6.39 (dd, *J* = 9.7, 2.8 Hz, 1H), 3.46 (dd, *J* = 16.9, 9.8 Hz, 1H), 3.22 (dd, *J* = 16.9, 2.9 Hz, 1H), 2.25 (s, 3H), 1.85 (s, 3H).

^{13}C NMR (126 MHz, $CDCl_3$) δ 147.57, 141.16, 138.83, 138.03, 133.15, 128.79, 128.76, 128.07, 126.61, 124.82, 123.90, 113.72, 95.66, 73.07, 36.29, 12.40, 10.43.

HRMS (ESI) m/z $[M + H]^+$ calculated for $C_{19}H_{18}BrN_3O_2S$: 432.0376, found 432.0375.

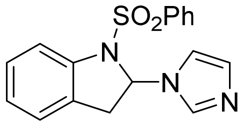

2-(1*H*-imidazol-1-yl)-1-(phenylsulfonyl)indoline (67):

^1H NMR (500 MHz, $CDCl_3$) δ 7.53 (s, 1H), 7.41 (d, *J* = 8.0 Hz, 3H), 7.36 (d, *J* = 8.1 Hz, 1H), 7.26 (t, *J* = 7.7 Hz, 2H), 7.16 (dd, *J* = 9.3, 6.4 Hz, 1H), 7.10 (d, *J* = 7.5 Hz, 1H), 6.98 (t, *J* = 7.5 Hz, 1H), 6.80 (s, 1H), 6.59 (s, 1H), 6.37 (d, *J* = 8.9 Hz, 1H), 3.47 (dd, *J* = 17.2, 9.0 Hz, 1H), 3.02 (d, *J* = 17.1 Hz, 1H).

^{13}C NMR (126 MHz, $CDCl_3$) δ 140.11, 138.52, 136.10, 133.57, 130.44, 129.33, 128.74, 127.97, 126.69, 125.26, 124.68, 116.01, 114.58, 71.69, 37.18.

HRMS (ESI) m/z $[M + H]^+$ calculated for $C_{17}H_{15}N_3O_2S$: 181.0972, found 181.0970.

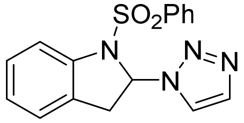

1-(phenylsulfonyl)-2-(1*H*-1,2,3-triazol-1-yl)indoline (68):

^1H NMR (500 MHz, $CDCl_3$) δ 7.76 (d, *J* = 1.1 Hz, 1H), 7.66 (dd, *J* = 8.2, 1.5 Hz, 2H), 7.62 (d, *J* = 8.0 Hz, 2H), 7.59 – 7.50 (m, 1H), 7.42 (t, *J* = 7.8 Hz, 2H), 7.29 (t, *J* = 7.8 Hz, 1H), 7.19 (d, *J* = 7.5 Hz, 1H), 7.12 (t, *J* = 7.5 Hz, 1H), 6.77 (dd, *J* = 8.7, 1.7 Hz, 1H), 3.66 – 3.59 (m, 1H), 3.43 (dd, *J* = 17.2, 8.7 Hz, 1H).

^{13}C NMR (126 MHz, $CDCl_3$) δ 140.07, 137.90, 134.27, 133.95, 129.52, 129.00, 128.72, 126.91, 125.60, 125.45, 122.57, 116.04, 74.97, 36.23.

HRMS (ESI) m/z $[M + H]^+$ calculated for $C_{17}H_{15}N_4O_2S$: 327.0910, found 327.0908.

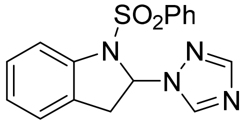

1-(phenylsulfonyl)-2-(1*H*-1,2,4-triazol-1-yl)indoline (69):

^1H NMR (500 MHz, $CDCl_3$) δ 8.25 (s, 1H), 7.70 (s, 1H), 7.57 – 7.46 (m, 2H), 7.45 – 7.37 (m, 2H), 7.26 (t, *J* = 7.9 Hz, 2H), 7.13 (t, *J* = 7.8 Hz, 1H), 7.04 (d, *J* = 7.5 Hz, 1H), 6.96 (t, *J* = 7.4 Hz, 1H), 6.45 (dd, *J* = 8.1, 2.7 Hz, 1H), 3.48 – 3.13 (m, 2H).

^{13}C NMR (126 MHz, $CDCl_3$) δ 152.53, 143.13, 140.06, 138.07, 133.75, 129.35, 128.70, 128.48, 126.67, 125.18, 125.14, 115.25, 73.91, 35.74.

HRMS (ESI) m/z $[M + H]^+$ calculated for $C_{17}H_{15}N_4O_2S$: 327.0910, found 327.0910.

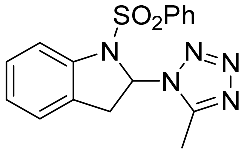

2-(5-methyl-1*H*-tetrazol-1-yl)-1-(phenylsulfonyl)indoline (70):

^{1}H NMR (500 MHz, $CDCl_3$) δ 7.66 (dd, *J* = 21.4, 7.9 Hz, 3H), 7.54 (t, *J* = 7.7 Hz, 1H), 7.39 (t, *J* = 7.7 Hz, 2H), 7.31 (t, *J* = 7.9 Hz, 1H), 7.21 (d, *J* = 7.5 Hz, 1H), 7.12 (t, *J* = 7.5 Hz, 1H), 7.04 (d, *J* = 9.1 Hz, 1H), 3.60 (dd, *J* = 17.3, 9.2 Hz, 1H), 3.34 (d, *J* = 17.1 Hz, 1H), 2.36 (d, *J* = 2.6 Hz, 3H).

^{13}C NMR (126 MHz, $CDCl_3$) δ 163.66, 140.60, 138.20, 133.70, 129.28, 128.64, 128.06, 126.89, 125.12, 124.95, 115.00, 76.29, 36.11, 10.97.

^{1}H NMR (500 MHz, $CDCl_3$) δ 7.86 – 7.51 (m, 4H), 7.42 (t, *J* = 7.8 Hz, 2H), 7.30 (t, *J* = 8.0 Hz, 1H), 7.20 (d, *J* = 7.6 Hz, 1H), 7.15 (d, *J* = 7.7 Hz, 1H), 6.69 (d, *J* = 8.9 Hz, 1H), 3.54 (dd, *J* = 17.3, 9.2 Hz, 1H), 3.34 (d, *J* = 17.2 Hz, 1H), 2.66 (s, 3H).

^{13}C NMR (126 MHz, $CDCl_3$) δ 151.59, 139.90, 137.46, 134.12, 129.53, 128.76, 128.36, 126.67, 125.51, 125.02, 115.46, 72.25, 36.49, 9.36.

HRMS (ESI) m/z $[M + H]^+$ calculated for $C_{16}H_{15}N_5O_2S$: 342.1019, found 342.1018.

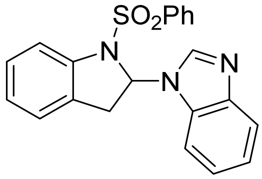

1-(1-(phenylsulfonyl)indolin-2-yl)-1*H*-benzo[d]imidazole (71):

^{1}H NMR (500 MHz, $CDCl_3$) δ 7.86 (s, 1H), 7.58 (d, *J* = 8.1 Hz, 1H), 7.49 (d, *J* = 8.1 Hz, 1H), 7.26 (t, *J* = 7.0 Hz, 3H), 7.21 – 7.11 (m, 2H), 7.02 (dt, *J* = 23.5, 7.6 Hz, 4H), 6.86 (t, *J* = 7.7 Hz, 1H), 6.66 (dd, *J* = 10.1, 2.7 Hz, 1H), 6.55 (d, *J* = 8.2 Hz, 1H), 3.62 (dd, *J* = 17.4, 9.9 Hz, 1H), 3.19 (dd, *J* = 17.4, 2.6 Hz, 1H).

^{13}C NMR (126 MHz, $CDCl_3$) δ 144.30, 142.36, 140.75, 138.15, 133.34, 131.27, 128.99, 128.94, 127.82, 126.20, 125.17, 124.65, 123.49, 122.74, 120.67, 114.05, 110.86, 71.61, 35.78.

HRMS (ESI) m/z $[M + H]^+$ calculated for $C_{21}H_{17}N_3O_2S$: 376.1114, found 376.1112.

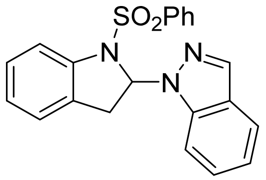

1-(1-(phenylsulfonyl)indolin-2-yl)-1*H*-indazole (72):

^{1}H NMR (500 MHz, $CDCl_3$) δ 8.16 (s, 1H), 7.66 (d, *J* = 8.1 Hz, 1H), 7.58 (dt, *J* = 16.7, 8.9 Hz, 4H), 7.43 (t, *J* = 7.4 Hz, 1H), 7.26 (qd, *J* = 13.2, 11.4, 8.1 Hz, 4H), 7.16 (d, *J* = 7.4 Hz, 1H), 7.10 (t, *J* = 7.5 Hz, 1H), 7.03 (t, *J* = 7.6 Hz, 1H), 6.79 (dd, *J* = 8.7, 2.4 Hz, 1H), 3.58 (d, *J* = 17.0 Hz, 1H), 3.49 (dd, *J* = 17.2, 8.6 Hz, 1H).

^{13}C NMR (126 MHz, $CDCl_3$) δ 149.25, 140.69, 138.08, 133.60, 129.27, 129.12, 128.46, 126.84, 126.62, 125.43, 125.16, 122.16, 122.06, 121.67, 120.67, 117.86, 115.49, 77.35, 37.06.

HRMS (ESI) m/z $[M + H]^+$ calculated for $C_{21}H_{17}N_3O_2S$: 376.1114, found 376.1114.

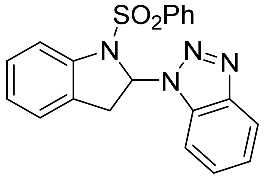

1-(1-(phenylsulfonyl)indolin-2-yl)-1*H*-benzo[*d*][1,2,3]triazole (73):

^{1}H NMR (500 MHz, Chloroform-*d*) δ 8.01 (dd, *J* = 6.3, 3.2 Hz, 1H), 7.73 (d, *J* = 8.1 Hz, 1H), 7.58 (d, *J* = 7.8 Hz, 2H), 7.42 (q, *J* = 8.0 Hz, 2H), 7.37 – 7.19 (m, 8H), 7.17 (dd, *J* = 6.5, 3.2 Hz, 1H), 3.73 (dd, *J* = 17.3, 9.6 Hz, 1H), 3.51 (dd, *J* = 17.4, 2.7 Hz, 1H).

^{13}C NMR (126 MHz, $CDCl_3$) δ 146.48, 140.71, 137.82, 133.48, 131.20, 129.04, 128.81, 128.75, 127.92, 126.53, 125.11, 125.07, 124.26, 120.04, 115.12, 110.51, 74.16, 35.97.

HRMS (ESI) m/z $[M + H]^+$ calculated for $C_{20}H_{16}N_4O_2S$: 377.1067, found 377.1065.

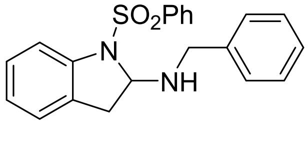

***N*-benzyl-1-(phenylsulfonyl)indolin-2-amine (74):**

^{1}H NMR (500 MHz, Chloroform-*d*) δ 7.68 – 7.47 (m, 3H), 7.42 (t, *J* = 7.6 Hz, 1H), 7.34 – 7.19 (m, 6H), 7.16 (tq, *J* = 6.3, 2.1, 1.6 Hz, 2H), 7.02 – 6.80 (m, 2H), 5.24 (dd, *J* = 8.6, 2.5 Hz, 1H), 3.86 (q, *J* = 13.3 Hz, 2H), 2.85 (dd, *J* = 17.0, 8.7 Hz, 1H), 2.67 (dd, *J* = 16.9, 2.5 Hz, 1H), 2.30 (s, 1H).

^{13}C NMR (126 MHz, $CDCl_3$) δ 140.56, 139.92, 138.67, 133.09, 131.31, 129.11, 128.48, 128.39, 128.03, 127.10, 126.91, 125.09, 124.80, 117.33, 78.49, 47.53, 35.21.

HRMS (ESI) m/z $[M + H]^+$ calculated for $C_{21}H_{20}N_2O_2S$: 365.1318, found 365.1317.

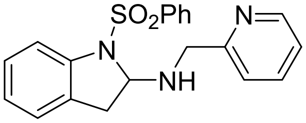

1-(phenylsulfonyl)-*N*-(pyridin-2-ylmethyl)indolin-2-amine (75):

^{1}H NMR (500 MHz, $CDCl_3$) δ 8.42 (dd, *J* = 5.0, 1.8 Hz, 1H), 7.64 – 7.47 (m, 4H), 7.39 (t, *J* = 7.5 Hz, 1H), 7.26 (t, *J* = 7.8 Hz, 2H), 7.18 (d, *J* = 7.8 Hz, 1H), 7.15 – 7.08 (m, 1H), 7.04 (dd, *J* = 7.6, 4.9 Hz, 1H), 6.95 (dt, *J* = 14.6, 7.3 Hz, 2H), 5.28 (dd, *J* = 8.5, 2.2 Hz, 1H), 4.33 – 3.69 (m, 2H), 2.82 (dd, *J* = 16.9, 8.4 Hz, 2H), 2.68 (dd, *J* = 17.0, 2.3 Hz, 1H).

^{13}C NMR (126 MHz, $CDCl_3$) δ 158.97, 149.23, 140.33, 138.61, 136.37, 132.96, 131.45, 128.98, 127.88, 126.78, 125.00, 124.80, 122.40, 121.89, 117.55, 78.33, 48.98, 35.33.

HRMS (ESI) m/z $[M + H]^+$ calculated for $C_{20}H_{19}N_3O_2S$: 366.1271, found 366.1270.

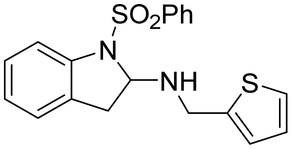

1-(phenylsulfonyl)-*N*-(thiophen-2-ylmethyl)indolin-2-amine (76):

^{1}H NMR (500 MHz, $CDCl_3$) δ 7.98 – 7.80 (m, 3H), 7.71 (t, *J* = 7.4 Hz, 1H), 7.58 (t, *J* = 7.7 Hz, 2H), 7.49 – 7.43 (m, 1H), 7.40 (dd, *J* = 5.1, 1.3 Hz, 1H), 7.25 (d, *J* = 6.9 Hz, 2H), 7.21 – 7.01 (m, 2H), 5.50 (dd, *J* = 8.6, 2.3 Hz, 1H), 4.41 (d, *J* = 14.1 Hz, 1H), 4.33 (d, *J* = 14.1 Hz, 1H), 3.11 (dd, *J* = 17.0, 8.6 Hz, 1H), 2.94 (dd, *J* = 17.0, 2.3 Hz, 1H), 2.68 (s, 1H).

^{13}C NMR (126 MHz, $CDCl_3$) δ 143.59, 140.40, 138.56, 133.14, 131.39, 129.13, 128.08, 126.91, 126.78, 125.25, 125.12, 124.97, 124.58, 117.65, 78.00, 42.88, 35.36.

HRMS (ESI) m/z $[M + H]^+$ calculated for $C_{19}H_{18}N_2O_2S_2$: 371.0882, found 371.0881.

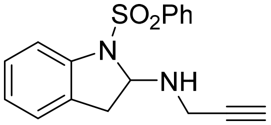

1-(phenylsulfonyl)-*N*-(prop-2-yn-1-yl)indolin-2-amine (77):

^{1}H NMR (500 MHz, $CDCl_3$) δ 7.85 (dd, *J* = 7.9, 2.2 Hz, 3H), 7.71 (t, *J* = 7.5 Hz, 1H), 7.58 (t, *J* = 7.7 Hz, 2H), 7.51 – 7.41 (m, 1H), 7.25 (d, *J* = 7.0 Hz, 2H), 5.42 (d, *J* = 8.3 Hz, 1H), 4.10 – 3.65 (m, 2H), 3.07 (dd, *J* = 17.0, 8.4 Hz, 1H), 2.90 (dd, *J* = 17.0, 2.2 Hz, 1H), 2.46 (s, 1H).

^{13}C NMR (126 MHz, $CDCl_3$) δ 140.27, 138.45, 133.23, 131.25, 129.17, 128.17, 126.93, 125.17, 125.13, 117.81, 86.19, 81.74, 71.35, 35.37, 33.46.

HRMS (ESI) m/z $[M + H]^+$ calculated for $C_{17}H_{16}N_2O_2S$: 313.1005, found 313.1003.

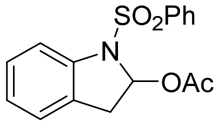

1-(phenylsulfonyl)indolin-2-yl acetate (78):

^{1}H NMR (500 MHz, $CDCl_3$) δ 7.91 (dd, *J* = 8.4, 1.3 Hz, 2H), 7.70 – 7.56 (m, 2H), 7.50 (t, *J* = 7.9 Hz, 2H), 7.35 – 7.23 (m, 1H), 7.18 (d, *J* = 7.4 Hz, 1H), 7.12 – 7.01 (m, 1H), 6.86 (dd, *J* = 7.0, 1.2 Hz, 1H), 3.34 (dd, *J* = 17.5, 7.0 Hz, 1H), 2.94 (d, *J* = 17.5 Hz, 1H), 2.02 (s, 3H).

^{13}C NMR (126 MHz, $CDCl_3$) δ 169.76, 140.62, 139.26, 133.55, 129.30, 128.53, 128.24, 127.17, 125.31, 124.29, 114.49, 85.81, 36.98, 21.11.

HRMS (ESI) m/z $[M + H]^+$ calculated for $C_{16}H_{15}NO_4S$: 318.0795, found 318.0794.

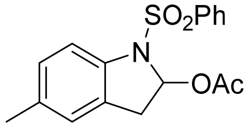

5-methyl-1-(phenylsulfonyl)indolin-2-yl acetate (79):

^{1}H NMR (500 MHz, $CDCl_3$) δ 7.66 (d, *J* = 7.9 Hz, 2H), 7.36 (d, *J* = 8.0 Hz, 1H), 7.26 (t, *J* = 7.5 Hz, 3H), 6.85 (d, *J* = 8.2 Hz, 1H), 6.76 (s, 1H), 6.59 (d, *J* = 6.8 Hz, 1H), 3.04 (dd, *J* = 17.5, 6.9 Hz, 1H), 2.65 (d, *J* = 17.5 Hz, 1H), 2.09 (s, 3H), 1.80 (s, 3H).

^{13}C NMR (126 MHz, $CDCl_3$) δ 169.81, 139.17, 138.26, 134.18, 133.46, 129.27, 128.73, 127.15, 126.82, 125.95, 114.45, 86.05, 36.96, 21.13, 21.02.

HRMS (ESI) m/z $[M + H]^+$ calculated for $C_{17}H_{17}NO_4S$: 332.0951, found 332.0949.

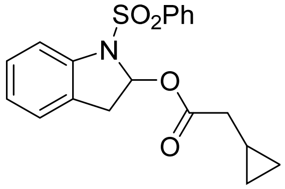

1-(phenylsulfonyl)indolin-2-yl 2-cyclopropylacetate (80):

^{1}H NMR (500 MHz, $CDCl_3$) δ 7.72 – 7.57 (m, 2H), 7.36 (td, *J* = 7.8, 4.9 Hz, 2H), 7.26 (t, *J* = 7.9 Hz, 2H), 7.04 (t, *J* = 7.7 Hz, 1H), 6.95 (d, *J* = 7.4 Hz, 1H), 6.84 (t, *J* = 7.5 Hz, 1H), 6.65 (dd, *J* = 7.0, 1.3 Hz, 1H), 3.13 (dd, *J* = 17.6, 7.1 Hz, 1H), 2.71 (d, *J* = 17.5 Hz, 1H), 1.95 (qd, *J* = 16.0, 7.1 Hz, 2H), 0.80 (tdd, *J* = 10.3, 9.1, 5.1 Hz, 1H), 0.33 (dd, *J* = 8.1, 1.6 Hz, 2H), -0.07 (dd, *J* = 8.8, 4.9 Hz, 2H).

^{13}C NMR (126 MHz, $CDCl_3$) δ 172.06, 140.65, 139.26, 133.53, 129.30, 128.57, 128.21, 127.17, 125.30, 124.26, 114.50, 85.69, 39.31, 37.09, 6.64, 4.47, 4.43.

HRMS (ESI) m/z $[M + H]^+$ calculated for $C_{19}H_{19}NO_4S$: 358.1108, found 358.1107.

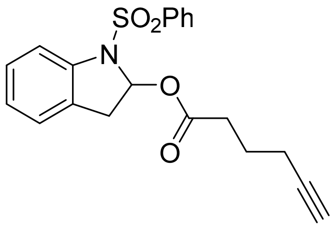

1-(phenylsulfonyl)indolin-2-yl hex-5-ynoate (81):

^{1}H NMR (500 MHz, $CDCl_3$) δ 8.24 – 7.97 (m, 2H), 7.87 – 7.74 (m, 2H), 7.69 (t, *J* = 7.7 Hz, 2H), 7.55 – 7.40 (m, 1H), 7.36 (d, *J* = 7.4 Hz, 1H), 7.26 (t, *J* = 7.5 Hz, 1H), 7.06 (d, *J* = 7.0 Hz, 1H), 3.53 (dd, *J* = 17.6, 7.1 Hz, 1H), 3.12 (d, *J* = 17.5 Hz, 1H), 2.60 (td, *J* = 7.4, 4.2 Hz, 2H), 2.46 (td, *J* = 6.9, 2.6 Hz, 2H), 2.18 (t, *J* = 2.7 Hz, 1H), 2.03 (p, *J* = 7.2 Hz, 2H).

^{13}C NMR (126 MHz, $CDCl_3$) δ 171.89, 140.58, 139.18, 133.58, 129.33, 128.46, 128.24, 127.15, 125.31, 124.28, 114.44, 85.69, 83.21, 69.43, 36.99, 32.92, 23.32, 17.86.

HRMS (ESI) m/z $[M + H]^+$ calculated for $C_{20}H_{19}NO_4S$: 370.1108, found 370.1106.

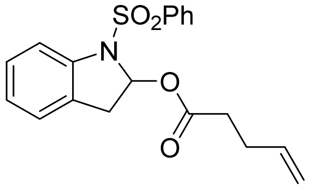

1-(phenylsulfonyl)indolin-2-yl pent-4-enoate (82):

^{1}H NMR (500 MHz, $CDCl_3$) δ 7.67 (d, *J* = 7.8 Hz, 2H), 7.36 (d, *J* = 7.7 Hz, 2H), 7.26 (t, *J* = 7.7 Hz, 2H), 7.12 – 6.99 (m, 1H), 6.94 (d, *J* = 7.4 Hz, 1H), 6.84 (t, *J* = 7.5 Hz, 1H), 6.63 (d, *J* = 6.9 Hz, 1H), 5.81 – 5.46 (m, 1H), 4.96 – 4.59 (m, 2H), 3.11 (dd, *J* = 17.5, 7.0 Hz, 1H), 2.68 (d, *J* = 17.5 Hz, 1H), 2.13 (t, *J* = 3.6 Hz, 4H).

^{13}C NMR (126 MHz, $CDCl_3$) δ 171.86, 140.61, 139.21, 136.39, 133.55, 129.31, 128.52, 128.23, 127.15, 125.30, 124.28, 115.89, 114.48, 85.76, 37.04, 33.52, 28.62.

HRMS (ESI) m/z $[M + H]^+$ calculated for $C_{19}H_{19}NO_4S$: 357.1035, found 357.1033.

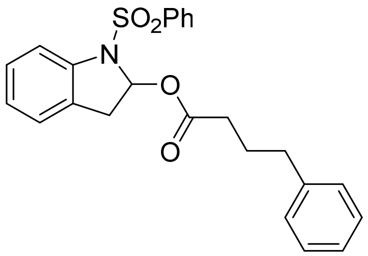

1-(phenylsulfonyl)indolin-2-yl 4-phenylbutanoate (83):

^{1}H NMR (500 MHz, $CDCl_3$) δ 8.05 – 7.86 (m, 2H), 7.77 – 7.58 (m, 2H), 7.52 (t, *J* = 7.9 Hz, 2H), 7.36 (dt, *J* = 14.5, 7.6 Hz, 3H), 7.32 – 7.27 (m, 1H), 7.25 (dd, *J* = 7.8, 2.3 Hz, 3H), 7.15 (t, *J* = 7.5 Hz, 1H), 6.95 (dd, *J* = 7.2, 1.2 Hz, 1H), 3.42 (dd, *J* = 17.5, 7.0 Hz, 1H), 2.98 (d, *J* = 17.5 Hz, 1H), 2.74 (t, *J* = 7.5 Hz, 2H), 2.34 (hept, *J* = 7.9, 7.4 Hz, 2H), 2.02 (p, *J* = 7.5 Hz, 2H).

^{13}C NMR (126 MHz, $CDCl_3$) δ 172.26, 141.31, 140.62, 139.24, 133.51, 129.28, 128.65, 128.54, 128.50, 128.23, 127.13, 126.14, 125.31, 124.26, 114.46, 85.67, 37.01, 35.09, 33.58, 26.27.

HRMS (ESI) m/z $[M + H]^+$ calculated for $C_{24}H_{23}NO_4S$: 422.1421, found 422.1420.

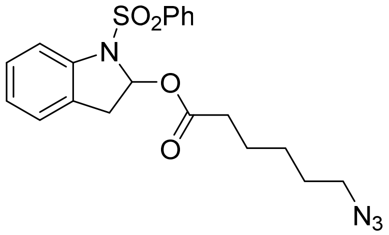

1-(phenylsulfonyl)indolin-2-yl 6-azidohexanoate (84):

^{1}H NMR (500 MHz, $CDCl_3$) δ 7.66 (dd, *J* = 7.7, 1.6 Hz, 2H), 7.45 – 7.33 (m, 2H), 7.26 (t, *J* = 7.8 Hz, 2H), 7.09 – 6.98 (m, 1H), 6.94 (d, *J* = 7.4 Hz, 1H), 6.84 (t, *J* = 7.5 Hz, 1H), 6.62 (dd, *J* = 7.2, 1.2 Hz, 1H), 3.11 (dd, *J* = 17.6, 7.1 Hz, 1H), 3.05 (t, *J* = 6.9 Hz, 2H), 2.68 (d, *J* = 17.5 Hz, 1H), 2.24 – 1.92 (m, 2H), 1.40 (dp, *J* = 20.0, 7.3 Hz, 4H), 1.29 – 1.13 (m, 2H).

^{13}C NMR (126 MHz, $CDCl_3$) δ 172.24, 140.59, 139.18, 133.56, 129.32, 128.49, 128.25, 127.13, 125.31, 124.31, 114.51, 85.64, 51.29, 37.01, 34.10, 28.60, 26.20, 24.22.

HRMS (ESI) m/z $[M + H]^+$ calculated for $C_{20}H_{22}N_4O_4S$: 415.1435, found 415.1433.

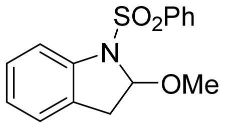

2-methoxy-1-(phenylsulfonyl)indoline (85):

^{1}H NMR (500 MHz, $CDCl_3$) δ 7.79 – 7.64 (m, 2H), 7.62 (d, *J* = 8.0 Hz, 1H), 7.55 – 7.43 (m, 1H), 7.37 (t, *J* = 7.9 Hz, 2H), 7.25 – 7.17 (m, 1H), 7.12 – 6.96 (m, 2H), 5.50 (dd, *J* = 4.9, 2.2 Hz, 1H), 3.51 (s, 3H), 2.90 – 2.64 (m, 2H).

^{13}C NMR (126 MHz, $CDCl_3$) δ 140.28, 138.91, 133.21, 131.62, 129.09, 127.87, 126.95, 125.20, 125.13, 117.69, 93.50, 55.22, 36.48.

HRMS (ESI) m/z $[M + H]^+$ calculated for $C_{15}H_{15}NO_3S$: 290.0845, found 290.0843.

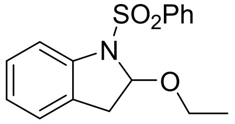

2-ethoxy-1-(phenylsulfonyl)indoline (86):

^1H NMR (500 MHz, $CDCl_3$) δ 7.56 (d, J = 7.9 Hz, 2H), 7.50 (d, J = 8.0 Hz, 1H), 7.39 (t, J = 7.5 Hz, 1H), 7.26 (t, J = 7.7 Hz, 2H), 7.12 (t, J = 7.7 Hz, 1H), 6.95 (dt, J = 14.8, 7.4 Hz, 2H), 5.50 (dd, J = 4.9, 2.5 Hz, 1H), 3.87 (dq, J = 10.0, 7.1 Hz, 1H), 3.58 (dq, J = 9.8, 6.9 Hz, 1H), 2.66 (d, J = 4.1 Hz, 2H), 1.09 (t, J = 7.0 Hz, 3H).

^{13}C NMR (126 MHz, $CDCl_3$) δ 140.33, 138.97, 133.15, 131.76, 129.05, 127.78, 126.93, 125.16, 125.09, 117.62, 92.19, 63.31, 36.71, 14.93.

HRMS (ESI) m/z $[M + H]^+$ calculated for $C_{16}H_{17}NO_3S$: 304.1002, found 304.1001.

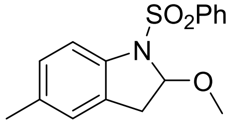

2-methoxy-5-methyl-1-(phenylsulfonyl)indoline (87):

^1H NMR (500 MHz, $CDCl_3$) δ 7.64 (dd, J = 8.1, 1.5 Hz, 2H), 7.50 (t, J = 7.3 Hz, 2H), 7.37 (t, J = 7.7 Hz, 2H), 7.03 (d, J = 8.2 Hz, 1H), 6.89 (s, 1H), 5.45 (dd, J = 5.4, 1.6 Hz, 1H), 3.51 (s, 3H), 3.04 – 2.53 (m, 2H), 2.27 (s, 3H).

^{13}C NMR (126 MHz, $CDCl_3$) δ 138.90, 137.86, 135.10, 133.13, 131.84, 129.08, 128.50, 126.97, 125.78, 117.71, 93.71, 55.24, 36.53, 21.16.

HRMS (ESI) m/z $[M + H]^+$ calculated for $C_{16}H_{17}NO_3S$: 304.1002, found 304.1002.

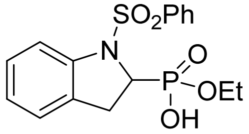

ethyl hydrogen (1-(phenylsulfonyl)indolin-2-yl)phosphonate (88):

^1H NMR (500 MHz, $CDCl_3$) δ 7.66 (d, J = 7.7 Hz, 2H), 7.60 (d, J = 8.1 Hz, 1H), 7.50 (t, J = 7.6 Hz, 1H), 7.36 (t, J = 7.8 Hz, 2H), 7.22 (t, J = 7.7 Hz, 1H), 7.06 (dt, J = 14.7, 7.4 Hz, 2H), 5.59 (dd, J = 4.8, 2.3 Hz, 1H), 4.25 – 3.83 (m, 1H), 3.68 (dq, J = 8.8, 7.0 Hz, 1H), 2.76 (d, J = 4.9 Hz, 2H), 1.19 (dd, J = 8.0, 6.5 Hz, 3H).

^{13}C NMR (126 MHz, $CDCl_3$) δ 140.32, 138.94, 133.16, 131.77, 129.06, 127.80, 126.93, 125.17, 125.12, 117.66, 92.20, 63.34, 36.72, 14.94.

HRMS (ESI) m/z $[M + H]^+$ calculated for $C_{16}H_{18}NO_5PS$: 368.0716, found 368.0715.

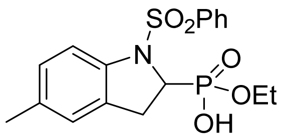

ethyl hydrogen (5-methyl-1-(phenylsulfonyl)indolin-2-yl)phosphonate (89):

^{1}H NMR (500 MHz, $CDCl_3$) δ 7.77 – 7.59 (m, 2H), 7.56 – 7.43 (m, 2H), 7.36 (t, *J* = 7.9 Hz, 2H), 7.02 (dd, *J* = 8.1, 1.8 Hz, 1H), 6.88 (s, 1H), 5.55 (dd, *J* = 5.2, 1.8 Hz, 1H), 3.97 (dq, *J* = 9.8, 7.1 Hz, 1H), 3.67 (dq, *J* = 9.7, 7.0 Hz, 1H), 2.84 – 2.59 (m, 2H), 2.27 (s, 3H), 1.19 (t, *J* = 7.0 Hz, 3H).

^{13}C NMR (126 MHz, $CDCl_3$) δ 138.95, 137.93, 134.97, 133.07, 132.00, 129.03, 128.40, 126.95, 125.81, 117.64, 92.39, 63.28, 36.72, 21.16, 14.94.

HRMS (ESI) m/z $[M + H]^+$ calculated for $C_{17}H_{20}NO_5PS$: 382.0873, found 382.0873.

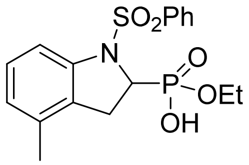

ethyl hydrogen (4-methyl-1-(phenylsulfonyl)indolin-2-yl)phosphonate (90):

^{1}H NMR (500 MHz, $CDCl_3$) δ 7.67 – 7.48 (m, 2H), 7.44 – 7.36 (m, 1H), 7.32 (d, *J* = 8.0 Hz, 1H), 7.26 (t, *J* = 7.9 Hz, 2H), 7.02 (t, *J* = 7.8 Hz, 1H), 6.75 (d, *J* = 7.6 Hz, 1H), 5.53 (dd, *J* = 6.1, 1.2 Hz, 1H), 3.87 (dq, *J* = 9.7, 7.1 Hz, 1H), 3.58 (dq, *J* = 9.7, 7.0 Hz, 1H), 2.80 – 2.44 (m, 2H), 2.01 (s, 3H), 1.10 (t, *J* = 7.0 Hz, 3H).
^{13}C NMR (126 MHz, $CDCl_3$) δ 139.98, 139.02, 134.62, 133.10, 130.36, 129.01, 127.83, 126.98, 126.06, 114.79, 92.00, 63.33, 35.60, 18.80, 14.97.
HRMS (ESI) m/z $[M + H]^+$ calculated for $C_{17}H_{20}NO_5PS$: 382.0873, found 382.0871.

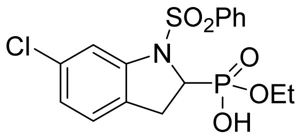

ethyl hydrogen (6-chloro-1-(phenylsulfonyl)indolin-2-yl)phosphonate (91):

^{1}H NMR (500 MHz, $CDCl_3$) δ 7.86 – 7.65 (m, 2H), 7.60 (d, *J* = 1.8 Hz, 1H), 7.53 (t, *J* = 7.5 Hz, 1H), 7.41 (t, *J* = 7.7 Hz, 2H), 7.07 – 6.82 (m, 2H), 5.60 (dd, *J* = 4.8, 2.5 Hz, 1H), 3.94 (dq, *J* = 9.7, 7.1 Hz, 1H), 3.65 (dq, *J* = 9.7, 7.0 Hz, 1H), 2.96 – 2.60 (m, 2H), 1.18 (t, *J* = 7.0 Hz, 3H).

^{13}C NMR (126 MHz, $CDCl_3$) δ 141.61, 138.89, 133.50, 133.43, 130.21, 129.26, 126.96, 125.90, 125.12, 117.83, 92.69, 63.48, 36.33, 14.92.

HRMS (ESI) m/z $[M + H]^+$ calculated for $C_{16}H_{17}ClNO_5PS$: 402.0326, found 402.0325.

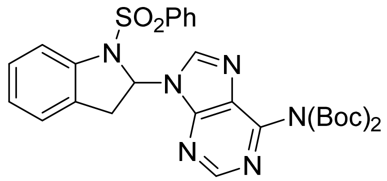

***tert*-butyl (*tert*-butoxycarbonyl)(9-(1-(phenylsulfonyl)indolin-2-yl)-9*H*-purin-6-yl)carbamate (92):**

^{1}H NMR (500 MHz, $CDCl_3$) δ 8.81 (s, 1H), 8.08 (s, 1H), 7.74 (d, *J* = 8.1 Hz, 1H), 7.71 – 7.65 (m, 2H), 7.55 (t, *J* = 7.5 Hz, 1H), 7.40 (t, *J* = 7.8 Hz, 2H), 7.34 (td, *J* = 7.7, 1.8 Hz, 1H), 7.23 – 7.06 (m, 2H), 6.83 (dd, *J* = 8.9, 2.5 Hz, 1H), 3.53 (dd, *J* = 17.3, 9.0 Hz, 1H), 3.28 (dd, *J* = 17.3, 2.5 Hz, 1H), 1.46 (s, 20H).

^{13}C NMR (126 MHz, $CDCl_3$) δ 152.39, 152.33, 150.63, 150.60, 142.39, 140.46, 137.51, 134.07, 129.54, 128.99, 128.92, 128.33, 126.97, 125.57, 125.54, 115.76, 83.98, 70.80, 37.25, 27.93.

HRMS (ESI) m/z $[M + H]^+$ calculated for $C_{29}H_{32}N_6O_6S$: 593.2177, found 593.2176.

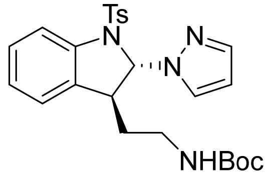

***tert*-butyl (2-(-2-(1*H*-pyrazol-1-yl)-1-tosylindolin-3-yl)ethyl)carbamate (93):**

^{1}H NMR (500 MHz, $CDCl_3$) δ 7.63 (dd, *J* = 19.8, 8.0 Hz, 3H), 7.53 (d, *J* = 1.7 Hz, 1H), 7.45 – 7.35 (m, 2H), 7.32 – 7.24 (m, 3H), 7.21 (t, *J* = 7.5 Hz, 1H), 6.86 (d, *J* = 7.9 Hz, 1H), 6.26 (d, *J* = 2.2 Hz, 1H), 4.90 (s, 0H), 3.73 (p, *J* = 4.4 Hz, 1H), 3.30 (dt, *J* = 14.5, 7.2 Hz, 1H), 3.19 (dt, *J* = 13.8, 6.5 Hz, 1H), 2.48 (s, 3H), 1.60 (s, 9H), 1.27 (ddt, *J* = 15.7, 11.8, 6.1 Hz, 1H).

^{13}C NMR (126 MHz, $CDCl_3$) δ 156.26, 144.50 (d, *J* = 78.9 Hz), 140.78 (d, *J* = 9.7 Hz), 139.86, 135.96, 132.61, 129.71, 129.16, 128.42, 126.95, 124.32, 123.69, 113.83, 106.10, 79.12 (d, *J* = 87.2 Hz), 60.49, 43.95, 38.89, 28.53, 27.56, 21.61.

HRMS (ESI) m/z $[M + H]^+$ calculated for $C_{29}H_{32}N_6O_6S$: 593.2177, found 593.2176.

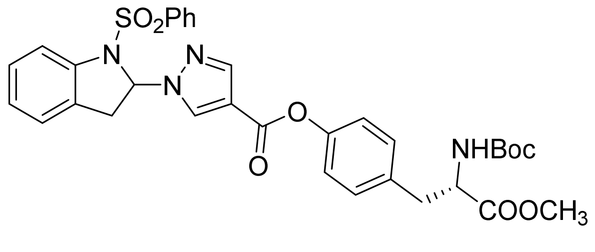

4-((*S*)-2-((*tert*-butoxycarbonyl)amino)-3-methoxy-3-oxopropyl)phenyl 1-(1-(phenylsulfonyl)indolin-2-yl)-1*H*-pyrazole-4-carboxylate (94):

^{1}H NMR (500 MHz, $CDCl_3$) δ 8.47 (s, 1H), 8.15 (s, 1H), 7.81 (dd, *J* = 22.5, 8.0 Hz, 3H), 7.72 (t, *J* = 7.4 Hz, 1H), 7.62 – 7.53 (m, 2H), 7.52 – 7.41 (m, 1H), 7.40 – 7.17 (m, 6H), 6.90 – 6.62 (m, 1H), 5.19 (d, *J* = 8.3 Hz, 1H), 4.75 (d, *J* = 7.2 Hz, 1H), 3.88 (d, *J* = 1.8 Hz, 3H), 3.67 (d, *J* = 17.0 Hz, 1H), 3.58 (dd, *J* = 17.2, 8.7 Hz, 1H), 3.26 (dq, *J* = 14.1, 8.0 Hz, 2H), 1.59 (s, 9H).

^{13}C NMR (126 MHz, $CDCl_3$) δ 172.33, 160.91, 155.19, 149.50, 142.58, 140.27, 138.07, 133.82, 133.76, 132.88, 130.40, 129.40, 128.92, 128.61, 126.87, 125.37, 125.29, 121.86, 115.62, 114.68, 80.12, 54.46, 52.39, 37.80, 36.14, 28.40.

HRMS (ESI) m/z $[M + H]^+$ calculated for $C_{33}H_{34}N_4O_8S$: 647.2170, found 647.2168.

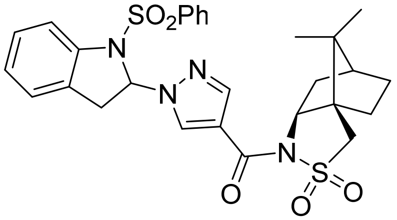

((3a*R*,6*S*,7a*S*)-8,8-dimethyl-2,2-dioxidotetrahydro-3*H*-3a,6-methanobenzo[c]isothiazol-1(4*H*)-yl)(1-(1-(phenylsulfonyl)indolin-2-yl)-1*H*-pyrazol-4-yl)methanone (95):

^{1}H NMR (500 MHz, $CDCl_3$) δ 8.30 (d, *J* = 13.7 Hz, 1H), 7.85 (d, *J* = 23.3 Hz, 1H), 7.55 – 7.49 (m, 1H), 7.49 – 7.44 (m, 1H), 7.39 (dt, *J* = 21.7, 7.9 Hz, 2H), 7.27 (dt, *J* = 11.2, 7.8 Hz, 2H), 7.18 – 7.12 (m, 1H), 7.07 (t, *J* = 6.9 Hz, 1H), 6.96 (td, *J* = 7.5, 3.5 Hz, 1H), 6.44 (ddd, *J* = 13.8, 8.7, 2.4 Hz, 1H), 4.02 (ddd, *J* = 7.5, 4.6, 2.6 Hz, 1H), 3.63 – 3.13 (m, 4H), 2.10 – 1.73 (m, 5H), 1.38 – 1.31 (m, 1H), 1.28 (q, *J* = 3.8, 2.8 Hz, 1H), 1.15 (d, *J* = 21.1 Hz, 3H), 0.89 (d, *J* = 7.7 Hz, 3H).

^{13}C NMR (126 MHz, $CDCl_3$) δ 161.38 (d, *J* = 10.7 Hz), 142.85 (d, *J* = 26.1 Hz), 140.38 (d, *J* = 15.4 Hz), 138.42 (d, *J* = 4.6 Hz), 133.59 (d, *J* = 53.8 Hz), 133.51, 129.22 (d, *J* = 8.3 Hz), 128.73 (d, *J* = 45.0 Hz), 128.24 (d, *J* = 5.8 Hz), 126.79 (d, *J* = 10.6 Hz), 125.00 (d, *J* = 8.2 Hz), 124.66 (d, *J* = 19.4 Hz), 117.06 (d, *J* = 2.4 Hz), 114.61 (d, *J* = 28.3 Hz), 75.81 (d, *J* = 11.9 Hz), 65.95 (d, *J* = 15.3 Hz), 53.56 (d, *J* = 12.7 Hz), 48.14 (d, *J* = 3.6 Hz), 47.81, 45.01 (d, *J* = 4.6 Hz), 38.47 (d, *J* = 5.3 Hz), 35.93 (d, *J* = 44.7 Hz), 33.08 (d, *J* = 5.7 Hz), 26.53, 21.14 (d, *J* = 17.1 Hz), 19.92 (d, *J* = 2.3 Hz).

HRMS (ESI) m/z $[M + H]^+$ calculated for $C_{28}H_{30}N_4O_5S_2$: 567.1730, found 567.1728.

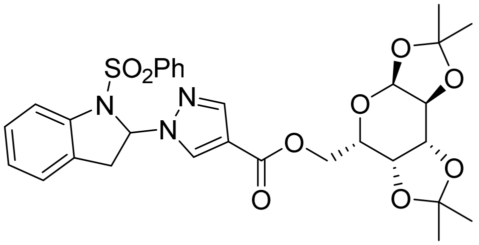

((3a*S*,5*S*,5a*R*,8a*R*,8b*S*)-2,2,7,7-tetramethyltetrahydro-5*H*-bis([1,3]dioxolo)[4,5-*b*:4',5'-*d*]pyran-5-yl)methyl 1-(1-(phenylsulfonyl)indolin-2-yl)-1*H*-pyrazole-4-carboxylate (96):

^{1}H NMR (500 MHz, CDCl$_3$) δ 8.00 (t, *J* = 2.5 Hz, 1H), 7.71 (d, *J* = 5.6 Hz, 1H), 7.61 – 7.44 (m, 3H), 7.40 (t, *J* = 7.4 Hz, 1H), 7.31 – 7.22 (m, 2H), 7.20 – 7.11 (m, 1H), 7.04 (d, *J* = 7.6 Hz, 1H), 6.96 (t, *J* = 7.6 Hz, 1H), 6.35 (dt, *J* = 5.6, 2.5 Hz, 1H), 5.42 (d, *J* = 4.8 Hz, 1H), 4.51 (dd, *J* = 7.8, 2.4 Hz, 1H), 4.42 – 4.12 (m, 4H), 4.01 (dd, *J* = 5.1, 2.6 Hz, 1H), 3.30 (d, *J* = 5.7 Hz, 2H), 1.38 (d, *J* = 8.3 Hz, 3H), 1.34 (s, 3H), 1.22 (s, 3H), 1.20 (d, *J* = 4.7 Hz, 3H).

^{13}C NMR (126 MHz, CDCl$_3$) δ 162.49, 142.22 (d, *J* = 2.7 Hz), 139.31 (d, *J* = 270.3 Hz), 133.71, 132.43 (d, *J* = 20.1 Hz), 129.34, 128.68 (dd, *J* = 42.1, 2.7 Hz), 126.81 (d, *J* = 3.0 Hz), 125.20 (d, *J* = 3.8 Hz), 125.18 (d, *J* = 18.0 Hz), 115.39, 115.22 (d, *J* = 11.5 Hz), 109.36 (d, *J* = 103.6 Hz), 96.40, 76.13 (d, *J* = 7.2 Hz), 71.16, 70.71 (d, *J* = 21.5 Hz), 66.18 (d, *J* = 8.4 Hz), 63.43, 36.74, 36.16 (d, *J* = 6.3 Hz), 26.17 (d, *J* = 1.9 Hz), 26.08, 25.11, 24.58.

HRMS (ESI) m/z $[M + H]^+$ calculated for $C_{30}H_{33}N_3O_9S$: 612.2010, found 612.2009.

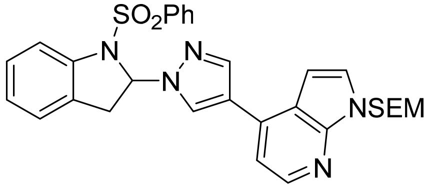

4-(1-(1-(phenylsulfonyl)indolin-2-yl)-1*H*-pyrazol-4-yl)-1-((2-(trimethylsilyl)ethoxy)methyl)-1*H*-pyrrolo[2,3-*b*]pyridine (97):

^{1}H NMR (500 MHz, CDCl$_3$) δ 8.22 (d, *J* = 4.9 Hz, 1H), 8.06 (s, 1H), 7.83 (s, 1H), 7.56 (d, *J* = 7.9 Hz, 2H), 7.49 (d, *J* = 8.1 Hz, 1H), 7.39 (t, *J* = 7.4 Hz, 1H), 7.31 (d, *J* = 3.7 Hz, 1H), 7.26 (t, *J* = 7.6 Hz, 2H), 7.22 – 7.12 (m, 3H), 7.03 (t, *J* = 7.3 Hz, 2H), 6.66 – 6.48 (m, 2H), 5.62 (s, 2H), 3.55 (d, *J* = 17.0 Hz, 1H), 3.50 – 3.34 (m, 3H), 0.84 (t, *J* = 8.2 Hz, 2H), -0.14 (d, *J* = 1.7 Hz, 9H).

^{13}C NMR (126 MHz, CDCl$_3$) δ 148.99, 143.60, 140.52, 139.63, 138.63, 137.23, 136.27, 133.57, 132.73, 129.26, 128.45, 128.10, 126.83, 125.22, 124.96, 120.99, 117.59, 115.07, 113.91, 100.51, 76.01, 73.13, 66.33, 35.92, 17.90, -1.30.

HRMS (ESI) m/z $[M + H]^+$ calculated for $C_{30}H_{33}N_5O_3SSi$: 572.2146, found 572.2144.

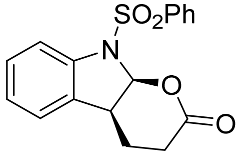

***trans*-9-(phenylsulfonyl)-4,4a,9,9a-tetrahydropyrano[2,3-*b*]indol-2(3*H*)-one (99):**

^{1}H NMR (500 MHz, CDCl$_3$) δ 8.27 – 7.85 (m, 2H), 7.59 – 7.50 (m, 1H), 7.47 (dd, *J* = 8.6, 6.9 Hz, 2H), 7.27 (d, *J* = 8.1 Hz, 1H), 7.19 (q, *J* = 7.9, 7.4 Hz, 1H), 7.08 (d, *J* = 7.5 Hz, 1H), 7.02 (t, *J* = 7.5 Hz, 1H), 6.54 (d, *J* = 7.5 Hz, 1H), 3.72 (d, *J* = 6.0 Hz, 1H), 2.63 – 2.24 (m, 2H), 2.21 – 2.07 (m, 2H).

^{13}C NMR (126 MHz, CDCl$_3$) δ 168.99, 140.68, 139.04, 133.74, 129.54, 129.31, 129.11, 127.96, 124.39, 123.96, 113.77, 91.55, 38.18, 26.97, 21.06.

HRMS (ESI) m/z $[M + H]^+$ calculated for $C_{17}H_{15}NO_4S$: 330.0795 found 330.0793.

5. Spectral Data

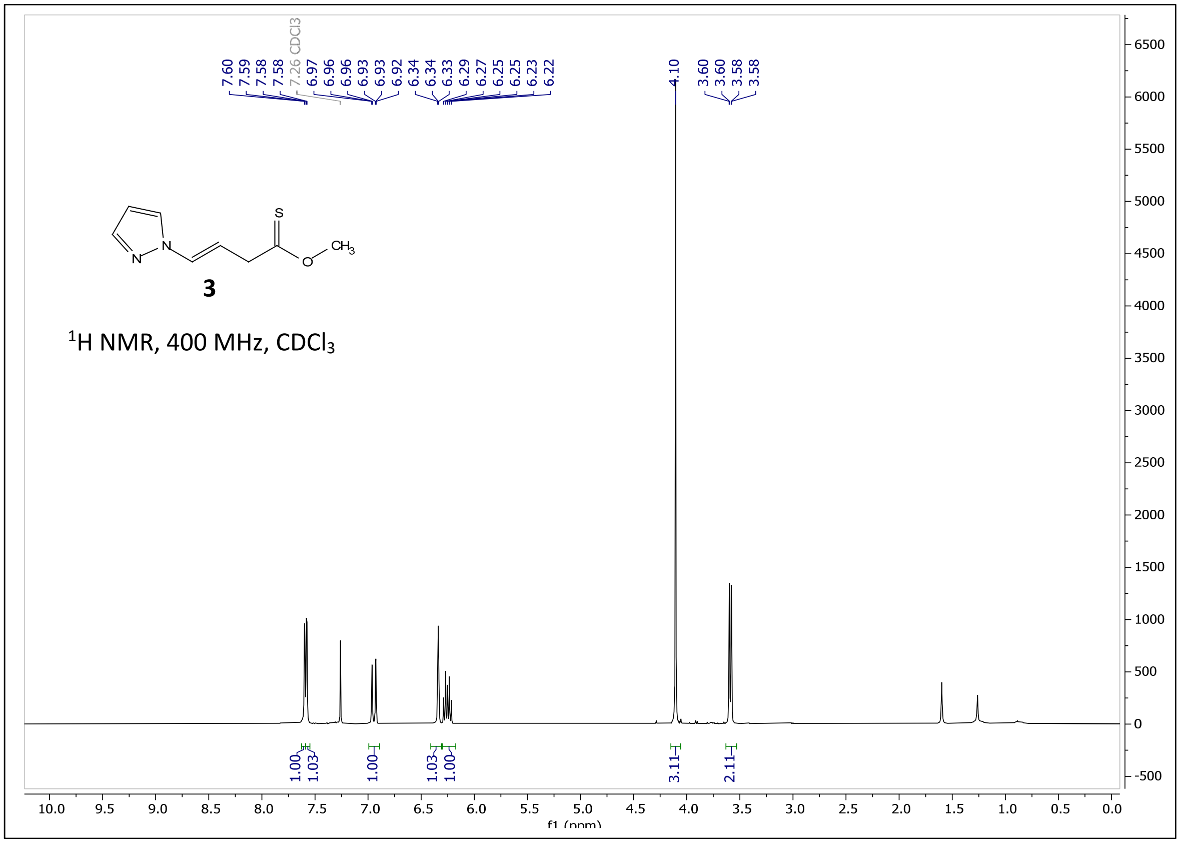

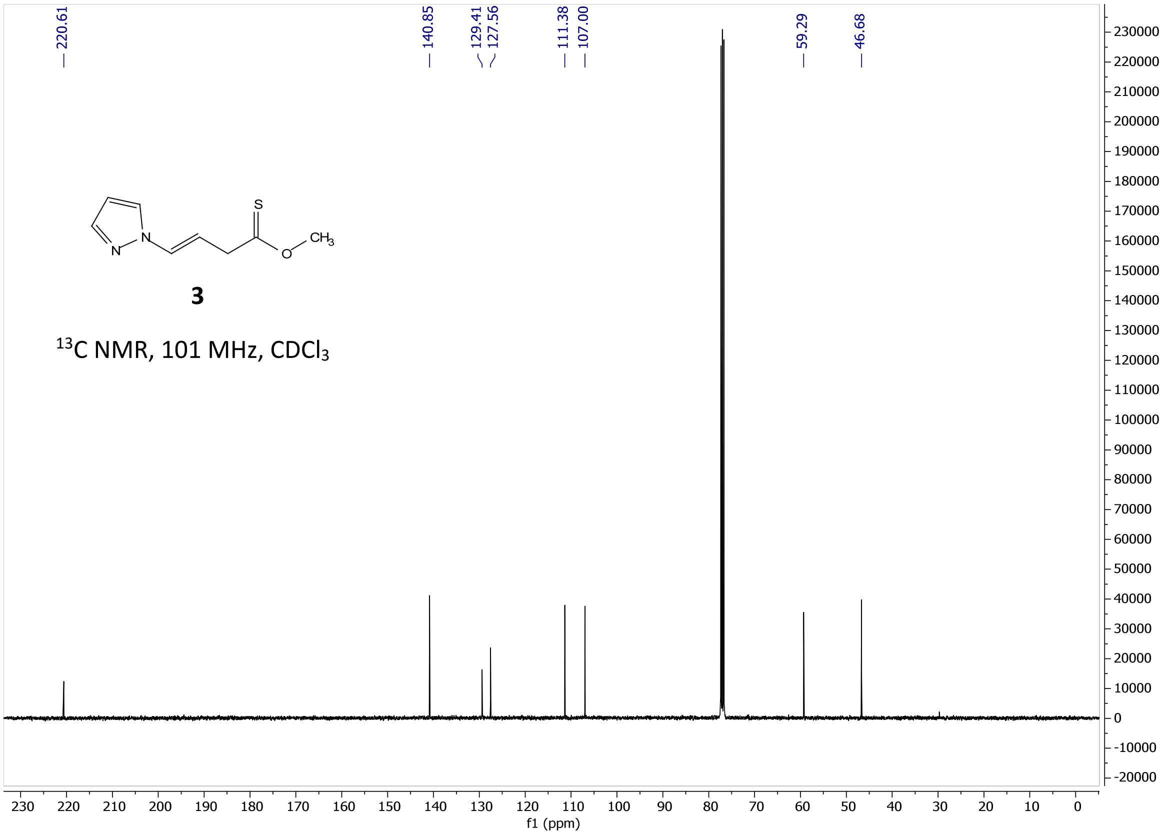
220.61
140.85
129.41
127.56
111.38
107.00
59.29
46.68
3
^{13}C NMR, 101 MHz, $CDCl_3$
f1 (ppm)

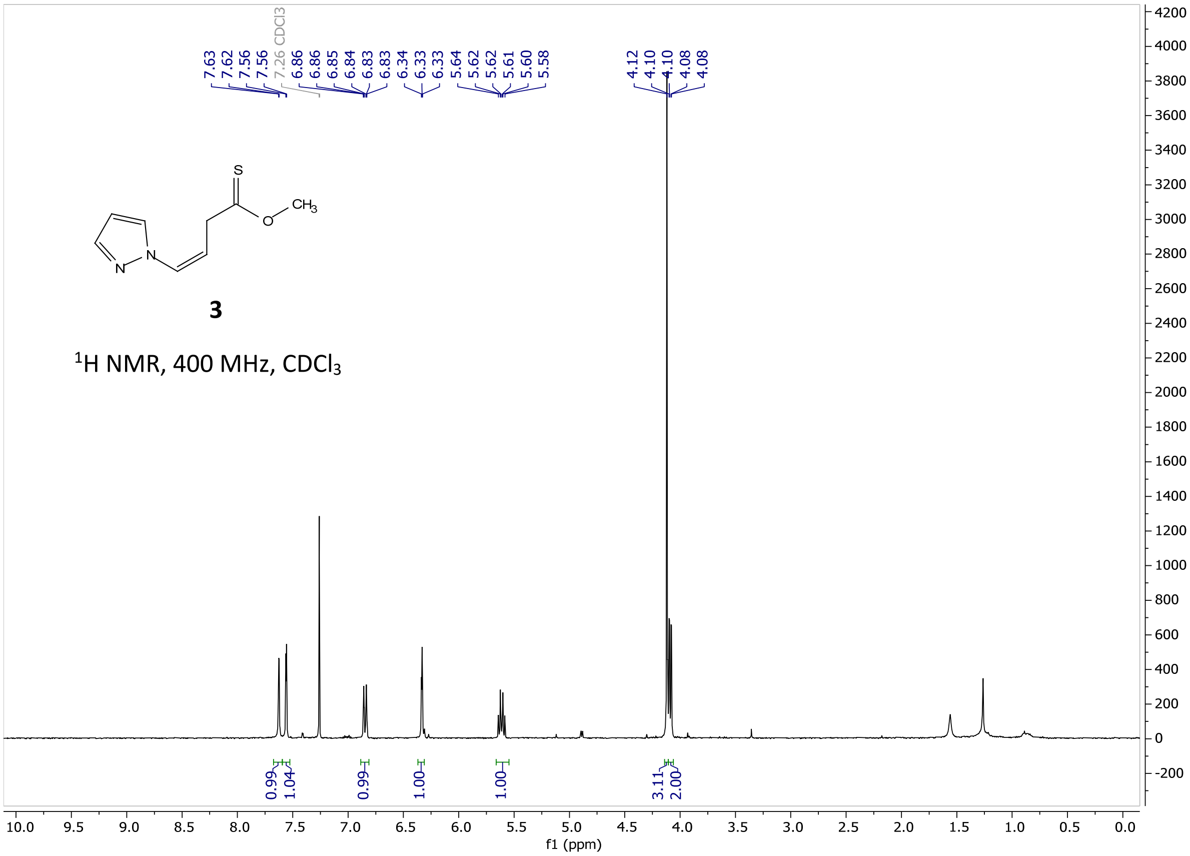
7.63
7.62
7.56
7.56
7.26 CDCl3
6.86
6.86
6.85
6.84
6.83
6.83
6.34
6.33
6.33
5.64
5.62
5.62
5.61
5.60
5.58
4.12
4.10
4.10
4.08
4.08
3
^{1}H NMR, 400 MHz, $CDCl_3$
0.99
1.04
0.99
1.00
1.00
3.11
2.00
f1 (ppm)

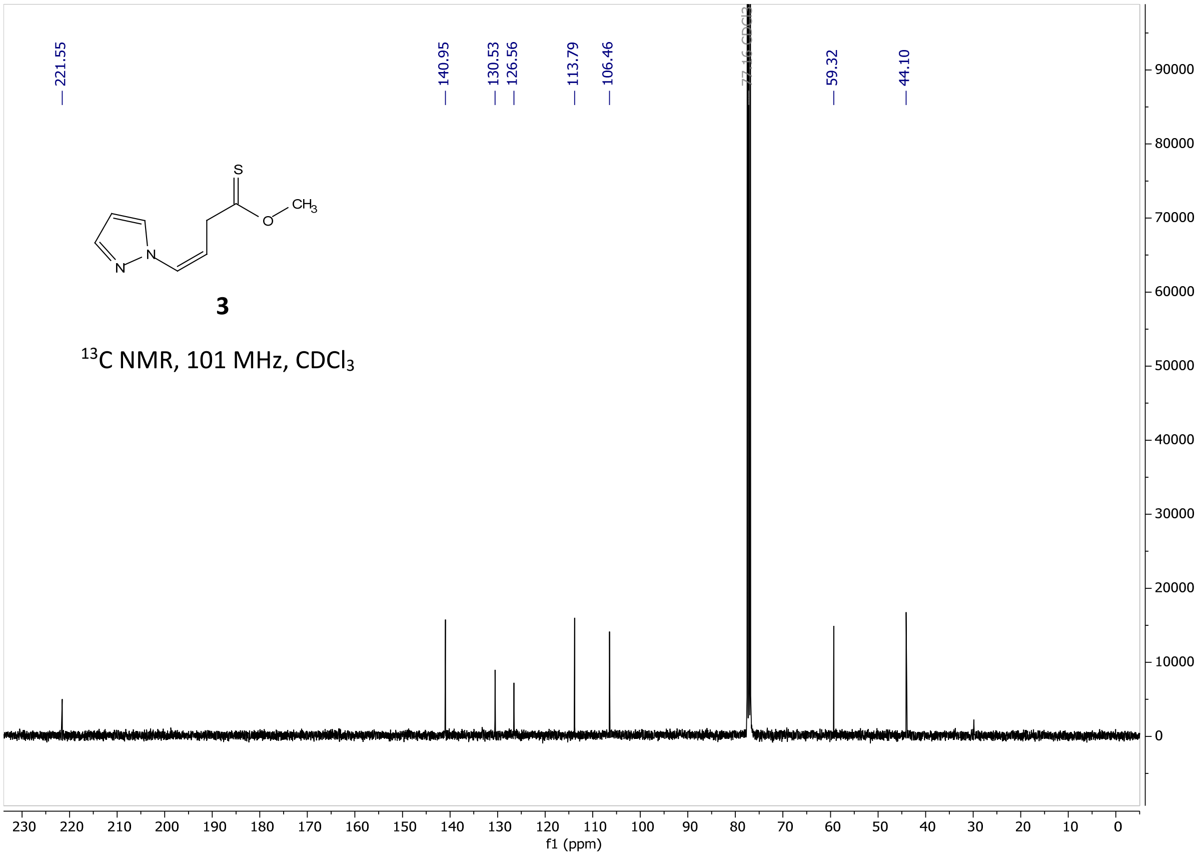

221.55
140.95
130.53
126.56
113.79
106.46
77.16 CDCl3
59.32
44.10
3
13C NMR, 101 MHz, CDCl3
f1 (ppm)

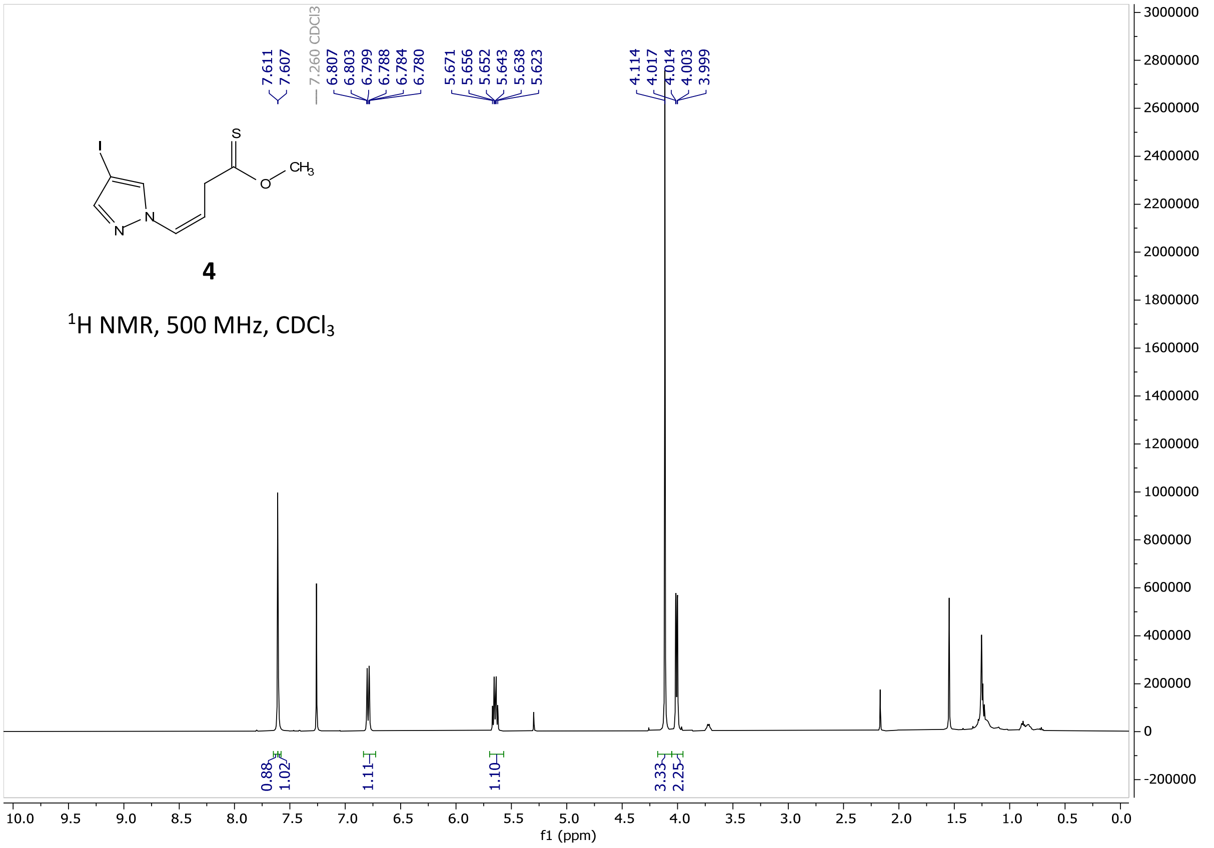

7.611
7.607
7.260 CDCl3
6.807
6.803
6.799
6.788
6.784
6.780
5.671
5.656
5.652
5.643
5.638
5.623
4.114
4.017
4.014
4.003
3.999
4
1H NMR, 500 MHz, CDCl3
0.88
1.02
1.11
1.10
3.33
2.25
f1 (ppm)

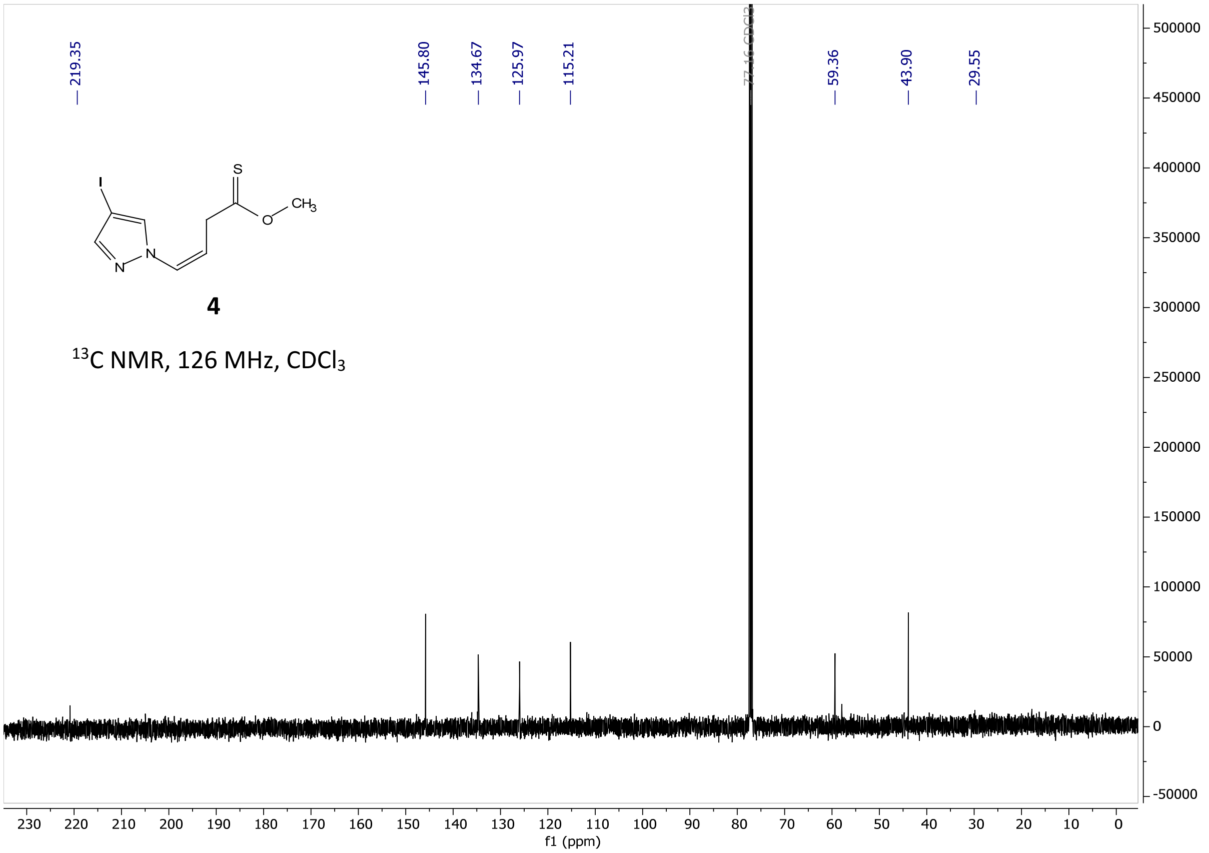
219.35
145.80
134.67
125.97
115.21
77.16 CDCl3
59.36
43.90
29.55
I
S
CH3
O
N
N
4
13C NMR, 126 MHz, CDCl3
f1 (ppm)

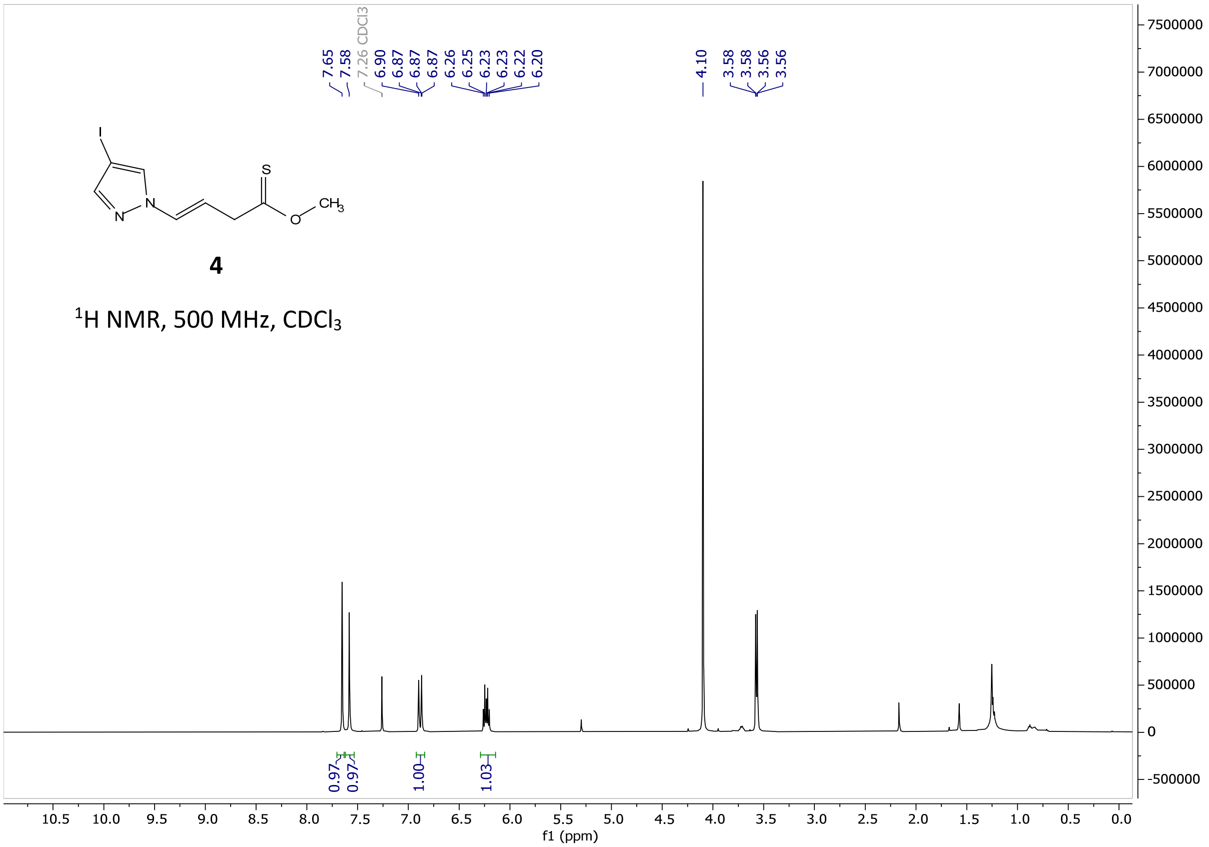
7.65
7.58
7.26 CDCl3
6.90
6.87
6.87
6.87
6.26
6.25
6.23
6.23
6.22
6.20
4.10
3.58
3.58
3.56
3.56
I
S
CH3
O
N
N
4
1H NMR, 500 MHz, CDCl3
0.97
0.97
1.00
1.03
f1 (ppm)

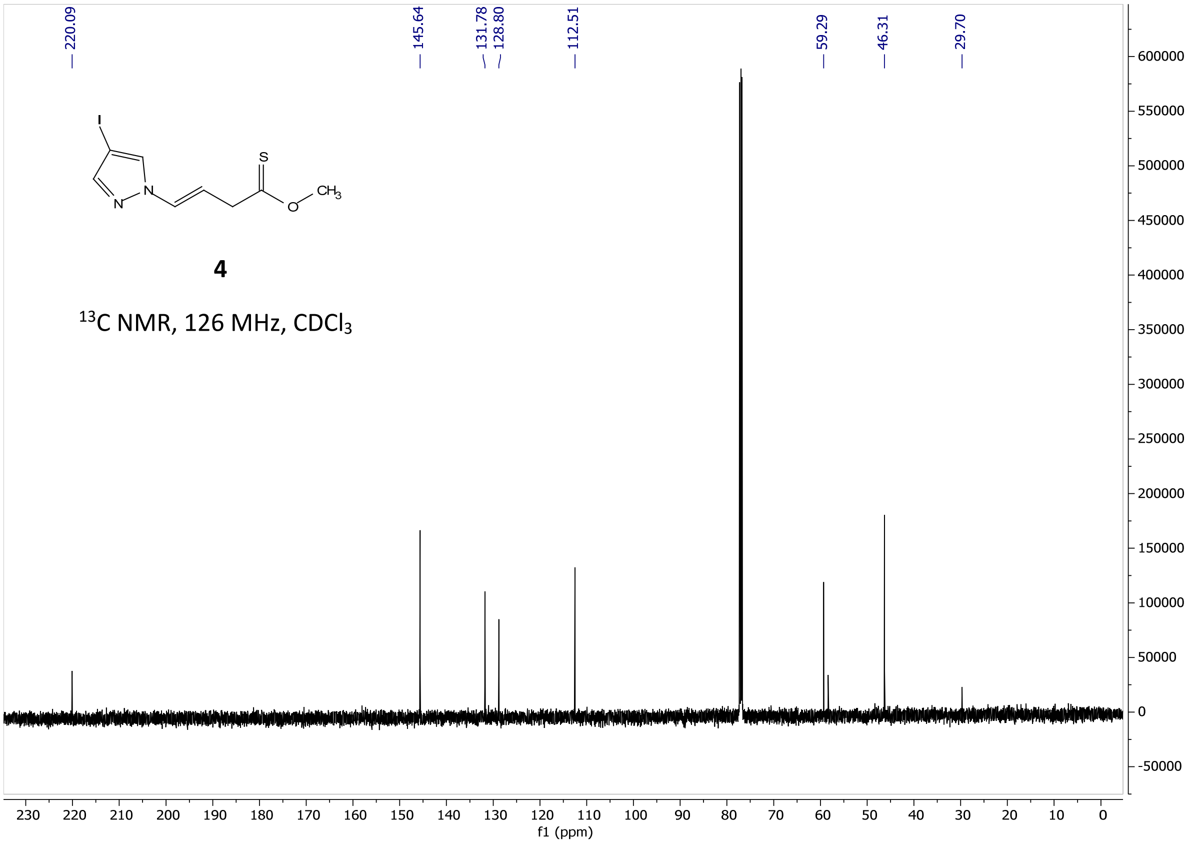
220.09
145.64
131.78
128.80
112.51
59.29
46.31
29.70
4
13C NMR, 126 MHz, CDCl3
f1 (ppm)

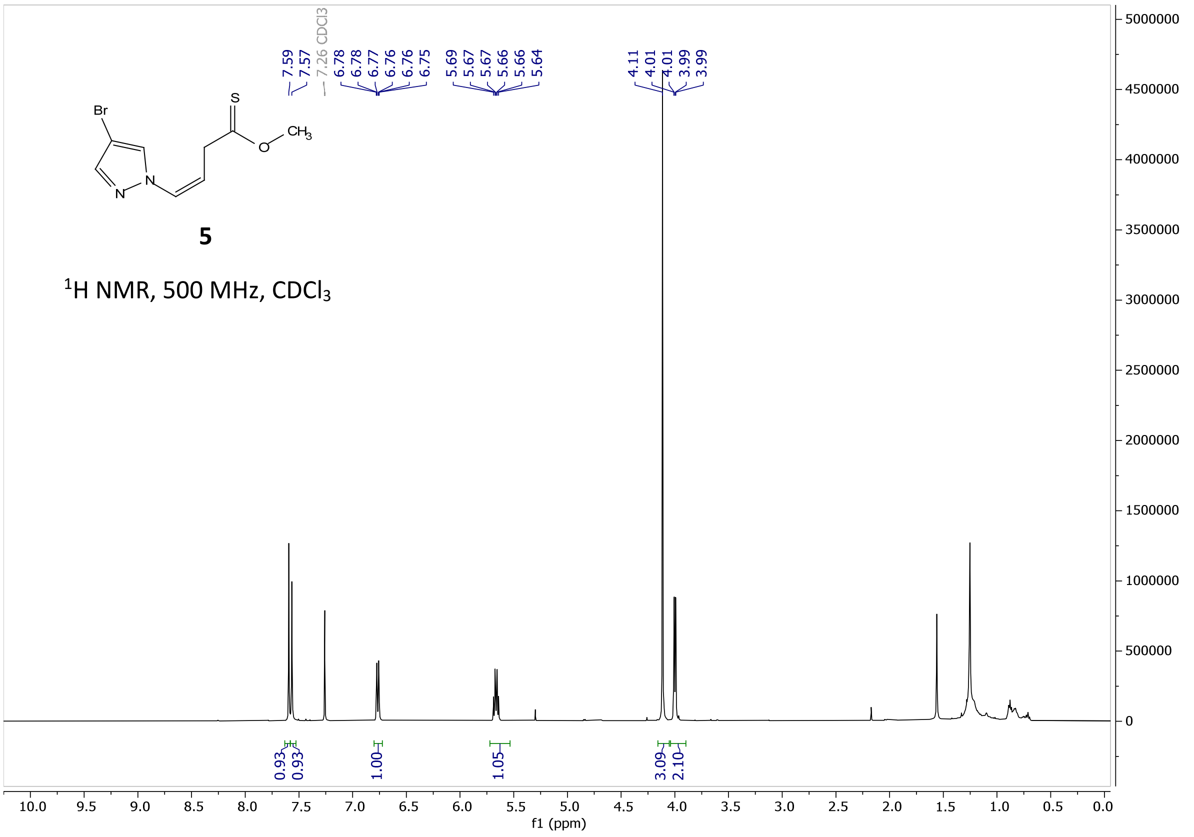
7.59
7.57
7.26 CDCl3
6.78
6.78
6.77
6.76
6.76
6.75
5.69
5.67
5.67
5.66
5.66
5.64
4.11
4.01
4.01
3.99
3.99
Br
5
1H NMR, 500 MHz, CDCl3
0.93
0.93
1.00
1.05
3.09
2.10
f1 (ppm)

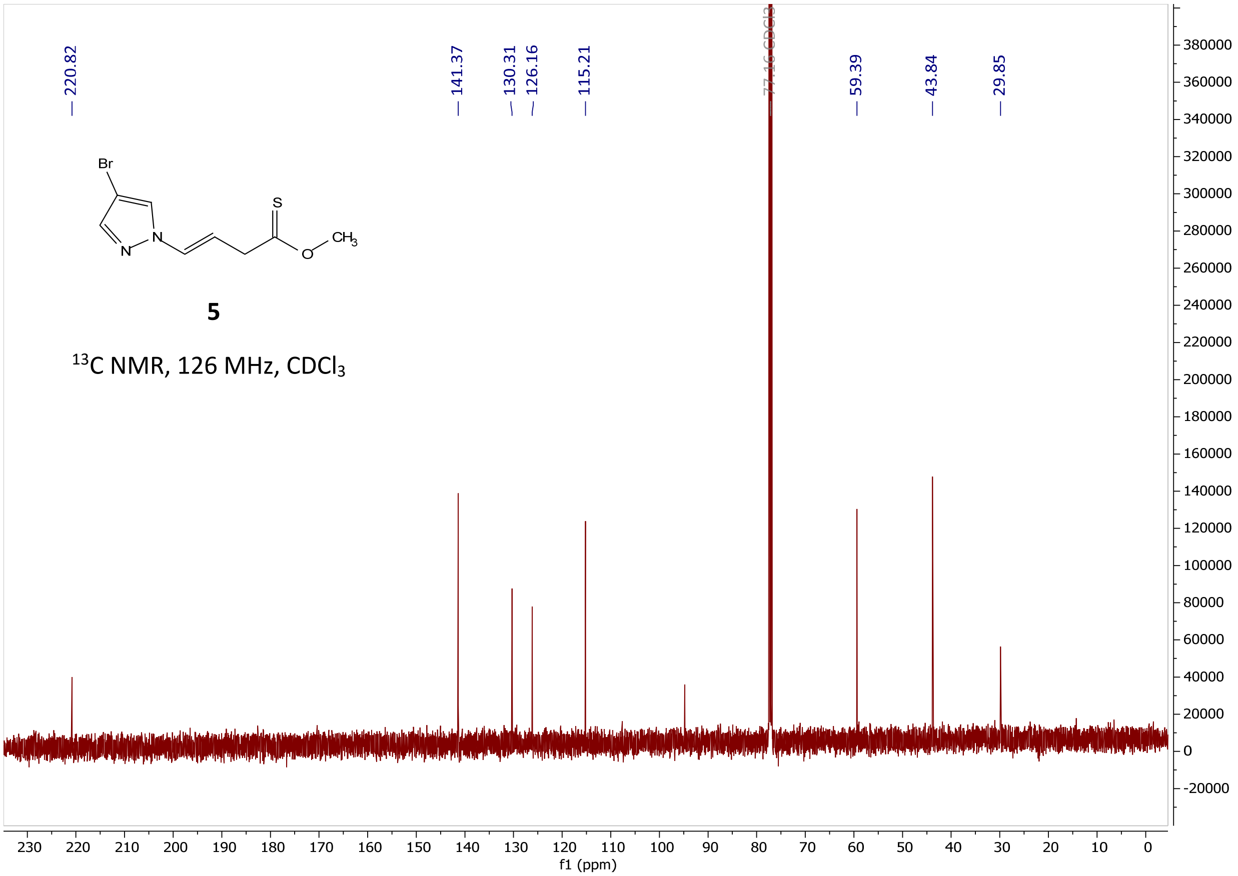
220.82
141.37
130.31
126.16
115.21
77.16 CDCl3
59.39
43.84
29.85
Br
N
N
S
O
CH3
5
13C NMR, 126 MHz, CDCl3
380000
360000
340000
320000
300000
280000
260000
240000
220000
200000
180000
160000
140000
120000
100000
80000
60000
40000
20000
0
-20000
230 220 210 200 190 180 170 160 150 140 130 120 110 100 90 80 70 60 50 40 30 20 10 0
f1 (ppm)

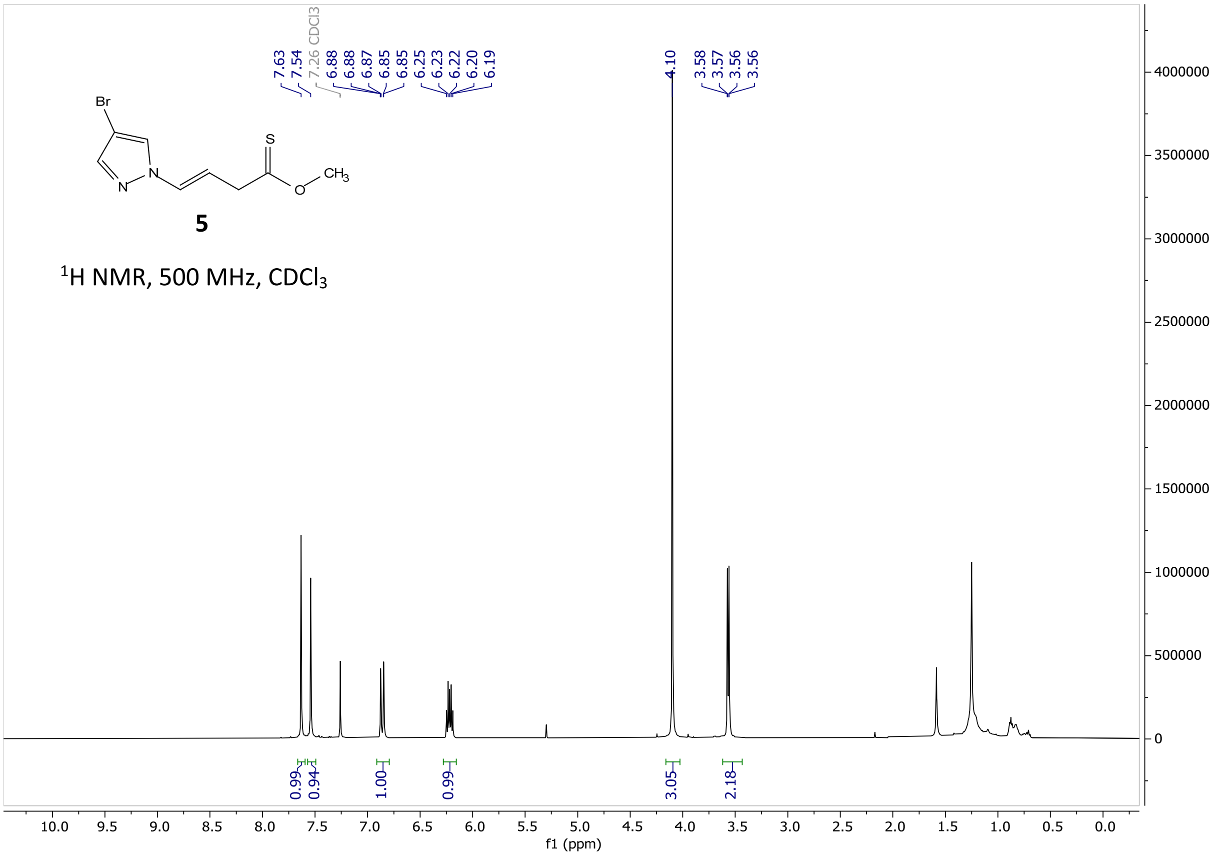
7.63
7.54
7.26 CDCl3
6.88
6.88
6.87
6.85
6.85
6.25
6.23
6.22
6.20
6.19
4.10
3.58
3.57
3.56
3.56
Br
S
N
N
CH3
O
5
1H NMR, 500 MHz, CDCl3
0.99
0.94
1.00
0.99
3.05
2.18
f1 (ppm)

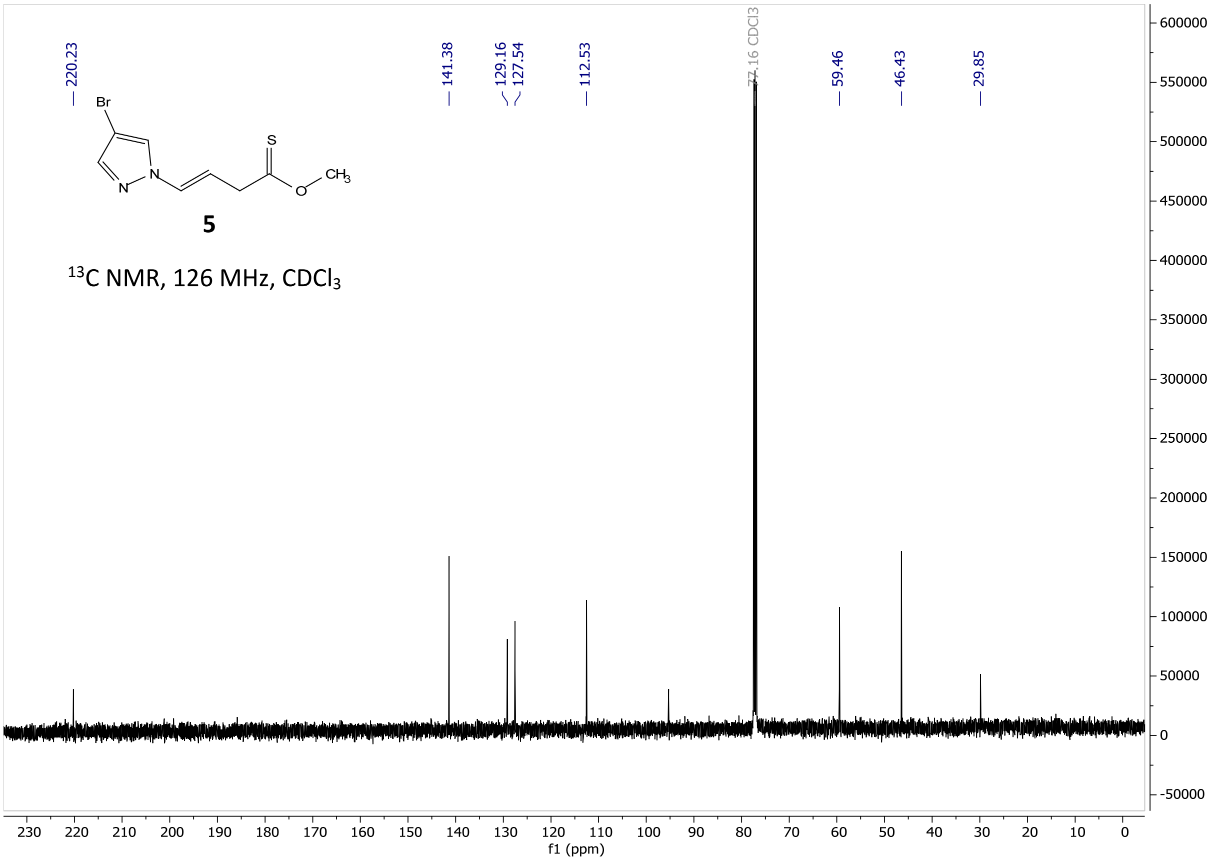
220.23
141.38
129.16
127.54
112.53
77.16 CDCl3
59.46
46.43
29.85
Br
S
N
N
CH3
O
5
13C NMR, 126 MHz, CDCl3
f1 (ppm)

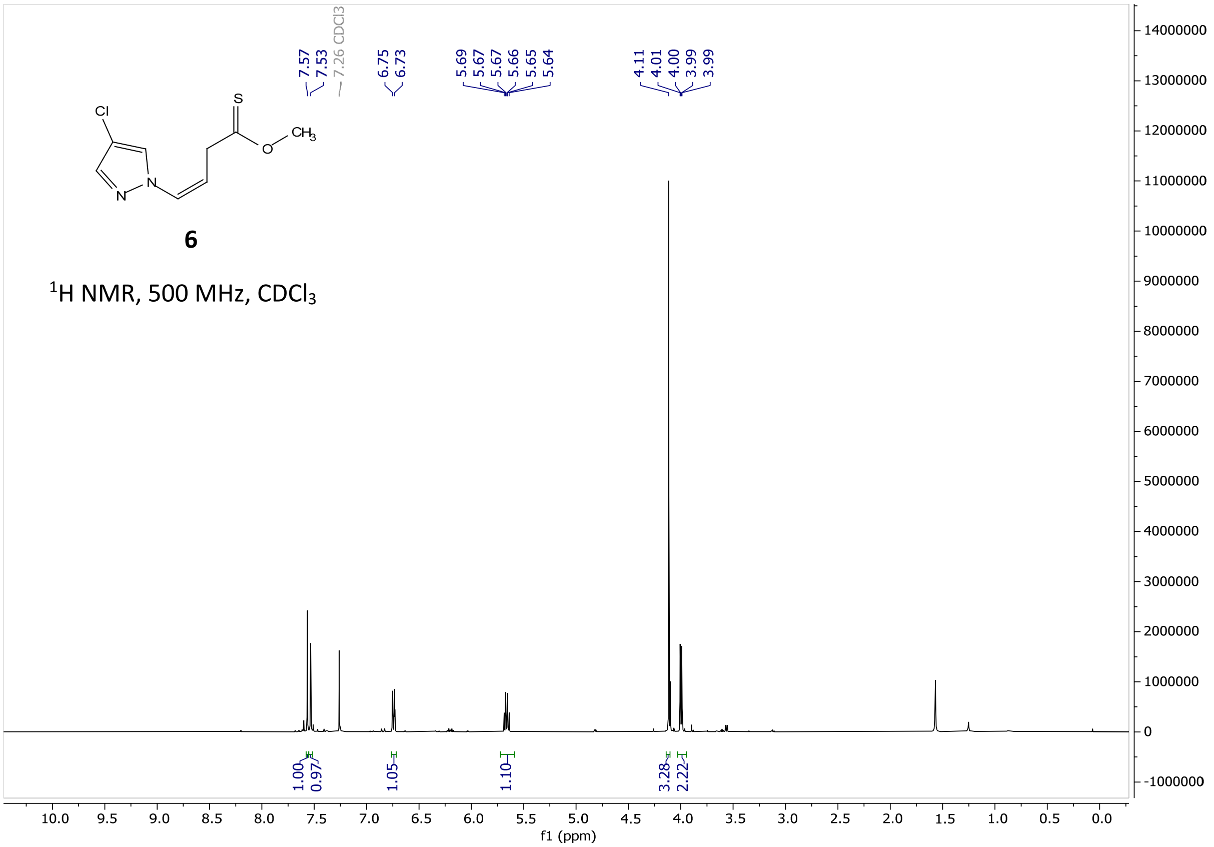
6
^{1}H NMR, 500 MHz, CDCl$_3$
7.57
7.53
7.26 CDCl3
6.75
6.73
5.69
5.67
5.67
5.66
5.65
5.64
4.11
4.01
4.00
3.99
3.99
1.00
0.97
1.05
1.10
3.28
2.22
f1 (ppm)

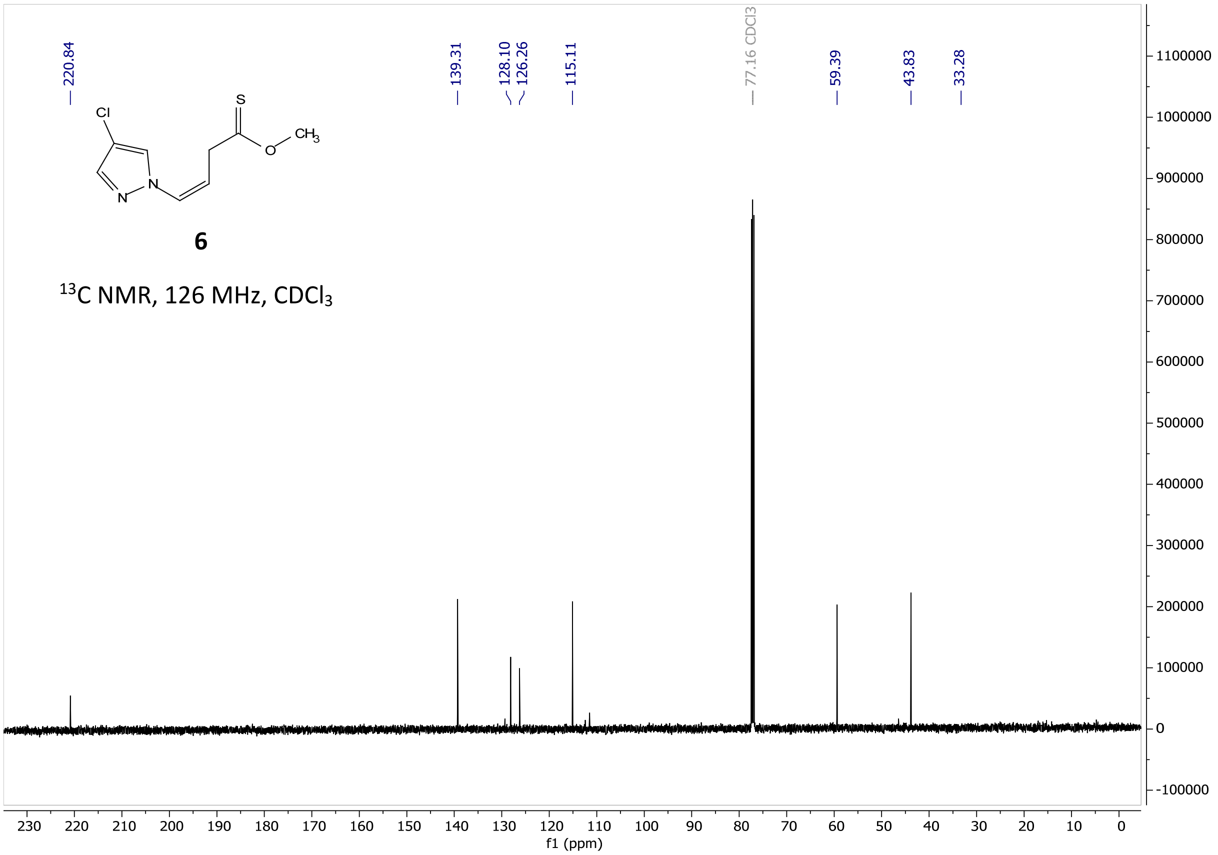
6
^{13}C NMR, 126 MHz, CDCl$_3$
220.84
139.31
128.10
126.26
115.11
77.16 CDCl3
59.39
43.83
33.28
f1 (ppm)

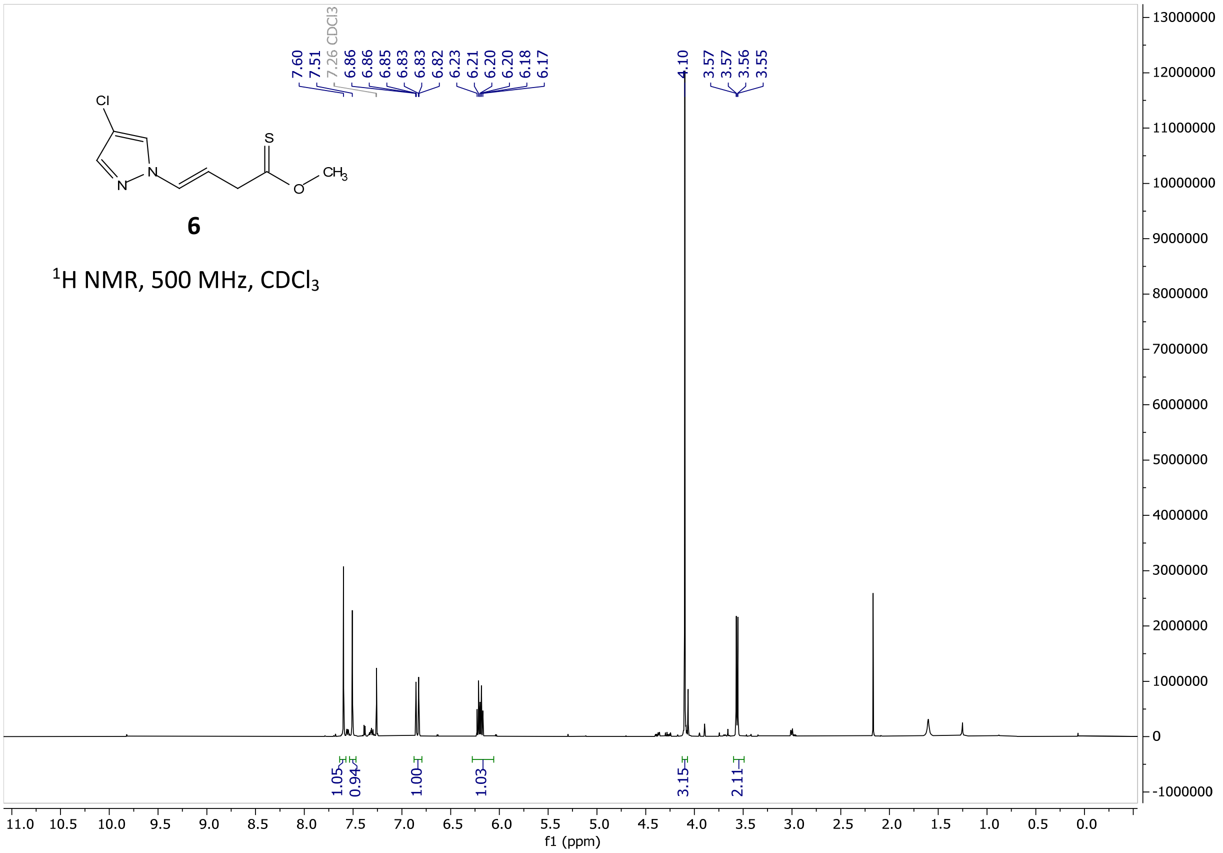
7.60
7.51
7.26 CDCl3
6.86
6.86
6.85
6.83
6.83
6.82
6.23
6.21
6.20
6.20
6.18
6.17
4.10
3.57
3.57
3.56
3.55
Cl
N
N
S
O
CH3
6
1H NMR, 500 MHz, CDCl3
1.05
0.94
1.00
1.03
3.15
2.11
f1 (ppm)

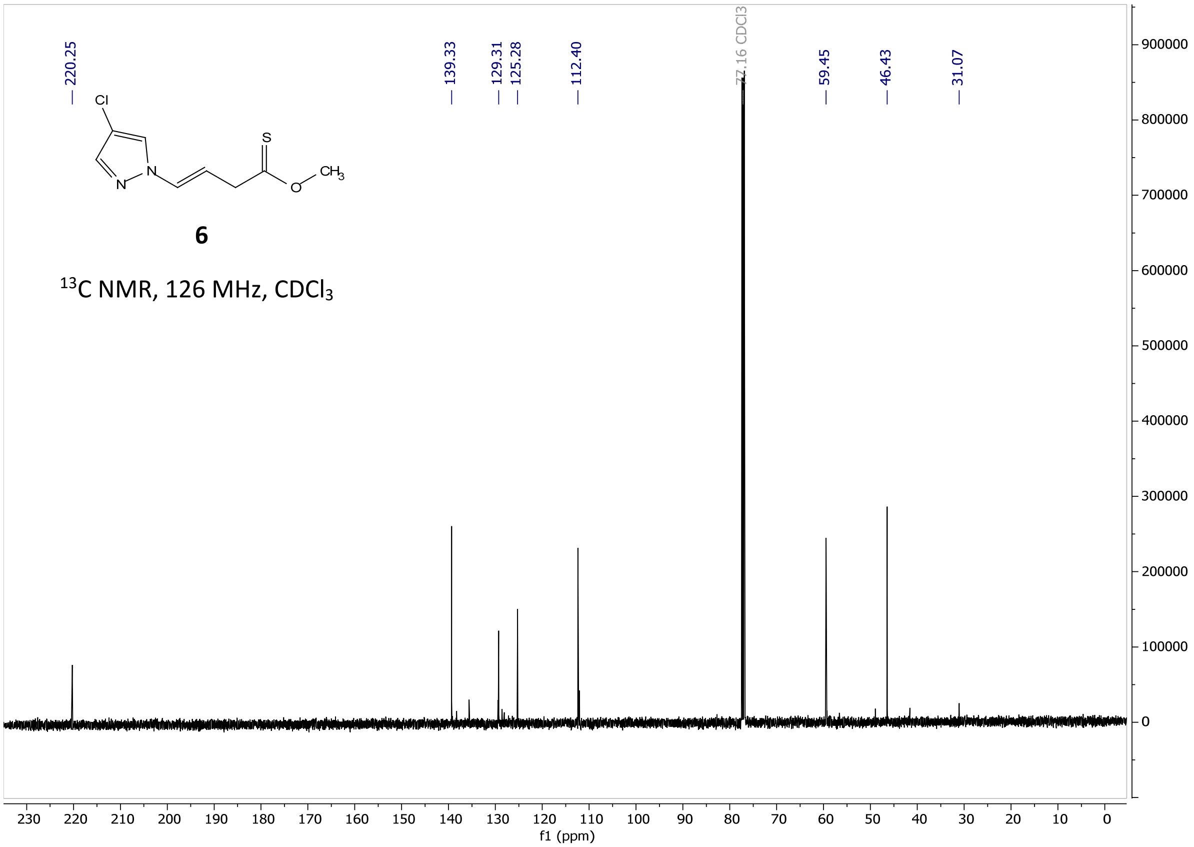
220.25
139.33
129.31
125.28
112.40
77.16 CDCl3
59.45
46.43
31.07
Cl
N
N
S
O
CH3
6
13C NMR, 126 MHz, CDCl3
f1 (ppm)

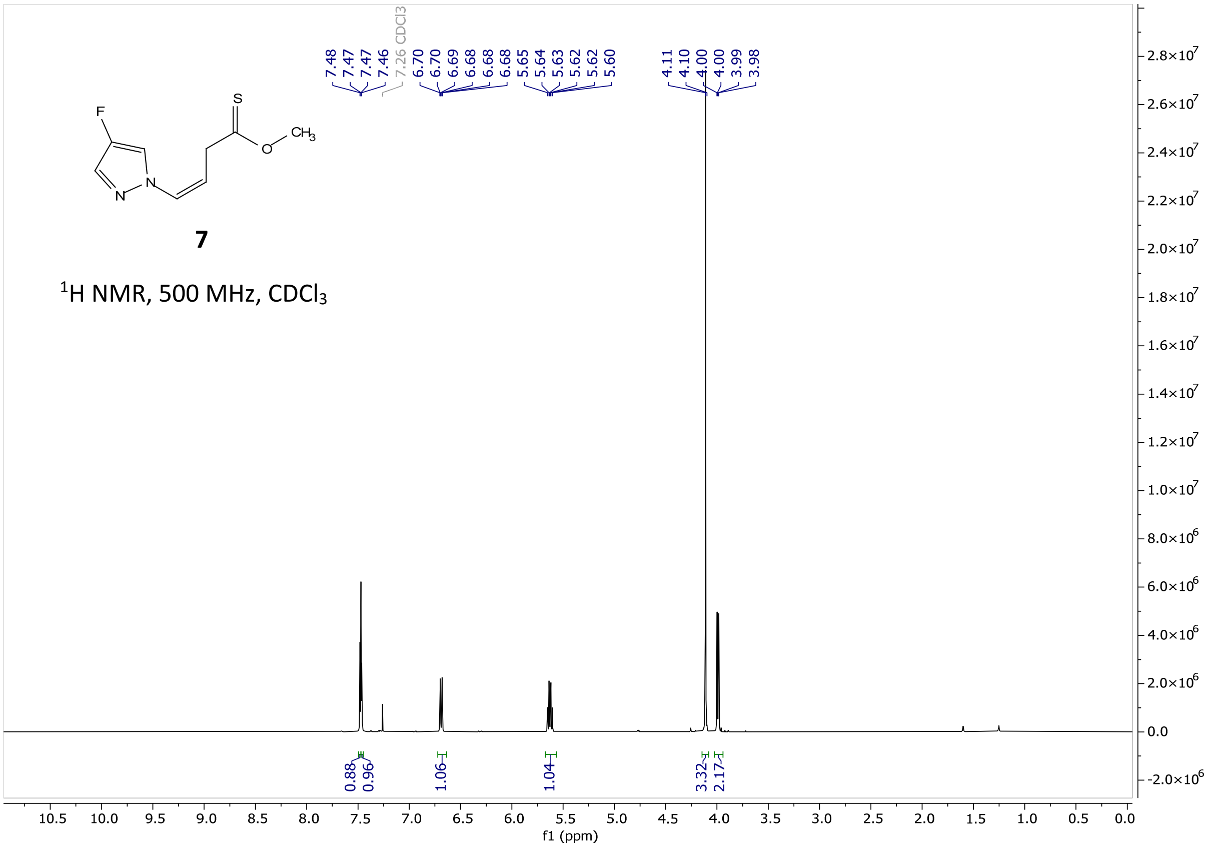

7.48
7.47
7.47
7.46
7.26 CDCl3
6.70
6.70
6.69
6.68
6.68
6.68
5.65
5.64
5.63
5.62
5.62
5.60
4.11
4.10
4.00
4.00
3.99
3.98
F
S
N
N
O
CH3
7
1H NMR, 500 MHz, CDCl3
0.88
0.96
1.06
1.04
3.32
2.17
f1 (ppm)

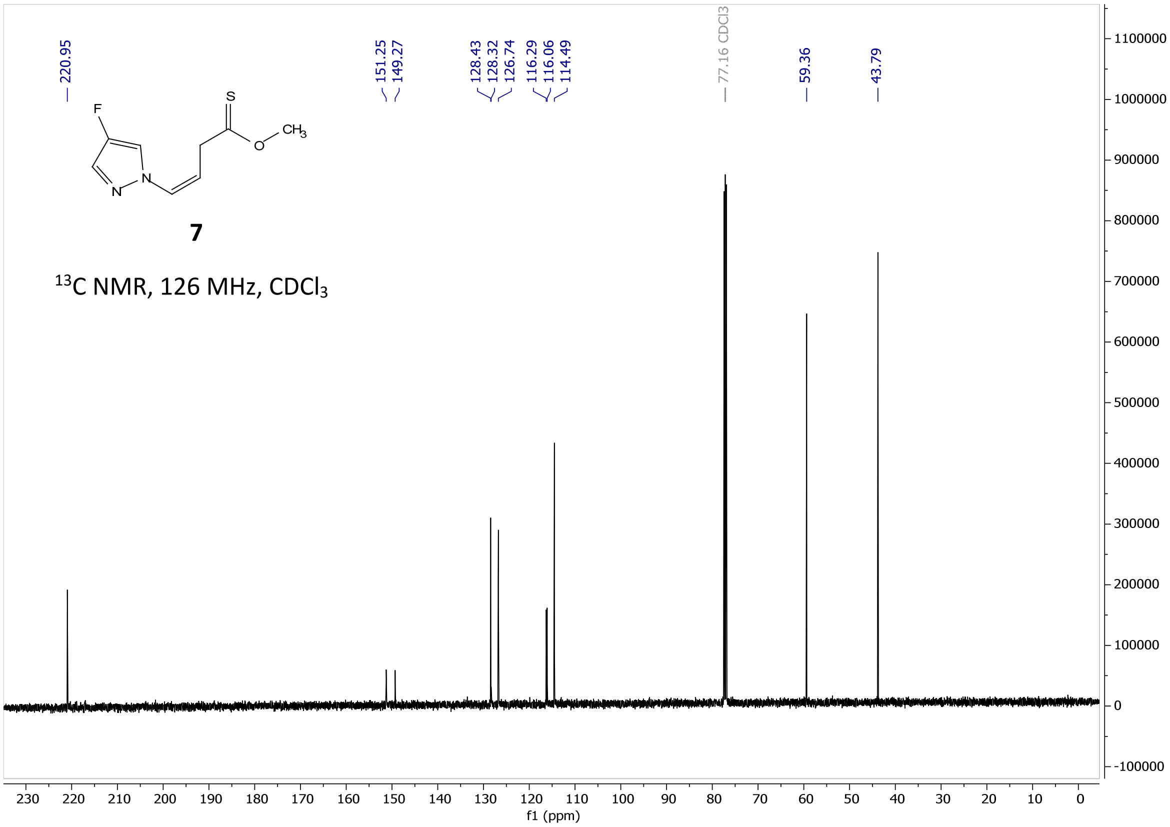

220.95
151.25
149.27
128.43
128.32
126.74
116.29
116.06
114.49
77.16 CDCl3
59.36
43.79
F
S
N
N
O
CH3
7
13C NMR, 126 MHz, CDCl3
f1 (ppm)

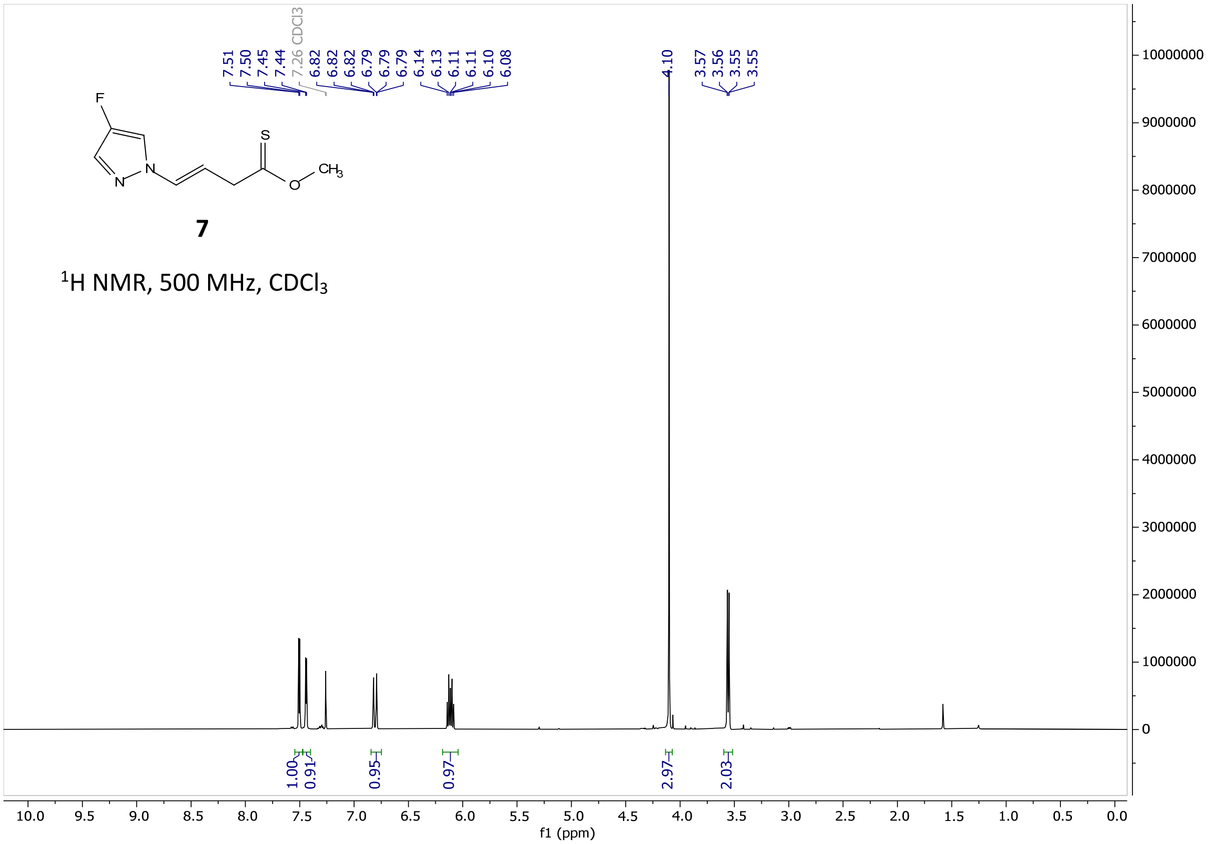
7
^{1}H NMR, 500 MHz, CDCl$_3$
7.51
7.50
7.45
7.44
7.26 CDCl3
6.82
6.82
6.82
6.79
6.79
6.79
6.14
6.13
6.11
6.11
6.10
6.08
4.10
3.57
3.56
3.55
3.55
1.00
0.91
0.95
0.97
2.97
2.03
f1 (ppm)

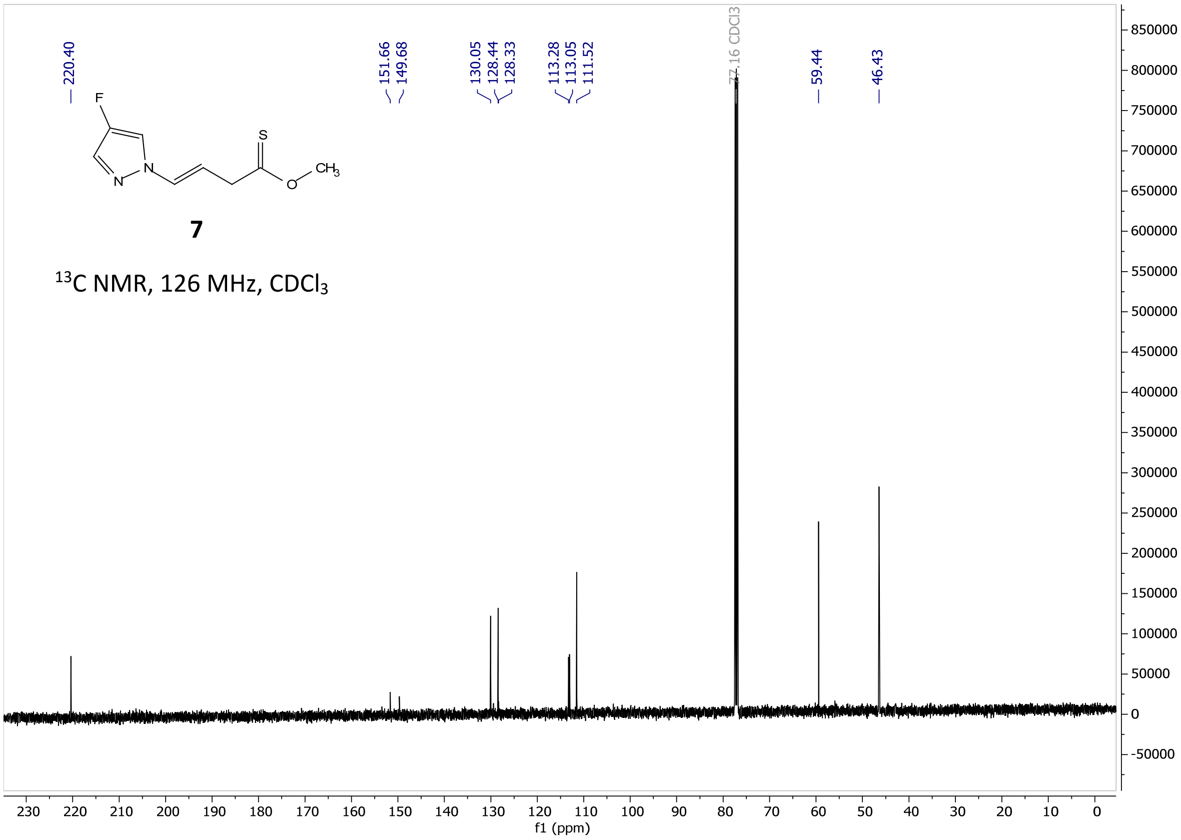
7
^{13}C NMR, 126 MHz, CDCl$_3$
220.40
151.66
149.68
130.05
128.44
128.33
113.28
113.05
111.52
77.16 CDCl3
59.44
46.43
f1 (ppm)

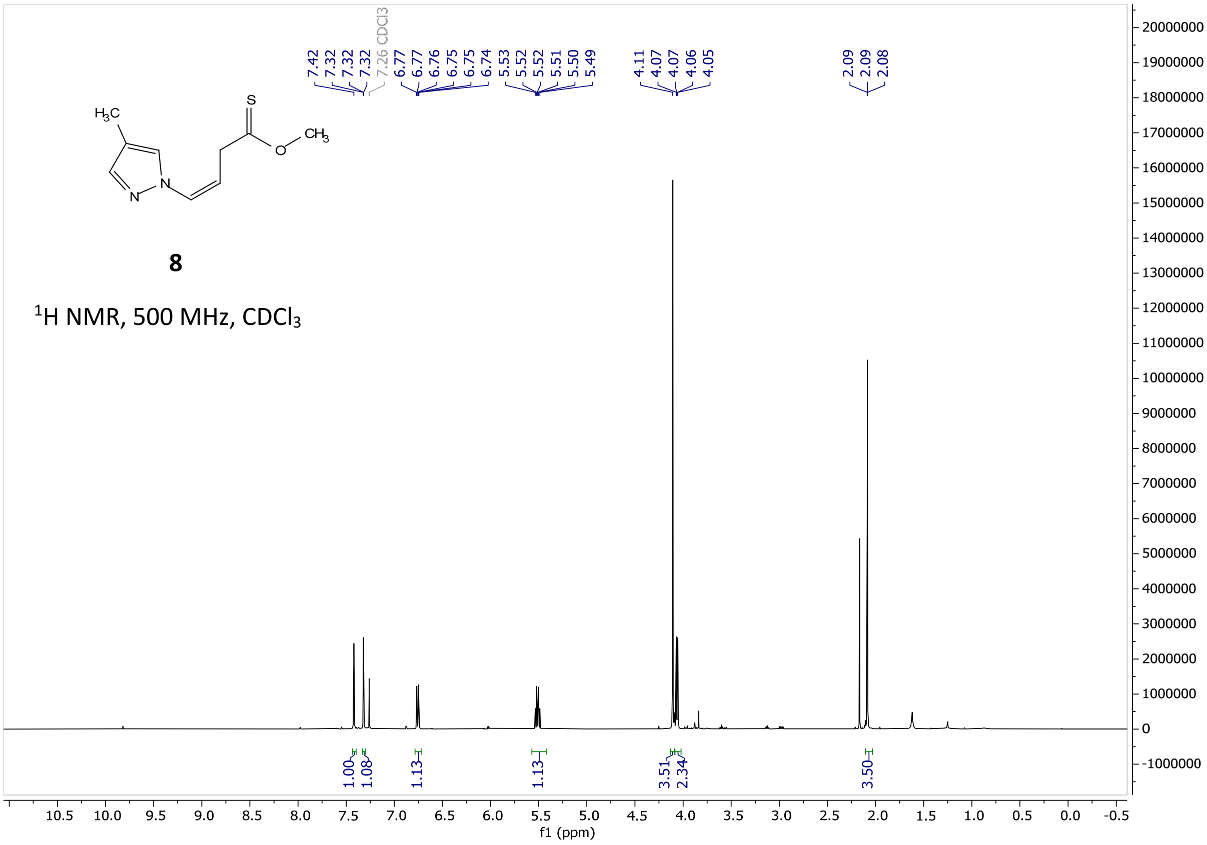
7.42
7.32
7.32
7.32
7.26 CDCl3
6.77
6.77
6.76
6.75
6.75
6.74
5.53
5.52
5.52
5.51
5.50
5.49
4.11
4.07
4.07
4.06
4.05
2.09
2.09
2.08
H_3C
S
O
CH_3
N
N
8
^1H NMR, 500 MHz, $CDCl_3$
1.00
1.08
1.13
1.13
3.51
2.34
3.50
f1 (ppm)

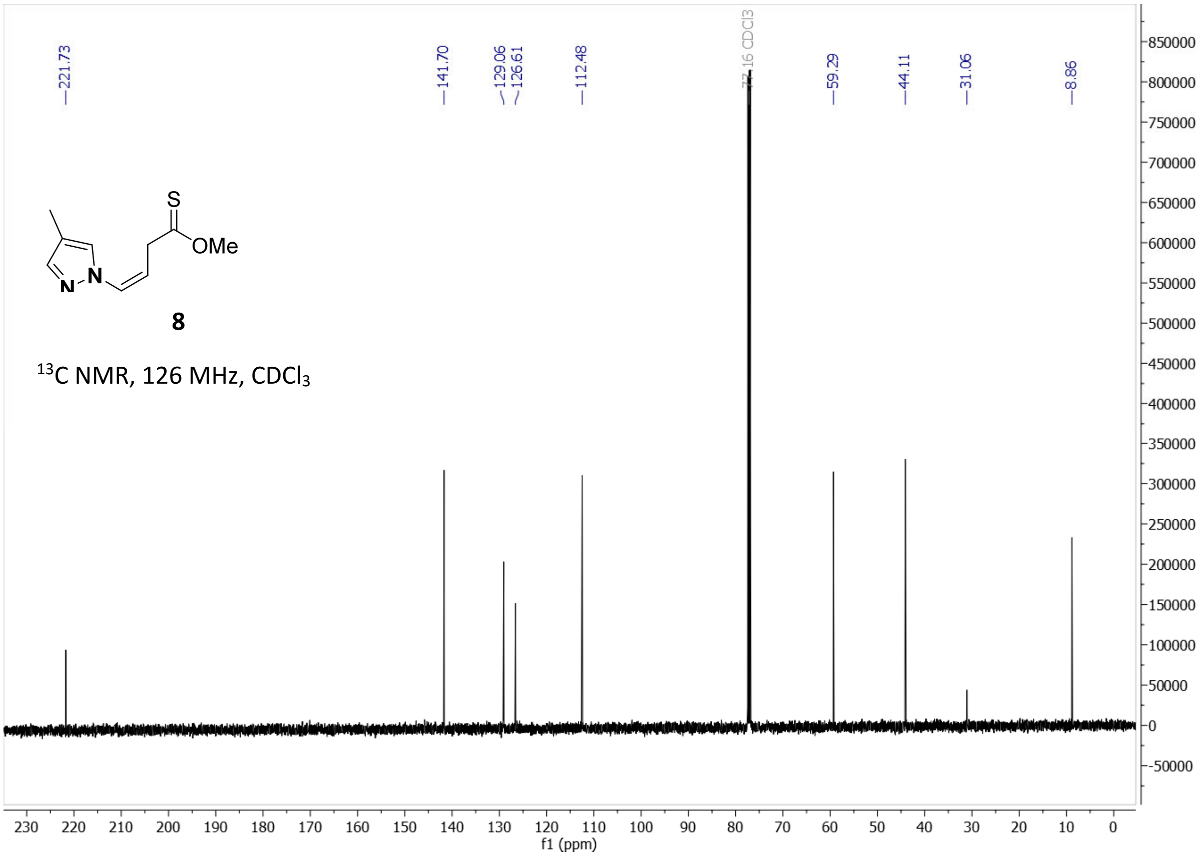
221.73
141.70
129.06
126.61
112.48
77.16 CDCl3
59.29
44.11
31.06
8.86
S
OMe
N
N
8
^{13}C NMR, 126 MHz, $CDCl_3$
f1 (ppm)

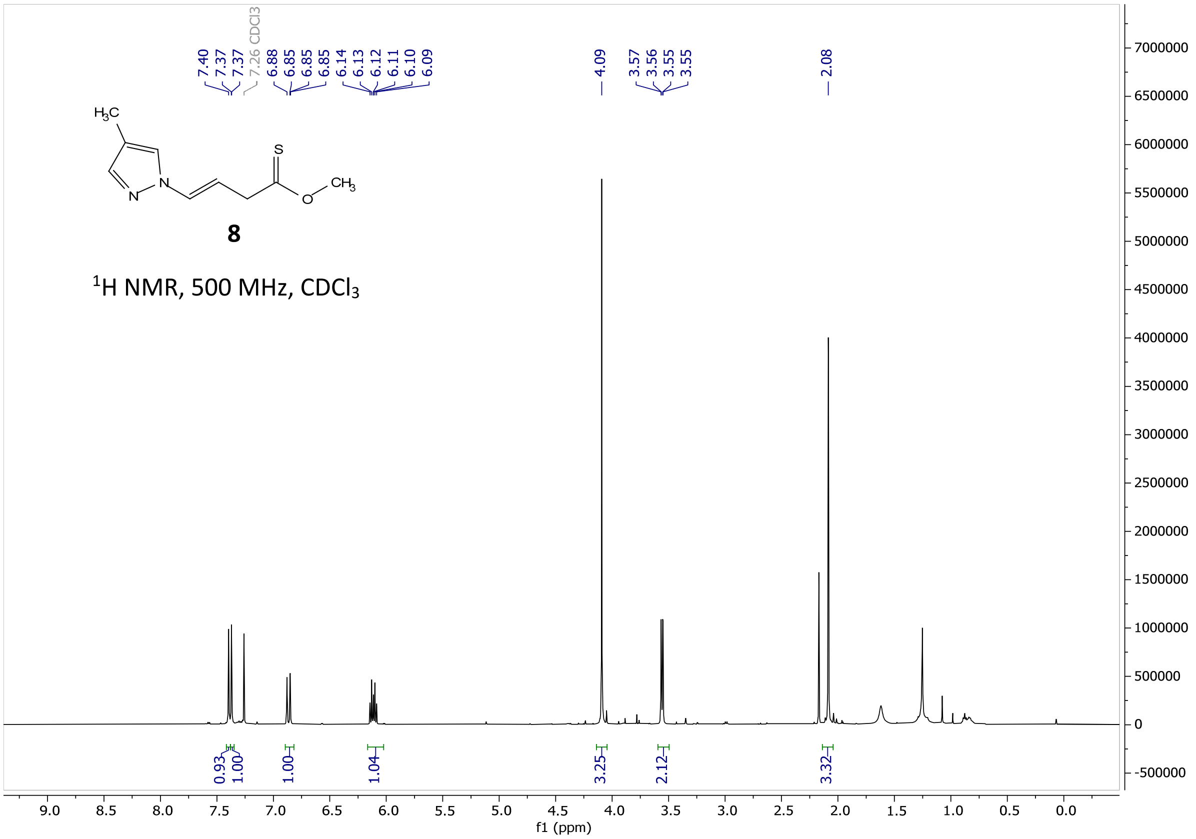
7.40
7.37
7.37
7.26 CDCl3
6.88
6.85
6.85
6.85
6.14
6.13
6.12
6.11
6.10
6.09
4.09
3.57
3.56
3.55
3.55
2.08
8
^{1}H NMR, 500 MHz, $CDCl_3$
0.93
1.00
1.00
1.04
3.25
2.12
3.32
f1 (ppm)

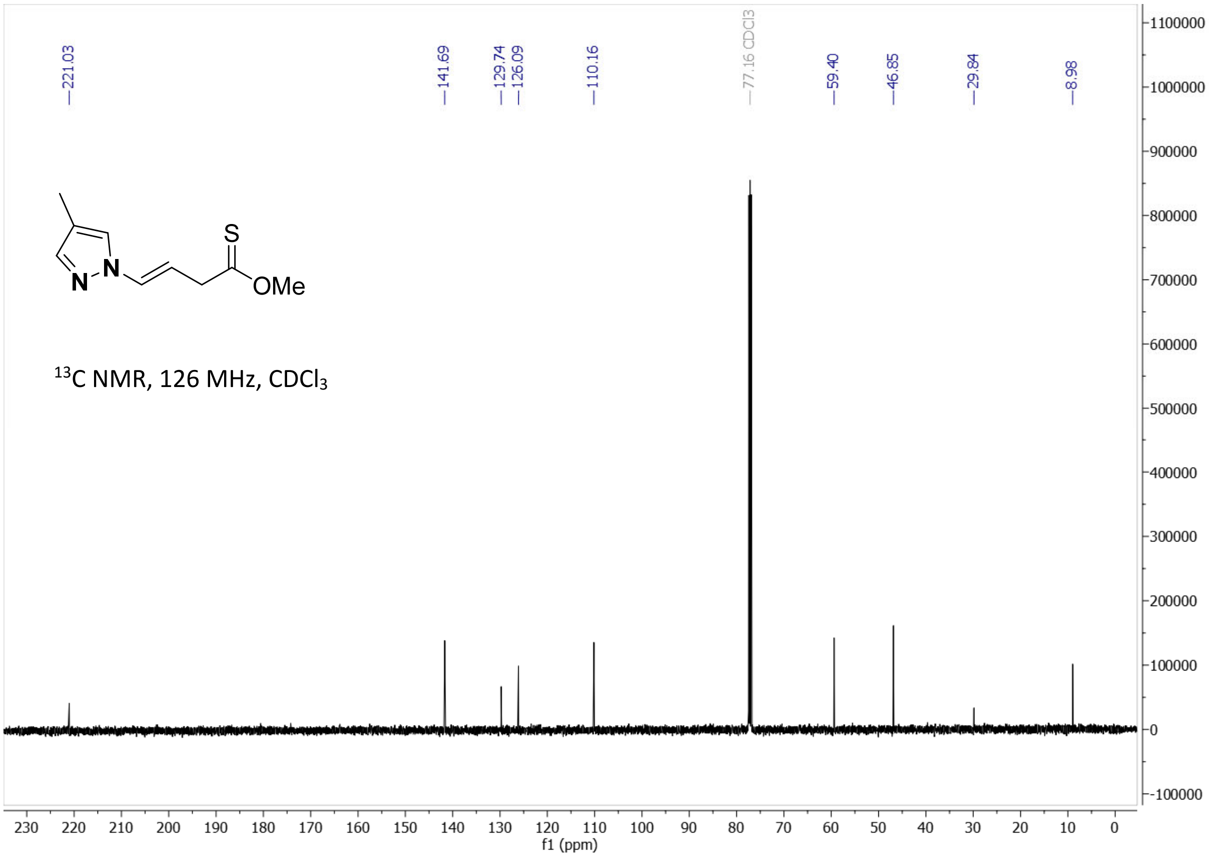
221.03
141.69
129.74
126.09
110.16
77.16 CDCl3
59.40
46.85
29.84
8.98
OMe
^{13}C NMR, 126 MHz, $CDCl_3$
f1 (ppm)

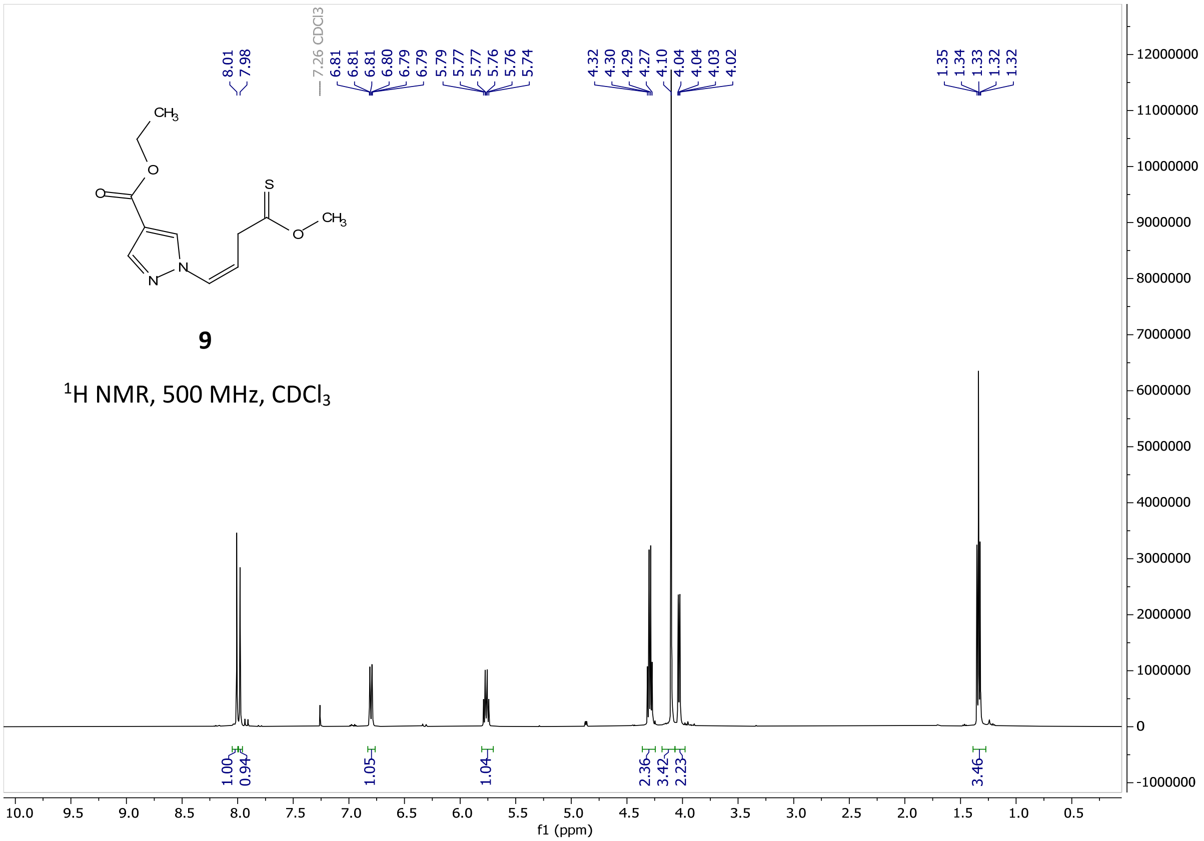
9
^{1}H NMR, 500 MHz, CDCl$_3$
8.01 7.98
7.26 CDCl3
6.81 6.81 6.80 6.79 6.79
5.79 5.77 5.77 5.76 5.76 5.74
4.32 4.30 4.29 4.27 4.10 4.04 4.04 4.03 4.02
1.35 1.34 1.33 1.32 1.32
1.00 0.94 1.05 1.04 2.36 3.42 2.23 3.46
f1 (ppm)

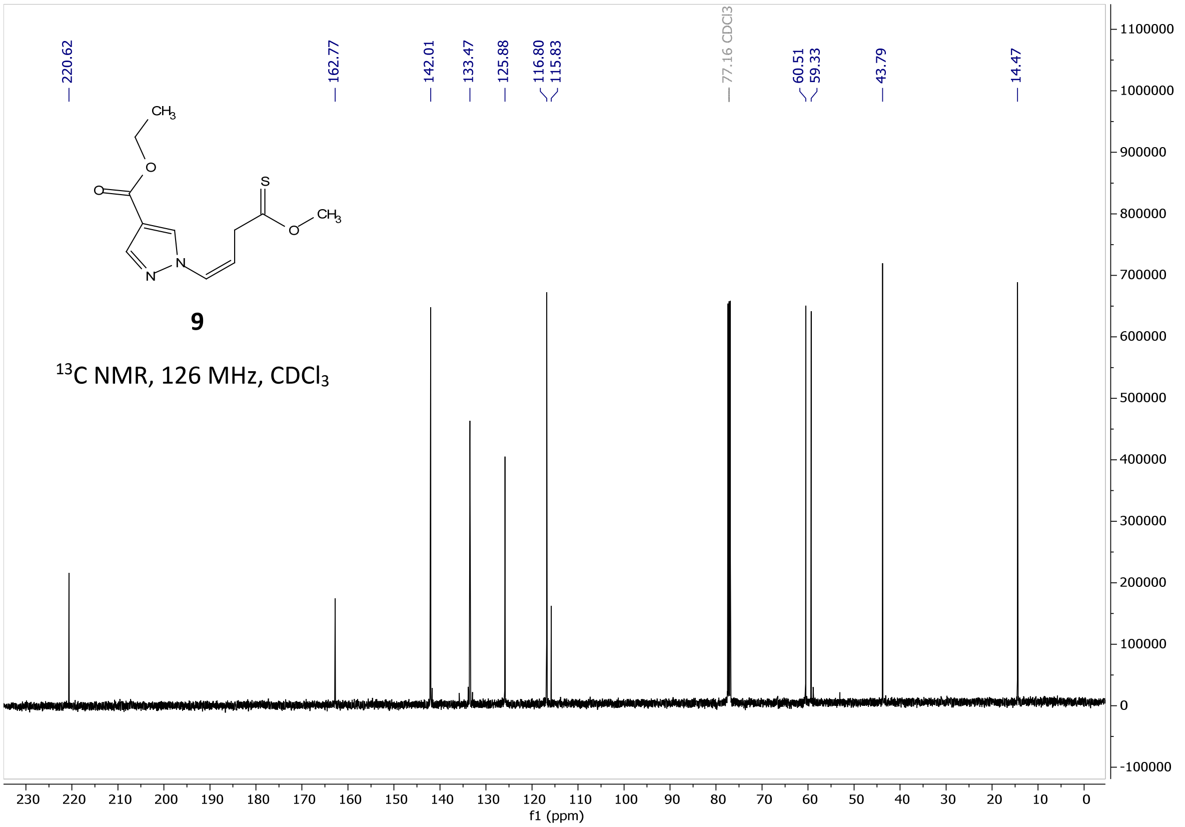
9
^{13}C NMR, 126 MHz, CDCl$_3$
220.62
162.77
142.01
133.47
125.88
116.80 115.83
77.16 CDCl3
60.51 59.33
43.79
14.47
f1 (ppm)

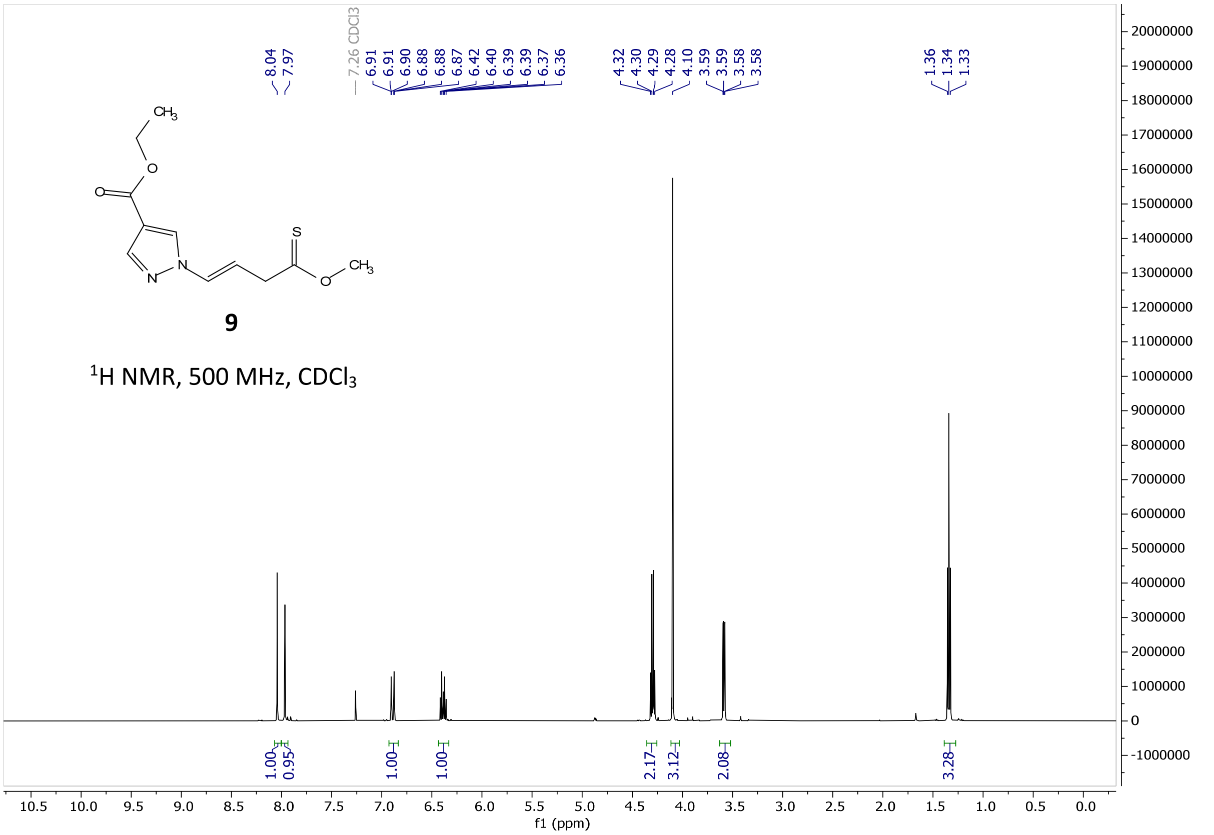
9
^{1}H NMR, 500 MHz, $CDCl_3$
8.04
7.97
7.26 CDCl3
6.91
6.91
6.90
6.88
6.88
6.87
6.42
6.40
6.39
6.39
6.37
6.36
4.32
4.30
4.29
4.28
4.10
3.59
3.59
3.58
3.58
1.36
1.34
1.33
1.00
0.95
1.00
1.00
2.17
3.12
2.08
3.28
f1 (ppm)

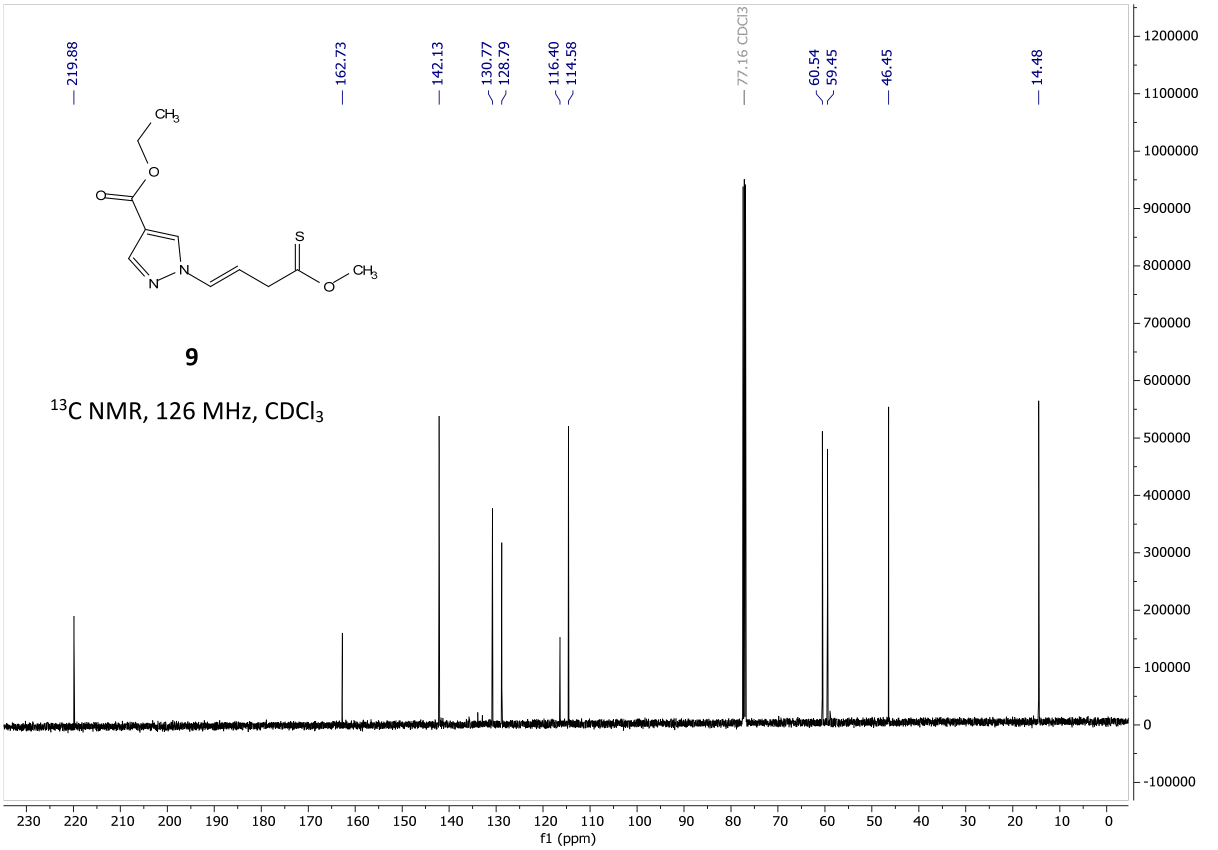
9
^{13}C NMR, 126 MHz, $CDCl_3$
219.88
162.73
142.13
130.77
128.79
116.40
114.58
77.16 CDCl3
60.54
59.45
46.45
14.48
f1 (ppm)

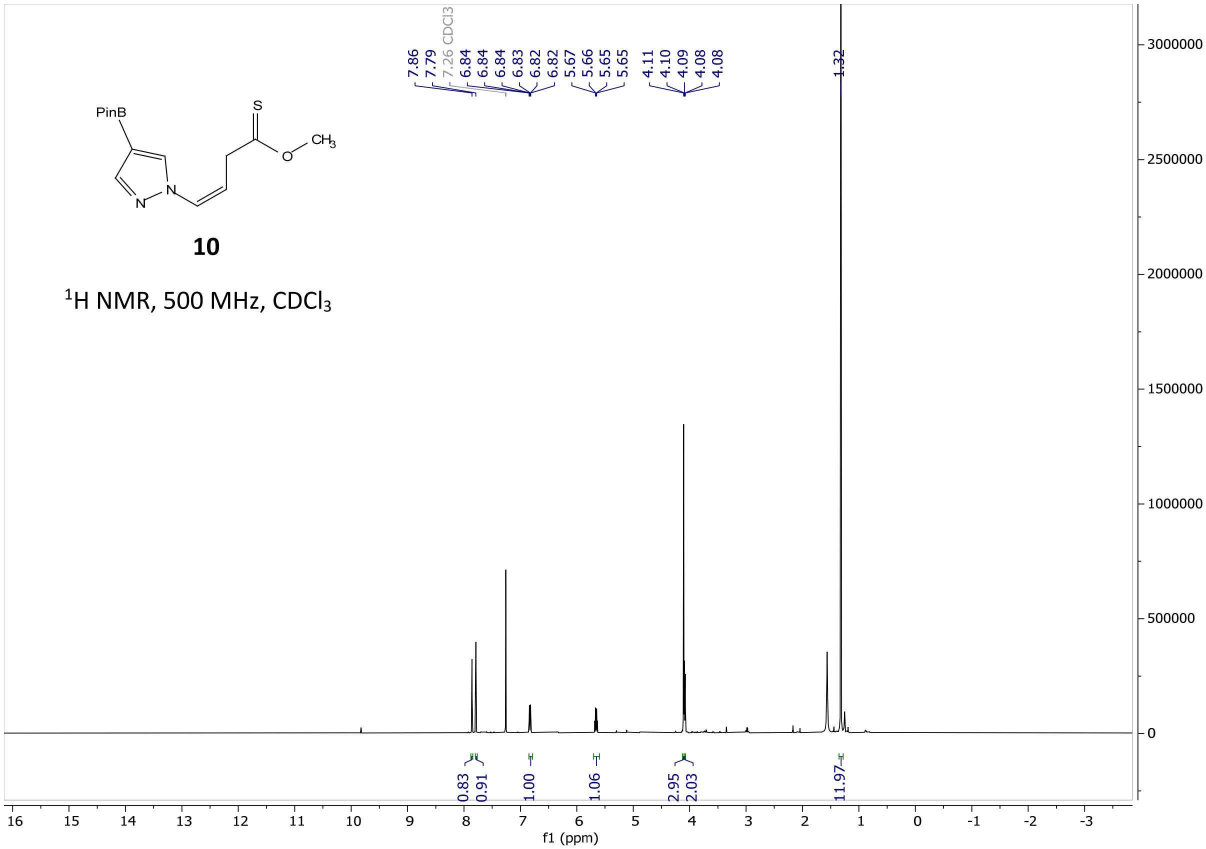
PinB
S
CH_3
O
N
N
10
^{1}H NMR, 500 MHz, $CDCl_3$
7.86
7.79
7.26 CDCl3
6.84
6.84
6.84
6.83
6.82
6.82
5.67
5.66
5.65
5.65
4.11
4.10
4.09
4.08
4.08
1.32
0.83
0.91
1.00
1.06
2.95
2.03
11.97
f1 (ppm)

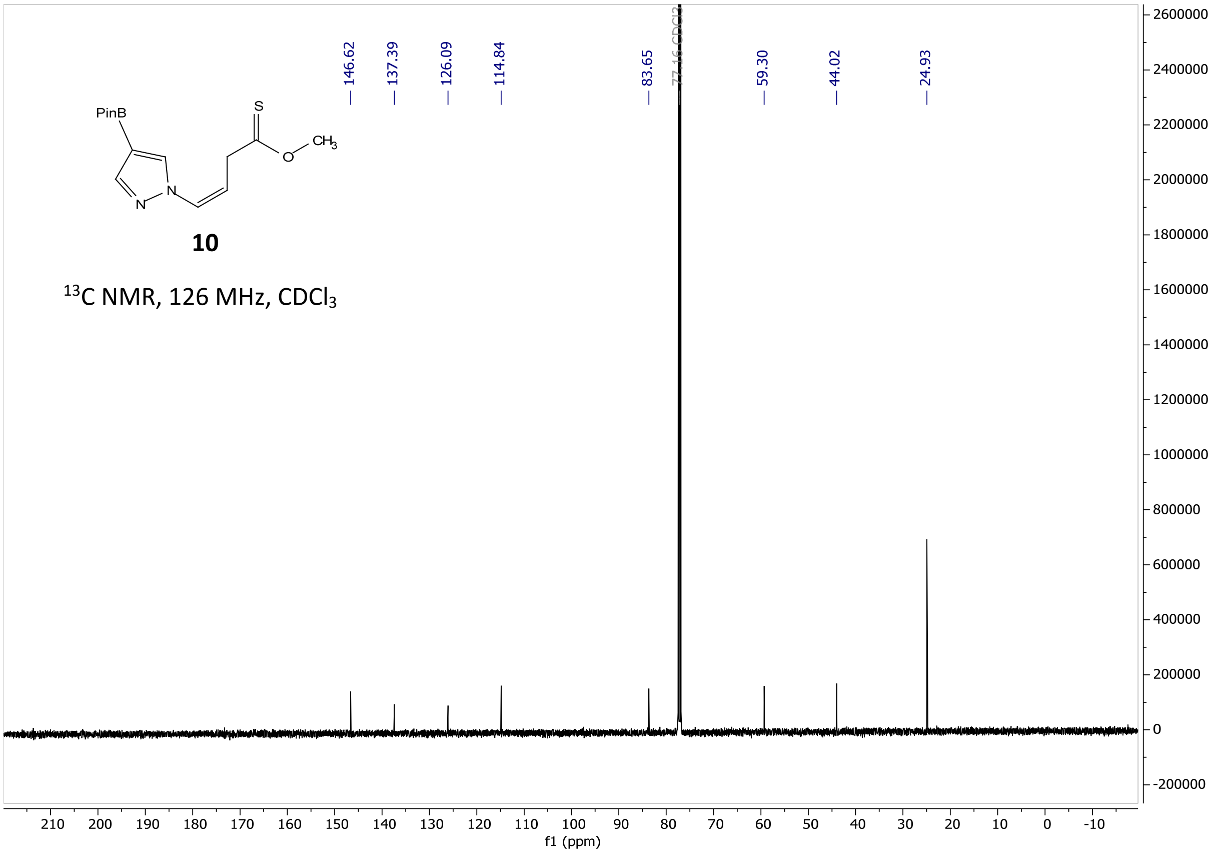
PinB
S
CH_3
O
N
N
10
^{13}C NMR, 126 MHz, $CDCl_3$
146.62
137.39
126.09
114.84
83.65
77.16 CDCl3
59.30
44.02
24.93
f1 (ppm)

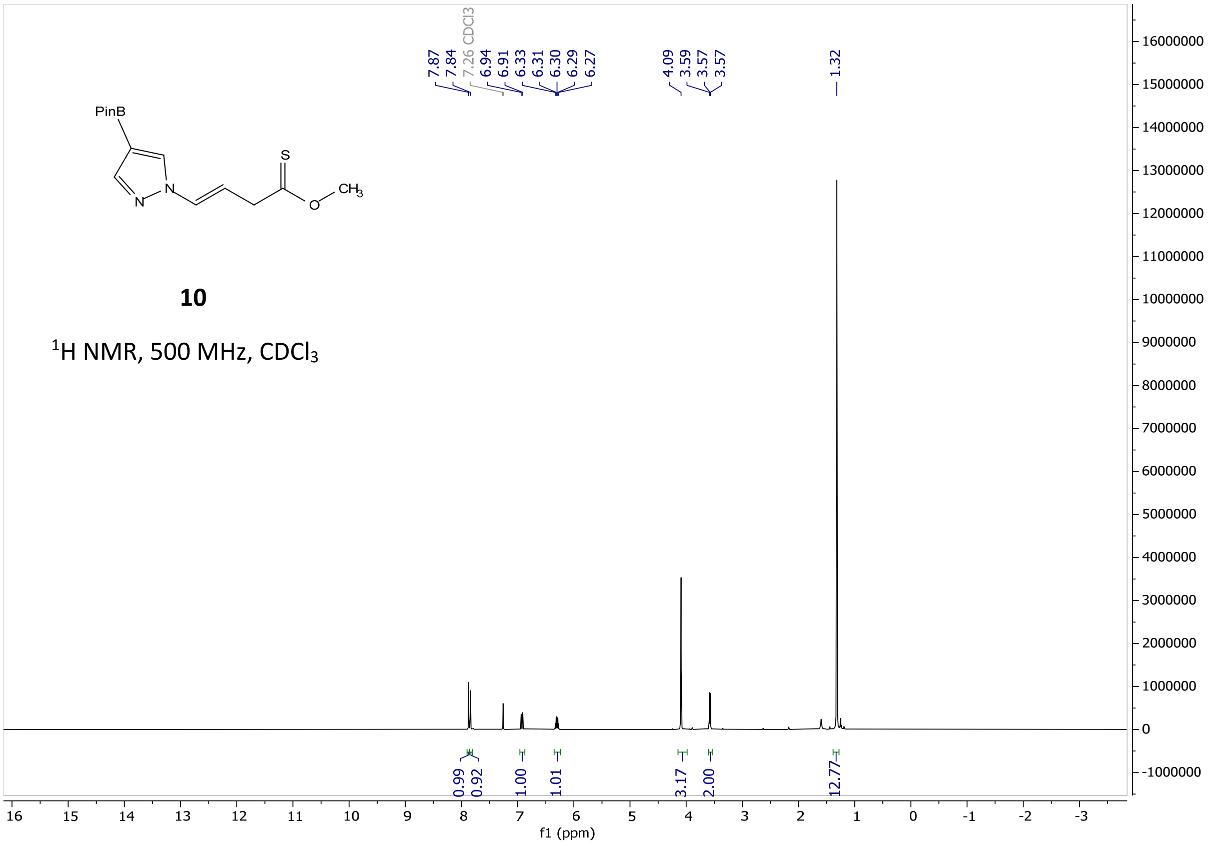
PinB
S
N
N
O
CH3
10
1H NMR, 500 MHz, CDCl3
7.87
7.84
7.26 CDCl3
6.94
6.91
6.33
6.31
6.30
6.29
6.27
4.09
3.59
3.57
3.57
1.32
0.99
0.92
1.00
1.01
3.17
2.00
12.77
f1 (ppm)

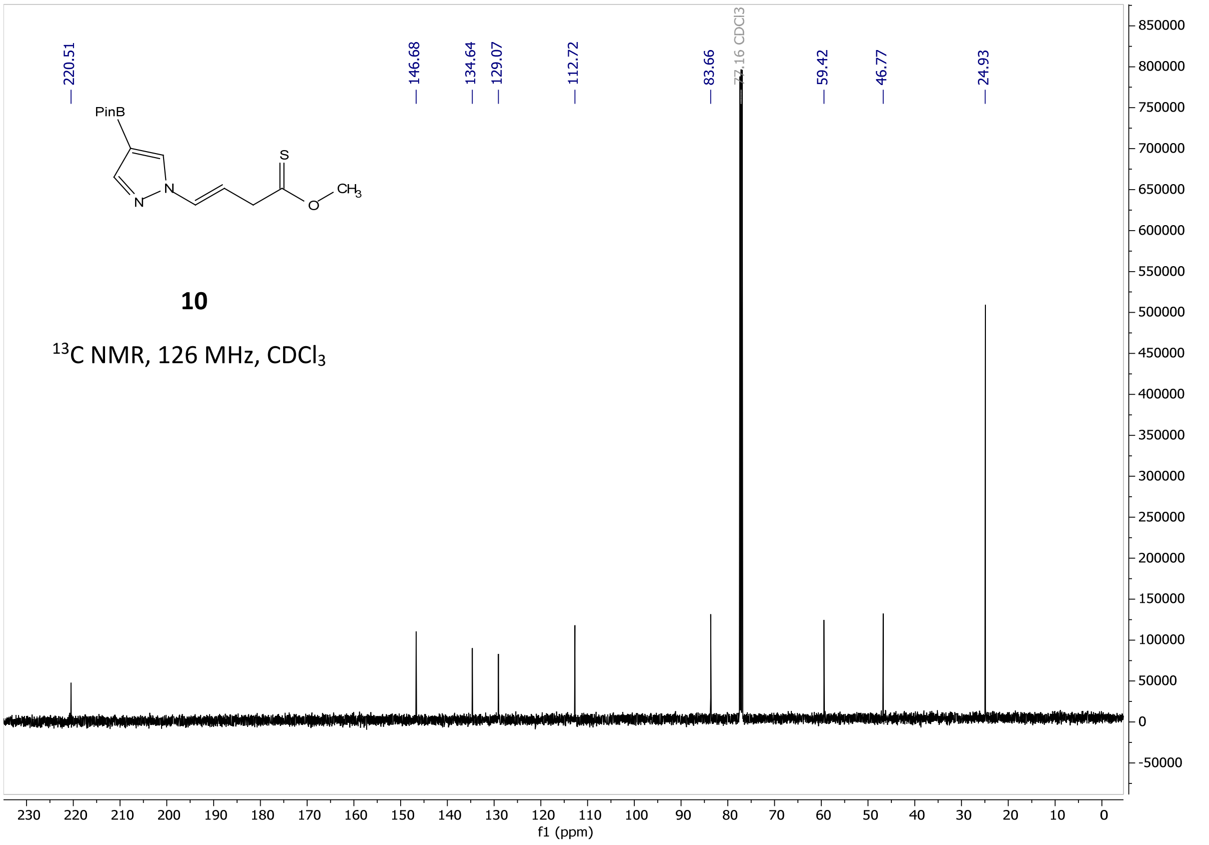
PinB
S
N
N
O
CH3
10
13C NMR, 126 MHz, CDCl3
220.51
146.68
134.64
129.07
112.72
83.66
77.16 CDCl3
59.42
46.77
24.93
f1 (ppm)

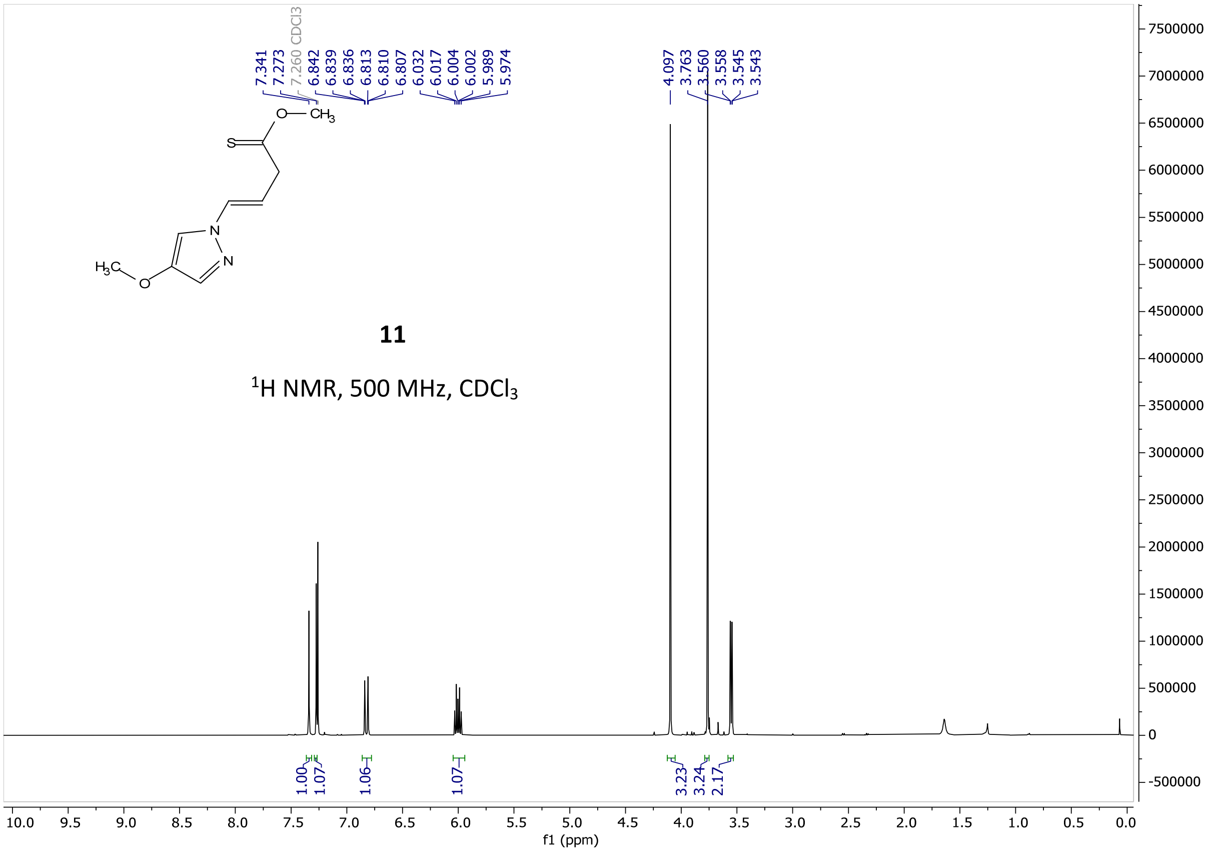
7.341
7.273
7.260 CDCl3
6.842
6.839
6.836
6.813
6.810
6.807
6.032
6.017
6.004
6.002
5.989
5.974
4.097
3.763
3.560
3.558
3.545
3.543
1.00
1.07
1.06
1.07
3.23
3.24
2.17
11
^{1}H NMR, 500 MHz, CDCl$_3$
f1 (ppm)

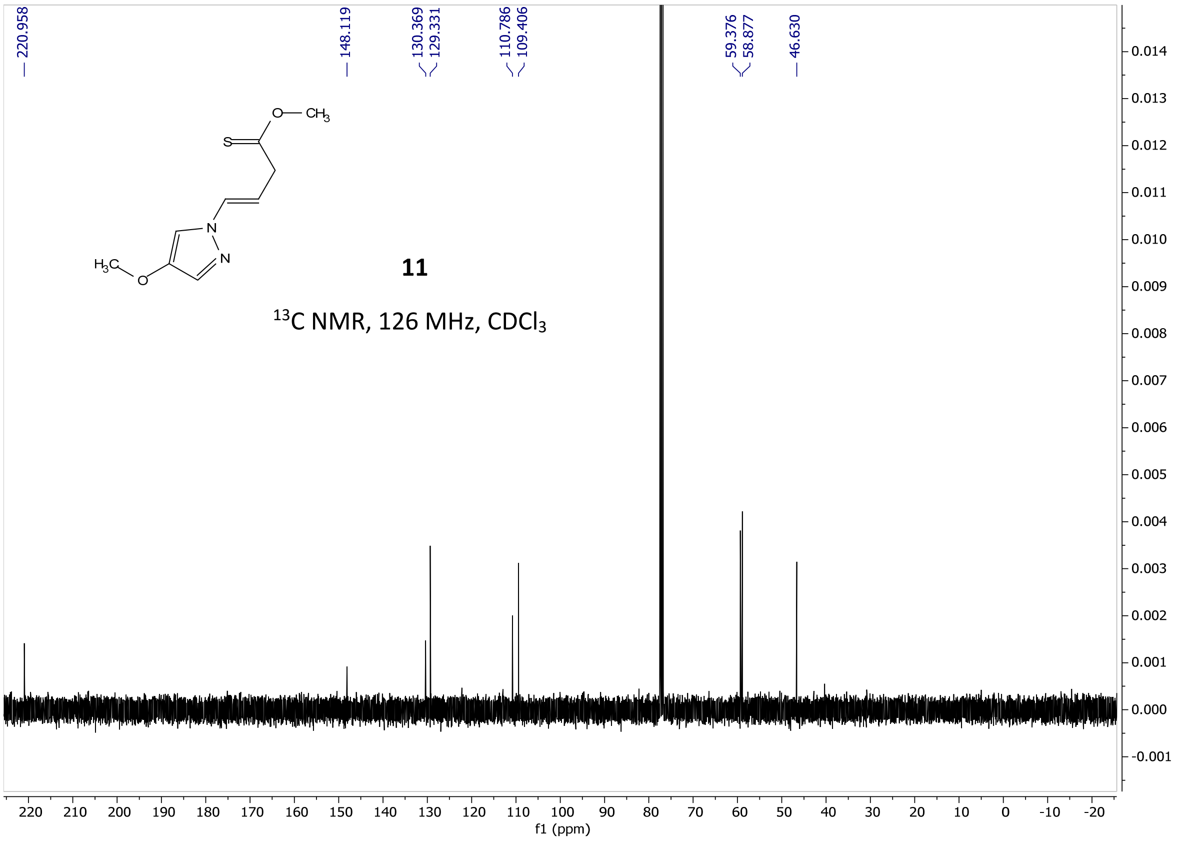
220.958
148.119
130.369
129.331
110.786
109.406
59.376
58.877
46.630
11
^{13}C NMR, 126 MHz, CDCl$_3$
f1 (ppm)

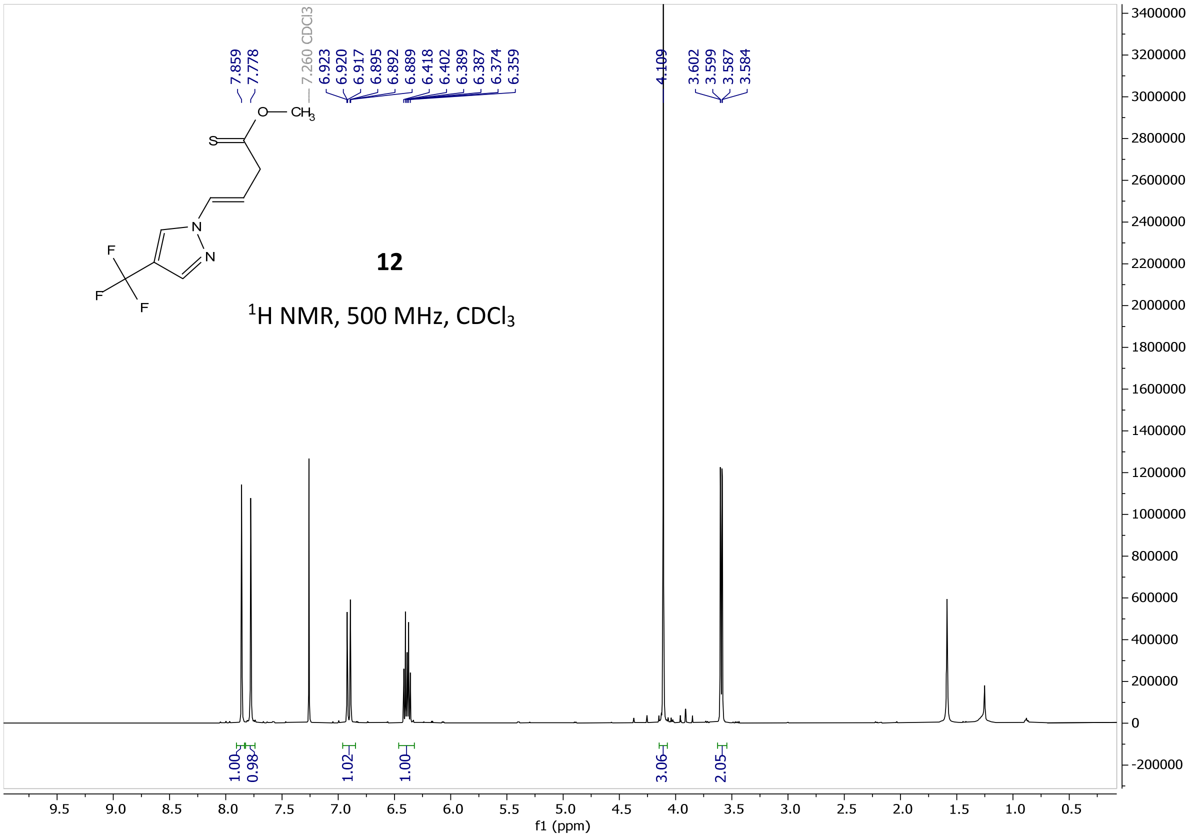
7.859
7.778
7.260 CDCl3
6.923
6.920
6.917
6.895
6.892
6.889
6.418
6.402
6.389
6.387
6.374
6.359
4.109
3.602
3.599
3.587
3.584
12
^{1}H NMR, 500 MHz, $CDCl_3$
1.00
0.98
1.02
1.00
3.06
2.05
f1 (ppm)

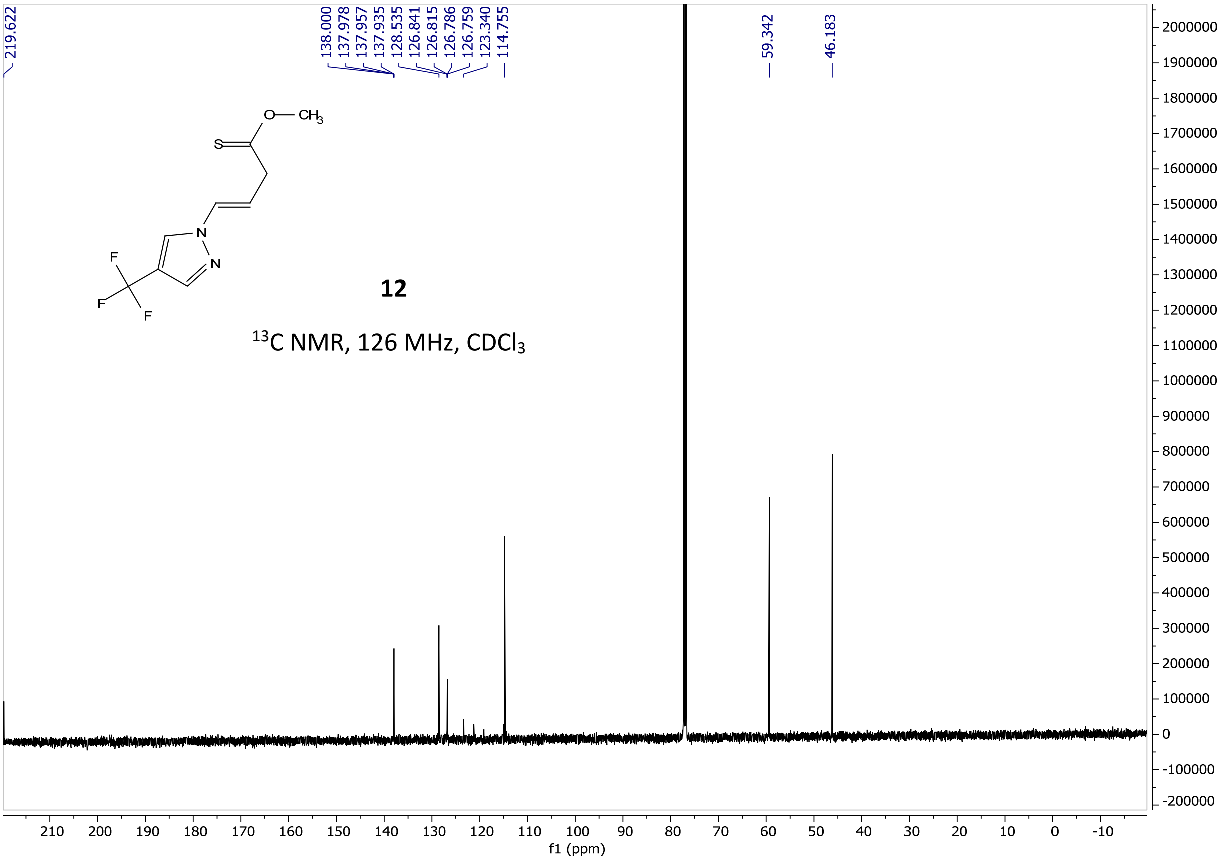
219.622
138.000
137.978
137.957
137.935
128.535
126.841
126.815
126.786
126.759
123.340
114.755
59.342
46.183
12
^{13}C NMR, 126 MHz, $CDCl_3$
f1 (ppm)

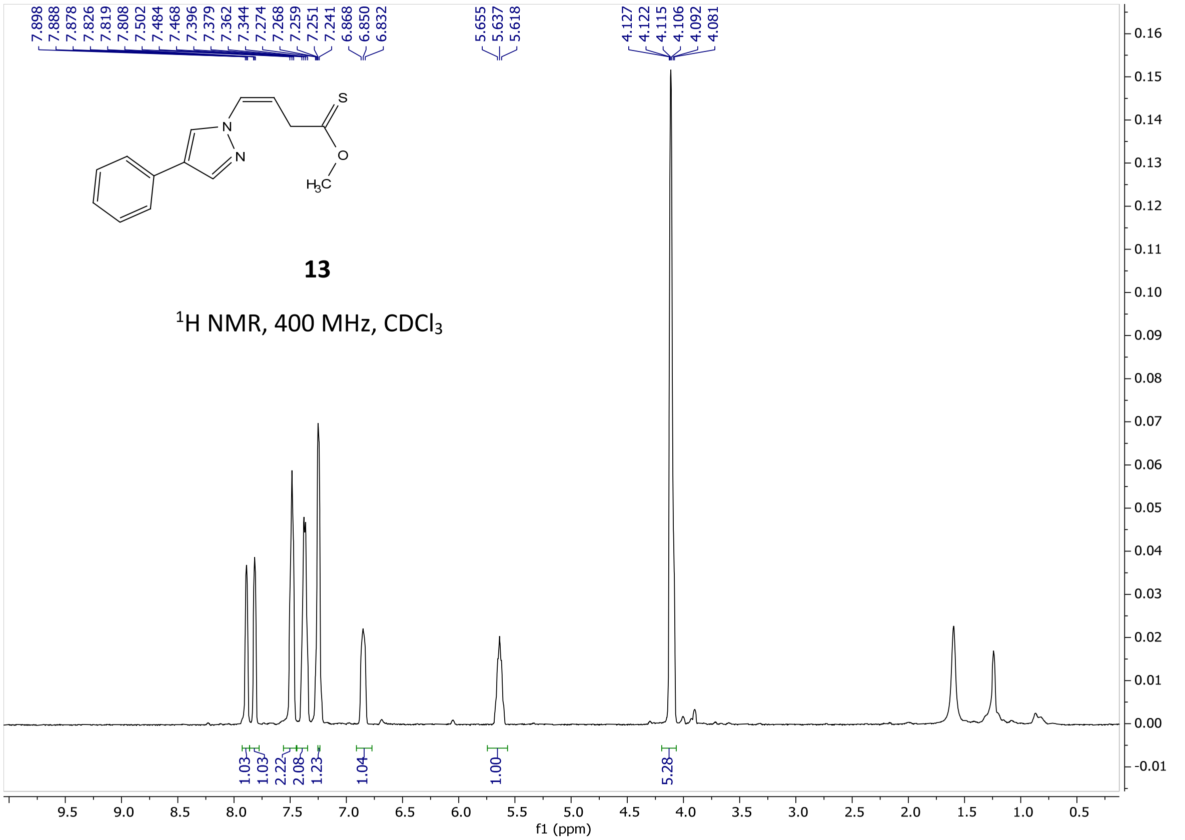
7.898
7.888
7.878
7.826
7.819
7.808
7.502
7.484
7.468
7.396
7.379
7.362
7.344
7.274
7.268
7.259
7.251
7.241
6.868
6.850
6.832
5.655
5.637
5.618
4.127
4.122
4.115
4.106
4.092
4.081
13
^{1}H NMR, 400 MHz, CDCl$_3$
1.03
1.03
2.22
2.08
1.23
1.04
1.00
5.28
f1 (ppm)

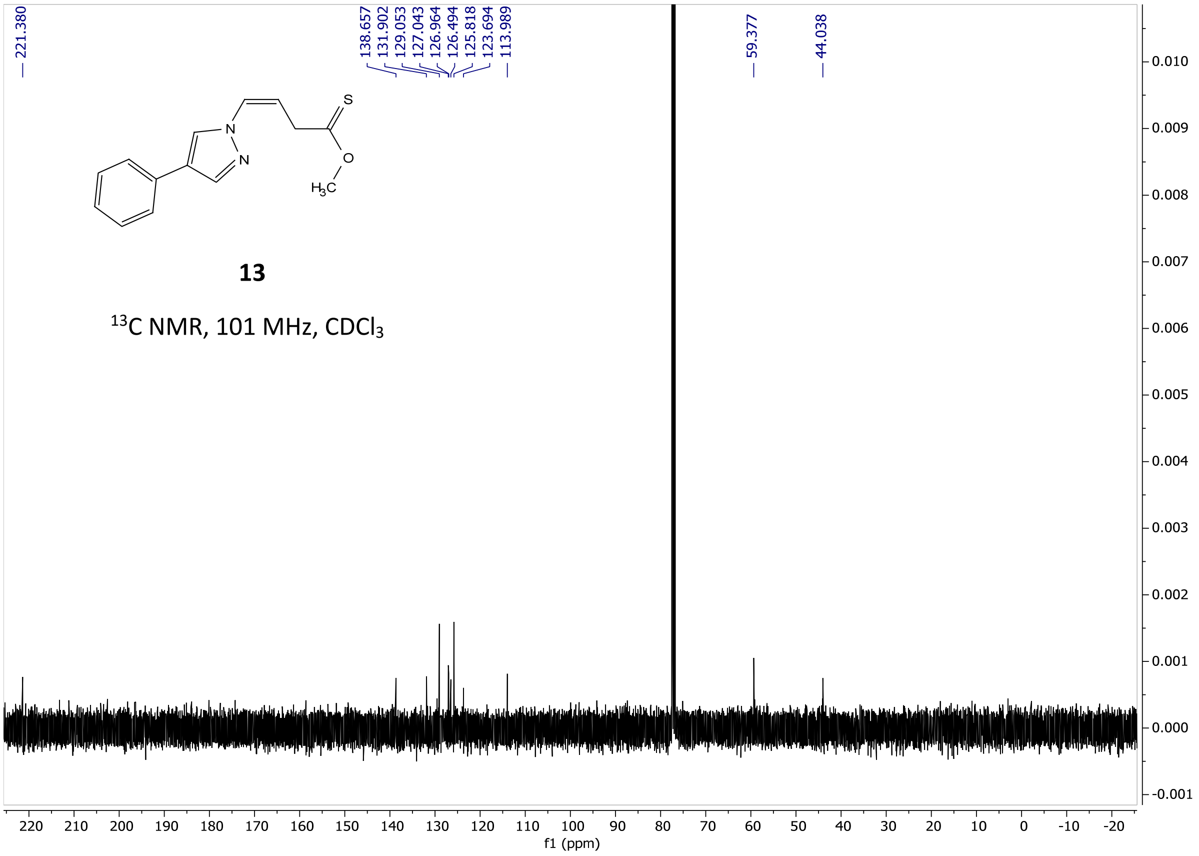
221.380
138.657
131.902
129.053
127.043
126.964
126.494
125.818
123.694
113.989
59.377
44.038
13
^{13}C NMR, 101 MHz, CDCl$_3$
f1 (ppm)

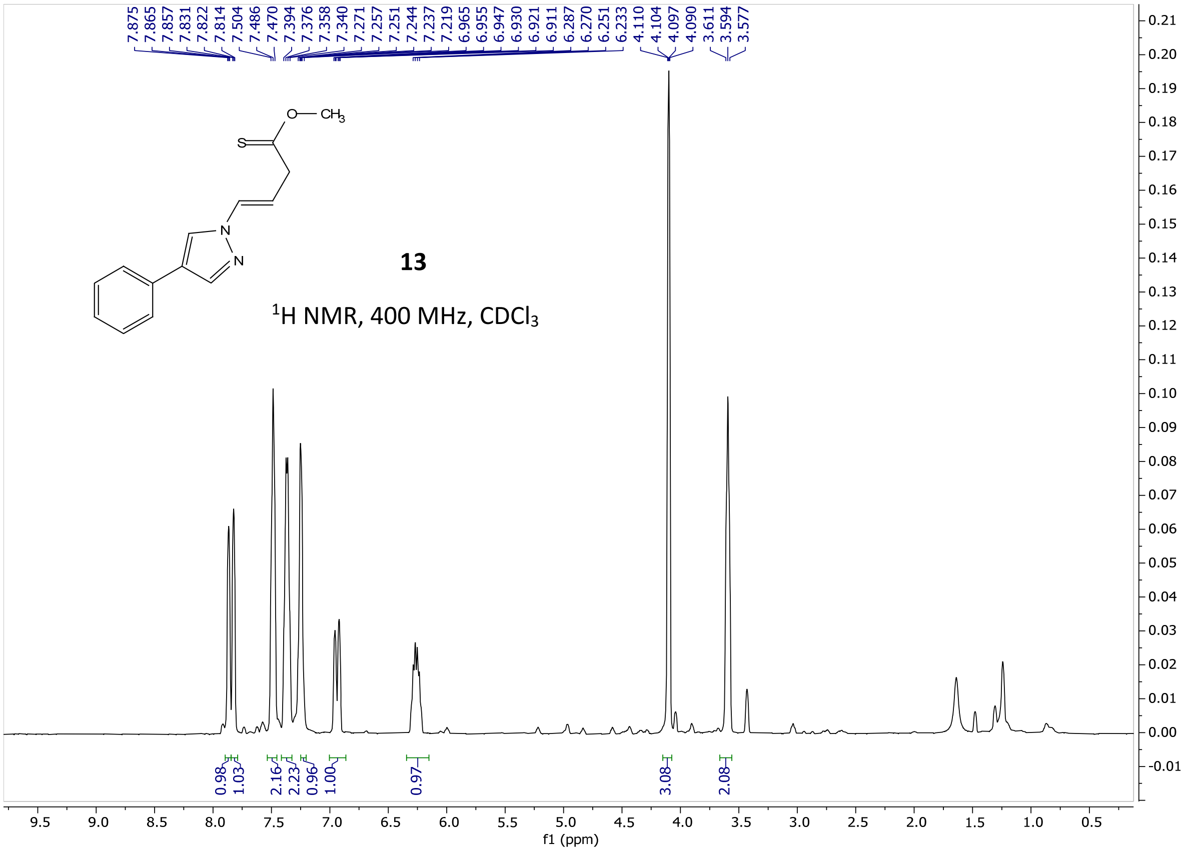
7.875
7.865
7.857
7.831
7.822
7.814
7.504
7.486
7.470
7.394
7.376
7.358
7.340
7.271
7.257
7.251
7.244
7.237
7.219
6.965
6.955
6.947
6.930
6.921
6.911
6.287
6.270
6.251
6.233
4.110
4.104
4.097
4.090
3.611
3.594
3.577
O
CH3
S
N
N
13
1H NMR, 400 MHz, CDCl3
0.98
1.03
2.16
2.23
0.96
1.00
0.97
3.08
2.08
f1 (ppm)

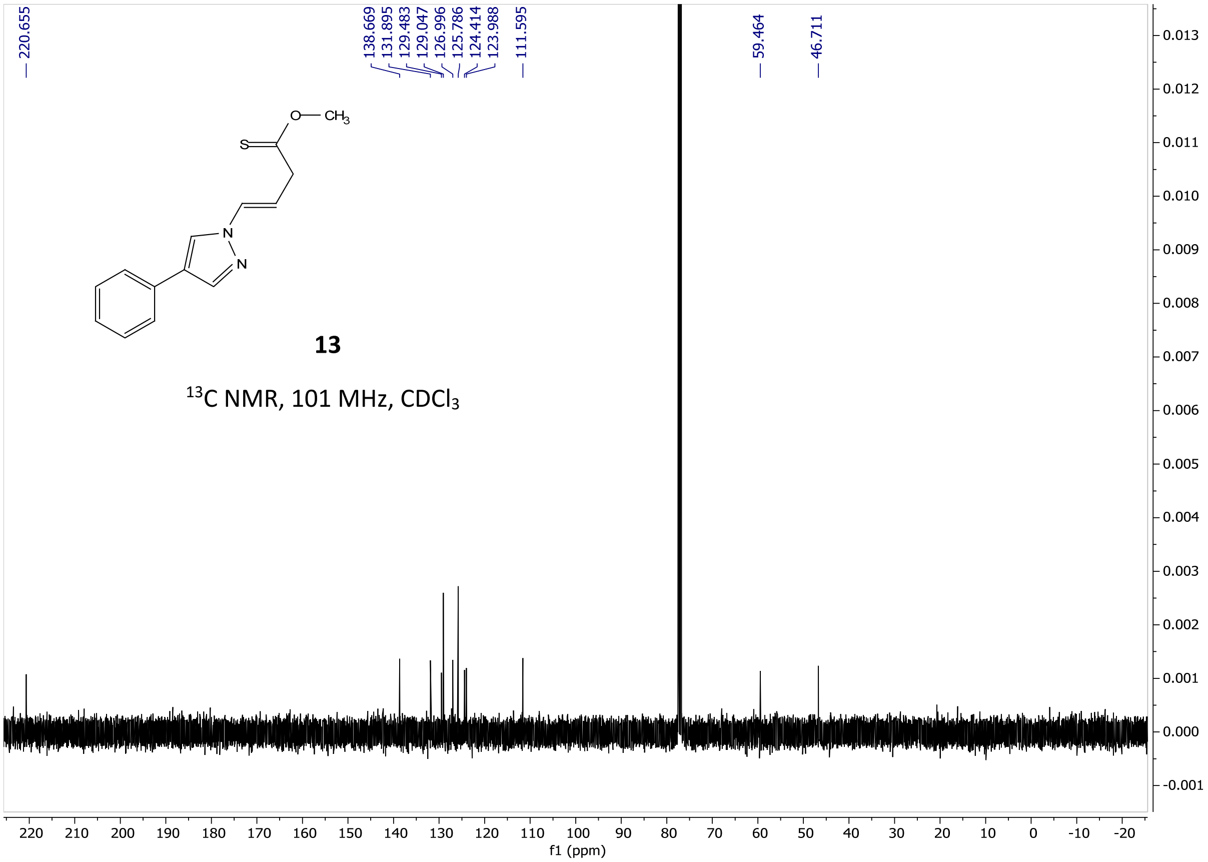
220.655
138.669
131.895
129.483
129.047
126.996
125.786
124.414
123.988
111.595
59.464
46.711
O
CH3
S
N
N
13
13C NMR, 101 MHz, CDCl3
f1 (ppm)

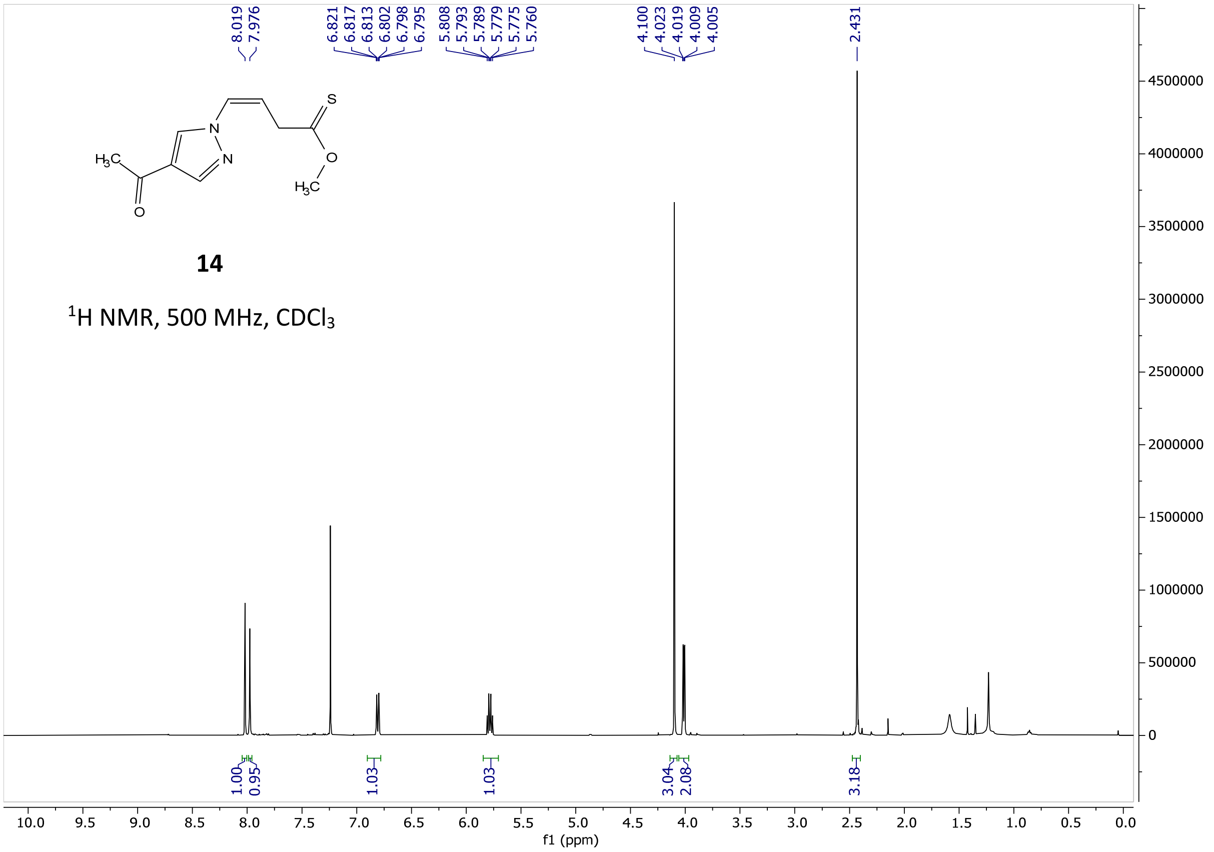
8.019
7.976
6.821
6.817
6.813
6.802
6.798
6.795
5.808
5.793
5.789
5.779
5.775
5.760
4.100
4.023
4.019
4.009
4.005
2.431
14
^{1}H NMR, 500 MHz, $CDCl_3$
1.00
0.95
1.03
1.03
3.04
2.08
3.18
f1 (ppm)

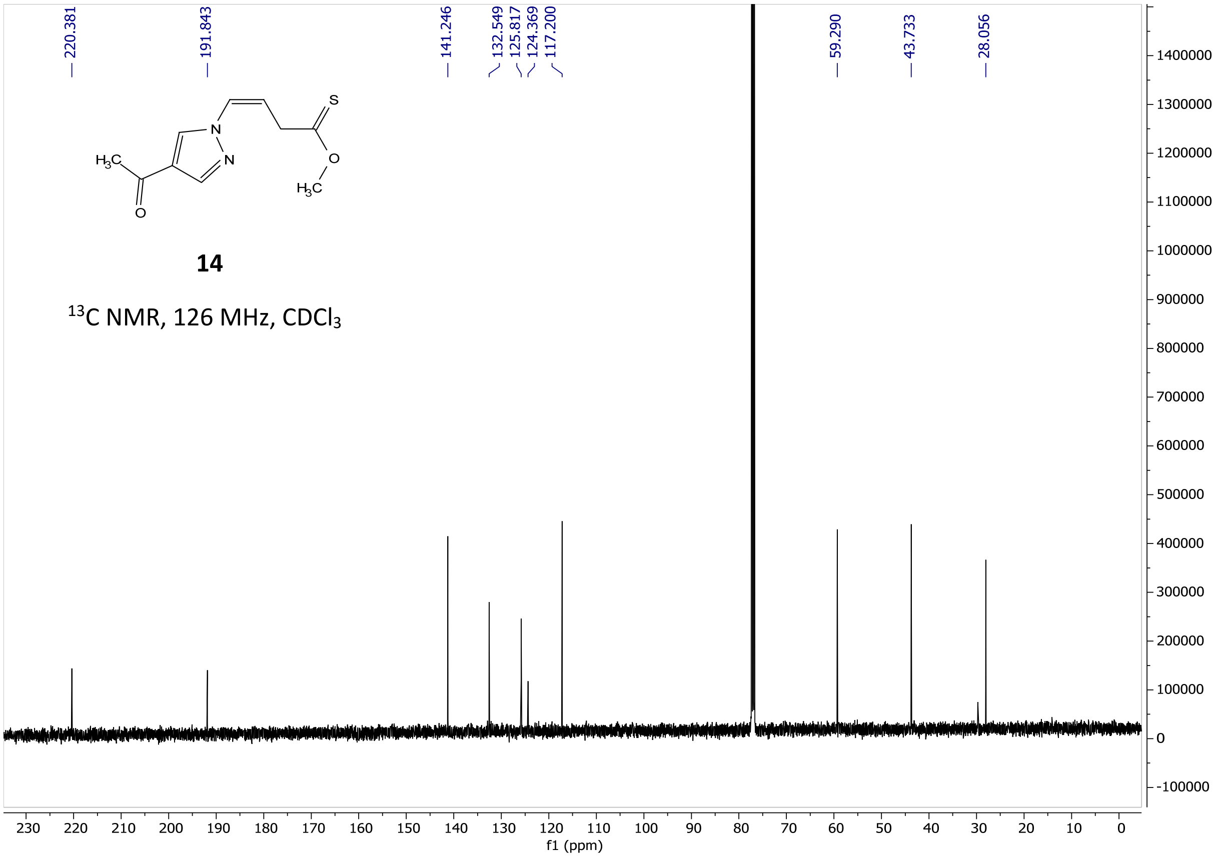
220.381
191.843
141.246
132.549
125.817
124.369
117.200
59.290
43.733
28.056
14
^{13}C NMR, 126 MHz, $CDCl_3$
f1 (ppm)

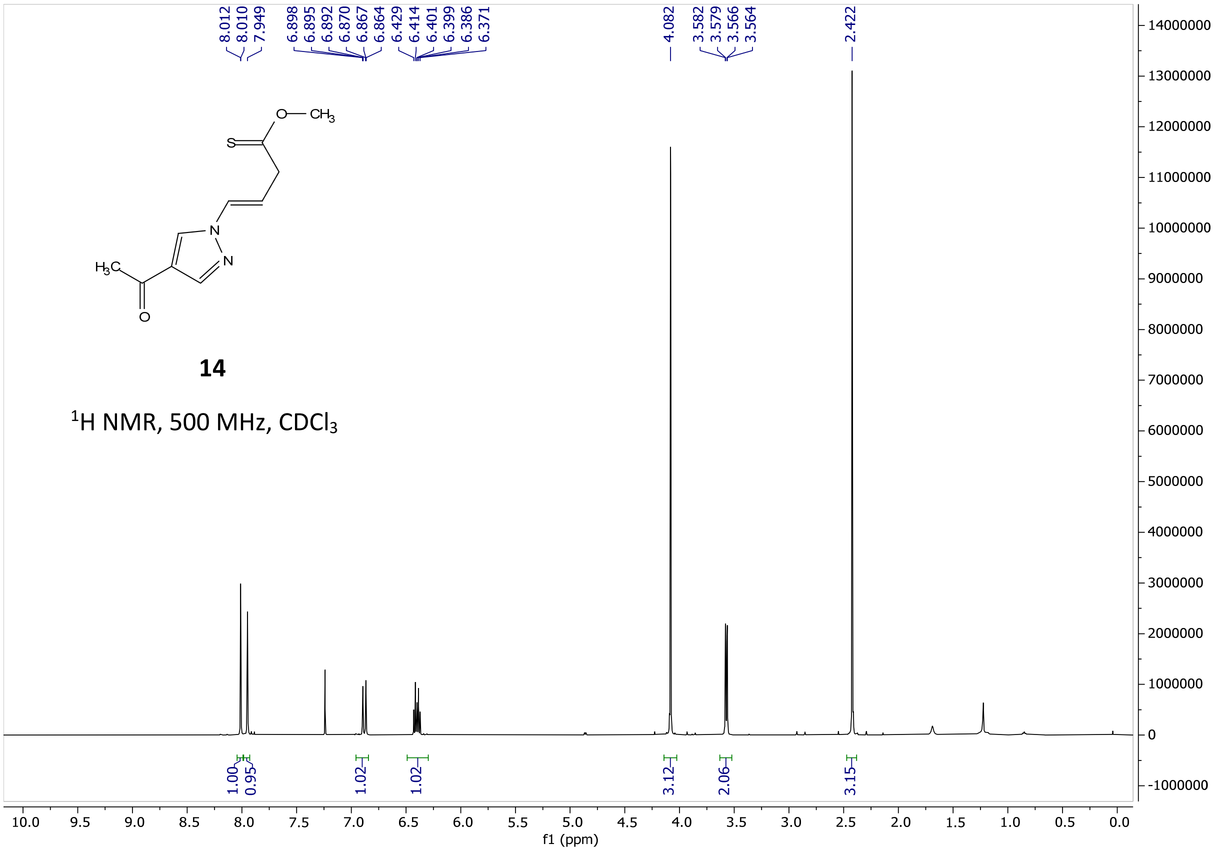
8.012
8.010
7.949
6.898
6.895
6.892
6.870
6.867
6.864
6.429
6.414
6.401
6.399
6.386
6.371
4.082
3.582
3.579
3.566
3.564
2.422
O—CH3
S
N
N
H3C
O
14
1H NMR, 500 MHz, CDCl3
1.00
0.95
1.02
1.02
3.12
2.06
3.15
f1 (ppm)

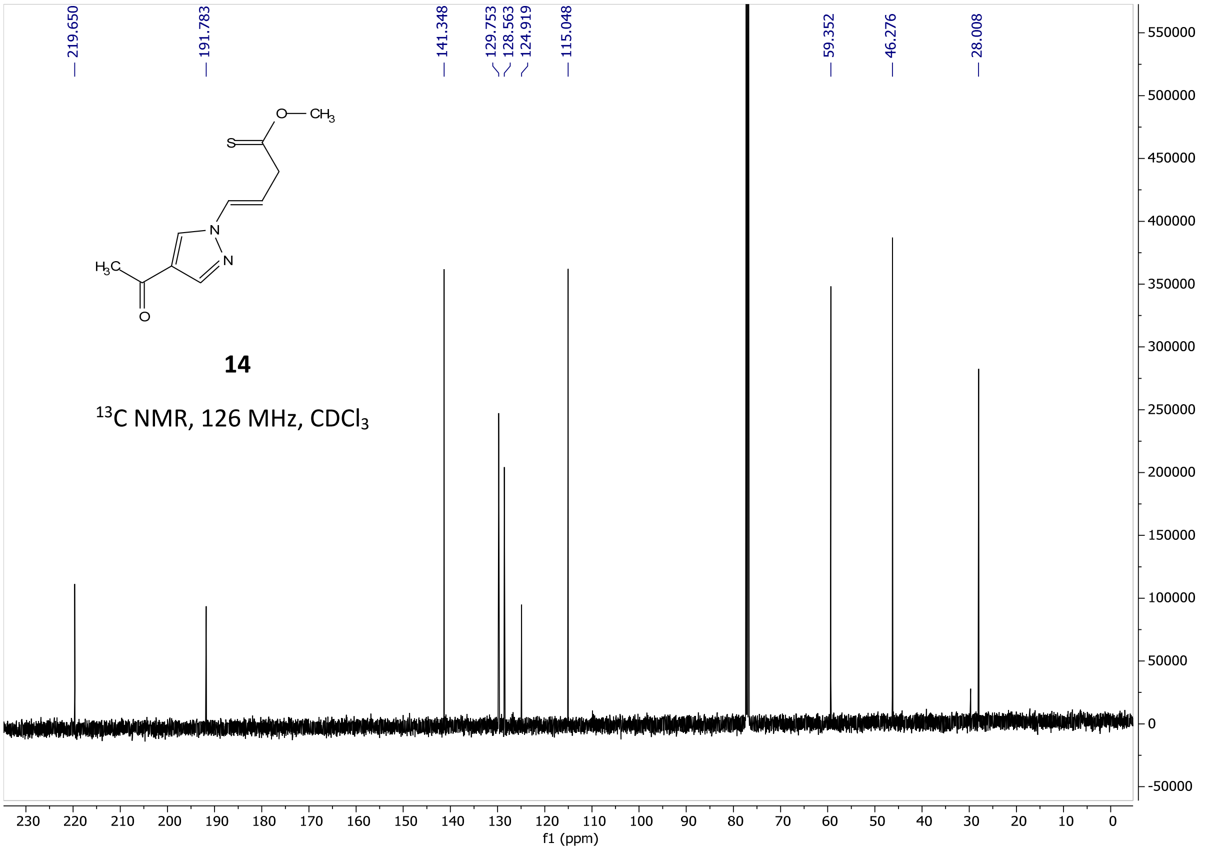
219.650
191.783
141.348
129.753
128.563
124.919
115.048
59.352
46.276
28.008
O—CH3
S
N
N
H3C
O
14
13C NMR, 126 MHz, CDCl3
f1 (ppm)

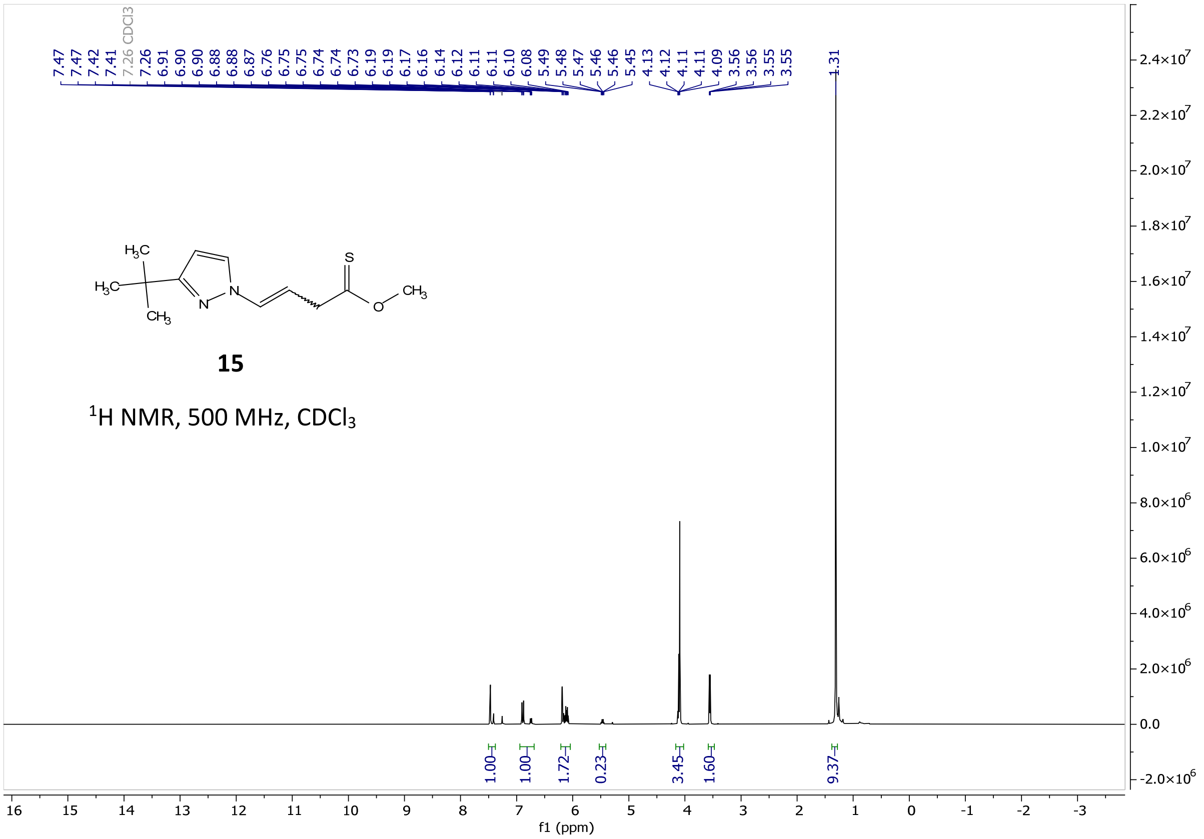
15
1H NMR, 500 MHz, CDCl3
f1 (ppm)

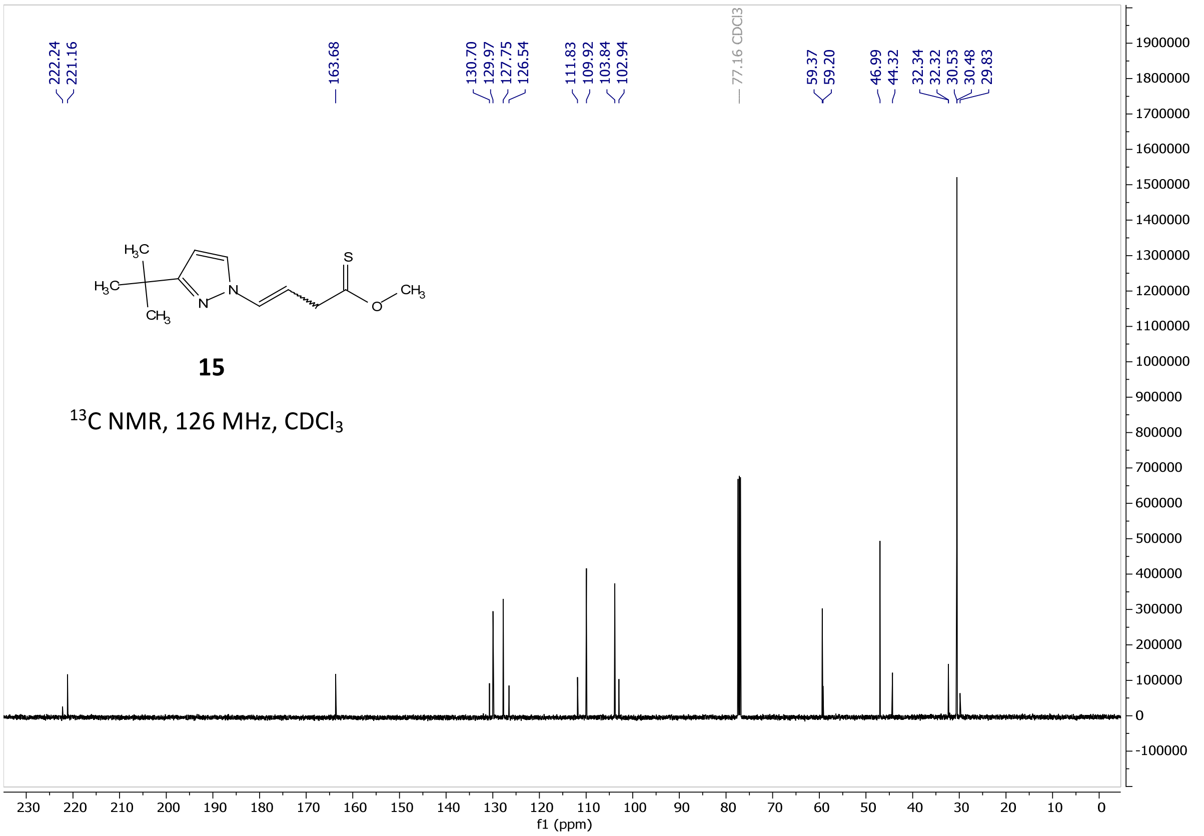
15
13C NMR, 126 MHz, CDCl3
f1 (ppm)

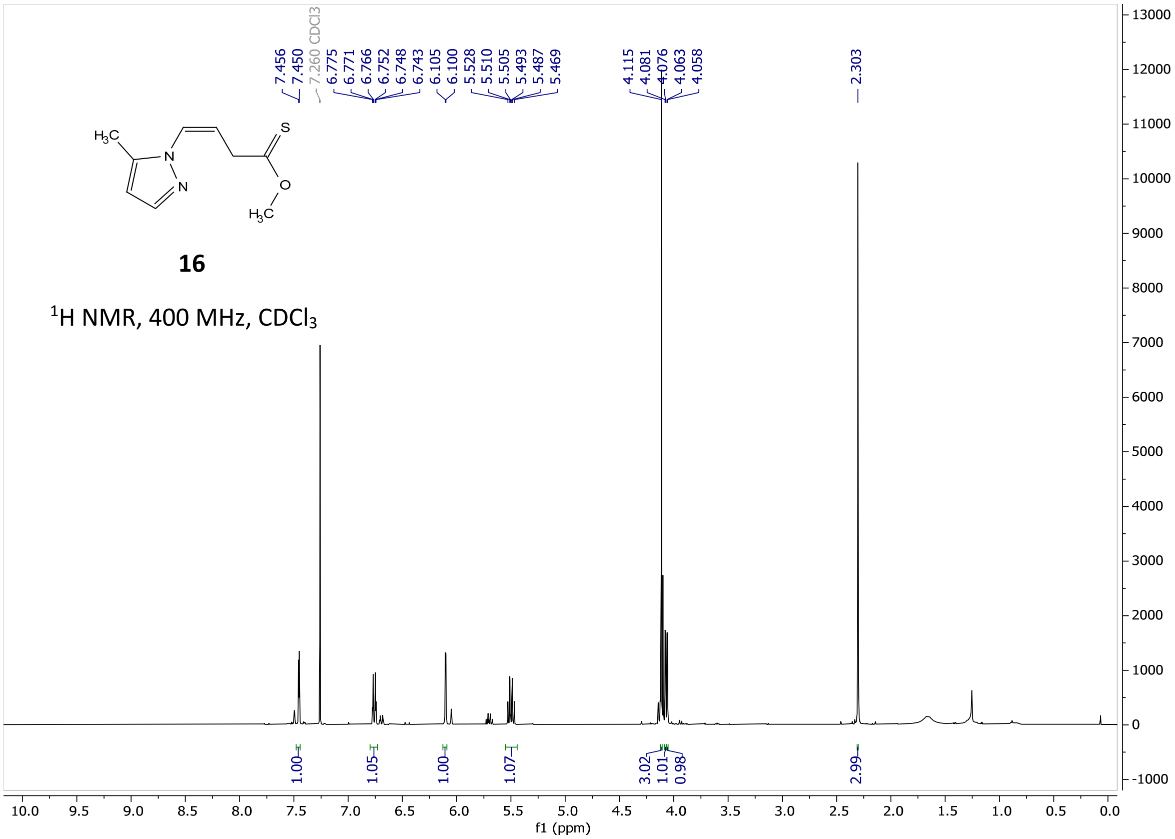
7.456
7.450
7.260 CDCl3
6.775
6.771
6.766
6.752
6.748
6.743
6.105
6.100
5.528
5.510
5.505
5.493
5.487
5.469
4.115
4.081
4.076
4.063
4.058
2.303
16
^{1}H NMR, 400 MHz, CDCl$_3$
1.00
1.05
1.00
1.07
3.02
1.01
0.98
2.99
f1 (ppm)

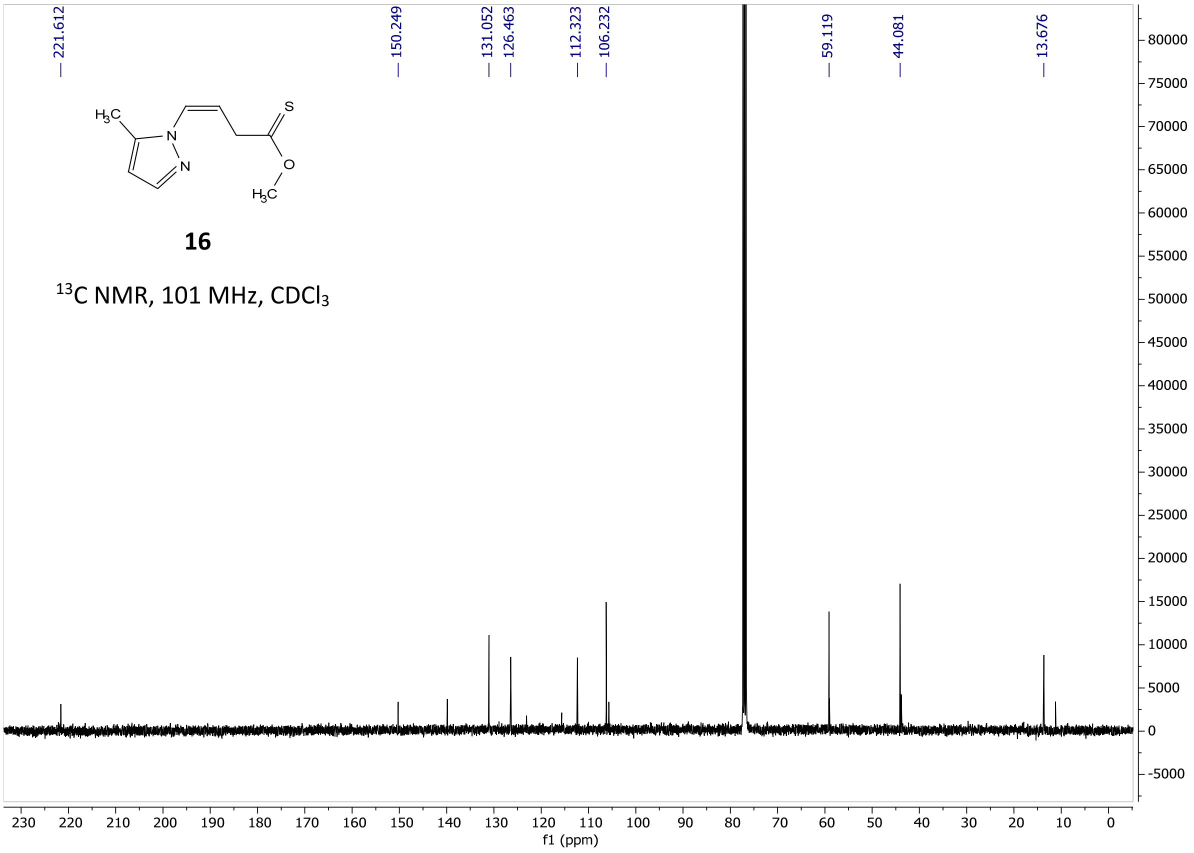
221.612
150.249
131.052
126.463
112.323
106.232
59.119
44.081
13.676
16
^{13}C NMR, 101 MHz, CDCl$_3$
f1 (ppm)

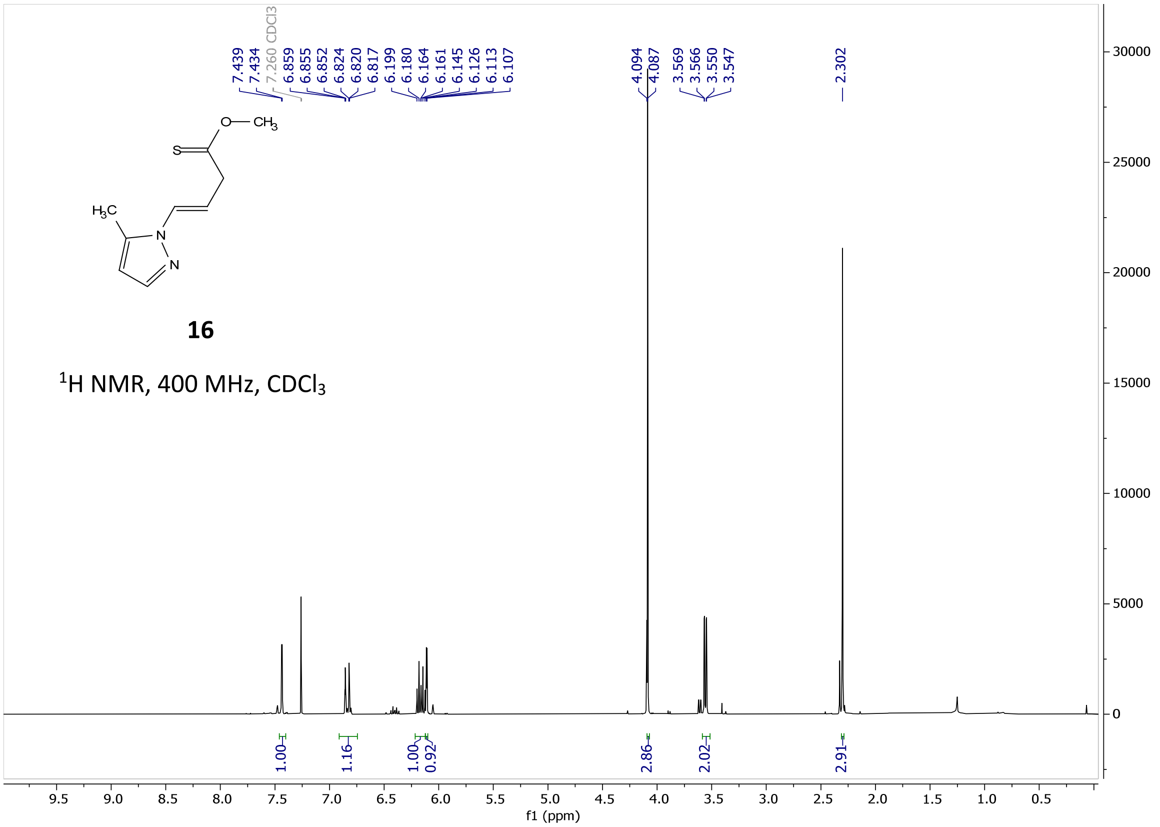

7.439
7.434
7.260 CDCl3
6.859
6.855
6.852
6.824
6.820
6.817
6.199
6.180
6.164
6.161
6.145
6.126
6.113
6.107
4.094
4.087
3.569
3.566
3.550
3.547
2.302
16
^{1}H NMR, 400 MHz, $CDCl_3$
1.00
1.16
1.00
0.92
2.86
2.02
2.91
f1 (ppm)

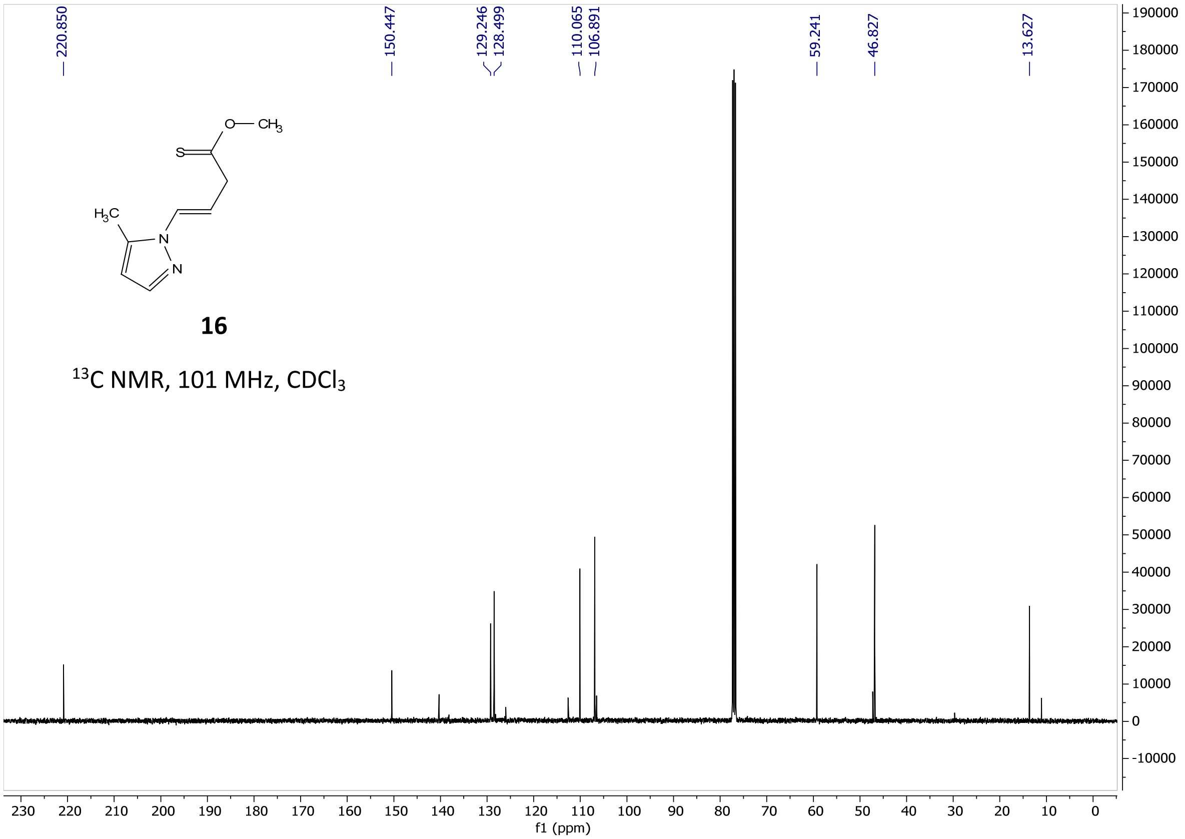

220.850
150.447
129.246
128.499
110.065
106.891
59.241
46.827
13.627
16
^{13}C NMR, 101 MHz, $CDCl_3$
f1 (ppm)

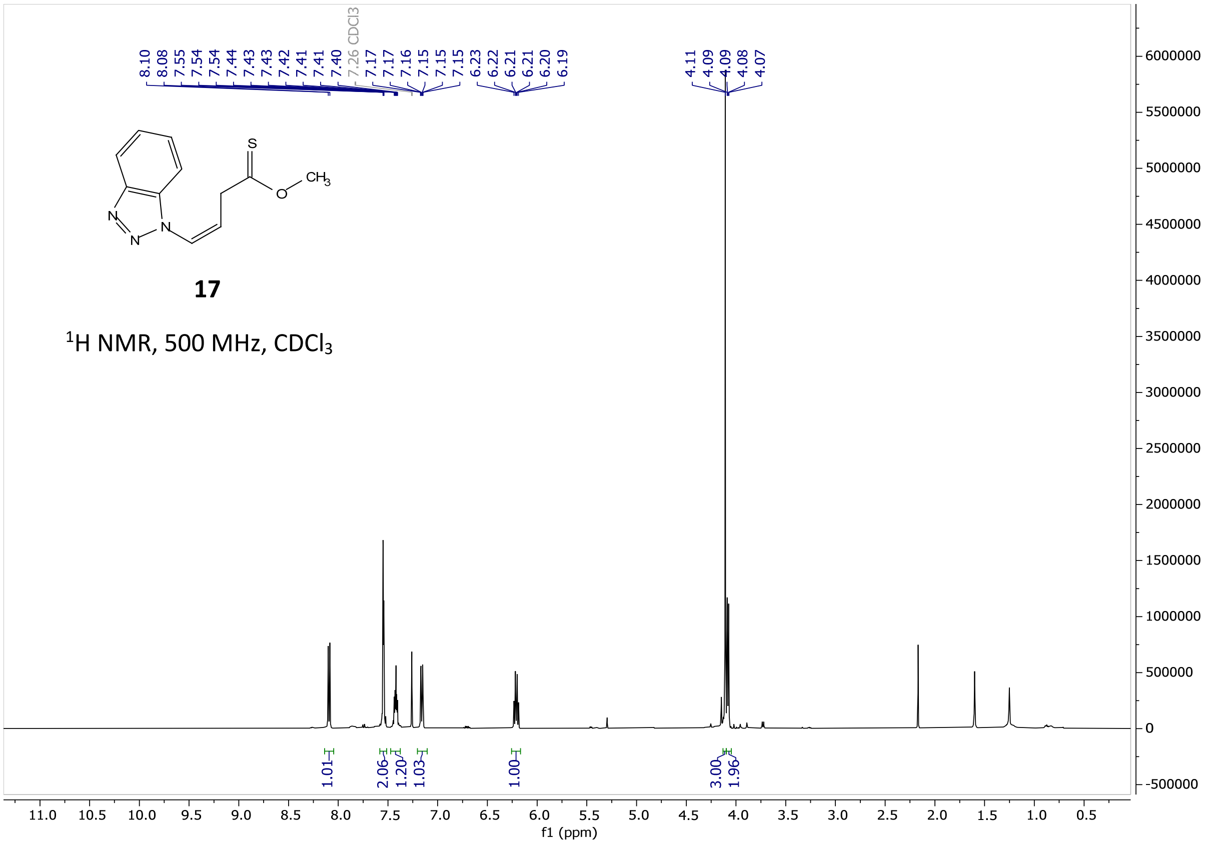
8.10 8.08 7.55 7.54 7.54 7.44 7.44 7.43 7.43 7.42 7.41 7.41 7.40 7.26 CDCl3 7.17 7.17 7.16 7.15 7.15 7.15 6.23 6.22 6.21 6.21 6.20 6.19
4.11 4.09 4.09 4.08 4.07
S
CH3
O
N
N
N
17
1H NMR, 500 MHz, CDCl3
1.01 2.06 1.20 1.03 1.00 3.00 1.96
f1 (ppm)

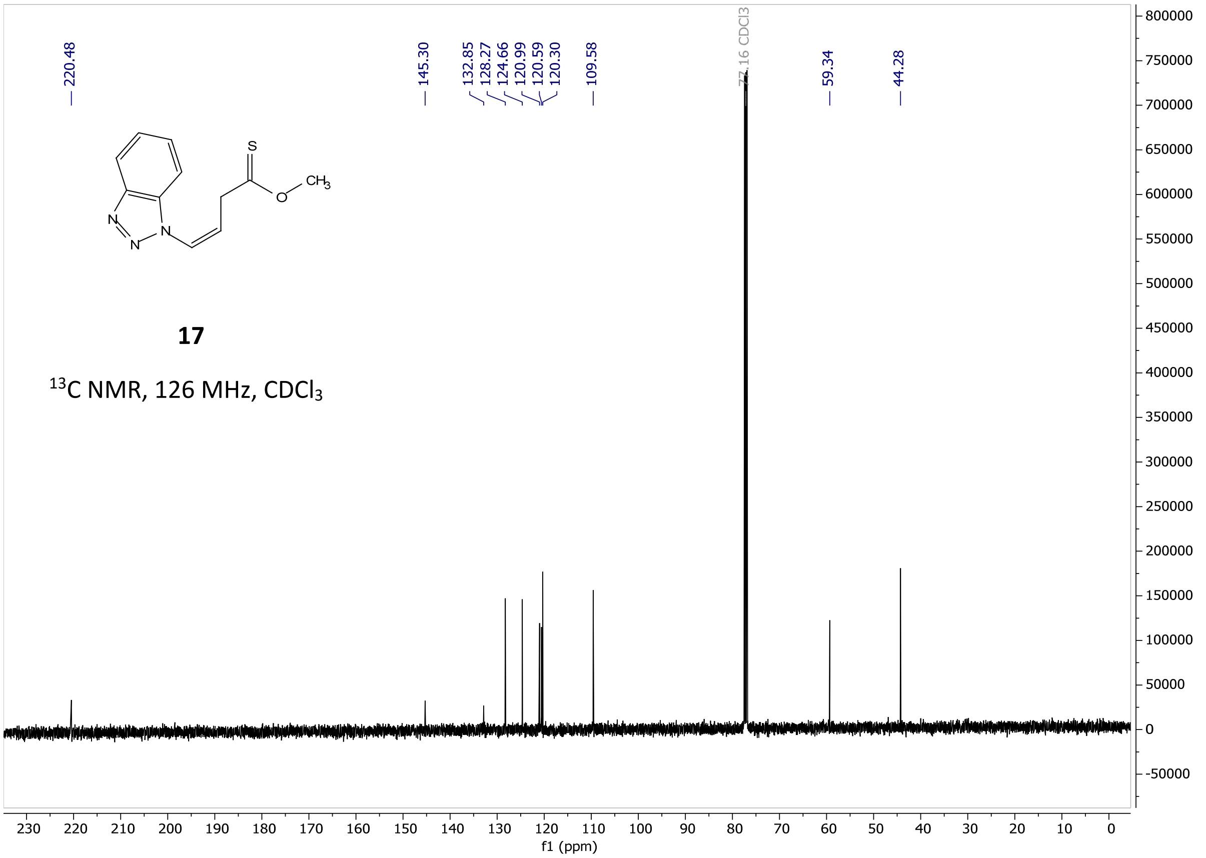
220.48
145.30
132.85 128.27 124.66 120.99 120.59 120.30
109.58
77.16 CDCl3
59.34
44.28
S
CH3
O
N
N
N
17
13C NMR, 126 MHz, CDCl3
f1 (ppm)

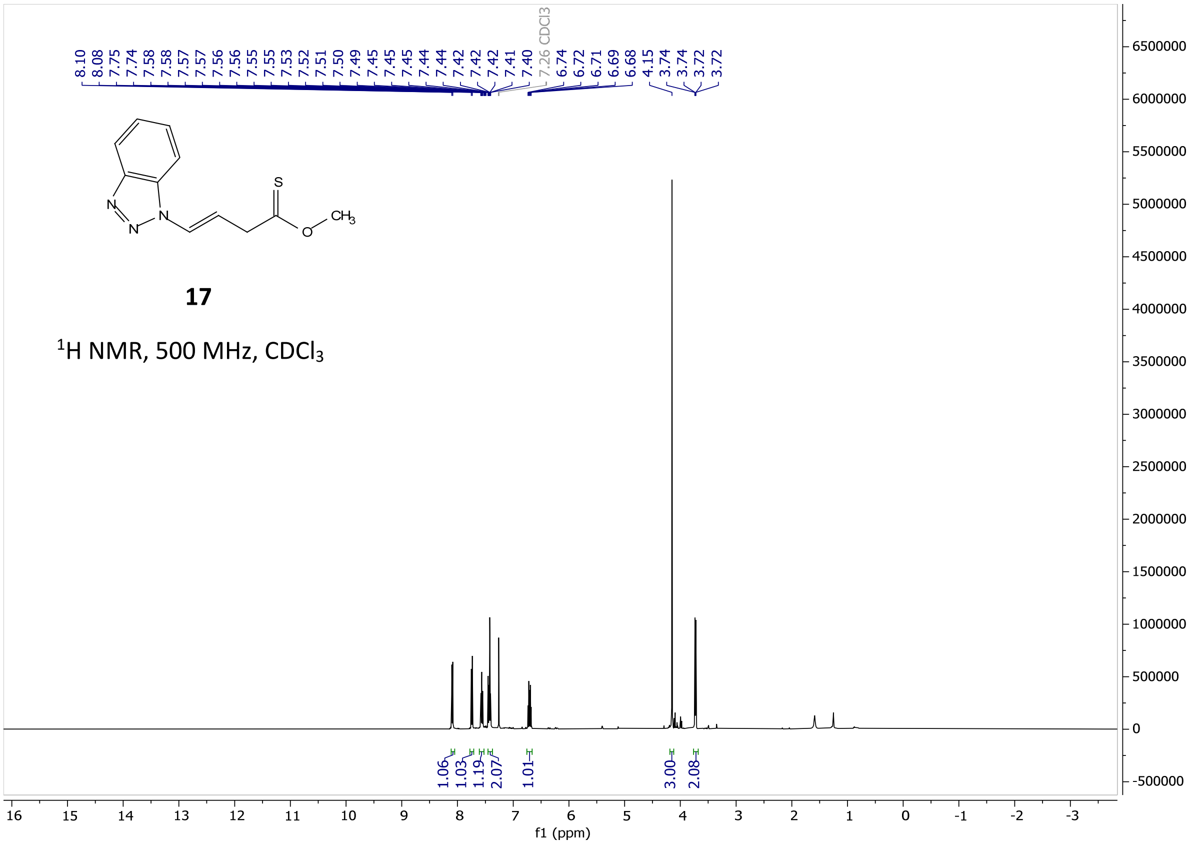
8.10 8.08 7.75 7.74 7.58 7.58 7.57 7.57 7.56 7.56 7.55 7.55 7.53 7.52 7.51 7.50 7.49 7.45 7.45 7.45 7.44 7.44 7.42 7.42 7.42 7.41 7.40
7.26 CDCl3
6.74 6.72 6.71 6.69 6.68 4.15 3.74 3.74 3.72 3.72
17
^{1}H NMR, 500 MHz, $CDCl_3$
1.06 1.03 1.19 2.07 1.01 3.00 2.08
f1 (ppm)

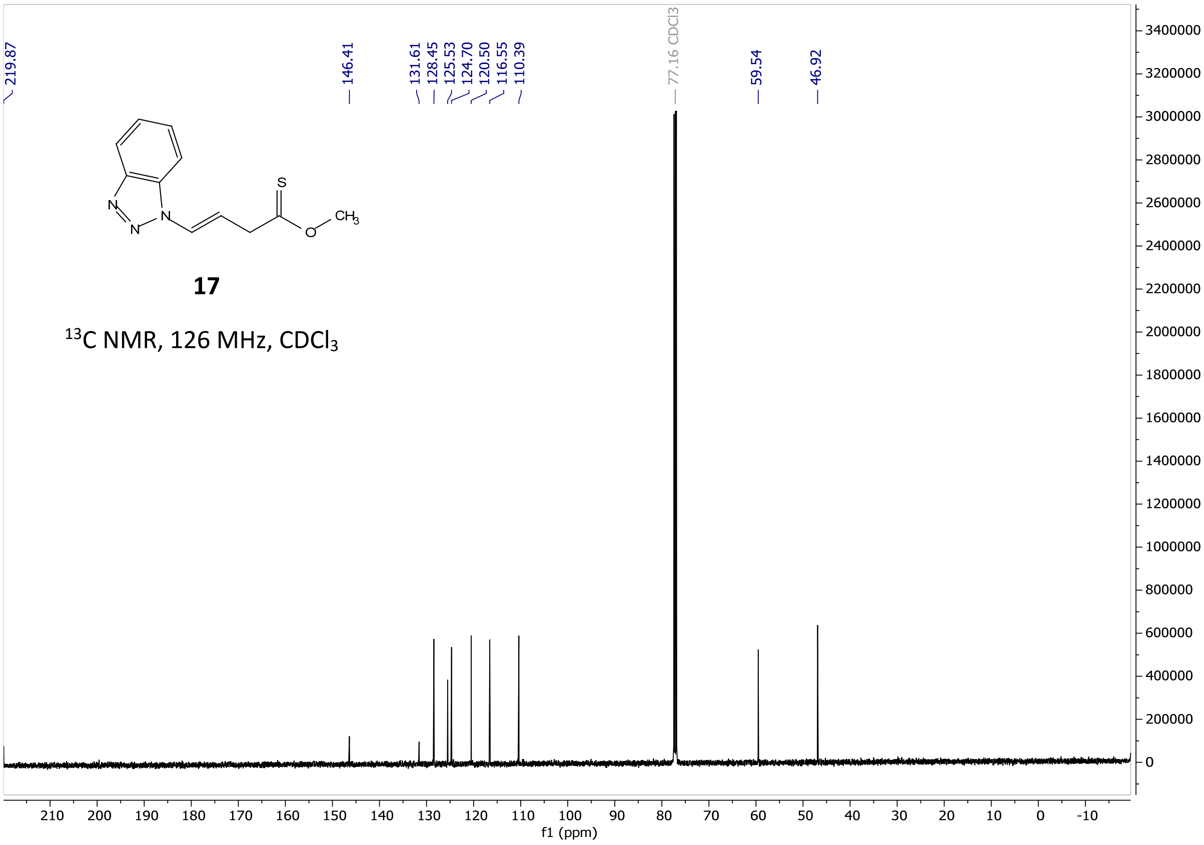
219.87
146.41
131.61 128.45 125.53 124.70 120.50 116.55 110.39
77.16 CDCl3
59.54
46.92
17
^{13}C NMR, 126 MHz, $CDCl_3$
f1 (ppm)

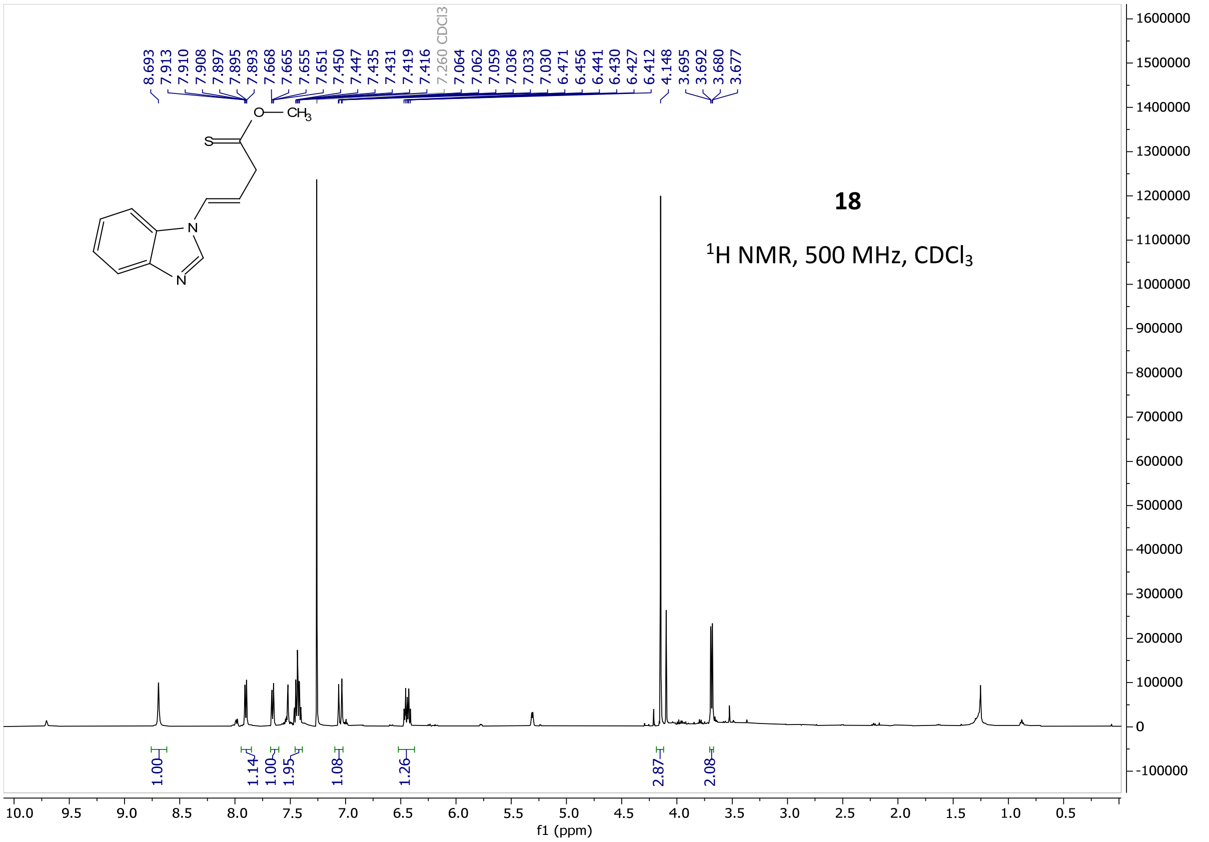
18
^{1}H NMR, 500 MHz, CDCl$_3$
8.693
7.913
7.910
7.908
7.897
7.895
7.893
7.668
7.665
7.655
7.651
7.450
7.447
7.435
7.431
7.419
7.416
7.260 CDCl3
7.064
7.062
7.059
7.036
7.033
7.030
6.471
6.456
6.441
6.430
6.427
6.412
4.148
3.695
3.692
3.680
3.677
1.00
1.14
1.00
1.95
1.08
1.26
2.87
2.08
f1 (ppm)

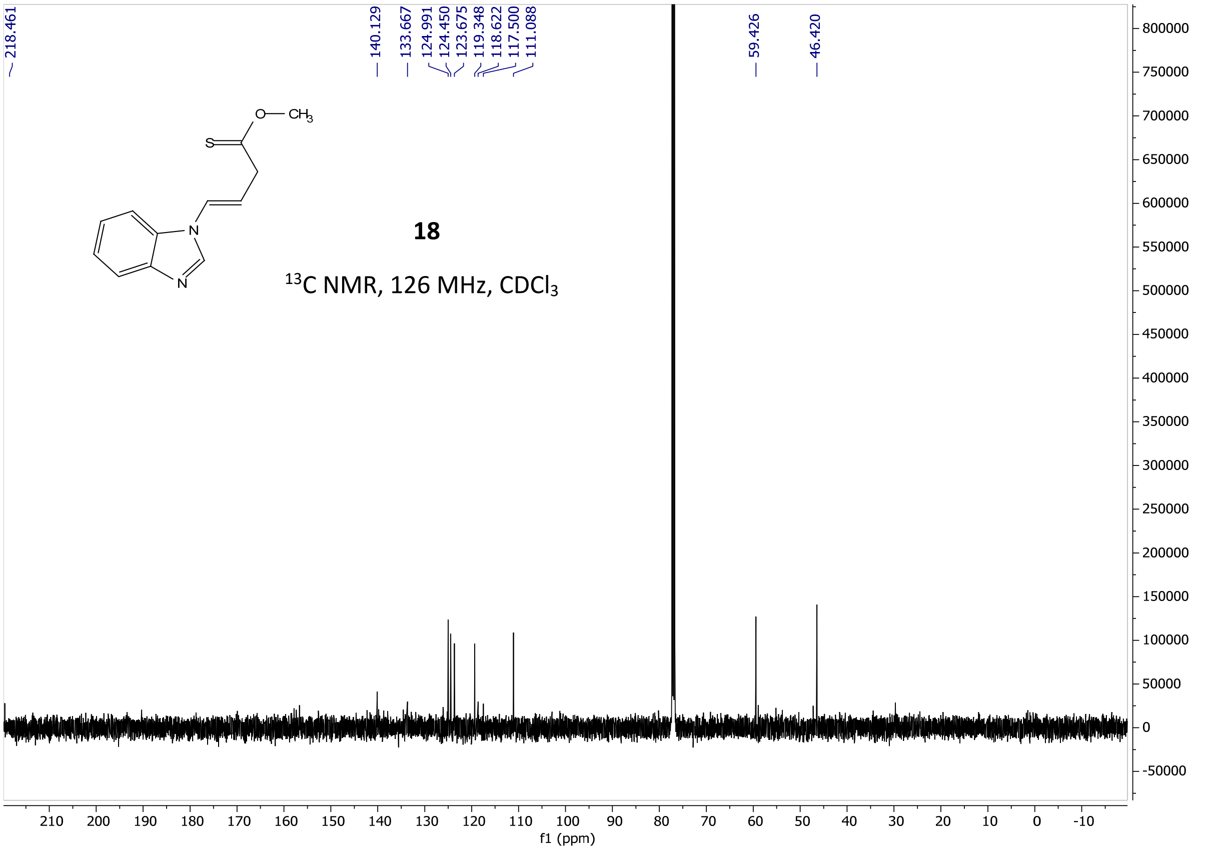
18
^{13}C NMR, 126 MHz, CDCl$_3$
218.461
140.129
133.667
124.991
124.450
123.675
119.348
118.622
117.500
111.088
59.426
46.420
f1 (ppm)

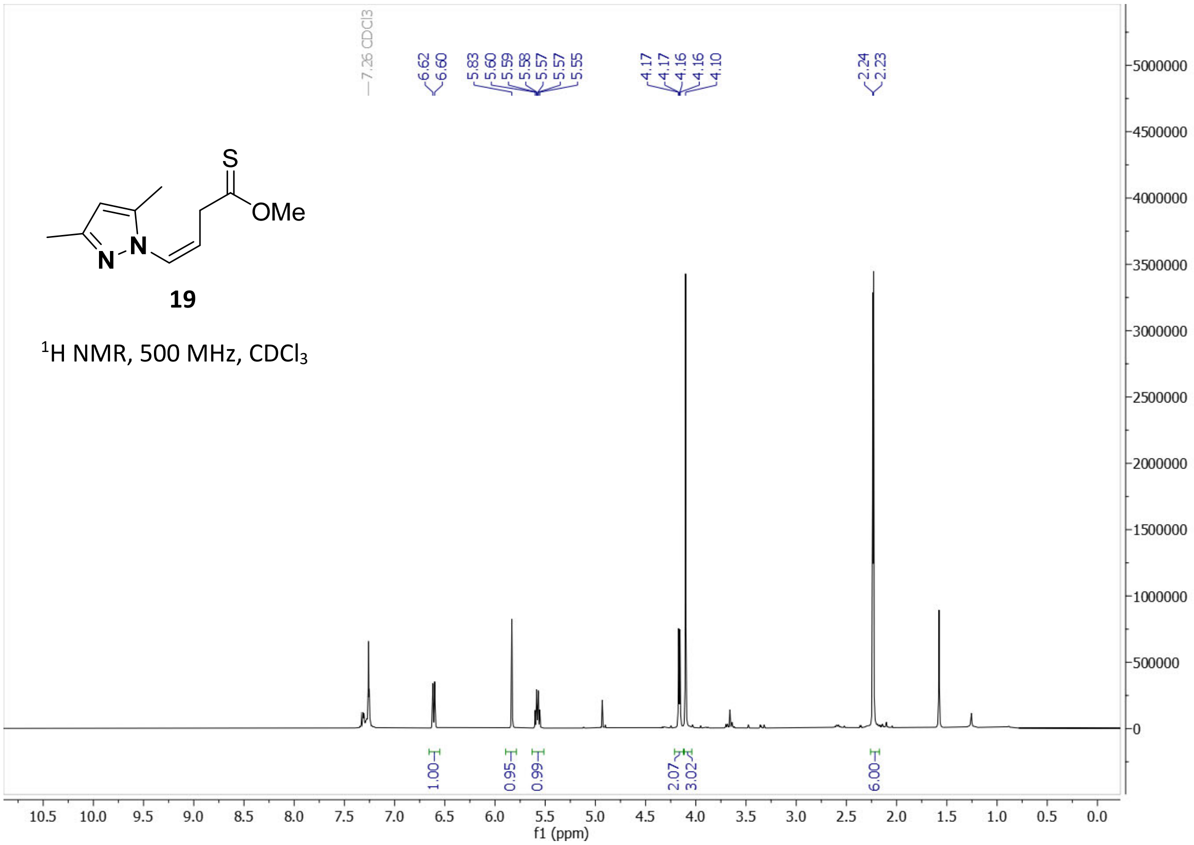

7.26 CDCl3
6.62
6.60
5.83
5.60
5.59
5.58
5.57
5.57
5.55
4.17
4.17
4.16
4.16
4.10
2.24
2.23
S
OMe
N
N
19
^{1}H NMR, 500 MHz, CDCl$_3$
1.00
0.95
0.99
2.07
3.02
6.00
f1 (ppm)

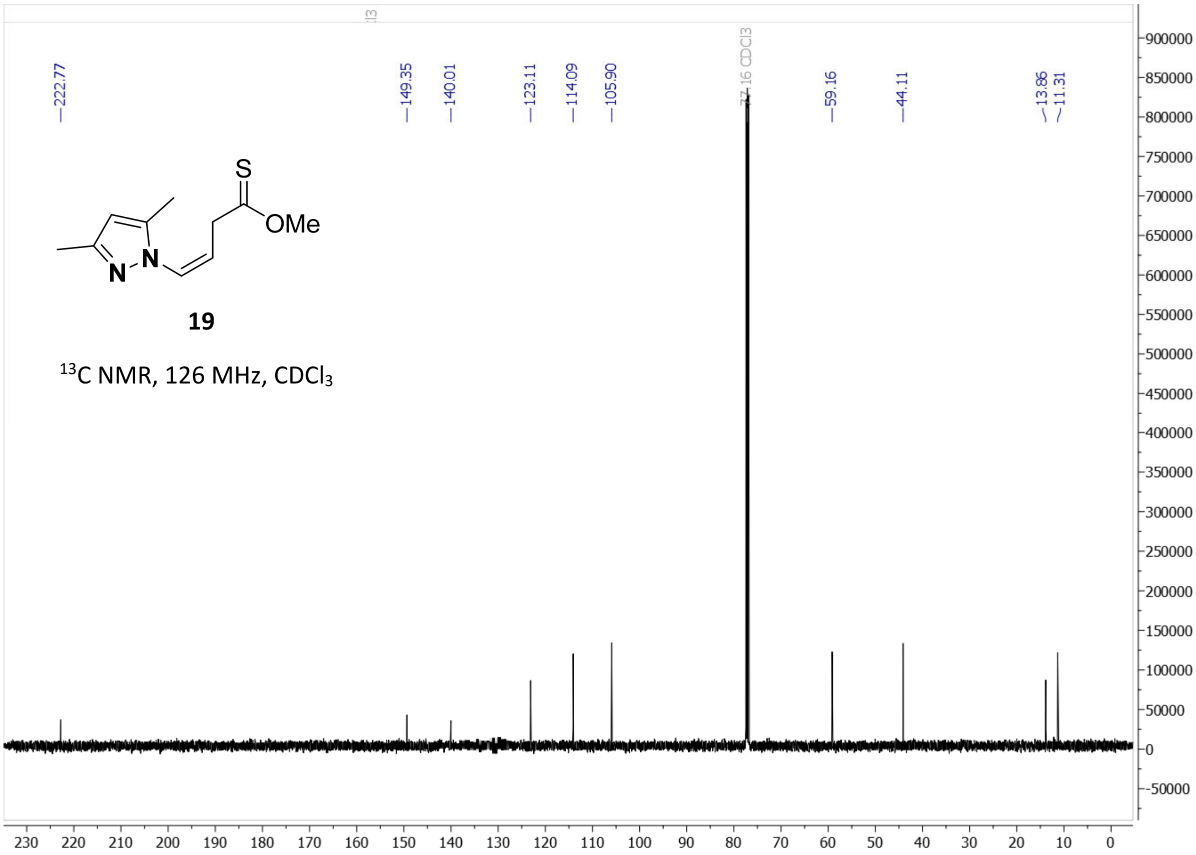

222.77
149.35
140.01
123.11
114.09
105.90
77.16 CDCl3
59.16
44.11
13.86
11.31
S
OMe
N
N
19
^{13}C NMR, 126 MHz, CDCl$_3$

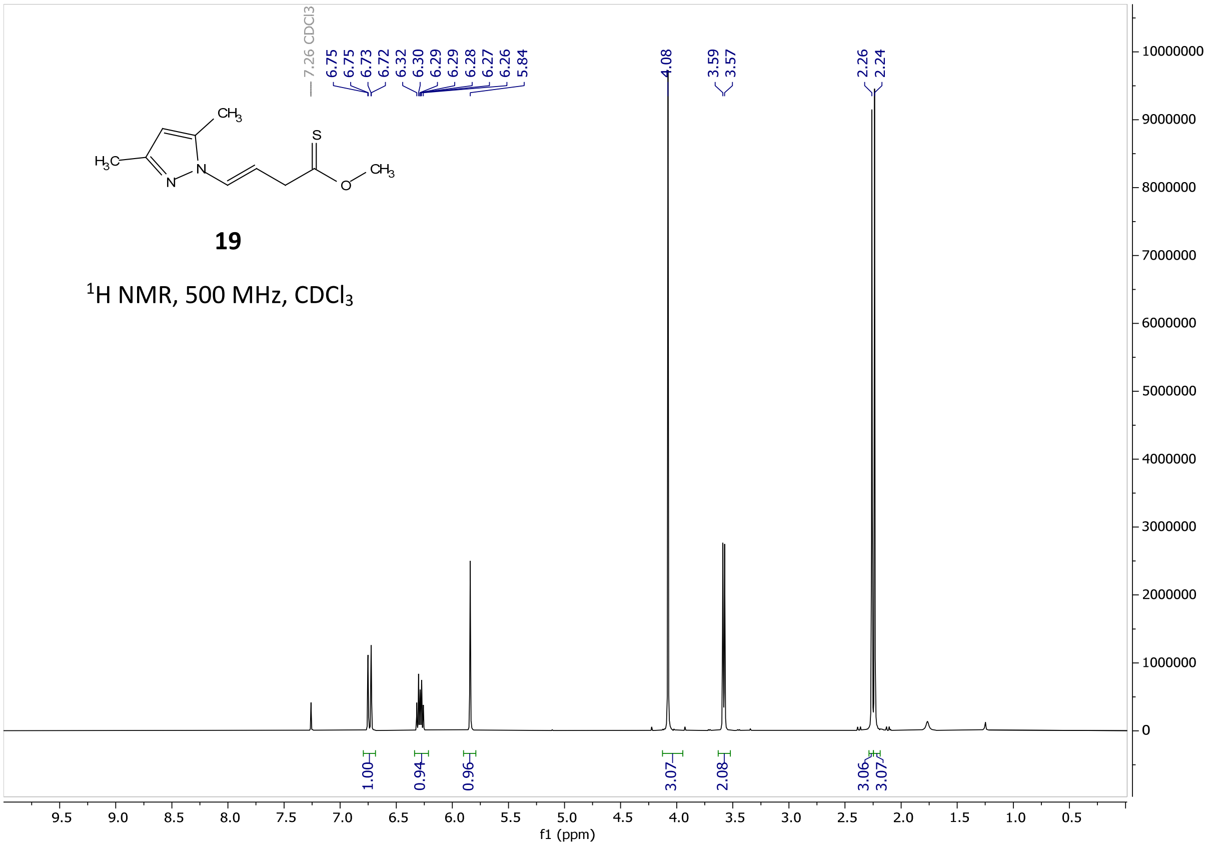

19
^{1}H NMR, 500 MHz, $CDCl_3$
7.26 CDCl3
6.75
6.75
6.73
6.72
6.32
6.30
6.29
6.29
6.28
6.27
6.26
5.84
4.08
3.59
3.57
2.26
2.24
1.00
0.94
0.96
3.07
2.08
3.06
3.07
f1 (ppm)

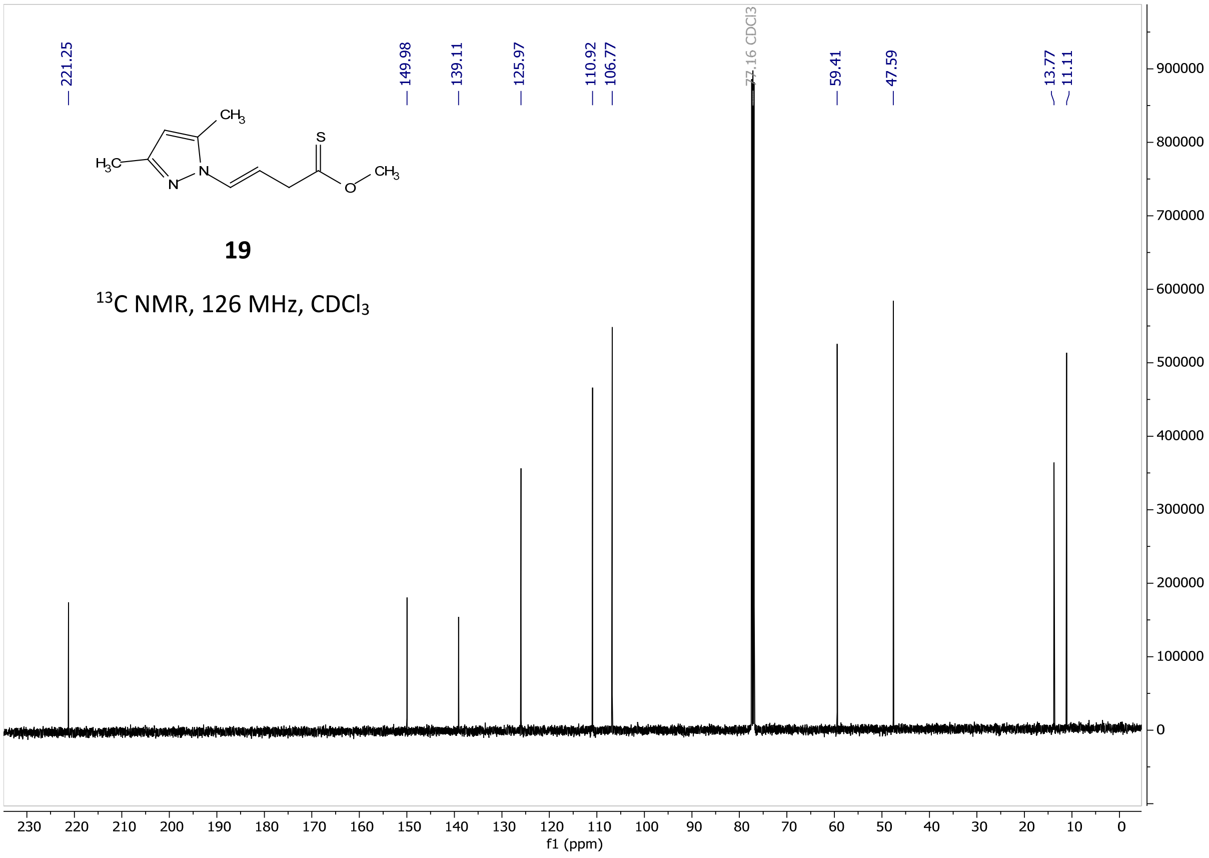

19
^{13}C NMR, 126 MHz, $CDCl_3$
221.25
149.98
139.11
125.97
110.92
106.77
77.16 CDCl3
59.41
47.59
13.77
11.11
f1 (ppm)

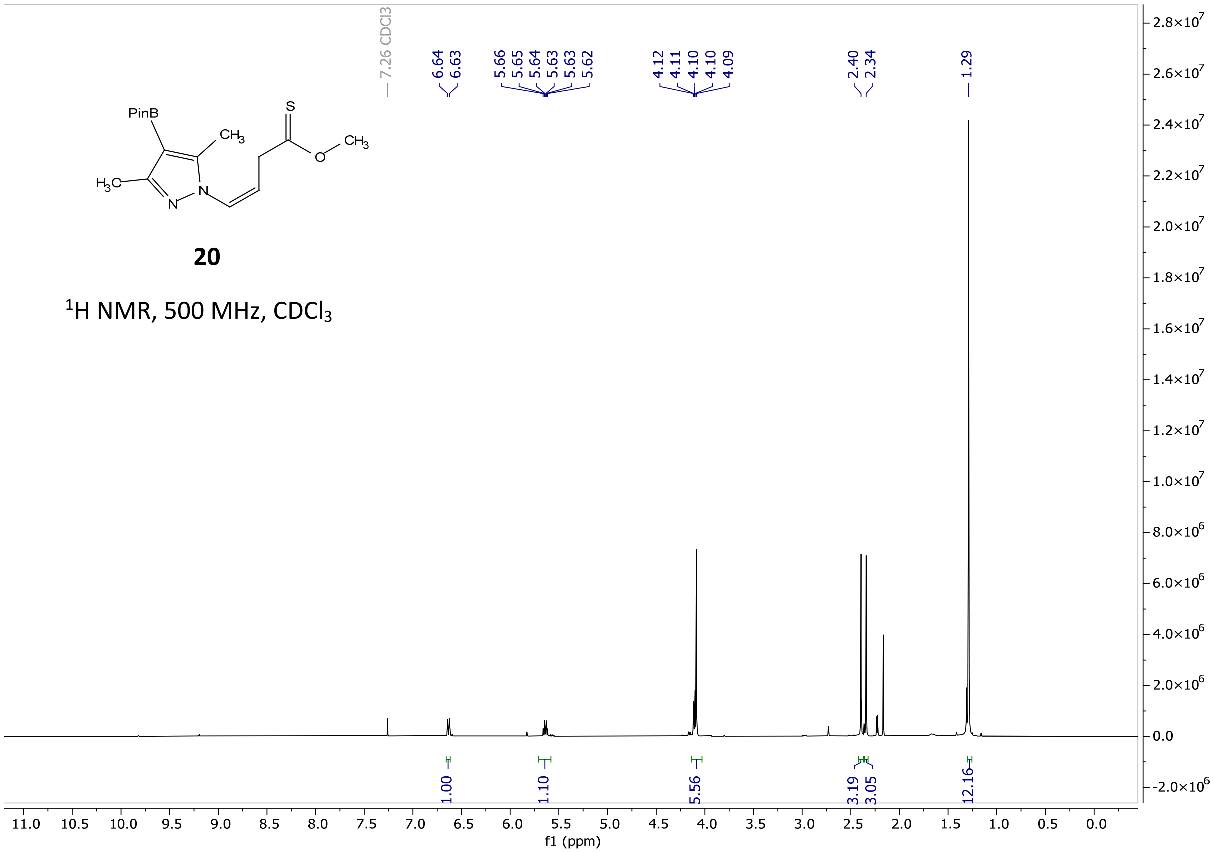
PinB
20
^{1}H NMR, 500 MHz, CDCl$_3$
7.26 CDCl3
6.64
6.63
5.66
5.65
5.64
5.63
5.63
5.62
4.12
4.11
4.10
4.10
4.09
2.40
2.34
1.29
1.00
1.10
5.56
3.19
3.05
12.16
f1 (ppm)

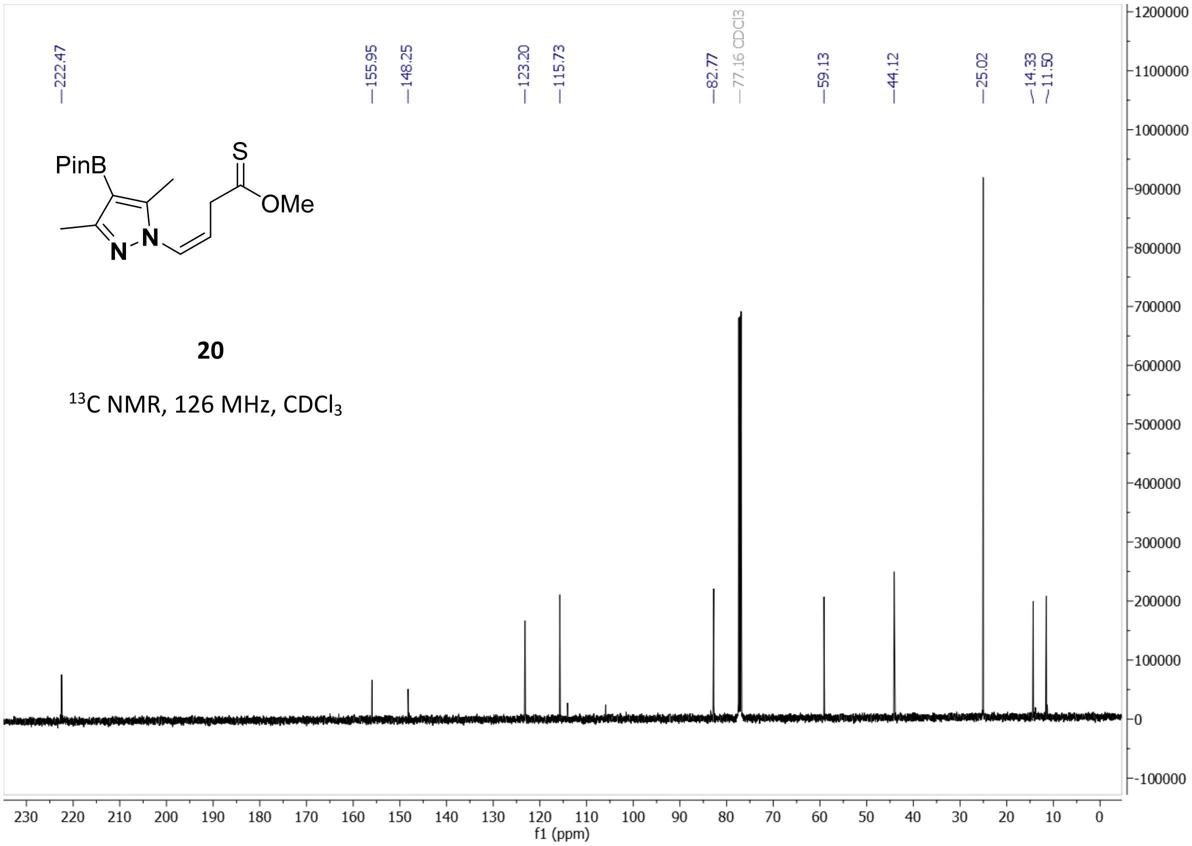
PinB
OMe
20
^{13}C NMR, 126 MHz, CDCl$_3$
222.47
155.95
148.25
123.20
115.73
82.77
77.16 CDCl3
59.13
44.12
25.02
14.33
11.50
f1 (ppm)

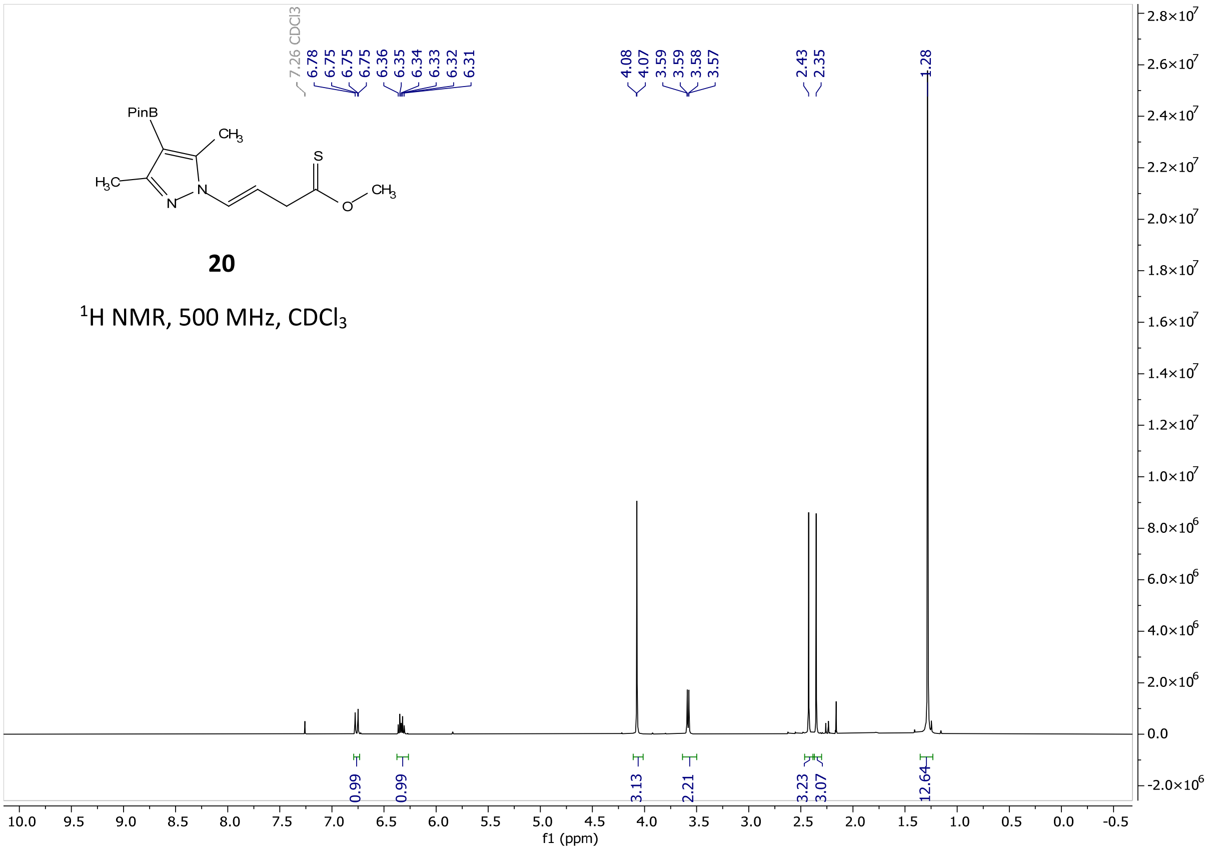
7.26 CDCl3
6.78
6.75
6.75
6.75
6.36
6.35
6.34
6.33
6.32
6.31
4.08
4.07
3.59
3.59
3.58
3.57
2.43
2.35
1.28
PinB
CH3
H3C
N
N
S
O
CH3
20
1H NMR, 500 MHz, CDCl3
0.99
0.99
3.13
2.21
3.23
3.07
12.64
f1 (ppm)

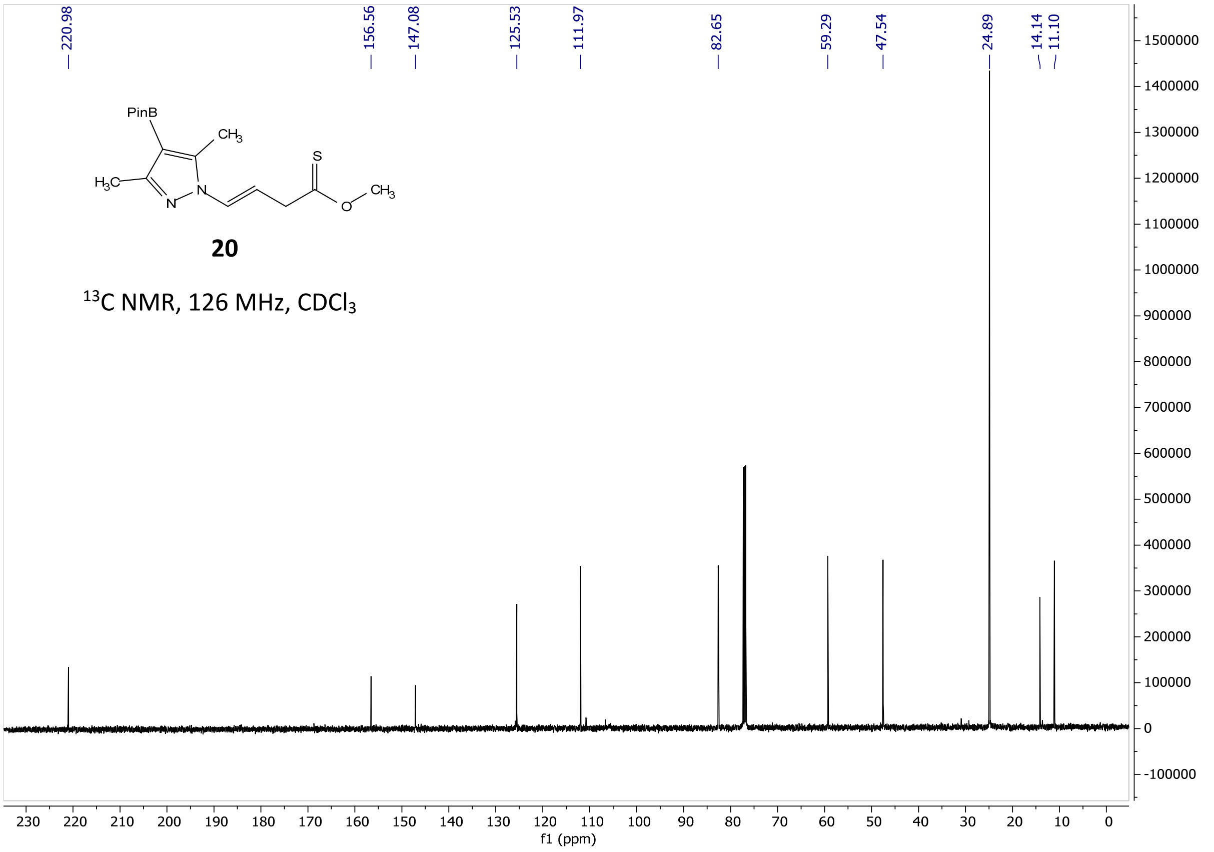
220.98
156.56
147.08
125.53
111.97
82.65
59.29
47.54
24.89
14.14
11.10
PinB
CH3
H3C
N
N
S
O
CH3
20
13C NMR, 126 MHz, CDCl3
f1 (ppm)

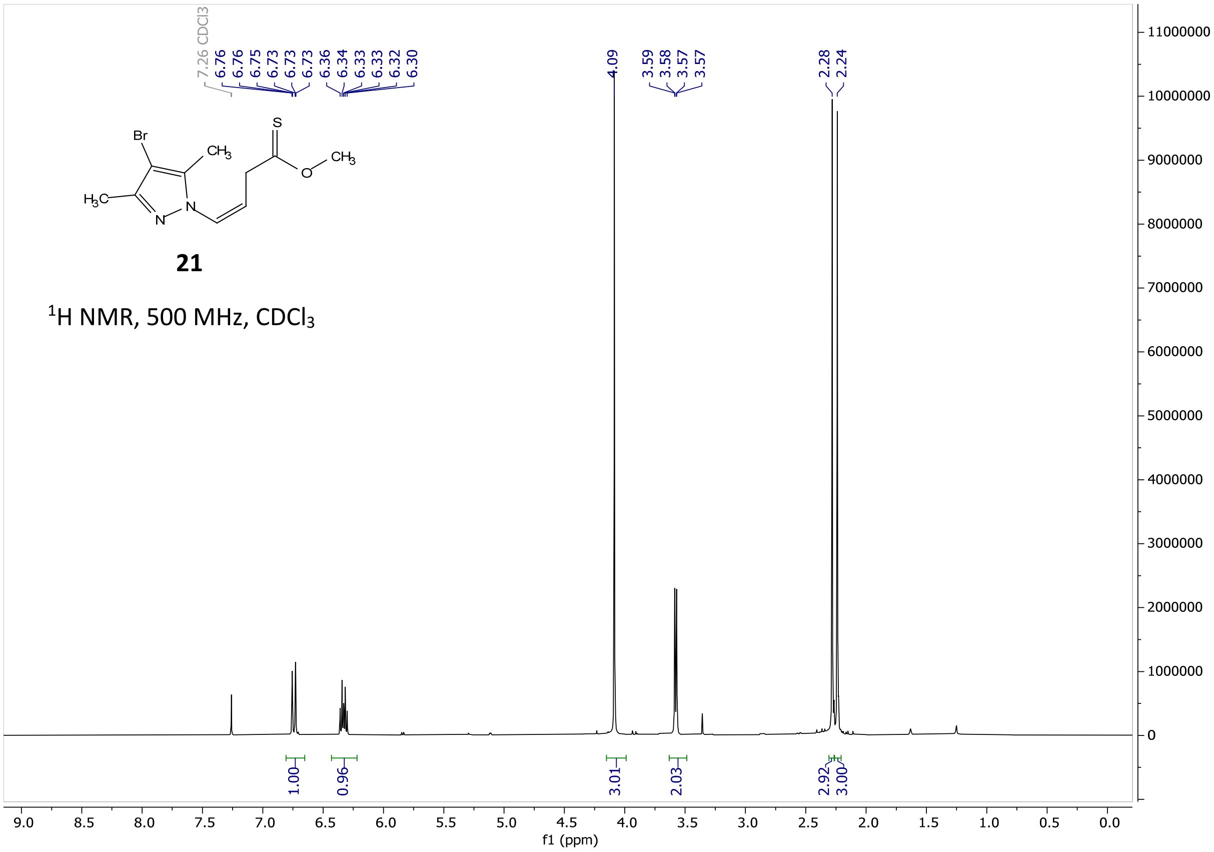
7.26 CDCl3
6.76
6.76
6.75
6.73
6.73
6.73
6.36
6.34
6.33
6.33
6.32
6.30
4.09
3.59
3.58
3.57
3.57
2.28
2.24
Br
CH3
S
O
CH3
H3C
N
N
21
1H NMR, 500 MHz, CDCl3
1.00
0.96
3.01
2.03
2.92
3.00
f1 (ppm)

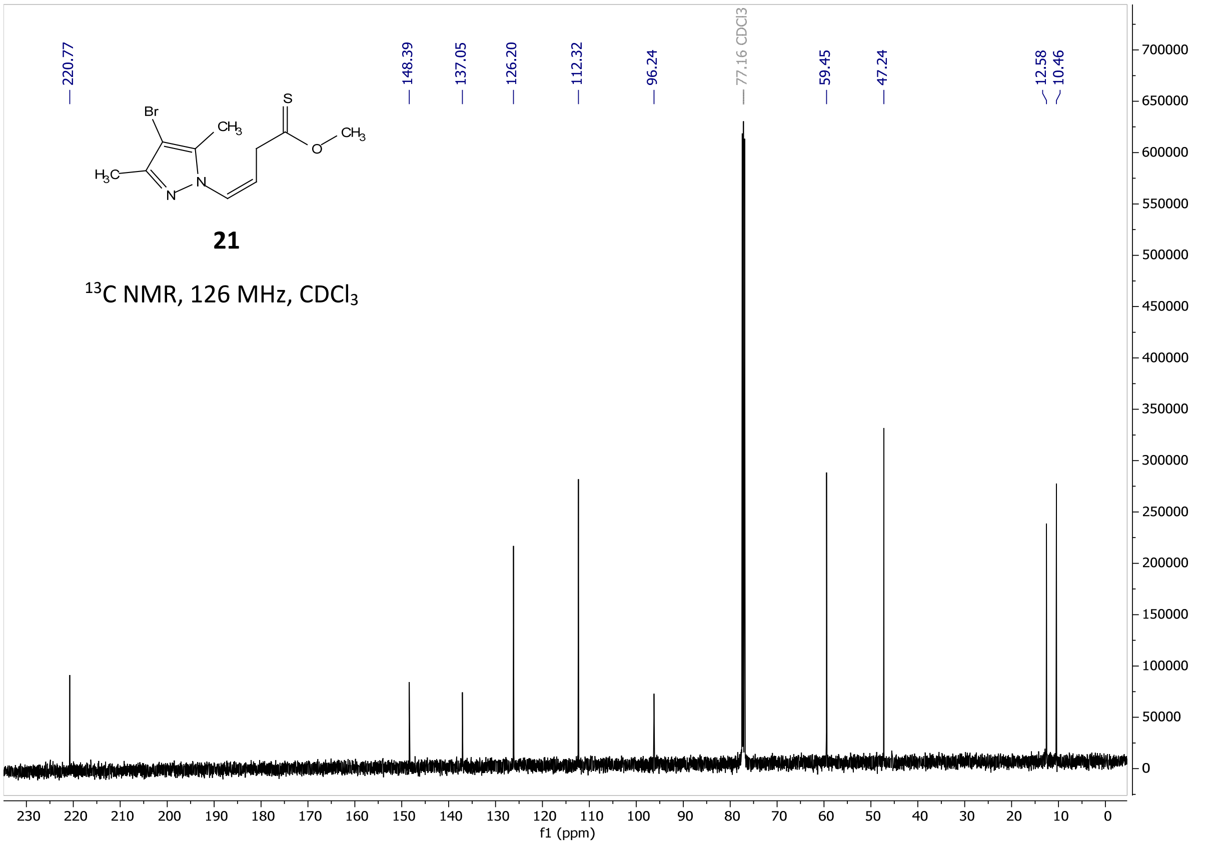
220.77
148.39
137.05
126.20
112.32
96.24
77.16 CDCl3
59.45
47.24
12.58
10.46
Br
CH3
S
O
CH3
H3C
N
N
21
13C NMR, 126 MHz, CDCl3
f1 (ppm)

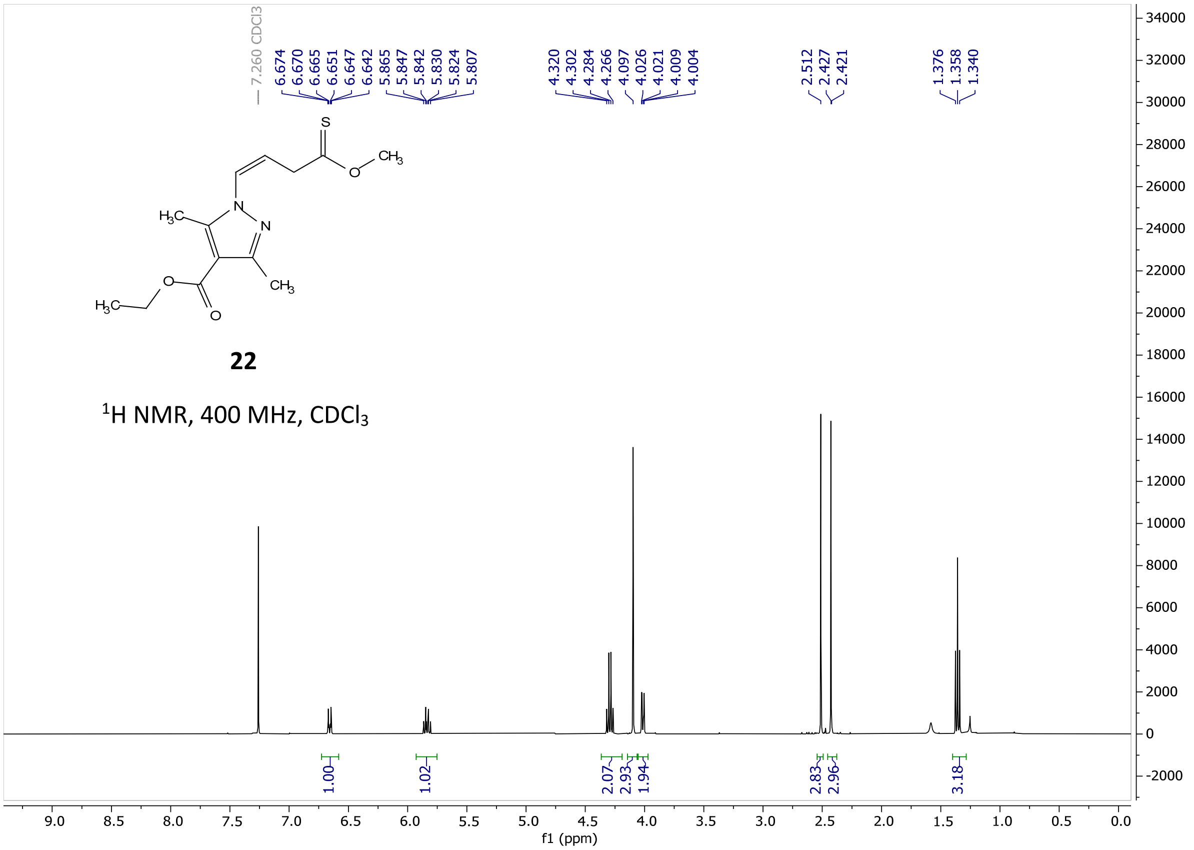

22

^{1}H NMR, 400 MHz, $CDCl_3$

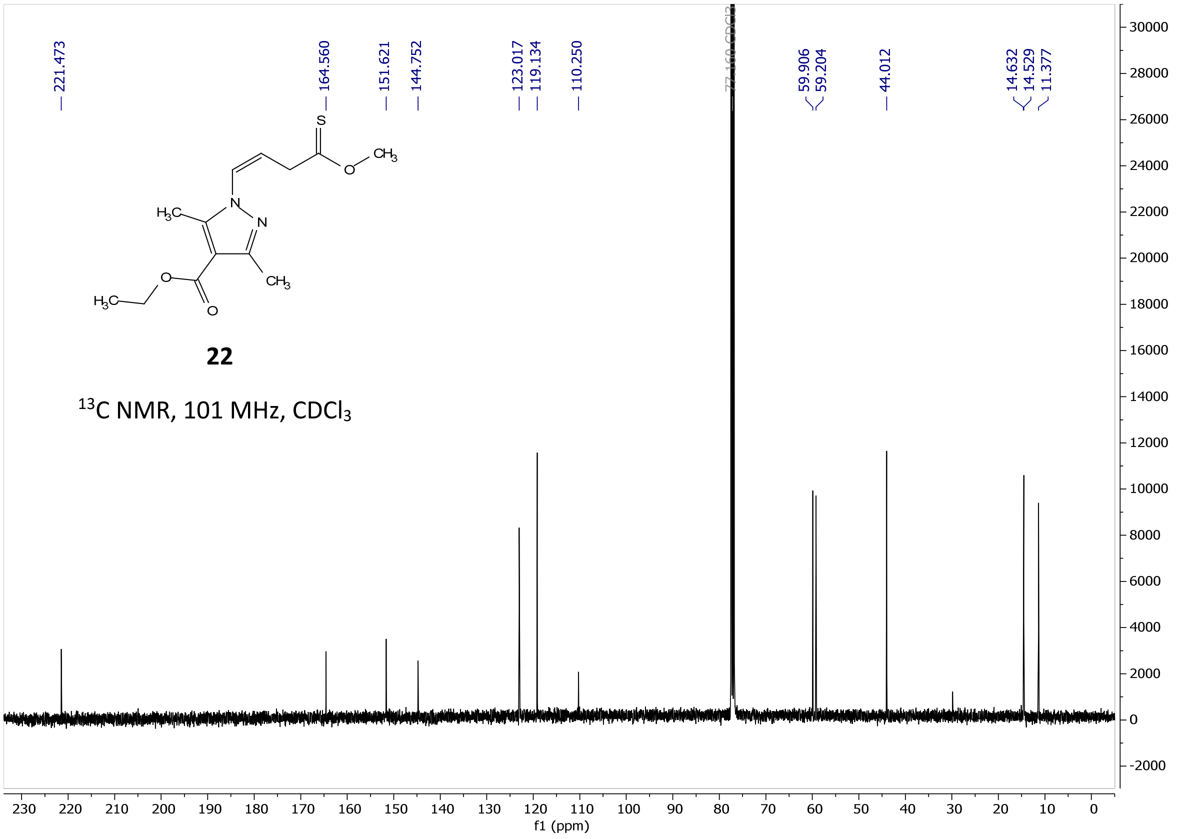

22

^{13}C NMR, 101 MHz, $CDCl_3$

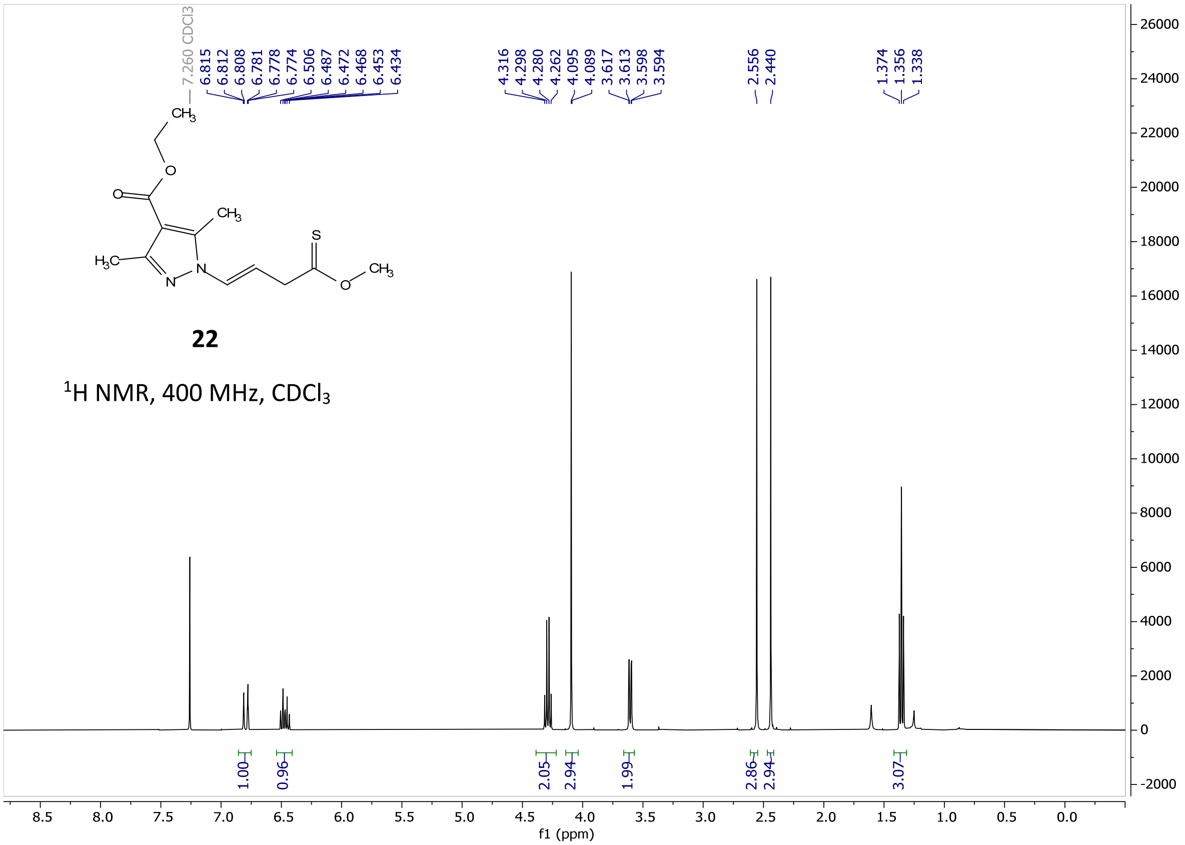
22
^{1}H NMR, 400 MHz, CDCl$_3$
7.260 CDCl3
6.815
6.812
6.808
6.781
6.778
6.774
6.506
6.487
6.472
6.468
6.453
6.434
4.316
4.298
4.280
4.262
4.095
4.089
3.617
3.613
3.598
3.594
2.556
2.440
1.374
1.356
1.338
1.00
0.96
2.05
2.94
1.99
2.86
2.94
3.07
f1 (ppm)

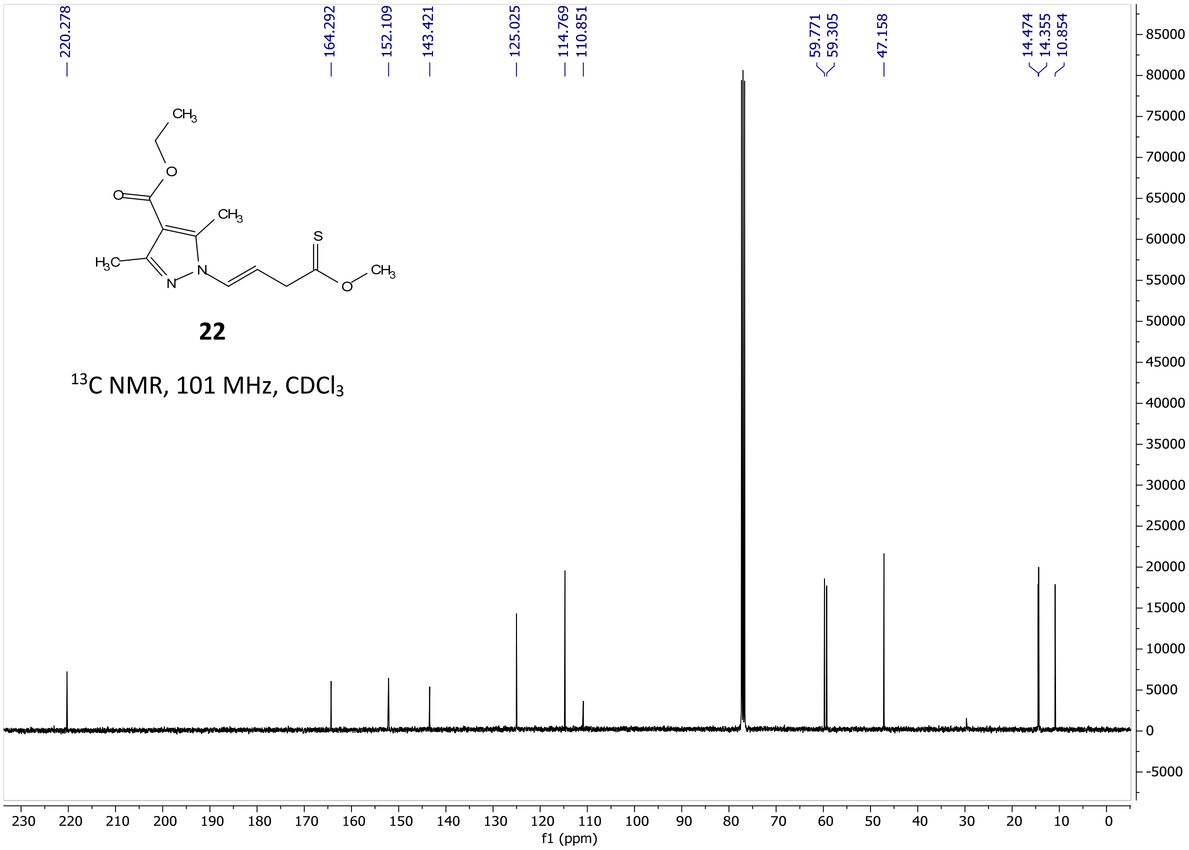
22
^{13}C NMR, 101 MHz, CDCl$_3$
220.278
164.292
152.109
143.421
125.025
114.769
110.851
59.771
59.305
47.158
14.474
14.355
10.854
f1 (ppm)

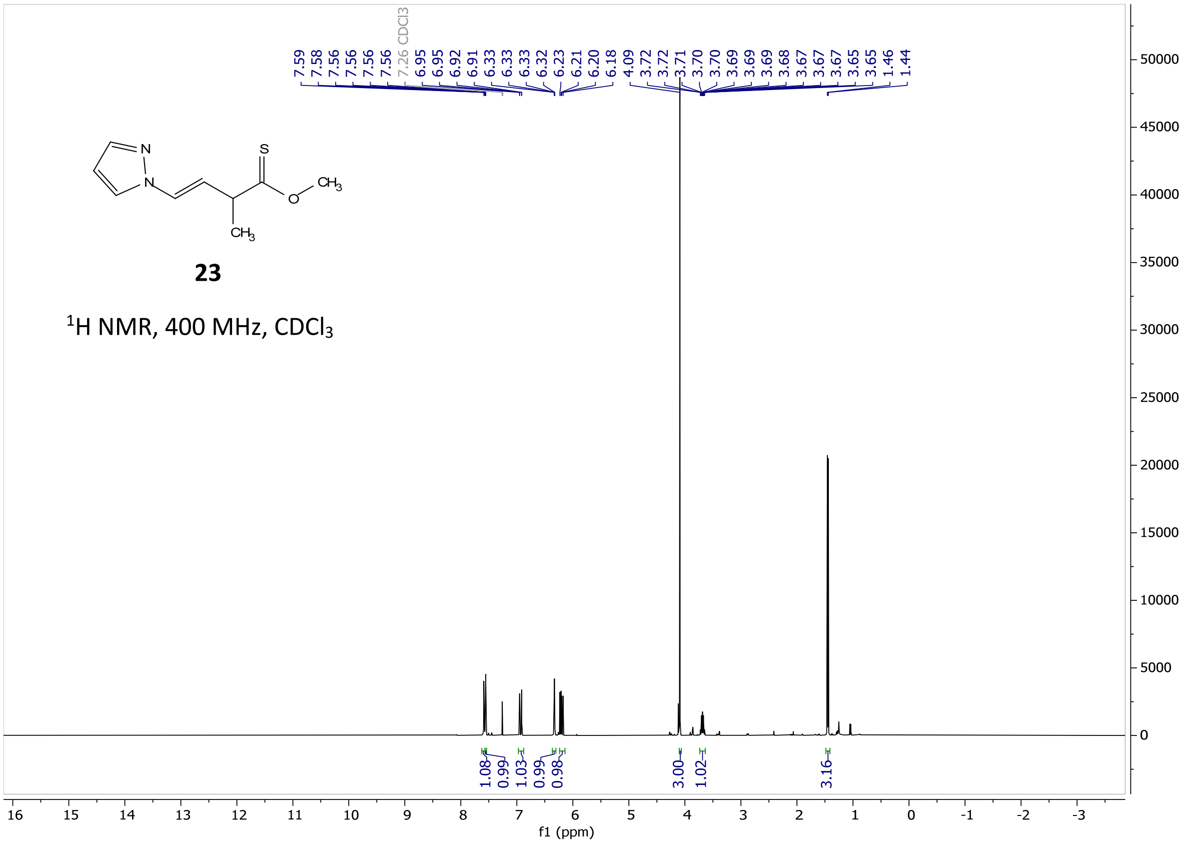

23

^{1}H NMR, 400 MHz, $CDCl_3$

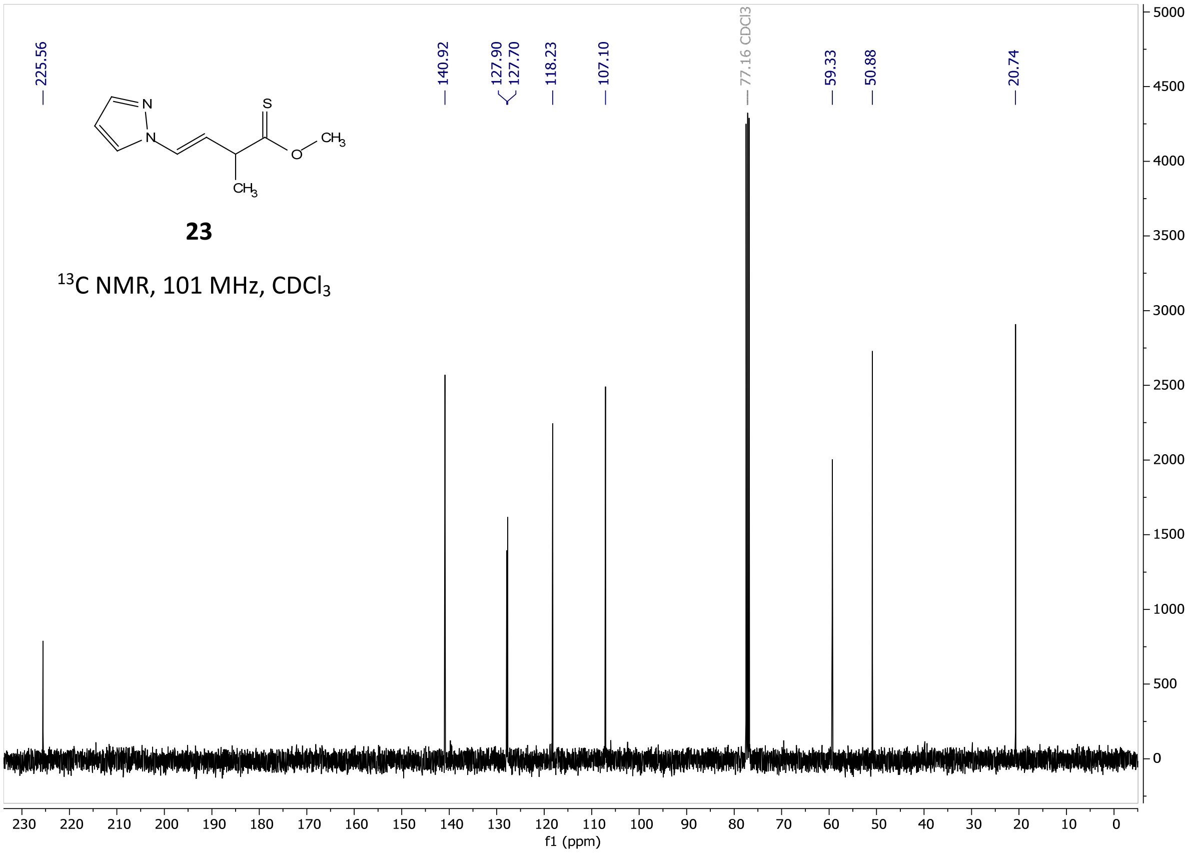

23

^{13}C NMR, 101 MHz, $CDCl_3$

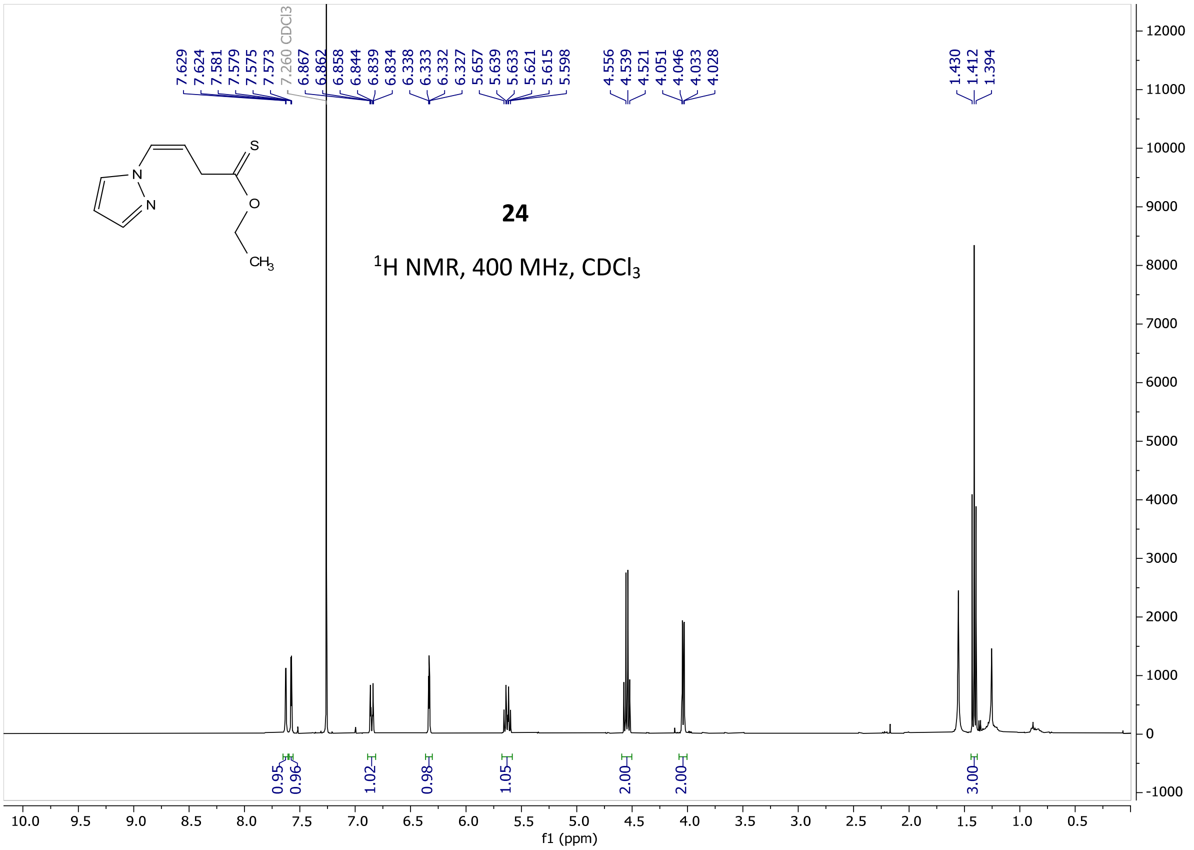
7.629
7.624
7.581
7.579
7.575
7.573
7.260 CDCl3
6.867
6.862
6.858
6.844
6.839
6.834
6.338
6.333
6.332
6.327
5.657
5.639
5.633
5.621
5.615
5.598
4.556
4.539
4.521
4.051
4.046
4.033
4.028
1.430
1.412
1.394
S
N
N
O
CH3
24
1H NMR, 400 MHz, CDCl3
0.95
0.96
1.02
0.98
1.05
2.00
2.00
3.00
f1 (ppm)

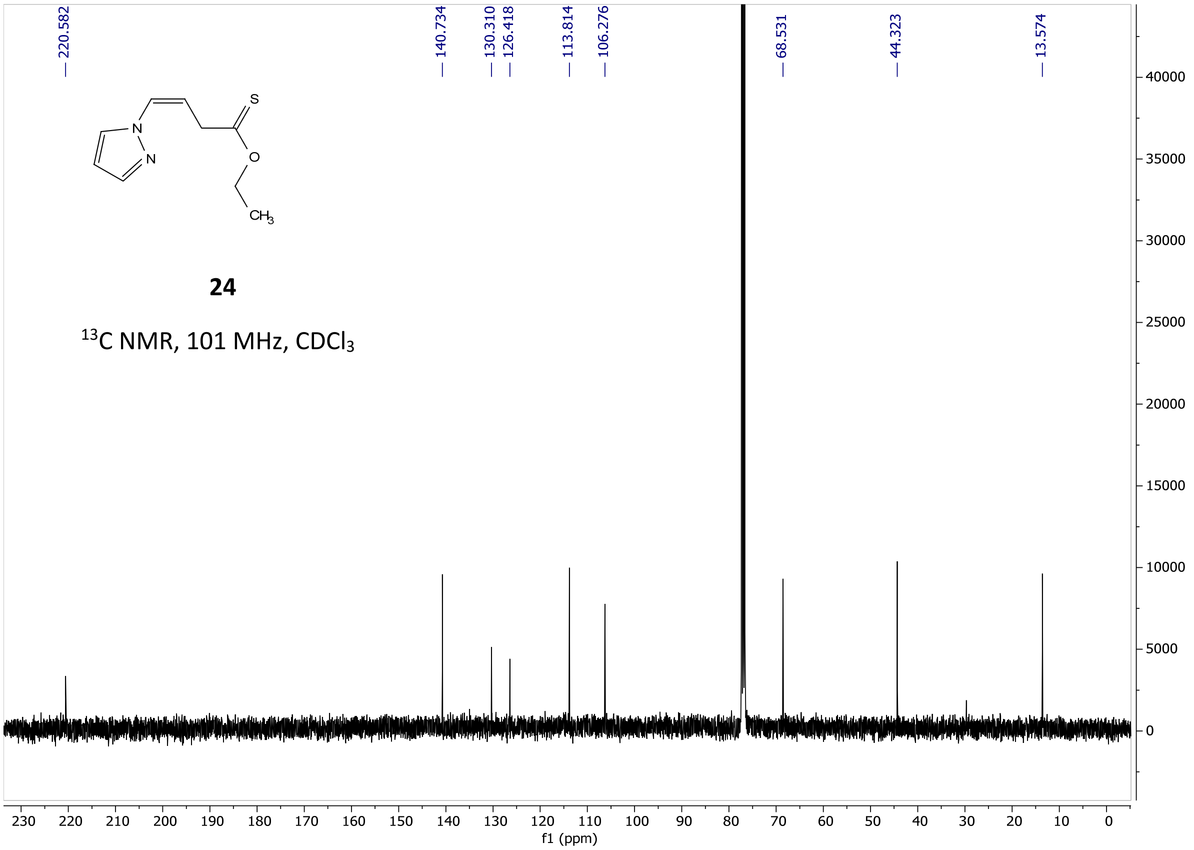
220.582
140.734
130.310
126.418
113.814
106.276
68.531
44.323
13.574
S
N
N
O
CH3
24
13C NMR, 101 MHz, CDCl3
f1 (ppm)

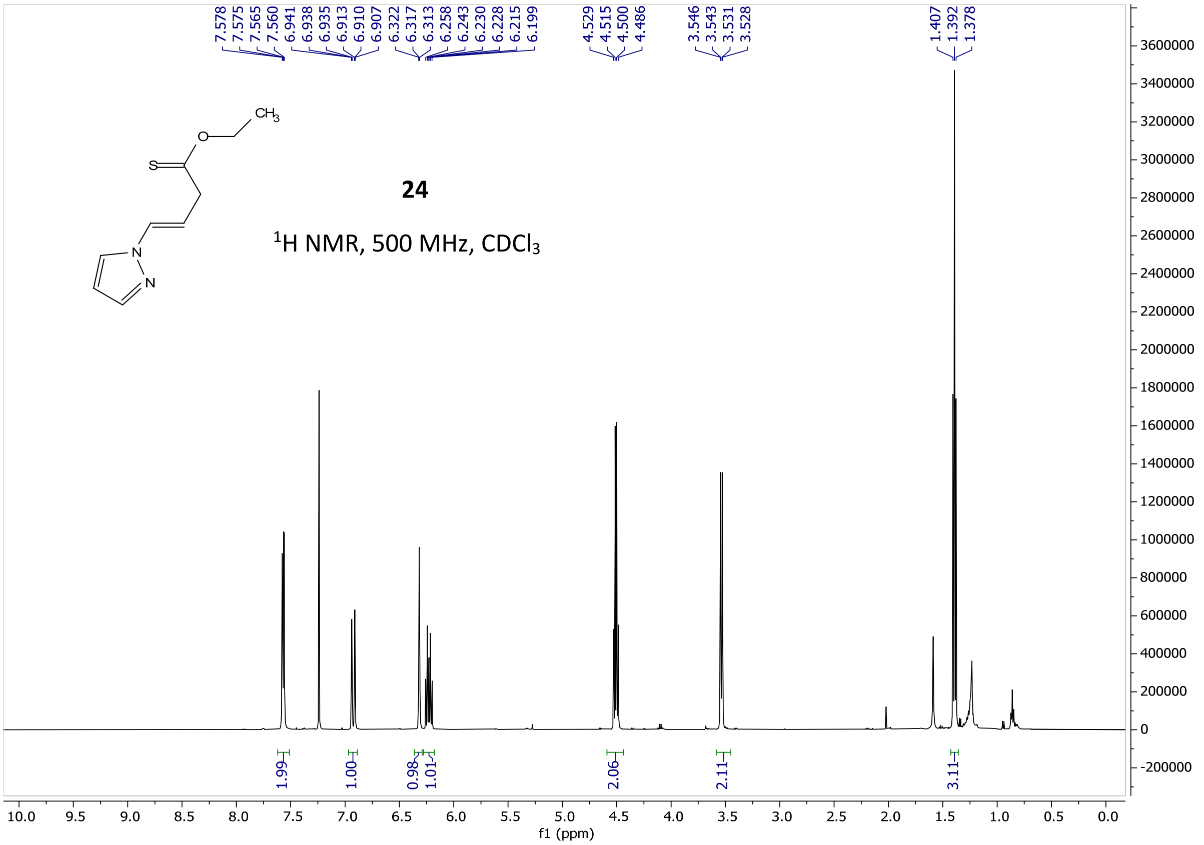

7.578
7.575
7.565
7.560
6.941
6.938
6.935
6.913
6.910
6.907
6.322
6.317
6.313
6.258
6.243
6.230
6.228
6.215
6.199
4.529
4.515
4.500
4.486
3.546
3.543
3.531
3.528
1.407
1.392
1.378
CH3
O
S
N
N
24
1H NMR, 500 MHz, CDCl3
1.99
1.00
0.98
1.01
2.06
2.11
3.11
f1 (ppm)

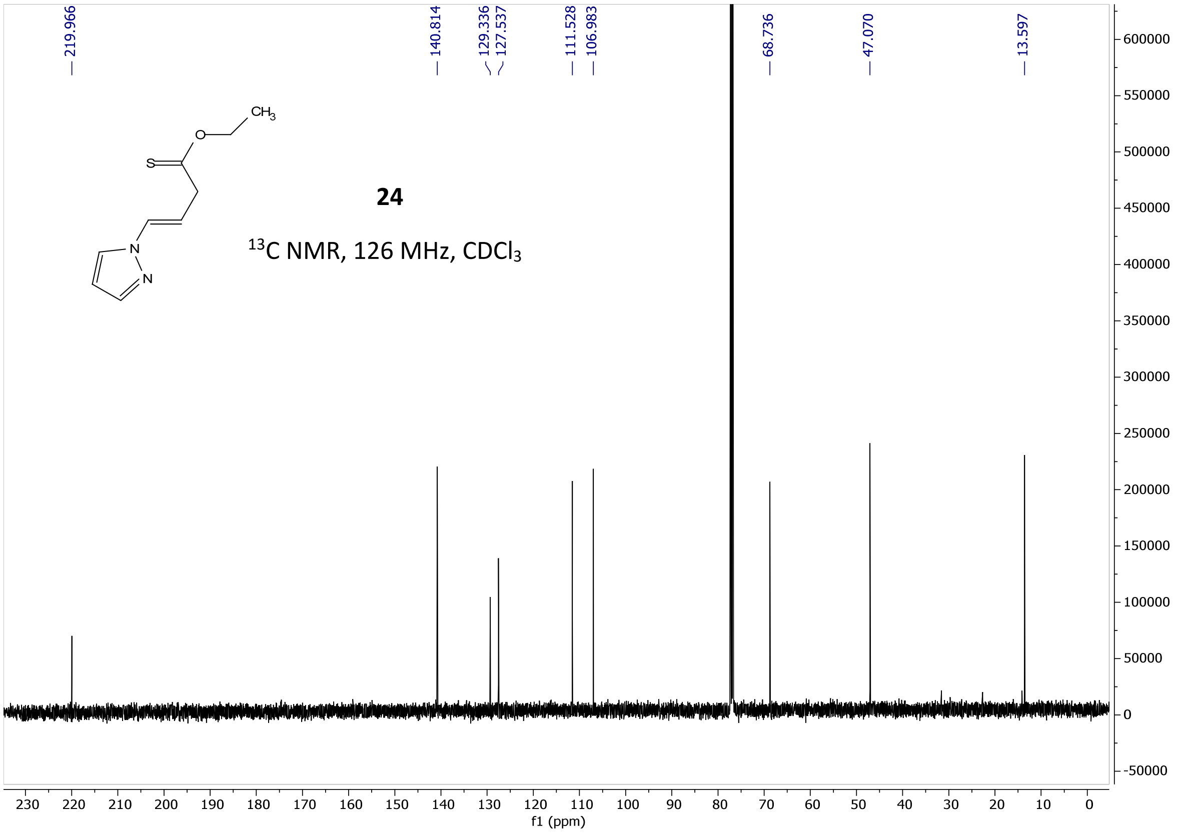

219.966
140.814
129.336
127.537
111.528
106.983
68.736
47.070
13.597
CH3
O
S
N
N
24
13C NMR, 126 MHz, CDCl3
f1 (ppm)

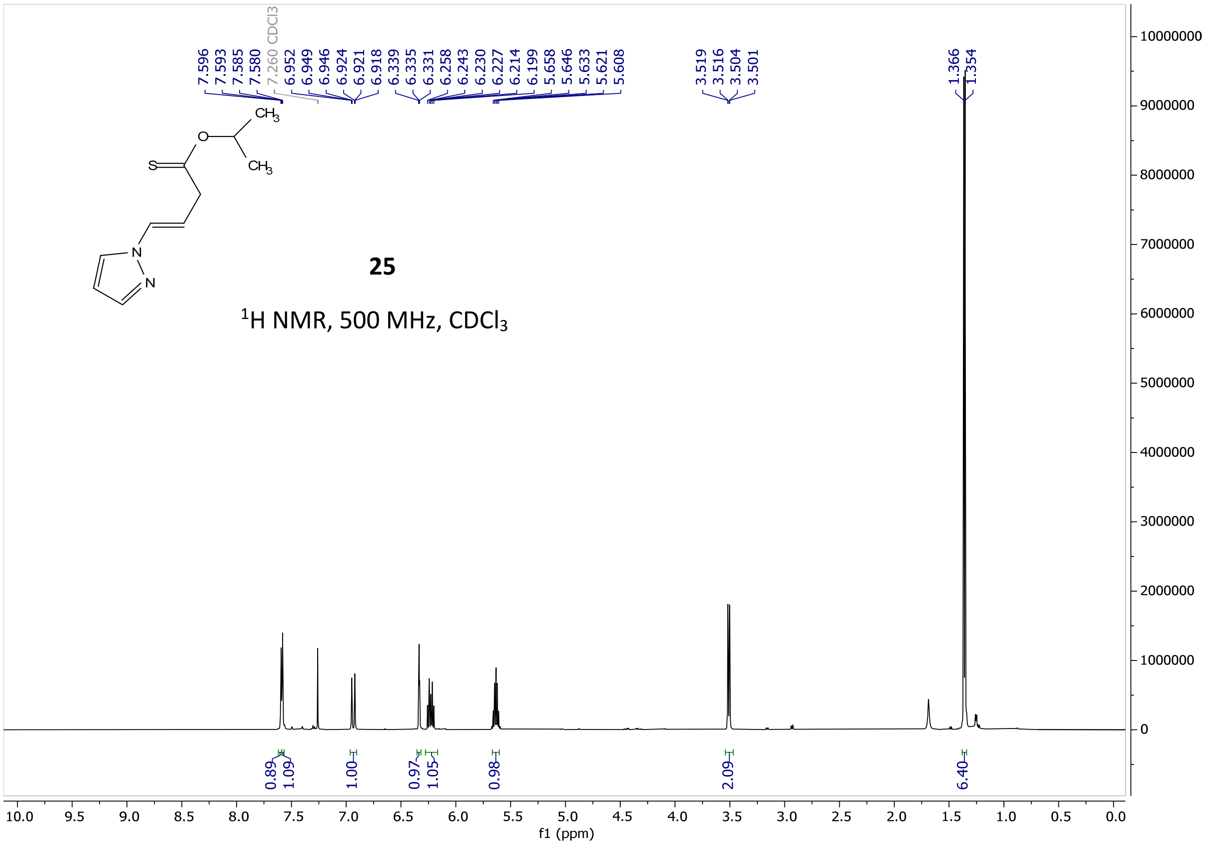
25
^{1}H NMR, 500 MHz, CDCl$_3$

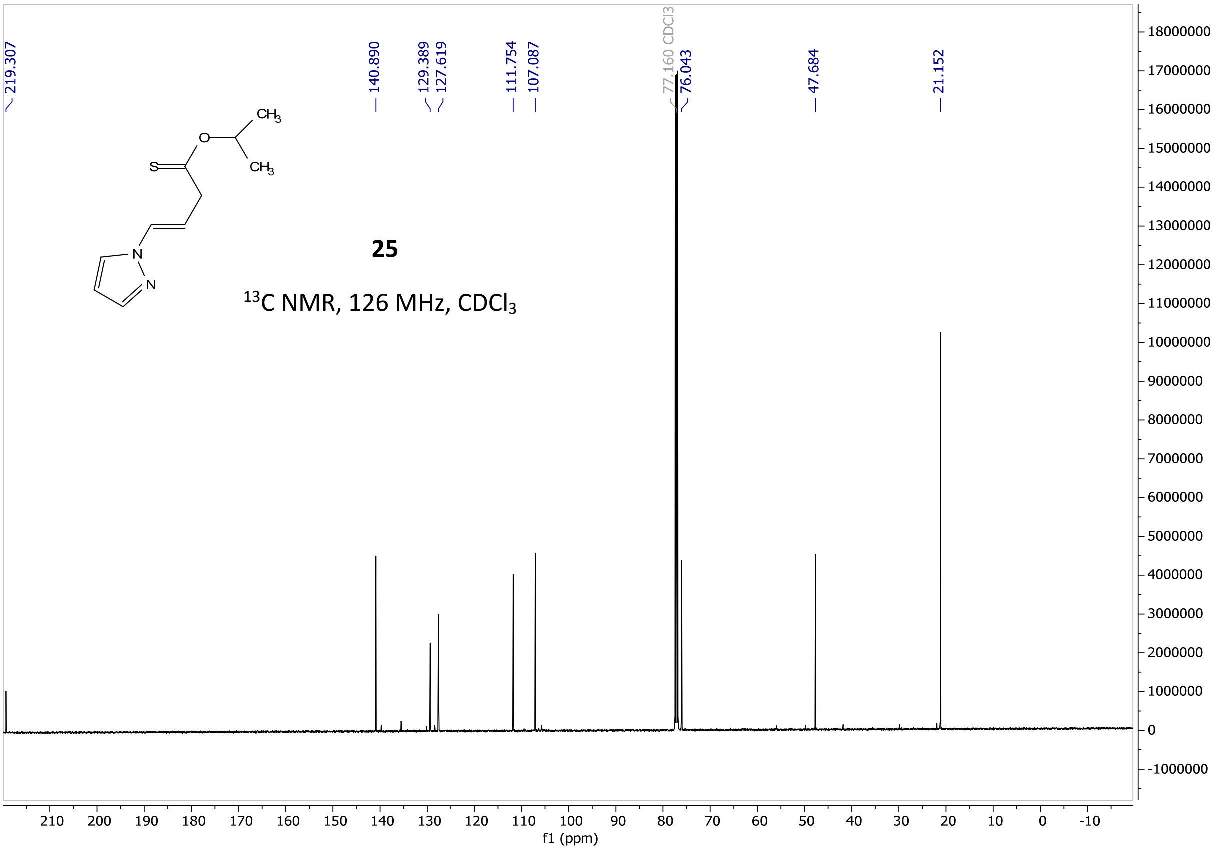
25
^{13}C NMR, 126 MHz, CDCl$_3$

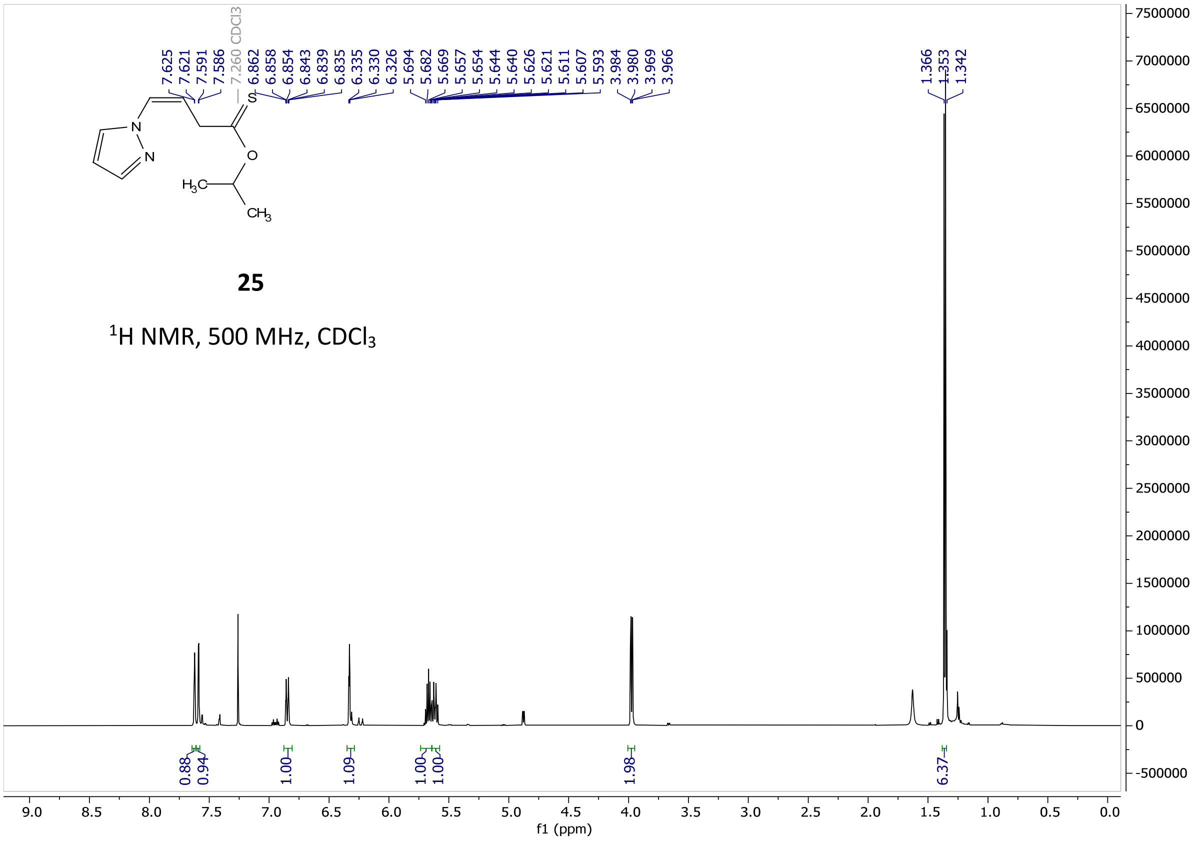
7.625
7.621
7.591
7.586
7.260 CDCl3
6.862
6.858
6.854
6.843
6.839
6.835
6.335
6.330
6.326
5.694
5.682
5.669
5.657
5.654
5.644
5.640
5.626
5.621
5.611
5.607
5.593
3.984
3.980
3.969
3.966
1.366
1.353
1.342
0.88
0.94
1.00
1.09
1.00
1.00
1.98
6.37
25
1H NMR, 500 MHz, CDCl3
f1 (ppm)

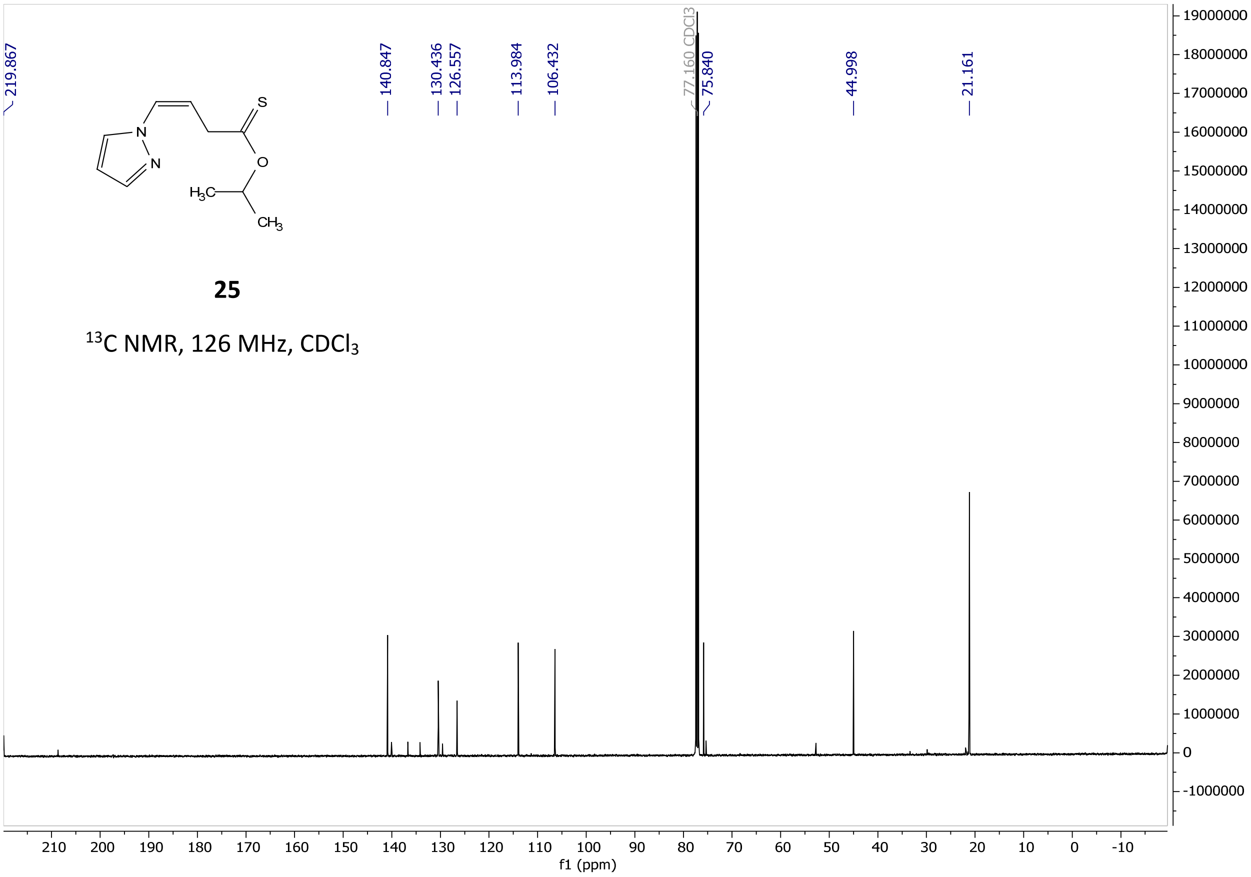
219.867
140.847
130.436
126.557
113.984
106.432
77.160 CDCl3
75.840
44.998
21.161
25
13C NMR, 126 MHz, CDCl3
f1 (ppm)

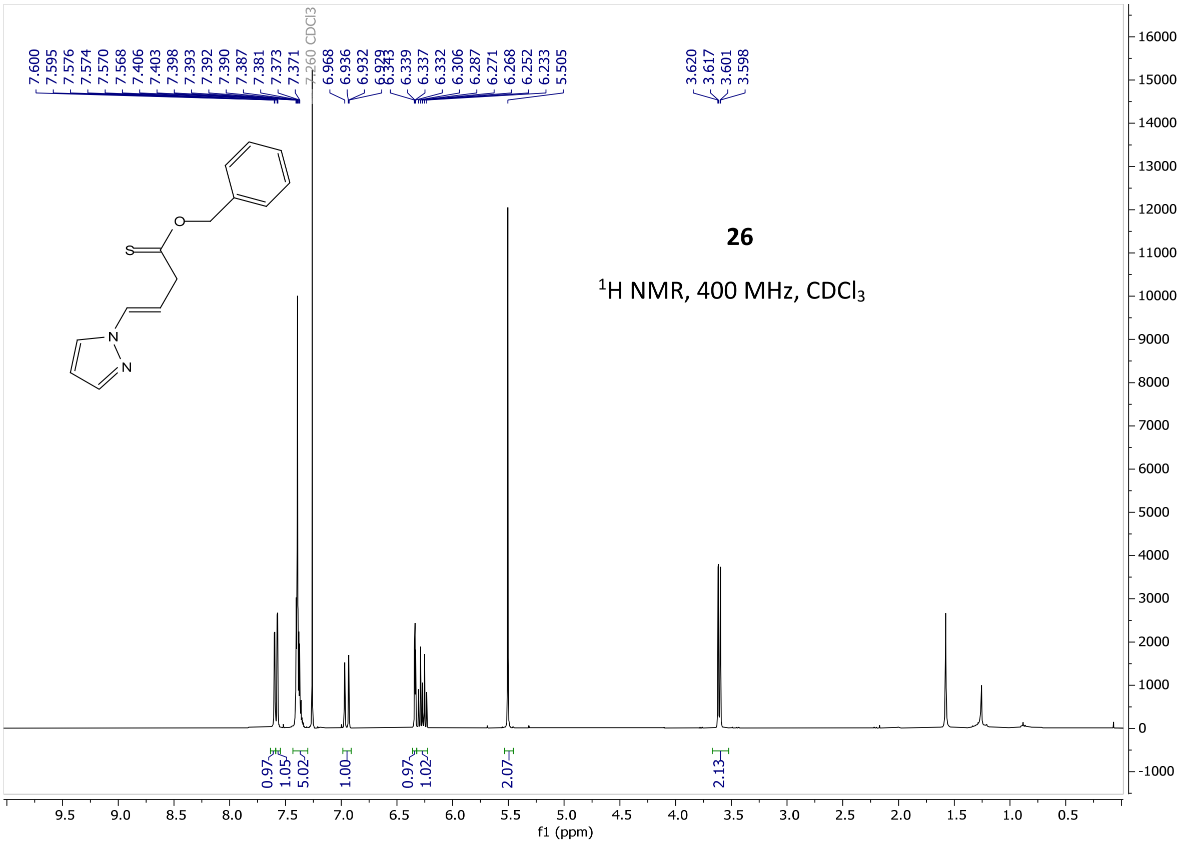

26
^{1}H NMR, 400 MHz, CDCl$_3$
f1 (ppm)

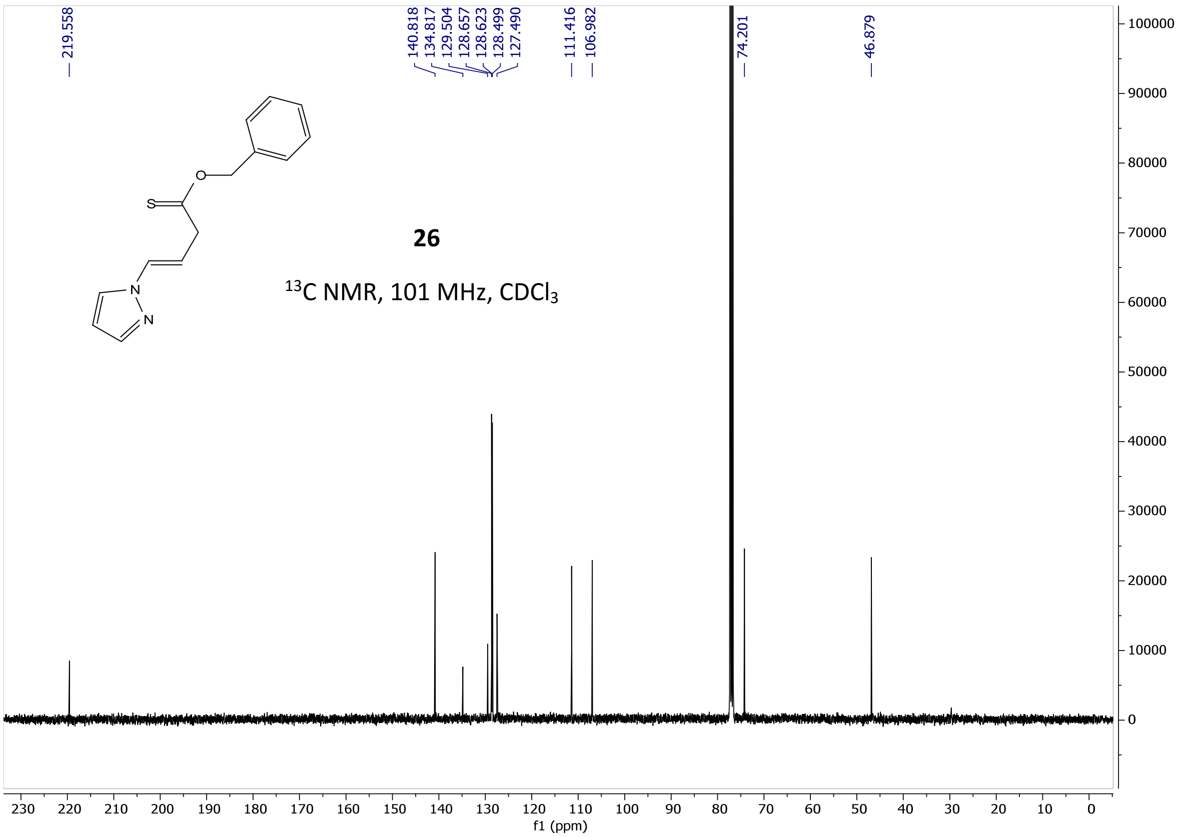

26
^{13}C NMR, 101 MHz, CDCl$_3$
f1 (ppm)

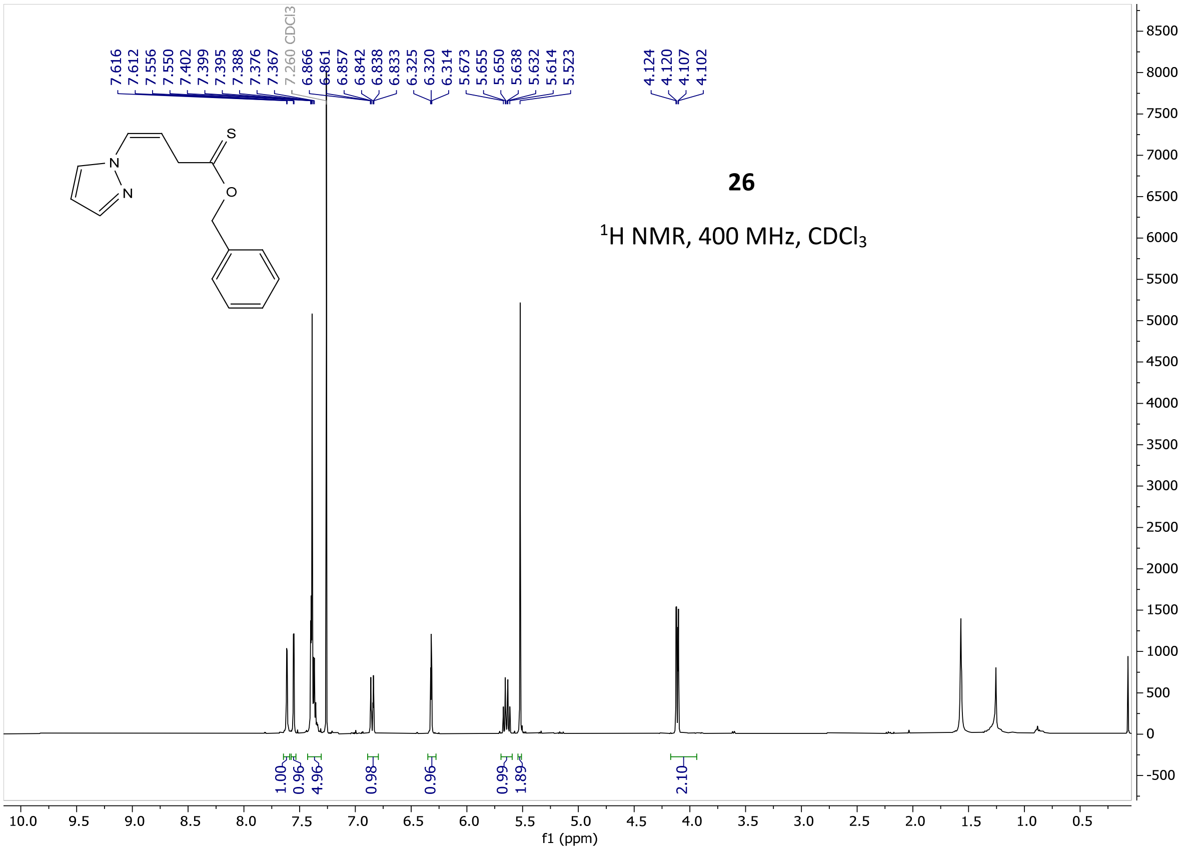
7.616 7.612 7.556 7.550 7.402 7.399 7.395 7.388 7.376 7.367 7.260 CDCl3 6.866 6.861 6.857 6.842 6.838 6.833 6.325 6.320 6.314 5.673 5.655 5.650 5.638 5.632 5.614 5.523
4.124 4.120 4.107 4.102
26
^{1}H NMR, 400 MHz, CDCl$_3$
1.00 0.96 4.96 0.98 0.96 0.99 1.89 2.10
f1 (ppm)

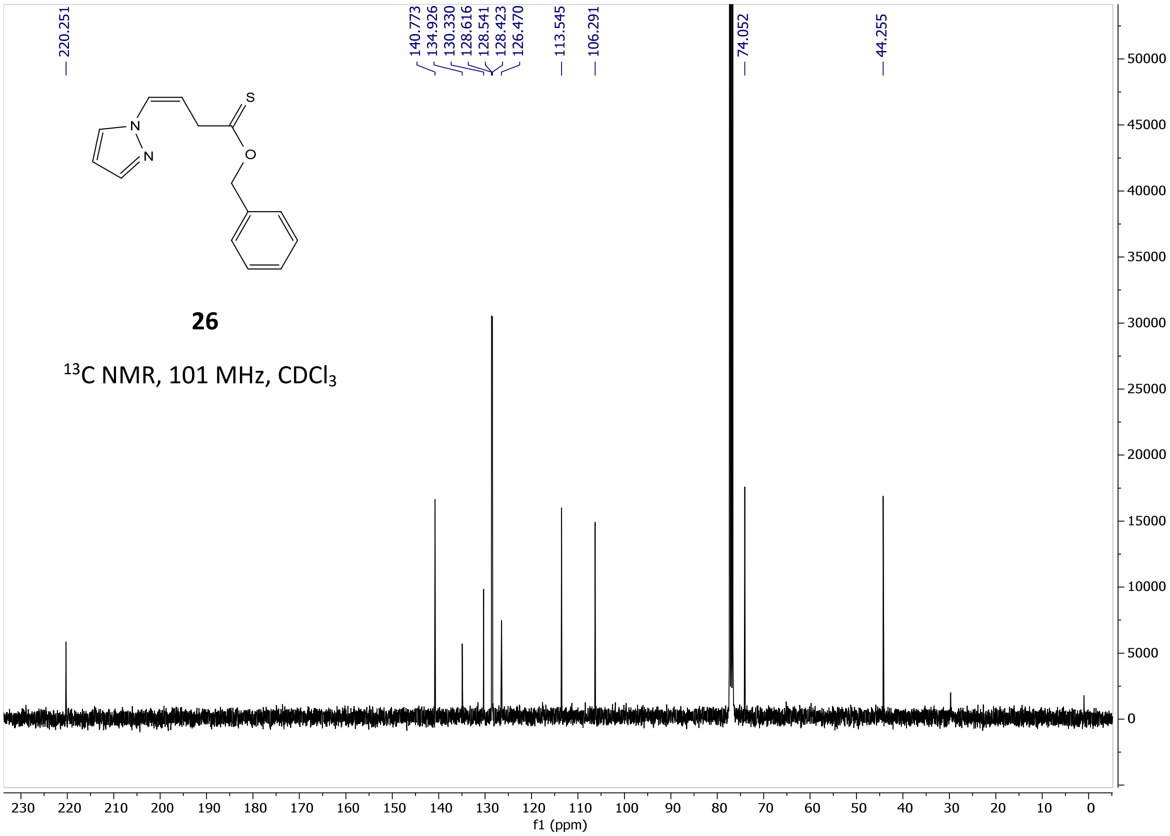
220.251
140.773 134.926 130.330 128.616 128.541 128.423 126.470
113.545
106.291
74.052
44.255
26
^{13}C NMR, 101 MHz, CDCl$_3$
f1 (ppm)

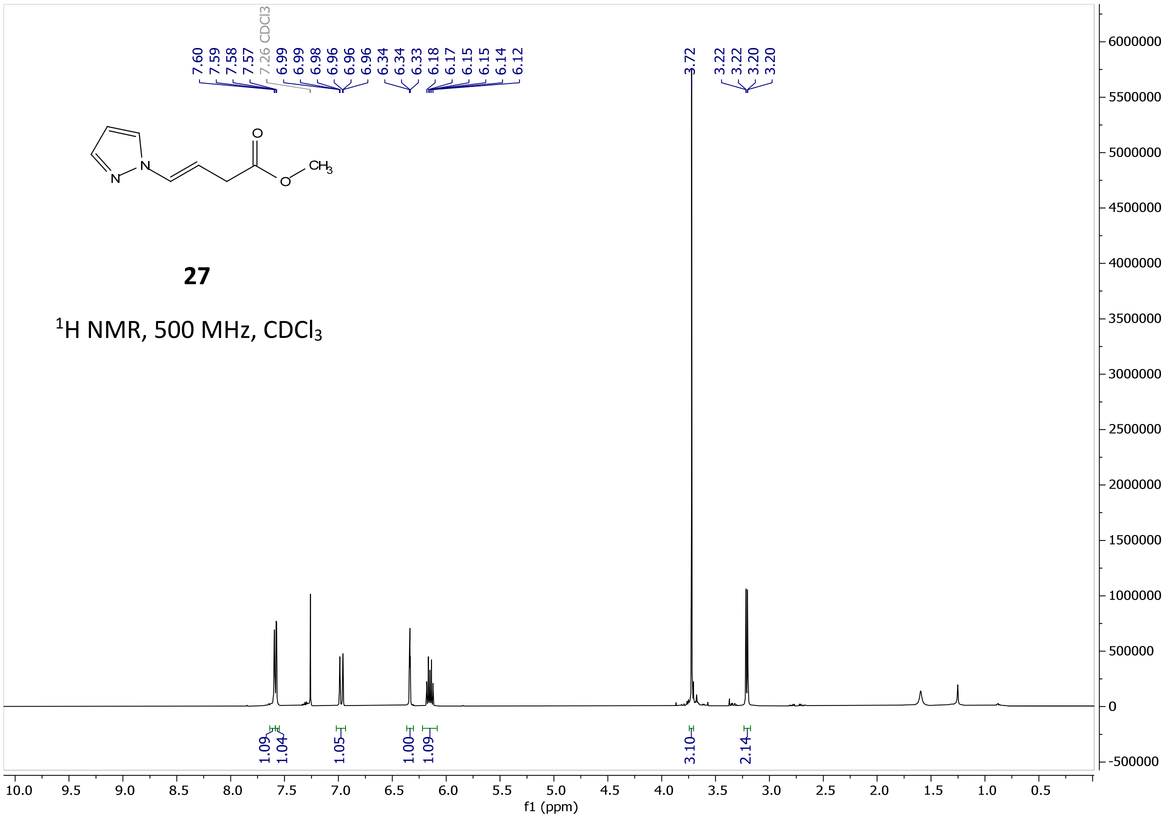
7.60
7.59
7.58
7.57
7.26 CDCl3
6.99
6.99
6.98
6.96
6.96
6.34
6.34
6.33
6.18
6.17
6.15
6.15
6.14
6.12
3.72
3.22
3.22
3.20
3.20
27
1H NMR, 500 MHz, CDCl3
1.09
1.04
1.05
1.00
1.09
3.10
2.14
f1 (ppm)

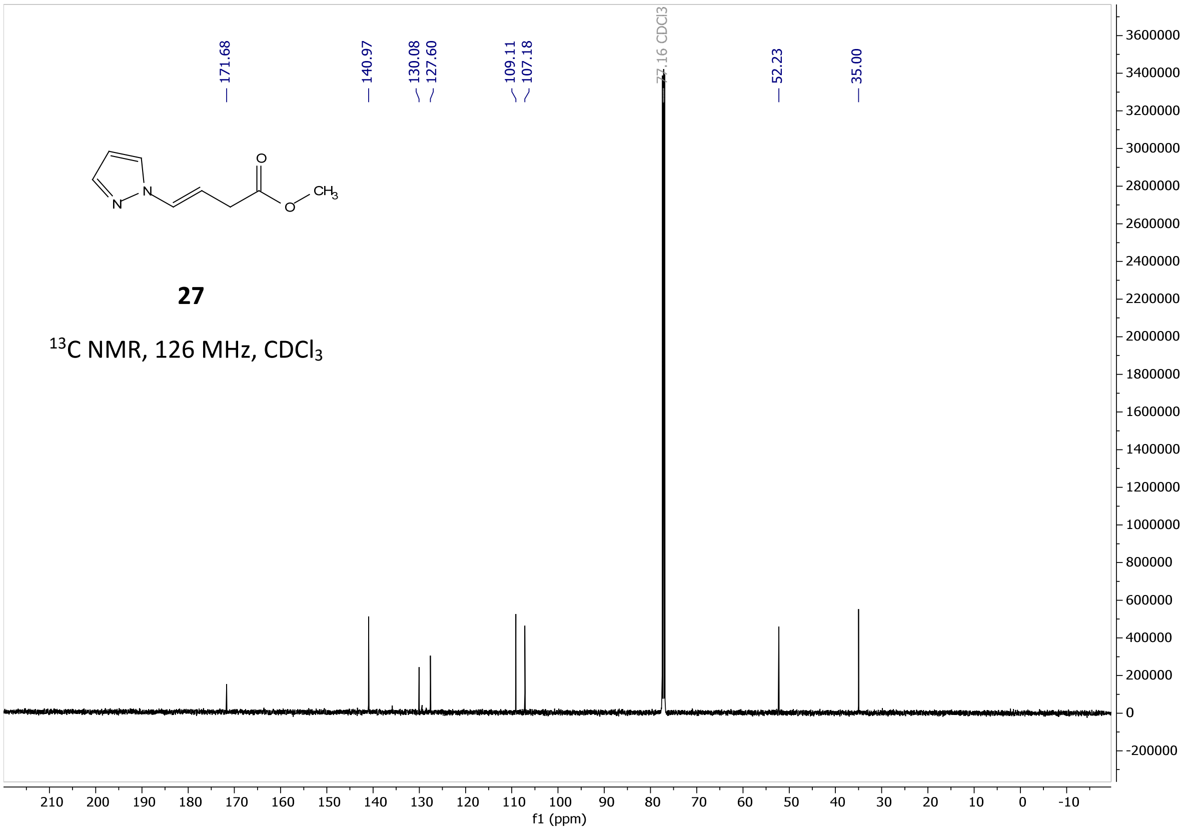
171.68
140.97
130.08
127.60
109.11
107.18
77.16 CDCl3
52.23
35.00
27
13C NMR, 126 MHz, CDCl3
f1 (ppm)

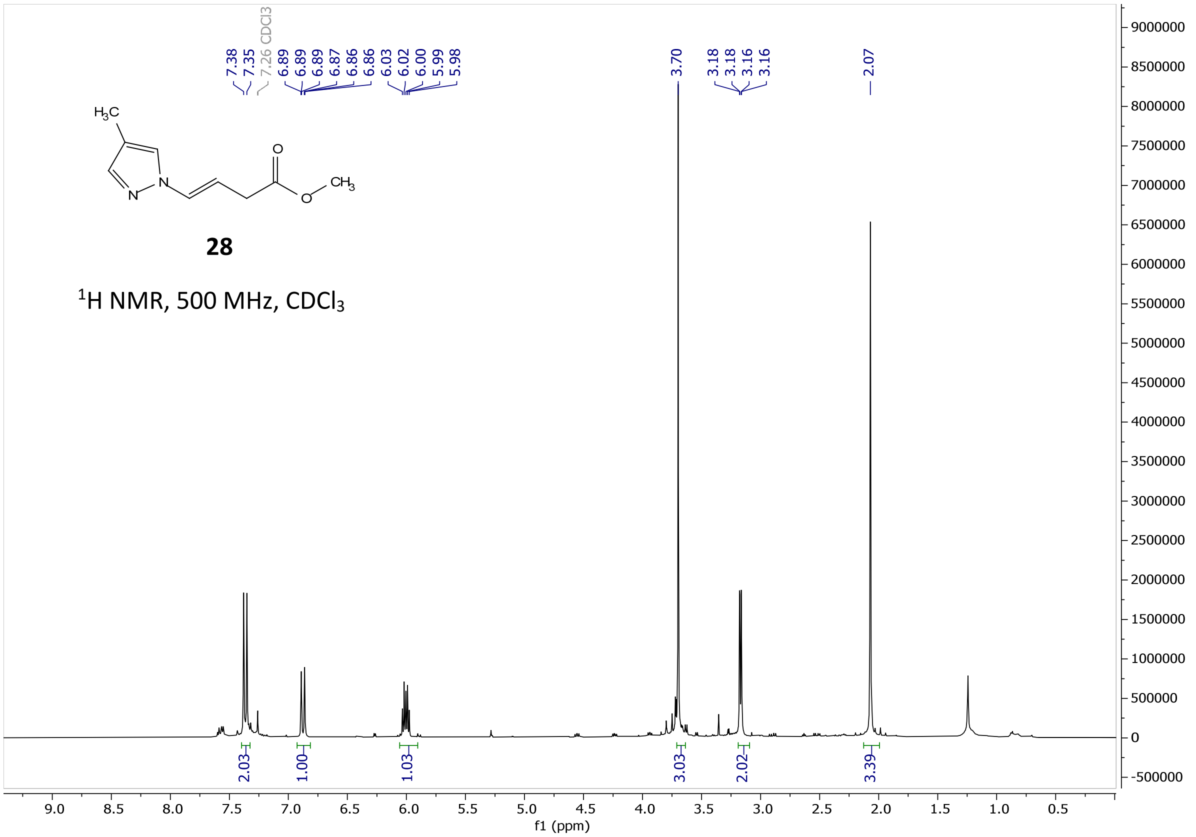
7.38
7.35
7.26 CDCl3
6.89
6.89
6.89
6.87
6.86
6.86
6.03
6.02
6.00
5.99
5.98
3.70
3.18
3.18
3.16
3.16
2.07
H3C
O
N
N
O
CH3
28
1H NMR, 500 MHz, CDCl3
2.03
1.00
1.03
3.03
2.02
3.39
f1 (ppm)

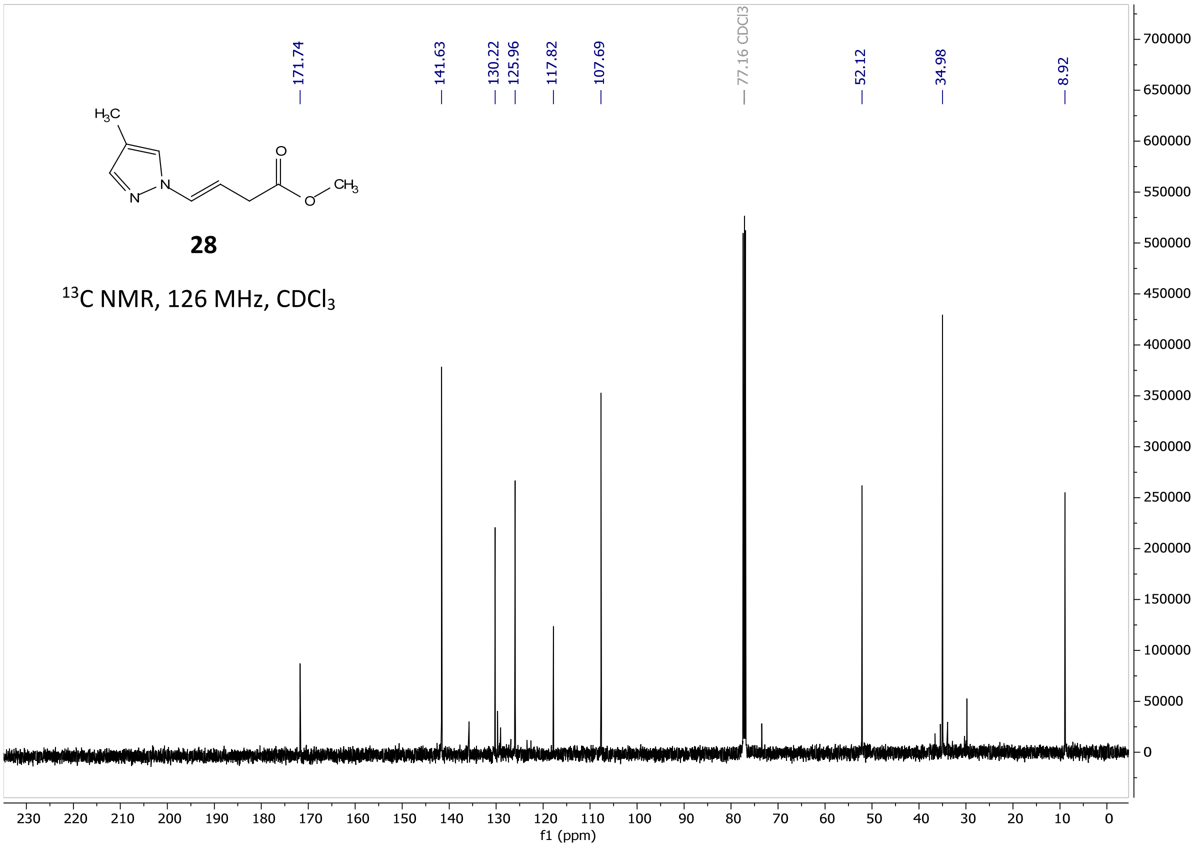
171.74
141.63
130.22
125.96
117.82
107.69
77.16 CDCl3
52.12
34.98
8.92
H3C
O
N
N
O
CH3
28
13C NMR, 126 MHz, CDCl3
f1 (ppm)

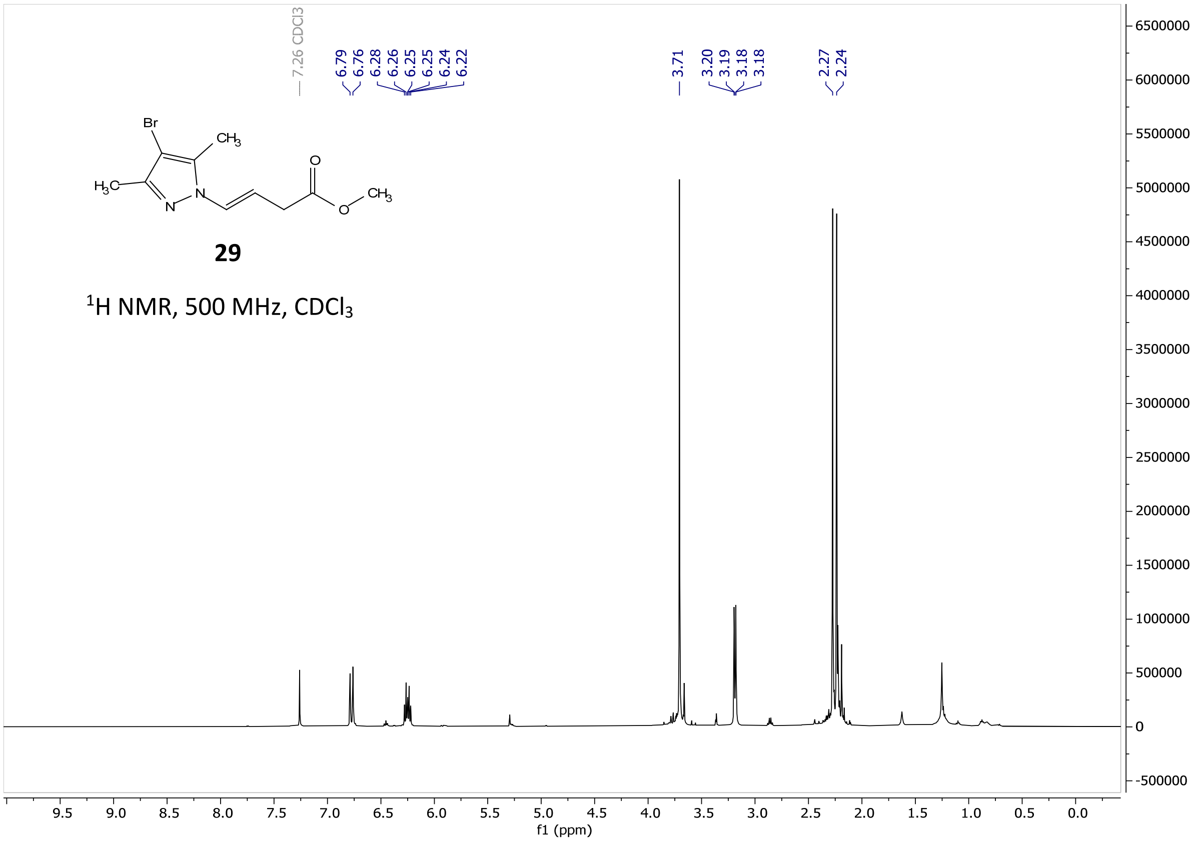
7.26 CDCl3
6.79
6.76
6.28
6.26
6.25
6.25
6.24
6.22
3.71
3.20
3.19
3.18
3.18
2.27
2.24
Br
CH3
H3C
N
N
O
O
CH3
29
1H NMR, 500 MHz, CDCl3
f1 (ppm)

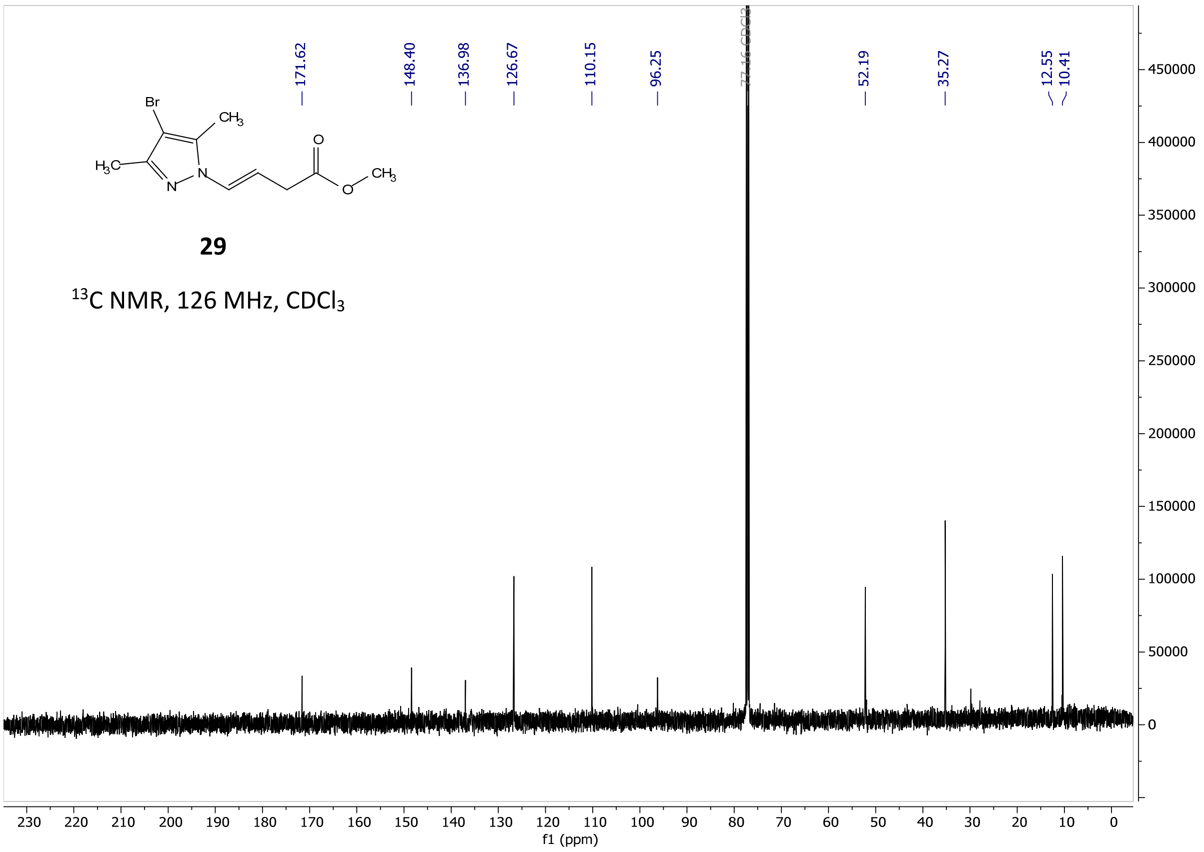
171.62
148.40
136.98
126.67
110.15
96.25
77.16 CDCl3
52.19
35.27
12.55
10.41
Br
CH3
H3C
N
N
O
O
CH3
29
13C NMR, 126 MHz, CDCl3
f1 (ppm)

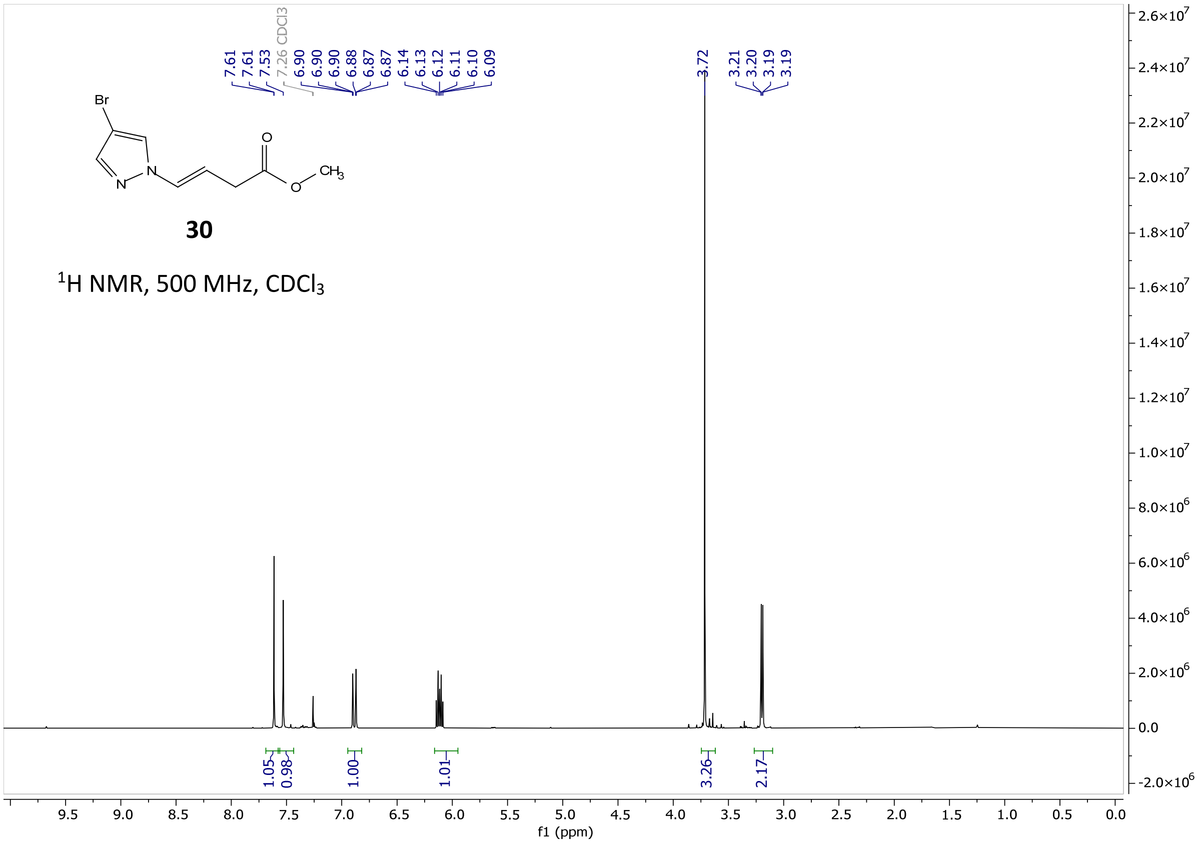

30
^{1}H NMR, 500 MHz, CDCl$_3$
7.61 7.61 7.53 7.26 CDCl3 6.90 6.90 6.90 6.88 6.87 6.87 6.14 6.13 6.12 6.11 6.10 6.09 3.72 3.21 3.20 3.19 3.19
1.05 0.98 1.00 1.01 3.26 2.17
f1 (ppm)

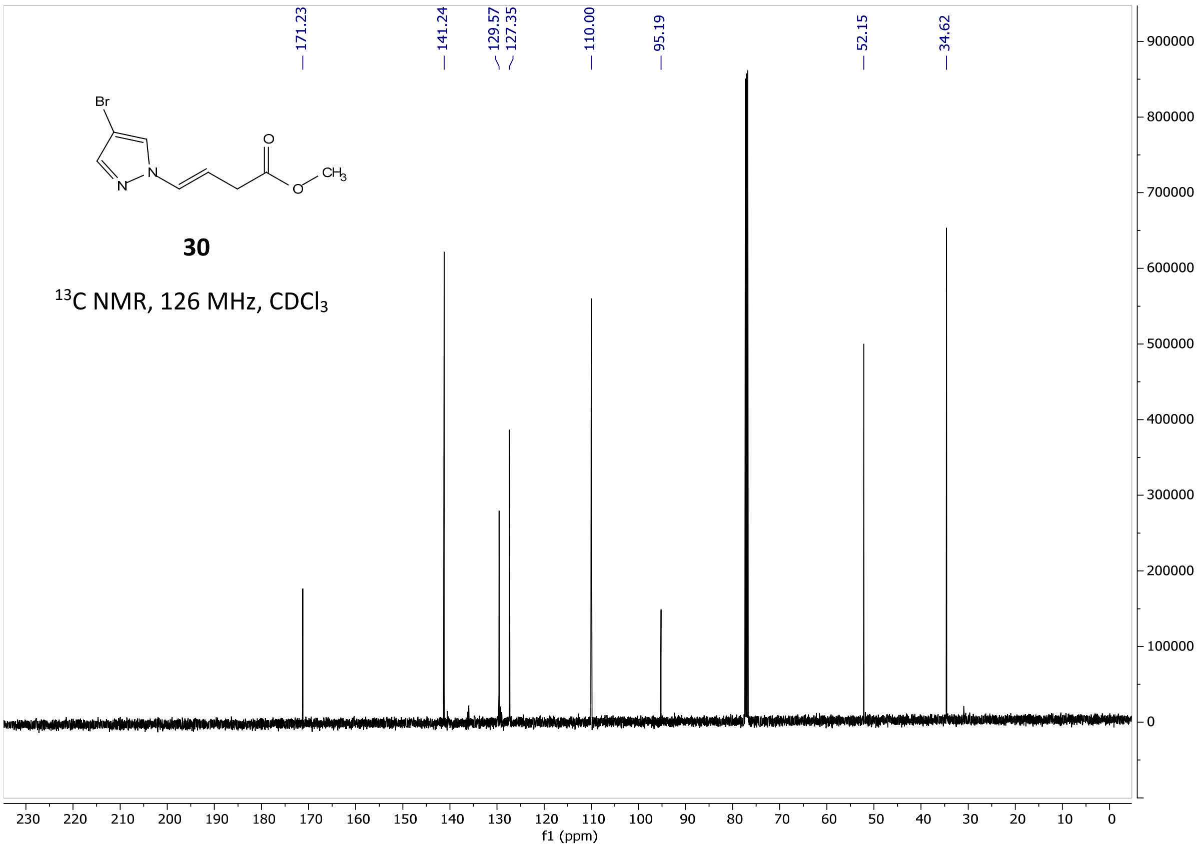

30
^{13}C NMR, 126 MHz, CDCl$_3$
171.23 141.24 129.57 127.35 110.00 95.19 52.15 34.62
f1 (ppm)

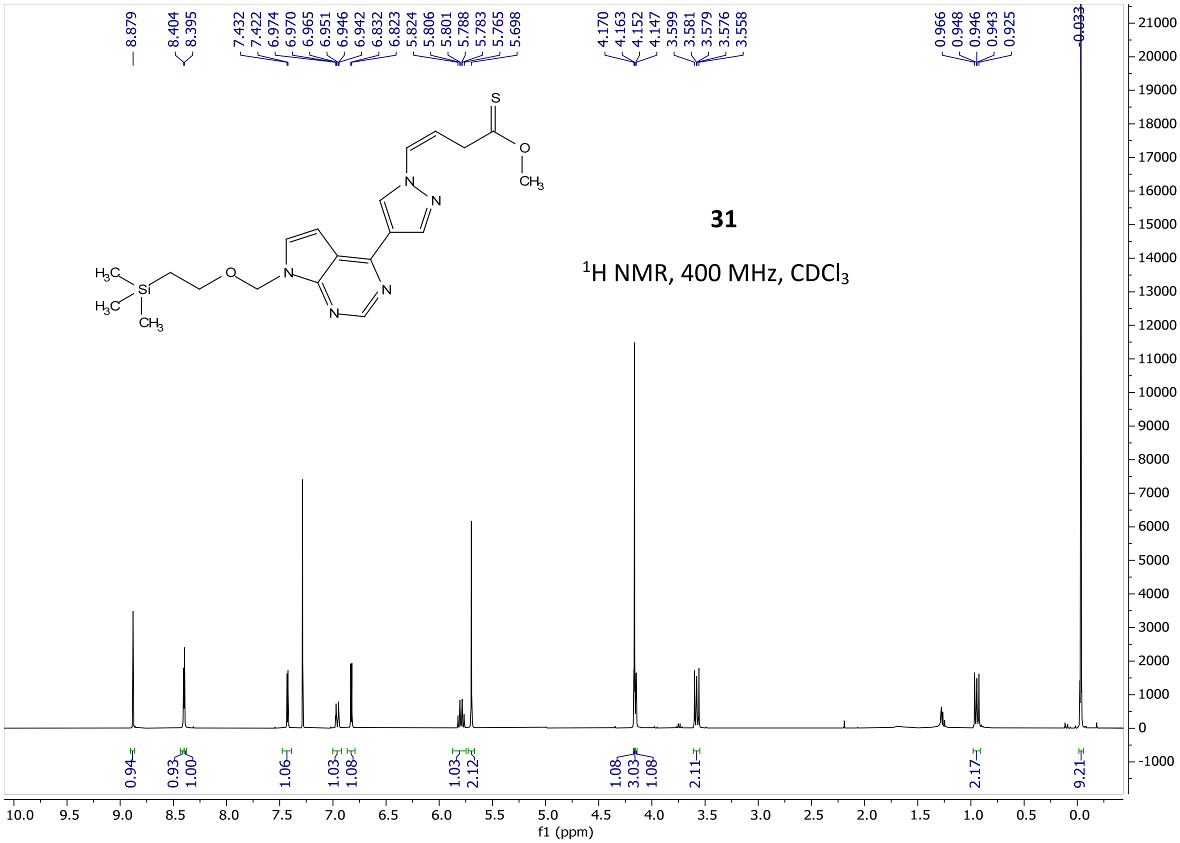
31
^{1}H NMR, 400 MHz, CDCl$_3$
f1 (ppm)

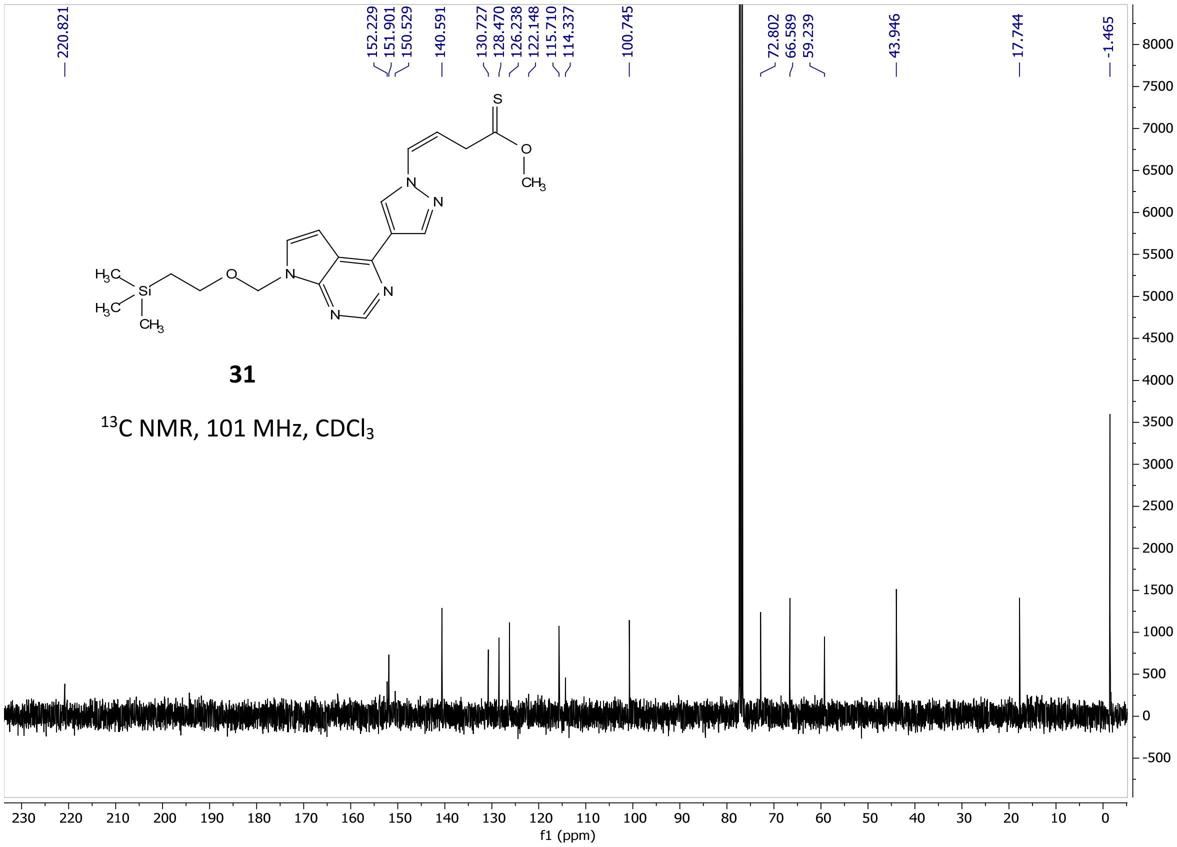
31
^{13}C NMR, 101 MHz, CDCl$_3$
f1 (ppm)

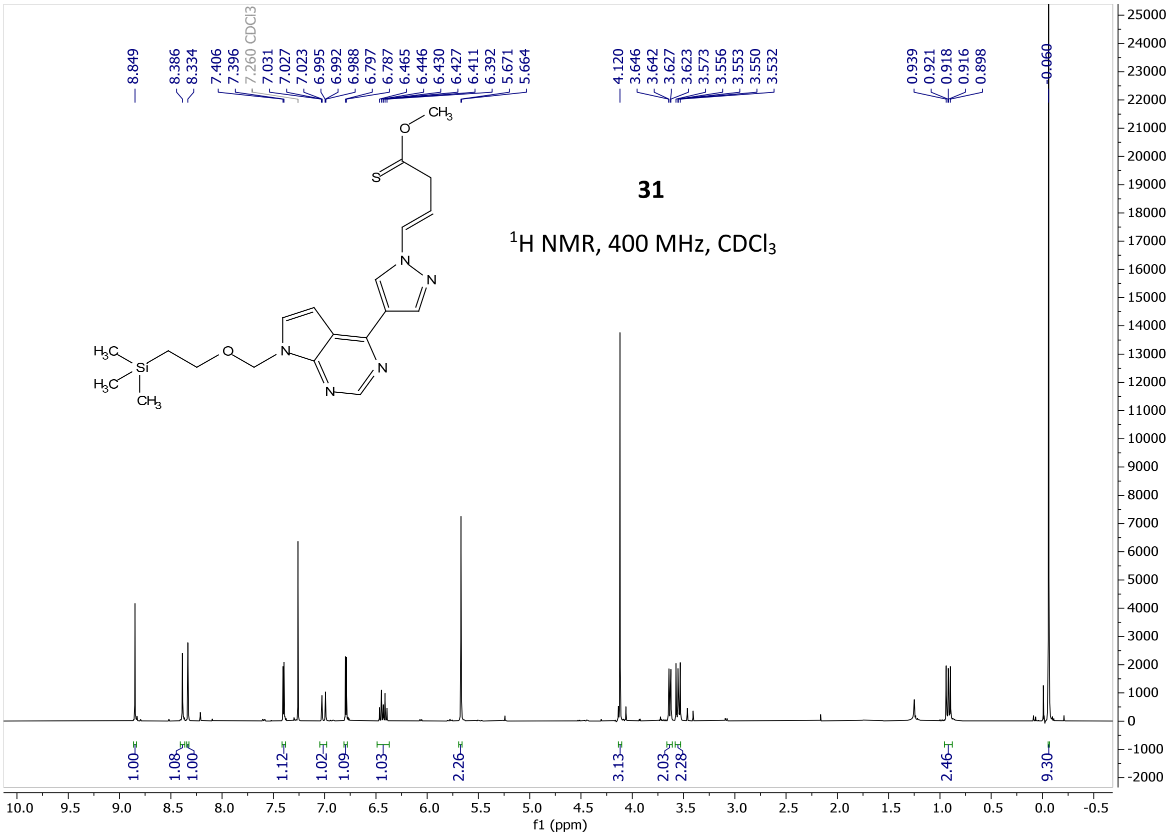
31
1H NMR, 400 MHz, CDCl3
8.849
8.386
8.334
7.406
7.396
7.260 CDCl3
7.031
7.027
7.023
6.995
6.992
6.988
6.797
6.787
6.465
6.446
6.430
6.427
6.411
6.392
5.671
5.664
4.120
3.646
3.642
3.627
3.623
3.573
3.556
3.553
3.550
3.532
0.939
0.921
0.918
0.916
0.898
0.060
1.00
1.08
1.00
1.12
1.02
1.09
1.03
2.26
3.13
2.03
2.28
2.46
9.30
f1 (ppm)

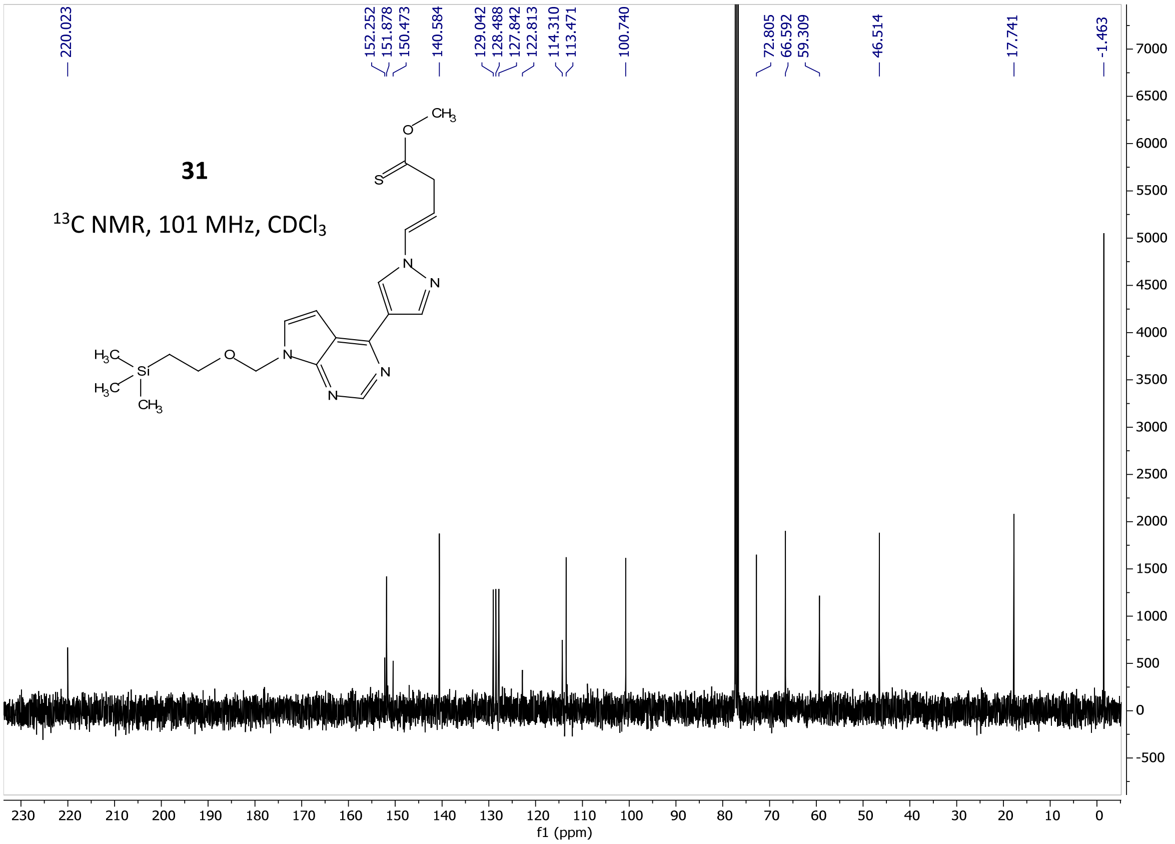
31
13C NMR, 101 MHz, CDCl3
220.023
152.252
151.878
150.473
140.584
129.042
128.488
127.842
122.813
114.310
113.471
100.740
72.805
66.592
59.309
46.514
17.741
-1.463
f1 (ppm)

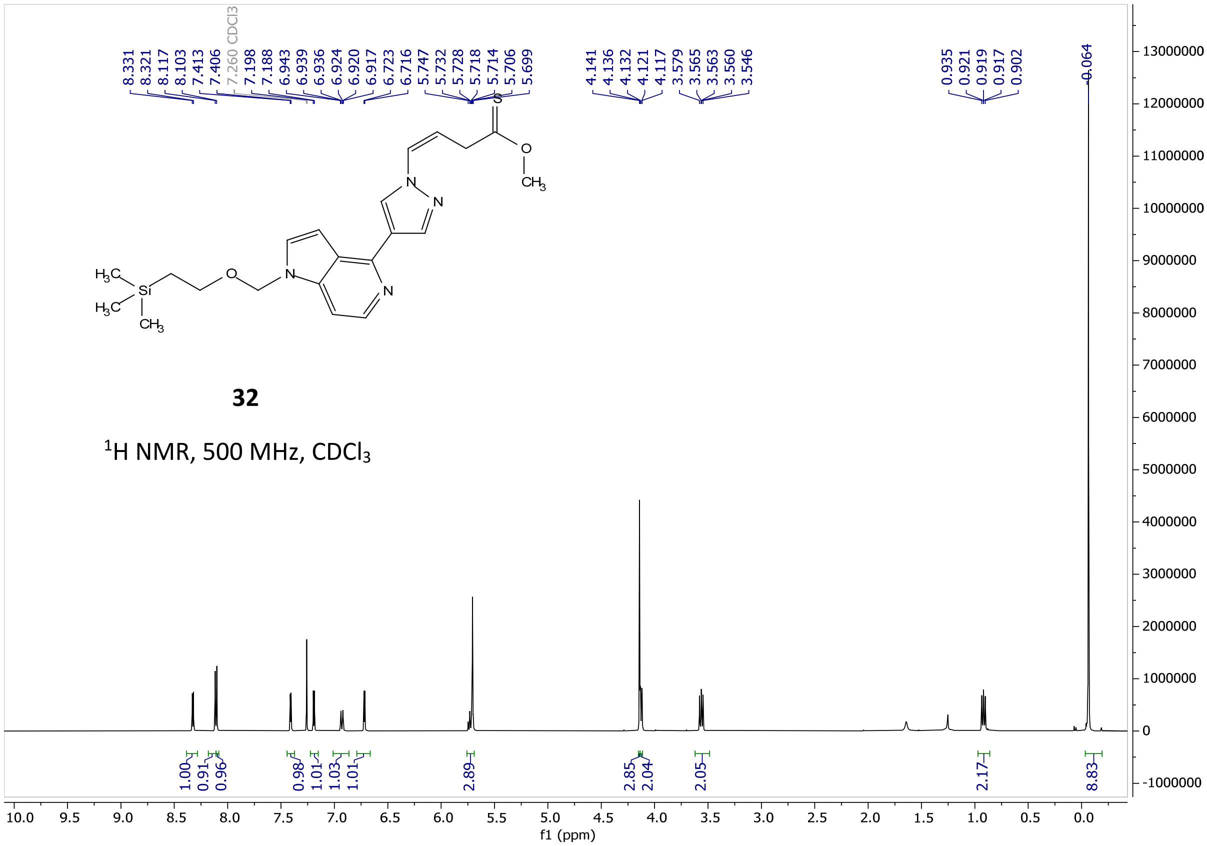
32
^{1}H NMR, 500 MHz, $CDCl_3$
8.331 8.321 8.117 8.103 7.413 7.406 7.260 CDCl3 7.198 7.188 6.943 6.939 6.936 6.924 6.920 6.917 6.723 6.716 5.747 5.732 5.728 5.718 5.714 5.706 5.699
4.141 4.136 4.132 4.121 4.117 3.579 3.565 3.563 3.560 3.546
0.935 0.921 0.919 0.917 0.902
-0.064
1.00 0.91 0.96 0.98 1.01 1.03 1.01 2.89 2.85 2.04 2.05 2.17 8.83
f1 (ppm)

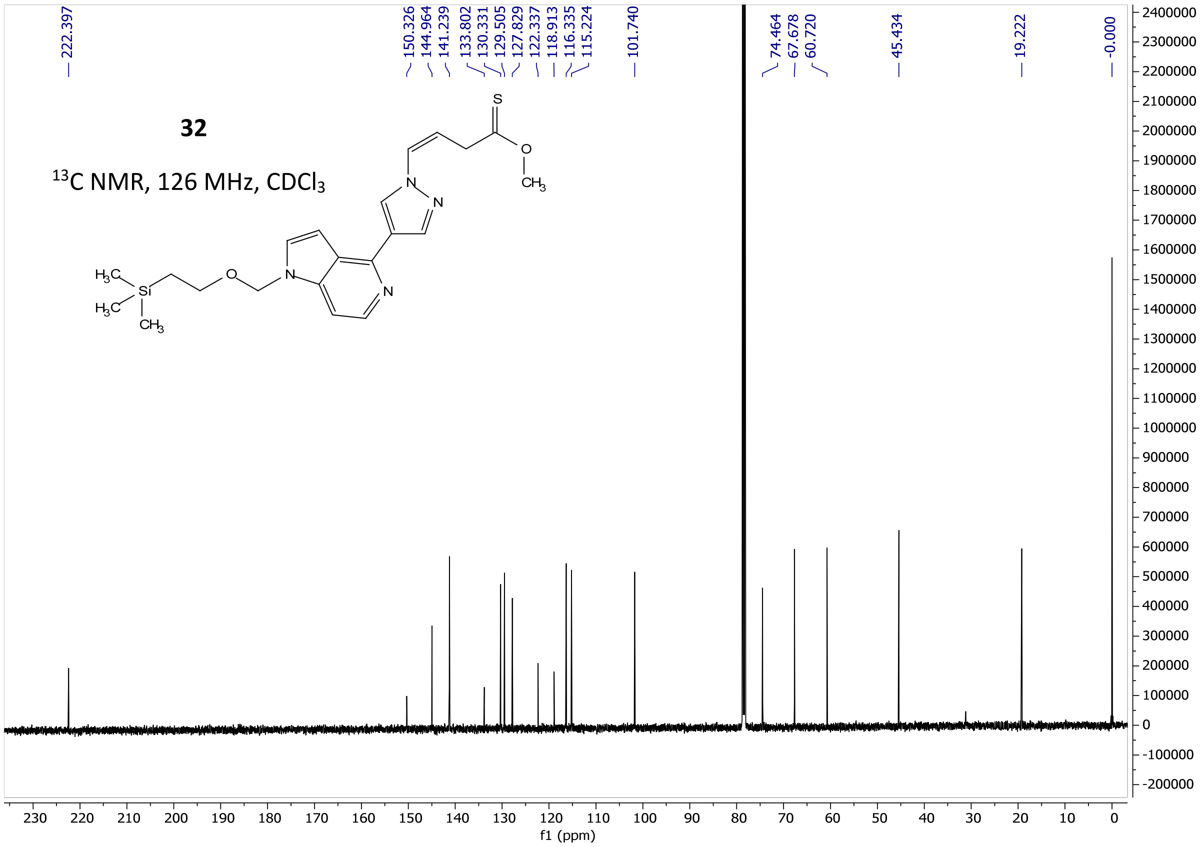
32
^{13}C NMR, 126 MHz, $CDCl_3$
222.397
150.326 144.964 141.239 133.802 130.331 129.505 127.829 122.337 118.913 116.335 115.224
101.740
74.464 67.678 60.720
45.434
19.222
-0.000
f1 (ppm)

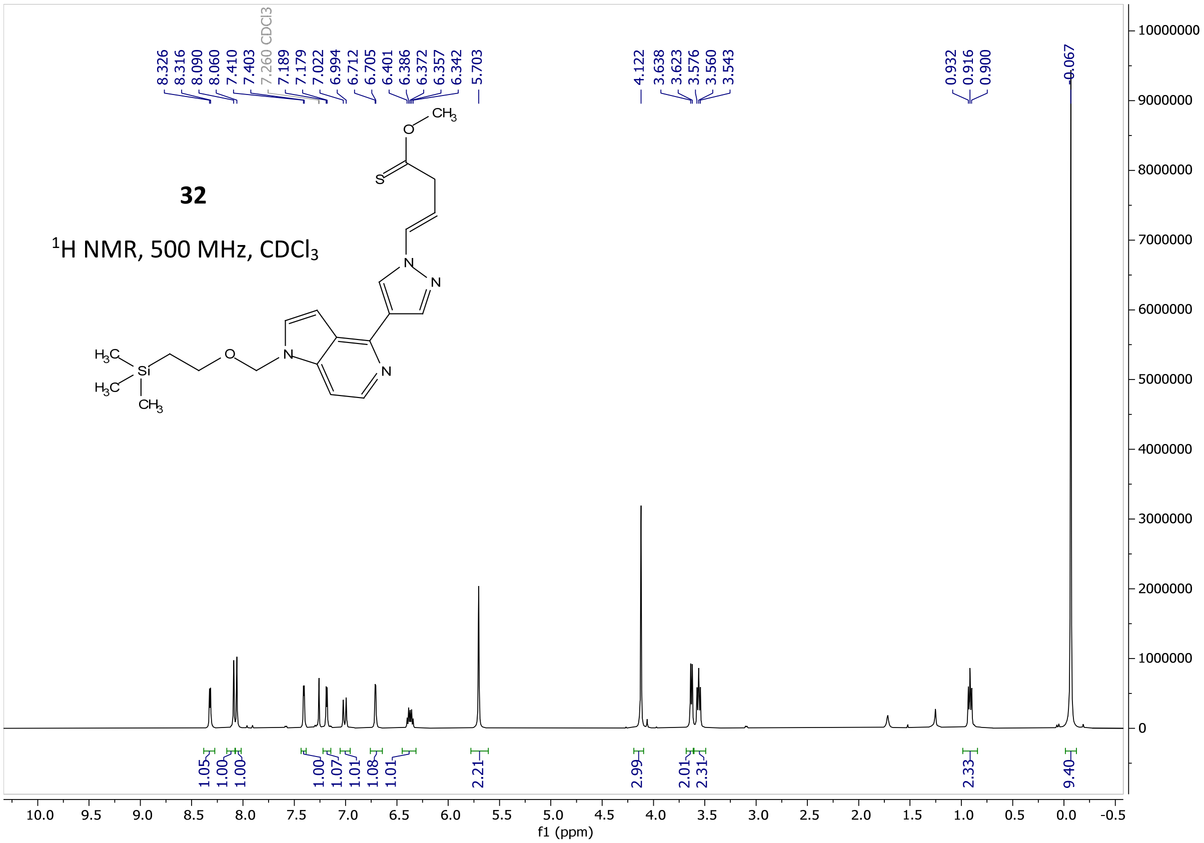
32
^{1}H NMR, 500 MHz, CDCl$_3$
8.326 8.316 8.090 8.060 7.410 7.403 7.260 CDCl3 7.189 7.179 7.022 6.994 6.712 6.705 6.401 6.386 6.372 6.357 6.342 5.703
4.122 3.638 3.623 3.576 3.560 3.543
0.932 0.916 0.900
-0.067
1.05 1.00 1.00 1.00 1.07 1.01 1.08 1.01 2.21 2.99 2.01 2.31 2.33 9.40
f1 (ppm)

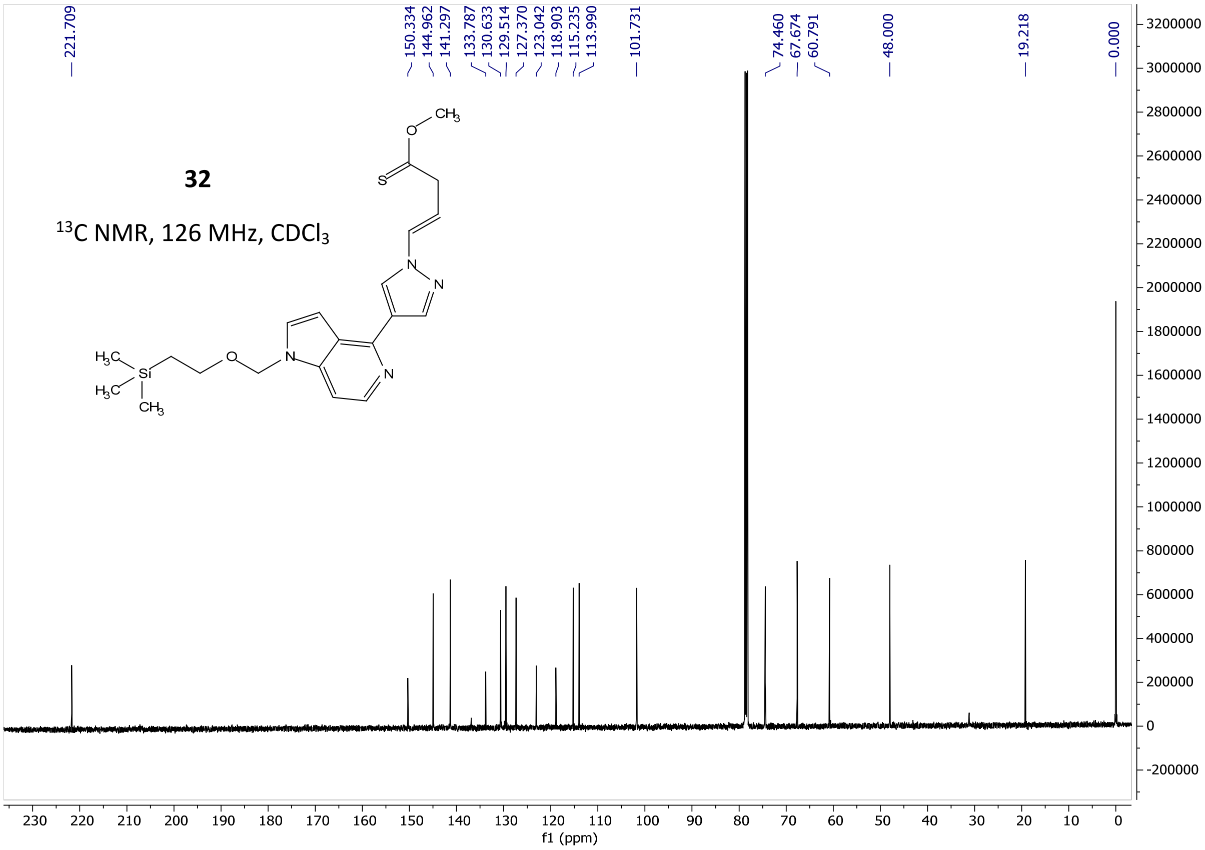
32
^{13}C NMR, 126 MHz, CDCl$_3$
221.709
150.334 144.962 141.297 133.787 130.633 129.514 127.370 123.042 118.903 115.235 113.990
101.731
74.460 67.674 60.791
48.000
19.218
0.000
f1 (ppm)

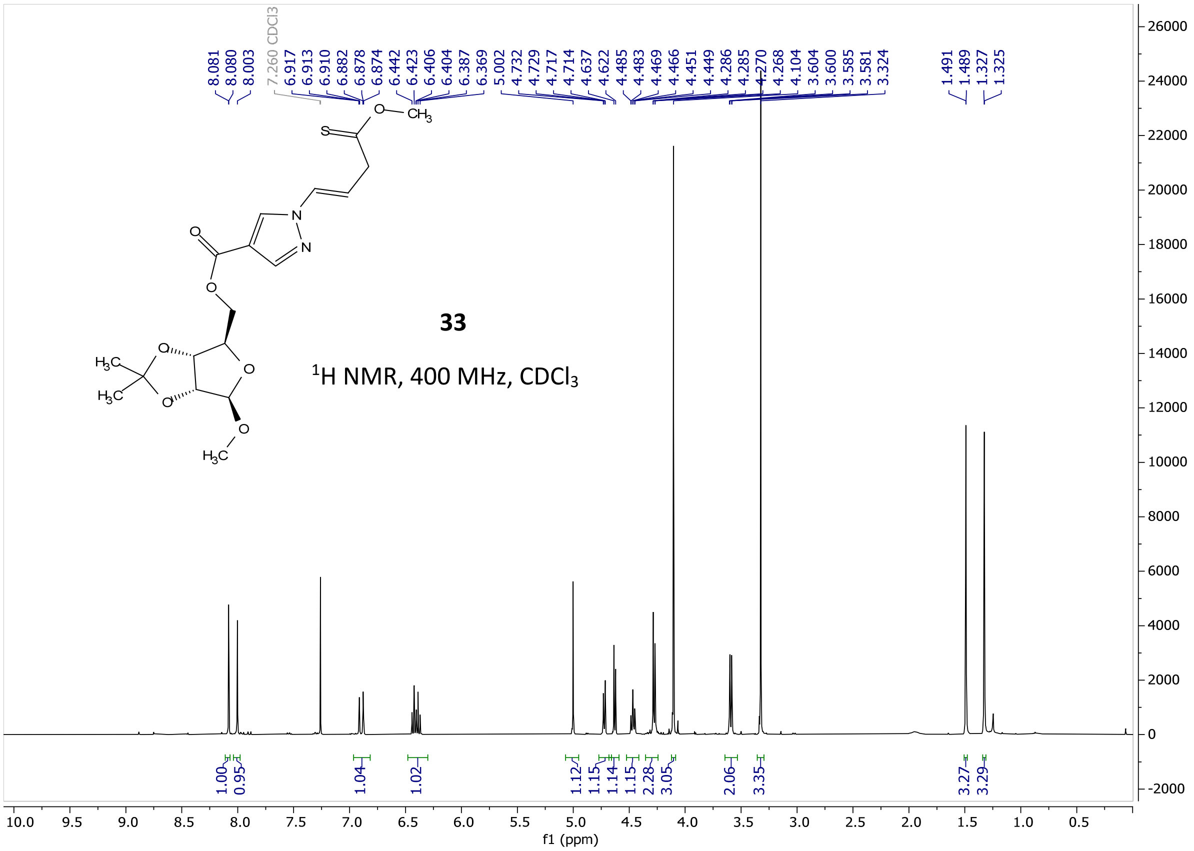

8.081 8.080 8.003 7.260 CDCl3 6.917 6.913 6.910 6.882 6.878 6.874 6.442 6.423 6.406 6.404 6.387 6.369 5.002 4.732 4.729 4.717 4.714 4.637 4.622 4.485 4.483 4.469 4.466 4.451 4.449 4.286 4.285 4.270 4.268 4.104 3.604 3.600 3.585 3.581 3.324 1.491 1.489 1.327 1.325
33
1H NMR, 400 MHz, CDCl3
1.00 0.95 1.04 1.02 1.12 1.15 1.14 1.15 2.28 3.05 2.06 3.35 3.27 3.29
f1 (ppm)

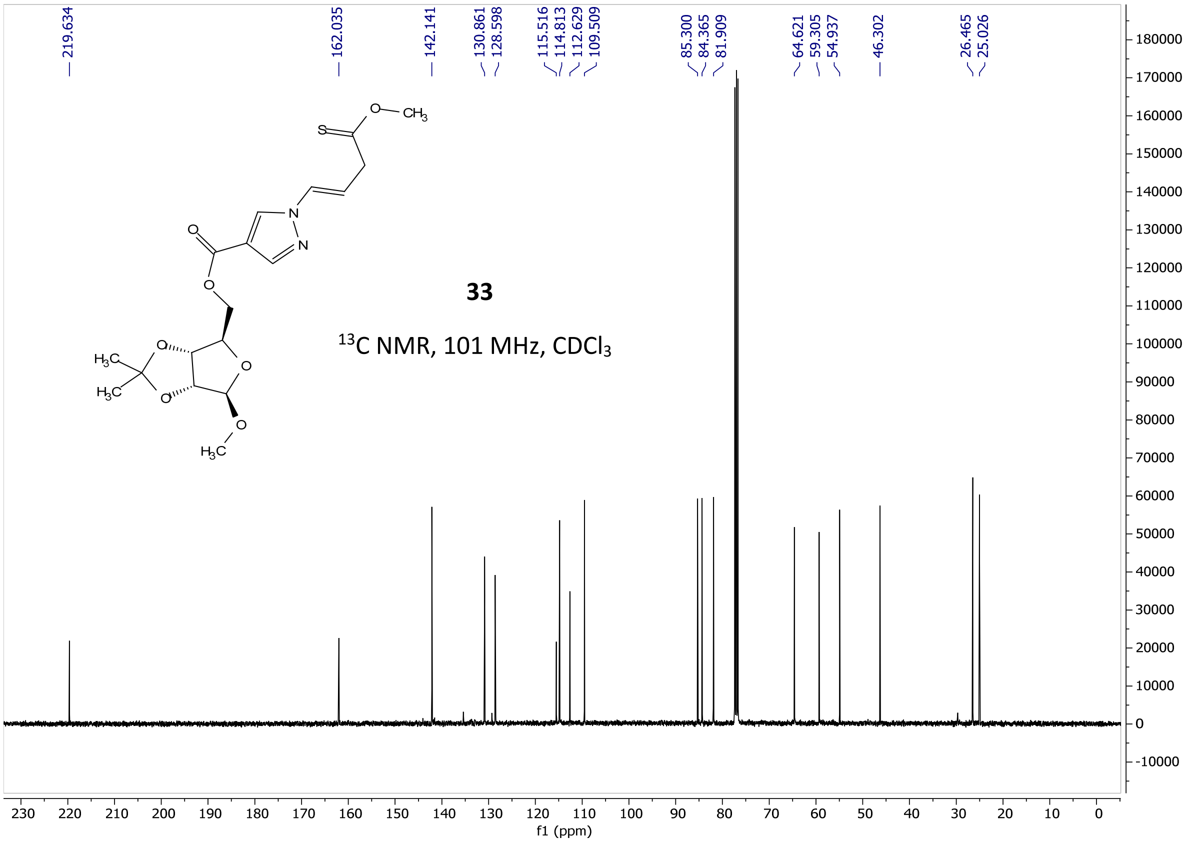

219.634 162.035 142.141 130.861 128.598 115.516 114.813 112.629 109.509 85.300 84.365 81.909 64.621 59.305 54.937 46.302 26.465 25.026
33
13C NMR, 101 MHz, CDCl3
f1 (ppm)

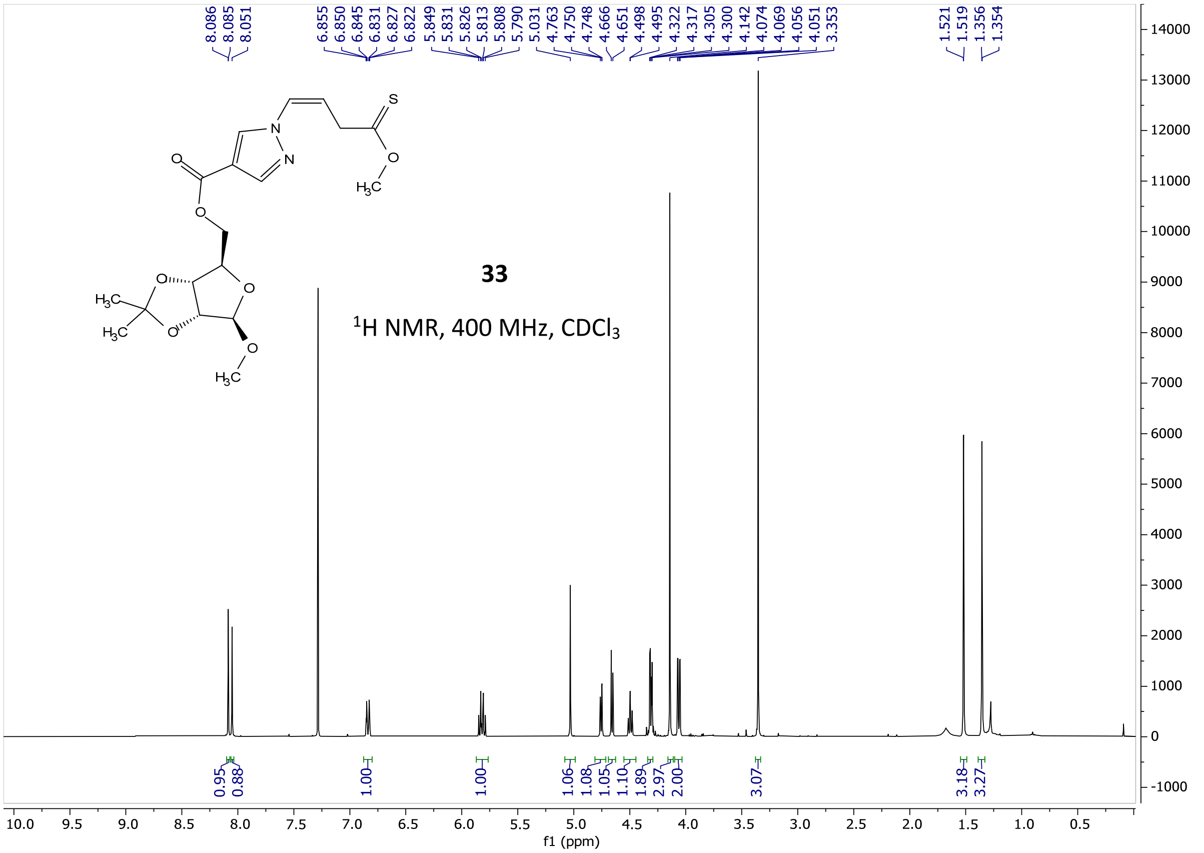
33
^{1}H NMR, 400 MHz, CDCl$_3$
f1 (ppm)

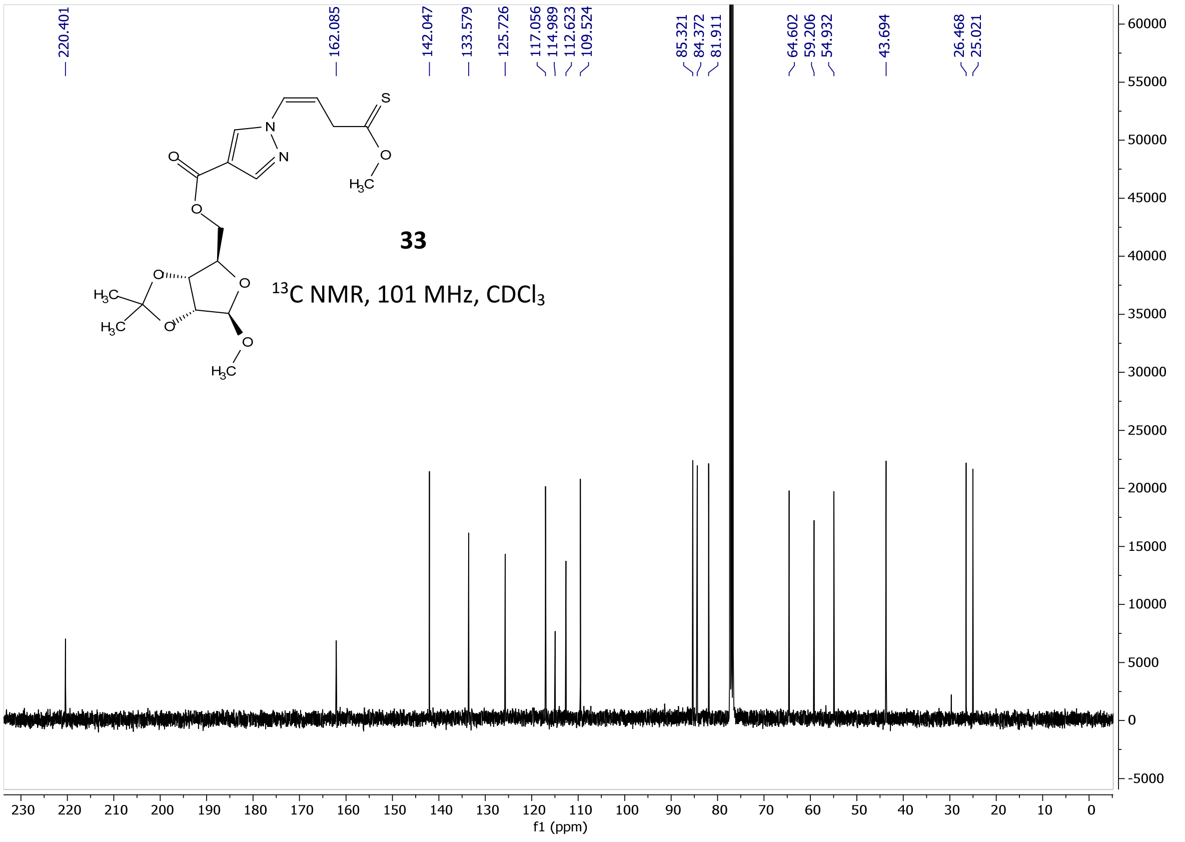
33
^{13}C NMR, 101 MHz, CDCl$_3$
f1 (ppm)

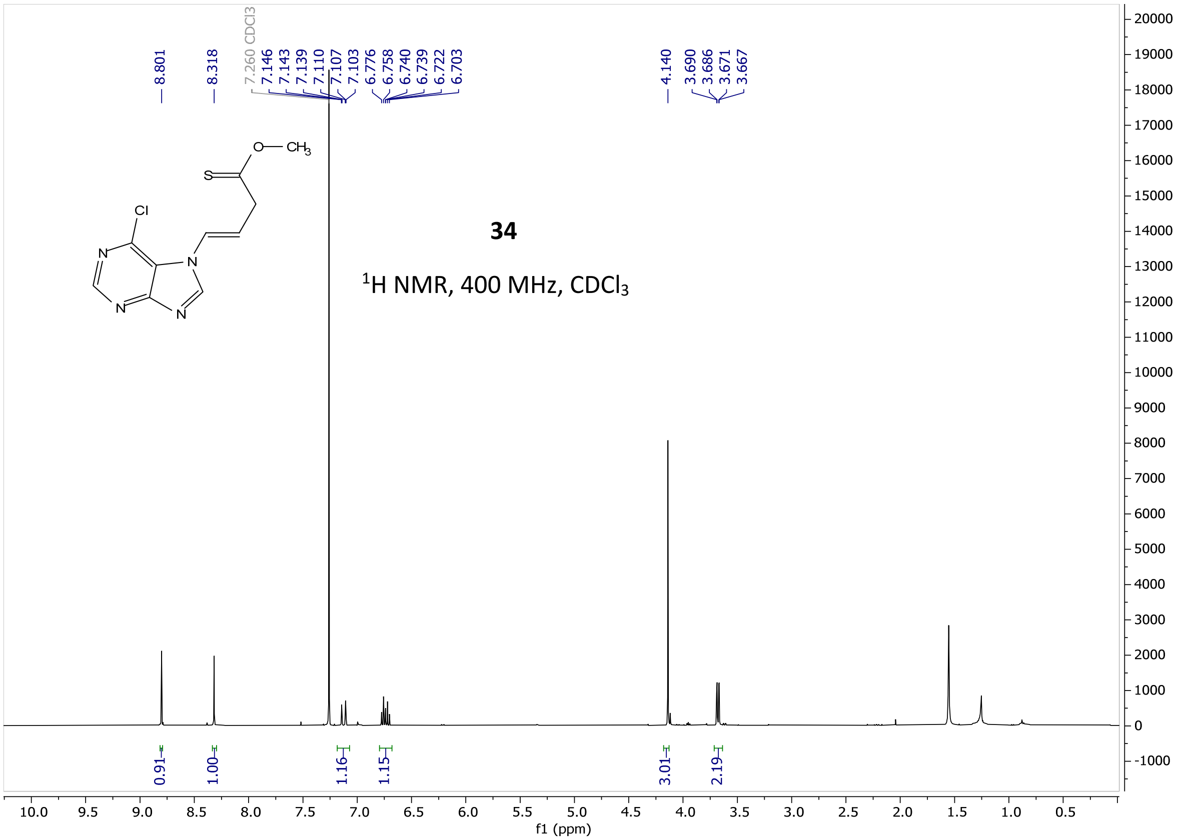

34

^{1}H NMR, 400 MHz, $CDCl_3$

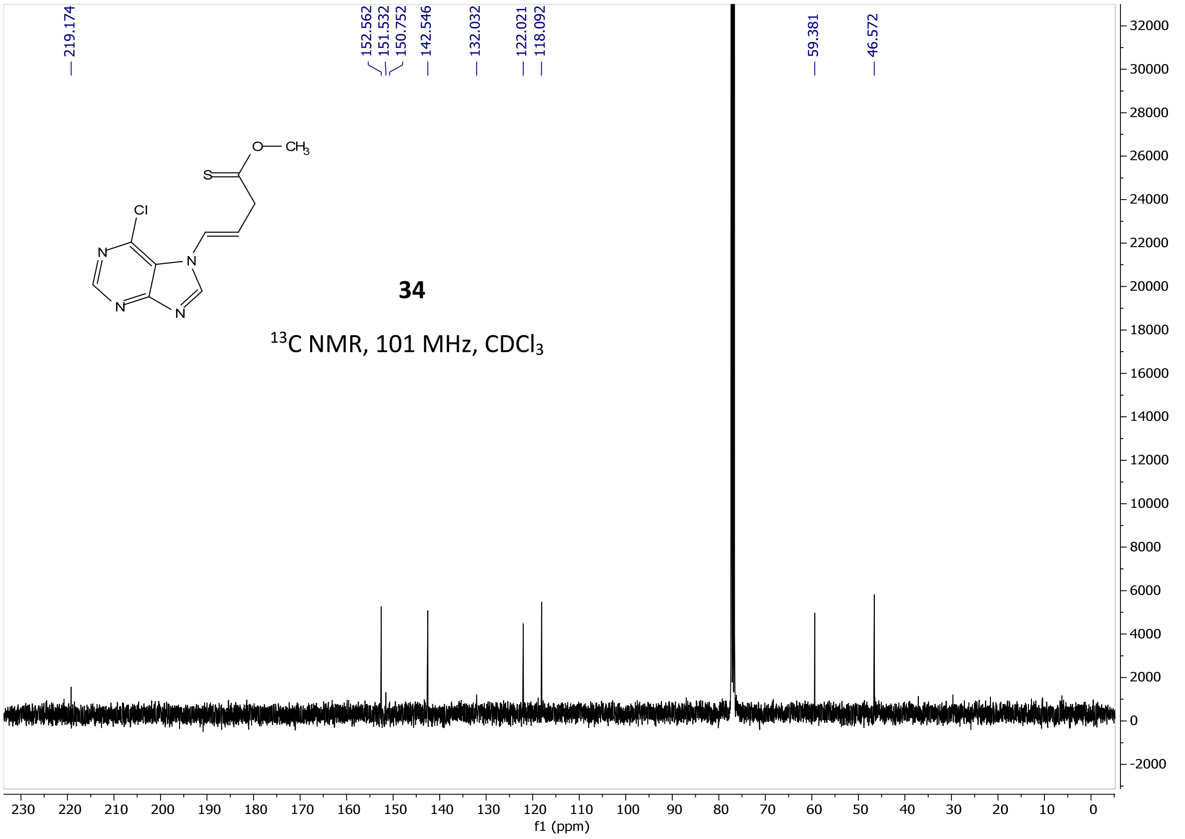

34

^{13}C NMR, 101 MHz, $CDCl_3$

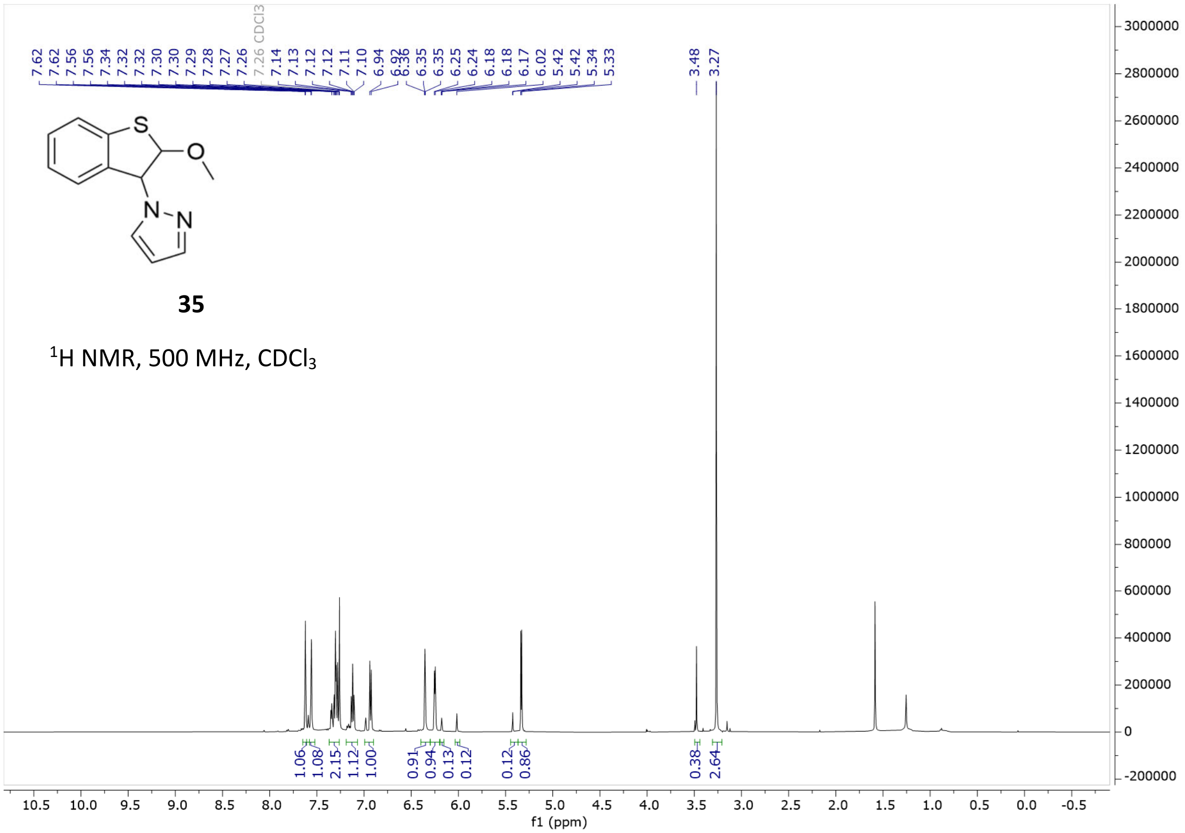
7.62 7.62 7.56 7.34 7.32 7.32 7.30 7.30 7.29 7.28 7.27 7.26 7.26 CDCl3 7.14 7.13 7.12 7.12 7.11 7.10 6.94 6.92 6.36 6.35 6.35 6.25 6.24 6.18 6.18 6.17 6.02 5.42 5.42 5.34 5.33
3.48 3.27
35
1H NMR, 500 MHz, CDCl3
1.06 1.08 2.15 1.12 1.00 0.91 0.94 0.13 0.12 0.12 0.86 0.38 2.64
f1 (ppm)

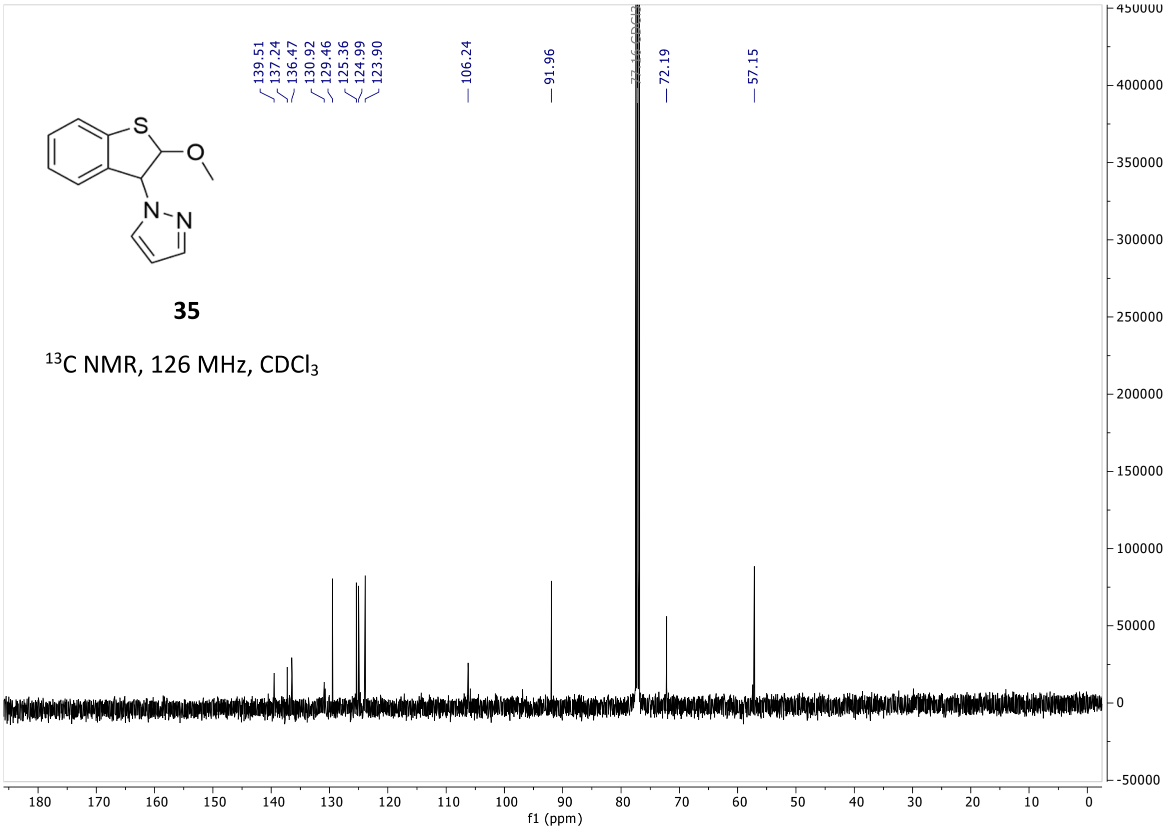
139.51 137.24 136.47 130.92 129.46 125.36 124.99 123.90
106.24
91.96
72.19
57.15
35
13C NMR, 126 MHz, CDCl3
f1 (ppm)

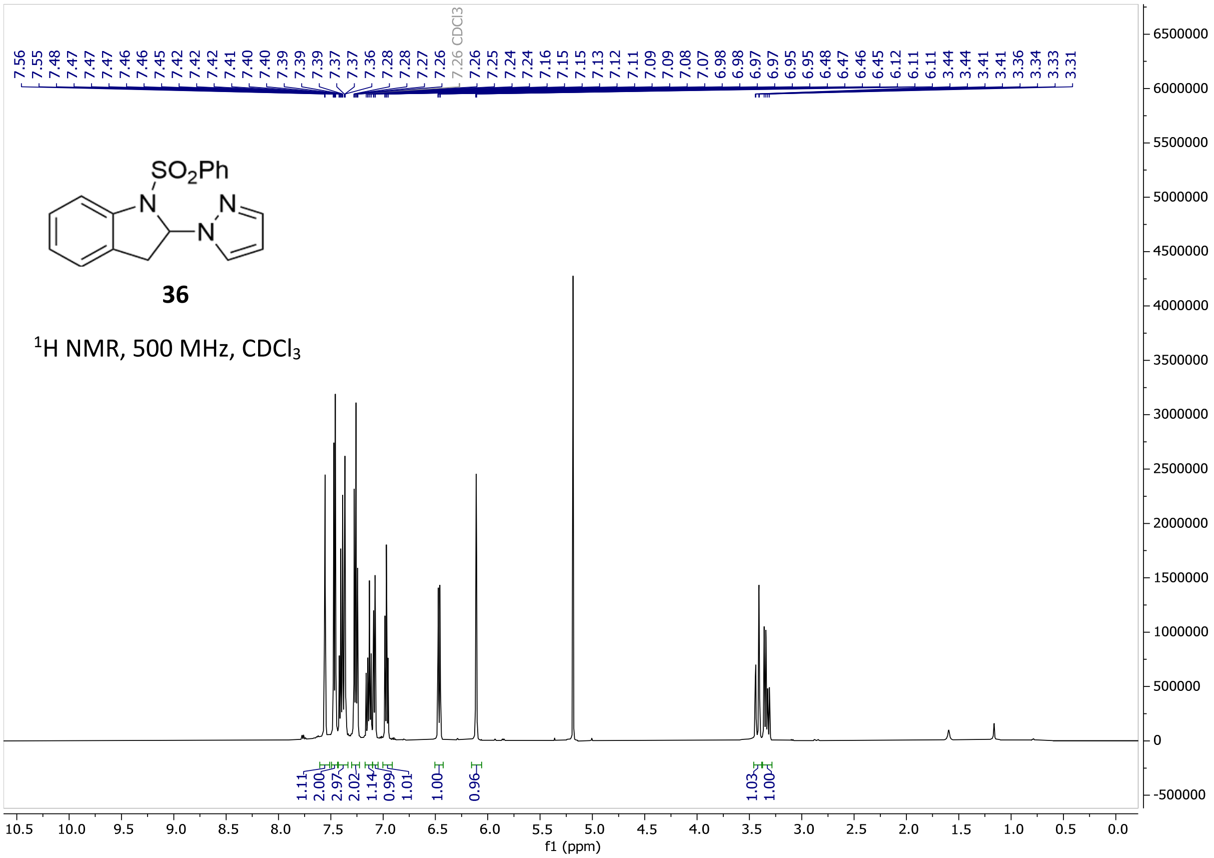
7.56 7.55 7.48 7.47 7.47 7.47 7.46 7.46 7.45 7.42 7.42 7.42 7.41 7.40 7.40 7.39 7.39 7.39 7.37 7.37 7.36 7.28 7.28 7.27 7.26 7.26 CDCl3 7.26 7.25 7.24 7.24 7.16 7.15 7.15 7.13 7.12 7.11 7.09 7.09 7.08 7.07 6.98 6.98 6.97 6.95 6.95 6.48 6.47 6.46 6.45 6.12 6.11 6.11 3.44 3.44 3.41 3.41 3.36 3.34 3.33 3.31
SO2Ph
N
N
N
36
1H NMR, 500 MHz, CDCl3
1.11 2.00 2.97 2.02 1.14 0.99 1.01 1.00 0.96 1.03 1.00
f1 (ppm)

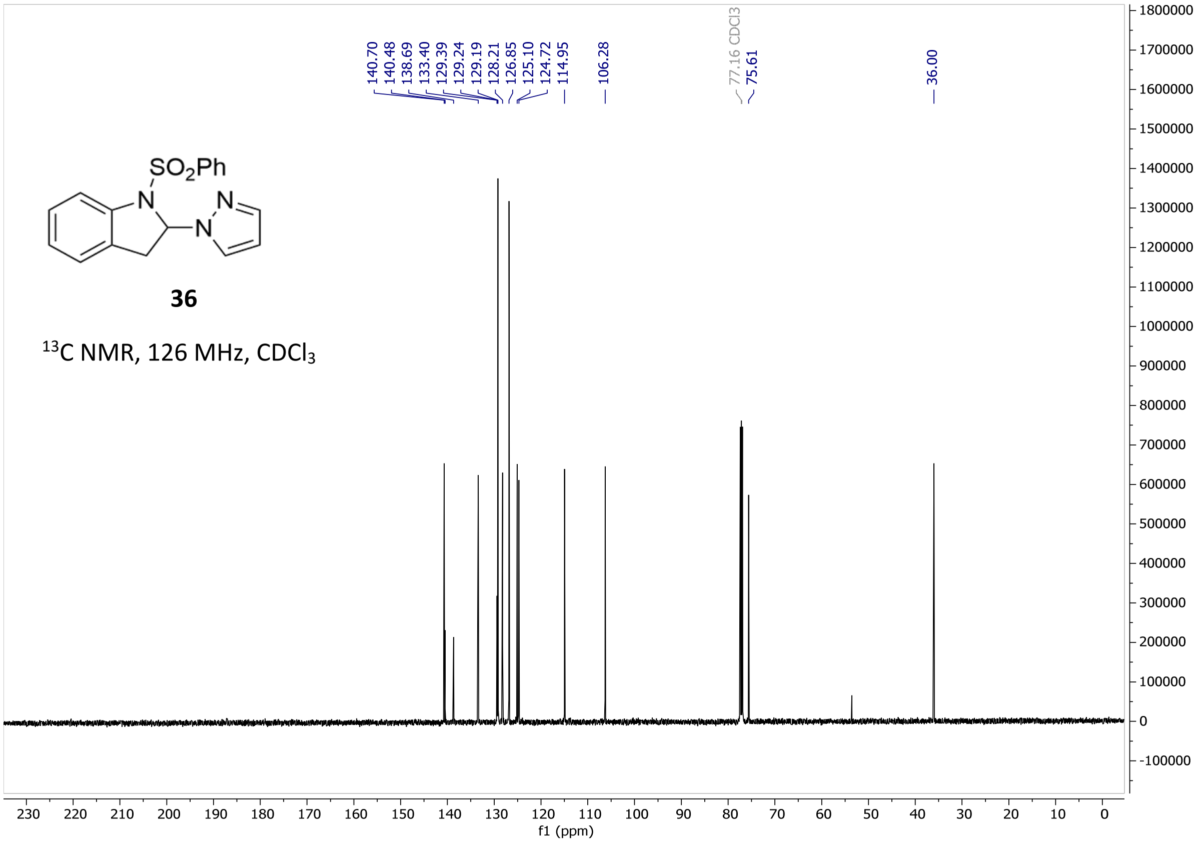
140.70 140.48 138.69 133.40 129.39 129.24 129.19 128.21 126.85 125.10 124.72 114.95 106.28
77.16 CDCl3 75.61
36.00
SO2Ph
N
N
N
36
13C NMR, 126 MHz, CDCl3
f1 (ppm)

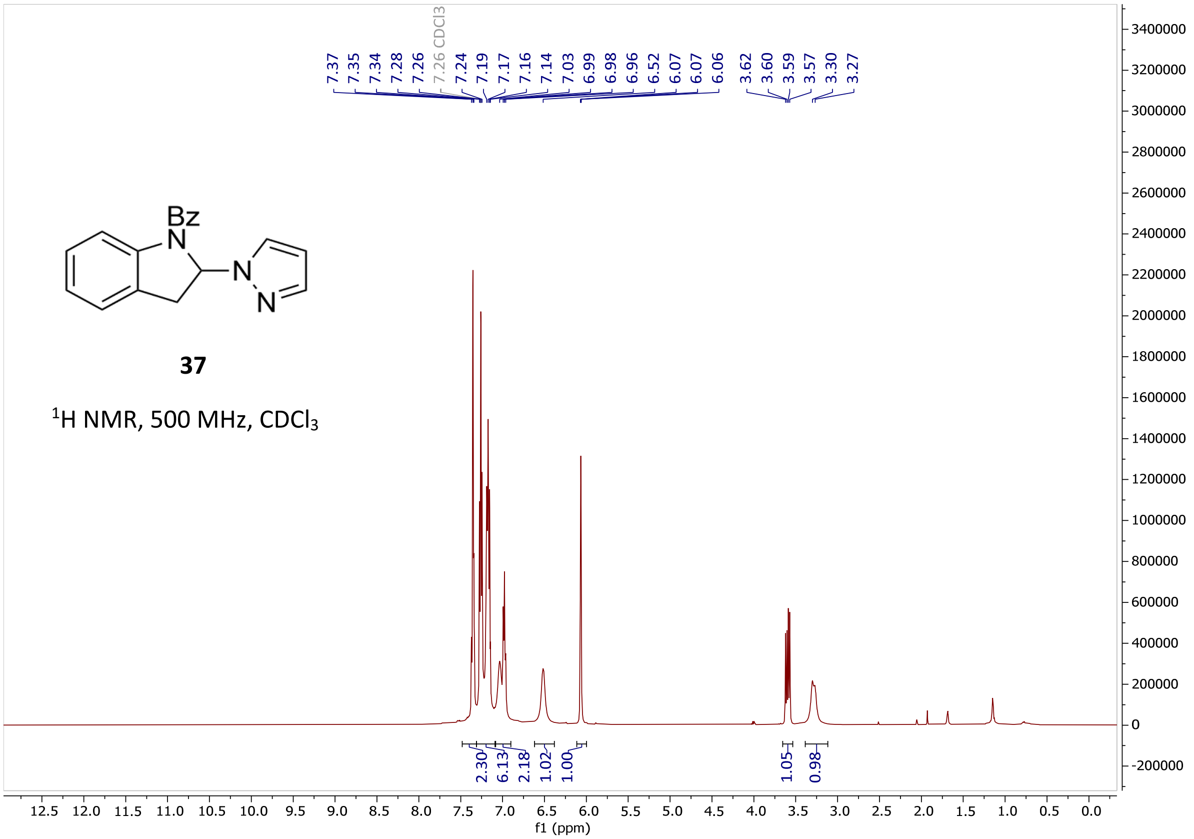
7.37
7.35
7.34
7.28
7.26
7.26 CDCl3
7.24
7.19
7.17
7.16
7.14
7.03
6.99
6.98
6.96
6.52
6.07
6.07
6.06
3.62
3.60
3.59
3.57
3.30
3.27
Bz
N
N
N
37
^{1}H NMR, 500 MHz, $CDCl_3$
2.30
6.13
2.18
1.02
1.00
1.05
0.98
f1 (ppm)

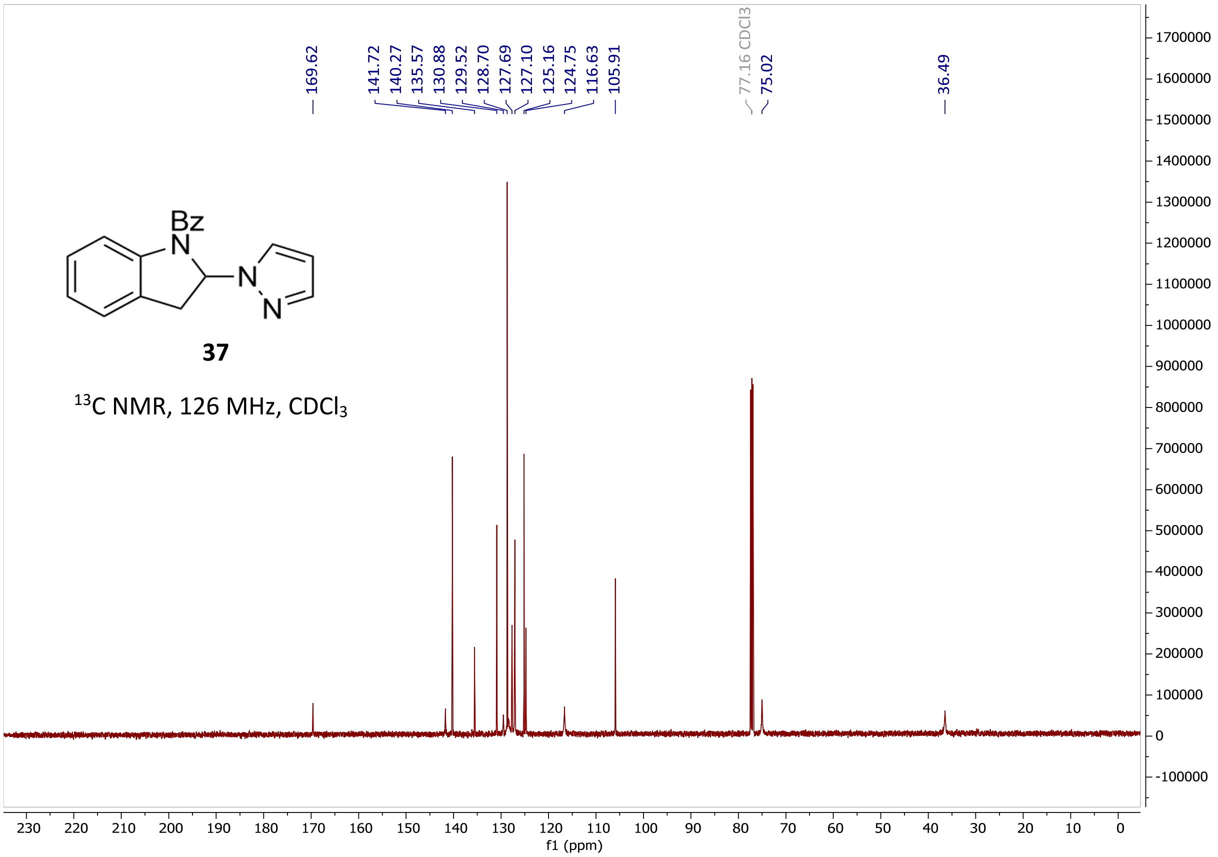
169.62
141.72
140.27
135.57
130.88
129.52
128.70
127.69
127.10
125.16
124.75
116.63
105.91
77.16 CDCl3
75.02
36.49
Bz
N
N
N
37
^{13}C NMR, 126 MHz, $CDCl_3$
f1 (ppm)

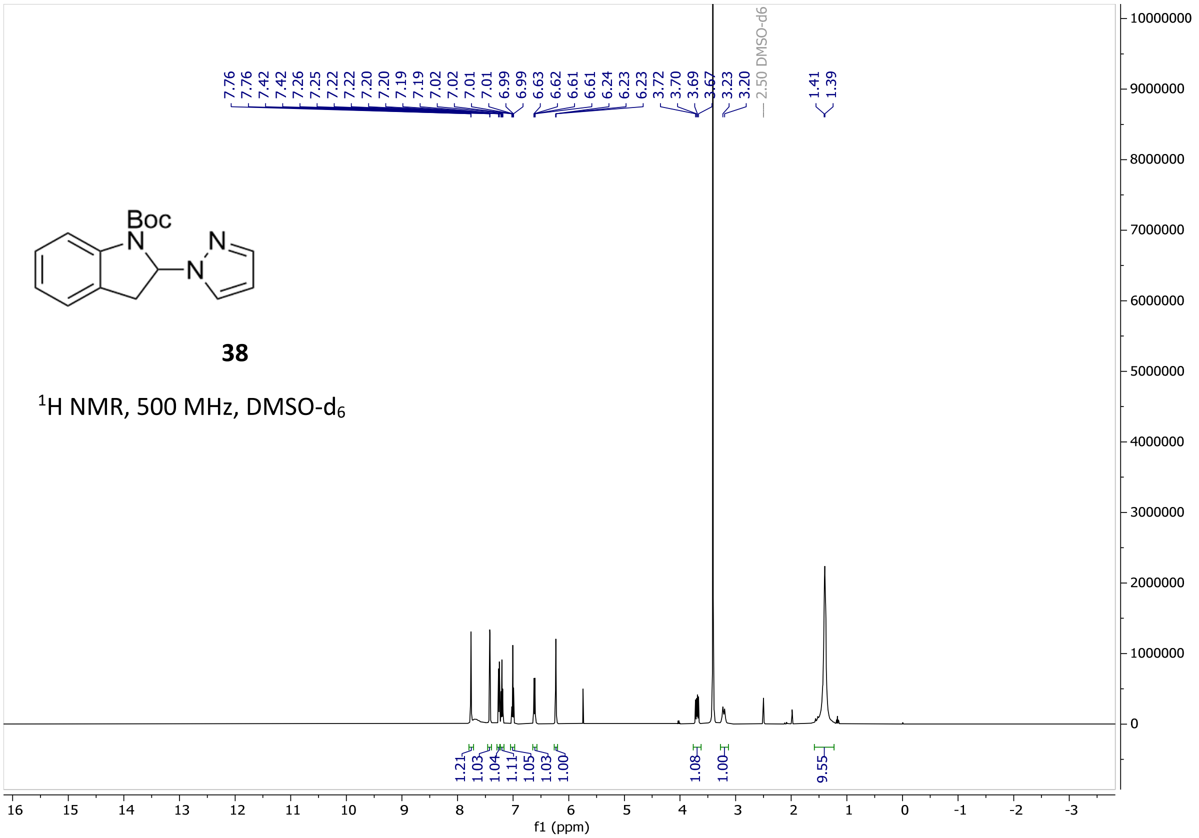
7.76 7.76 7.42 7.42 7.26 7.25 7.22 7.22 7.20 7.20 7.19 7.19 7.02 7.02 7.01 7.01 6.99 6.99 6.63 6.62 6.61 6.61 6.24 6.23 6.23 3.72 3.70 3.69 3.67 3.23 3.20
2.50 DMSO-d6
1.41 1.39
Boc
N
N
N
38
1H NMR, 500 MHz, DMSO-d6
1.21 1.03 1.04 1.11 1.05 1.03 1.00
1.08 1.00
9.55
f1 (ppm)

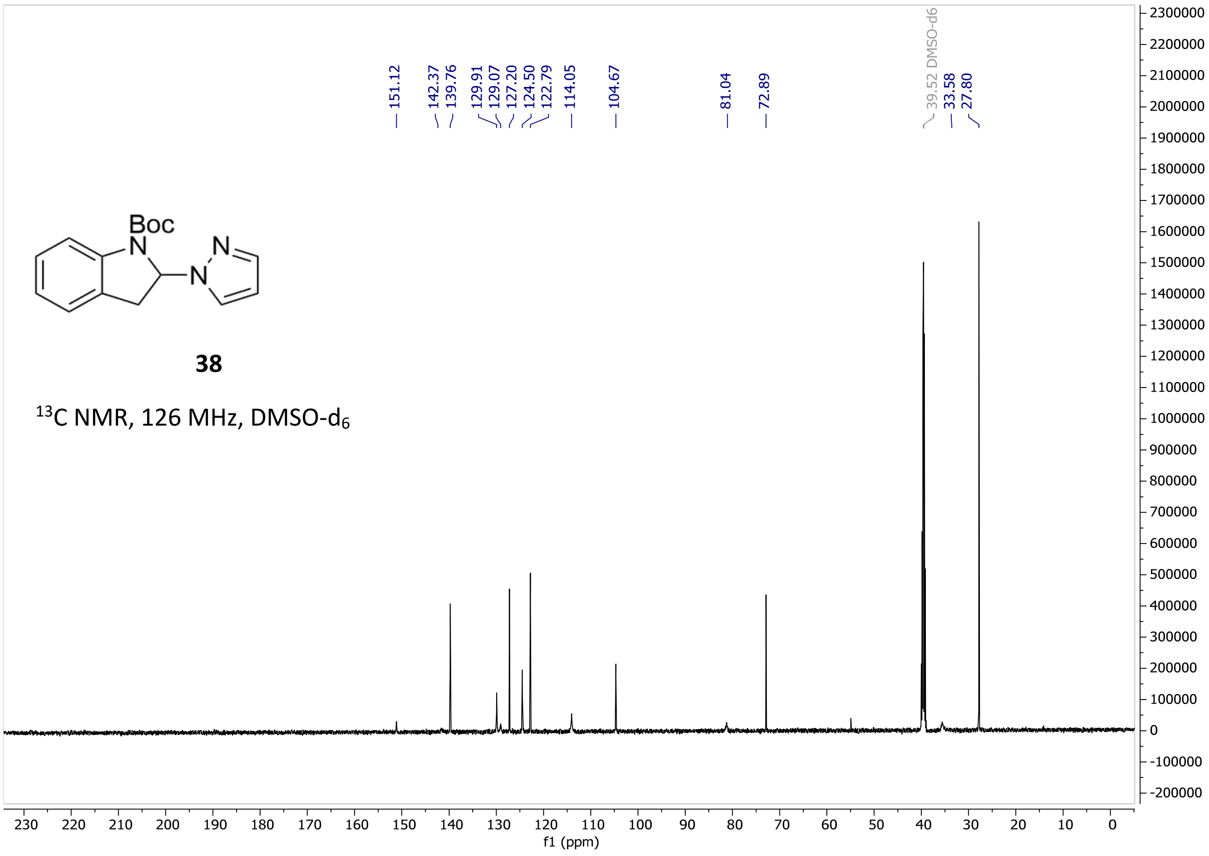
151.12
142.37 139.76
129.91 129.07 127.20 124.50 122.79
114.05
104.67
81.04
72.89
39.52 DMSO-d6
33.58 27.80
Boc
N
N
N
38
13C NMR, 126 MHz, DMSO-d6
f1 (ppm)

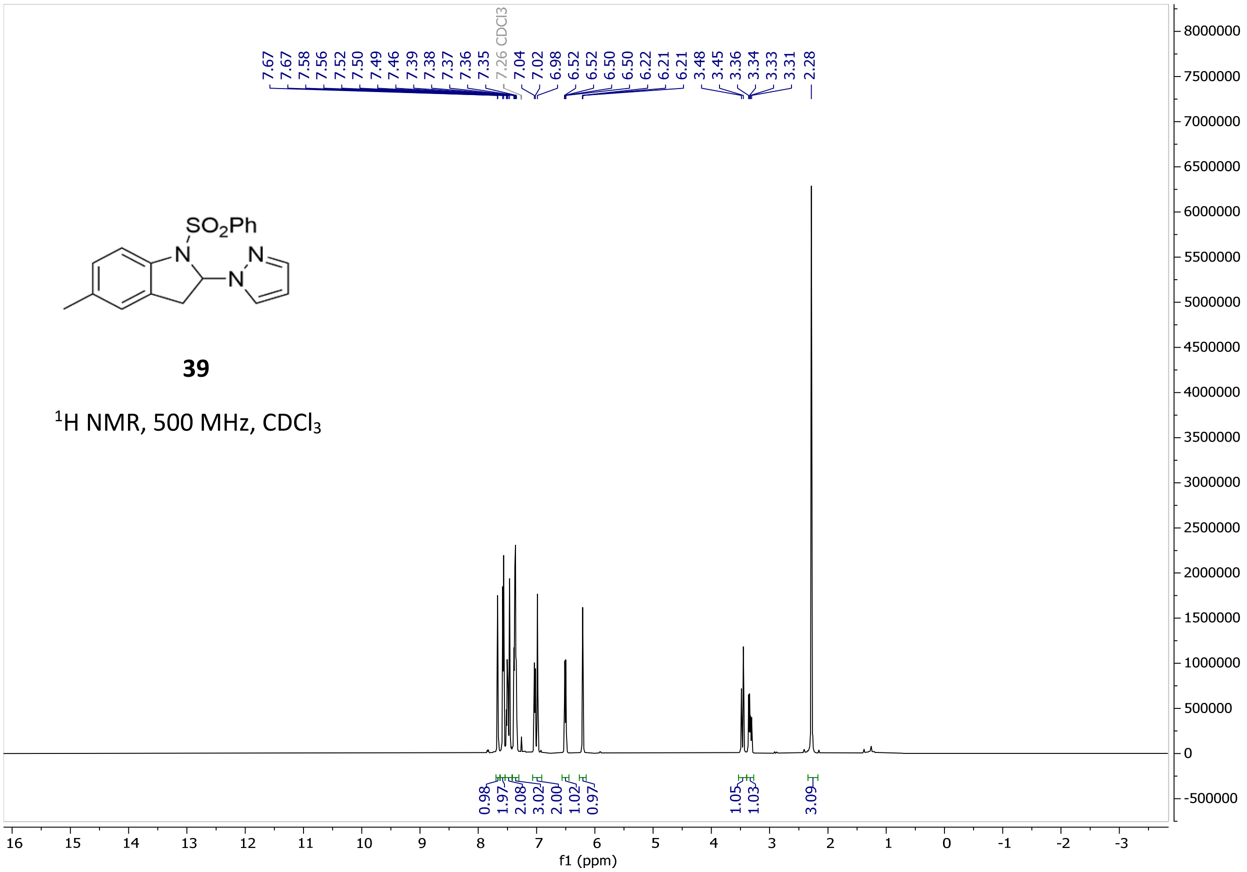
7.67
7.67
7.58
7.56
7.52
7.50
7.49
7.46
7.39
7.38
7.37
7.36
7.35
7.26 CDCl3
7.04
7.02
6.98
6.52
6.52
6.50
6.50
6.22
6.21
6.21
3.48
3.45
3.36
3.34
3.33
3.31
2.28
SO2Ph
N
N
N
39
1H NMR, 500 MHz, CDCl3
0.98
1.97
2.08
3.02
2.00
1.02
0.97
1.05
1.03
3.09
f1 (ppm)

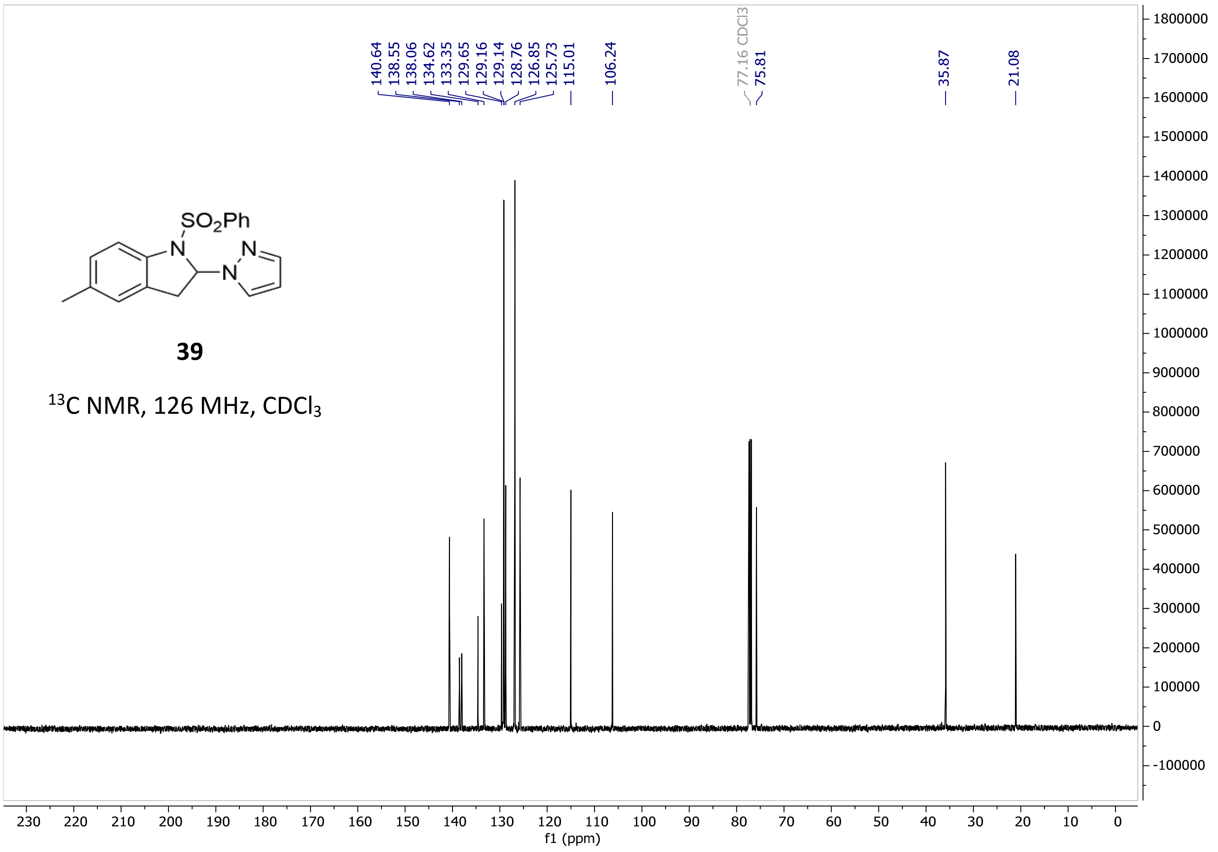
140.64
138.55
138.06
134.62
133.35
129.65
129.16
129.14
128.76
126.85
125.73
115.01
106.24
77.16 CDCl3
75.81
35.87
21.08
SO2Ph
N
N
N
39
13C NMR, 126 MHz, CDCl3
f1 (ppm)

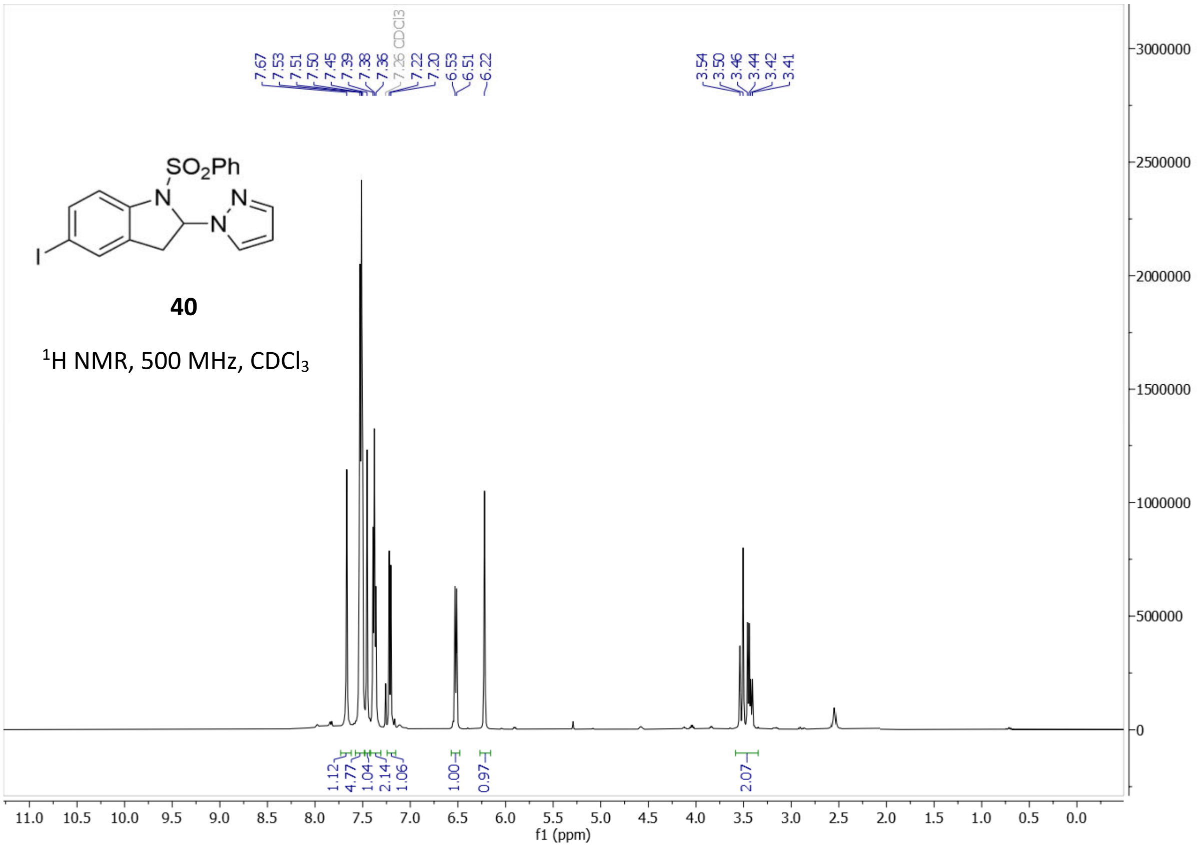
7.67
7.53
7.51
7.50
7.45
7.39
7.38
7.36
7.26 CDCl3
7.22
7.20
6.53
6.51
6.22
3.54
3.50
3.46
3.44
3.42
3.41
SO2Ph
N
N
N
I
40
1H NMR, 500 MHz, CDCl3
1.12
4.77
1.04
2.14
1.06
1.00
0.97
2.07
f1 (ppm)

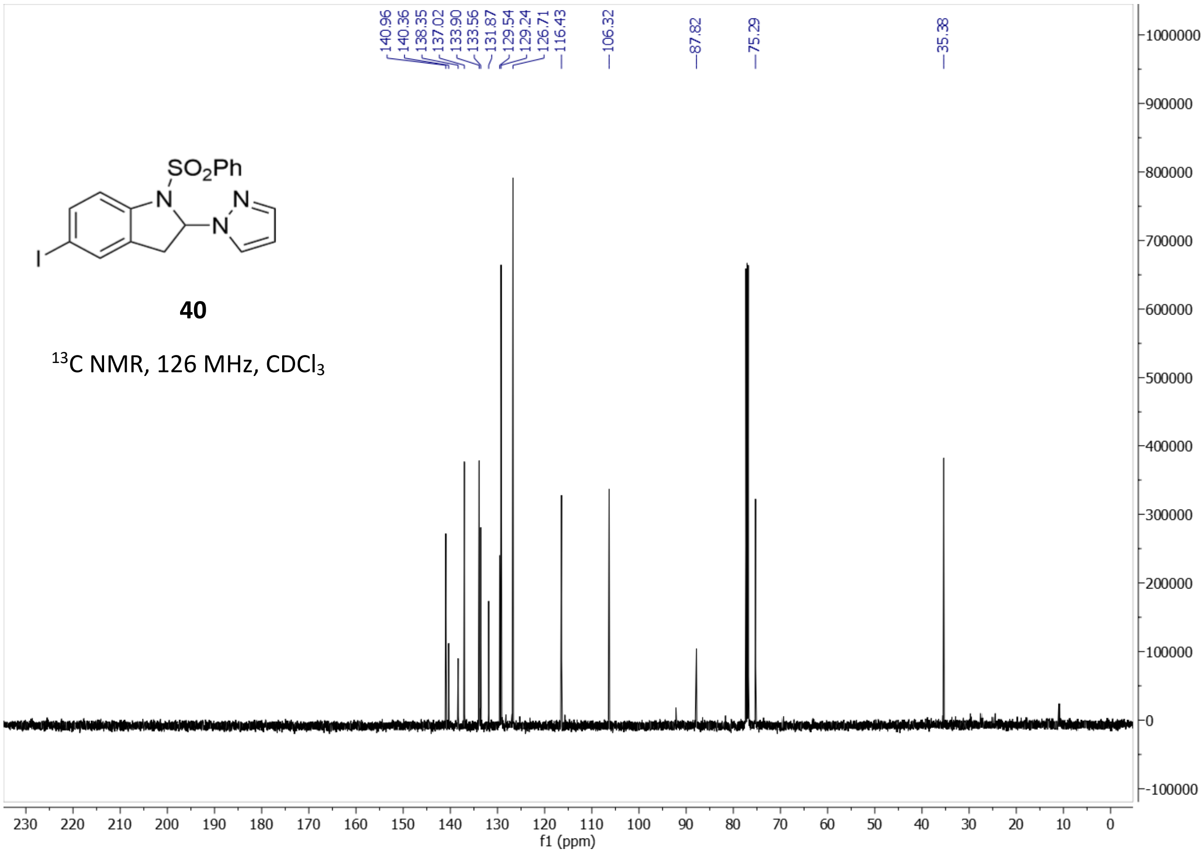
140.96
140.36
138.35
137.02
133.90
133.56
131.87
129.54
129.24
126.71
116.43
106.32
87.82
75.29
35.38
SO2Ph
N
N
N
I
40
13C NMR, 126 MHz, CDCl3
f1 (ppm)

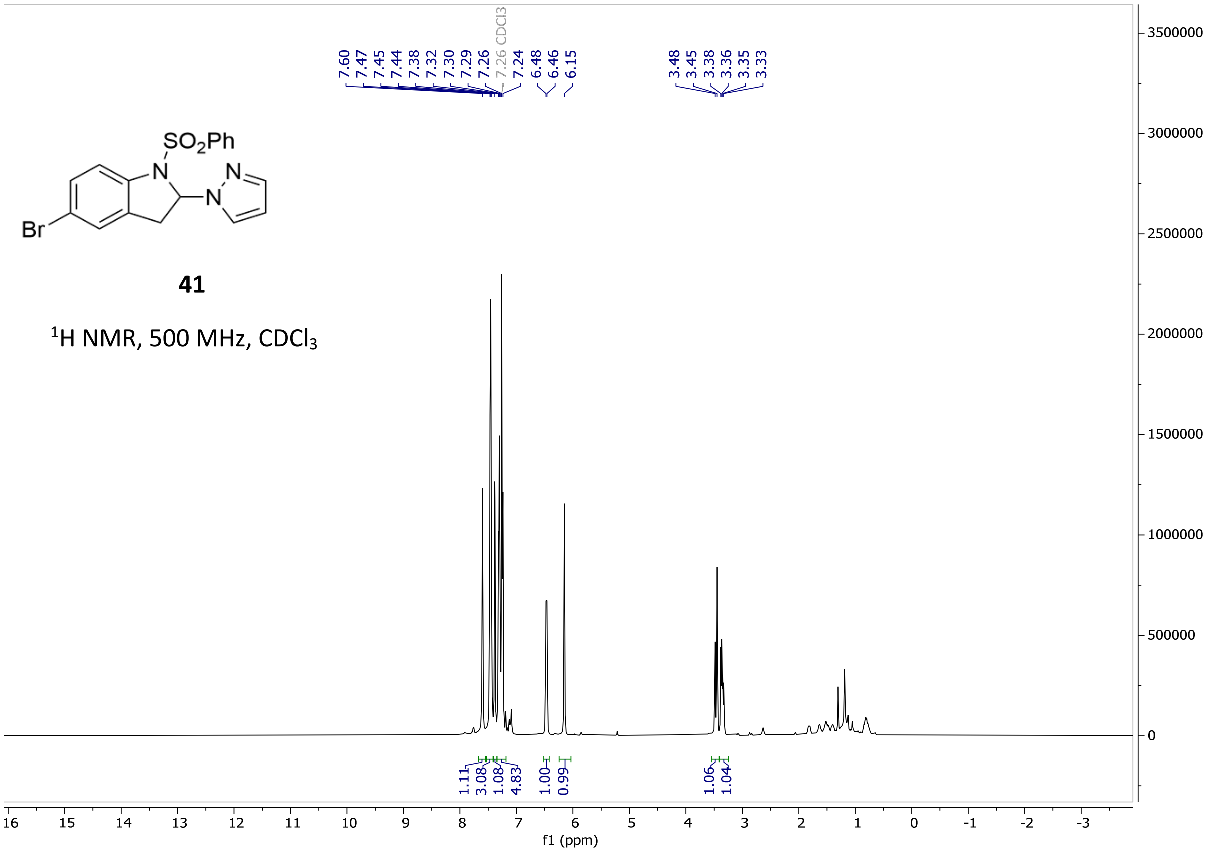
SO2Ph
N
N
N
Br
41
1H NMR, 500 MHz, CDCl3
7.60
7.47
7.45
7.44
7.38
7.32
7.30
7.29
7.26
7.26 CDCl3
7.24
6.48
6.46
6.15
3.48
3.45
3.38
3.36
3.35
3.33
1.11
3.08
1.08
4.83
1.00
0.99
1.06
1.04
f1 (ppm)

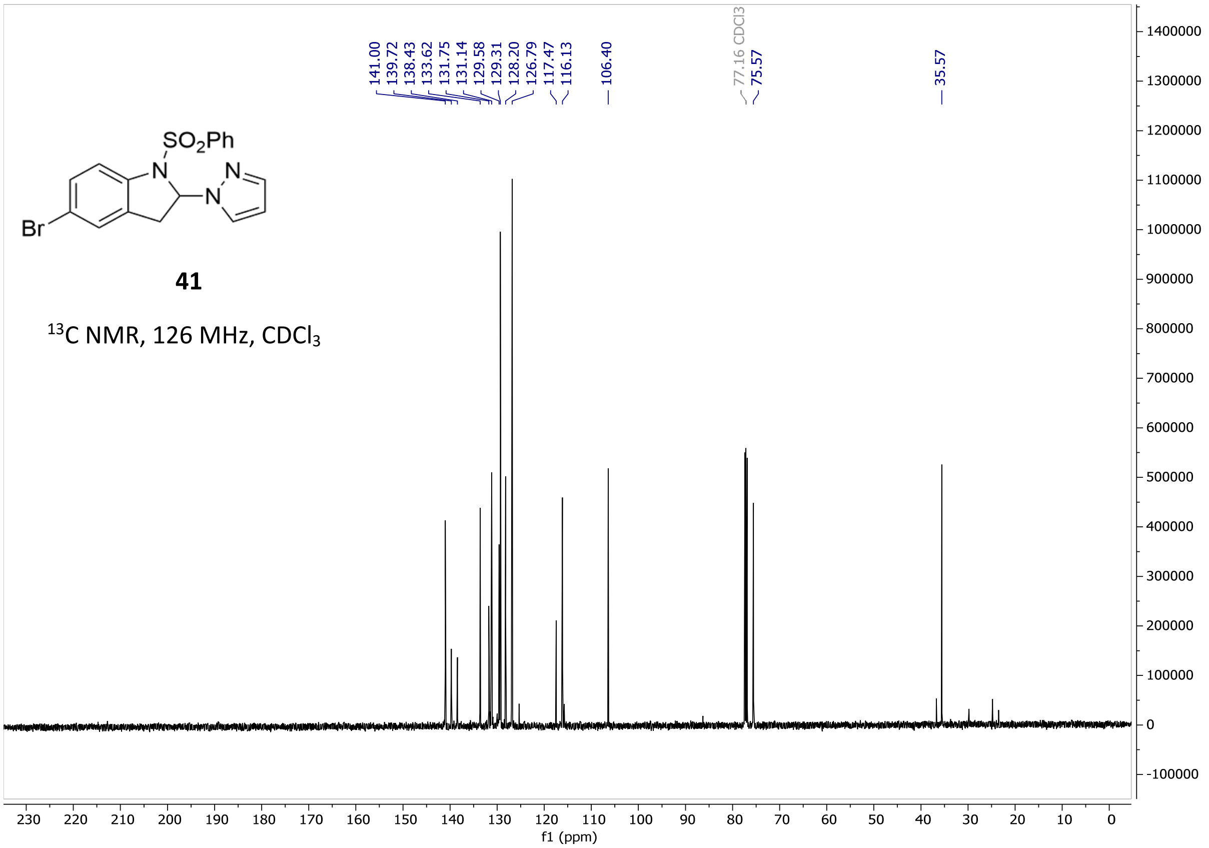
SO2Ph
N
N
N
Br
41
13C NMR, 126 MHz, CDCl3
141.00
139.72
138.43
133.62
131.75
131.14
129.58
129.31
128.20
126.79
117.47
116.13
106.40
77.16 CDCl3
75.57
35.57
f1 (ppm)

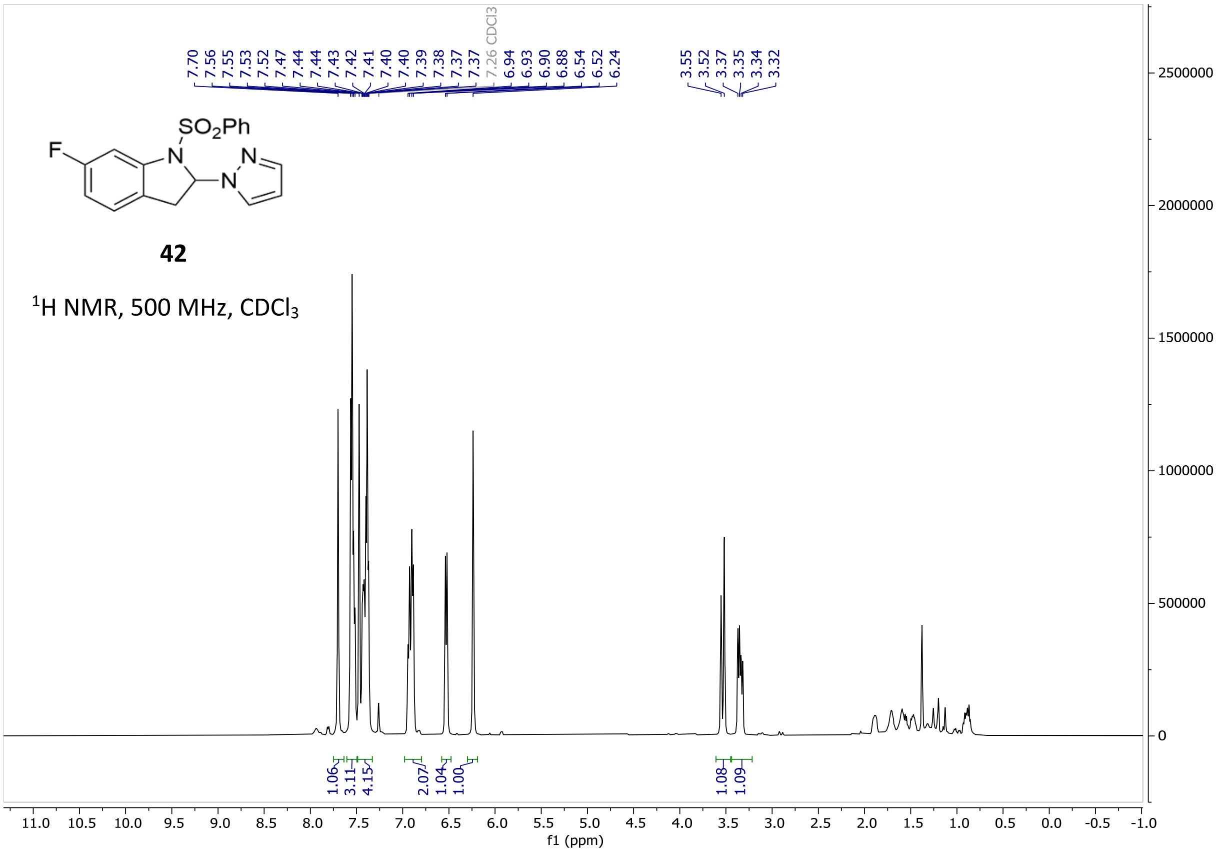
7.70 7.56 7.55 7.53 7.52 7.47 7.44 7.44 7.43 7.42 7.41 7.40 7.40 7.39 7.38 7.37 7.37 7.26 CDCl3 6.94 6.93 6.90 6.88 6.54 6.52 6.24
3.55 3.52 3.37 3.35 3.34 3.32
SO2Ph
F
N
N
N
42
1H NMR, 500 MHz, CDCl3
1.06 3.11 4.15 2.07 1.04 1.00 1.08 1.09
f1 (ppm)

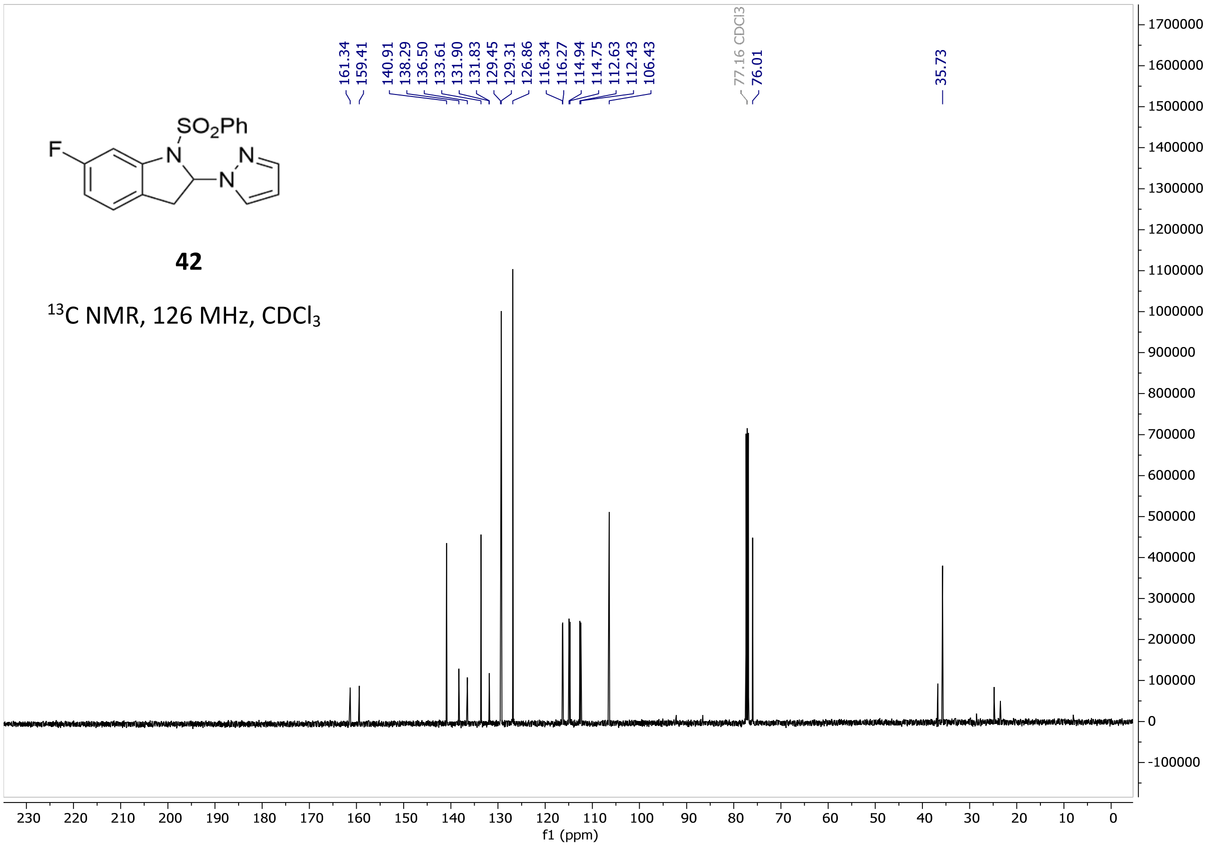
161.34 159.41 140.91 138.29 136.50 133.61 131.90 131.83 129.45 129.31 126.86 116.34 116.27 114.94 114.75 112.63 112.43 106.43
77.16 CDCl3 76.01
35.73
SO2Ph
F
N
N
N
42
13C NMR, 126 MHz, CDCl3
f1 (ppm)

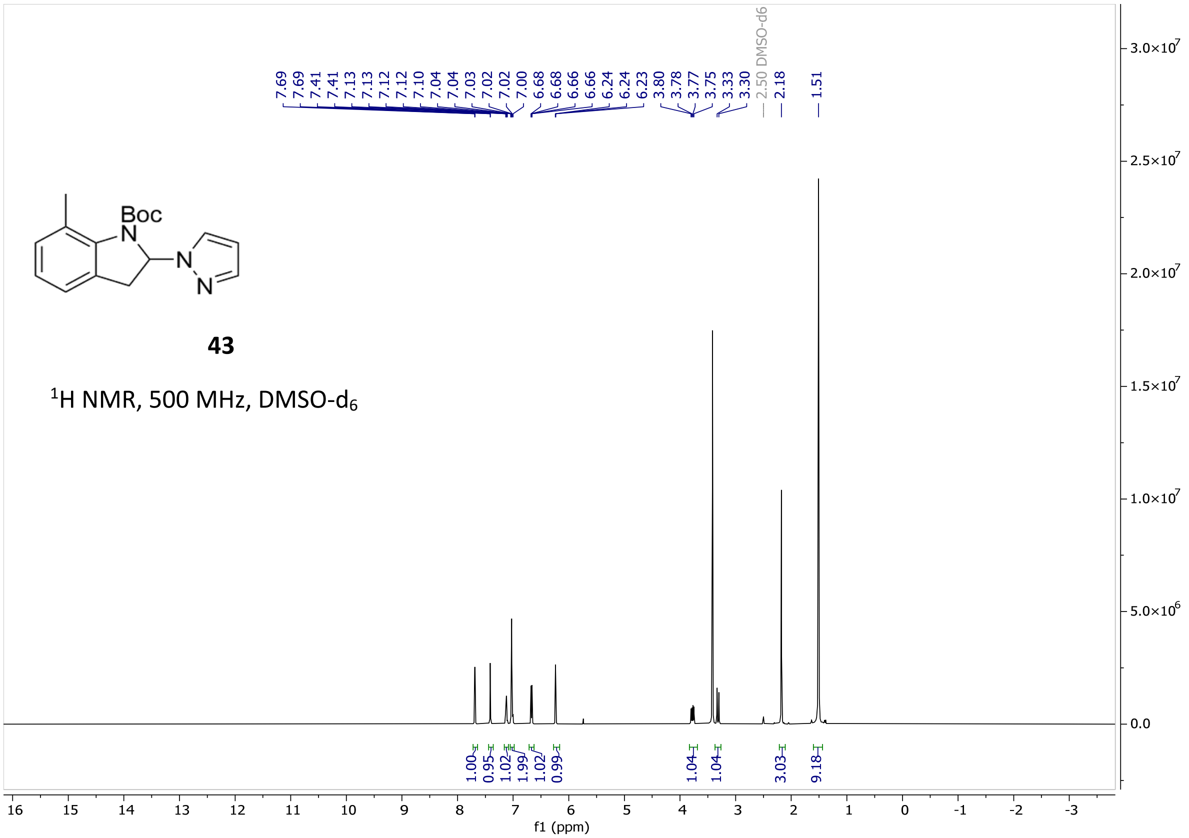

Boc
N
N
N
43
^{1}H NMR, 500 MHz, DMSO-d$_6$
7.69
7.69
7.41
7.41
7.13
7.13
7.12
7.12
7.10
7.04
7.04
7.03
7.02
7.02
7.00
6.68
6.68
6.66
6.66
6.24
6.24
6.23
3.80
3.78
3.77
3.75
3.33
3.30
2.50 DMSO-d6
2.18
1.51
1.00
0.95
1.02
1.99
1.02
0.99
1.04
1.04
3.03
9.18
f1 (ppm)

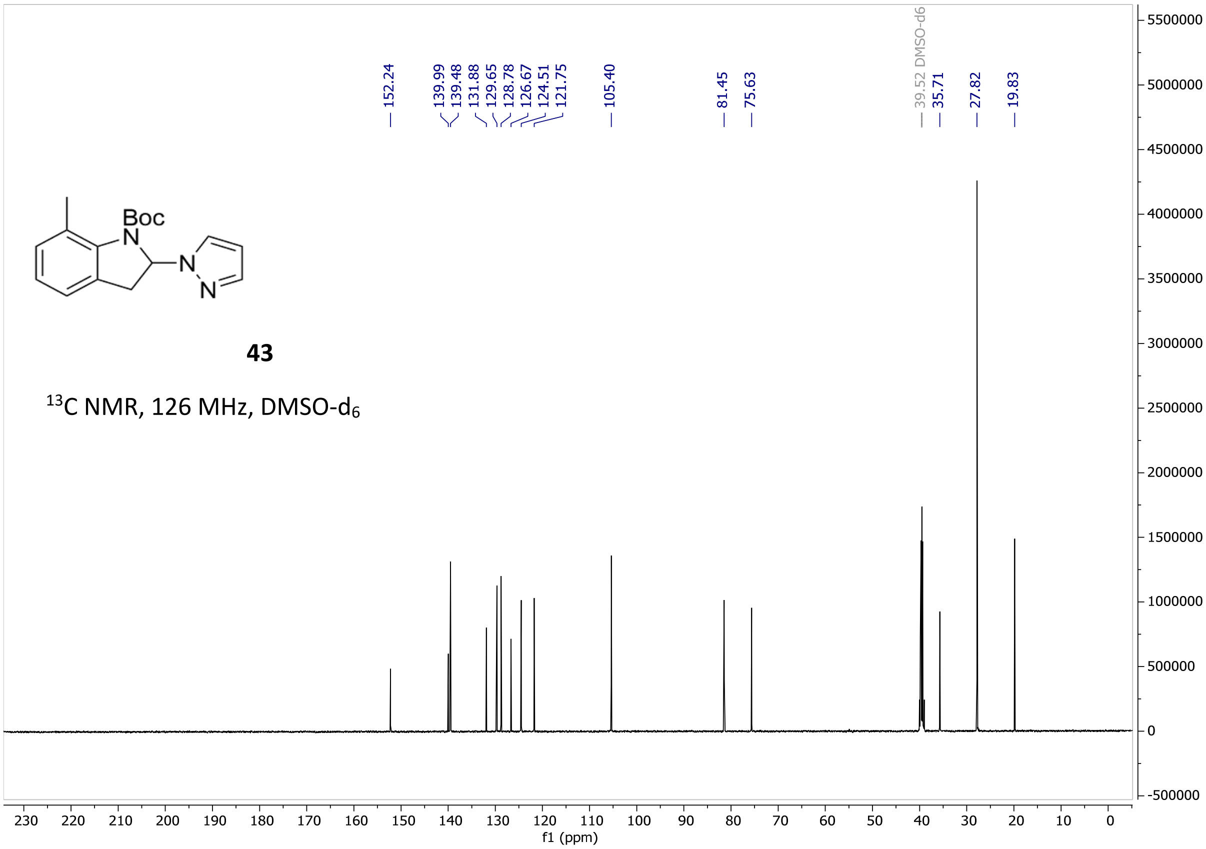

Boc
N
N
N
43
^{13}C NMR, 126 MHz, DMSO-d$_6$
152.24
139.99
139.48
131.88
129.65
128.78
126.67
124.51
121.75
105.40
81.45
75.63
39.52 DMSO-d6
35.71
27.82
19.83
f1 (ppm)

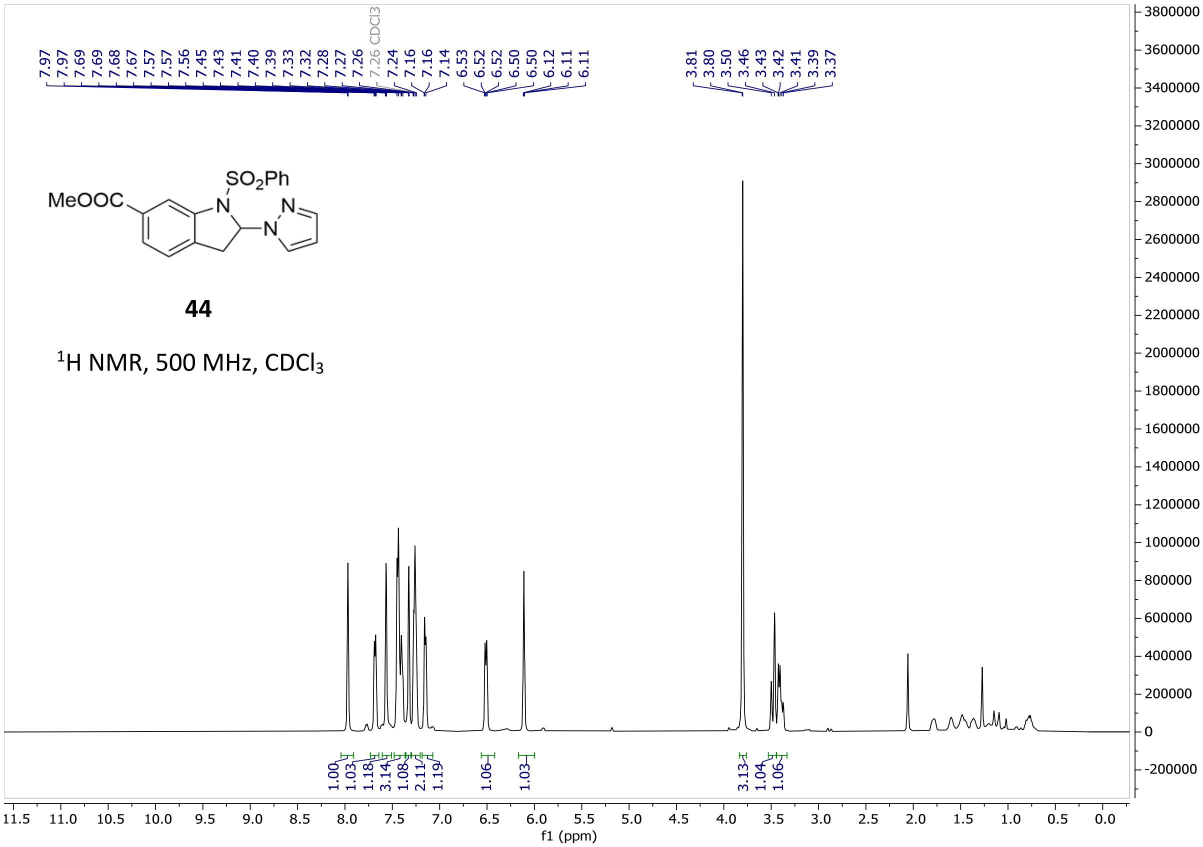
7.97 7.97 7.69 7.69 7.68 7.67 7.57 7.57 7.56 7.45 7.43 7.41 7.40 7.39 7.33 7.32 7.28 7.27 7.26 7.24 7.16 7.16 7.14 6.53 6.52 6.52 6.50 6.50 6.12 6.11 6.11
7.26 CDCl3
3.81 3.80 3.50 3.46 3.43 3.42 3.41 3.39 3.37
MeOOC
SO2Ph
N
N
N
44
1H NMR, 500 MHz, CDCl3
1.00 1.03 1.18 3.14 1.08 2.11 1.19 1.06 1.03 3.13 1.04 1.06
f1 (ppm)

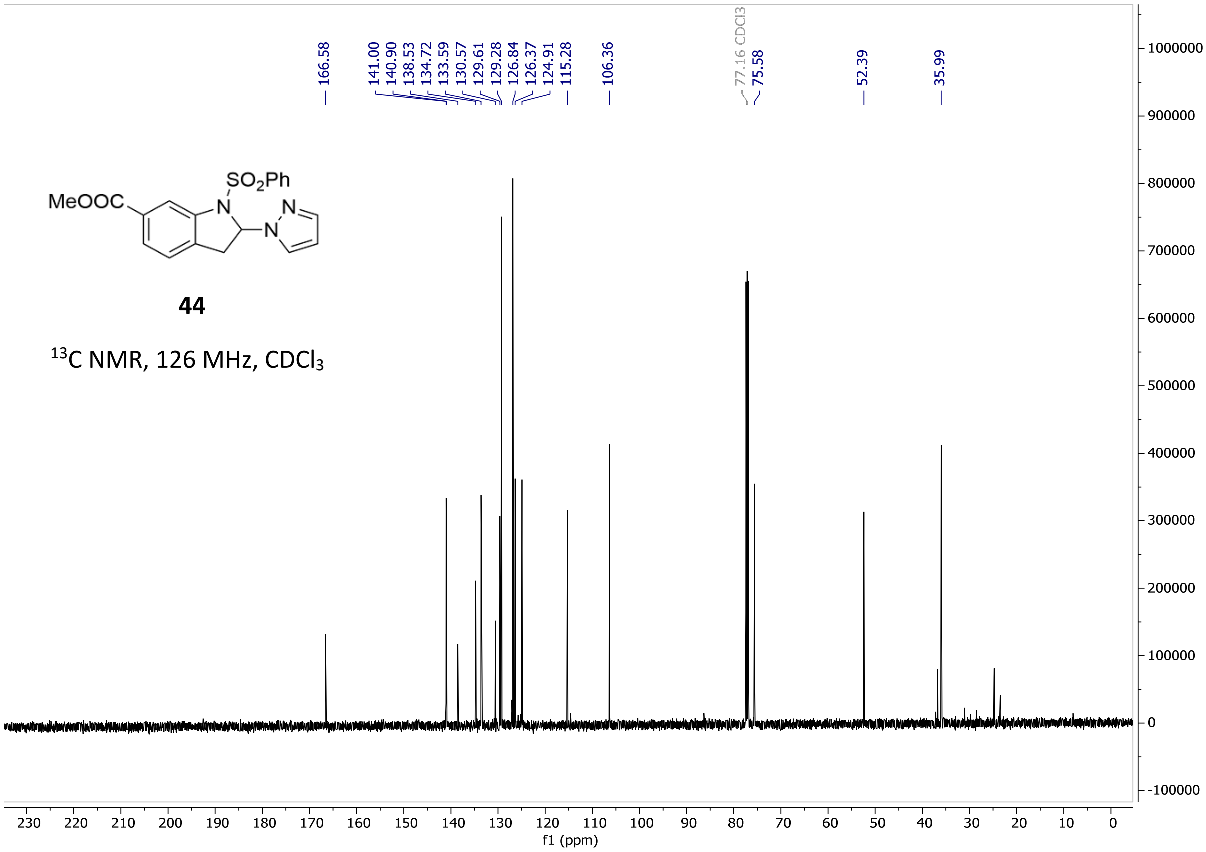
166.58
141.00 140.90 138.53 134.72 133.59 130.57 129.61 129.28 126.84 126.37 124.91 115.28
106.36
77.16 CDCl3
75.58
52.39
35.99
MeOOC
SO2Ph
N
N
N
44
13C NMR, 126 MHz, CDCl3
f1 (ppm)

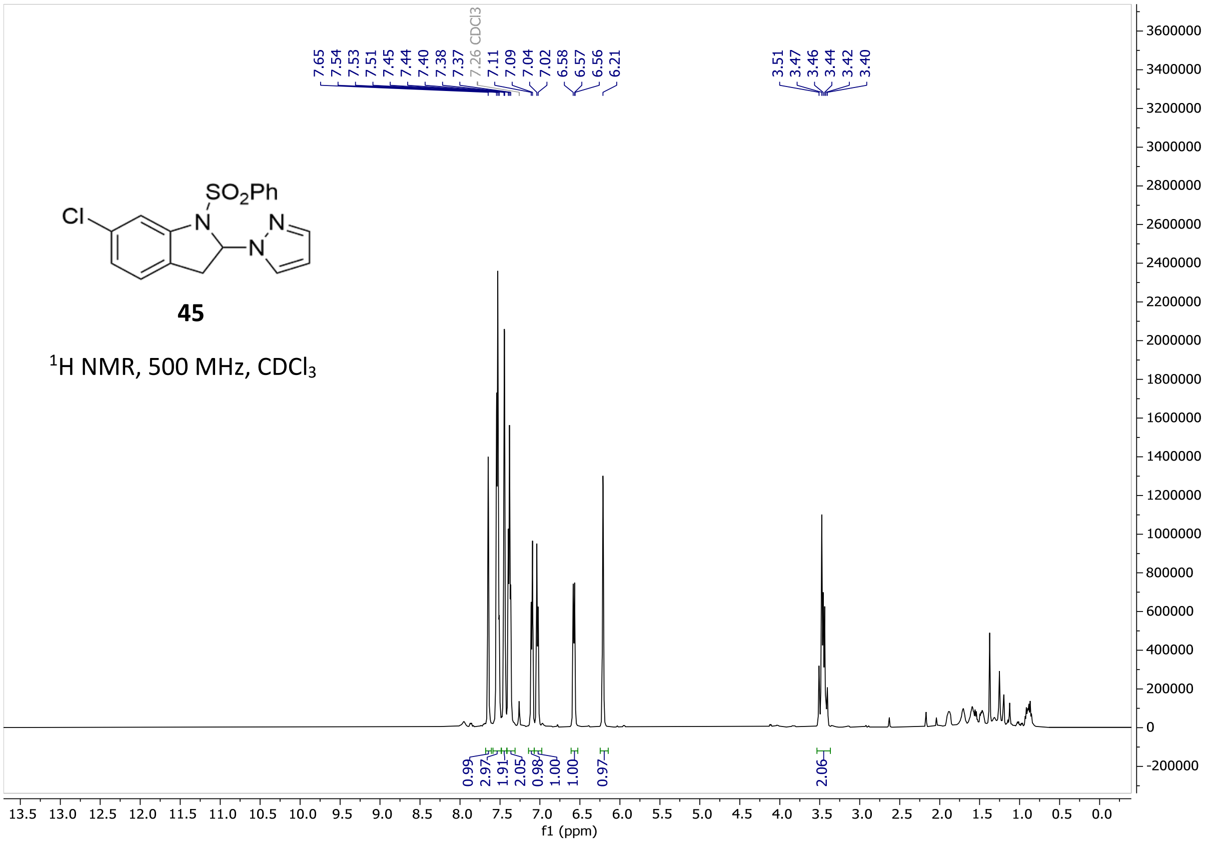
7.65
7.54
7.53
7.51
7.45
7.44
7.40
7.38
7.37
7.26 CDCl3
7.11
7.09
7.04
7.02
6.58
6.57
6.56
6.21
3.51
3.47
3.46
3.44
3.42
3.40
SO2Ph
Cl
N
N
N
45
1H NMR, 500 MHz, CDCl3
0.99
2.97
1.91
2.05
0.98
1.00
1.00
0.97
2.06
f1 (ppm)

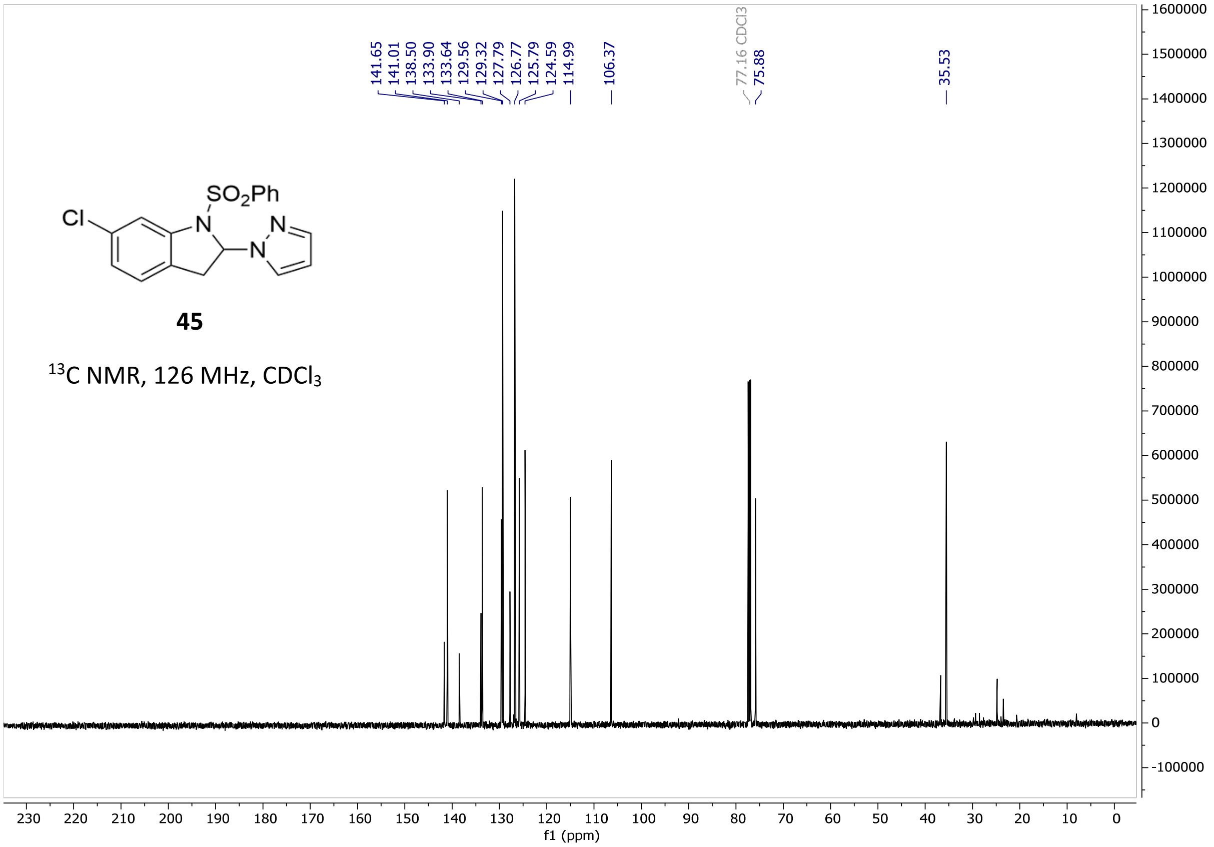
141.65
141.01
138.50
133.90
133.64
129.56
129.32
127.79
126.77
125.79
124.59
114.99
106.37
77.16 CDCl3
75.88
35.53
SO2Ph
Cl
N
N
N
45
13C NMR, 126 MHz, CDCl3
f1 (ppm)

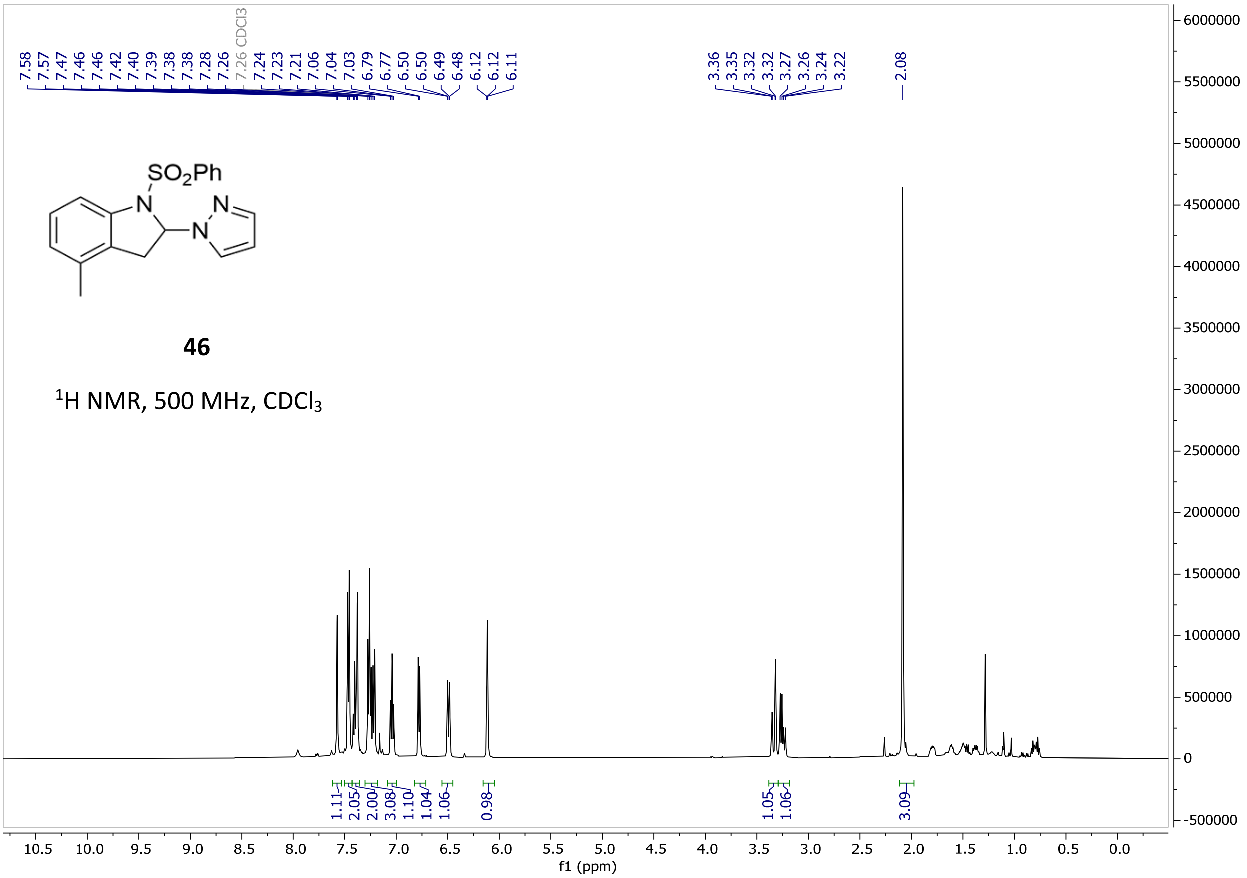
SO2Ph
N
N
N
46
1H NMR, 500 MHz, CDCl3
f1 (ppm)

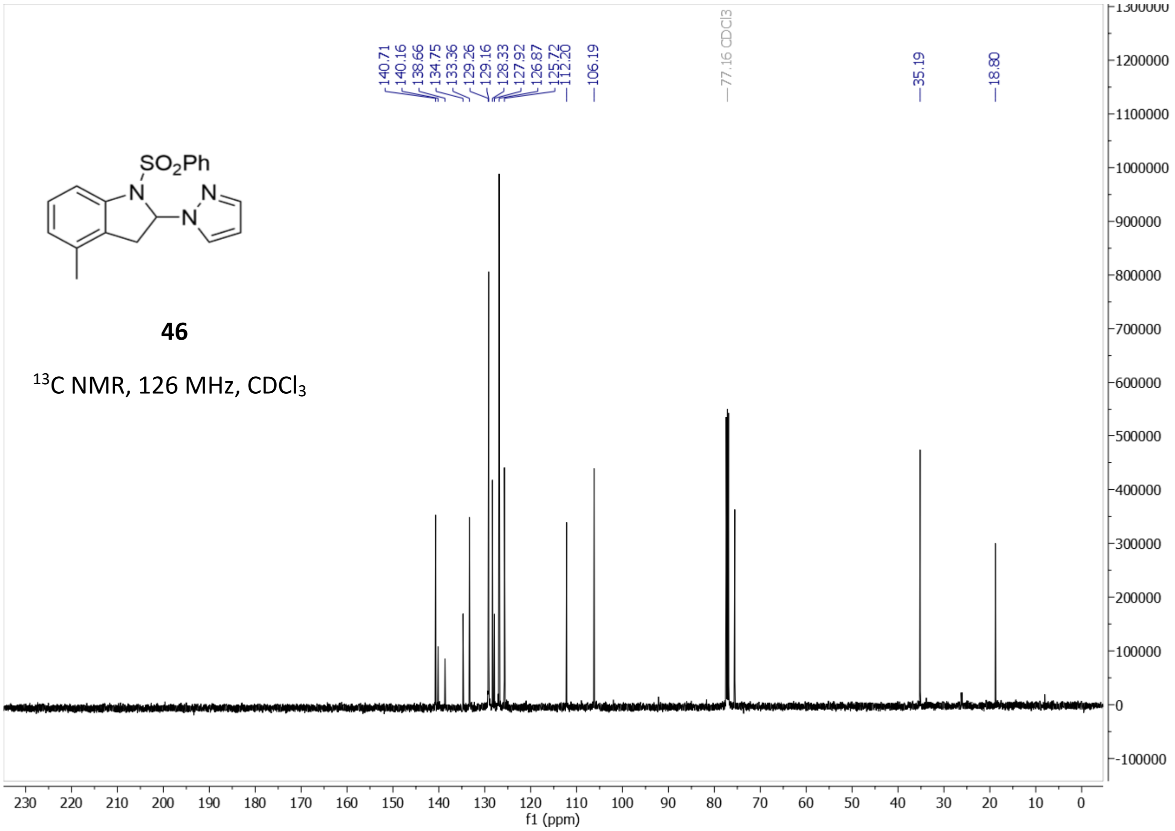
SO2Ph
N
N
N
46
13C NMR, 126 MHz, CDCl3
f1 (ppm)

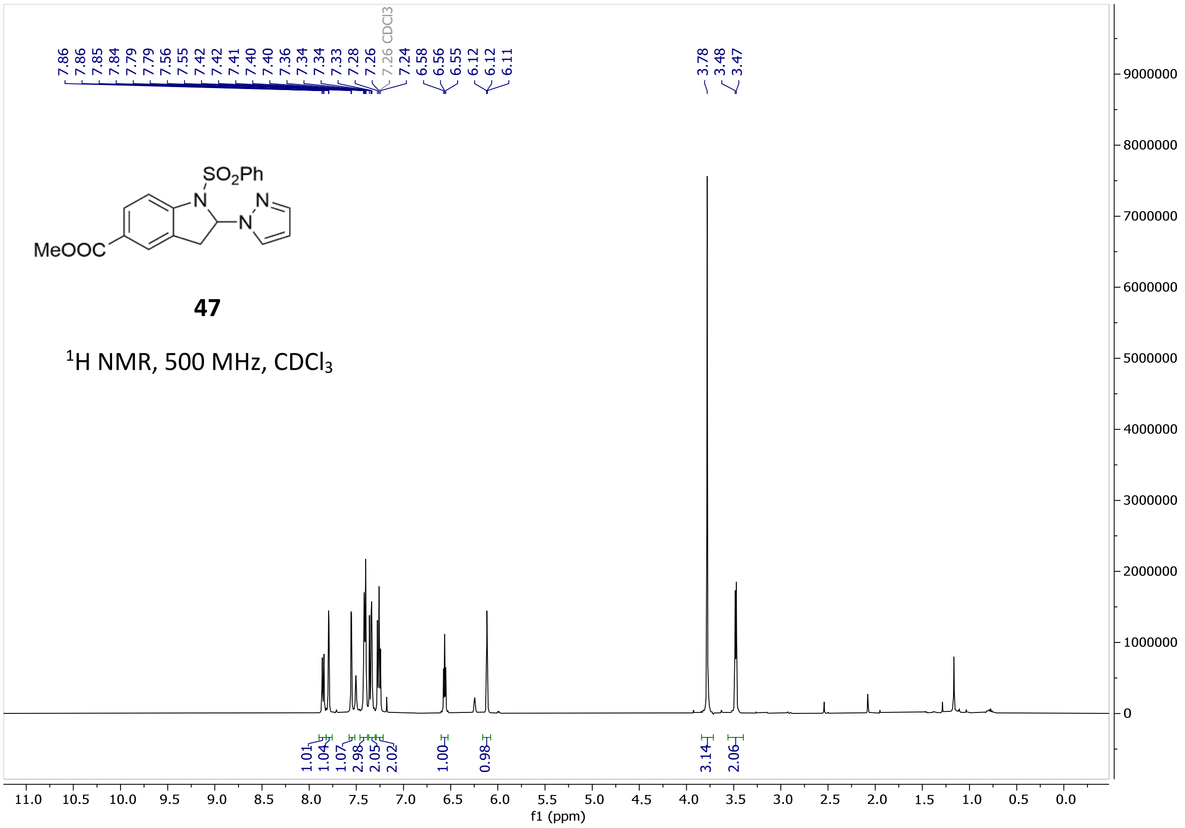
7.86
7.86
7.85
7.84
7.79
7.79
7.56
7.55
7.42
7.42
7.41
7.40
7.40
7.36
7.34
7.34
7.33
7.28
7.26
7.26 CDCl3
7.24
6.58
6.56
6.55
6.12
6.12
6.11
3.78
3.48
3.47
SO2Ph
N
N
N
MeOOC
47
1H NMR, 500 MHz, CDCl3
1.01
1.04
1.07
2.98
2.05
2.02
1.00
0.98
3.14
2.06
f1 (ppm)

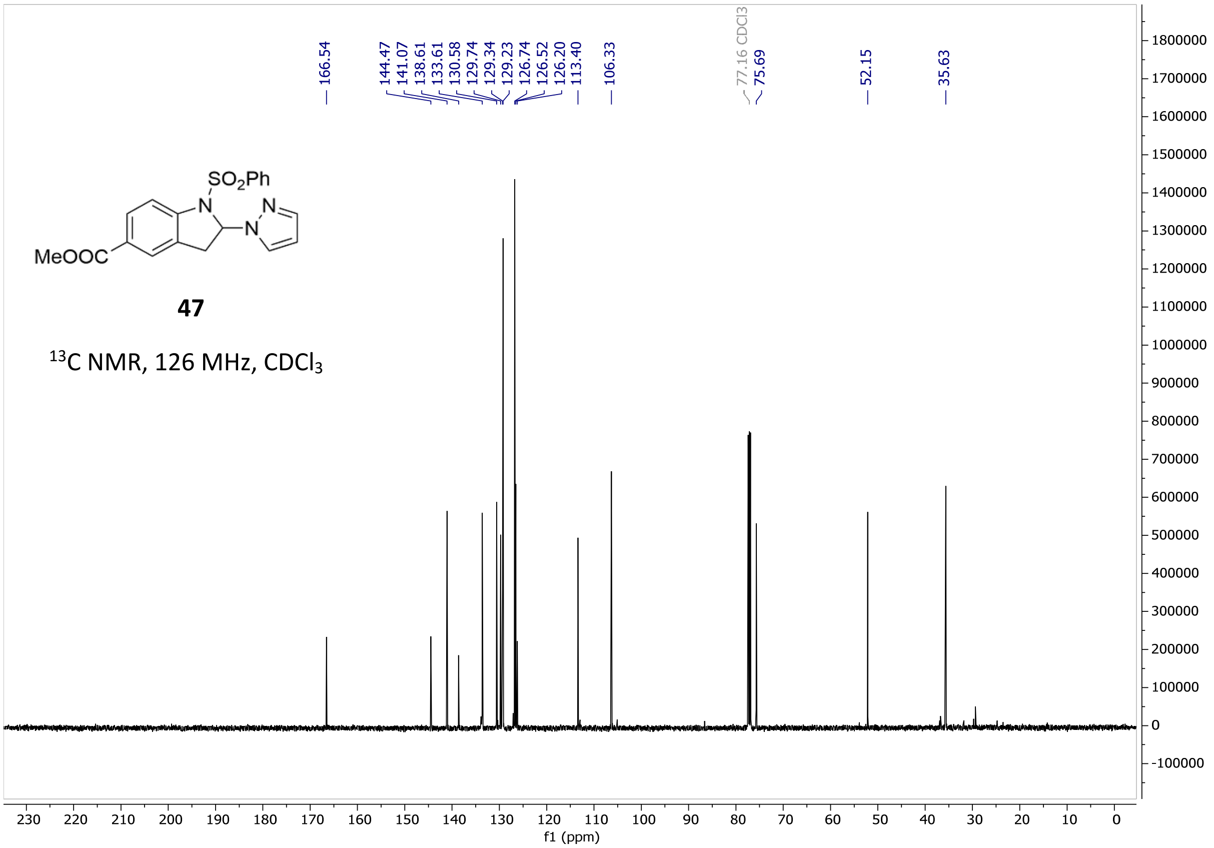
166.54
144.47
141.07
138.61
133.61
130.58
129.74
129.34
129.23
126.74
126.52
126.20
113.40
106.33
77.16 CDCl3
75.69
52.15
35.63
SO2Ph
N
N
N
MeOOC
47
13C NMR, 126 MHz, CDCl3
f1 (ppm)

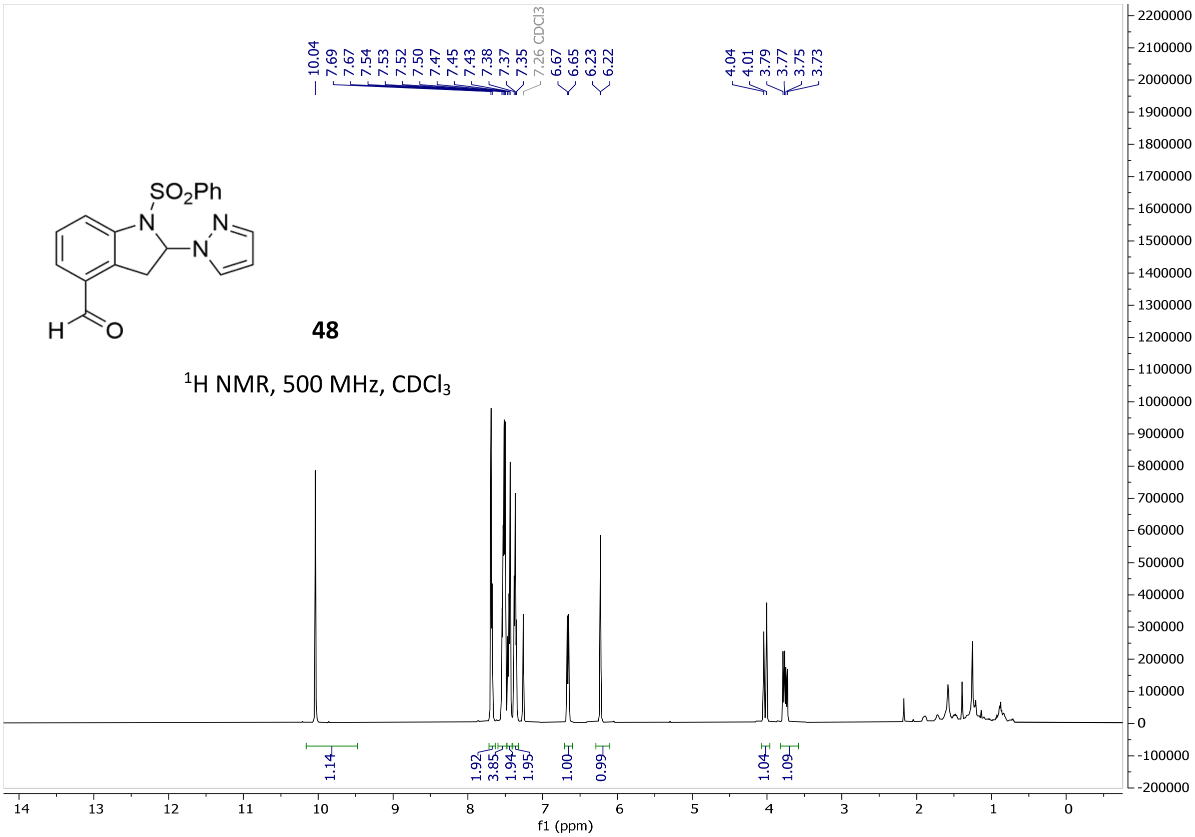

SO2Ph
N
N
N
H
O
48
1H NMR, 500 MHz, CDCl3
10.04
7.69
7.67
7.54
7.53
7.52
7.50
7.47
7.45
7.43
7.38
7.37
7.35
7.26 CDCl3
6.67
6.65
6.23
6.22
4.04
4.01
3.79
3.77
3.75
3.73
1.14
1.92
3.85
1.94
1.95
1.00
0.99
1.04
1.09
f1 (ppm)

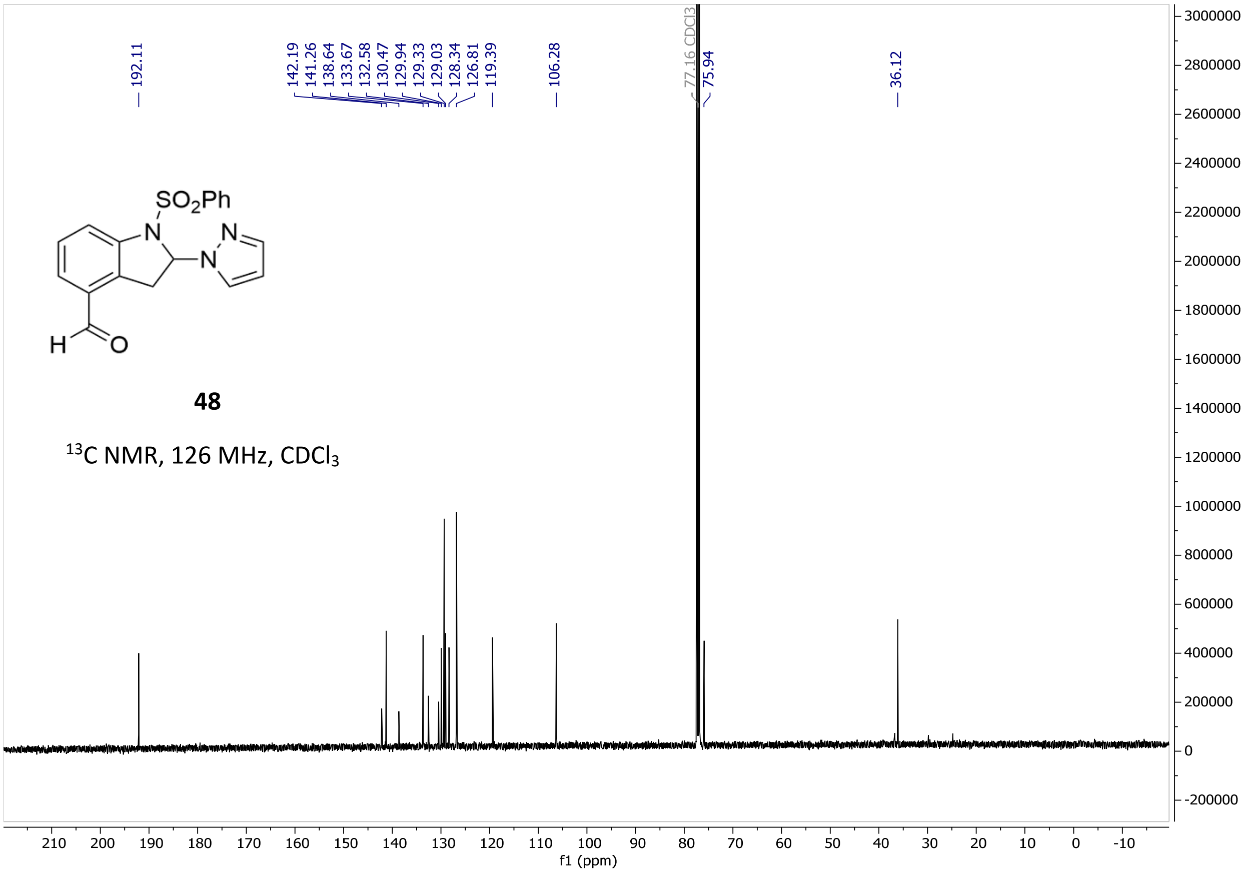

SO2Ph
N
N
N
H
O
48
13C NMR, 126 MHz, CDCl3
192.11
142.19
141.26
138.64
133.67
132.58
130.47
129.94
129.33
129.03
128.34
126.81
119.39
106.28
77.16 CDCl3
75.94
36.12
f1 (ppm)

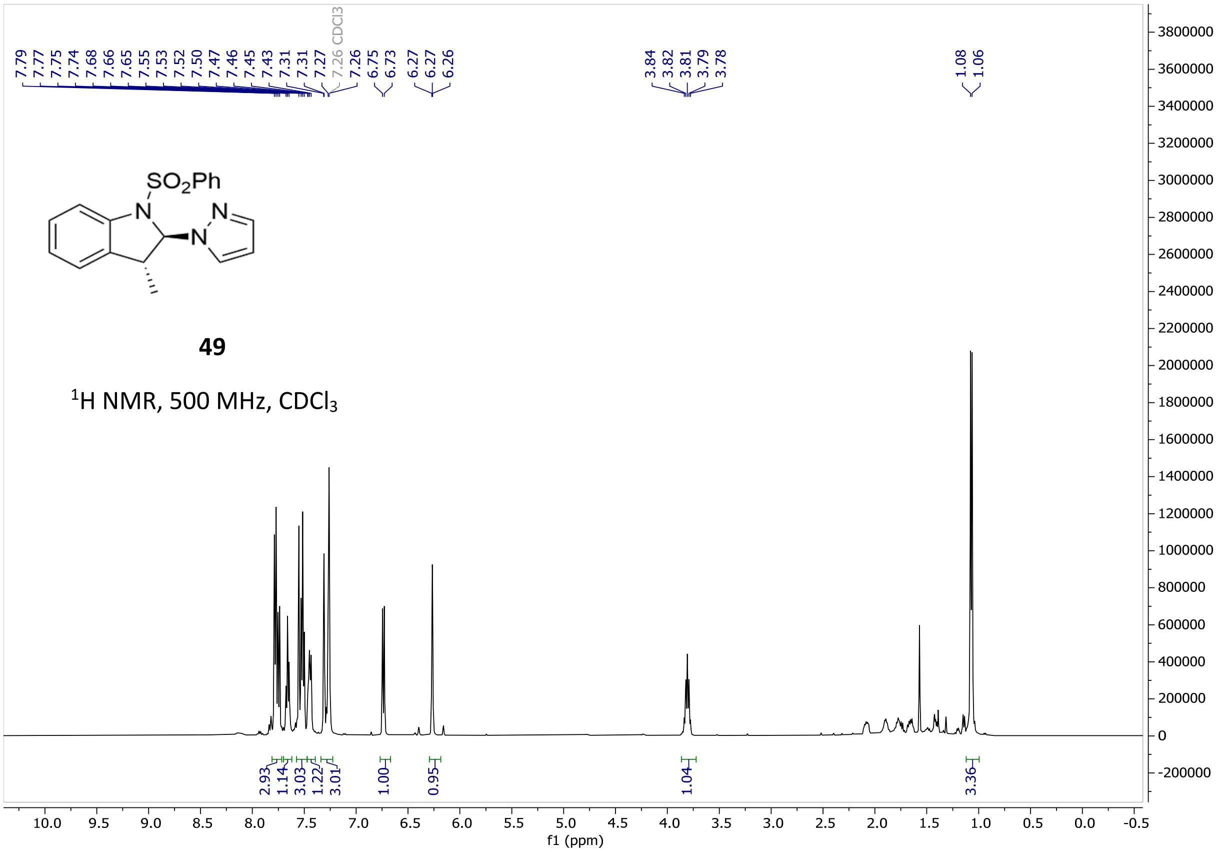

49

^{1}H NMR, 500 MHz, $CDCl_3$

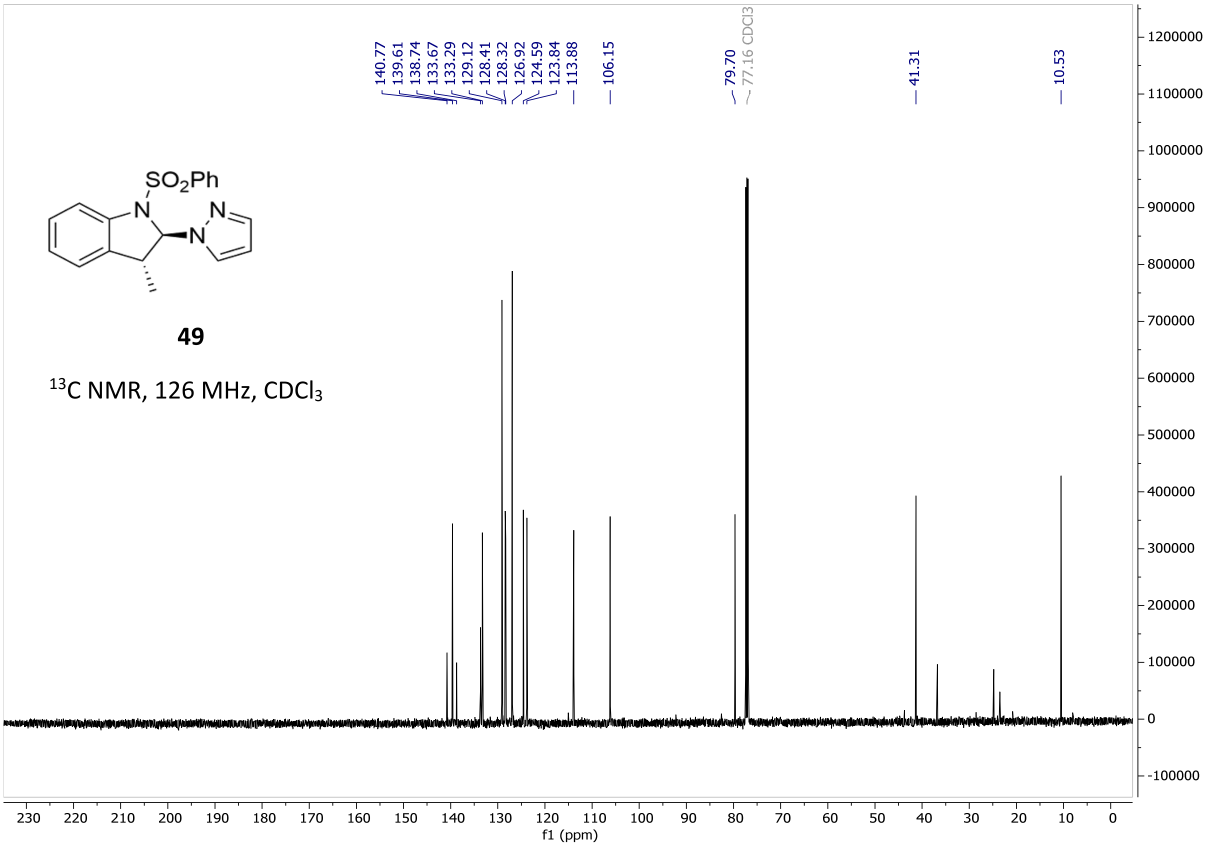

49

^{13}C NMR, 126 MHz, $CDCl_3$

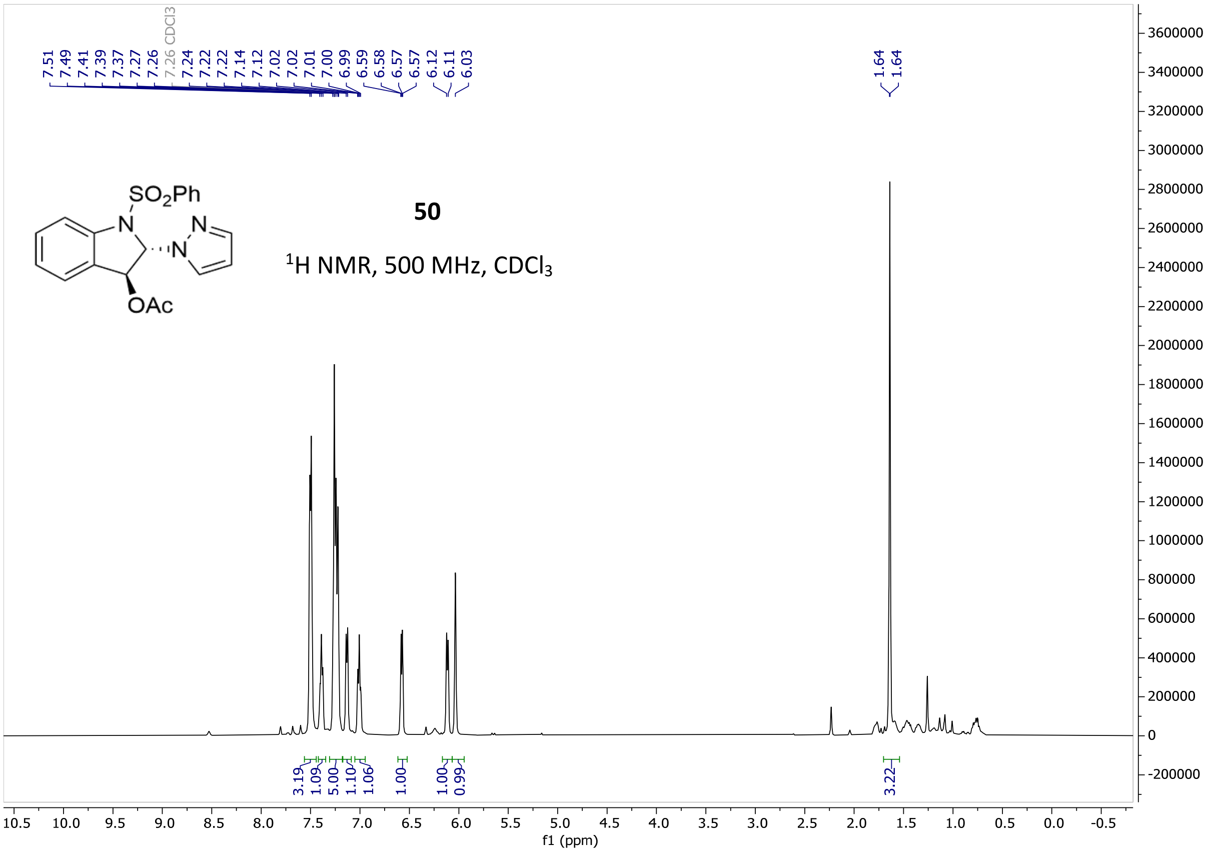
7.51
7.49
7.41
7.39
7.37
7.27
7.26
7.26 CDCl3
7.24
7.22
7.22
7.14
7.12
7.02
7.02
7.01
7.00
6.99
6.59
6.58
6.57
6.57
6.12
6.11
6.03
1.64
1.64
SO2Ph
N
N
OAc
50
1H NMR, 500 MHz, CDCl3
3.19
1.09
5.00
1.10
1.06
1.00
1.00
0.99
3.22
f1 (ppm)

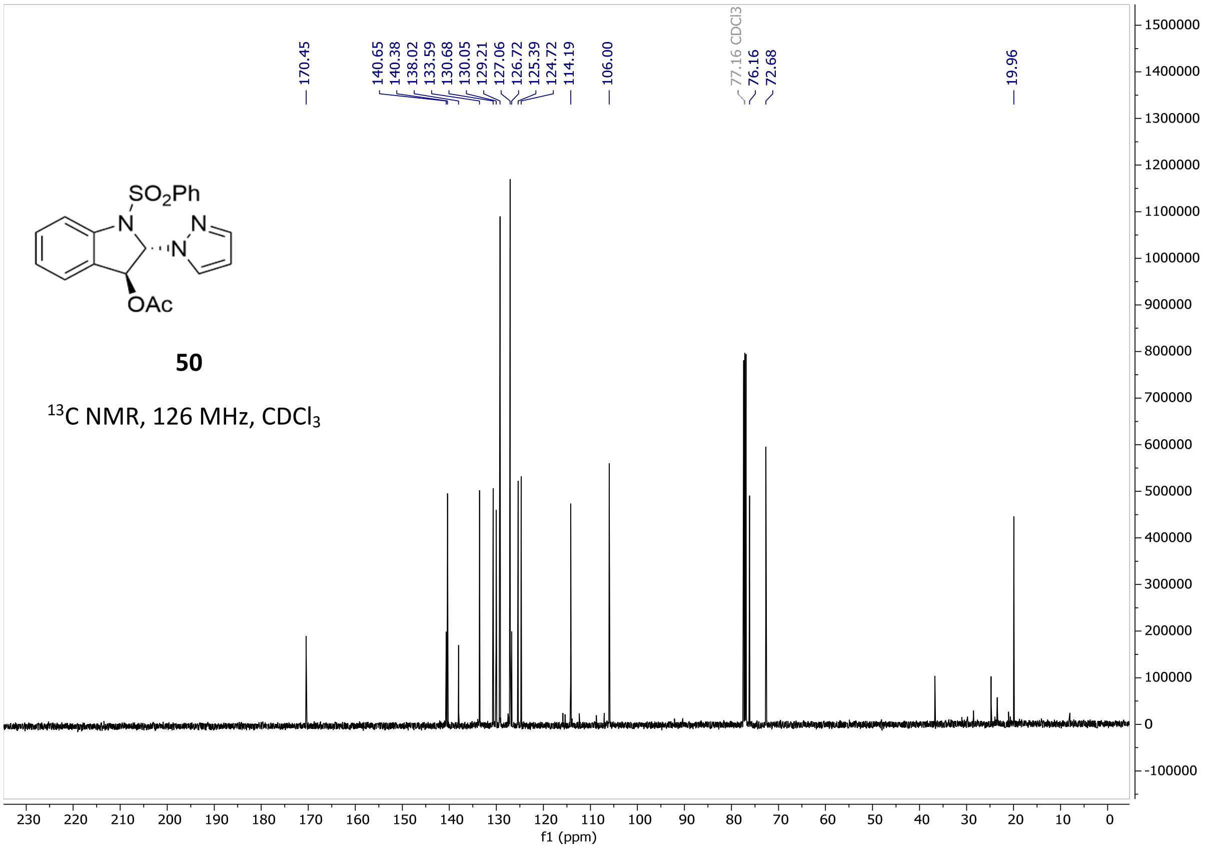
170.45
140.65
140.38
138.02
133.59
130.68
130.05
129.21
127.06
126.72
125.39
124.72
114.19
106.00
77.16 CDCl3
76.16
72.68
19.96
SO2Ph
N
N
OAc
50
13C NMR, 126 MHz, CDCl3
f1 (ppm)

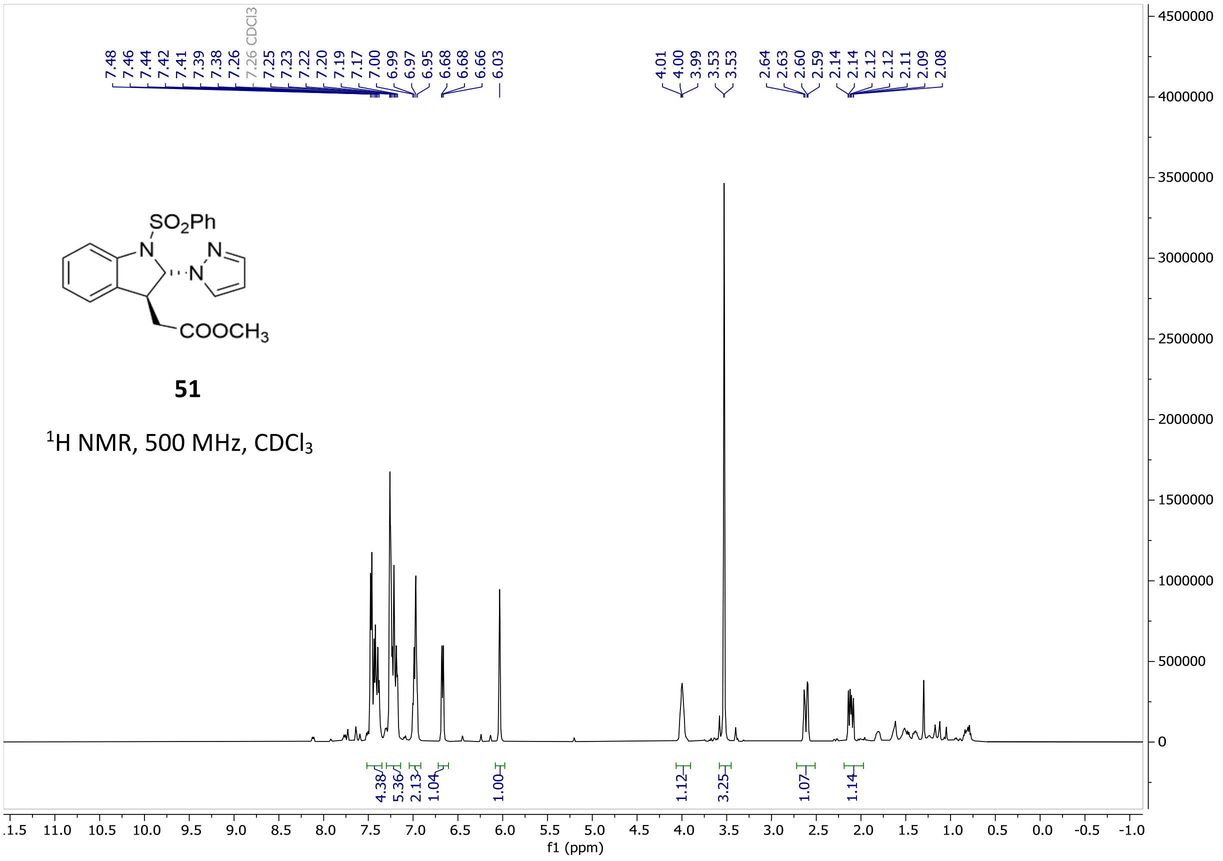
7.48 7.46 7.44 7.42 7.41 7.39 7.38 7.26 7.26 CDCl3 7.25 7.23 7.22 7.20 7.19 7.17 7.00 6.99 6.97 6.95 6.68 6.68 6.66 6.03
4.01 4.00 3.99 3.53 3.53 2.64 2.63 2.60 2.59 2.14 2.14 2.12 2.11 2.09 2.08
SO2Ph
N
N
N
COOCH3
51
1H NMR, 500 MHz, CDCl3
4.38 5.36 2.13 1.04 1.00 1.12 3.25 1.07 1.14
f1 (ppm)

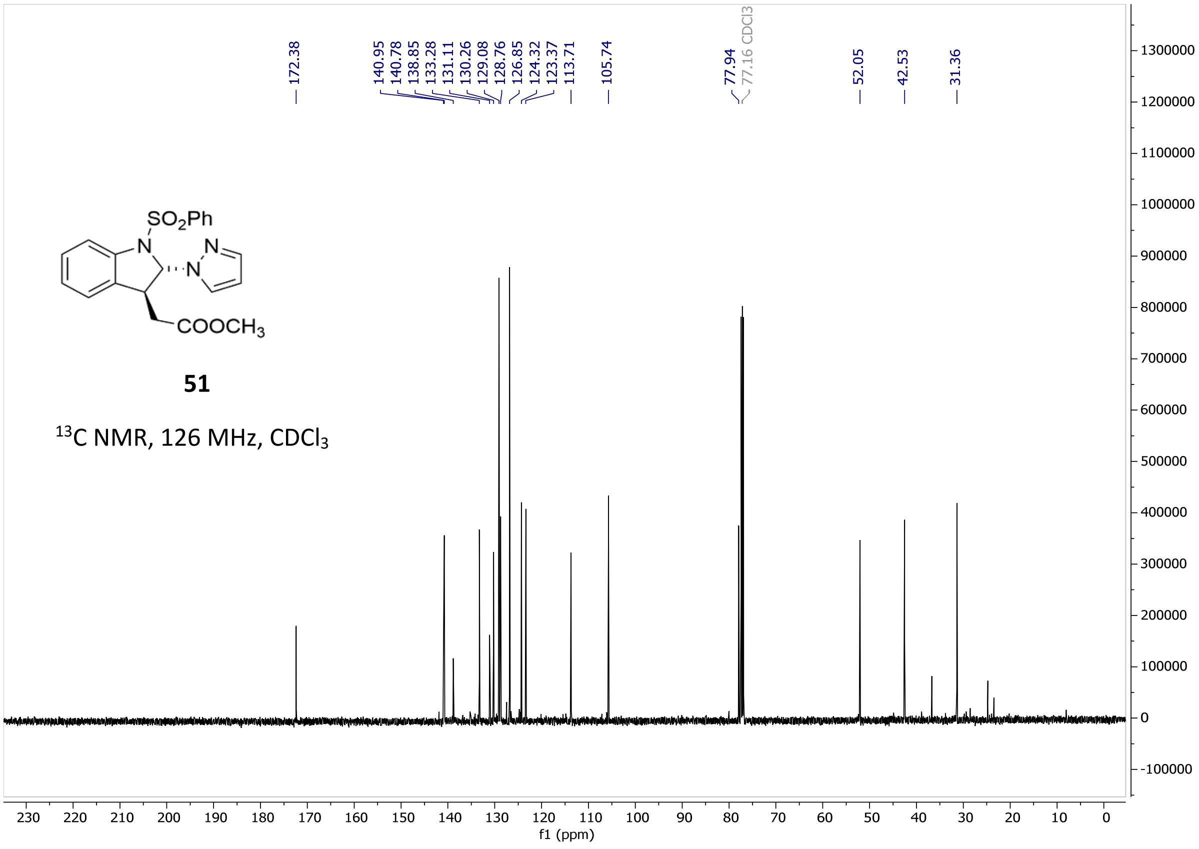
172.38
140.95 140.78 138.85 133.28 131.11 130.26 129.08 128.76 126.85 124.32 123.37 113.71 105.74
77.94 77.16 CDCl3
52.05 42.53 31.36
SO2Ph
N
N
N
COOCH3
51
13C NMR, 126 MHz, CDCl3
f1 (ppm)

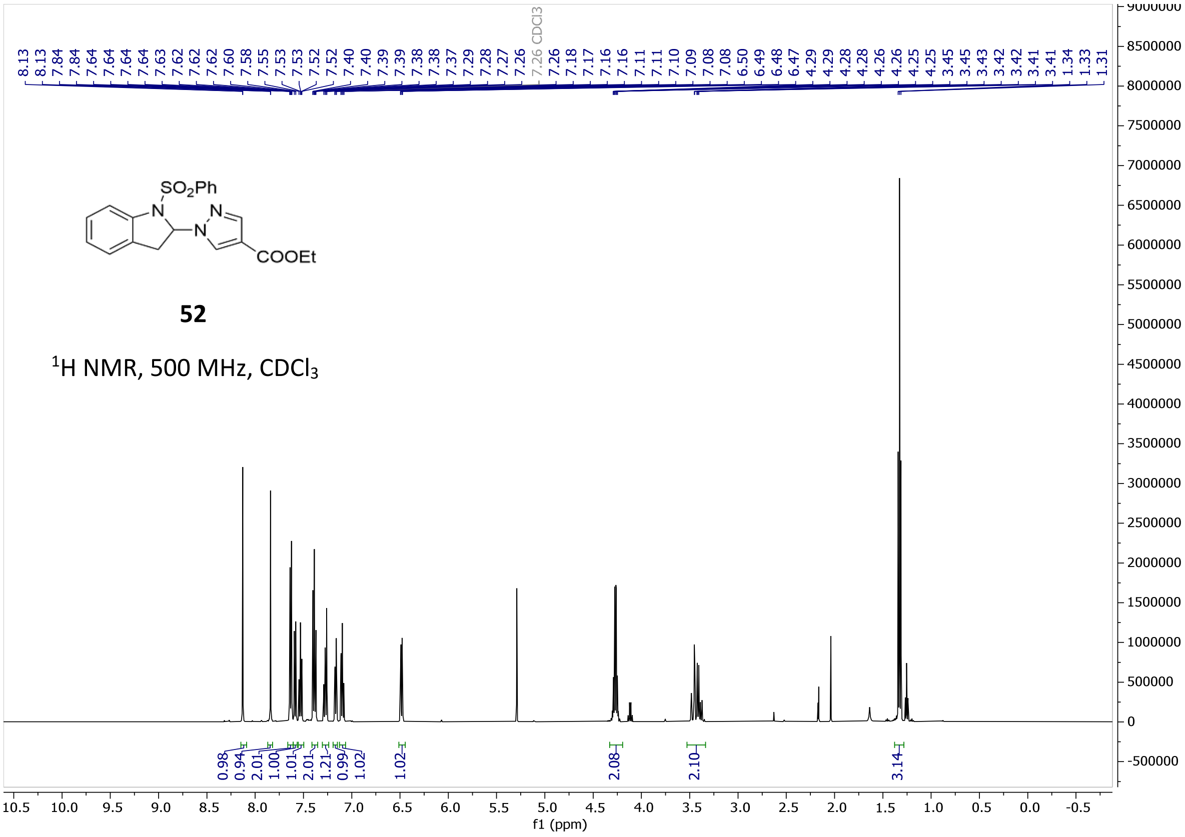
8.13 8.13 7.84 7.64 7.64 7.64 7.64 7.63 7.62 7.62 7.62 7.60 7.58 7.55 7.53 7.53 7.52 7.52 7.52 7.40 7.40 7.39 7.39 7.38 7.38 7.37 7.29 7.28 7.27 7.26 7.26 CDCl3 7.26 7.18 7.17 7.16 7.16 7.11 7.11 7.10 7.09 7.08 7.08 6.50 6.49 6.48 6.47 4.29 4.29 4.28 4.28 4.26 4.26 4.25 4.25 3.45 3.45 3.43 3.42 3.42 3.41 3.41 1.34 1.33 1.31
SO2Ph
N
N
N
COOEt
52
1H NMR, 500 MHz, CDCl3
0.98 0.94 2.01 1.00 1.01 2.01 1.21 0.99 1.02 1.02 2.08 2.10 3.14
f1 (ppm)

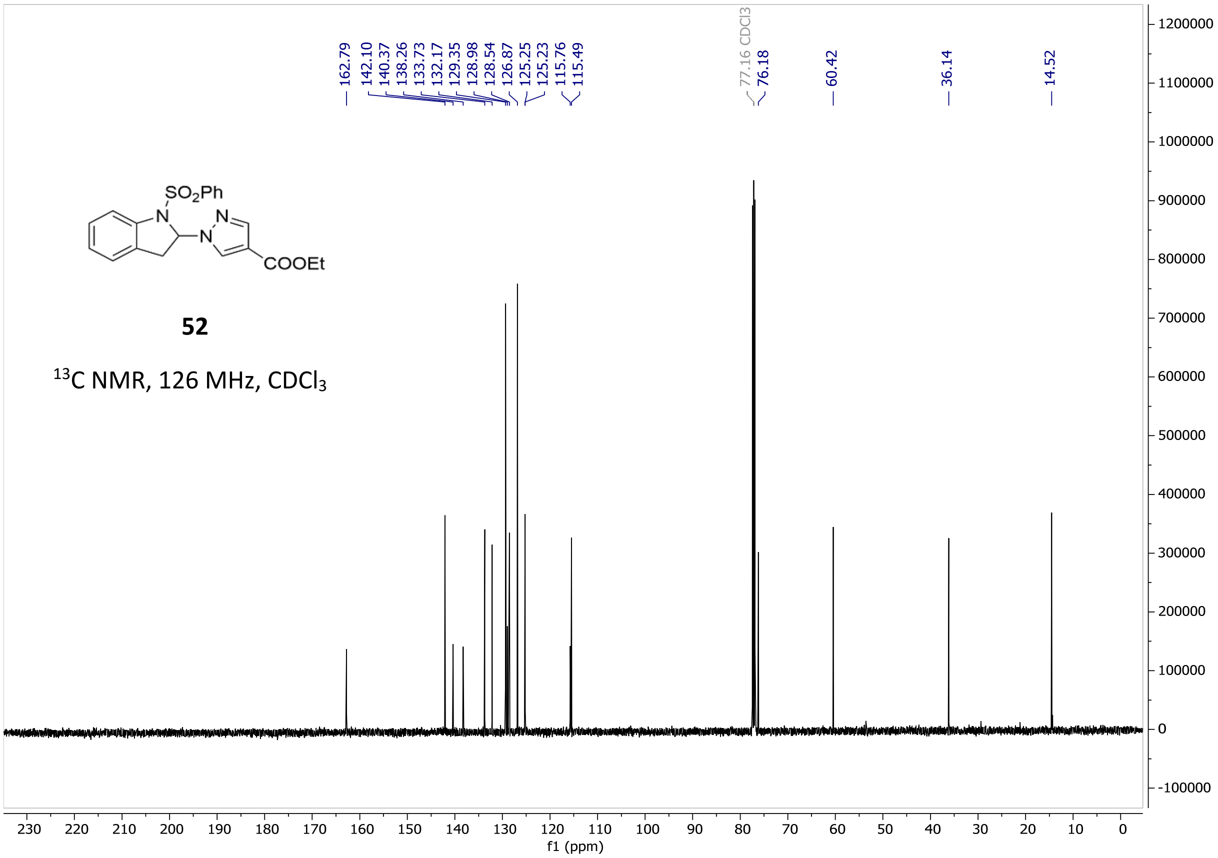
162.79 142.10 140.37 138.26 133.73 132.17 129.35 128.98 128.54 126.87 125.25 125.23 115.76 115.49 77.16 CDCl3 76.18 60.42 36.14 14.52
SO2Ph
N
N
N
COOEt
52
13C NMR, 126 MHz, CDCl3
f1 (ppm)

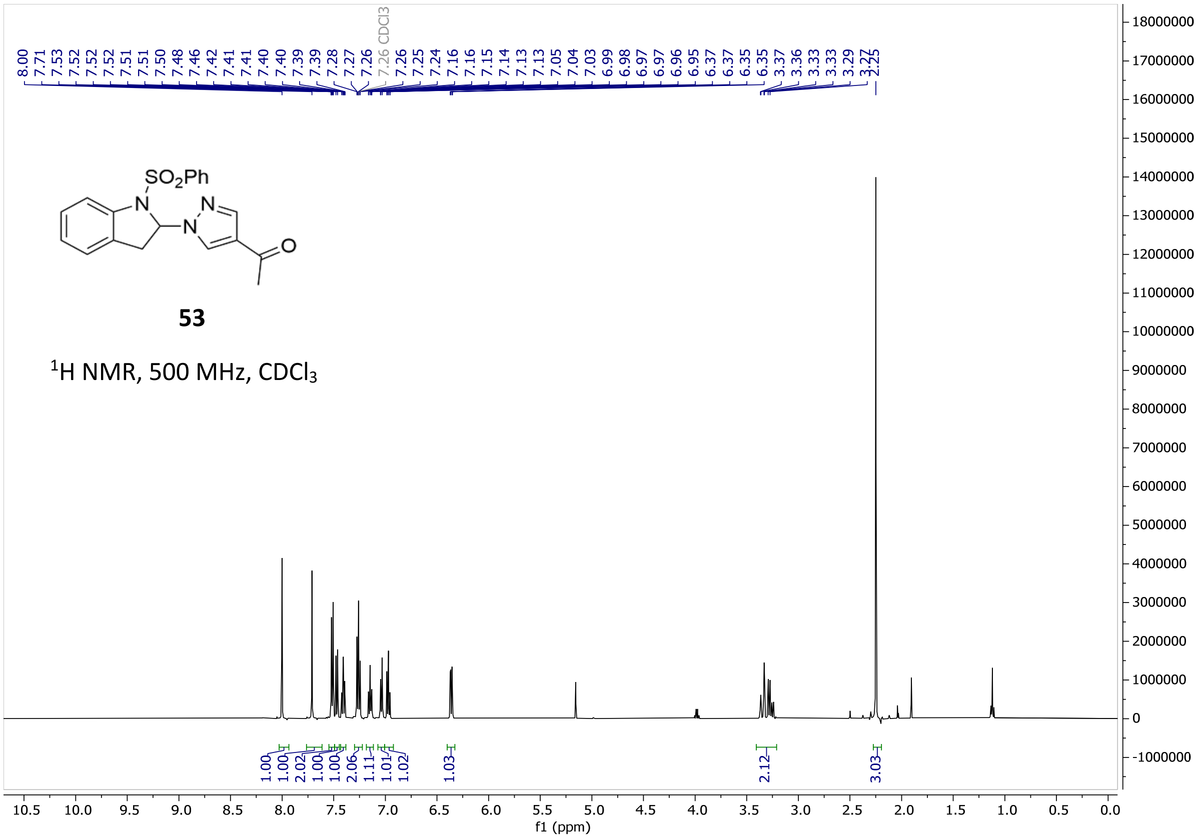
8.00 7.71 7.53 7.52 7.52 7.52 7.51 7.51 7.50 7.48 7.46 7.42 7.41 7.41 7.40 7.40 7.39 7.39 7.28 7.27 7.26 7.26 CDCl3 7.26 7.25 7.24 7.16 7.16 7.15 7.14 7.13 7.13 7.05 7.04 7.03 6.99 6.98 6.97 6.97 6.96 6.95 6.37 6.37 6.35 6.35 3.37 3.36 3.33 3.33 3.29 3.27 2.25
SO2Ph
N
N
53
1H NMR, 500 MHz, CDCl3
1.00 1.00 2.02 1.00 1.00 2.06 1.11 1.01 1.02 1.03 2.12 3.03
f1 (ppm)

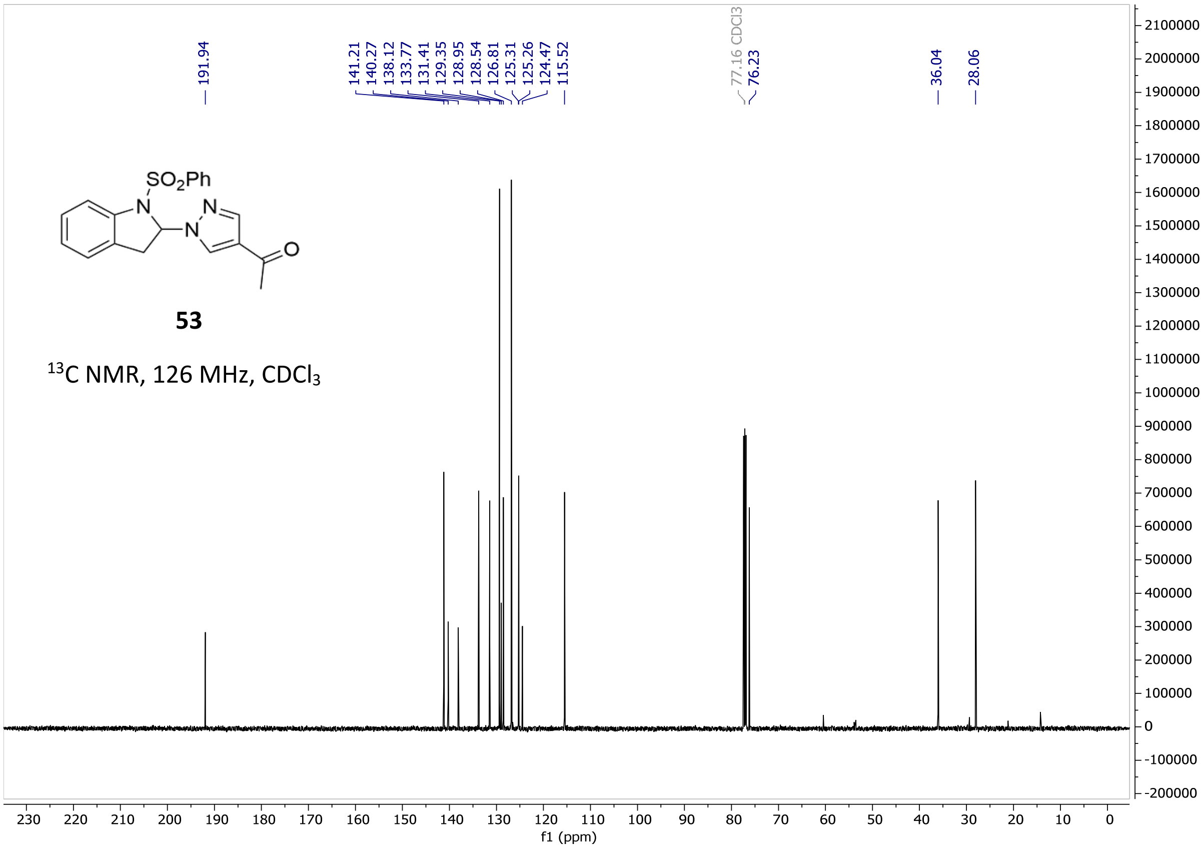
191.94
141.21 140.27 138.12 133.77 131.41 129.35 128.95 128.54 126.81 125.31 125.26 124.47 115.52
77.16 CDCl3 76.23
36.04 28.06
SO2Ph
N
N
53
13C NMR, 126 MHz, CDCl3
f1 (ppm)

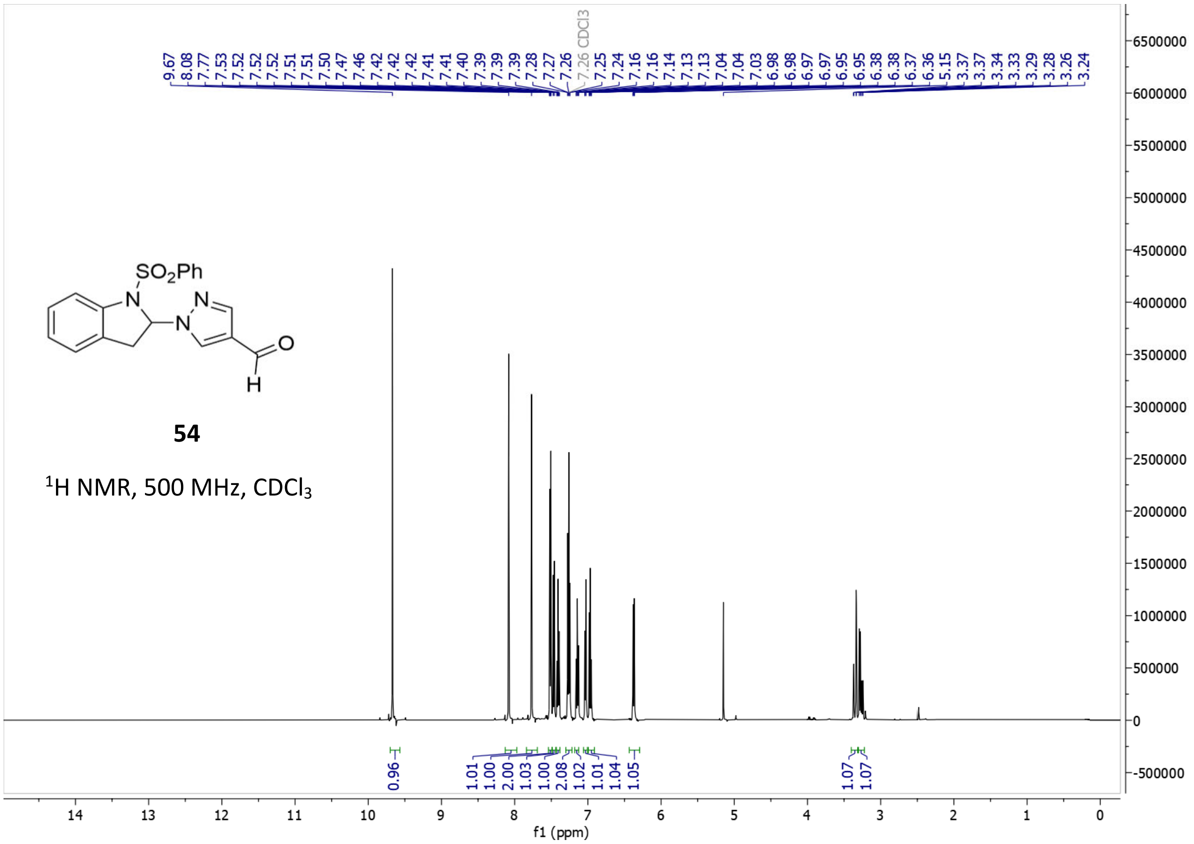
9.67 8.08 7.77 7.53 7.52 7.52 7.52 7.51 7.51 7.50 7.47 7.46 7.42 7.42 7.41 7.41 7.40 7.39 7.39 7.39 7.38 7.28 7.27 7.26 7.26 CDCl3 7.25 7.24 7.16 7.16 7.14 7.13 7.13 7.04 7.04 7.03 6.98 6.98 6.97 6.97 6.95 6.95 6.38 6.38 6.37 6.36 5.15 3.37 3.37 3.34 3.33 3.29 3.28 3.26 3.24
SO2Ph
N
N
N
O
H
54
1H NMR, 500 MHz, CDCl3
0.96 1.01 1.00 2.00 1.03 1.00 2.08 1.02 1.01 1.04 1.05 1.07 1.07
f1 (ppm)

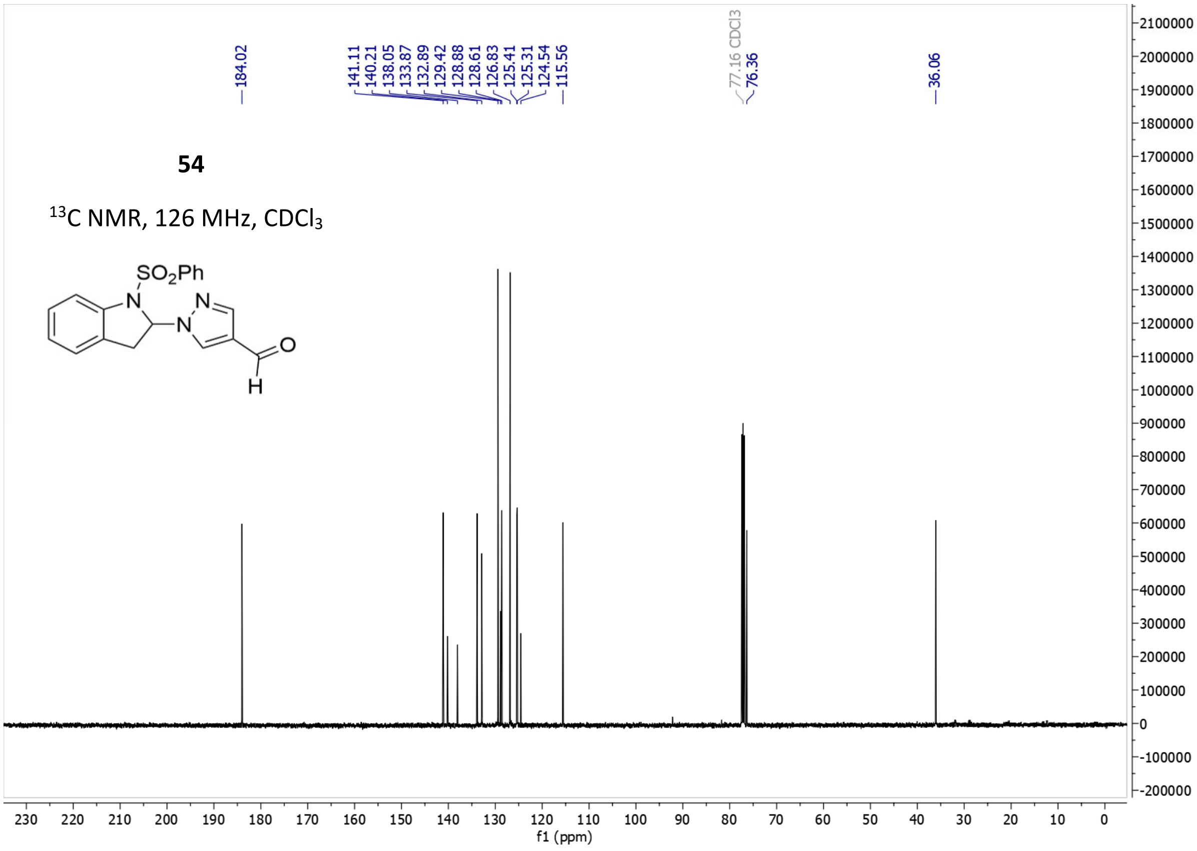
184.02
141.11 140.21 138.05 133.87 132.89 129.42 128.88 128.61 126.83 125.41 125.31 124.54 115.56
77.16 CDCl3 76.36
36.06
54
13C NMR, 126 MHz, CDCl3
SO2Ph
N
N
N
O
H
f1 (ppm)

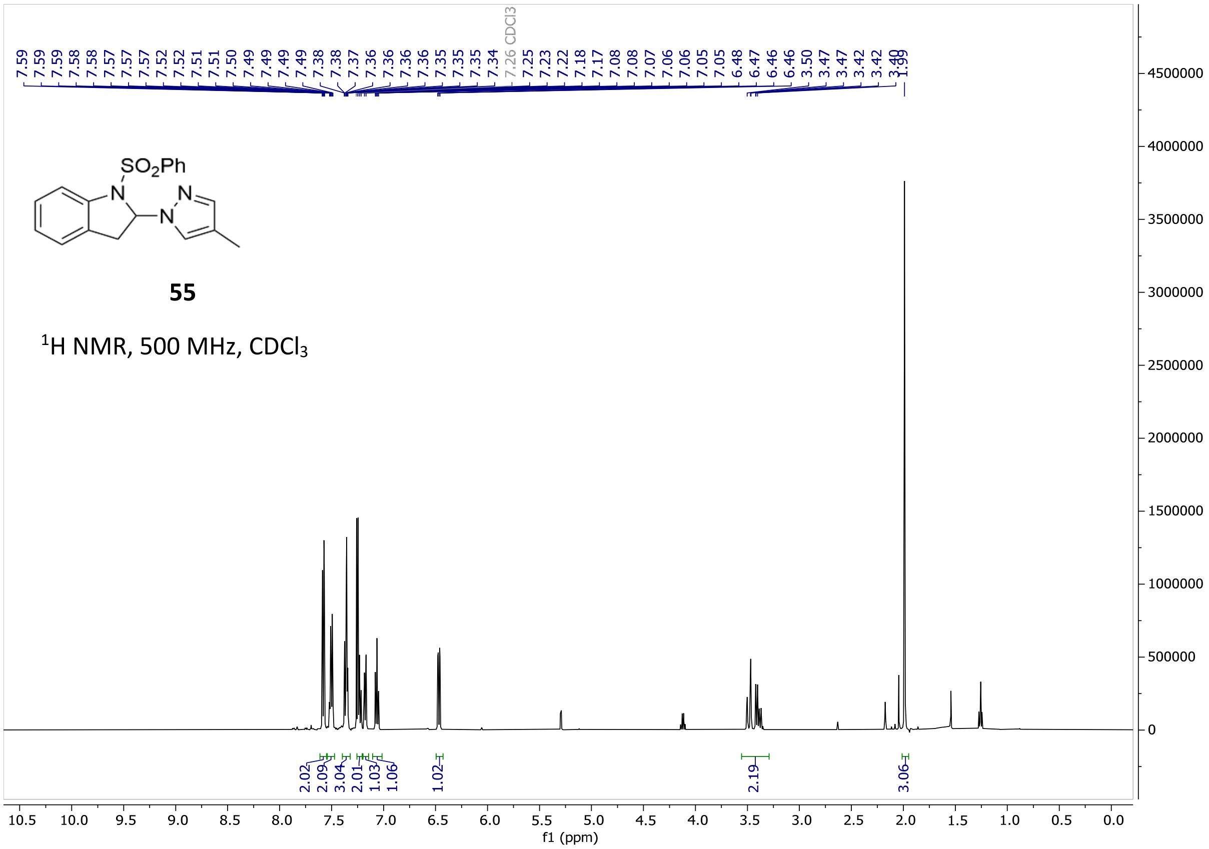
SO2Ph
N
N
N
55
1H NMR, 500 MHz, CDCl3
f1 (ppm)

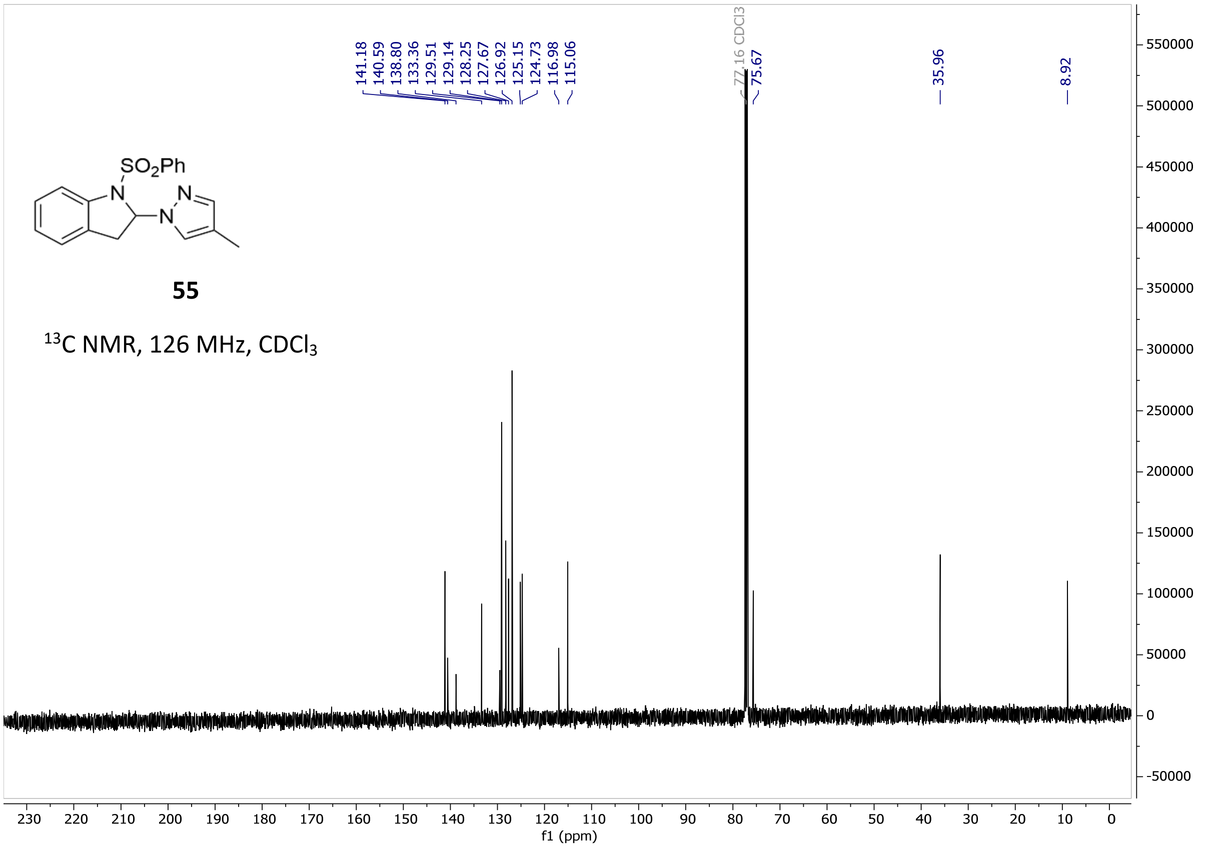
SO2Ph
N
N
N
55
13C NMR, 126 MHz, CDCl3
141.18
140.59
138.80
133.36
129.51
129.14
128.25
127.67
126.92
125.15
124.73
116.98
115.06
77.16 CDCl3
75.67
35.96
8.92
f1 (ppm)

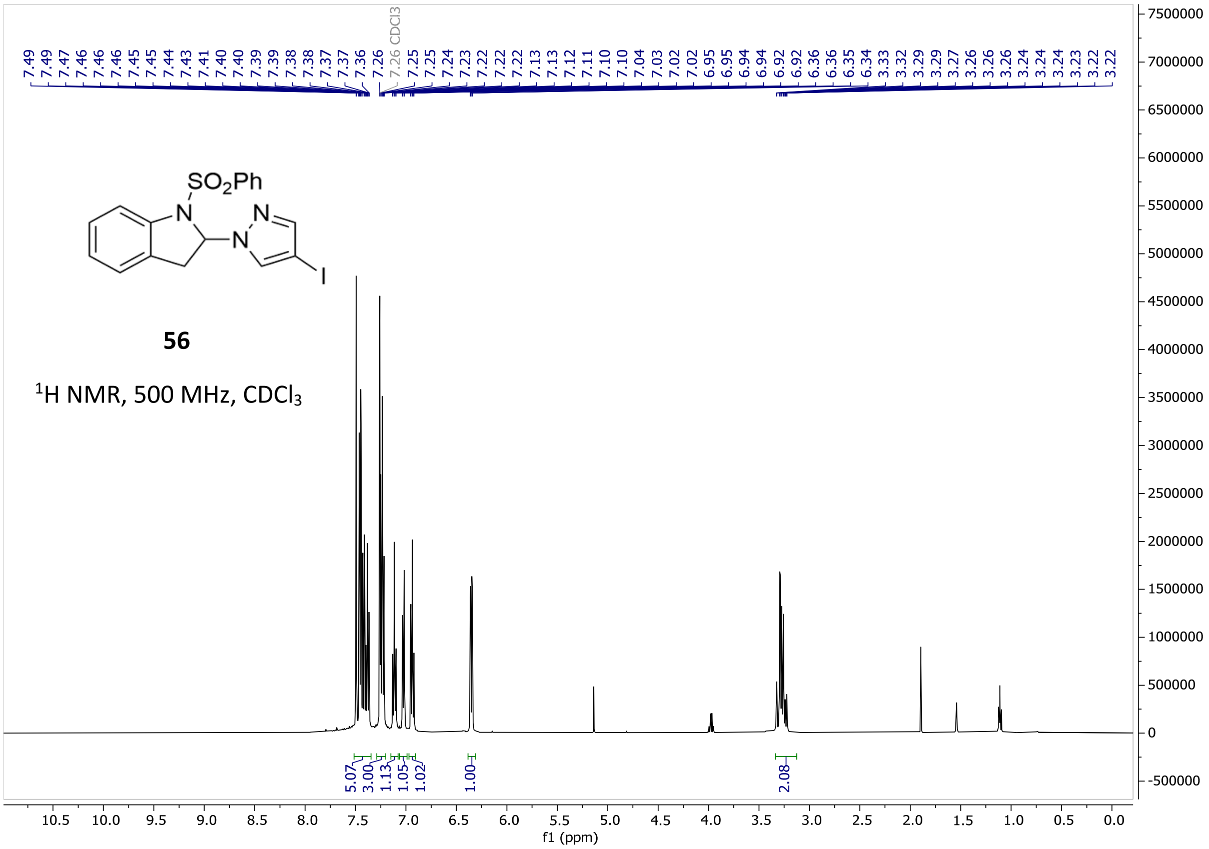
SO2Ph
N
N
N
I
56
1H NMR, 500 MHz, CDCl3
7.49 7.49 7.47 7.46 7.46 7.45 7.45 7.44 7.43 7.41 7.40 7.40 7.39 7.39 7.38 7.38 7.37 7.37 7.36 7.26 CDCl3 7.25 7.25 7.24 7.23 7.22 7.22 7.22 7.13 7.13 7.12 7.11 7.10 7.10 7.04 7.03 7.02 7.02 6.95 6.94 6.94 6.92 6.92 6.36 6.36 6.35 6.34 3.33 3.32 3.29 3.27 3.26 3.26 3.24 3.24 3.23 3.22 3.22
5.07 3.00 1.13 1.05 1.02 1.00 2.08
f1 (ppm)

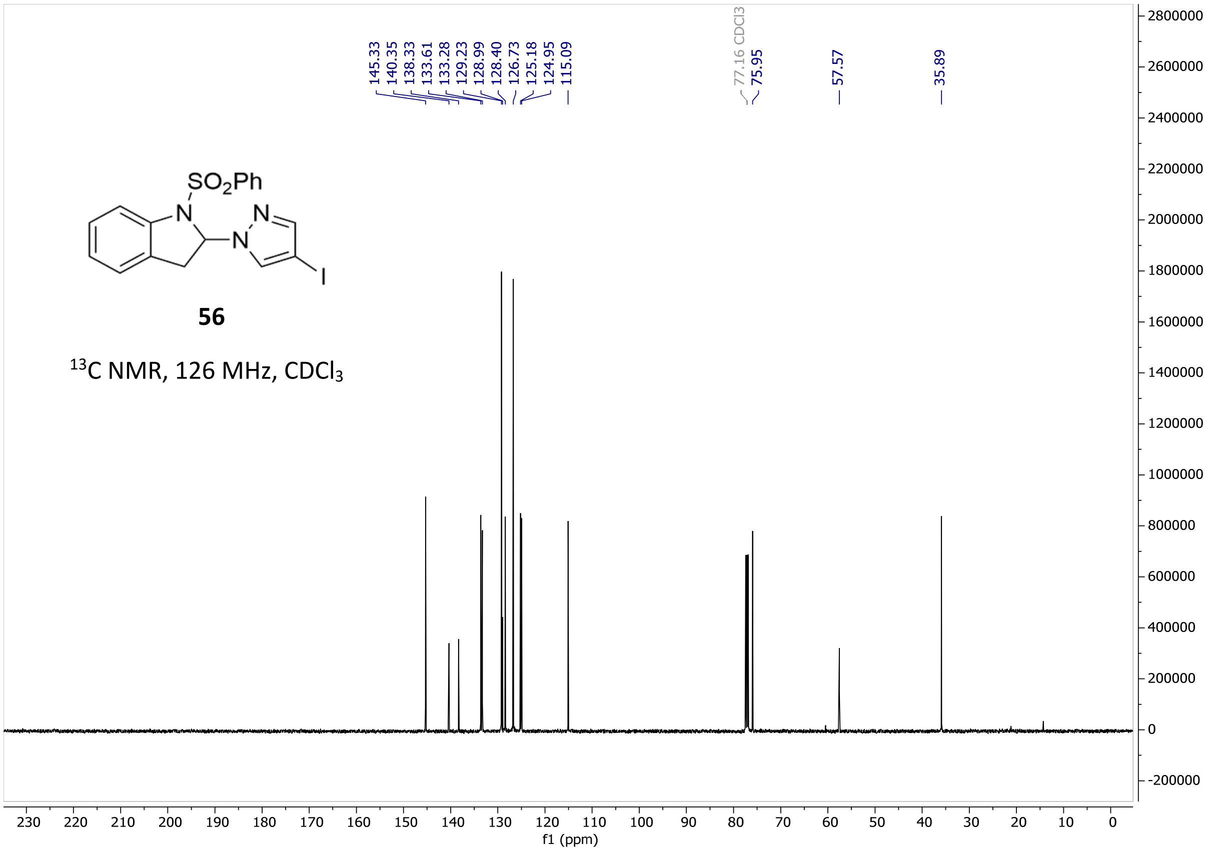
145.33 140.35 138.33 133.61 133.28 129.23 128.99 128.40 126.73 125.18 124.95 115.09
77.16 CDCl3 75.95
57.57
35.89
SO2Ph
N
N
N
I
56
13C NMR, 126 MHz, CDCl3
f1 (ppm)

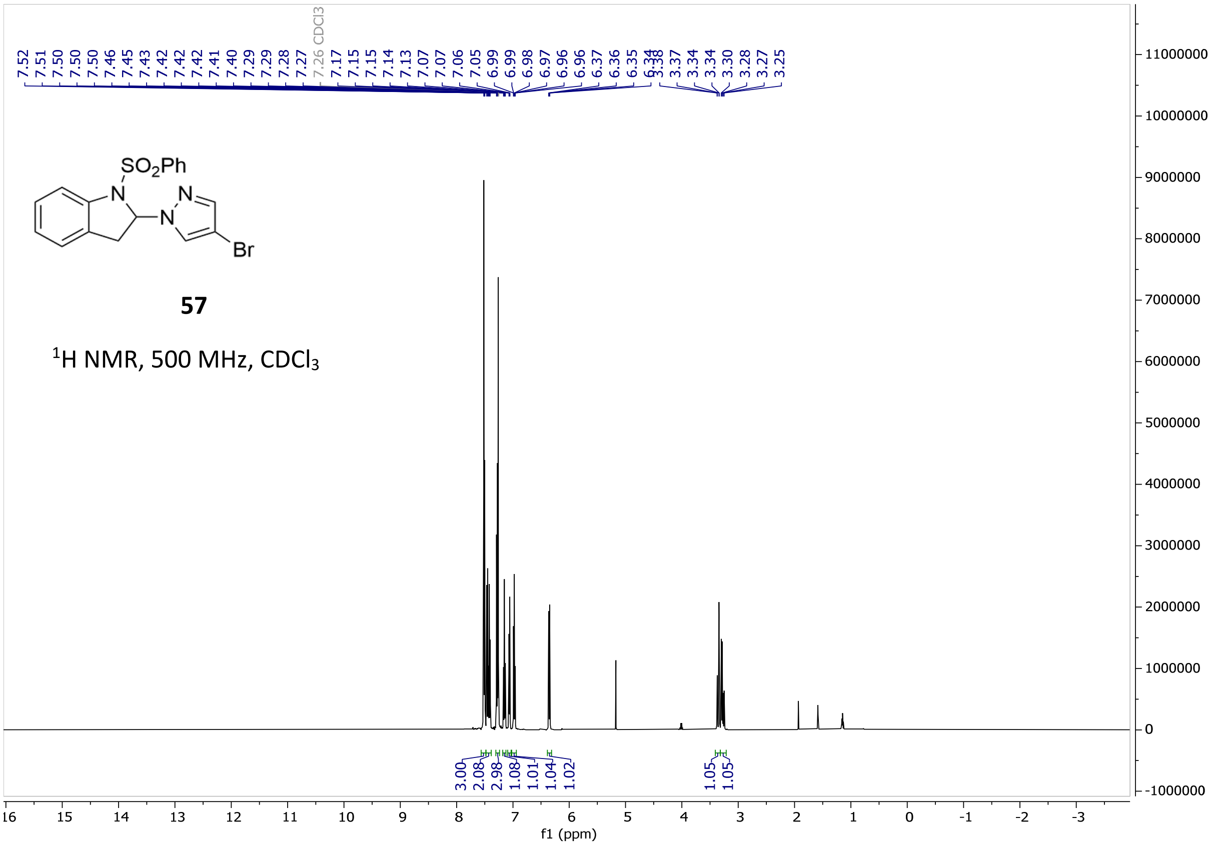
7.52 7.51 7.50 7.50 7.50 7.46 7.45 7.43 7.42 7.42 7.42 7.41 7.40 7.29 7.29 7.28 7.27 7.26 CDCl3 7.17 7.15 7.15 7.14 7.13 7.07 7.07 7.06 7.05 6.99 6.99 6.98 6.97 6.96 6.96 6.37 6.36 6.35 6.34 3.38 3.37 3.34 3.34 3.30 3.28 3.27 3.25
SO2Ph
N
N
N
Br
57
1H NMR, 500 MHz, CDCl3
3.00 2.08 2.98 1.08 1.01 1.04 1.02 1.05 1.05
f1 (ppm)

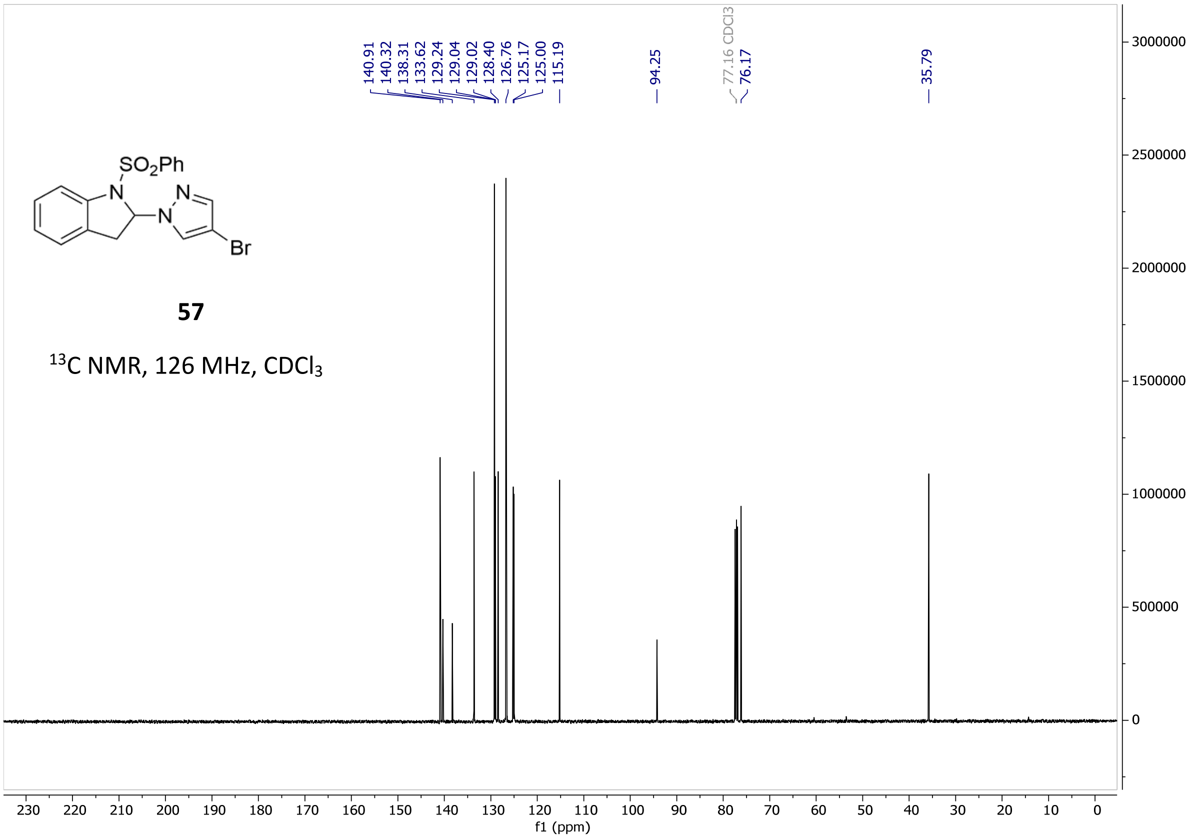
140.91 140.32 138.31 133.62 129.24 129.04 129.02 128.40 126.76 125.17 125.00 115.19 94.25 77.16 CDCl3 76.17 35.79
SO2Ph
N
N
N
Br
57
13C NMR, 126 MHz, CDCl3
f1 (ppm)

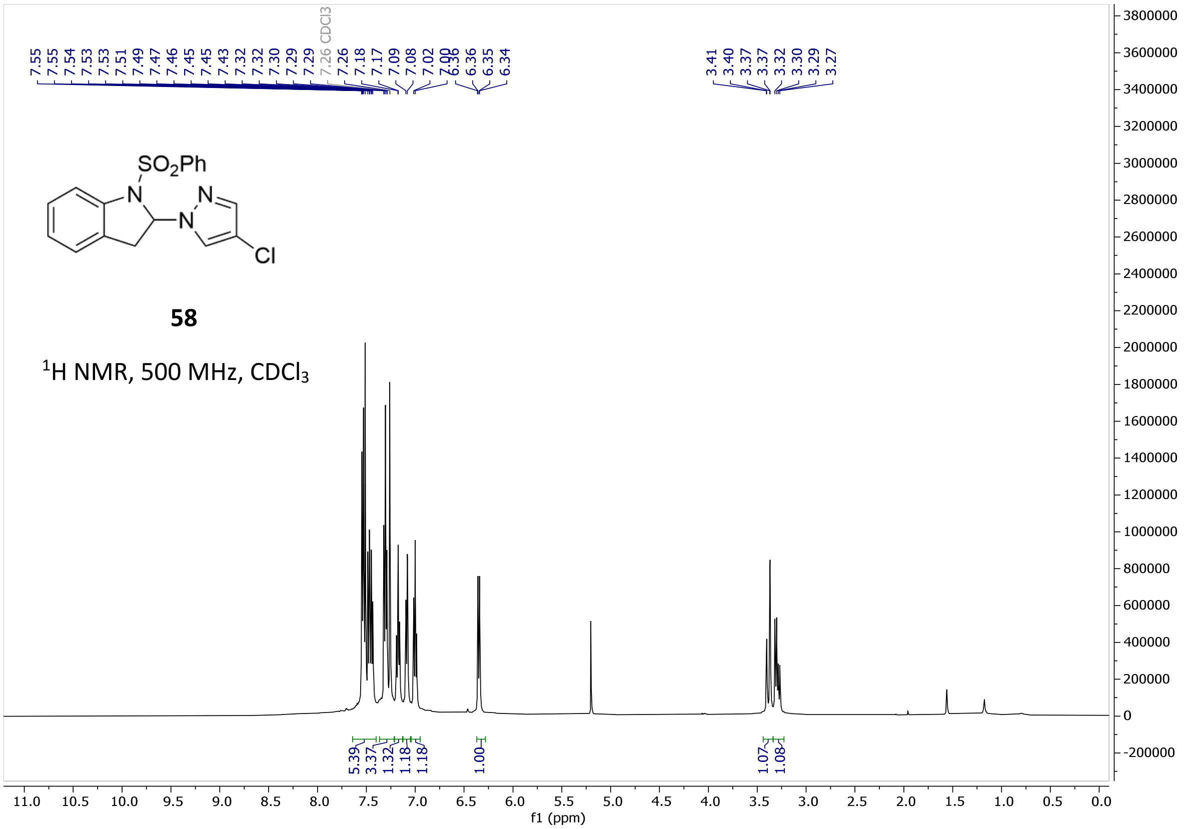
7.55
7.55
7.54
7.53
7.53
7.51
7.49
7.47
7.46
7.45
7.45
7.43
7.32
7.32
7.30
7.29
7.29
7.26 CDCl3
7.26
7.18
7.17
7.09
7.08
7.02
7.00
6.36
6.36
6.35
6.34
3.41
3.40
3.37
3.37
3.32
3.30
3.29
3.27
SO2Ph
N
N
N
Cl
58
1H NMR, 500 MHz, CDCl3
5.39
3.37
1.32
1.18
1.18
1.00
1.07
1.08
f1 (ppm)

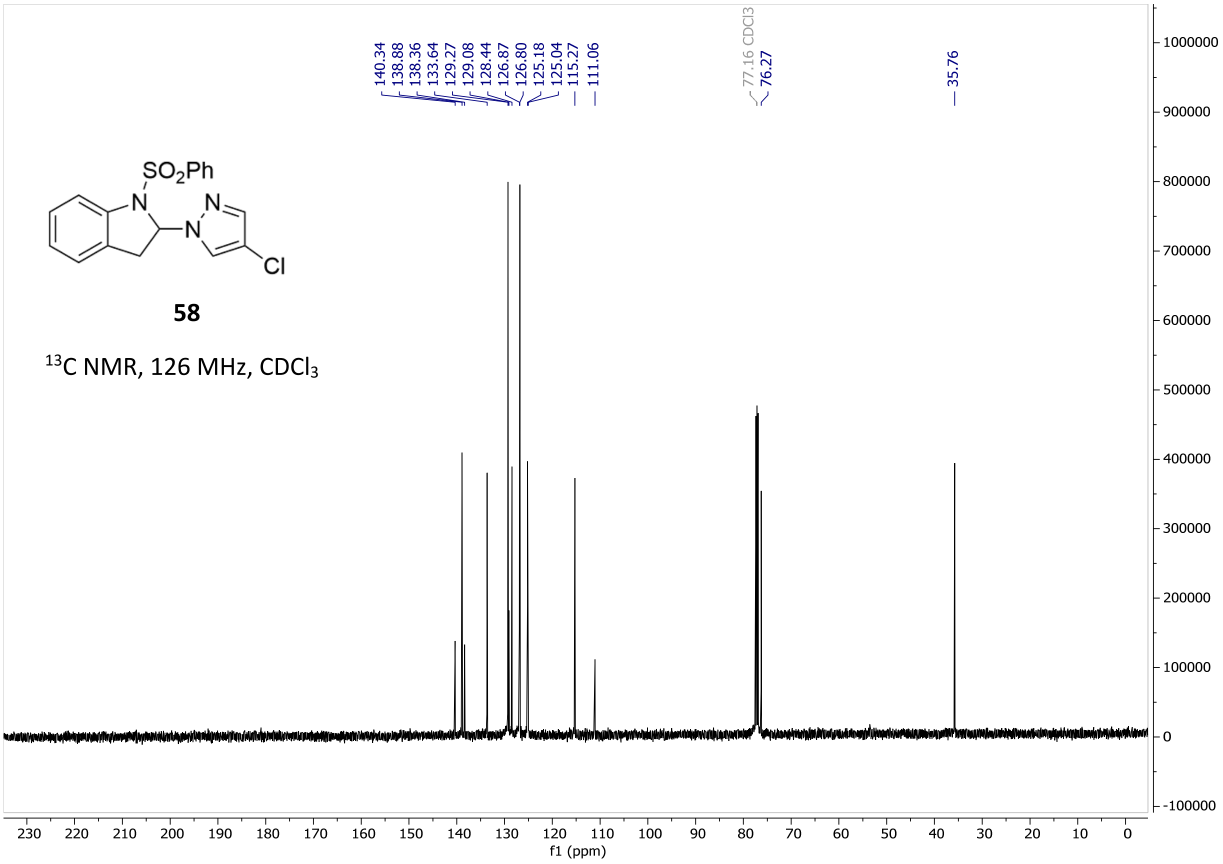
140.34
138.88
138.36
133.64
129.27
129.08
128.44
126.87
126.80
125.18
125.04
115.27
111.06
77.16 CDCl3
76.27
35.76
SO2Ph
N
N
N
Cl
58
13C NMR, 126 MHz, CDCl3
f1 (ppm)

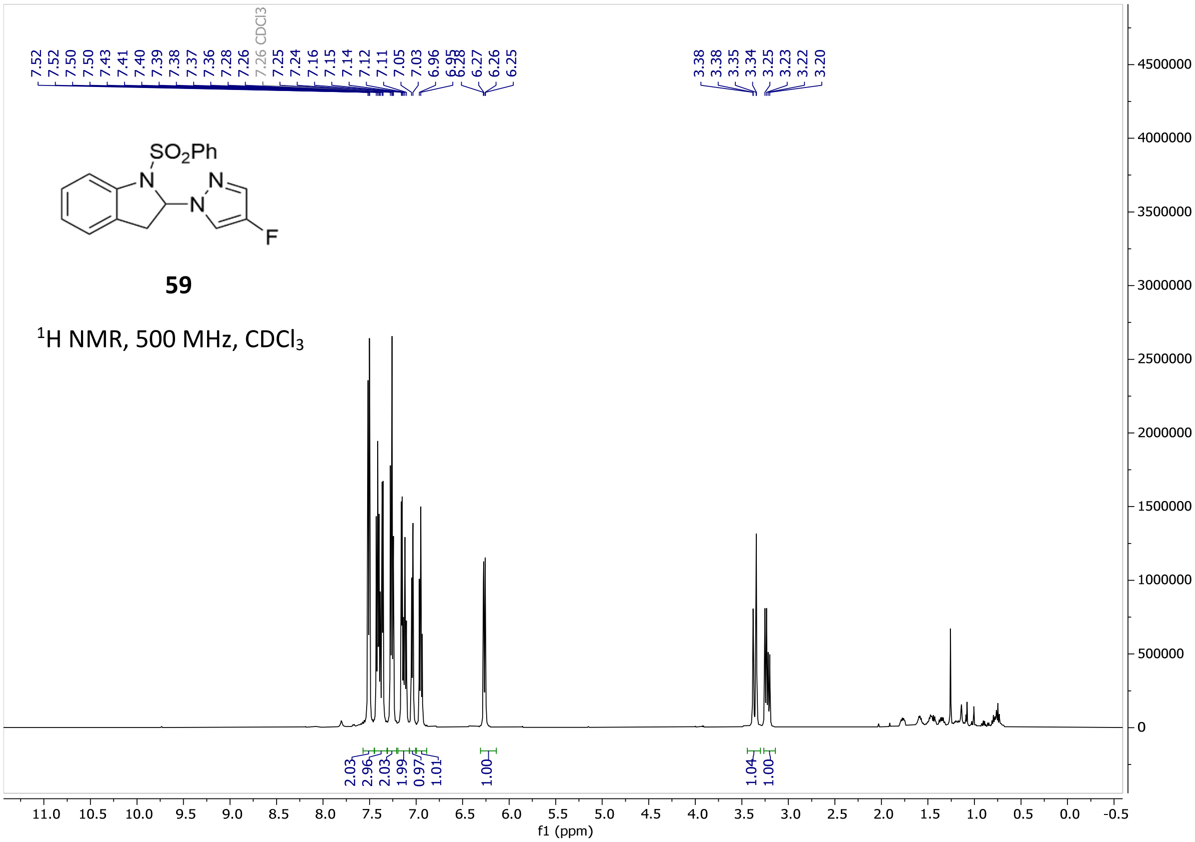

7.52
7.52
7.50
7.50
7.43
7.41
7.40
7.39
7.38
7.37
7.36
7.28
7.26
7.26 CDCl3
7.25
7.24
7.16
7.15
7.14
7.12
7.11
7.05
7.03
6.96
6.95
6.28
6.27
6.26
6.25
3.38
3.38
3.35
3.34
3.25
3.23
3.22
3.20
SO2Ph
N
N
N
F
59
1H NMR, 500 MHz, CDCl3
2.03
2.96
2.03
1.99
0.97
1.01
1.00
1.04
1.00
f1 (ppm)

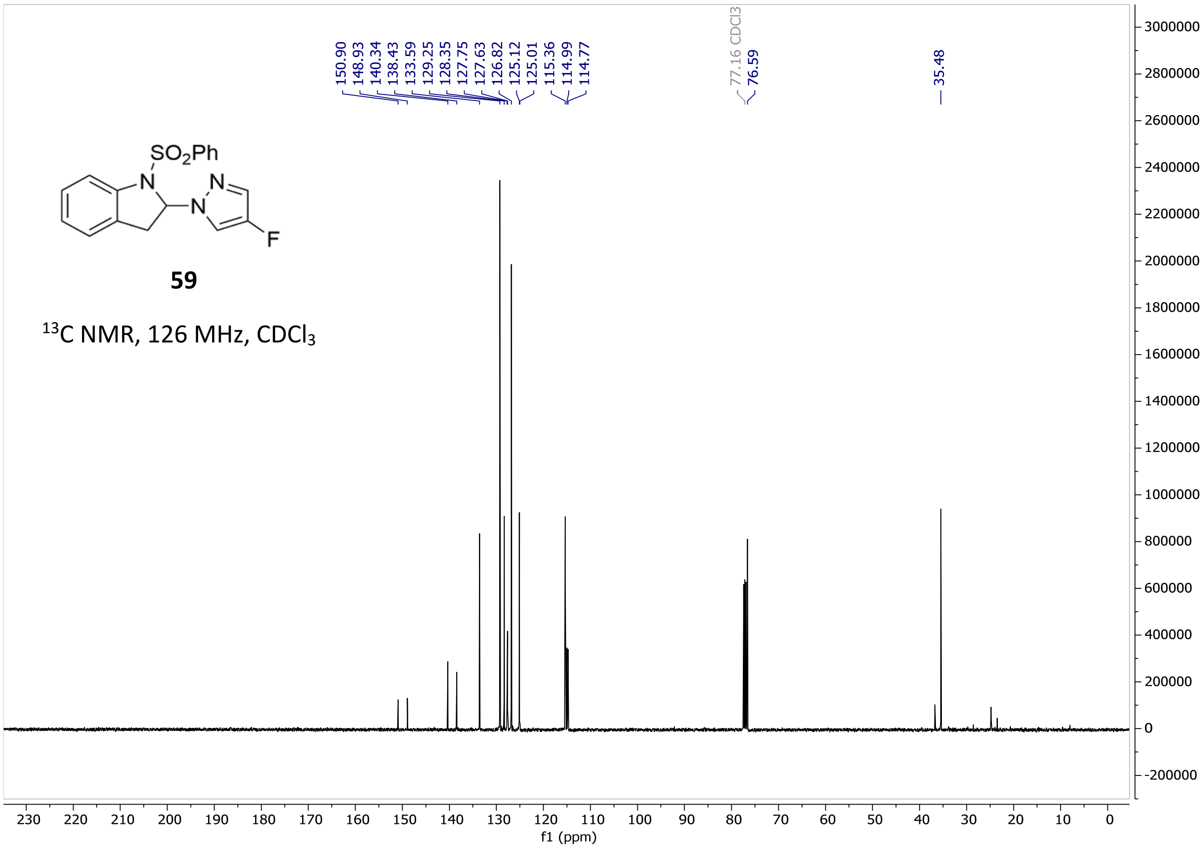

150.90
148.93
140.34
138.43
133.59
129.25
128.35
127.75
127.63
126.82
125.12
125.01
115.36
114.99
114.77
77.16 CDCl3
76.59
35.48
SO2Ph
N
N
N
F
59
13C NMR, 126 MHz, CDCl3
f1 (ppm)

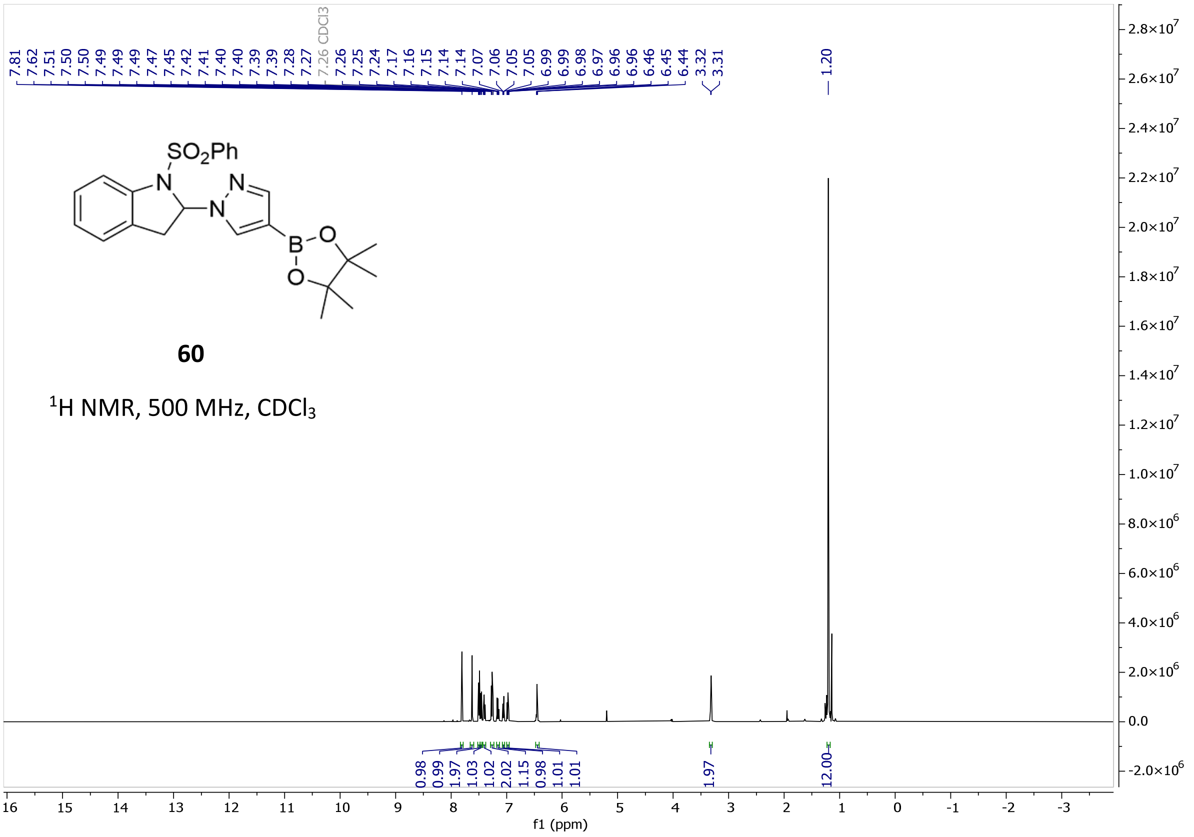
7.81
7.62
7.51
7.50
7.50
7.49
7.49
7.47
7.45
7.42
7.41
7.40
7.40
7.39
7.39
7.28
7.27
7.26 CDCl3
7.26
7.25
7.24
7.17
7.16
7.15
7.14
7.14
7.07
7.06
7.05
7.05
6.99
6.99
6.98
6.97
6.96
6.96
6.46
6.45
6.44
3.32
3.31
1.20
SO2Ph
N
N
N
B
O
O
60
1H NMR, 500 MHz, CDCl3
0.98
0.99
1.97
1.03
1.02
2.02
1.15
0.98
1.01
1.01
1.97
12.00
f1 (ppm)

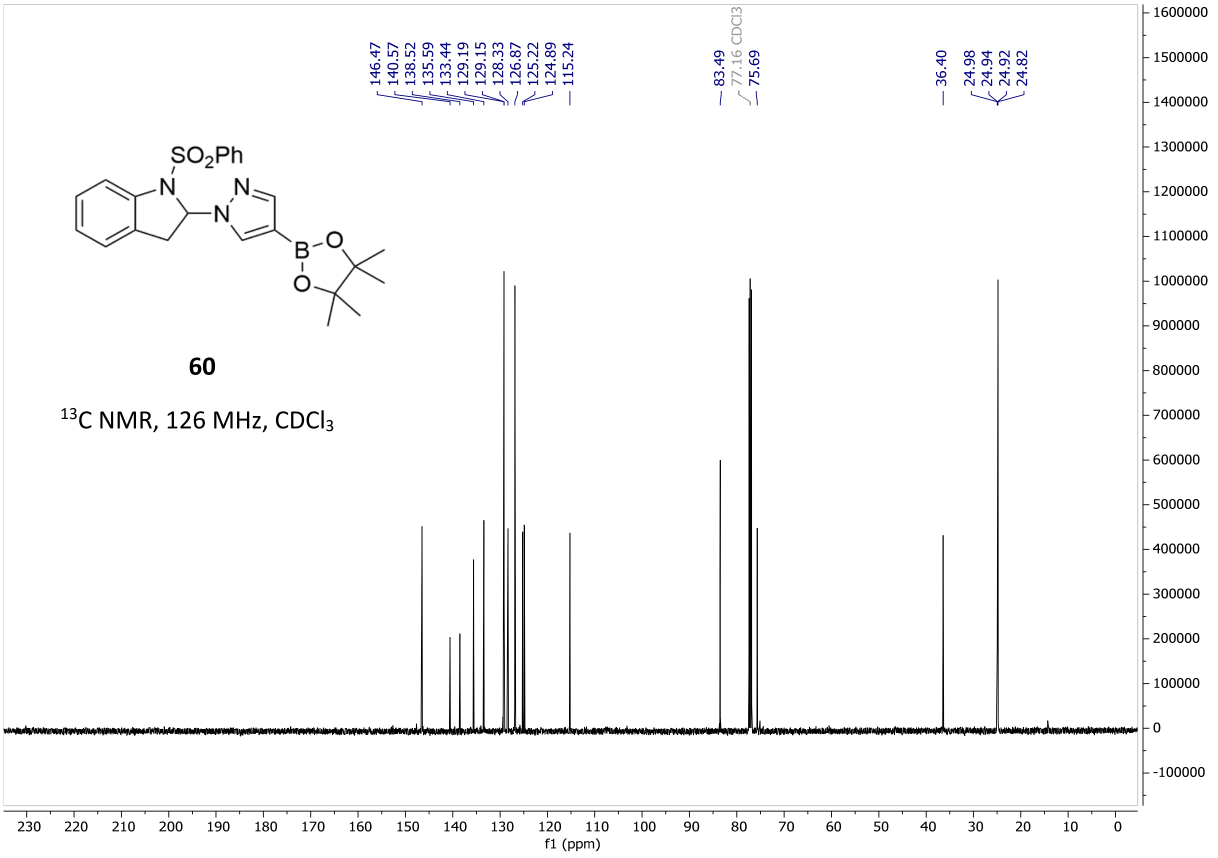
146.47
140.57
138.52
135.59
133.44
129.19
129.15
128.33
126.87
125.22
124.89
115.24
83.49
77.16 CDCl3
75.69
36.40
24.98
24.94
24.92
24.82
SO2Ph
N
N
N
B
O
O
60
13C NMR, 126 MHz, CDCl3
f1 (ppm)

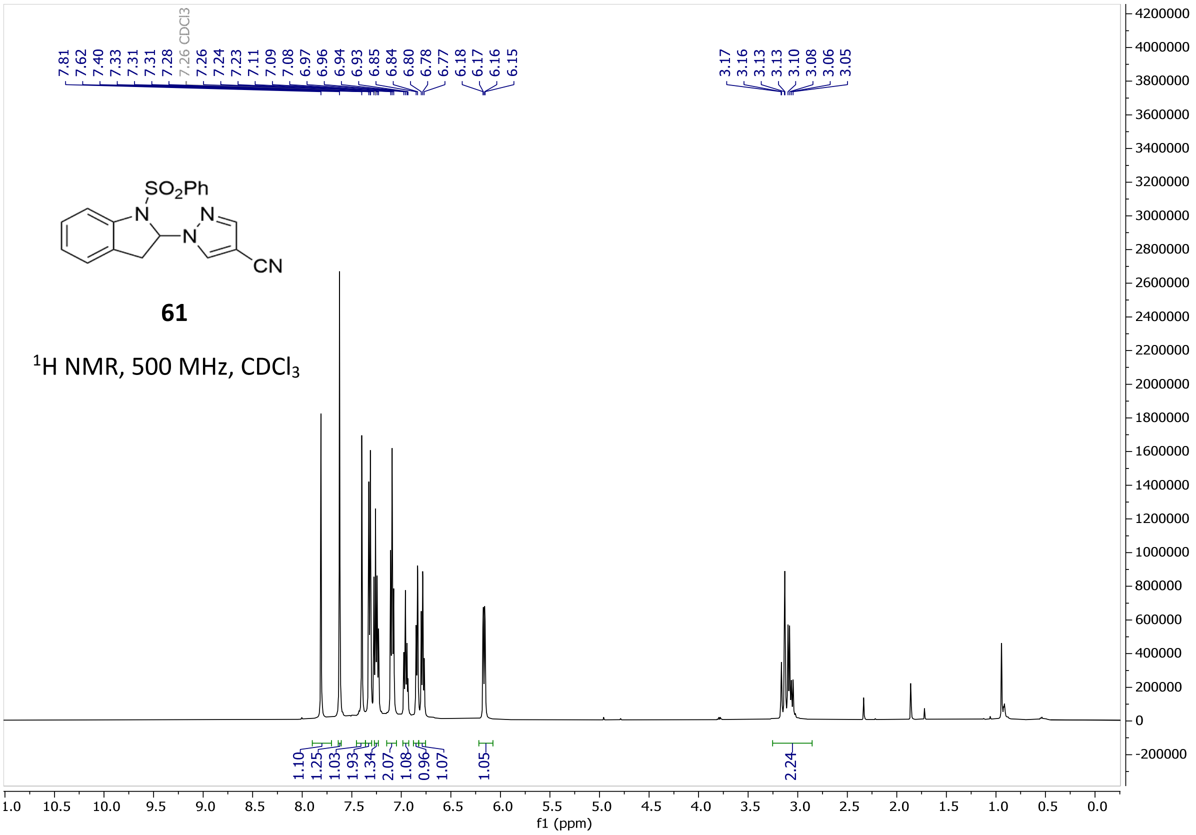
7.81 7.62 7.40 7.33 7.31 7.31 7.28 7.26 CDCl3 7.26 7.24 7.23 7.11 7.09 7.08 6.97 6.96 6.94 6.93 6.85 6.84 6.80 6.78 6.77 6.18 6.17 6.16 6.15
3.17 3.16 3.13 3.13 3.10 3.08 3.06 3.05
SO2Ph
N
N
N
CN
61
1H NMR, 500 MHz, CDCl3
1.10 1.25 1.03 1.93 1.34 2.07 1.08 0.96 1.07 1.05 2.24
f1 (ppm)

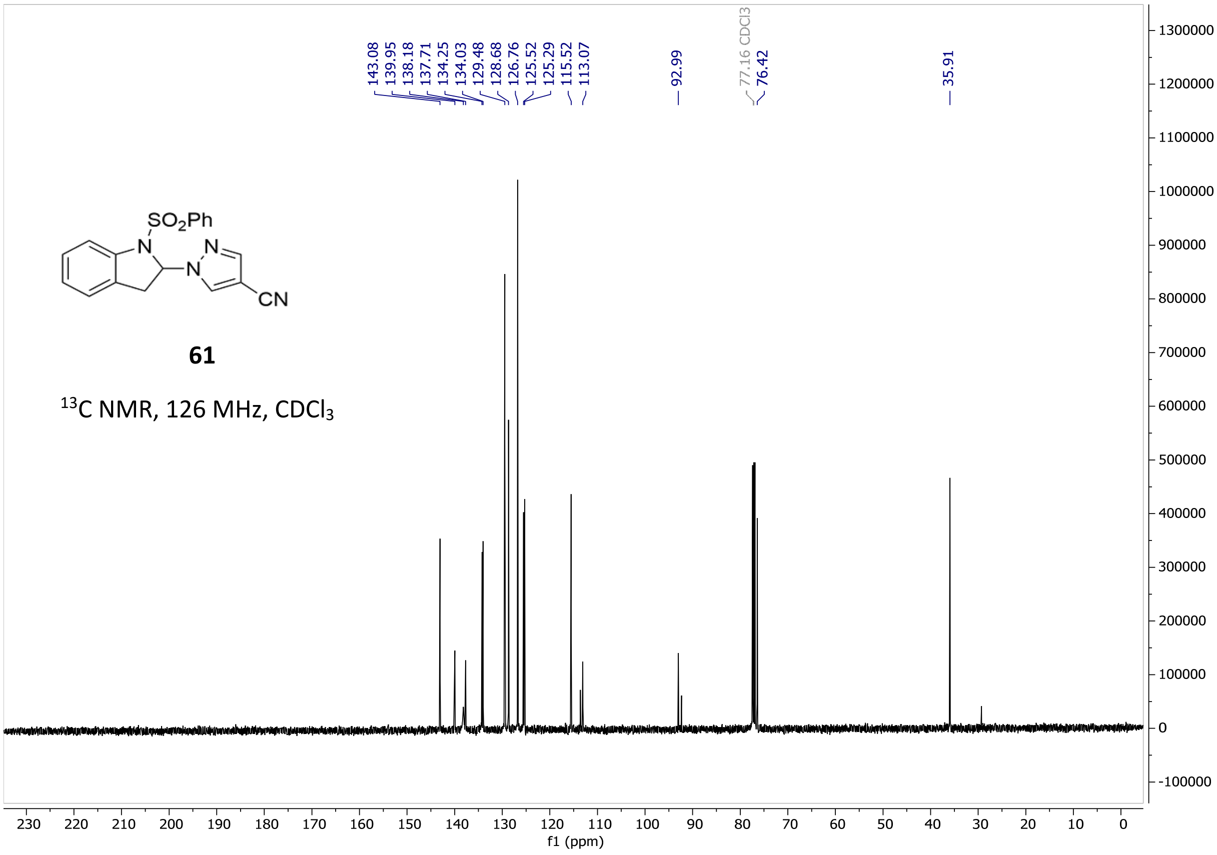
143.08 139.95 138.18 137.71 134.25 134.03 129.48 128.68 126.76 125.52 125.29 115.52 113.07
92.99
77.16 CDCl3 76.42
35.91
SO2Ph
N
N
N
CN
61
13C NMR, 126 MHz, CDCl3
f1 (ppm)

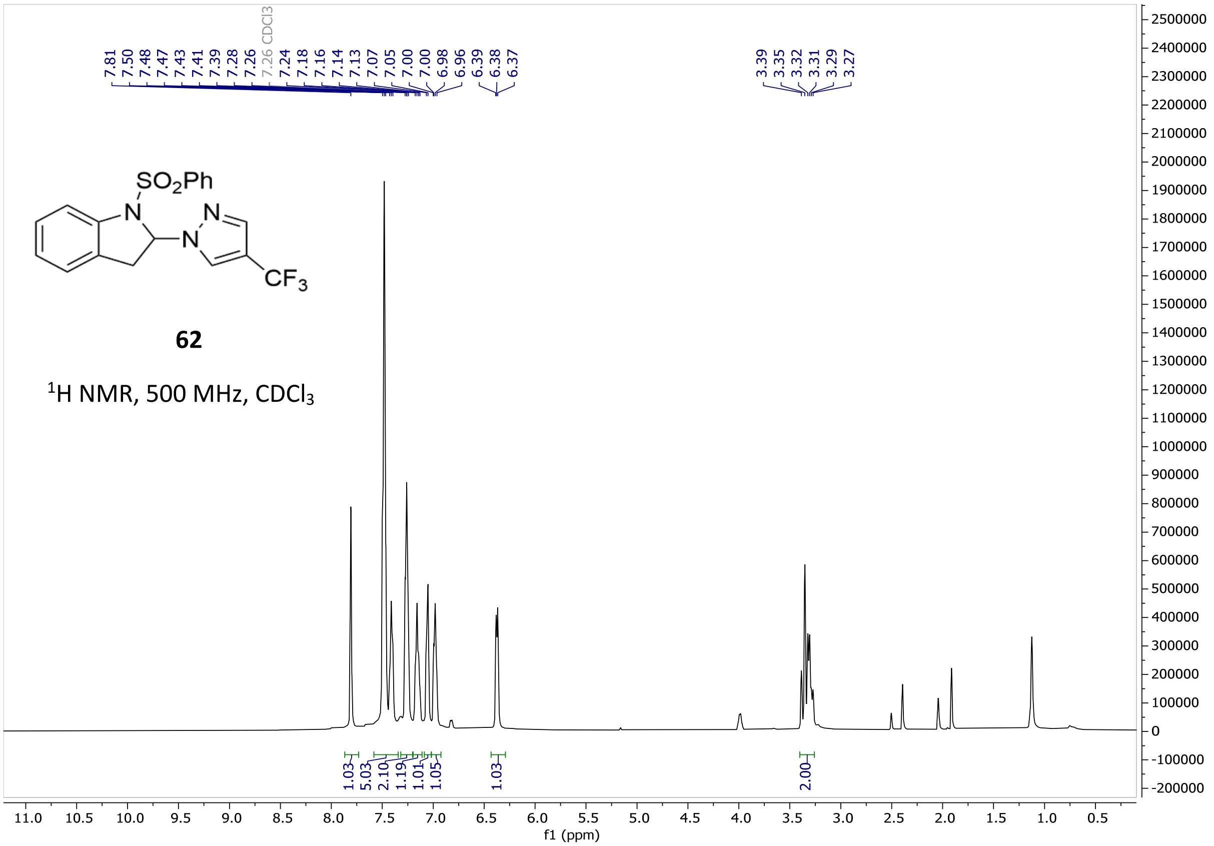

7.81
7.50
7.48
7.47
7.43
7.41
7.39
7.28
7.26
7.26 CDCl3
7.24
7.18
7.16
7.14
7.13
7.07
7.05
7.00
7.00
6.98
6.96
6.39
6.38
6.37
3.39
3.35
3.32
3.31
3.29
3.27
SO2Ph
N
N
N
CF3
62
1H NMR, 500 MHz, CDCl3
1.03
5.03
2.10
1.19
1.01
1.05
1.03
2.00
f1 (ppm)

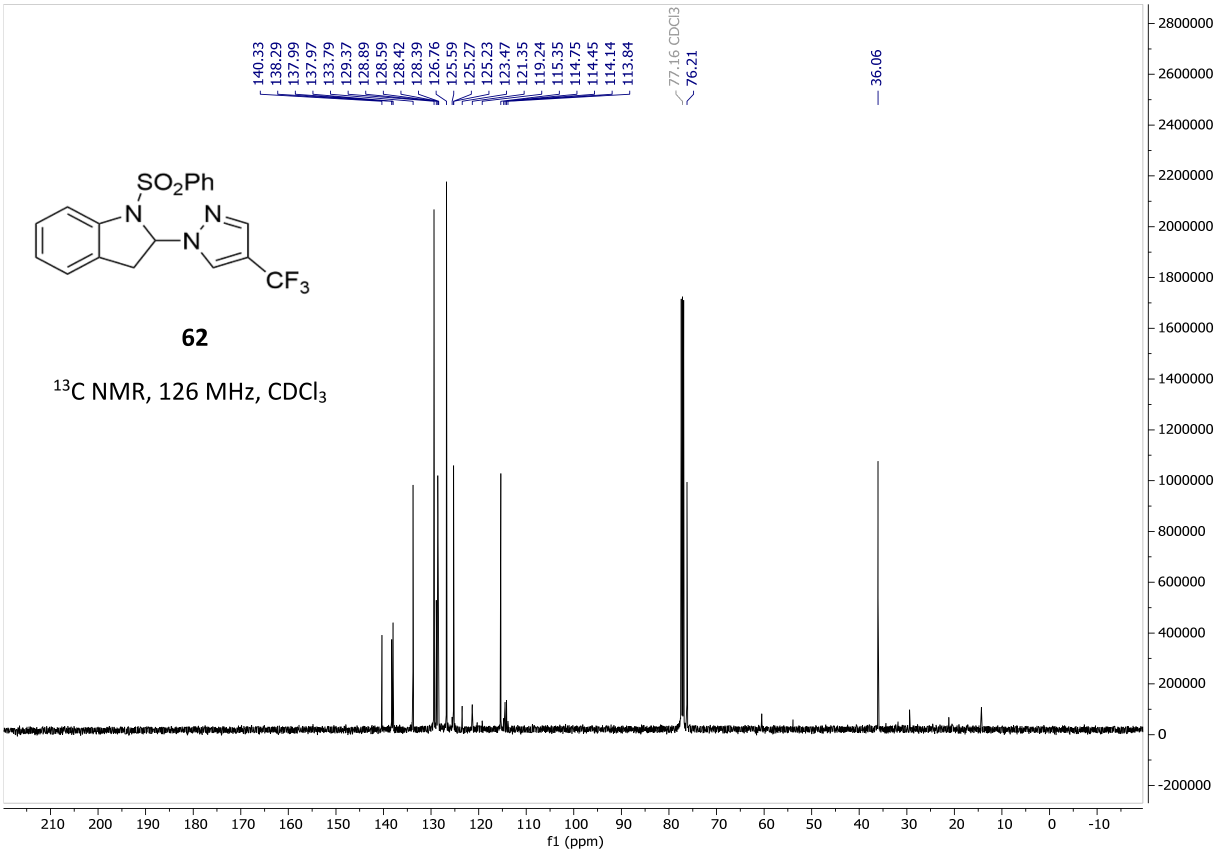

140.33
138.29
137.99
137.97
133.79
129.37
128.89
128.59
128.42
128.39
126.76
125.59
125.27
125.23
123.47
121.35
119.24
115.35
114.75
114.45
114.14
113.84
77.16 CDCl3
76.21
36.06
SO2Ph
N
N
N
CF3
62
13C NMR, 126 MHz, CDCl3
f1 (ppm)

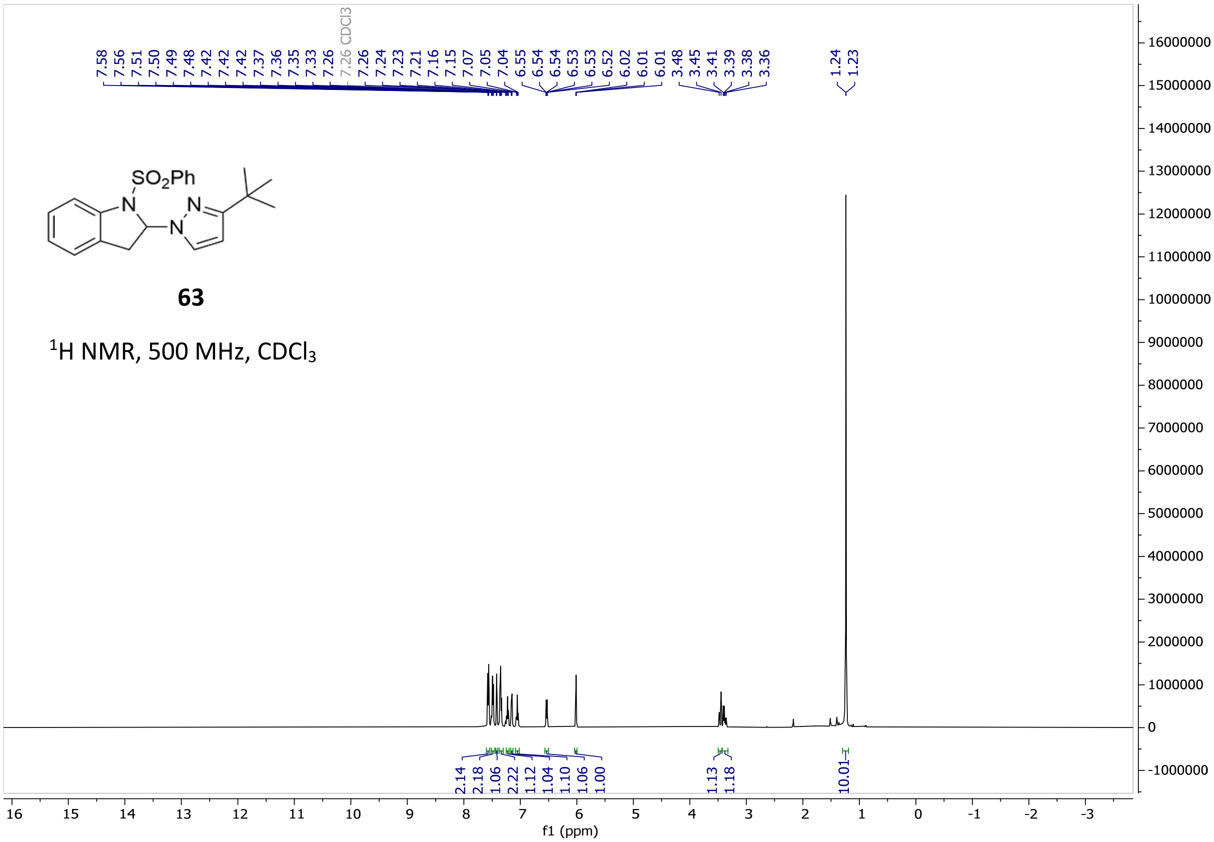
7.58 7.56 7.51 7.50 7.49 7.48 7.42 7.42 7.37 7.36 7.35 7.33 7.26 7.26 7.24 7.23 7.21 7.16 7.15 7.07 7.05 7.04 6.55 6.54 6.54 6.53 6.53 6.52 6.02 6.01 6.01 3.48 3.45 3.41 3.39 3.38 3.36 1.24 1.23
7.26 CDCl3
SO2Ph
N
N
N
63
1H NMR, 500 MHz, CDCl3
2.14 2.18 1.06 2.22 1.12 1.04 1.10 1.06 1.00 1.13 1.18 10.01
f1 (ppm)

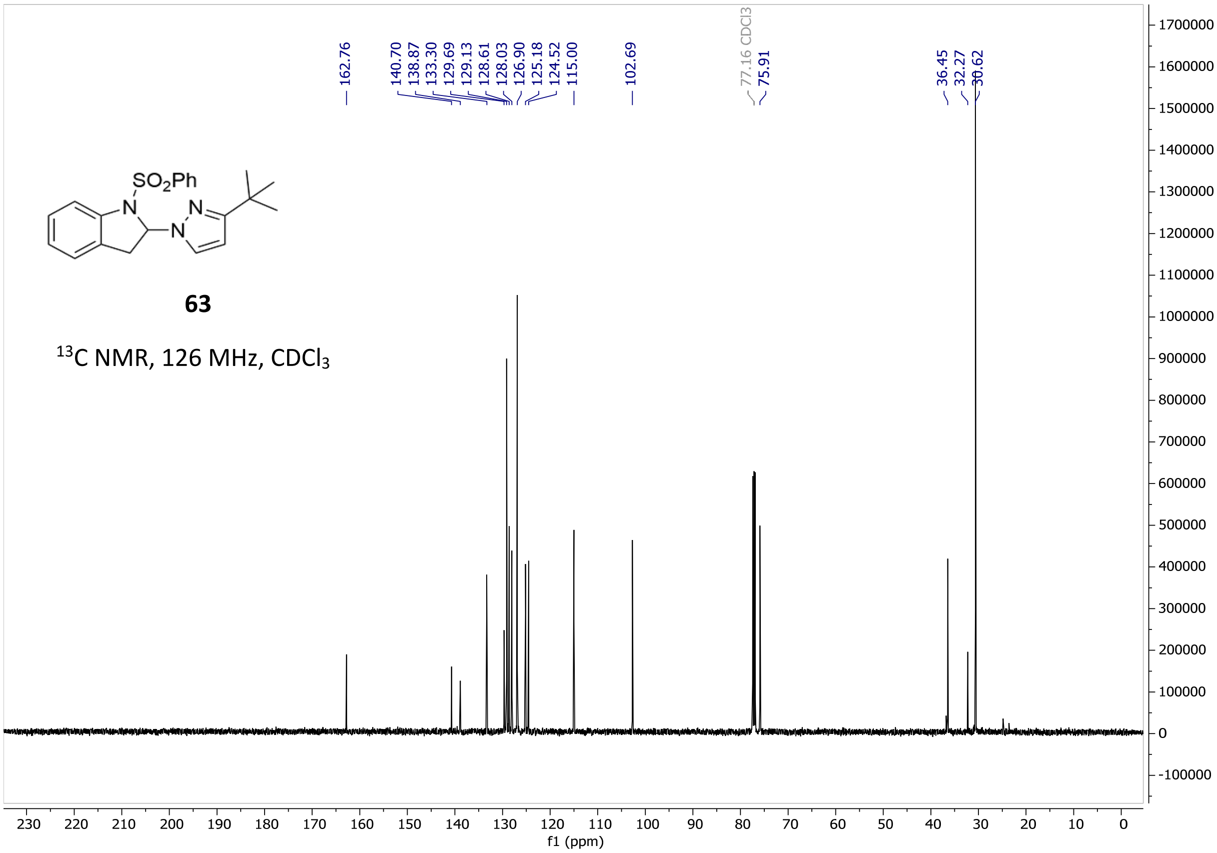
162.76
140.70 138.87 133.30 129.69 129.13 128.61 128.03 126.90 125.18 124.52 115.00
102.69
77.16 CDCl3
75.91
36.45 32.27 30.62
SO2Ph
N
N
N
63
13C NMR, 126 MHz, CDCl3
f1 (ppm)

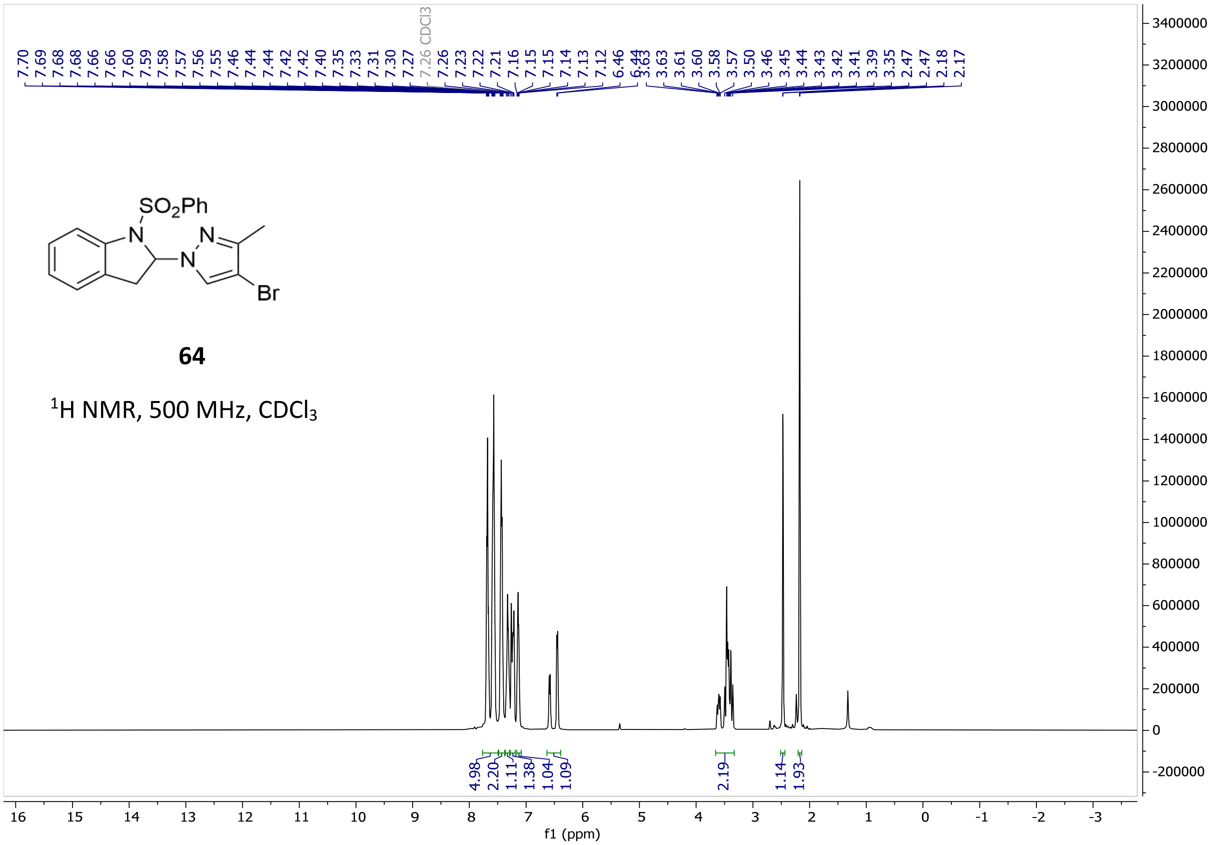
7.70 7.69 7.68 7.66 7.66 7.60 7.59 7.58 7.57 7.56 7.55 7.46 7.44 7.44 7.42 7.42 7.40 7.35 7.33 7.31 7.30 7.27 7.26 CDCl3 7.26 7.23 7.22 7.21 7.16 7.15 7.15 7.14 7.13 7.12 6.46 6.44 3.63 3.63 3.61 3.60 3.58 3.57 3.50 3.46 3.45 3.44 3.43 3.42 3.41 3.39 3.35 2.47 2.47 2.18 2.17
SO2Ph
N
N
N
Br
64
1H NMR, 500 MHz, CDCl3
4.98 2.20 1.11 1.38 1.04 1.09 2.19 1.14 1.93
f1 (ppm)

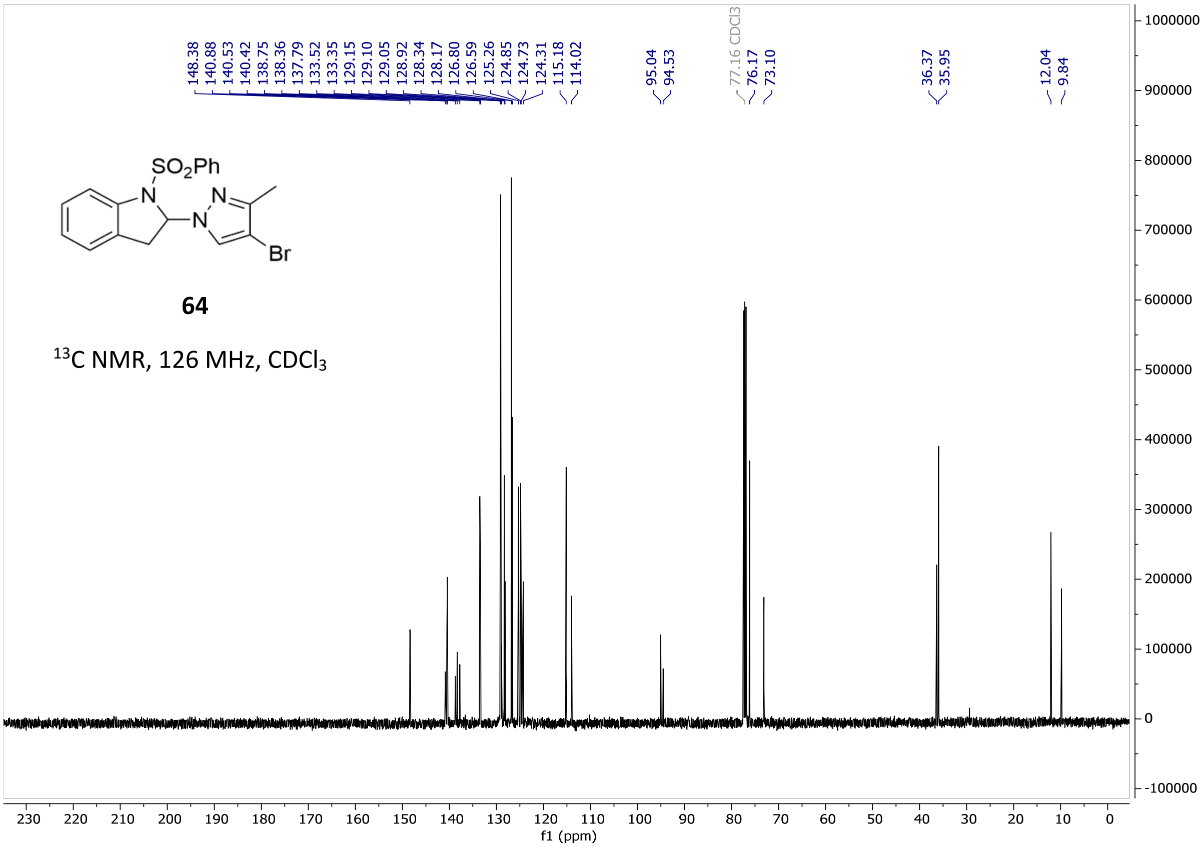
148.38 140.88 140.53 140.42 138.75 138.36 137.79 133.52 133.35 129.15 129.10 129.05 128.92 128.34 128.17 126.80 126.59 125.26 124.85 124.73 124.31 115.18 114.02 95.04 94.53 77.16 CDCl3 76.17 73.10 36.37 35.95 12.04 9.84
SO2Ph
N
N
N
Br
64
13C NMR, 126 MHz, CDCl3
f1 (ppm)

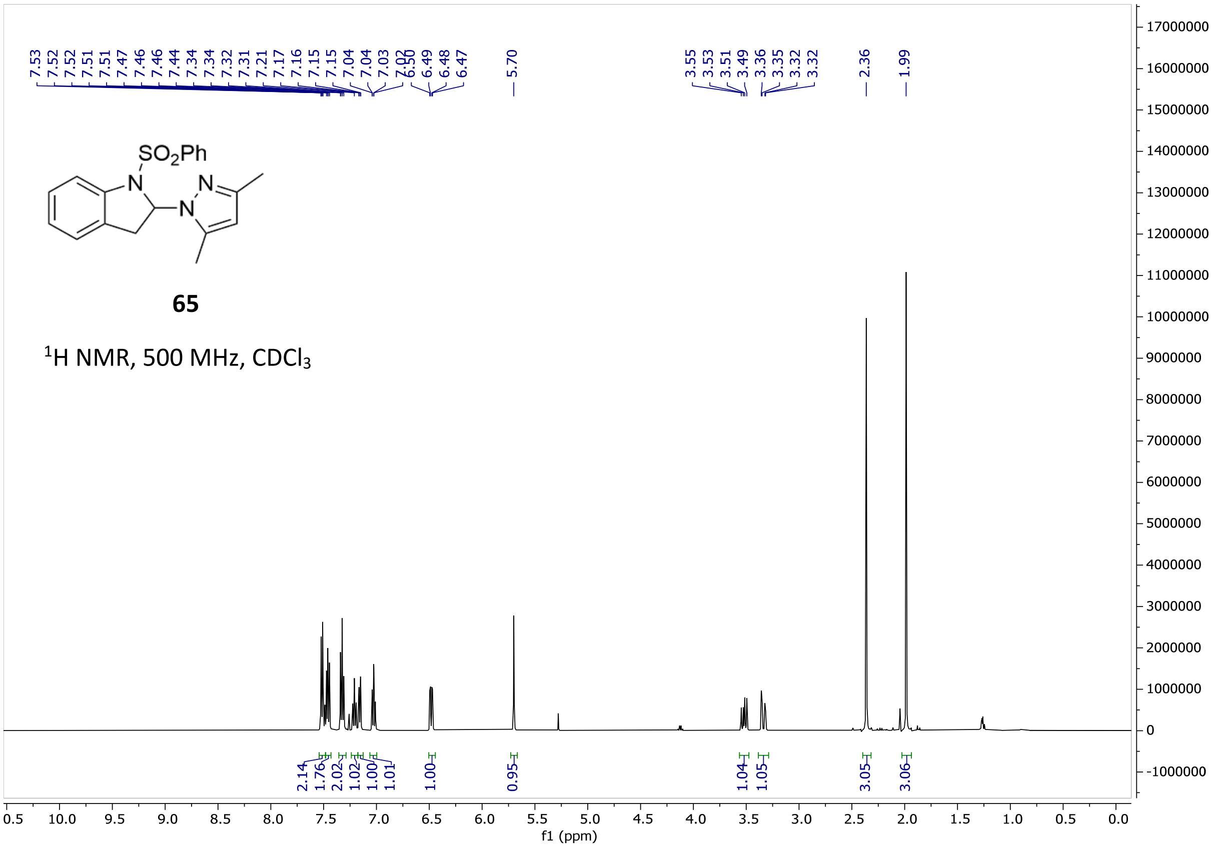
7.53 7.52 7.52 7.51 7.51 7.47 7.46 7.46 7.44 7.34 7.34 7.32 7.31 7.21 7.17 7.16 7.15 7.15 7.04 7.04 7.03 7.02 6.50 6.49 6.48 6.47
5.70
3.55 3.53 3.51 3.49 3.36 3.35 3.32 3.32
2.36
1.99
SO2Ph
N
N
N
65
1H NMR, 500 MHz, CDCl3
2.14 1.76 2.02 1.02 1.00 1.01
1.00
0.95
1.04 1.05
3.05
3.06
f1 (ppm)

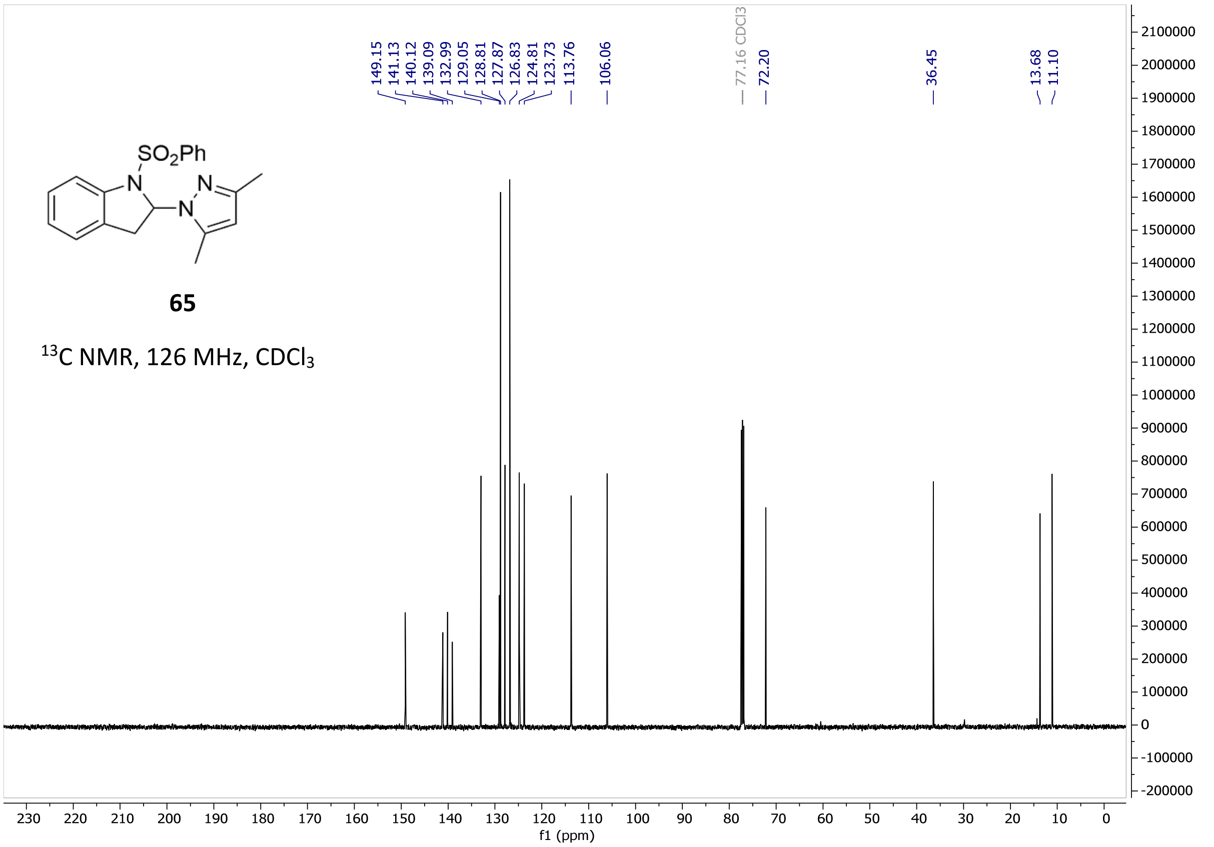
149.15 141.13 140.12 139.09 132.99 129.05 128.81 127.87 126.83 124.81 123.73 113.76 106.06
72.20
36.45
13.68 11.10
SO2Ph
N
N
N
65
13C NMR, 126 MHz, CDCl3
f1 (ppm)

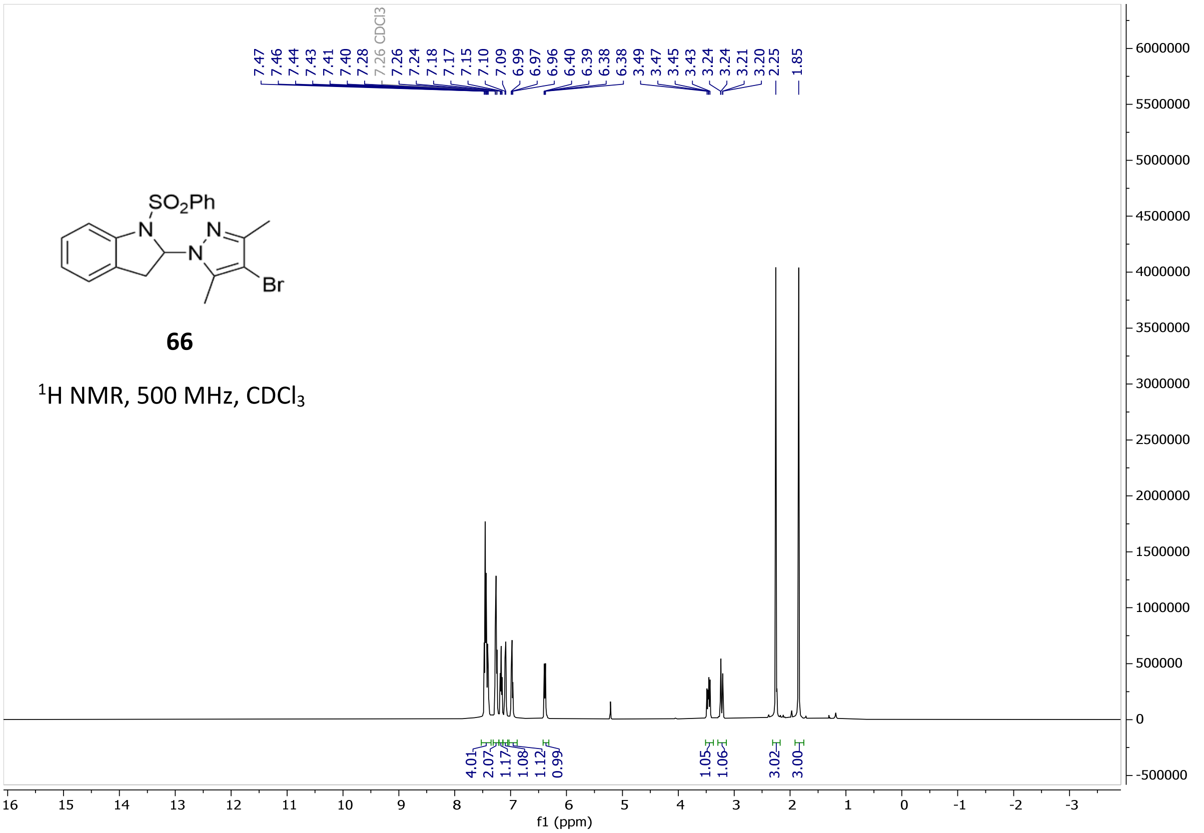
7.47 7.46 7.44 7.43 7.41 7.40 7.28 7.26 CDCl3 7.26 7.24 7.18 7.17 7.15 7.10 7.09 6.99 6.97 6.96 6.40 6.39 6.38 6.38 3.49 3.47 3.45 3.43 3.24 3.24 3.21 3.20 2.25 1.85
SO2Ph
N
N
Br
66
1H NMR, 500 MHz, CDCl3
4.01 2.07 1.17 1.08 1.12 0.99 1.05 1.06 3.02 3.00
f1 (ppm)

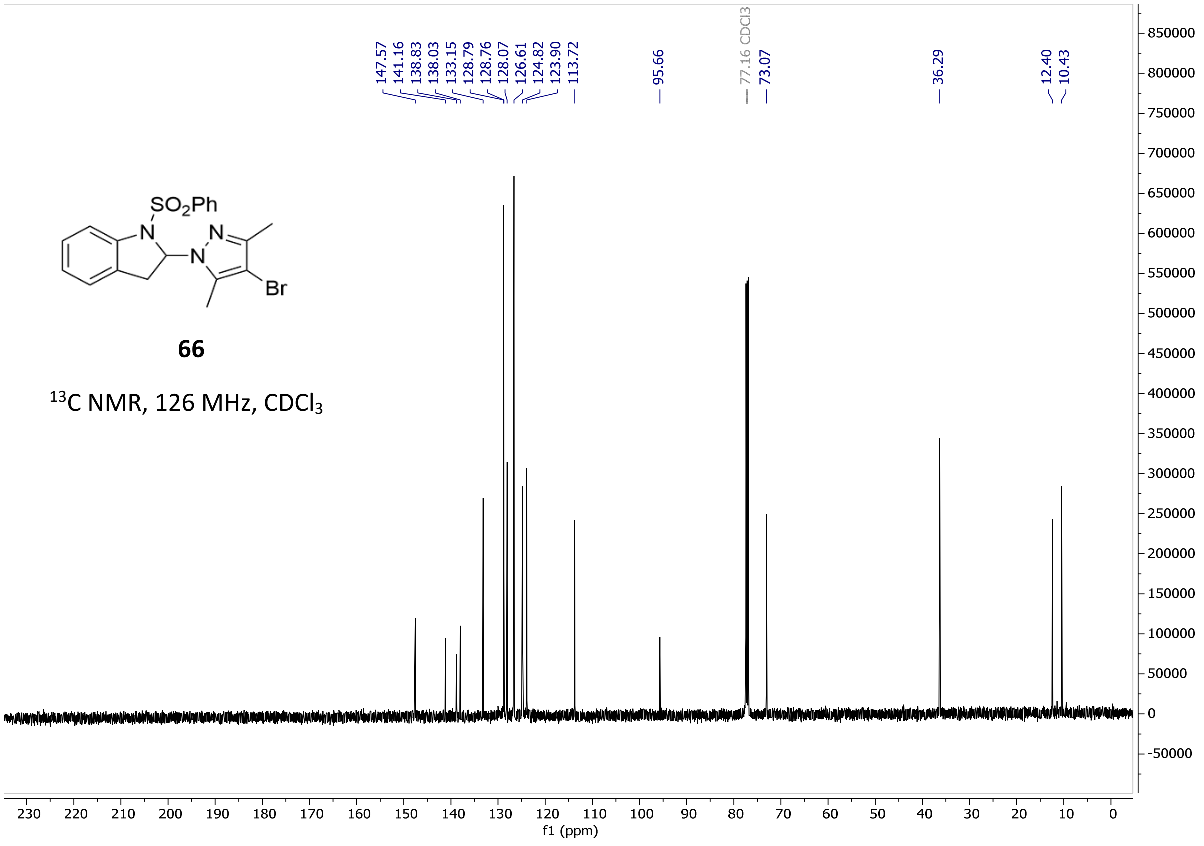
147.57 141.16 138.83 138.03 133.15 128.79 128.76 128.07 126.61 124.82 123.90 113.72 95.66 77.16 CDCl3 73.07 36.29 12.40 10.43
SO2Ph
N
N
Br
66
13C NMR, 126 MHz, CDCl3
f1 (ppm)

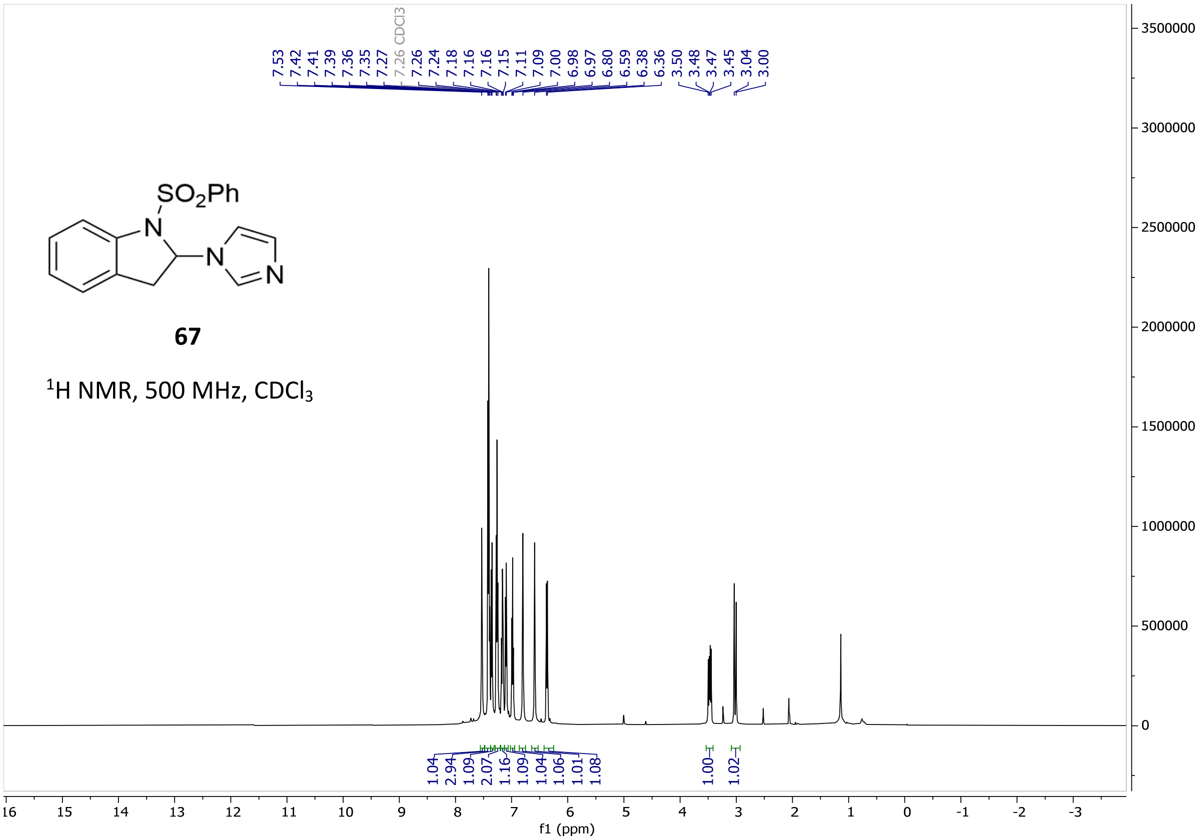

7.53 7.42 7.41 7.39 7.36 7.35 7.27 7.26 CDCl3 7.26 7.24 7.18 7.16 7.16 7.15 7.11 7.09 7.00 6.98 6.97 6.80 6.59 6.38 6.36 3.50 3.48 3.47 3.45 3.04 3.00
SO2Ph
N
N
N
67
1H NMR, 500 MHz, CDCl3
1.04 2.94 1.09 2.07 1.16 1.09 1.04 1.06 1.01 1.08
1.00 1.02
f1 (ppm)

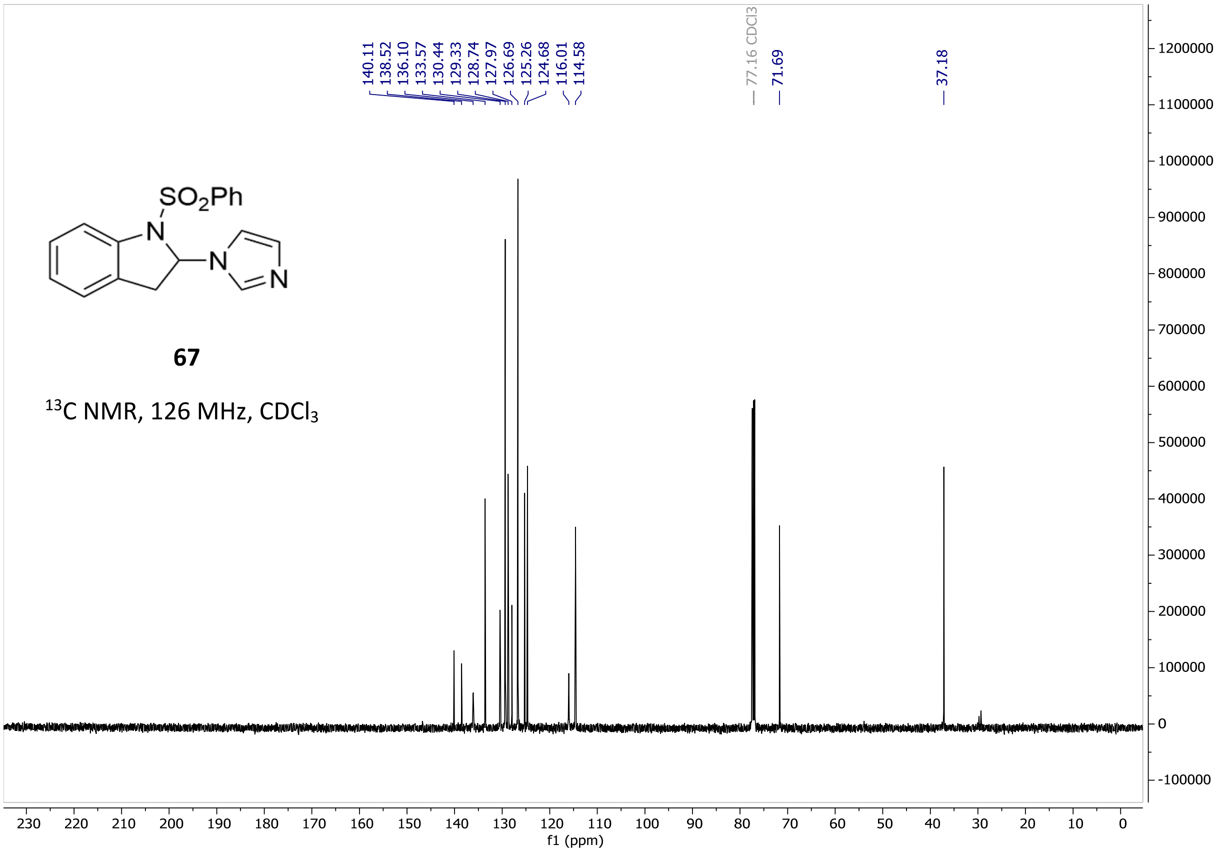

140.11 138.52 136.10 133.57 130.44 129.33 128.74 127.97 126.69 125.26 124.68 116.01 114.58
77.16 CDCl3
71.69
37.18
SO2Ph
N
N
N
67
13C NMR, 126 MHz, CDCl3
f1 (ppm)

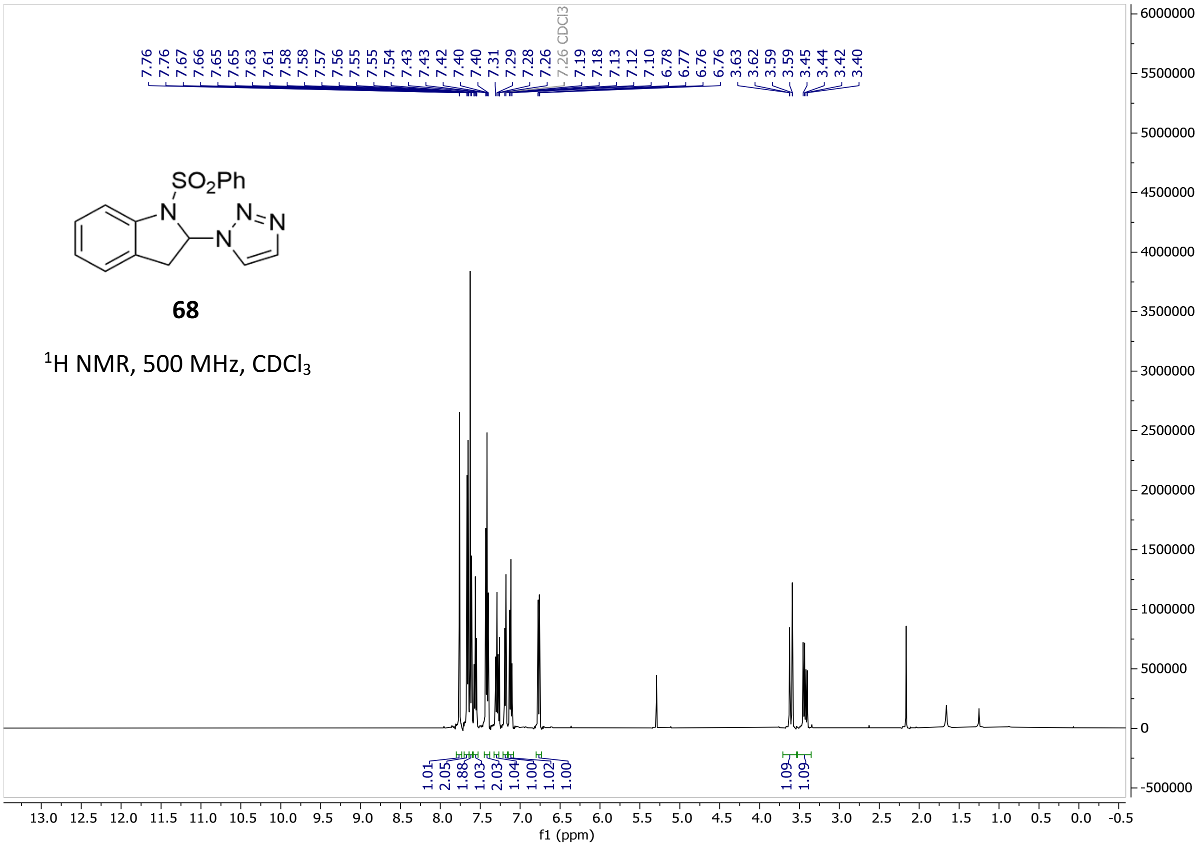
7.76
7.76
7.67
7.66
7.65
7.65
7.63
7.61
7.58
7.58
7.57
7.56
7.55
7.55
7.54
7.43
7.43
7.42
7.40
7.40
7.31
7.29
7.28
7.26 CDCl3
7.19
7.18
7.13
7.12
7.10
6.78
6.77
6.76
6.76
3.63
3.62
3.59
3.59
3.45
3.44
3.42
3.40
SO2Ph
N
N
N
N
68
1H NMR, 500 MHz, CDCl3
1.01
2.05
1.88
1.03
2.03
1.04
1.00
1.02
1.00
1.09
1.09
f1 (ppm)

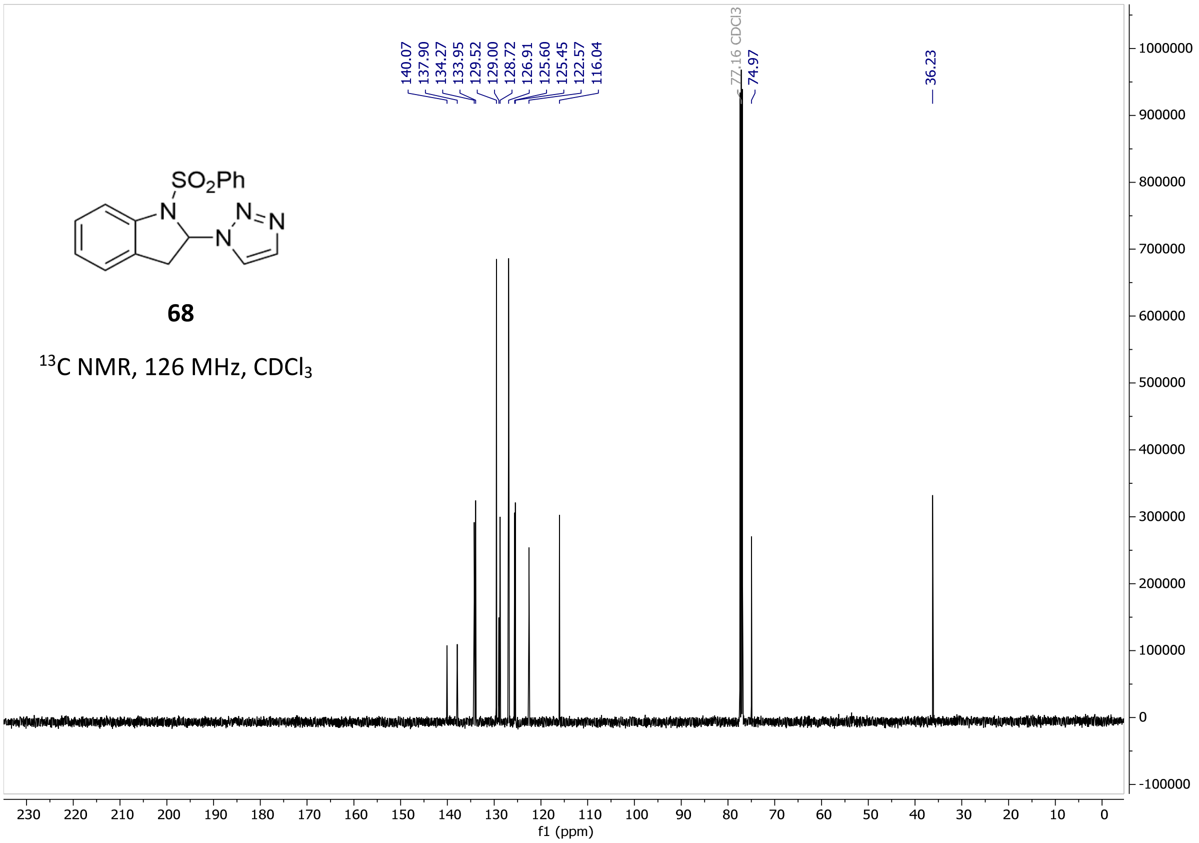
140.07
137.90
134.27
133.95
129.52
129.00
128.72
126.91
125.60
125.45
122.57
116.04
77.16 CDCl3
74.97
36.23
SO2Ph
N
N
N
N
68
13C NMR, 126 MHz, CDCl3
f1 (ppm)

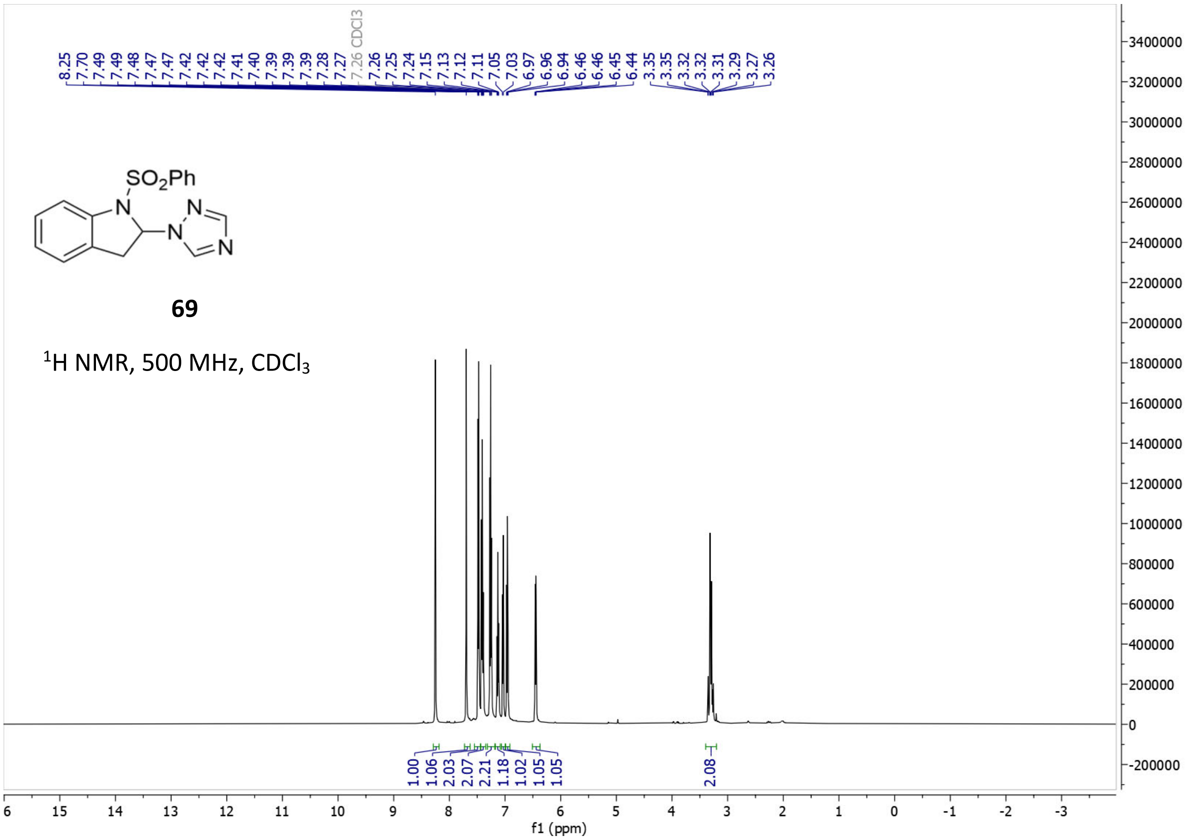

SO2Ph
N
N
N
N
69
1H NMR, 500 MHz, CDCl3
8.25 7.70 7.49 7.49 7.48 7.47 7.47 7.42 7.42 7.42 7.41 7.40 7.39 7.39 7.39 7.28 7.27 7.26 CDCl3 7.26 7.25 7.24 7.15 7.13 7.12 7.11 7.05 7.03 6.97 6.96 6.94 6.46 6.46 6.45 6.44 3.35 3.35 3.32 3.32 3.31 3.29 3.27 3.26
1.00 1.06 2.03 2.07 2.21 1.18 1.02 1.05 1.05 2.08
f1 (ppm)

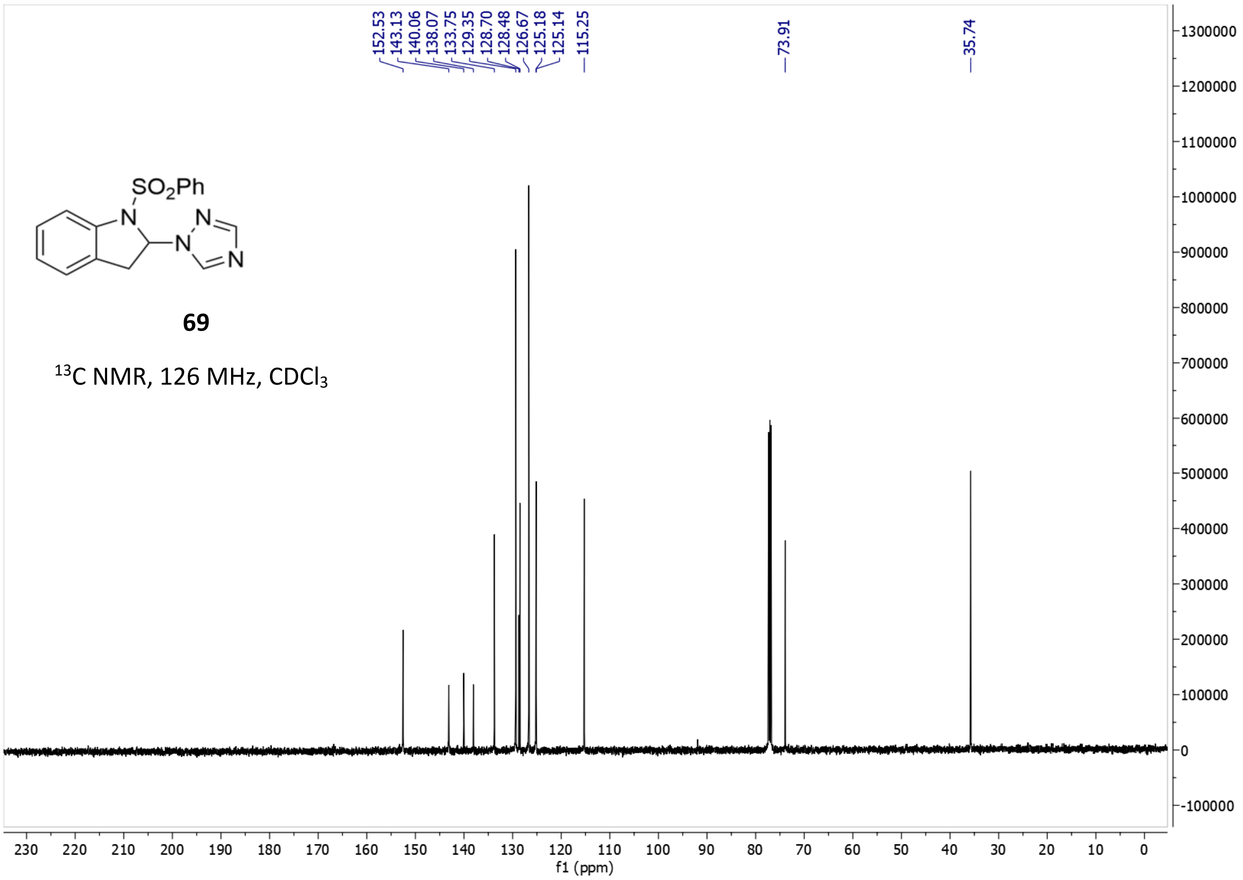

SO2Ph
N
N
N
N
69
13C NMR, 126 MHz, CDCl3
152.53 143.13 140.06 138.07 133.75 129.35 128.70 128.48 126.67 125.18 125.14 115.25
73.91
35.74
f1 (ppm)

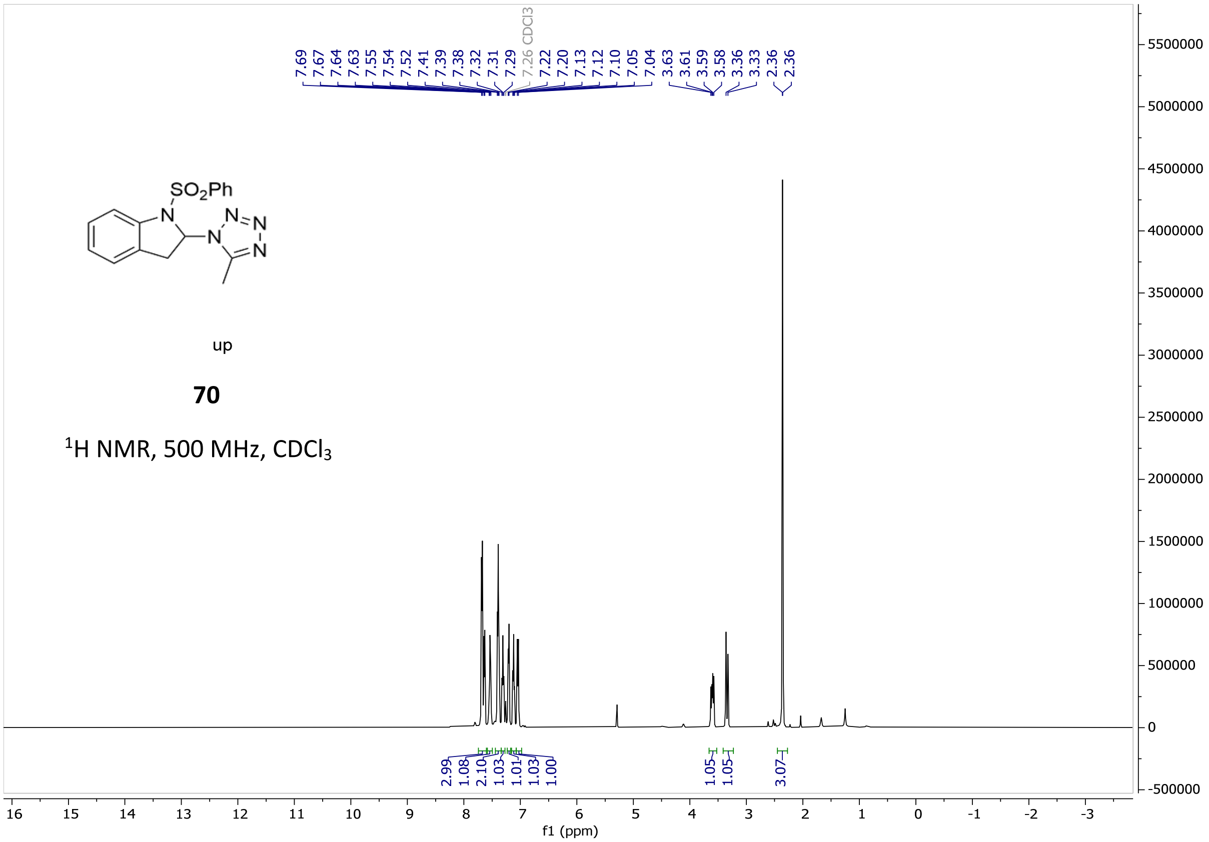
7.69 7.67 7.64 7.63 7.55 7.54 7.52 7.41 7.39 7.38 7.32 7.31 7.29 7.26 CDCl3 7.22 7.20 7.13 7.12 7.10 7.05 7.04 3.63 3.61 3.59 3.58 3.36 3.33 2.36 2.36
SO2Ph
N
N
N
N
N
N
up
70
1H NMR, 500 MHz, CDCl3
2.99 1.08 2.10 1.03 1.01 1.03 1.00 1.05 1.05 3.07
f1 (ppm)

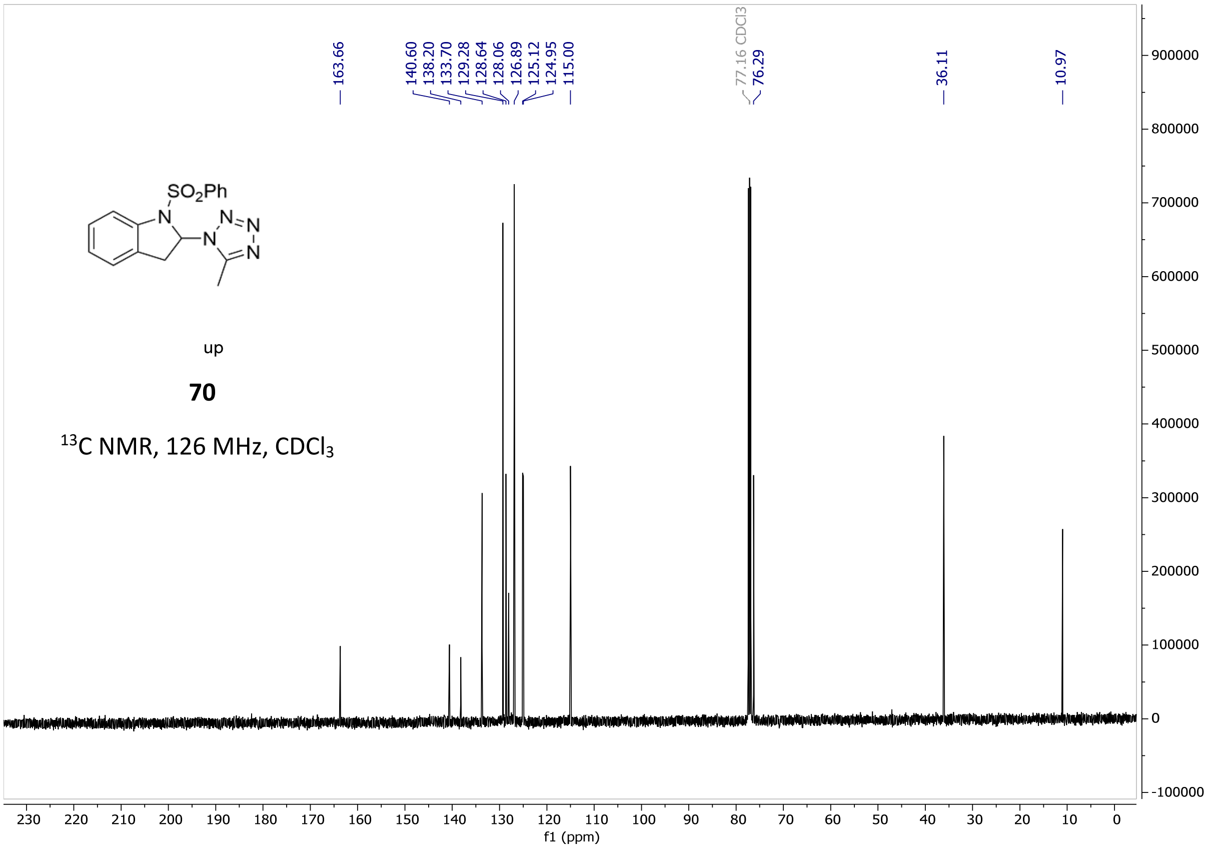
163.66
140.60 138.20 133.70 129.28 128.64 128.06 126.89 125.12 124.95 115.00
77.16 CDCl3 76.29
36.11
10.97
SO2Ph
N
N
N
N
N
N
up
70
13C NMR, 126 MHz, CDCl3
f1 (ppm)

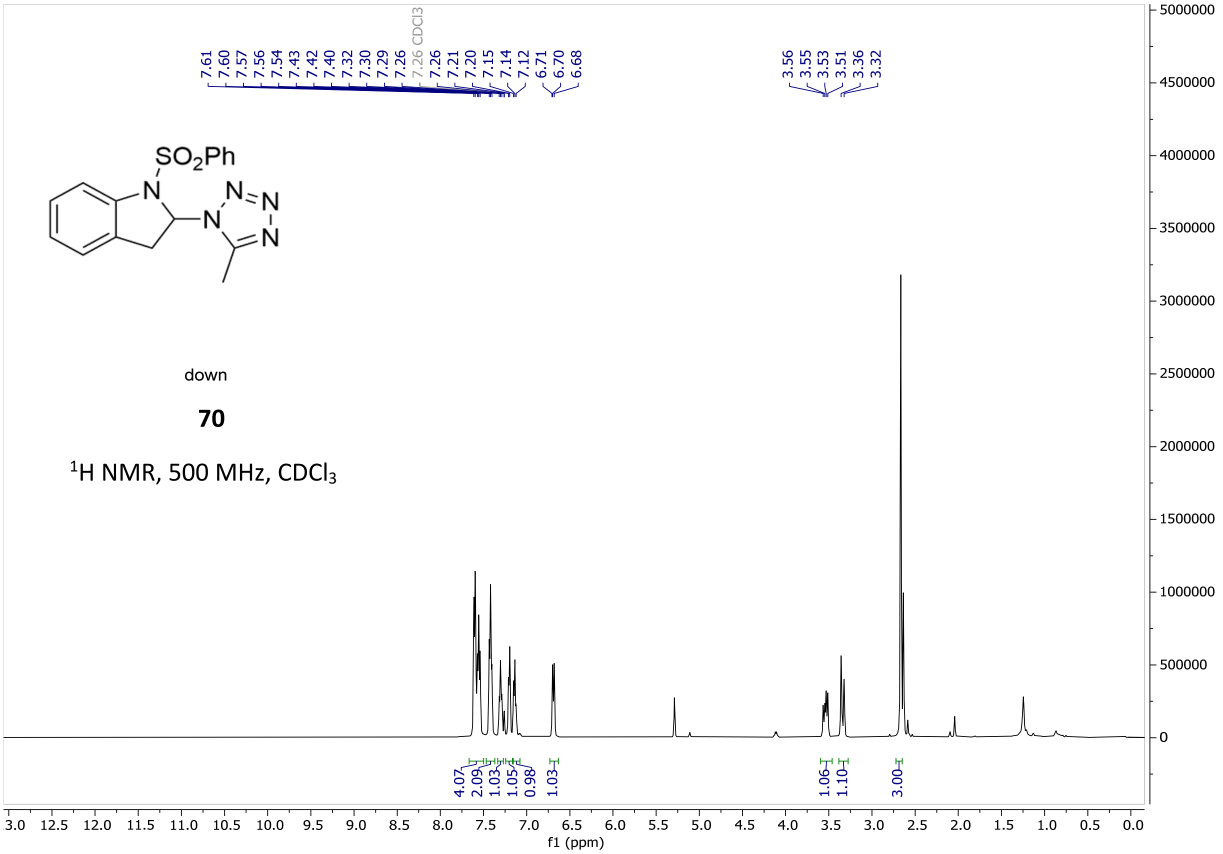
7.61
7.60
7.57
7.56
7.54
7.43
7.42
7.40
7.32
7.30
7.29
7.26
7.26 CDCl3
7.26
7.21
7.20
7.15
7.14
7.12
6.71
6.70
6.68
3.56
3.55
3.53
3.51
3.36
3.32
SO2Ph
N
N
N
N
N
down
70
1H NMR, 500 MHz, CDCl3
4.07
2.09
1.03
1.05
0.98
1.03
1.06
1.10
3.00
f1 (ppm)

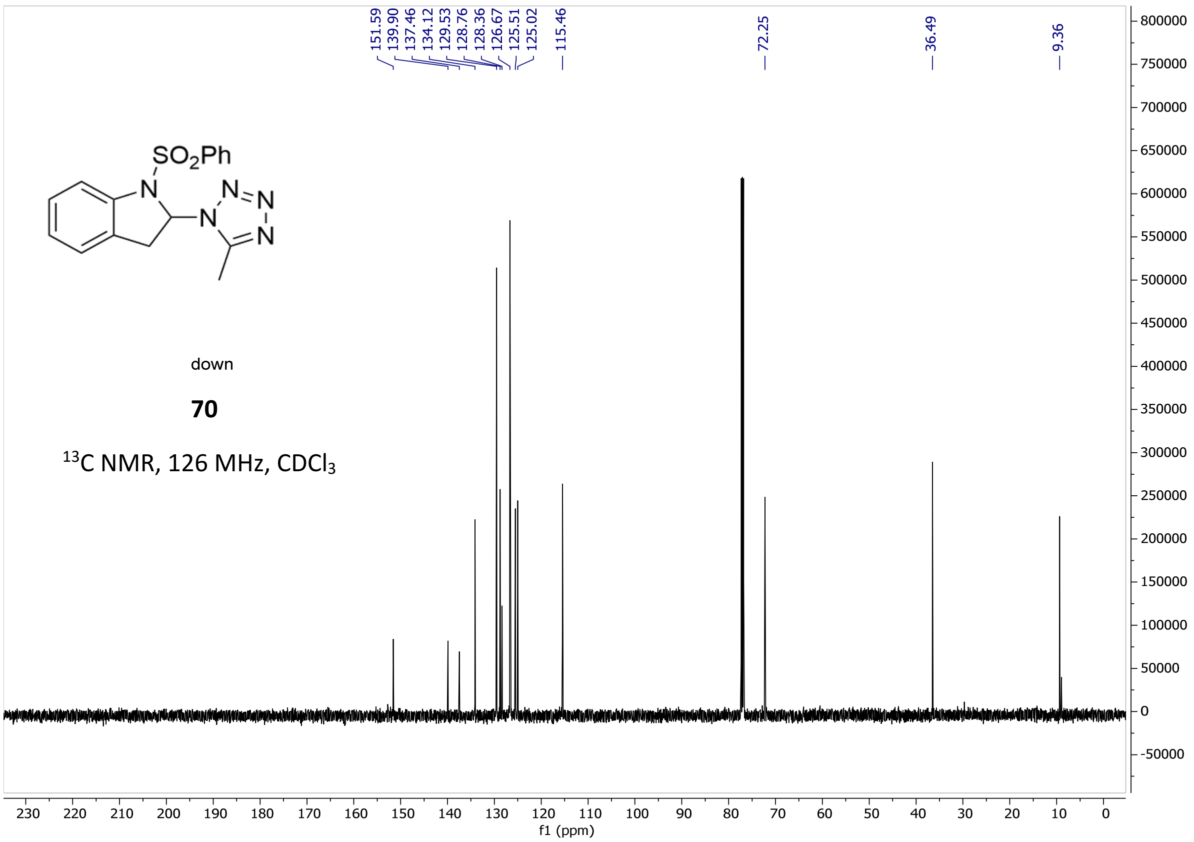
151.59
139.90
137.46
134.12
129.53
128.76
128.36
126.67
125.51
125.02
115.46
72.25
36.49
9.36
SO2Ph
N
N
N
N
N
down
70
13C NMR, 126 MHz, CDCl3
f1 (ppm)

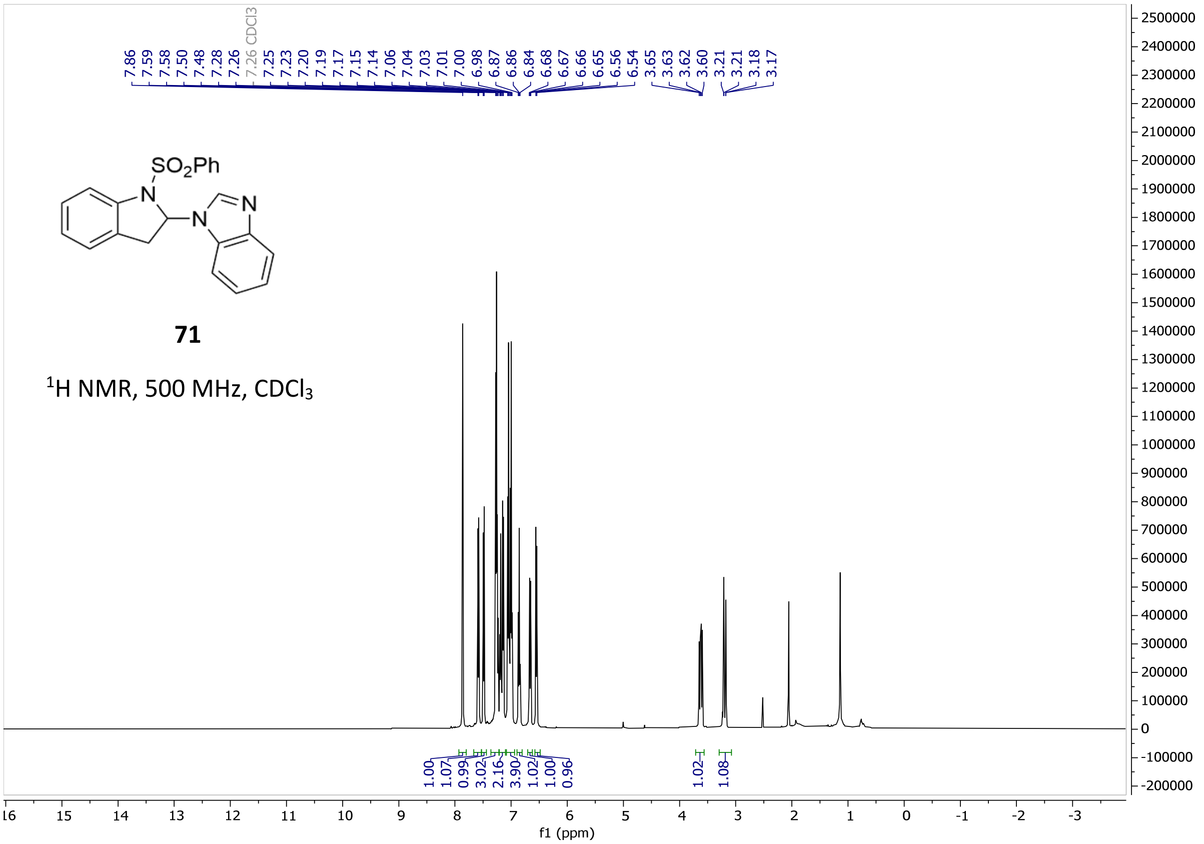
7.86
7.59
7.58
7.50
7.48
7.28
7.26
7.26 CDCl3
7.25
7.23
7.20
7.19
7.17
7.15
7.14
7.06
7.04
7.03
7.01
7.00
6.98
6.87
6.86
6.84
6.68
6.67
6.66
6.65
6.56
6.54
3.65
3.63
3.62
3.60
3.21
3.21
3.18
3.17
SO2Ph
N
N
N
71
1H NMR, 500 MHz, CDCl3
1.00
1.07
0.99
3.02
2.16
3.90
1.02
1.00
0.96
1.02
1.08
f1 (ppm)

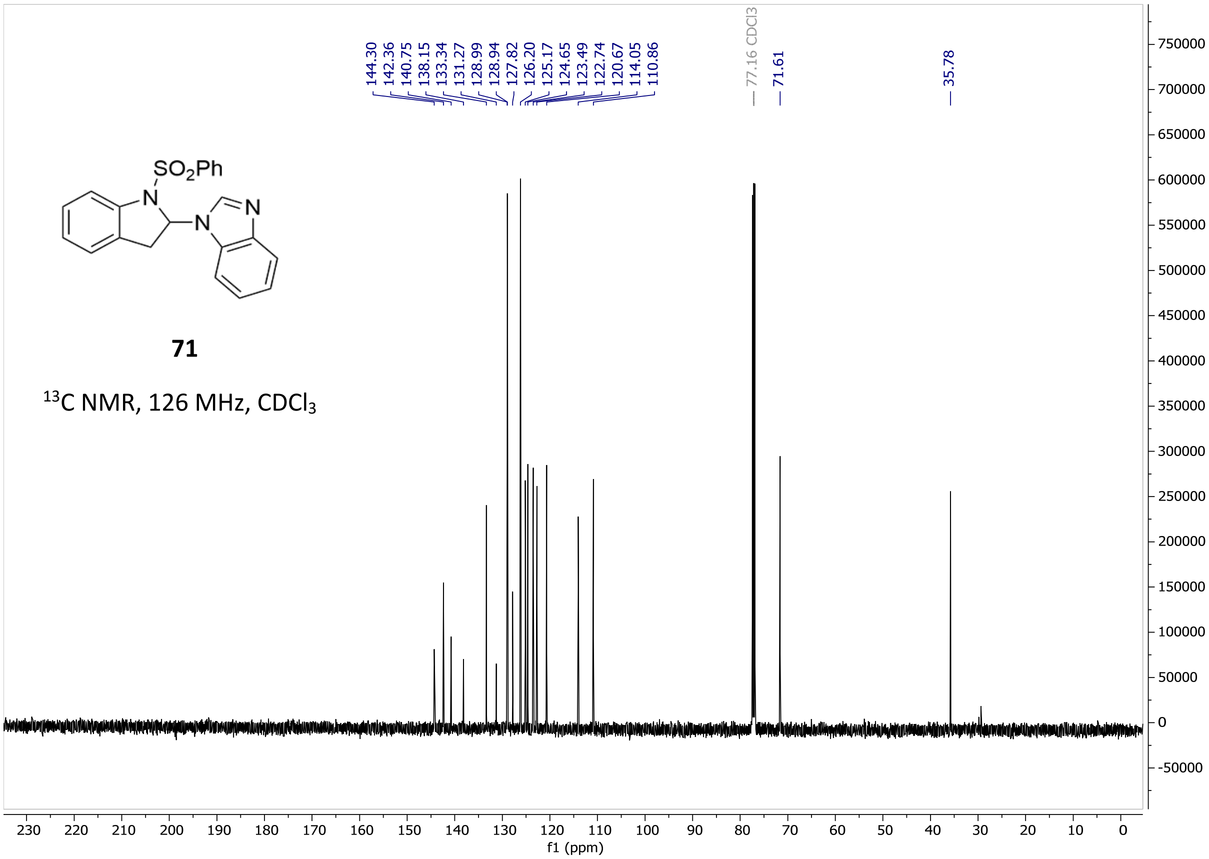
144.30
142.36
140.75
138.15
133.34
131.27
128.99
128.94
127.82
126.20
125.17
124.65
123.49
122.74
120.67
114.05
110.86
77.16 CDCl3
71.61
35.78
SO2Ph
N
N
N
71
13C NMR, 126 MHz, CDCl3
f1 (ppm)

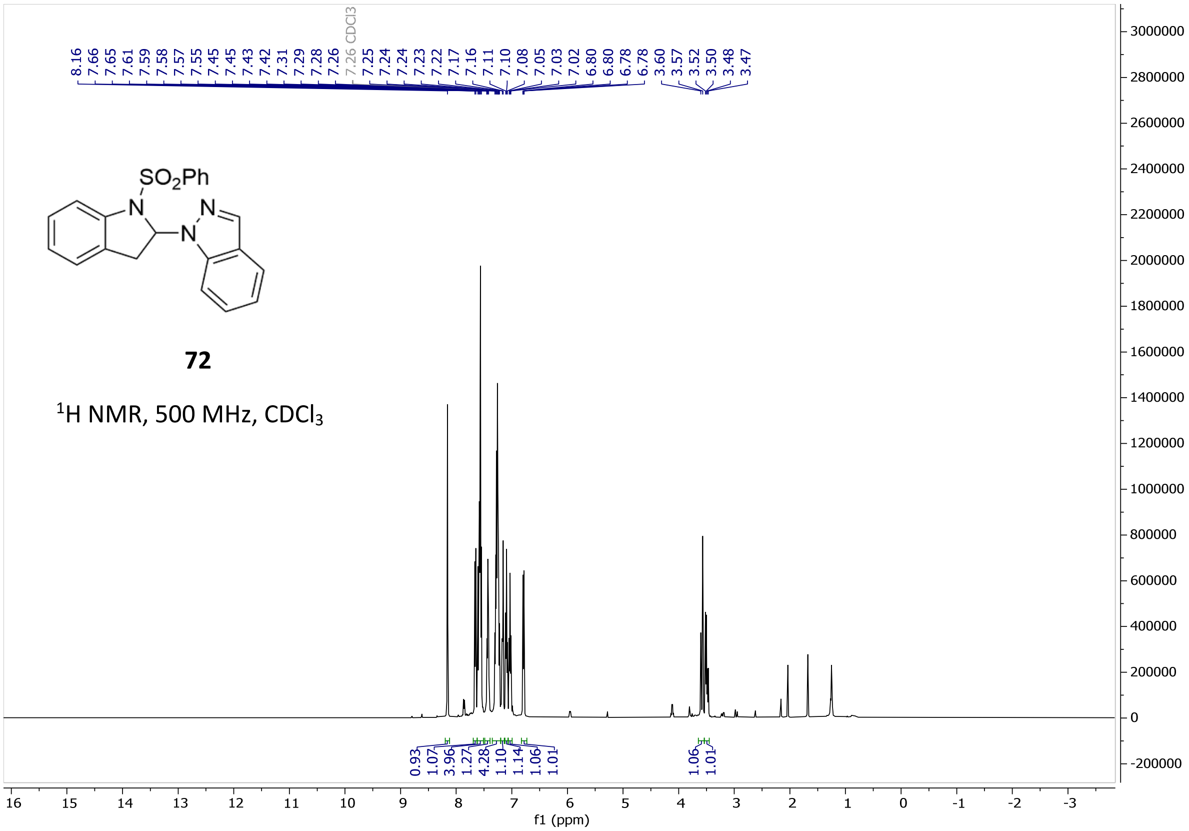
8.16 7.66 7.65 7.61 7.59 7.58 7.57 7.55 7.45 7.45 7.43 7.42 7.31 7.29 7.28 7.26 7.26 CDCl3 7.25 7.24 7.24 7.23 7.22 7.17 7.16 7.11 7.10 7.08 7.05 7.03 7.02 6.80 6.80 6.78 6.78 3.60 3.57 3.52 3.50 3.48 3.47
SO2Ph
N
N
N
72
1H NMR, 500 MHz, CDCl3
0.93 1.07 3.96 1.27 4.28 1.10 1.14 1.06 1.01
1.06 1.01
f1 (ppm)

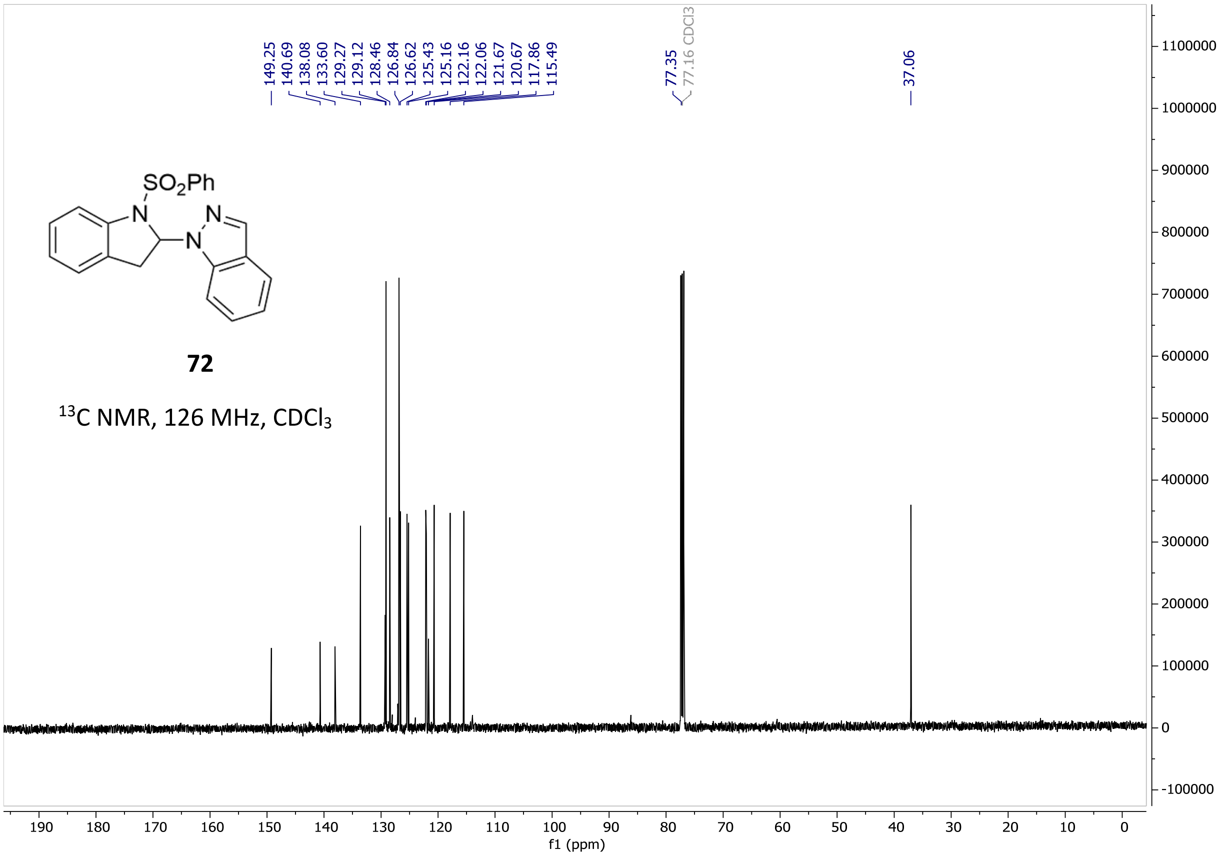
149.25 140.69 138.08 133.60 129.27 129.12 128.46 126.84 126.62 125.43 125.16 122.16 122.06 121.67 120.67 117.86 115.49
77.35 77.16 CDCl3
37.06
SO2Ph
N
N
N
72
13C NMR, 126 MHz, CDCl3
f1 (ppm)

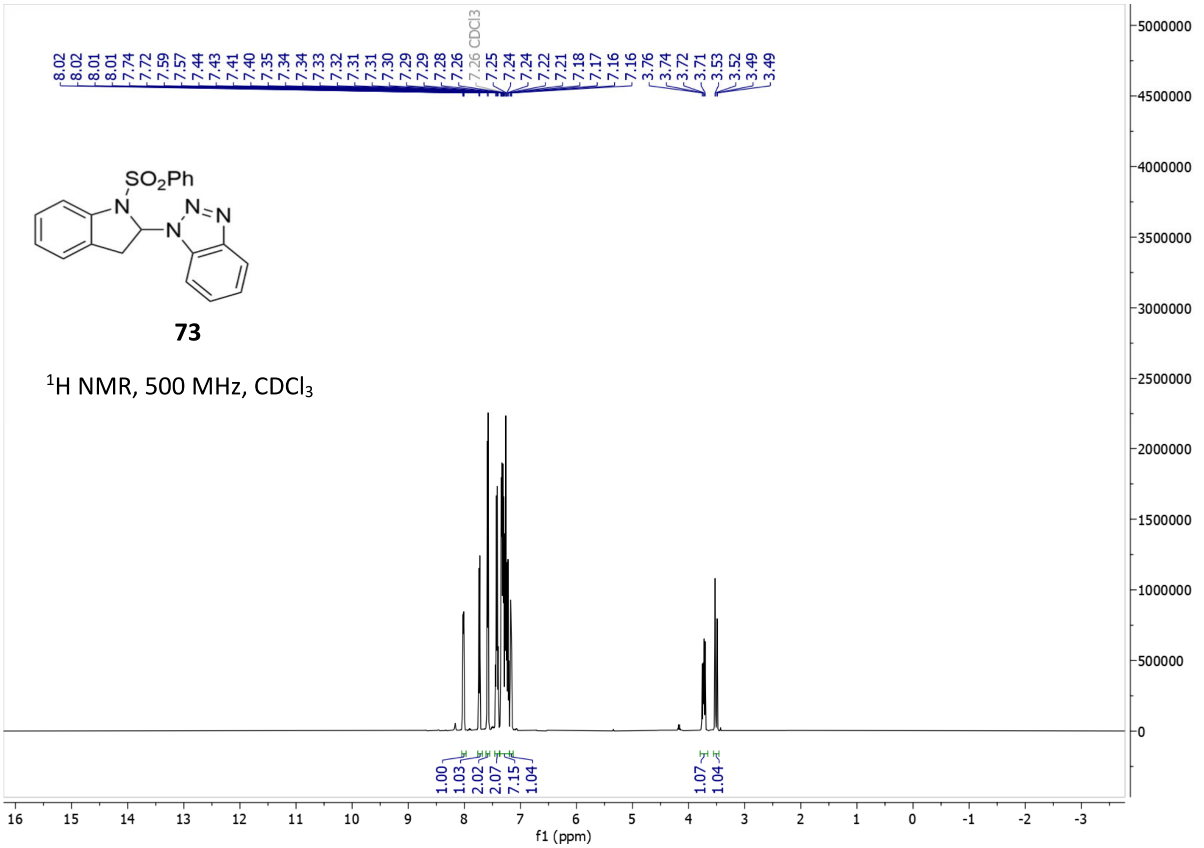

8.02
8.02
8.01
8.01
7.74
7.72
7.59
7.57
7.44
7.43
7.41
7.40
7.35
7.34
7.34
7.33
7.32
7.31
7.31
7.30
7.29
7.29
7.28
7.26
7.26 CDCl3
7.25
7.24
7.24
7.22
7.21
7.18
7.17
7.16
7.16
3.76
3.74
3.72
3.71
3.53
3.52
3.49
3.49
SO2Ph
N
N
N
N
73
1H NMR, 500 MHz, CDCl3
1.00
1.03
2.02
2.07
7.15
1.04
1.07
1.04
f1 (ppm)

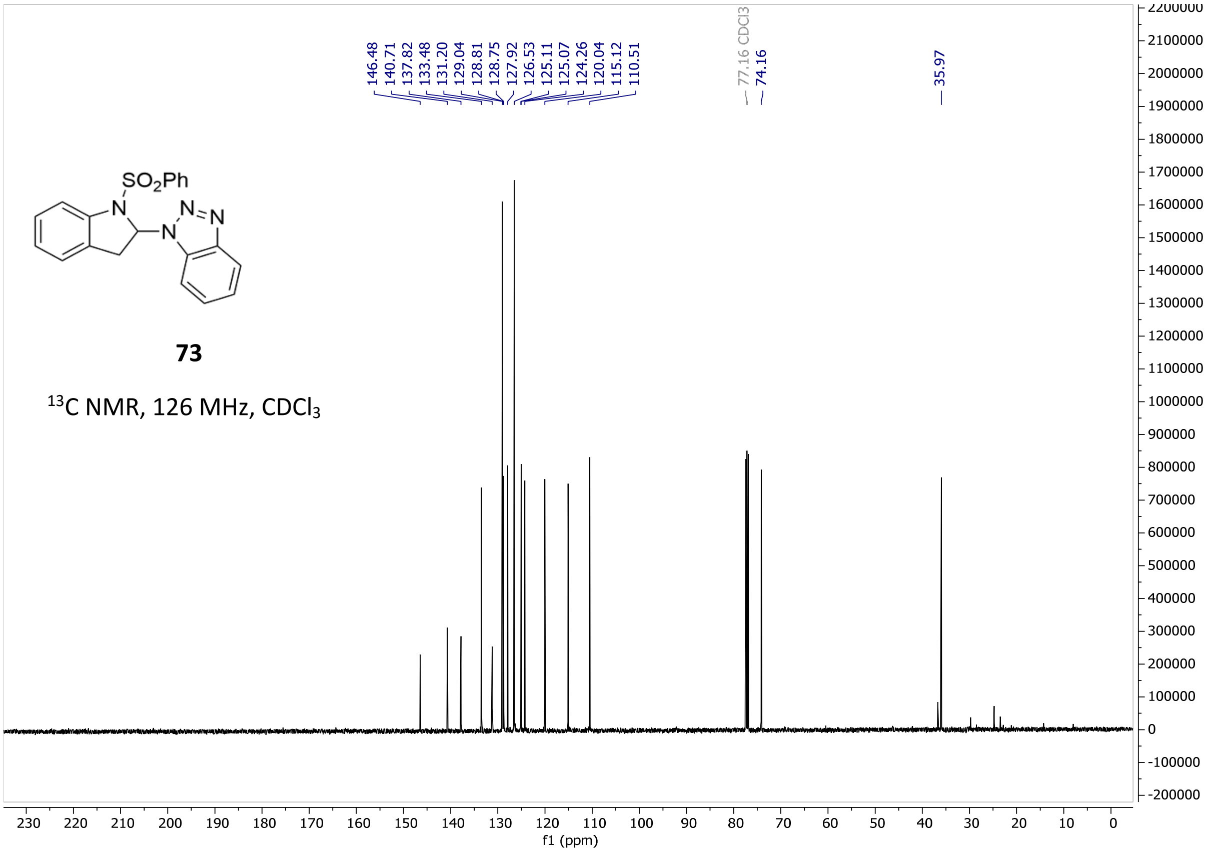

146.48
140.71
137.82
133.48
131.20
129.04
128.81
128.75
127.92
126.53
125.11
125.07
124.26
120.04
115.12
110.51
77.16 CDCl3
74.16
35.97
SO2Ph
N
N
N
N
73
13C NMR, 126 MHz, CDCl3
f1 (ppm)

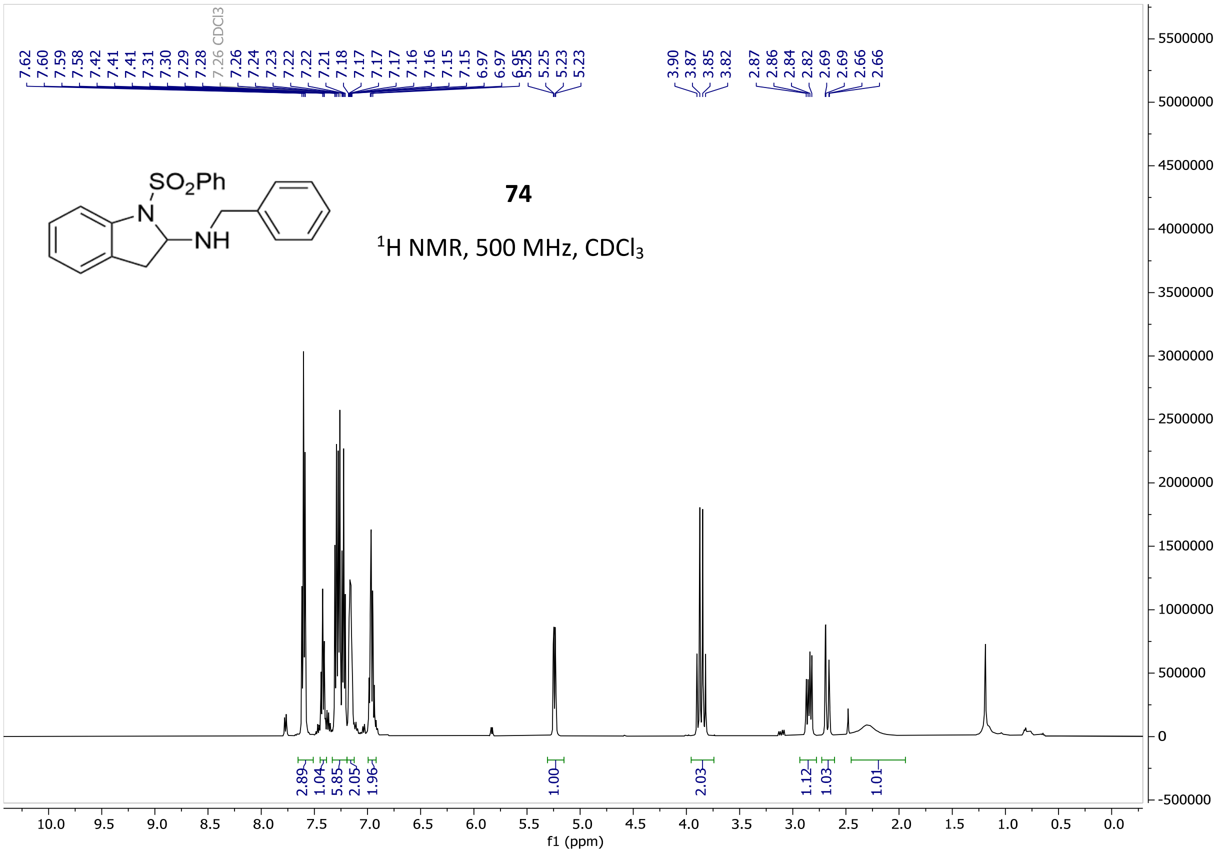

7.62
7.60
7.59
7.58
7.42
7.41
7.41
7.31
7.30
7.29
7.28
7.26 CDCl3
7.26
7.24
7.23
7.22
7.22
7.21
7.18
7.17
7.17
7.17
7.16
7.15
7.15
6.97
6.97
6.95
5.25
5.23
5.23
3.90
3.87
3.85
3.82
2.87
2.86
2.84
2.82
2.69
2.69
2.66
2.66
SO2Ph
N
NH
74
1H NMR, 500 MHz, CDCl3
2.89
1.04
5.85
2.05
1.96
1.00
2.03
1.12
1.03
1.01
f1 (ppm)

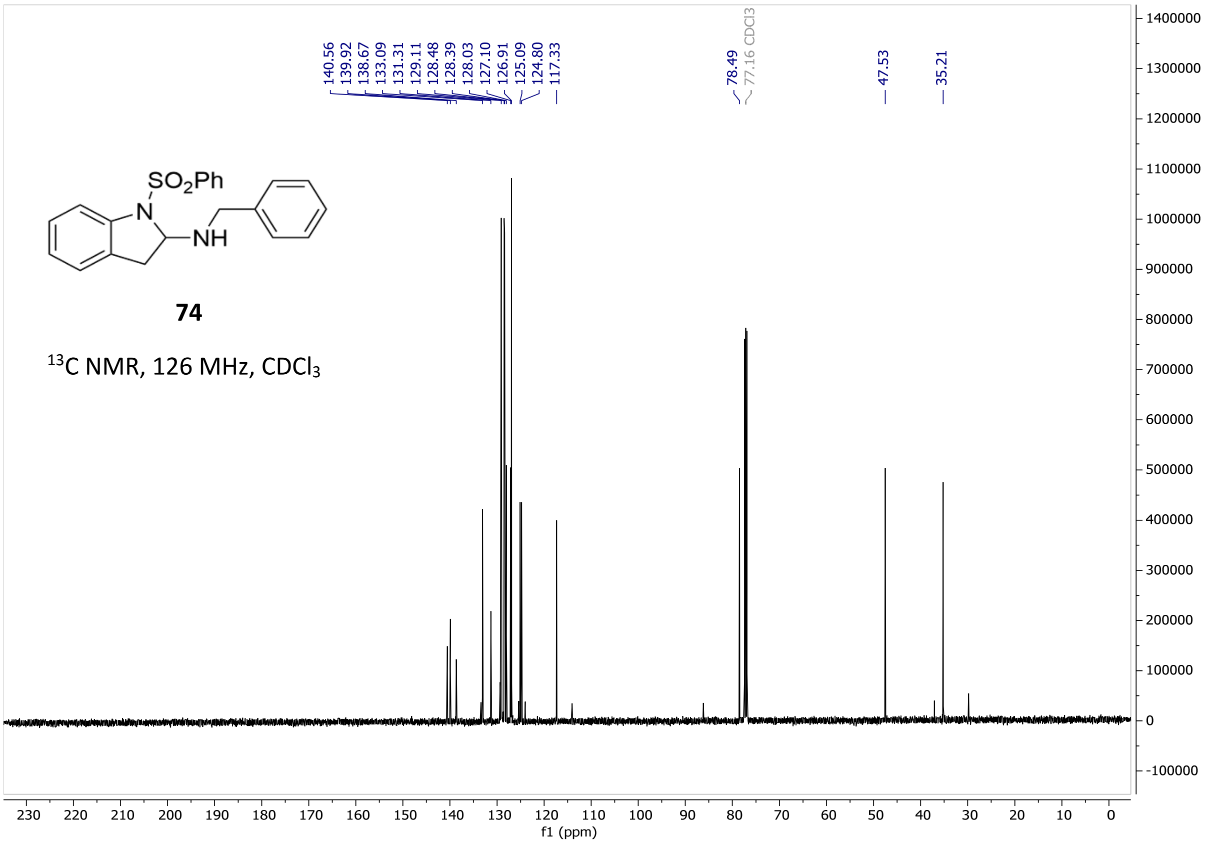

140.56
139.92
138.67
133.09
131.31
129.11
128.48
128.39
128.03
127.10
126.91
125.09
124.80
117.33
78.49
77.16 CDCl3
47.53
35.21
SO2Ph
N
NH
74
13C NMR, 126 MHz, CDCl3
f1 (ppm)

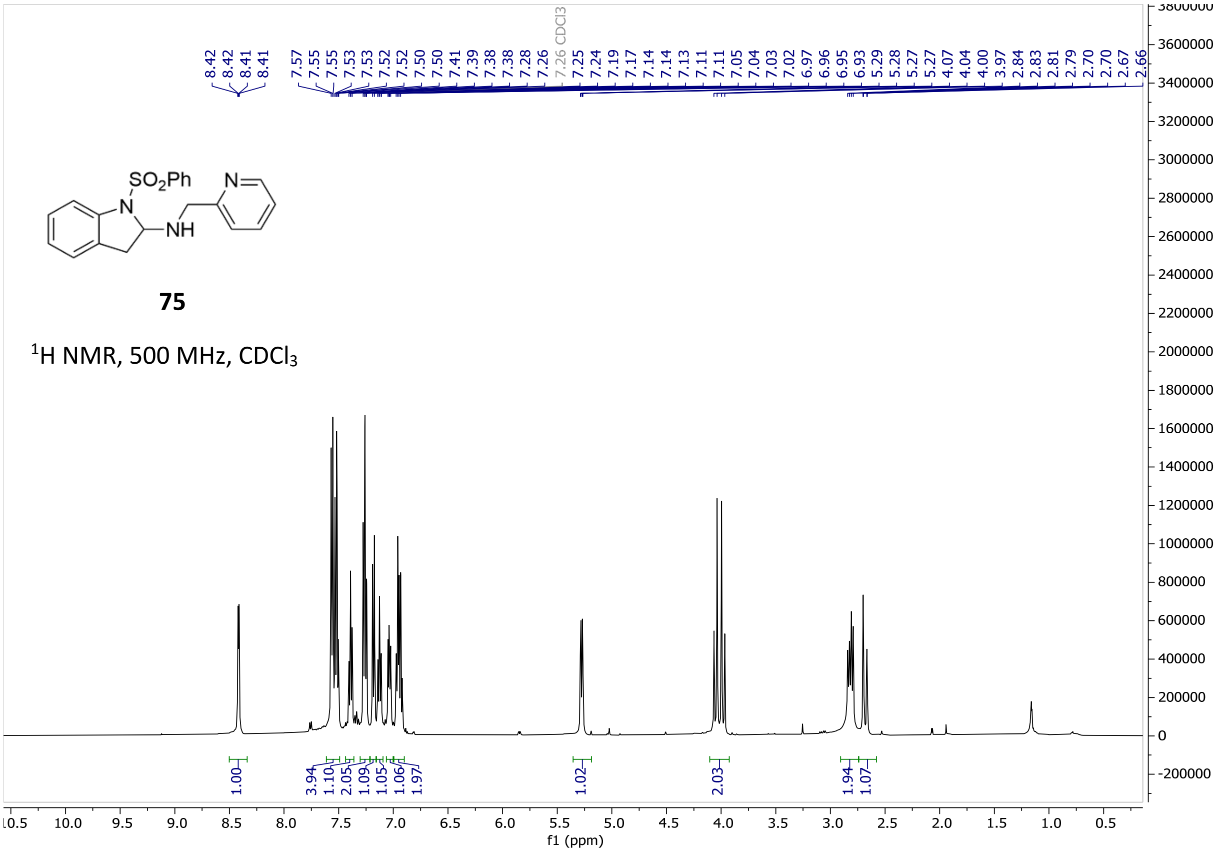
75
^{1}H NMR, 500 MHz, CDCl$_3$
f1 (ppm)

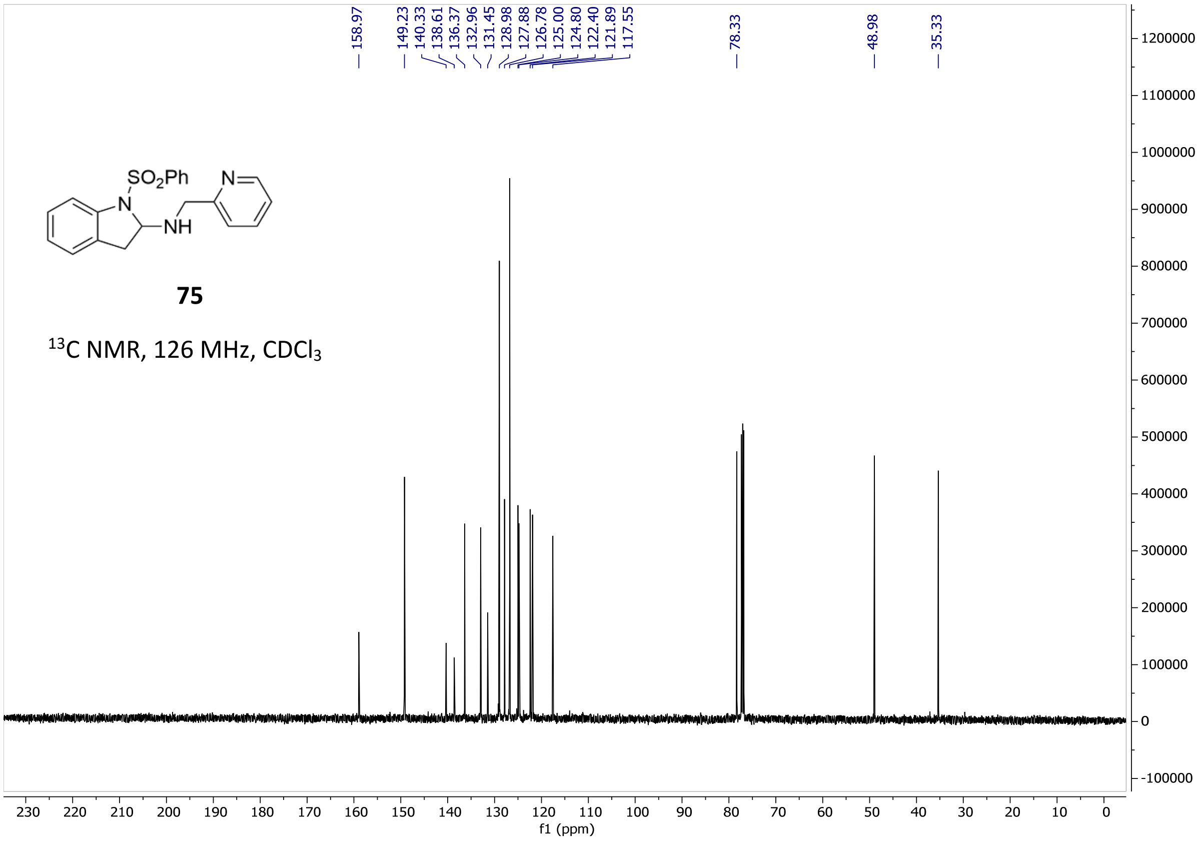
75
^{13}C NMR, 126 MHz, CDCl$_3$
f1 (ppm)

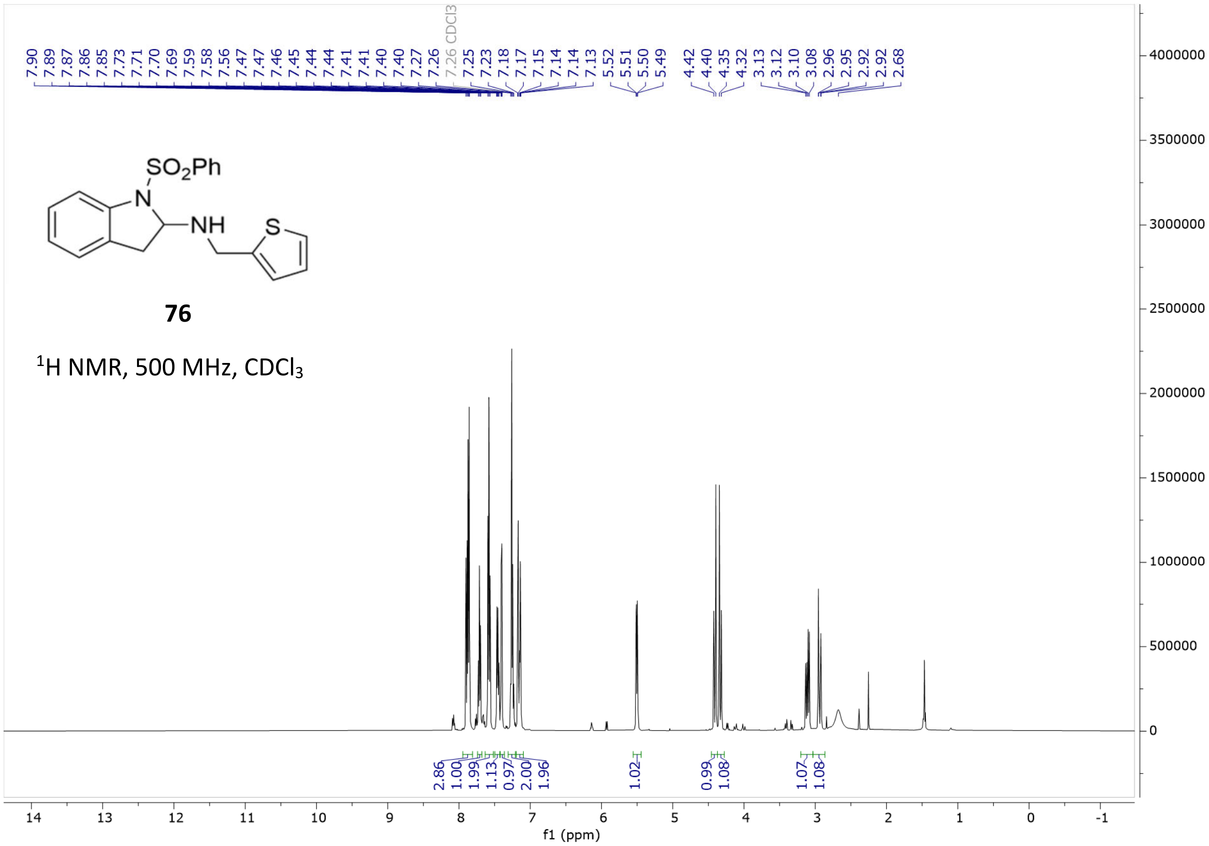
7.90
7.89
7.87
7.86
7.85
7.73
7.71
7.70
7.69
7.59
7.58
7.56
7.47
7.47
7.46
7.45
7.44
7.44
7.41
7.41
7.40
7.40
7.27
7.26
7.26 CDCl3
7.25
7.23
7.18
7.17
7.15
7.14
7.14
7.13
5.52
5.51
5.50
5.49
4.42
4.40
4.35
4.32
3.13
3.12
3.10
3.08
2.96
2.95
2.92
2.92
2.68
SO2Ph
N
NH
S
76
1H NMR, 500 MHz, CDCl3
2.86
1.00
1.99
1.13
0.97
2.00
1.96
1.02
0.99
1.08
1.07
1.08
f1 (ppm)

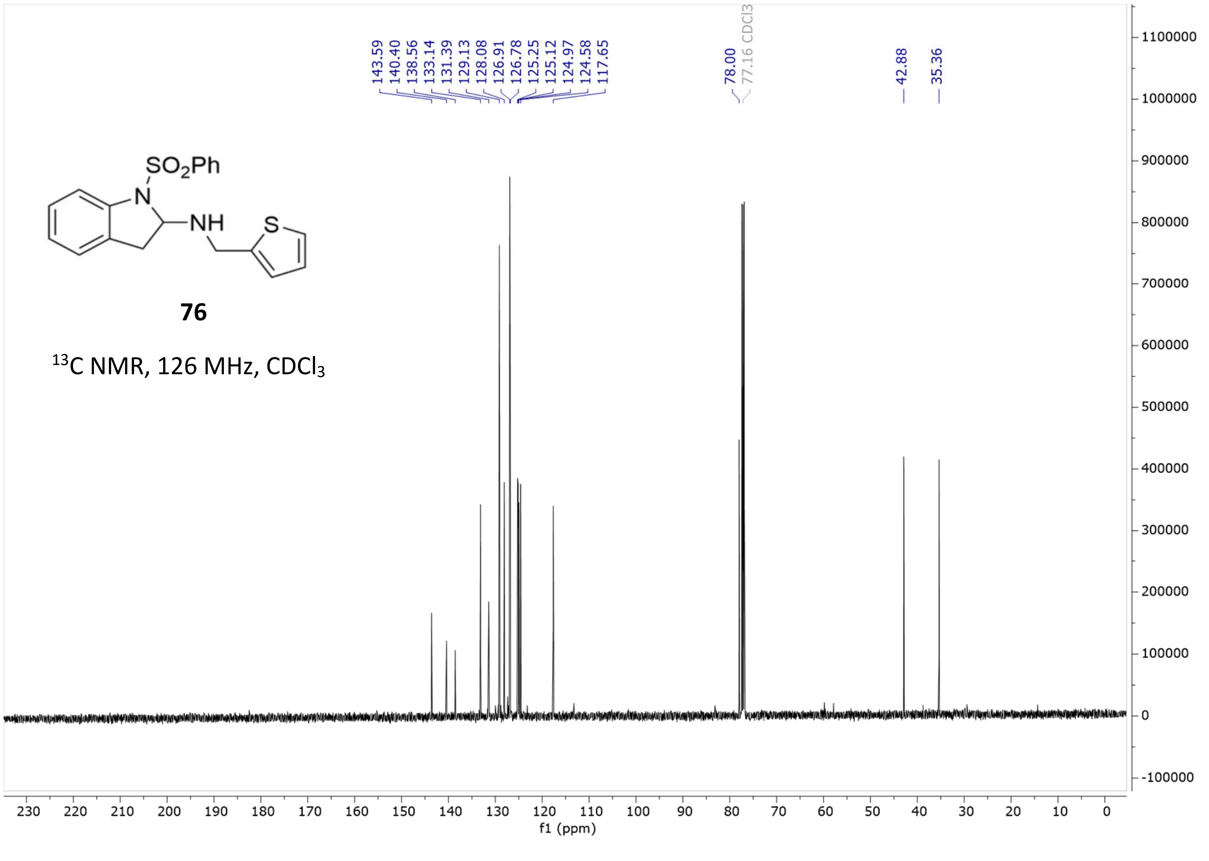
143.59
140.40
138.56
133.14
131.39
129.13
128.08
126.91
126.78
125.25
125.12
124.97
124.58
117.65
78.00
77.16 CDCl3
42.88
35.36
SO2Ph
N
NH
S
76
13C NMR, 126 MHz, CDCl3
f1 (ppm)

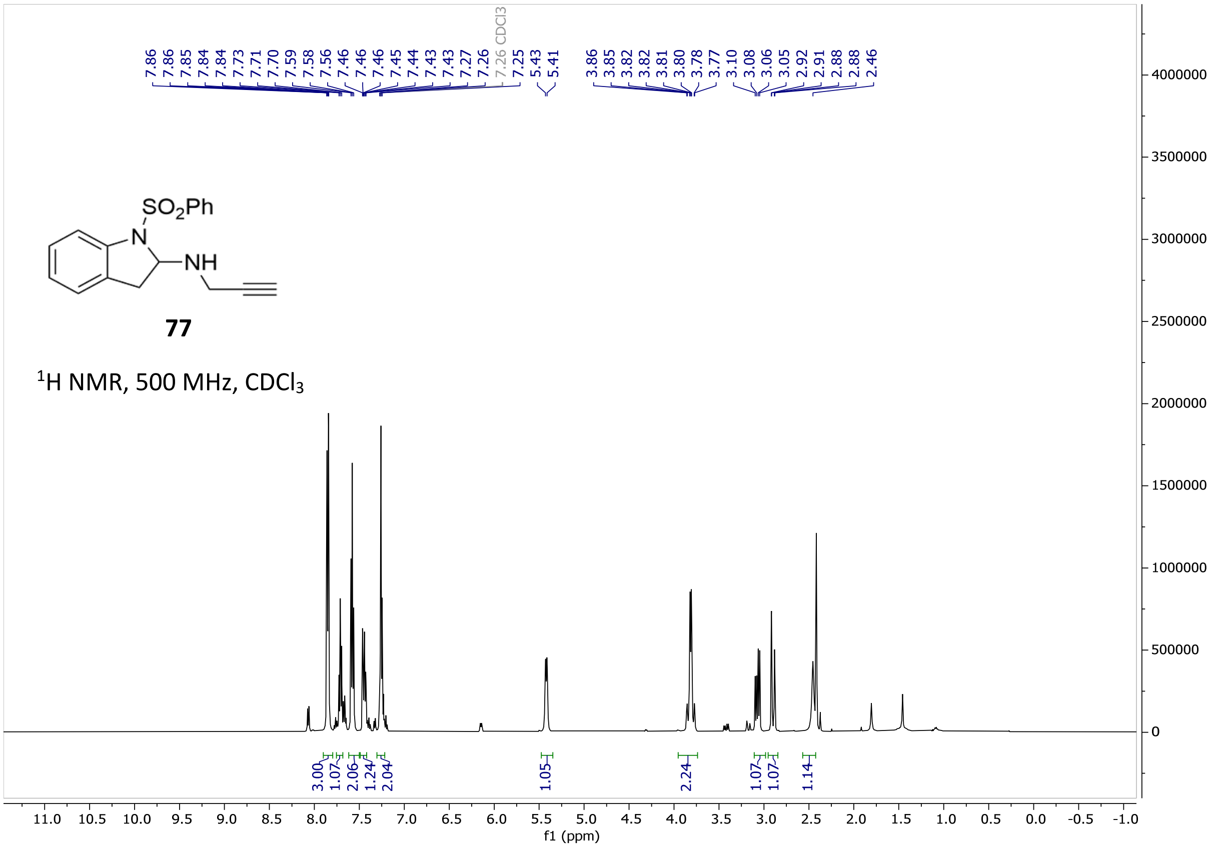
7.86 7.86 7.85 7.84 7.84 7.73 7.71 7.70 7.59 7.58 7.56 7.46 7.46 7.45 7.44 7.43 7.43 7.27 7.26 7.26 CDCl3 7.25
5.43 5.41
3.86 3.85 3.82 3.82 3.81 3.80 3.78 3.77 3.10 3.08 3.06 3.05 2.92 2.91 2.88 2.46
SO2Ph
N
NH
77
1H NMR, 500 MHz, CDCl3
3.00 1.07 2.06 1.24 2.04 1.05 2.24 1.07 1.07 1.14
f1 (ppm)

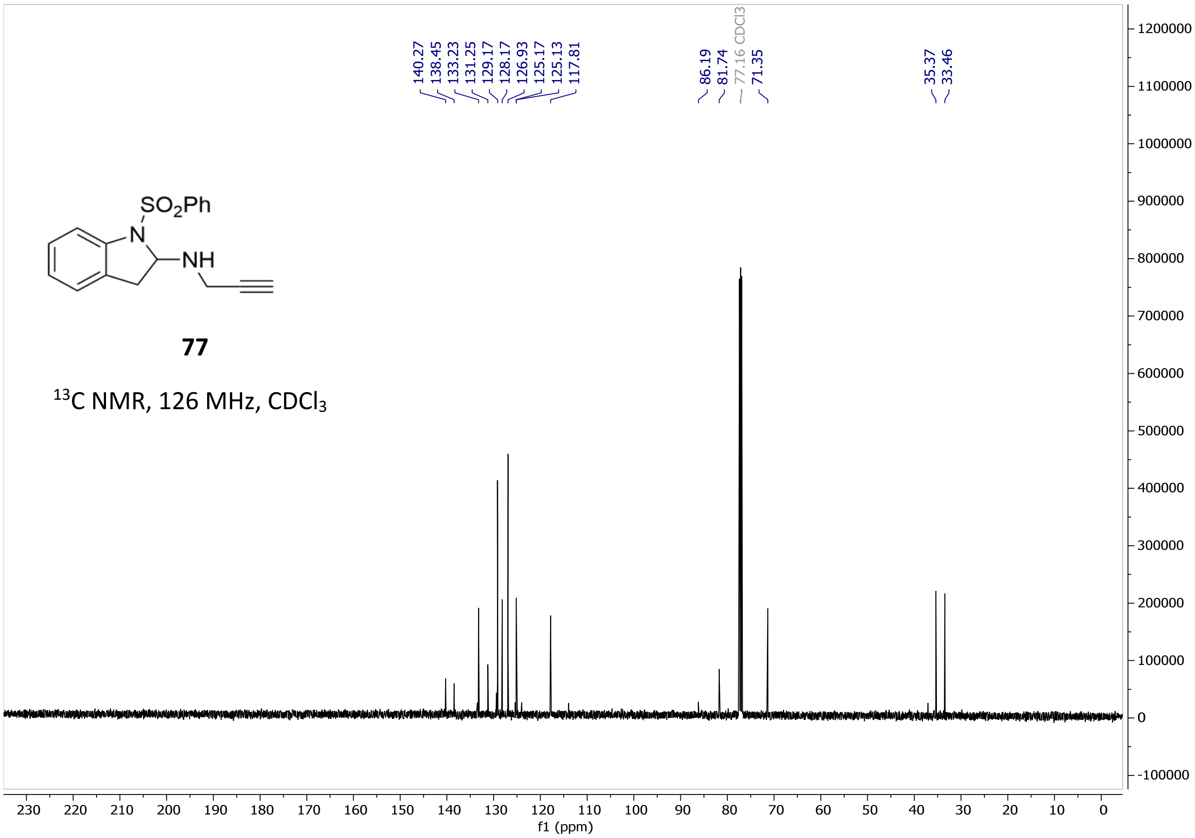
140.27 138.45 133.23 131.25 129.17 128.17 126.93 125.17 125.13 117.81
86.19 81.74 77.16 CDCl3 71.35
35.37 33.46
SO2Ph
N
NH
77
13C NMR, 126 MHz, CDCl3
f1 (ppm)

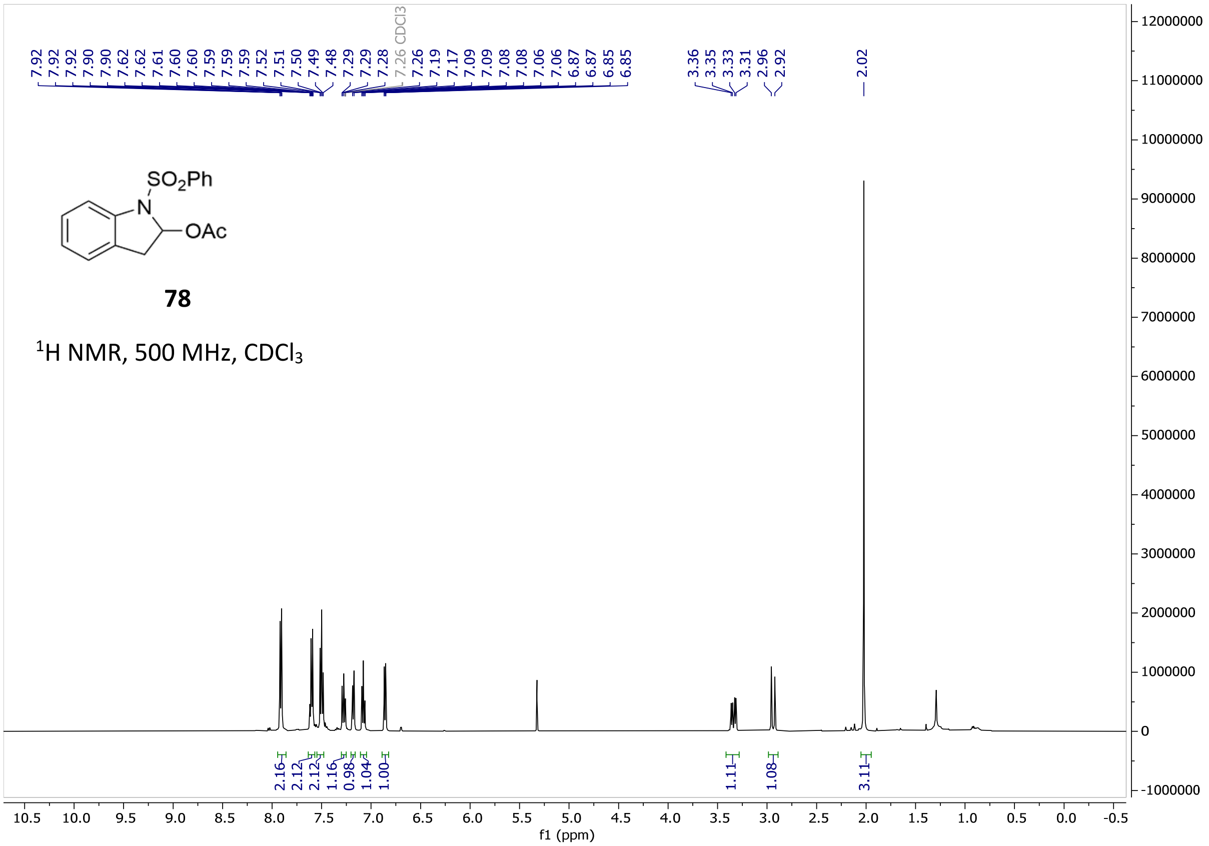
7.92
7.92
7.90
7.90
7.62
7.62
7.61
7.60
7.60
7.59
7.59
7.59
7.52
7.51
7.50
7.49
7.48
7.29
7.29
7.28
7.26 CDCl3
7.26
7.19
7.17
7.09
7.09
7.08
7.08
7.06
7.06
6.87
6.85
6.85
3.36
3.35
3.33
3.31
2.96
2.92
2.02
SO_2Ph
N
OAc
78
^1H NMR, 500 MHz, $CDCl_3$
2.16
2.12
2.12
1.16
0.98
1.04
1.00
1.11
1.08
3.11
f1 (ppm)

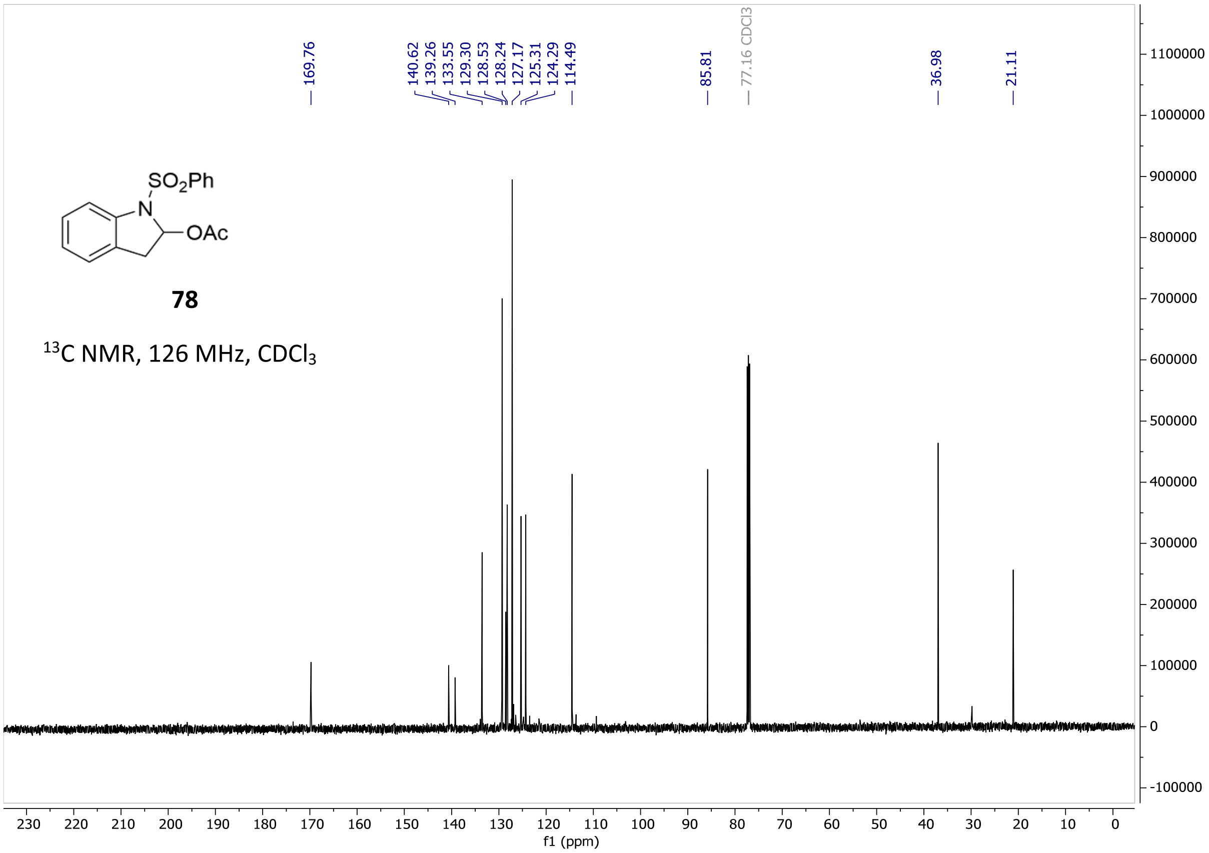
169.76
140.62
139.26
133.55
129.30
128.53
128.24
127.17
125.31
124.29
114.49
85.81
77.16 CDCl3
36.98
21.11
SO_2Ph
N
OAc
78
^{13}C NMR, 126 MHz, $CDCl_3$
f1 (ppm)

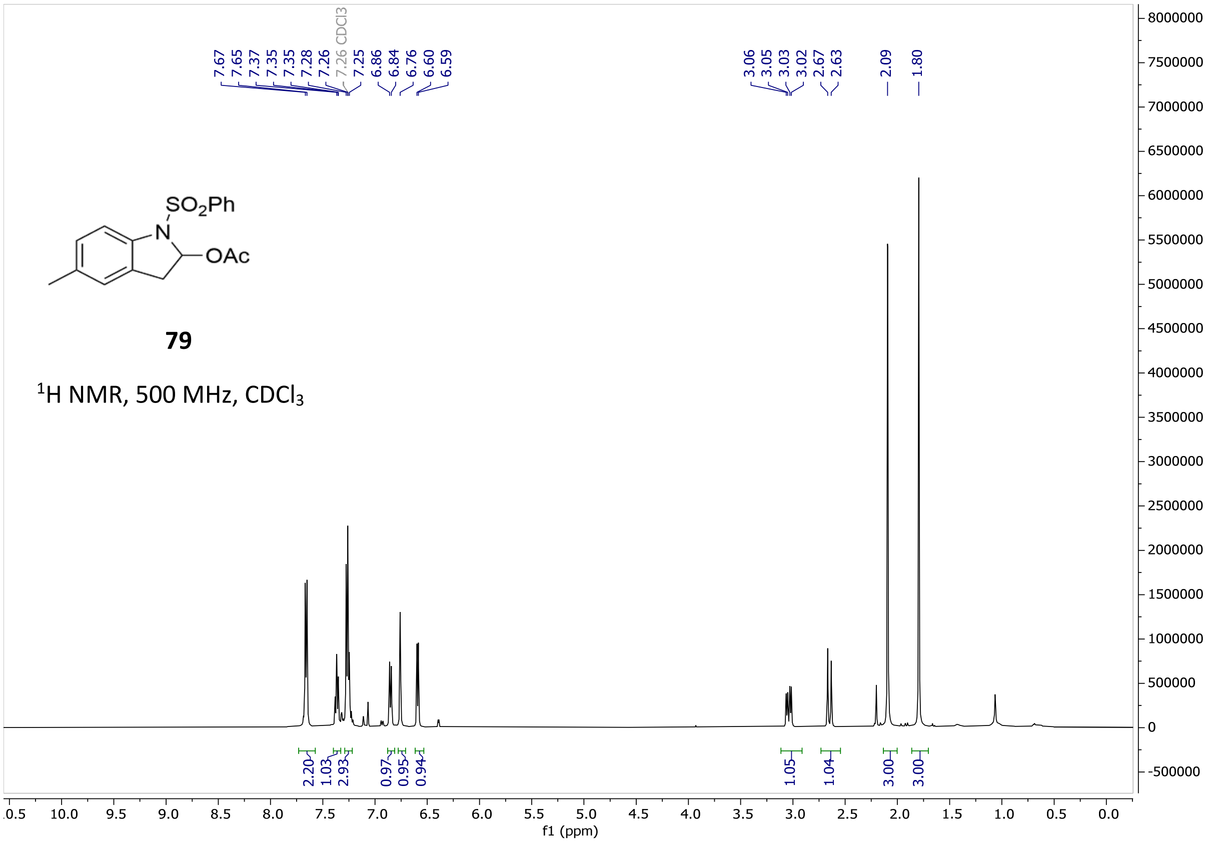
7.67
7.65
7.37
7.35
7.35
7.28
7.26
7.26 CDCl3
7.25
6.86
6.84
6.76
6.60
6.59
3.06
3.05
3.03
3.02
2.67
2.63
2.09
1.80
SO2Ph
N
OAc
79
1H NMR, 500 MHz, CDCl3
2.20
1.03
2.93
0.97
0.95
0.94
1.05
1.04
3.00
3.00
f1 (ppm)

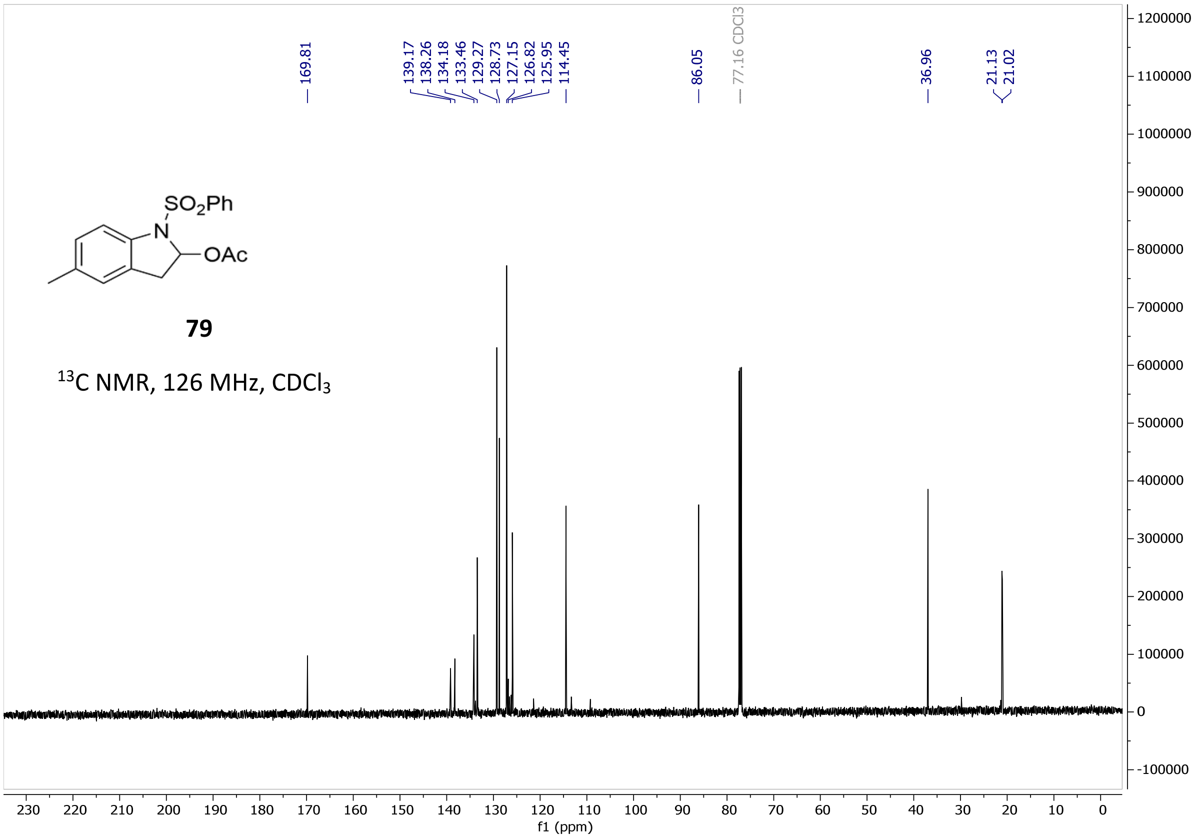
169.81
139.17
138.26
134.18
133.46
129.27
128.73
127.15
126.82
125.95
114.45
86.05
77.16 CDCl3
36.96
21.13
21.02
SO2Ph
N
OAc
79
13C NMR, 126 MHz, CDCl3
f1 (ppm)

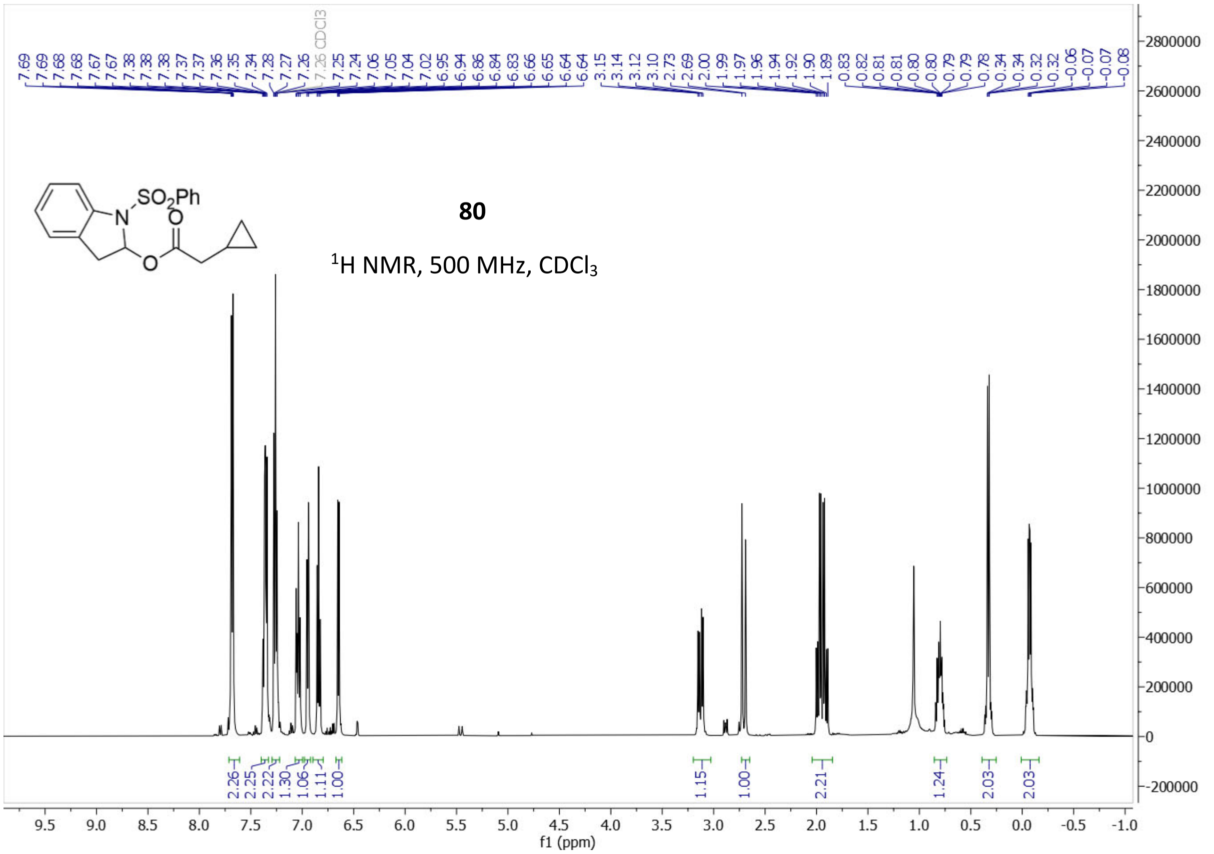
7.69 7.69 7.68 7.68 7.67 7.67 7.38 7.38 7.38 7.37 7.37 7.36 7.35 7.34 7.28 7.27 7.26 CDCl3 7.25 7.24 7.06 7.05 7.04 7.02 6.95 6.94 6.86 6.84 6.83 6.66 6.65 6.64 6.64 3.15 3.14 3.12 3.10 2.73 2.69 2.00 1.99 1.97 1.96 1.94 1.92 1.90 1.89 0.83 0.82 0.81 0.81 0.80 0.80 0.79 0.79 0.78 0.34 0.34 0.32 0.32 -0.06 -0.07 -0.07 -0.08
SO2Ph
80
1H NMR, 500 MHz, CDCl3
2.26 2.25 2.22 1.30 1.06 1.11 1.00 1.15 1.00 2.21 1.24 2.03 2.03
f1 (ppm)

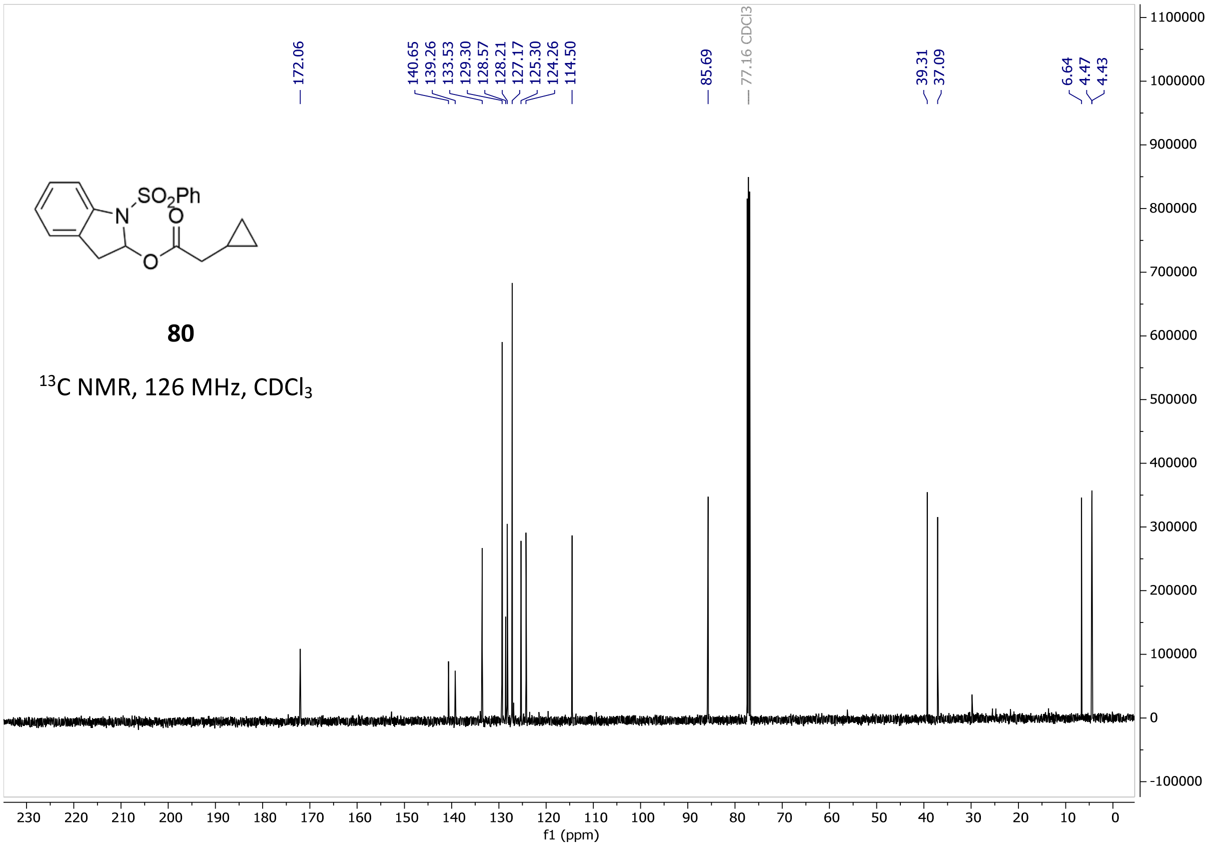
172.06 140.65 139.26 133.53 129.30 128.57 128.21 127.17 125.30 124.26 114.50 85.69 77.16 CDCl3 39.31 37.09 6.64 4.47 4.43
SO2Ph
80
13C NMR, 126 MHz, CDCl3
f1 (ppm)

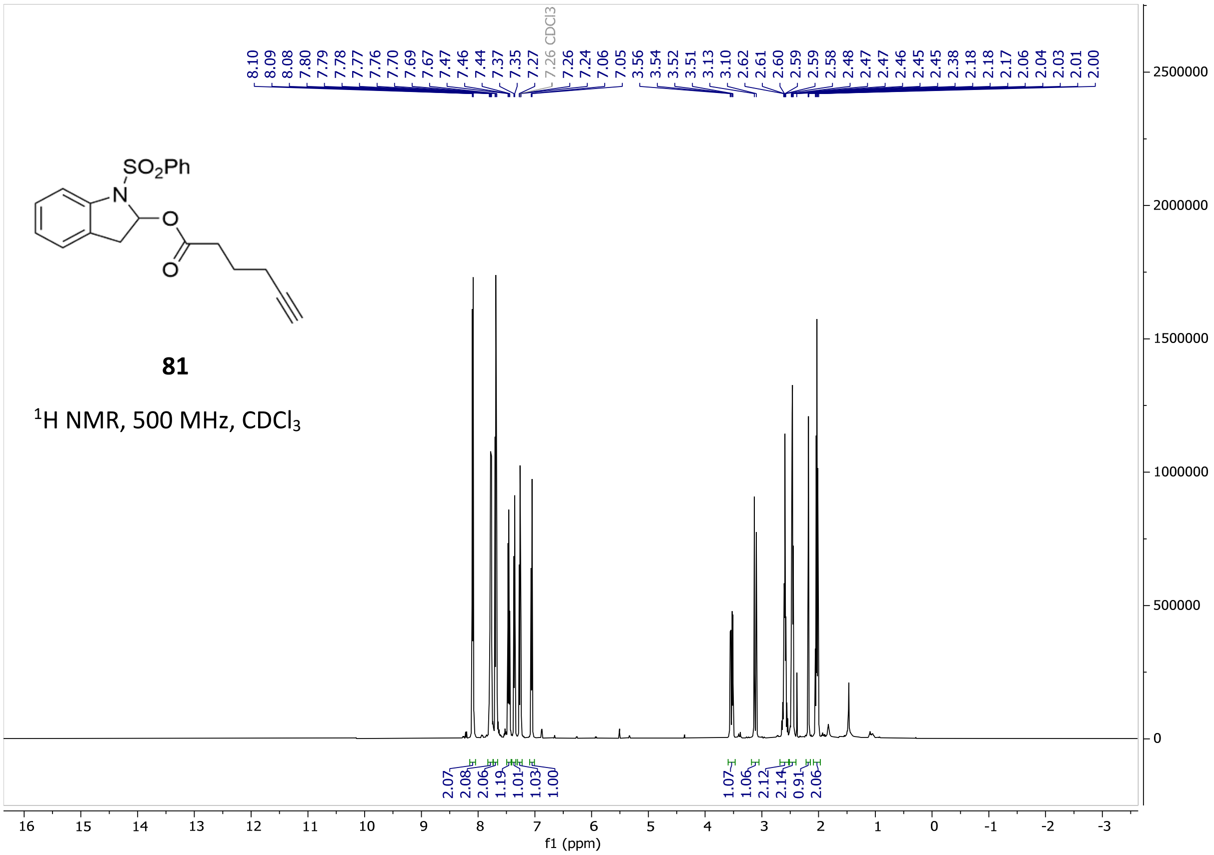
SO2Ph
N
O
O
81
1H NMR, 500 MHz, CDCl3
8.10 8.09 8.08 7.80 7.79 7.78 7.77 7.76 7.70 7.69 7.67 7.47 7.46 7.44 7.37 7.35 7.27 7.26 CDCl3 7.26 7.24 7.06 7.05 3.56 3.54 3.52 3.51 3.13 3.10 2.62 2.61 2.60 2.59 2.59 2.58 2.48 2.47 2.47 2.46 2.45 2.45 2.38 2.18 2.18 2.17 2.06 2.04 2.03 2.01 2.00
2.07 2.08 2.06 1.19 1.01 1.03 1.00 1.07 1.06 2.12 2.14 0.91 2.06
f1 (ppm)

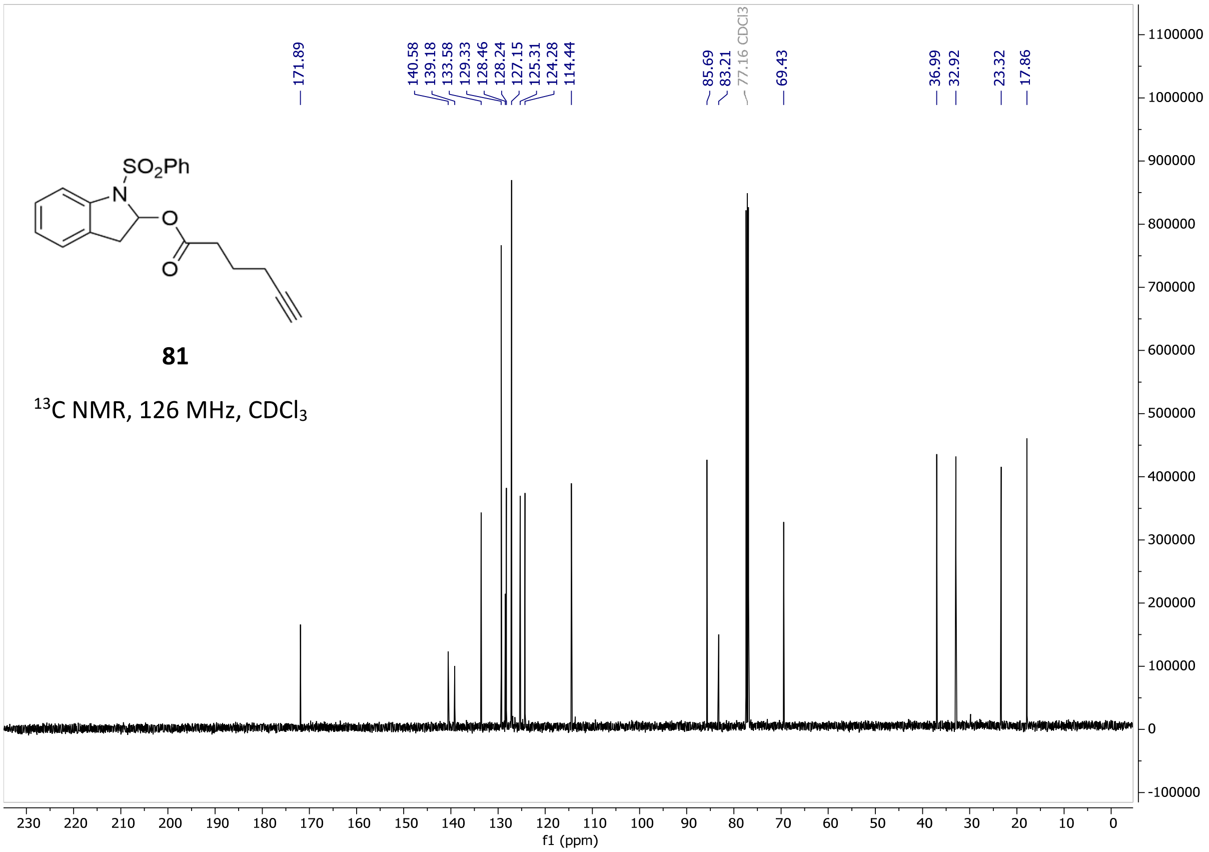
SO2Ph
N
O
O
81
13C NMR, 126 MHz, CDCl3
171.89 140.58 139.18 133.58 129.33 128.46 128.24 127.15 125.31 124.28 114.44 85.69 83.21 77.16 CDCl3 69.43 36.99 32.92 23.32 17.86
f1 (ppm)

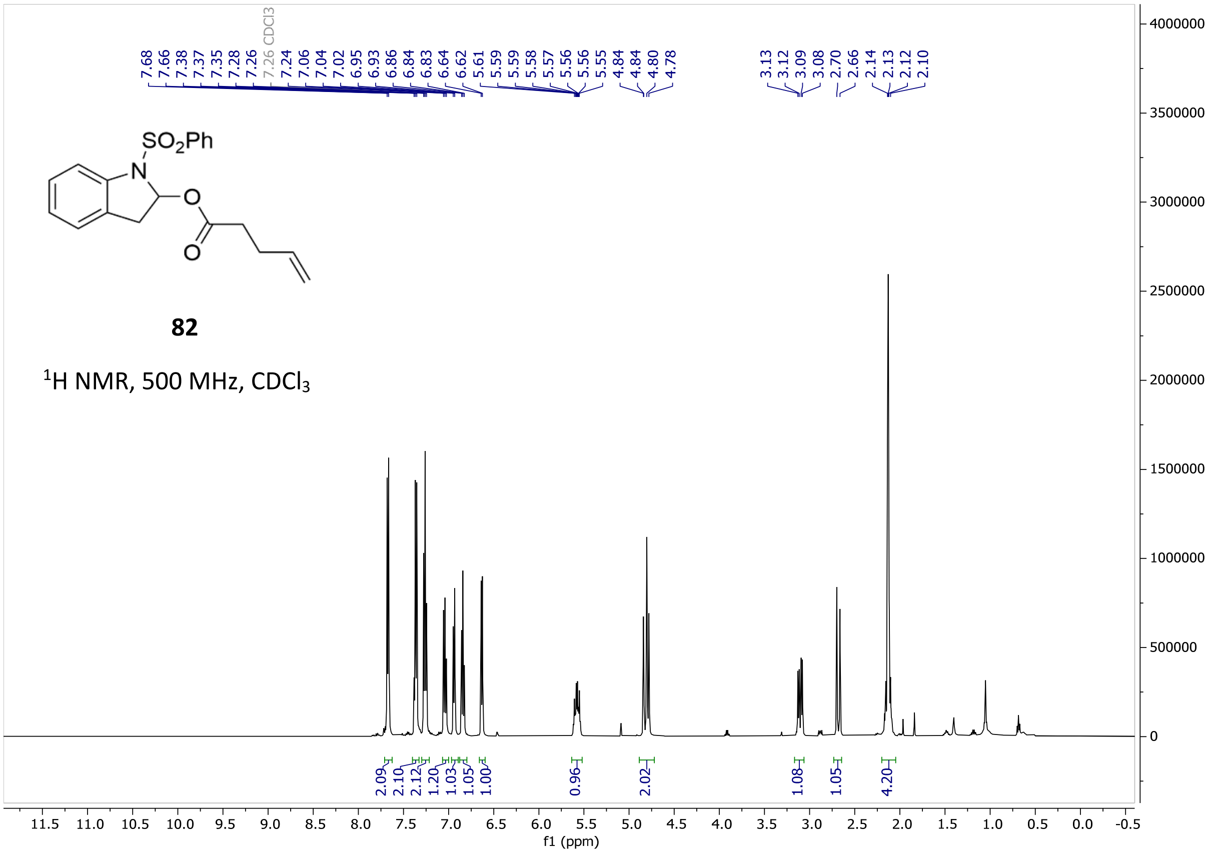
SO2Ph
N
O
O
82
1H NMR, 500 MHz, CDCl3
f1 (ppm)

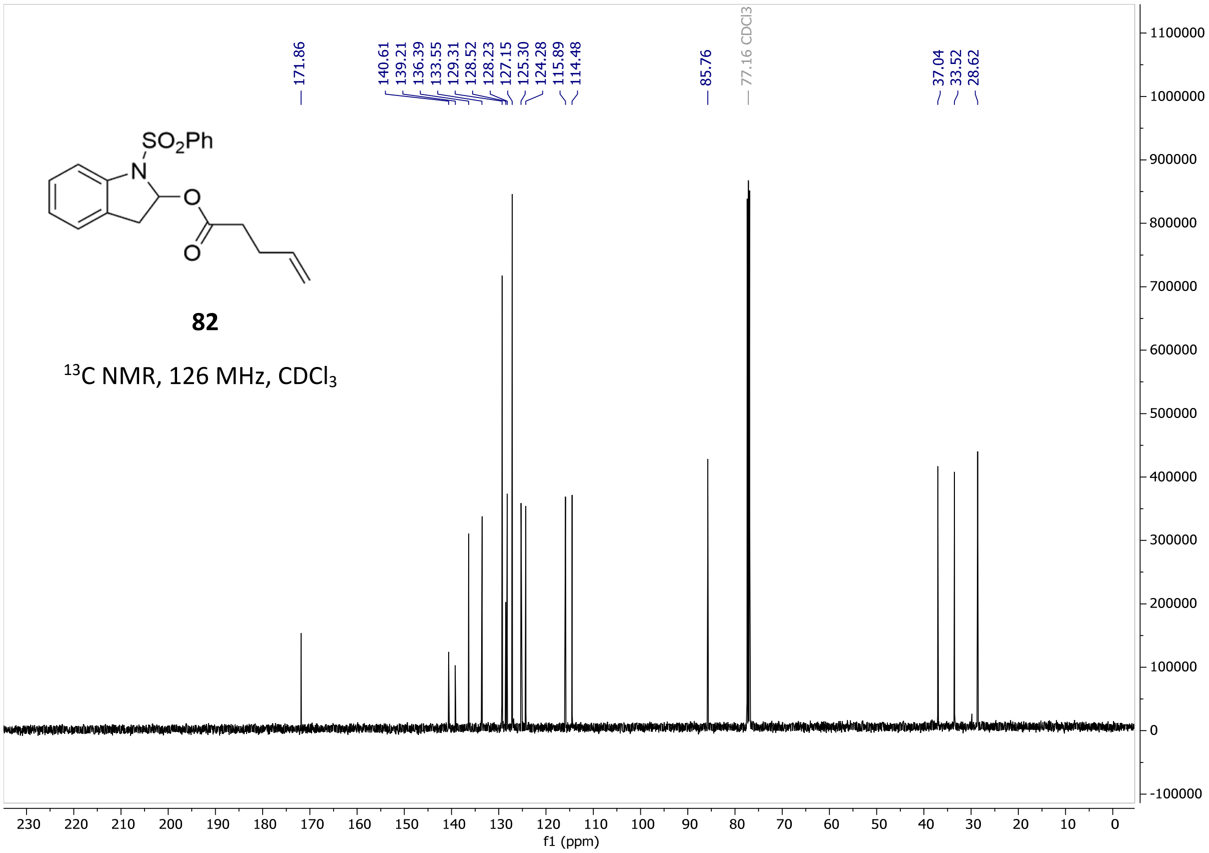
SO2Ph
N
O
O
82
13C NMR, 126 MHz, CDCl3
171.86
140.61
139.21
136.39
133.55
129.31
128.52
128.23
127.15
125.30
124.28
115.89
114.48
85.76
77.16 CDCl3
37.04
33.52
28.62
f1 (ppm)

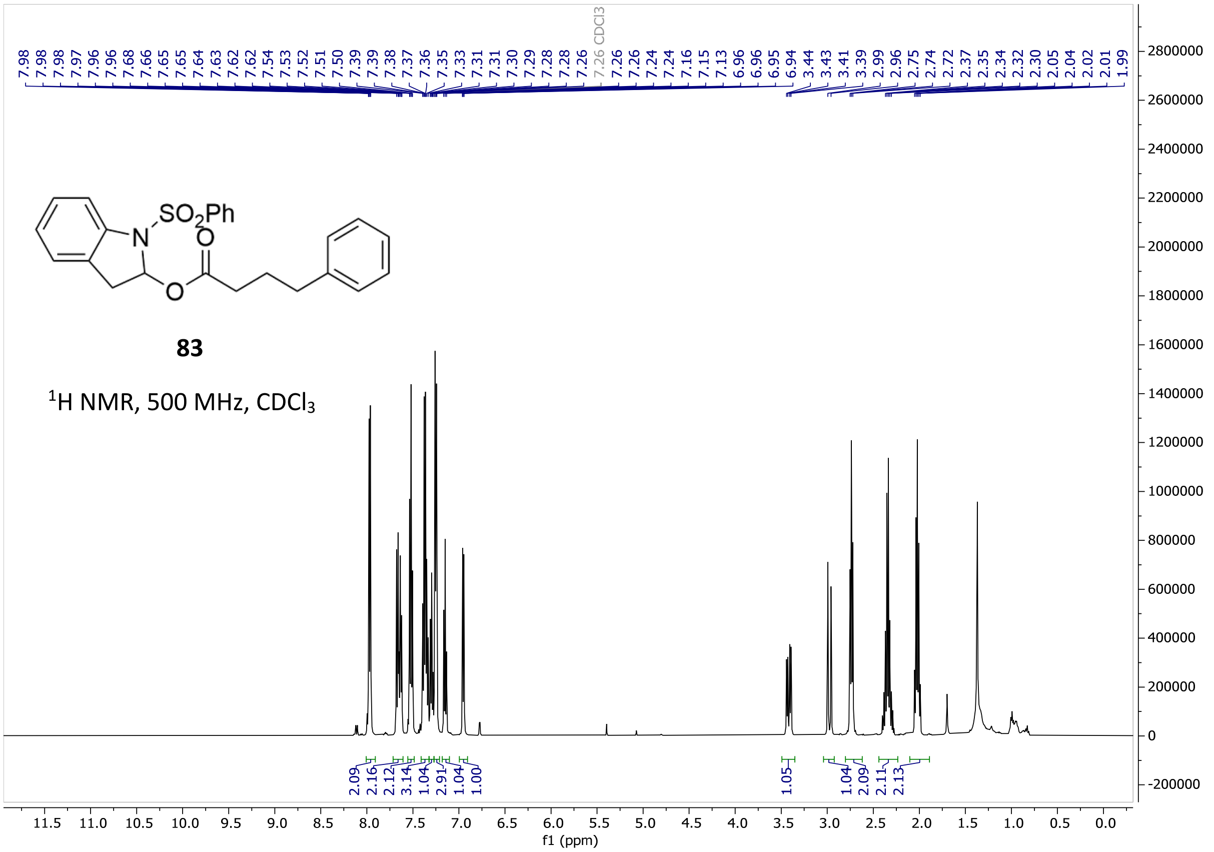
7.98 7.98 7.98 7.97 7.96 7.96 7.68 7.66 7.65 7.65 7.64 7.63 7.62 7.62 7.54 7.53 7.52 7.51 7.50 7.39 7.39 7.38 7.37 7.36 7.35 7.33 7.31 7.31 7.30 7.29 7.28 7.28 7.26 7.26 CDCl3 7.26 7.26 7.24 7.24 7.16 7.15 7.13 6.96 6.96 6.95 6.94 3.44 3.43 3.41 3.39 2.99 2.96 2.75 2.74 2.72 2.37 2.35 2.34 2.32 2.30 2.05 2.04 2.02 2.01 1.99
SO2Ph
N
O
O
83
1H NMR, 500 MHz, CDCl3
2.09 2.16 2.12 3.14 1.04 2.91 1.04 1.00
1.05 1.04 2.09 2.11 2.13
f1 (ppm)

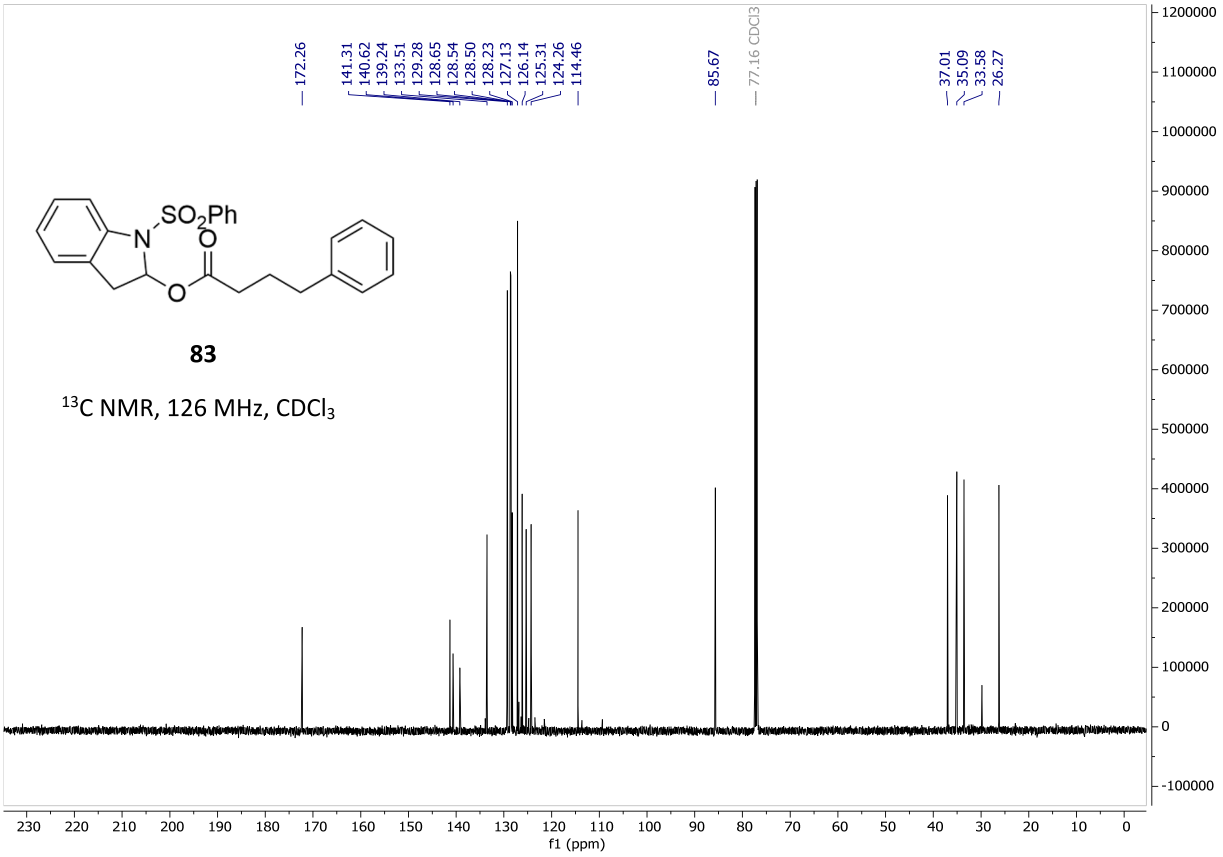
172.26
141.31 140.62 139.24 133.51 129.28 128.65 128.54 128.50 128.23 127.13 126.14 125.31 124.26 114.46
85.67
77.16 CDCl3
37.01 35.09 33.58 26.27
SO2Ph
N
O
O
83
13C NMR, 126 MHz, CDCl3
f1 (ppm)

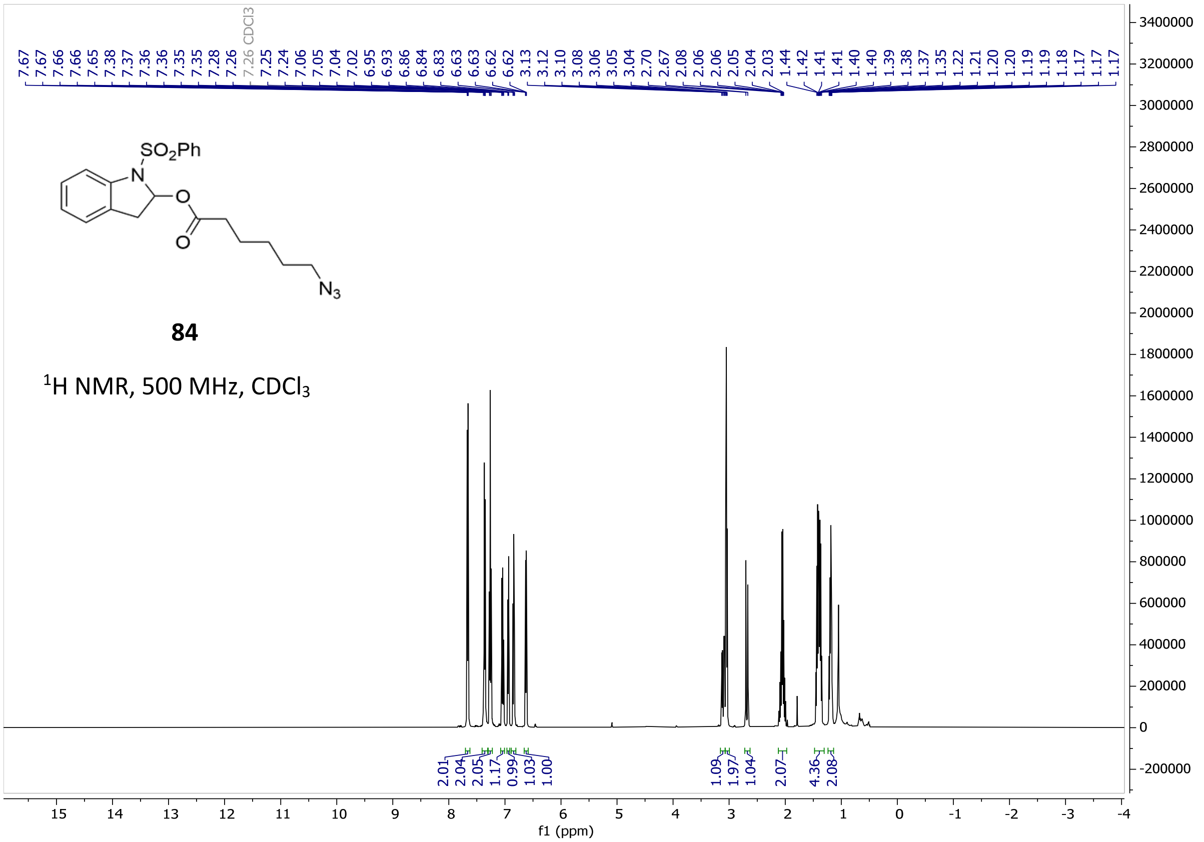
7.67 7.67 7.66 7.65 7.38 7.37 7.36 7.36 7.35 7.35 7.28 7.26 7.26 CDCl3 7.25 7.24 7.06 7.05 7.04 7.02 6.95 6.93 6.86 6.84 6.83 6.63 6.63 6.62 6.62 3.13 3.12 3.10 3.08 3.06 3.05 3.04 2.70 2.67 2.08 2.06 2.06 2.05 2.04 2.03 1.44 1.42 1.41 1.41 1.40 1.40 1.39 1.38 1.37 1.35 1.22 1.21 1.20 1.20 1.19 1.19 1.18 1.17 1.17 1.17
SO2Ph
N
O
O
N3
84
1H NMR, 500 MHz, CDCl3
2.01 2.04 2.05 1.17 0.99 1.03 1.00 1.09 1.97 1.04 2.07 4.36 2.08
f1 (ppm)

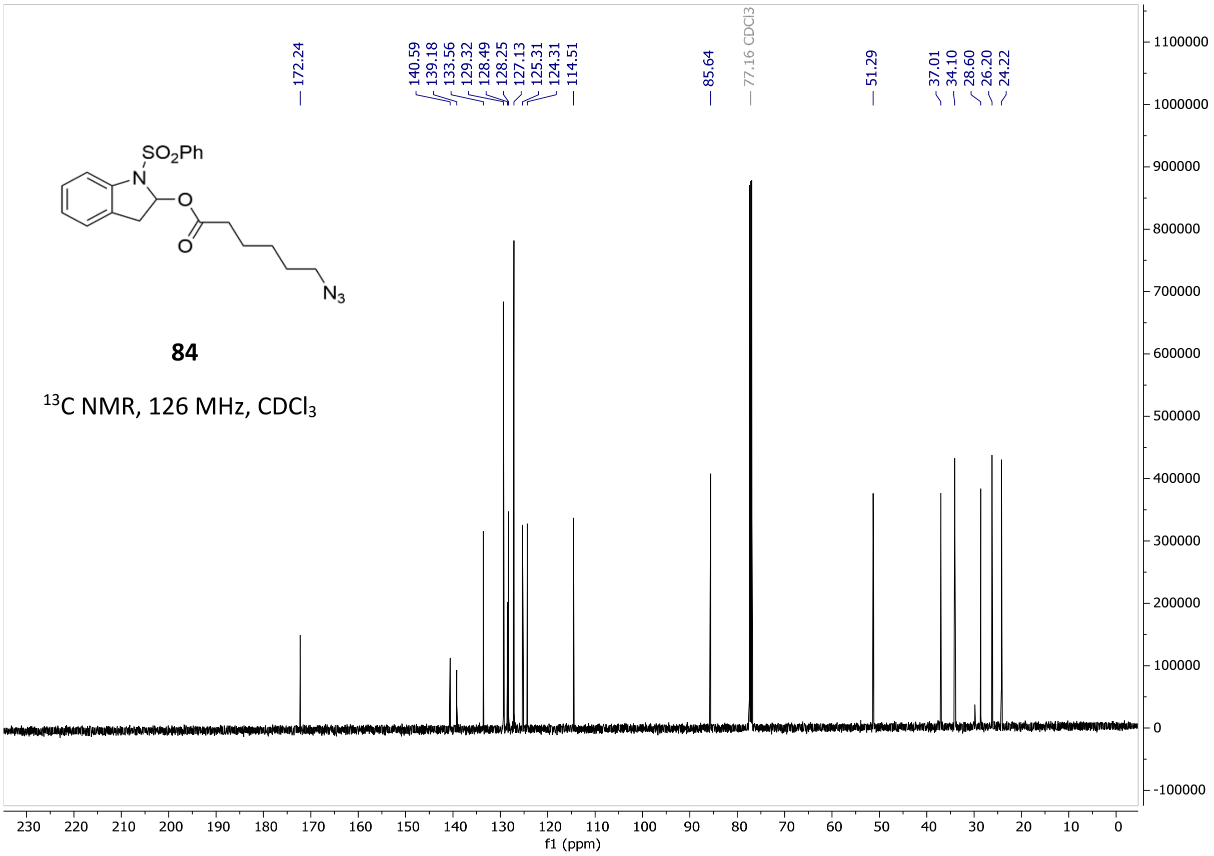
172.24
140.59 139.18 133.56 129.32 128.49 128.25 127.13 125.31 124.31 114.51
85.64
77.16 CDCl3
51.29
37.01 34.10 28.60 26.20 24.22
SO2Ph
N
O
O
N3
84
13C NMR, 126 MHz, CDCl3
f1 (ppm)

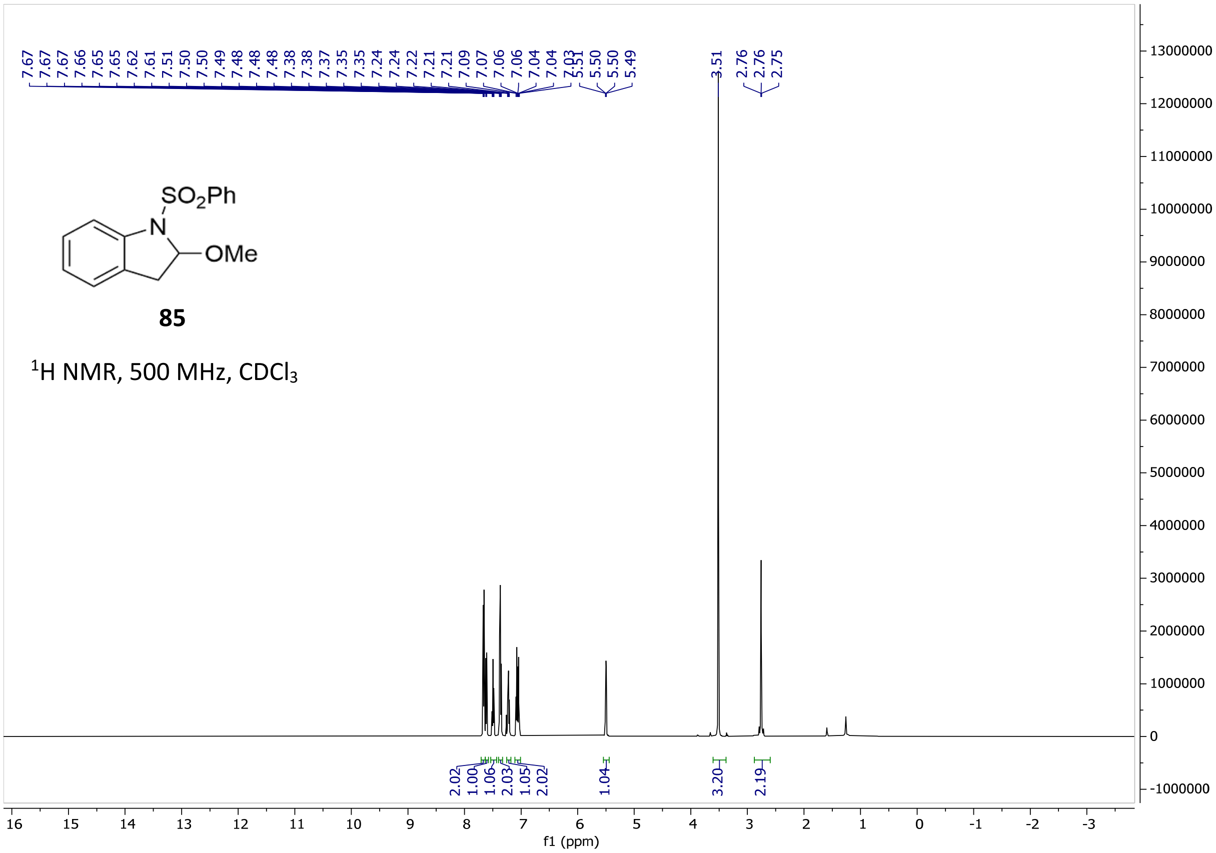
7.67 7.67 7.67 7.66 7.65 7.65 7.62 7.61 7.51 7.50 7.50 7.49 7.48 7.48 7.48 7.38 7.38 7.37 7.35 7.35 7.24 7.24 7.22 7.21 7.21 7.09 7.07 7.06 7.06 7.04 7.04 7.03 5.51 5.50 5.50 5.49 3.51 2.76 2.76 2.75
SO2Ph
N
OMe
85
1H NMR, 500 MHz, CDCl3
2.02 1.00 1.06 2.03 1.05 2.02 1.04 3.20 2.19
f1 (ppm)

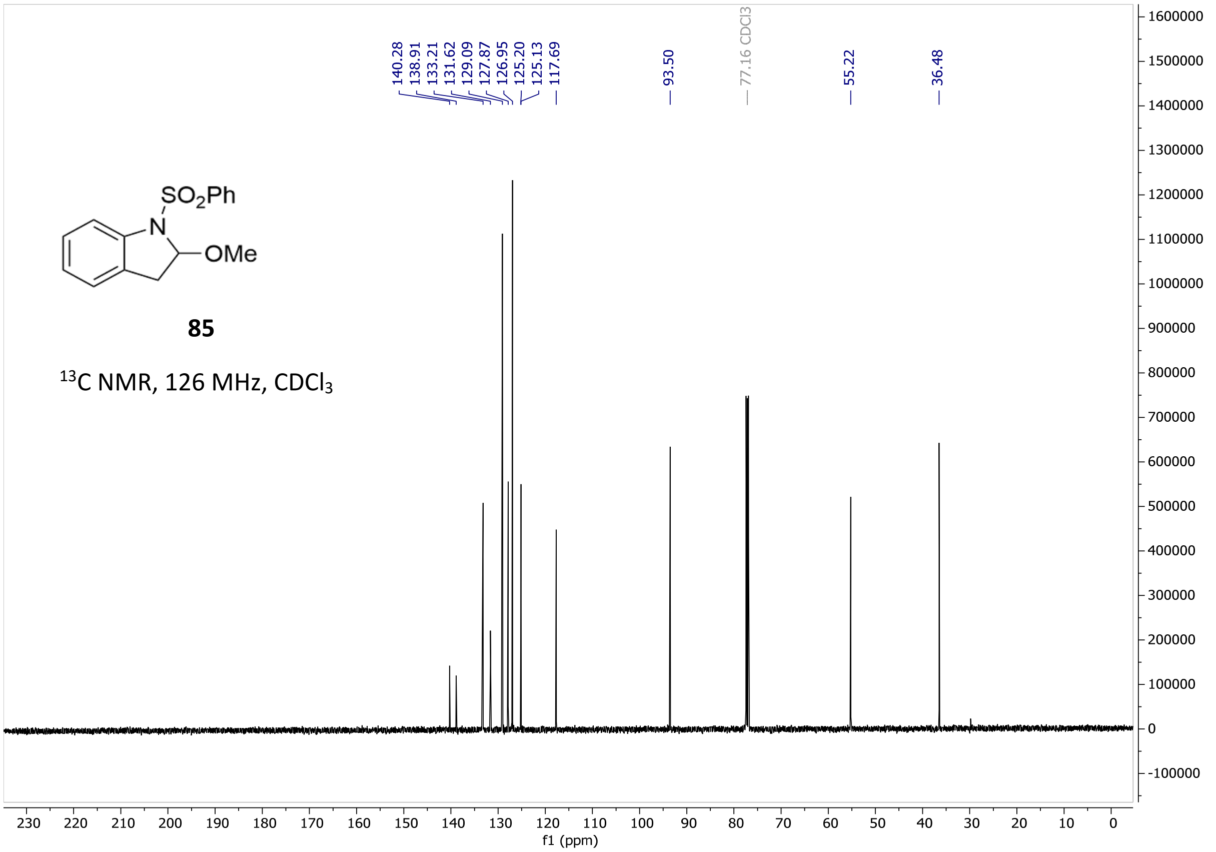
140.28 138.91 133.21 131.62 129.09 127.87 126.95 125.20 125.13 117.69
93.50
77.16 CDCl3
55.22
36.48
SO2Ph
N
OMe
85
13C NMR, 126 MHz, CDCl3
f1 (ppm)

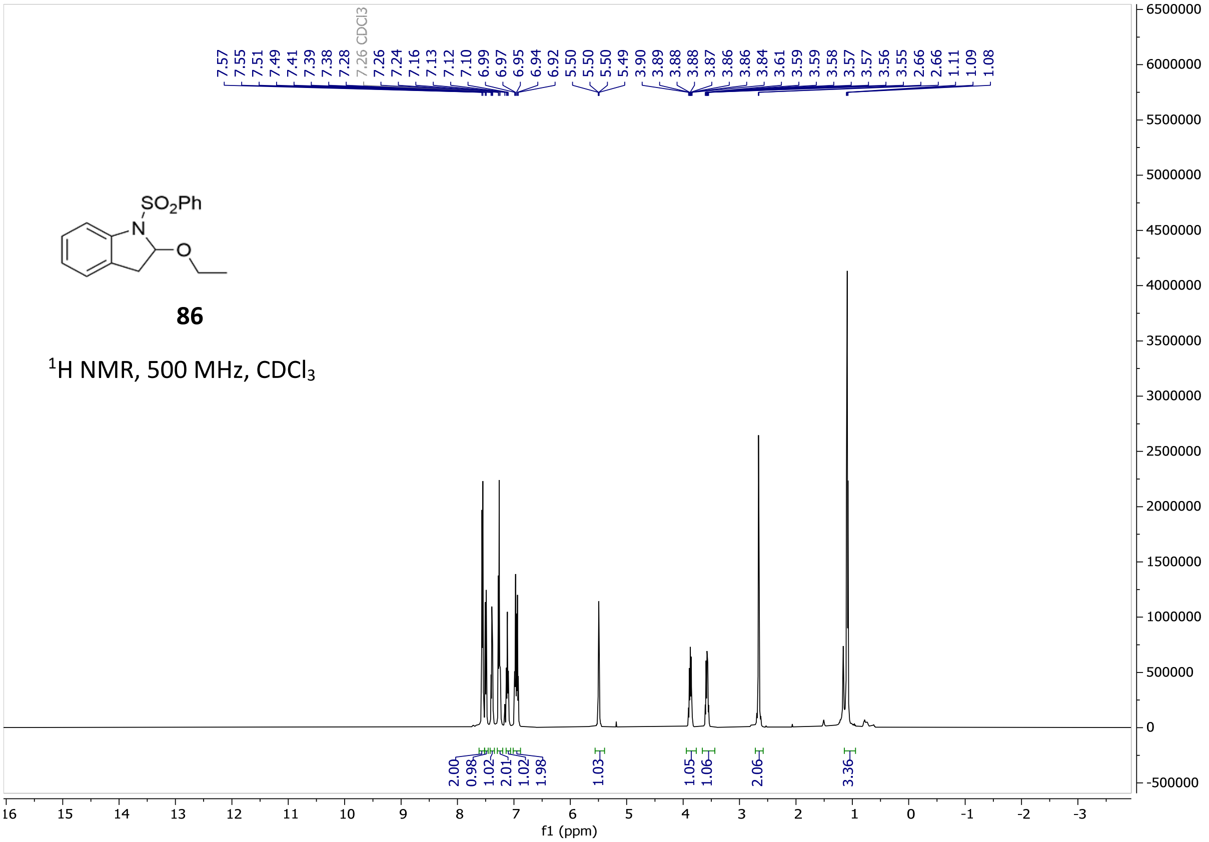
SO2Ph
N
O
86
1H NMR, 500 MHz, CDCl3
7.57 7.55 7.51 7.49 7.41 7.39 7.38 7.28 7.26 CDCl3 7.26 7.24 7.16 7.13 7.12 7.10 6.99 6.97 6.95 6.94 6.92 5.50 5.50 5.50 5.49 3.90 3.89 3.88 3.88 3.87 3.86 3.84 3.61 3.59 3.59 3.58 3.57 3.57 3.56 3.55 2.66 2.66 1.11 1.09 1.08
2.00 0.98 1.02 2.01 1.02 1.98 1.03 1.05 1.06 2.06 3.36
f1 (ppm)

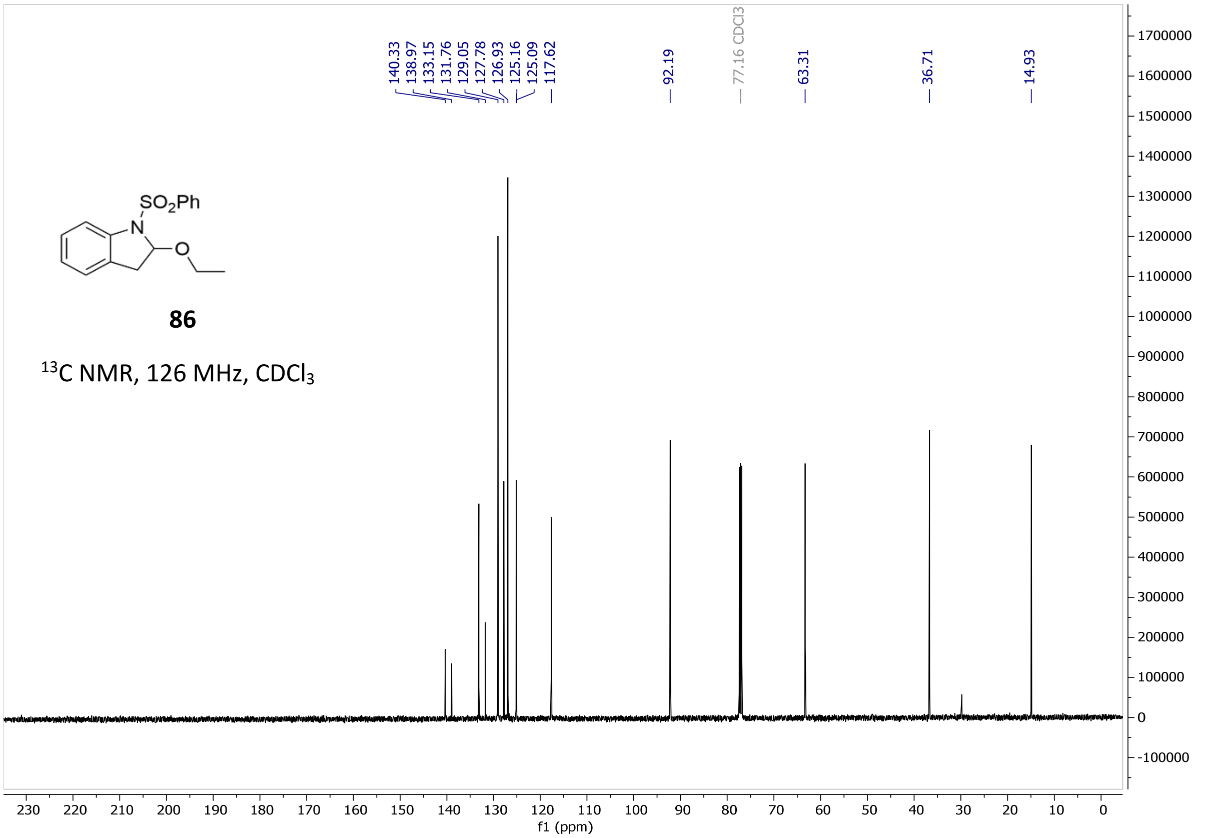
SO2Ph
N
O
86
13C NMR, 126 MHz, CDCl3
140.33 138.97 133.15 131.76 129.05 127.78 126.93 125.16 125.09 117.62 92.19 77.16 CDCl3 63.31 36.71 14.93
f1 (ppm)

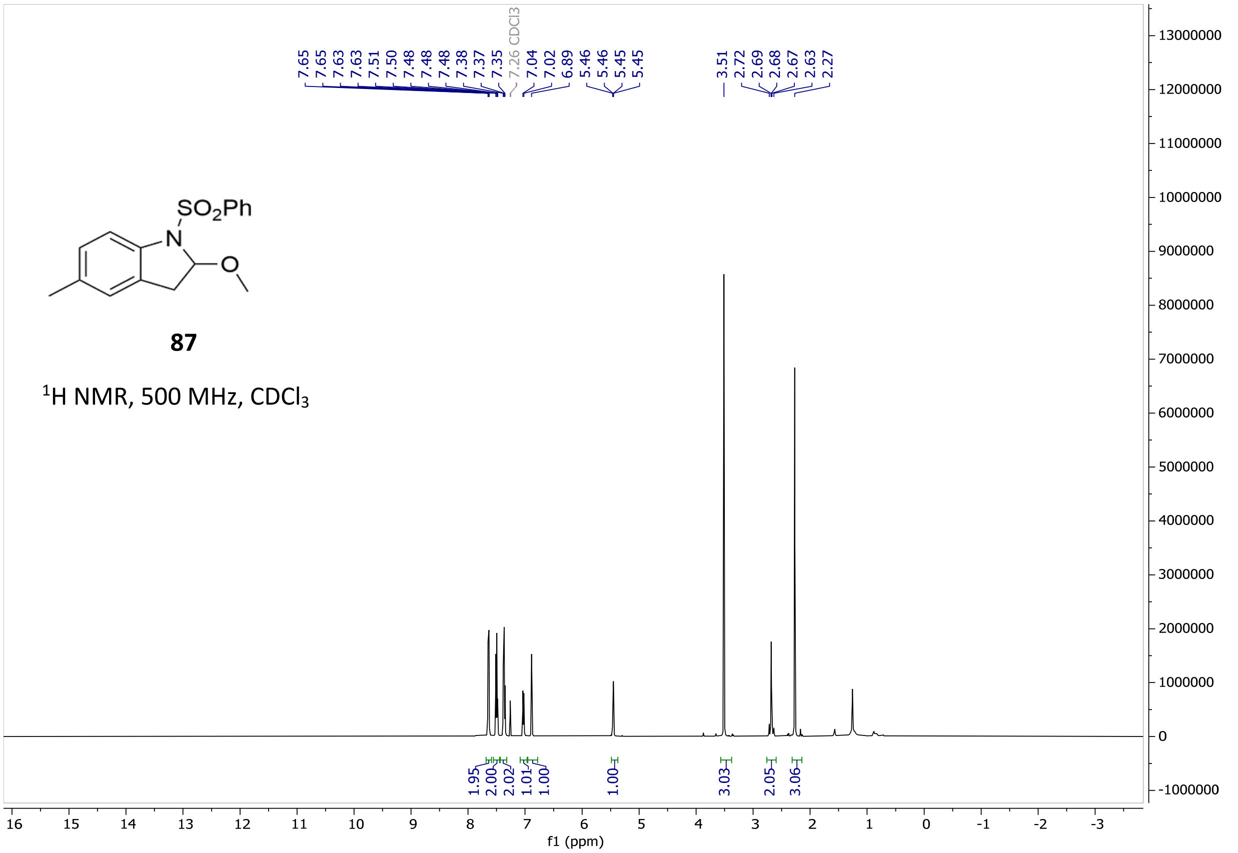

SO2Ph
N
O
87
1H NMR, 500 MHz, CDCl3
7.26 CDCl3
f1 (ppm)

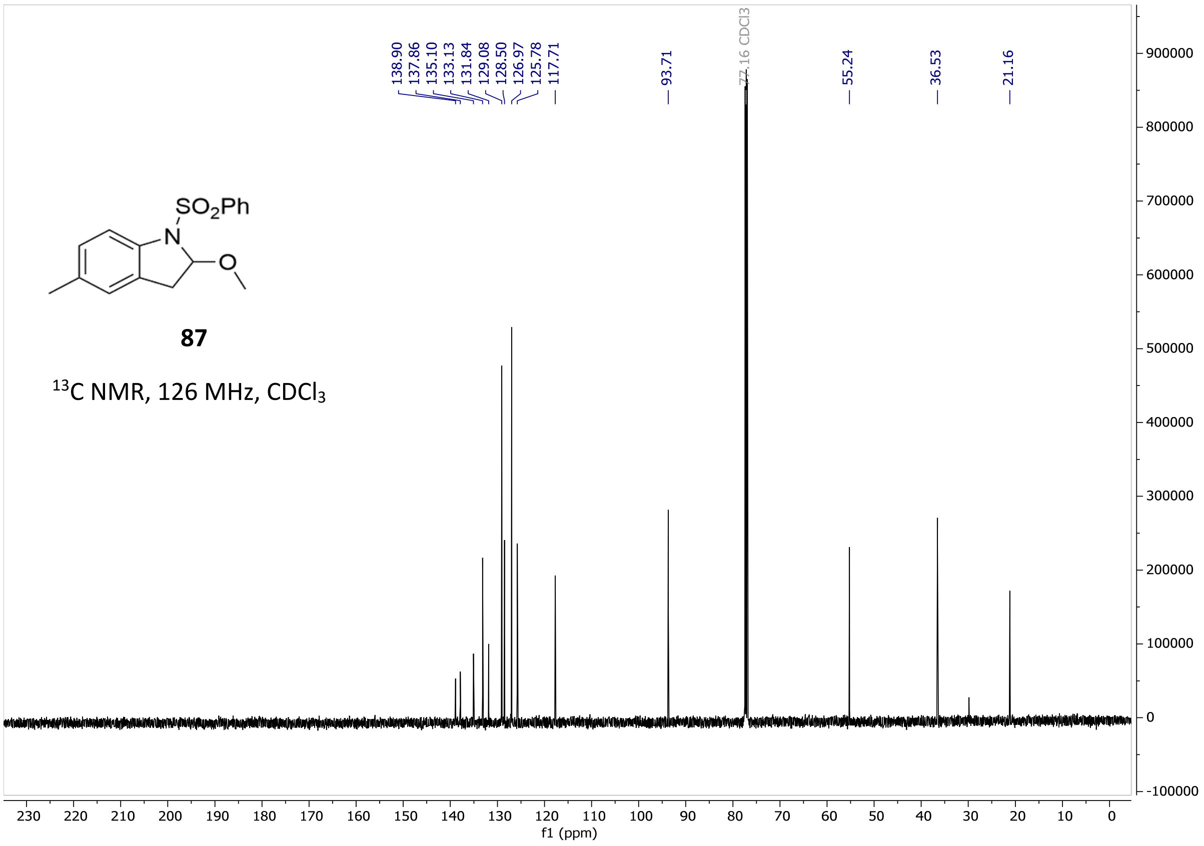

SO2Ph
N
O
87
13C NMR, 126 MHz, CDCl3
77.16 CDCl3
f1 (ppm)

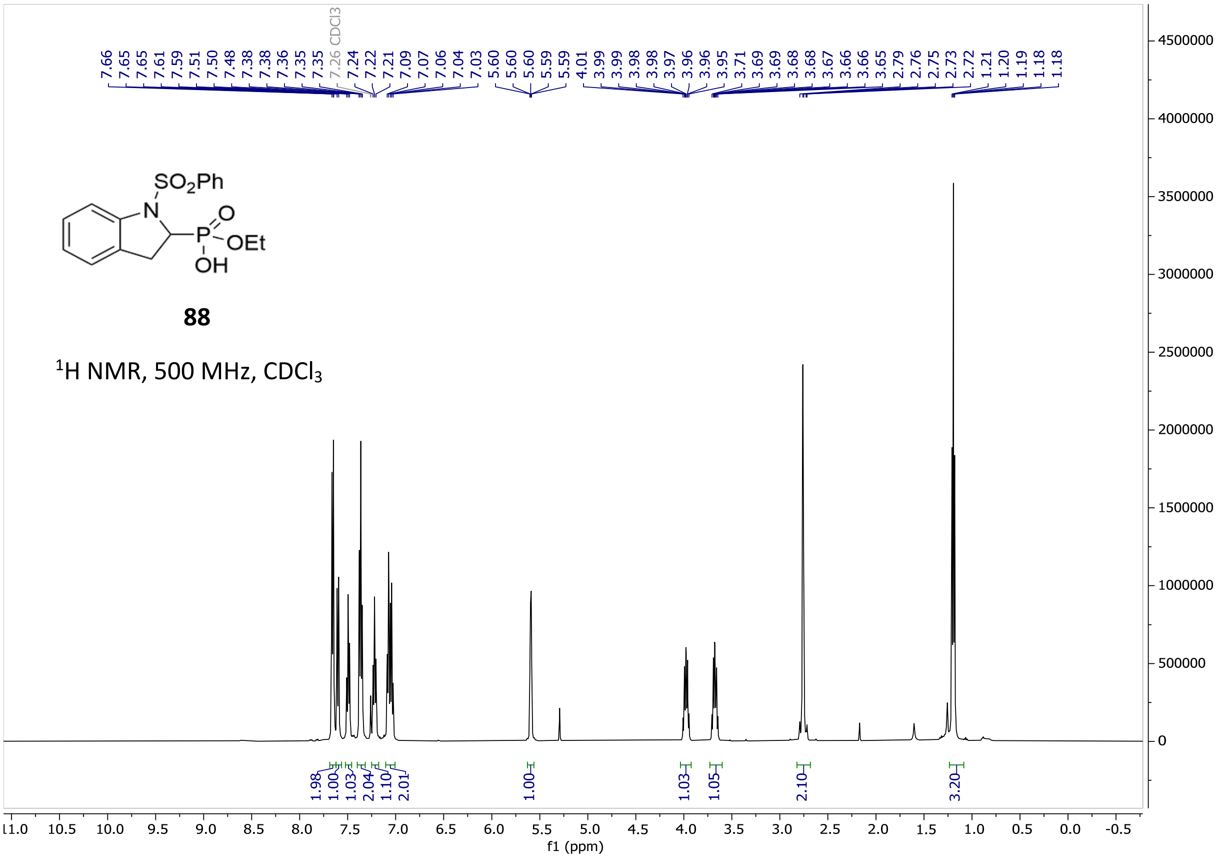
SO2Ph
N
P
O
OEt
OH
88
1H NMR, 500 MHz, CDCl3
f1 (ppm)

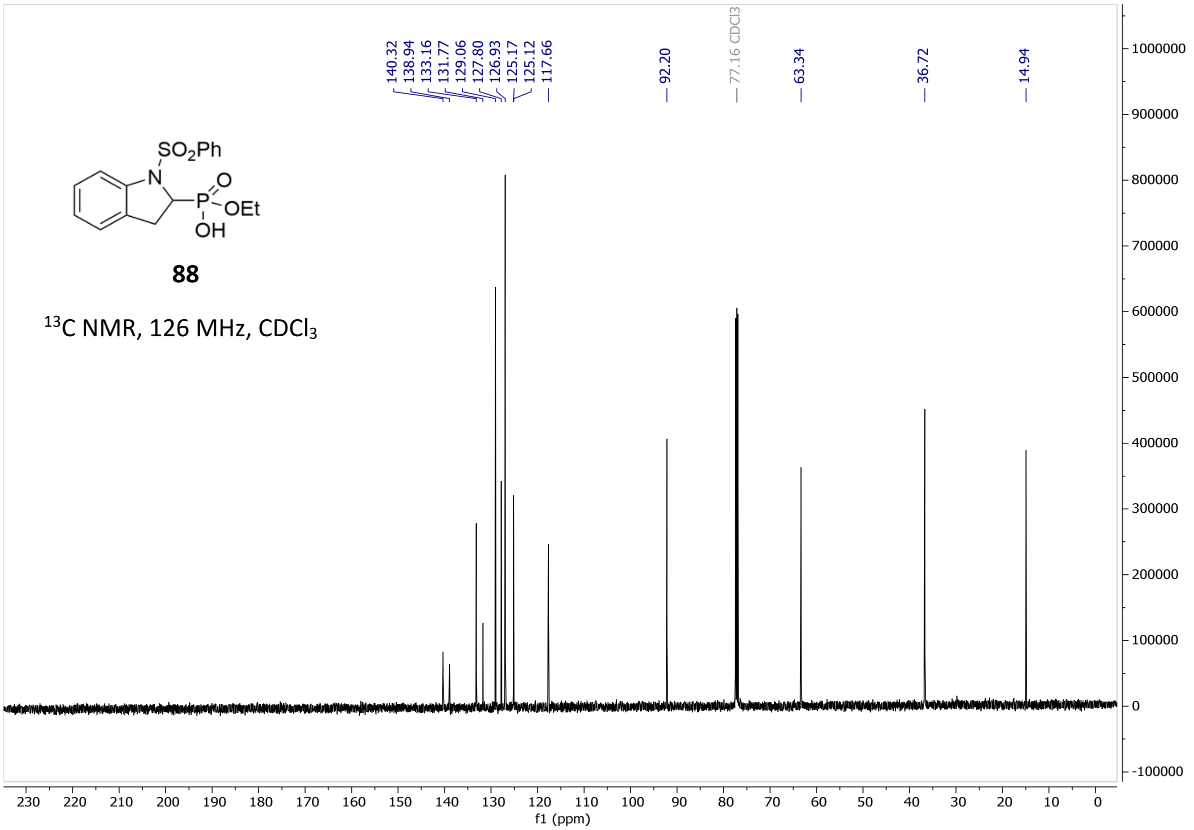
SO2Ph
N
P
O
OEt
OH
88
13C NMR, 126 MHz, CDCl3
f1 (ppm)

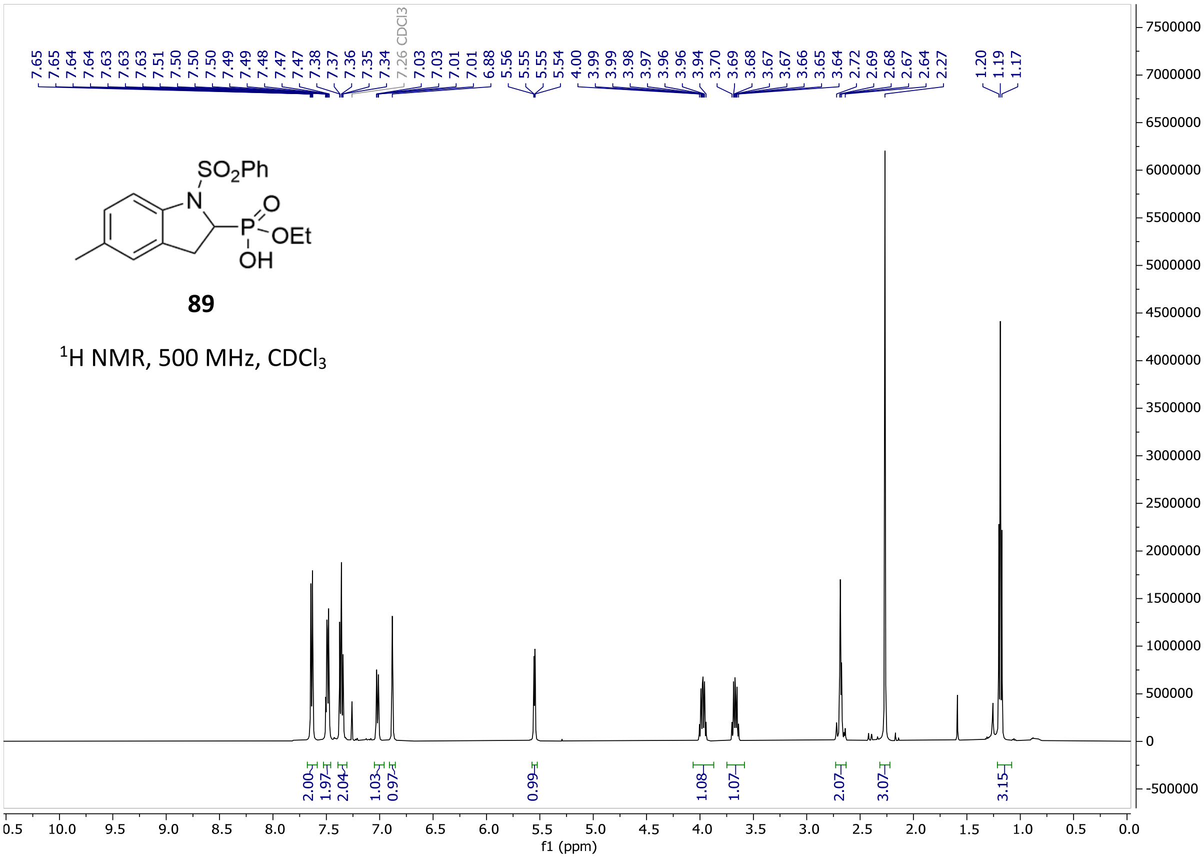
SO2Ph
N
P
O
OEt
OH
89
1H NMR, 500 MHz, CDCl3
7.26 CDCl3
f1 (ppm)

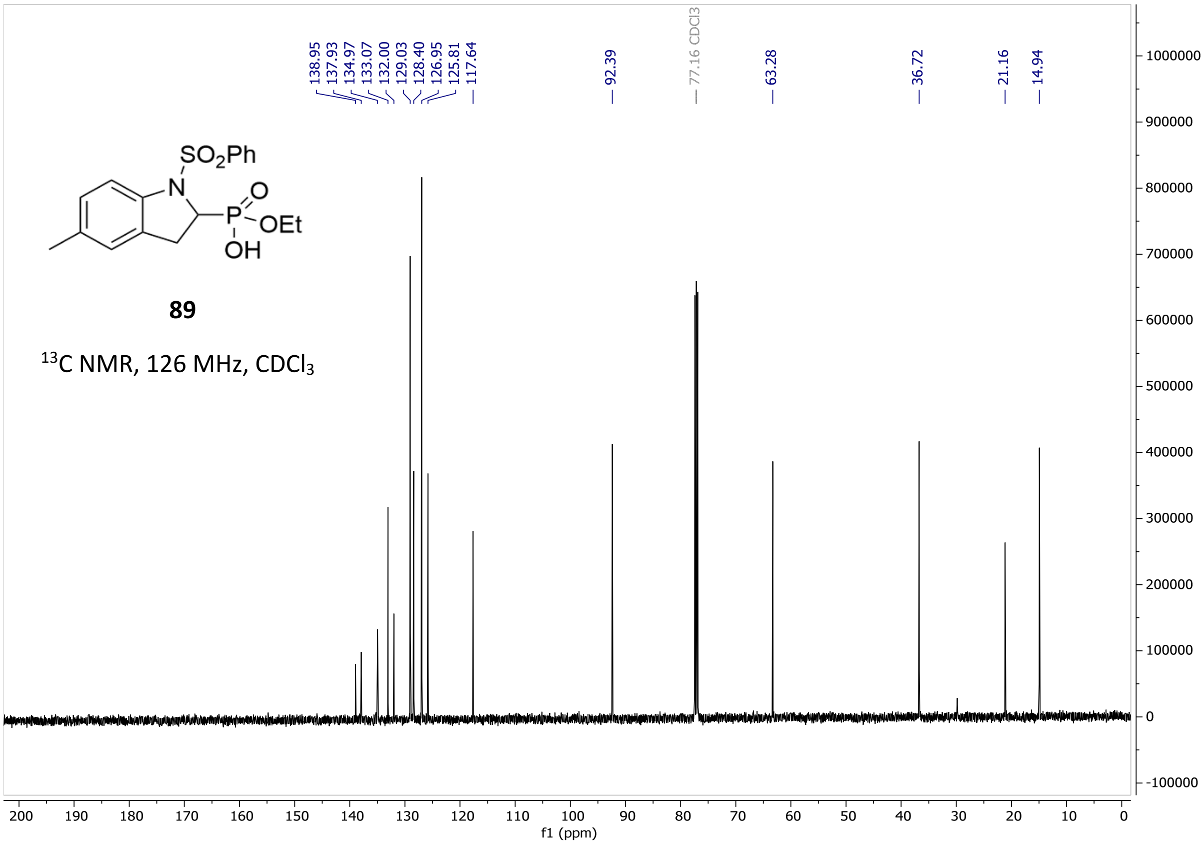
SO2Ph
N
P
O
OEt
OH
89
13C NMR, 126 MHz, CDCl3
138.95
137.93
134.97
133.07
132.00
129.03
128.40
126.95
125.81
117.64
92.39
77.16 CDCl3
63.28
36.72
21.16
14.94
f1 (ppm)

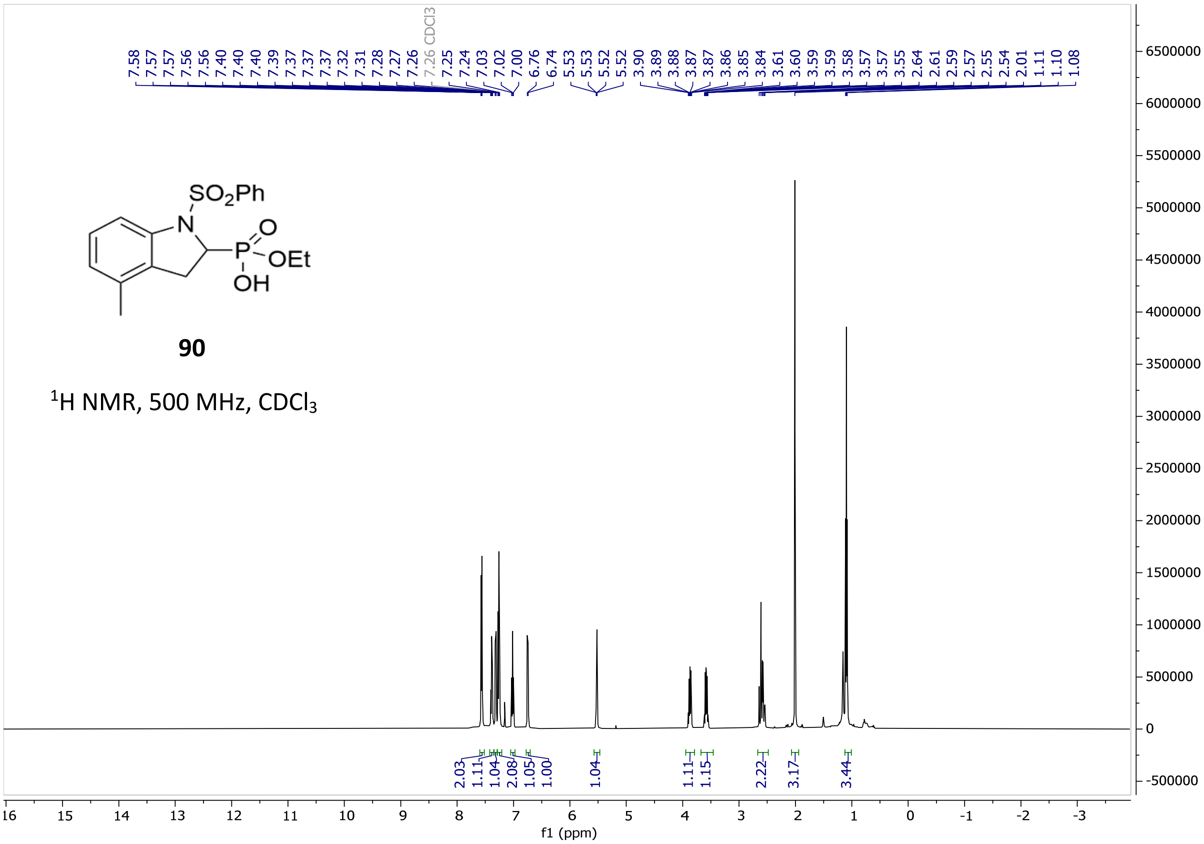
7.58 7.57 7.57 7.56 7.56 7.40 7.40 7.39 7.37 7.37 7.37 7.32 7.31 7.28 7.27 7.26 7.26 CDCl3 7.25 7.24 7.03 7.02 7.00 6.76 6.74 5.53 5.53 5.52 5.52 3.90 3.89 3.88 3.87 3.87 3.86 3.85 3.84 3.61 3.60 3.59 3.59 3.58 3.57 3.57 3.55 2.64 2.61 2.59 2.57 2.55 2.54 2.01 1.11 1.10 1.08
SO2Ph
N
P
O
OEt
OH
90
1H NMR, 500 MHz, CDCl3
2.03 1.11 1.04 2.08 1.05 1.00 1.04 1.11 1.15 2.22 3.17 3.44
f1 (ppm)

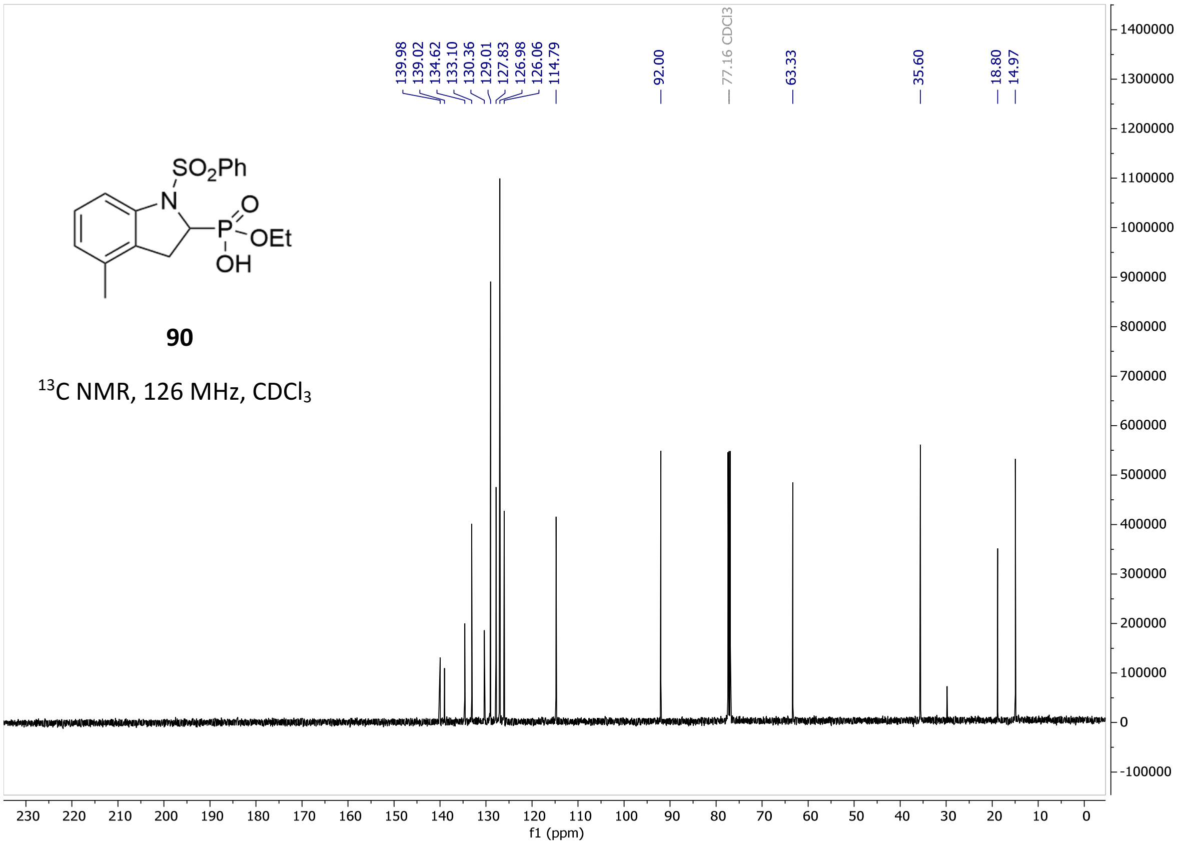
139.98 139.02 134.62 133.10 130.36 129.01 127.83 126.98 126.06 114.79 92.00 77.16 CDCl3 63.33 35.60 18.80 14.97
SO2Ph
N
P
O
OEt
OH
90
13C NMR, 126 MHz, CDCl3
f1 (ppm)

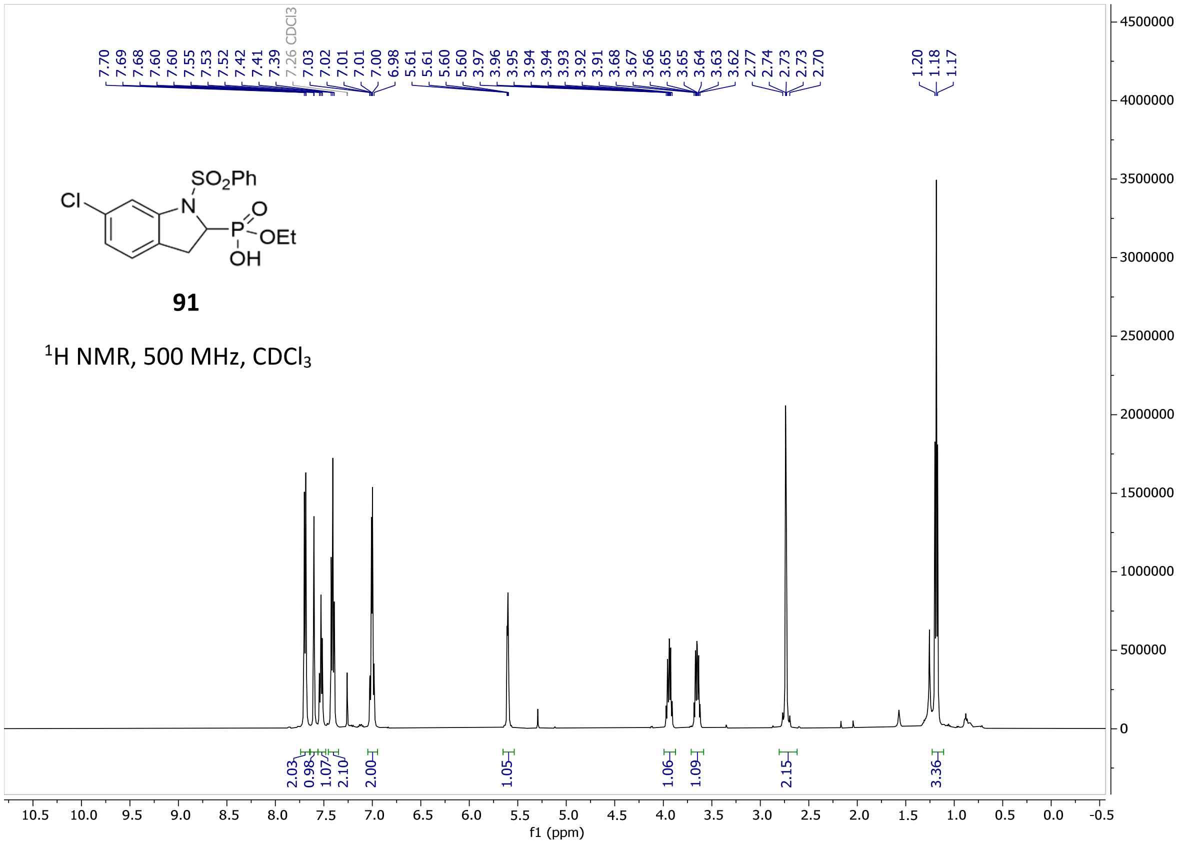
7.70
7.69
7.68
7.60
7.60
7.55
7.53
7.52
7.42
7.41
7.39
7.26 CDCl3
7.03
7.02
7.01
7.01
7.00
6.98
5.61
5.60
5.60
3.97
3.96
3.95
3.94
3.94
3.93
3.92
3.91
3.68
3.67
3.66
3.65
3.65
3.64
3.63
3.62
2.77
2.74
2.73
2.73
2.70
1.20
1.18
1.17
SO2Ph
Cl
N
O
P
OEt
OH
91
1H NMR, 500 MHz, CDCl3
2.03
0.98
1.07
2.10
2.00
1.05
1.06
1.09
2.15
3.36
f1 (ppm)

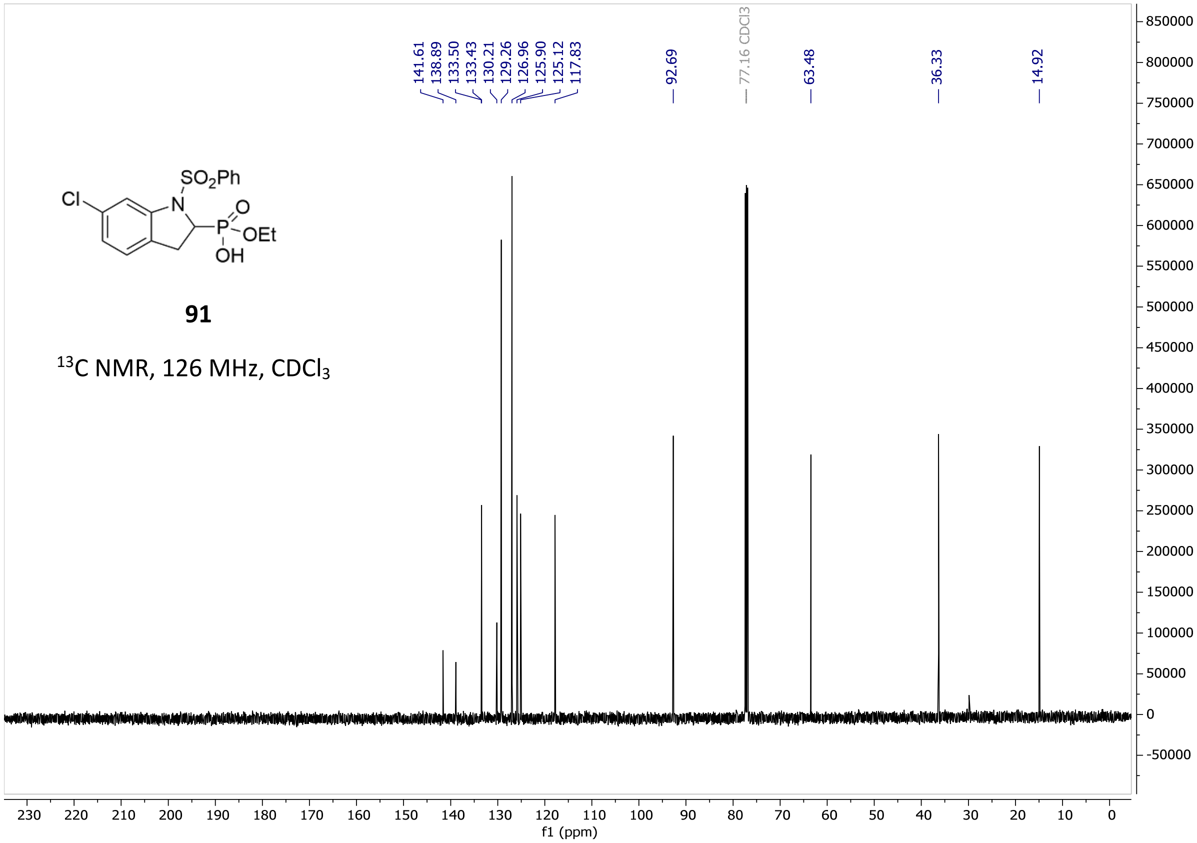
141.61
138.89
133.50
133.43
130.21
129.26
126.96
125.90
125.12
117.83
92.69
77.16 CDCl3
63.48
36.33
14.92
SO2Ph
Cl
N
O
P
OEt
OH
91
13C NMR, 126 MHz, CDCl3
f1 (ppm)

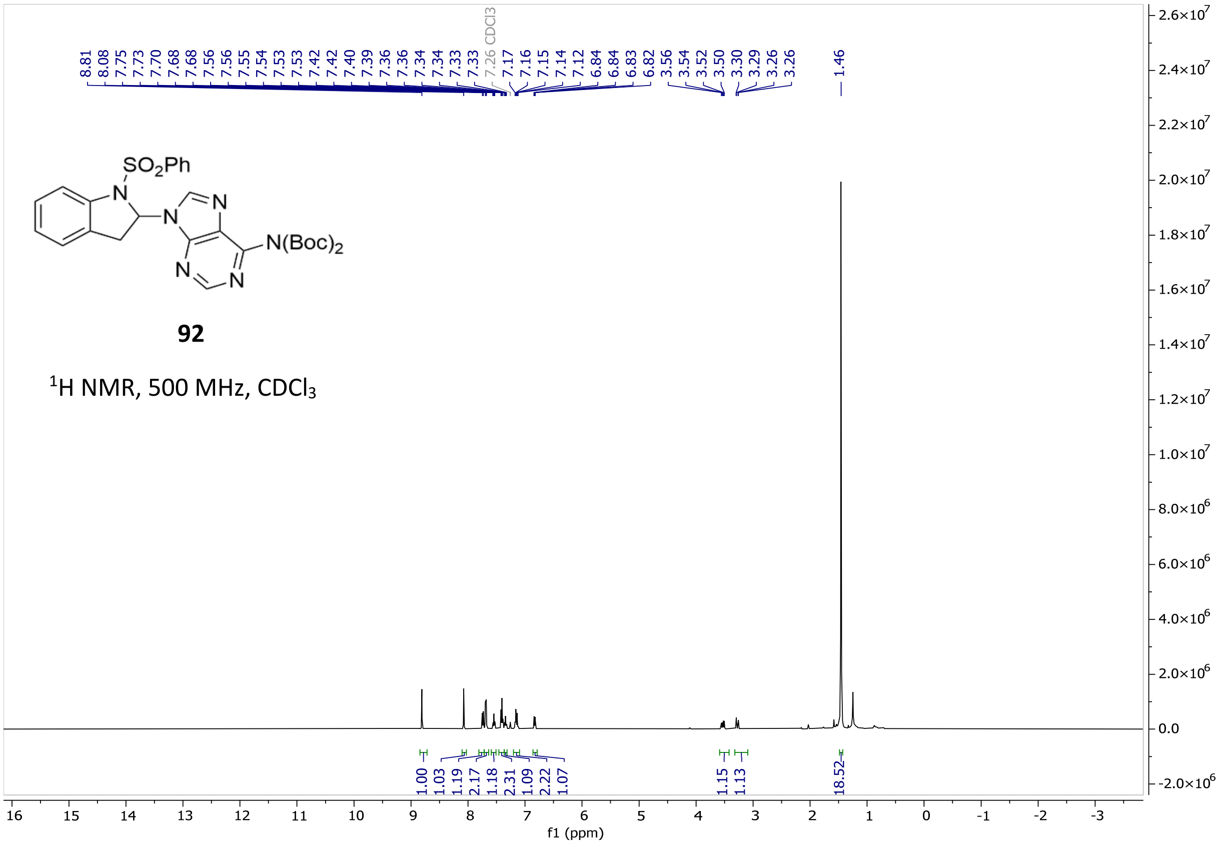
8.81 8.08 7.75 7.73 7.70 7.68 7.68 7.56 7.56 7.55 7.54 7.53 7.53 7.42 7.42 7.40 7.39 7.36 7.34 7.34 7.33 7.33 7.26 CDCl3 7.17 7.16 7.15 7.14 7.12 6.84 6.84 6.83 6.82 3.56 3.54 3.52 3.50 3.30 3.29 3.26 3.26
1.46
SO2Ph
N
N
N
N(Boc)2
N
N
92
1H NMR, 500 MHz, CDCl3
1.00 1.03 1.19 2.17 1.18 2.31 1.09 2.22 1.07
1.15 1.13
18.52
f1 (ppm)

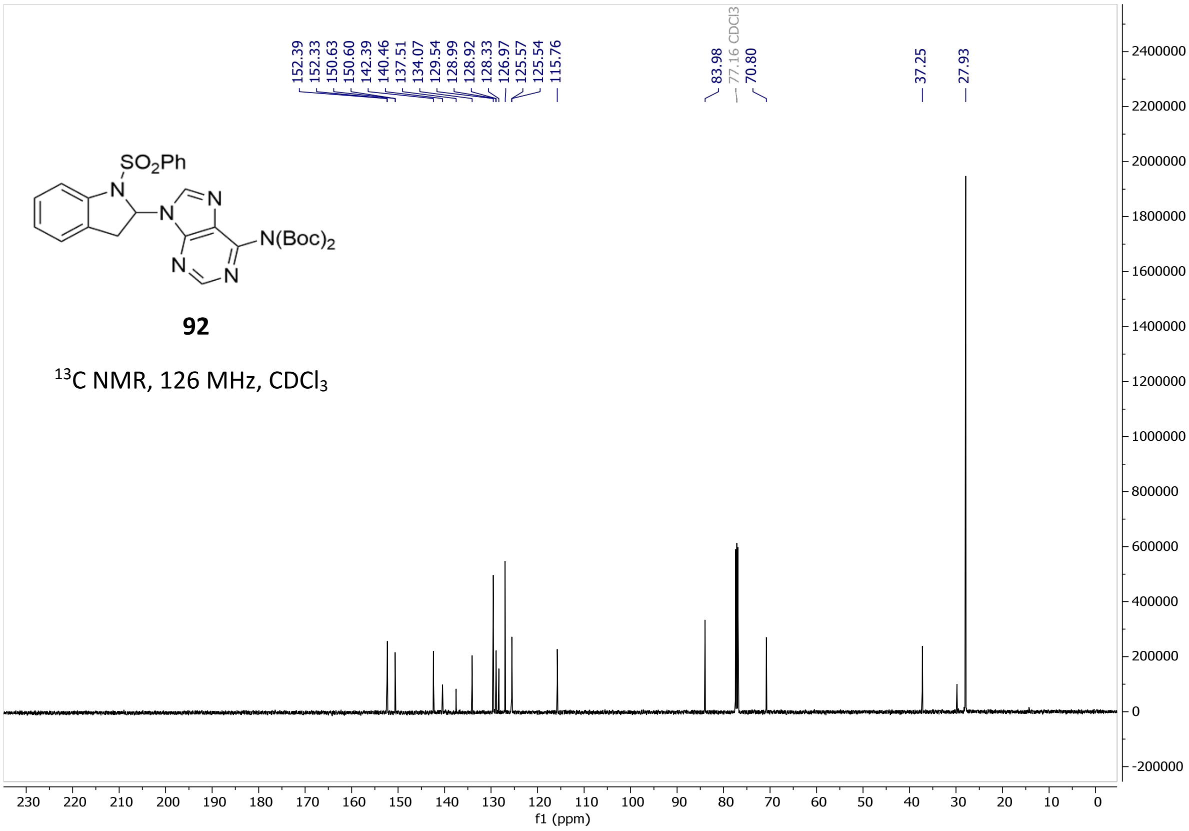
152.39 152.33 150.63 150.60 142.39 140.46 137.51 134.07 129.54 128.99 128.92 128.33 126.97 125.57 125.54 115.76
83.98 77.16 CDCl3 70.80
37.25
27.93
SO2Ph
N
N
N
N(Boc)2
N
N
92
13C NMR, 126 MHz, CDCl3
f1 (ppm)

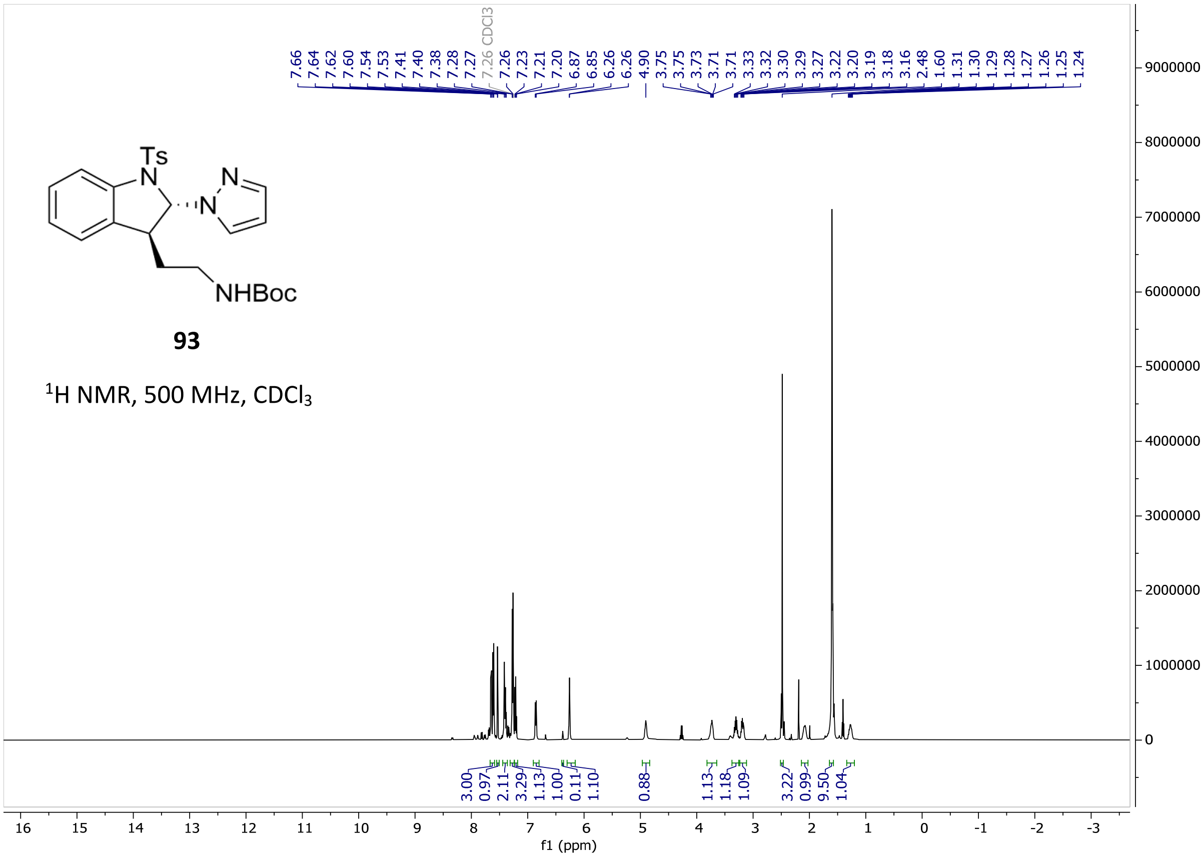
7.66 7.64 7.62 7.60 7.54 7.53 7.41 7.40 7.38 7.28 7.27 7.26 CDCl3 7.26 7.23 7.21 7.20 6.87 6.85 6.26 6.26 4.90 3.75 3.75 3.73 3.71 3.71 3.33 3.32 3.30 3.29 3.27 3.22 3.20 3.19 3.18 3.16 2.48 1.60 1.31 1.30 1.29 1.28 1.27 1.26 1.25 1.24
Ts
N
N
N
NHBoc
93
^{1}H NMR, 500 MHz, $CDCl_3$
3.00 0.97 2.11 3.29 1.13 1.00 0.11 1.10 0.88 1.13 1.18 1.09 3.22 0.99 9.50 1.04
f1 (ppm)

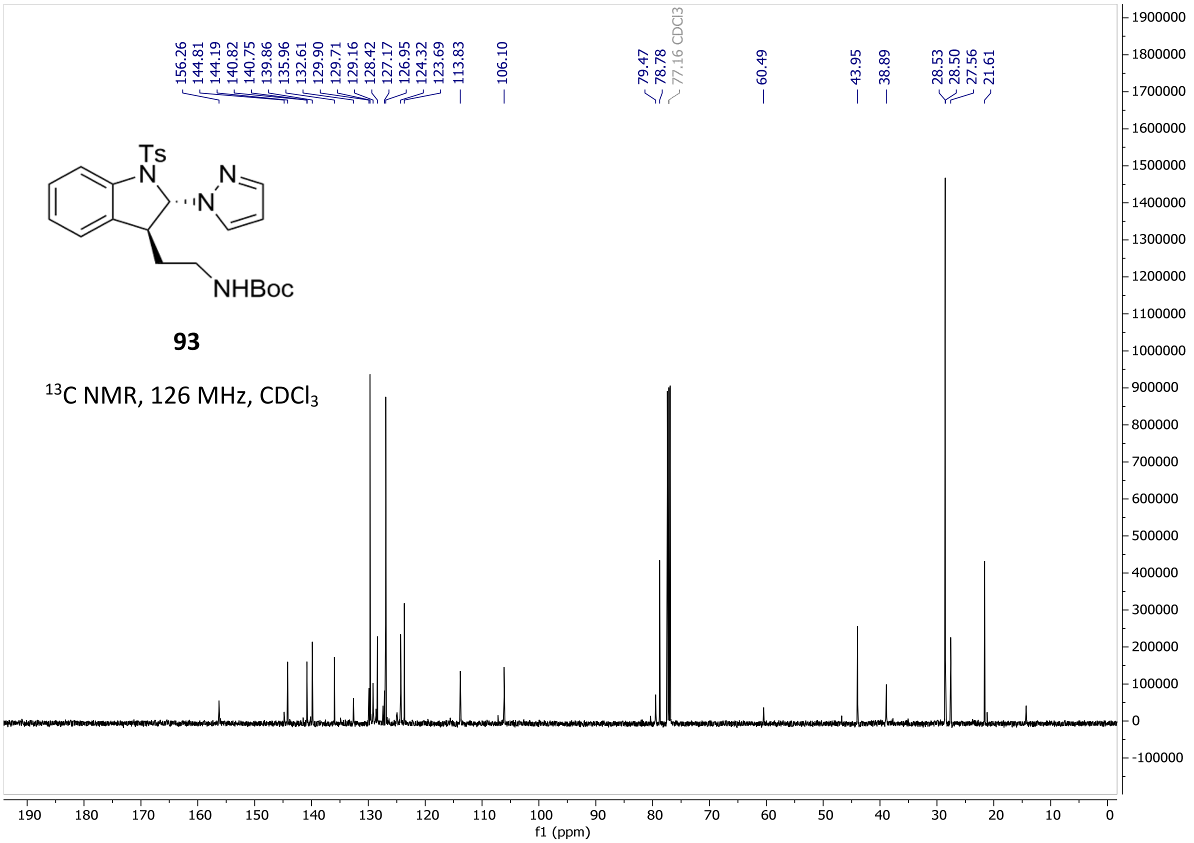
156.26 144.81 144.19 140.82 140.75 139.86 135.96 132.61 129.90 129.71 129.16 128.42 127.17 126.95 124.32 123.69 113.83 106.10 79.47 78.78 77.16 CDCl3 60.49 43.95 38.89 28.53 28.50 27.56 21.61
Ts
N
N
N
NHBoc
93
^{13}C NMR, 126 MHz, $CDCl_3$
f1 (ppm)

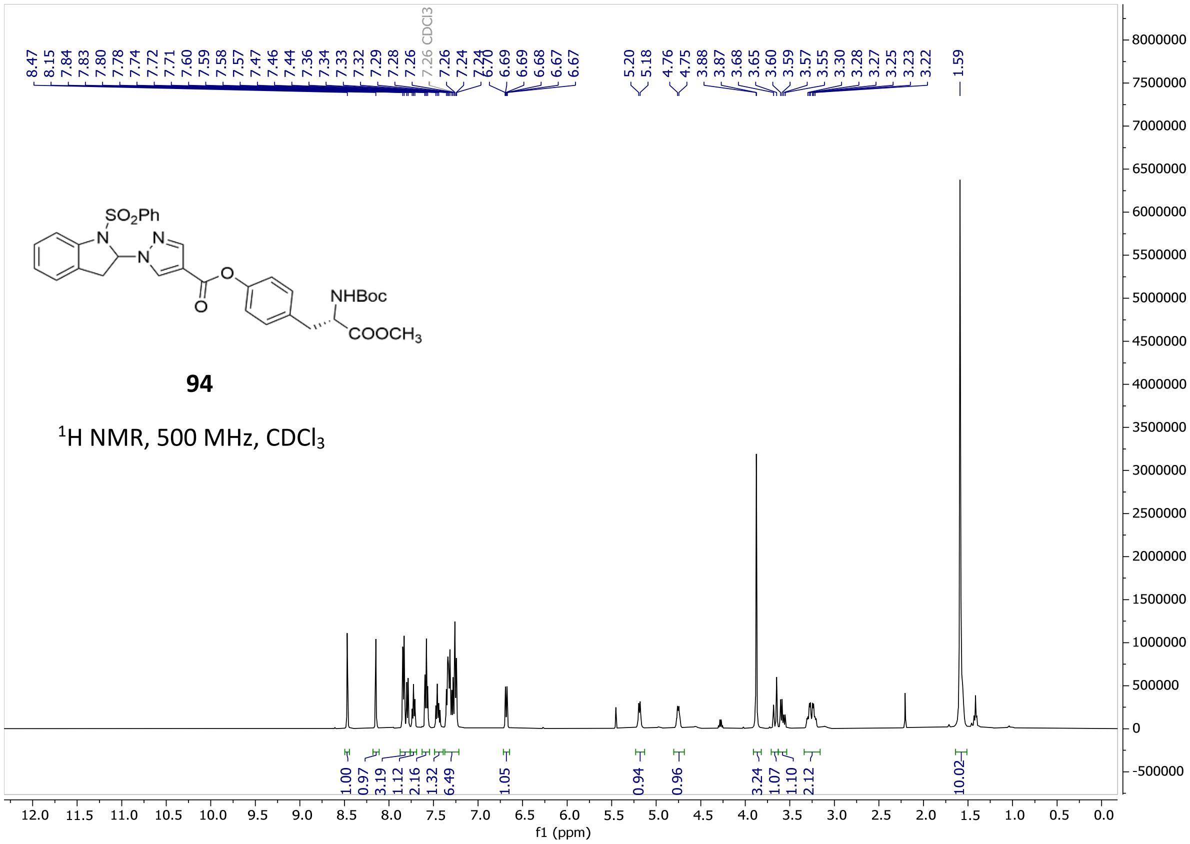

94

^{1}H NMR, 500 MHz, $CDCl_3$

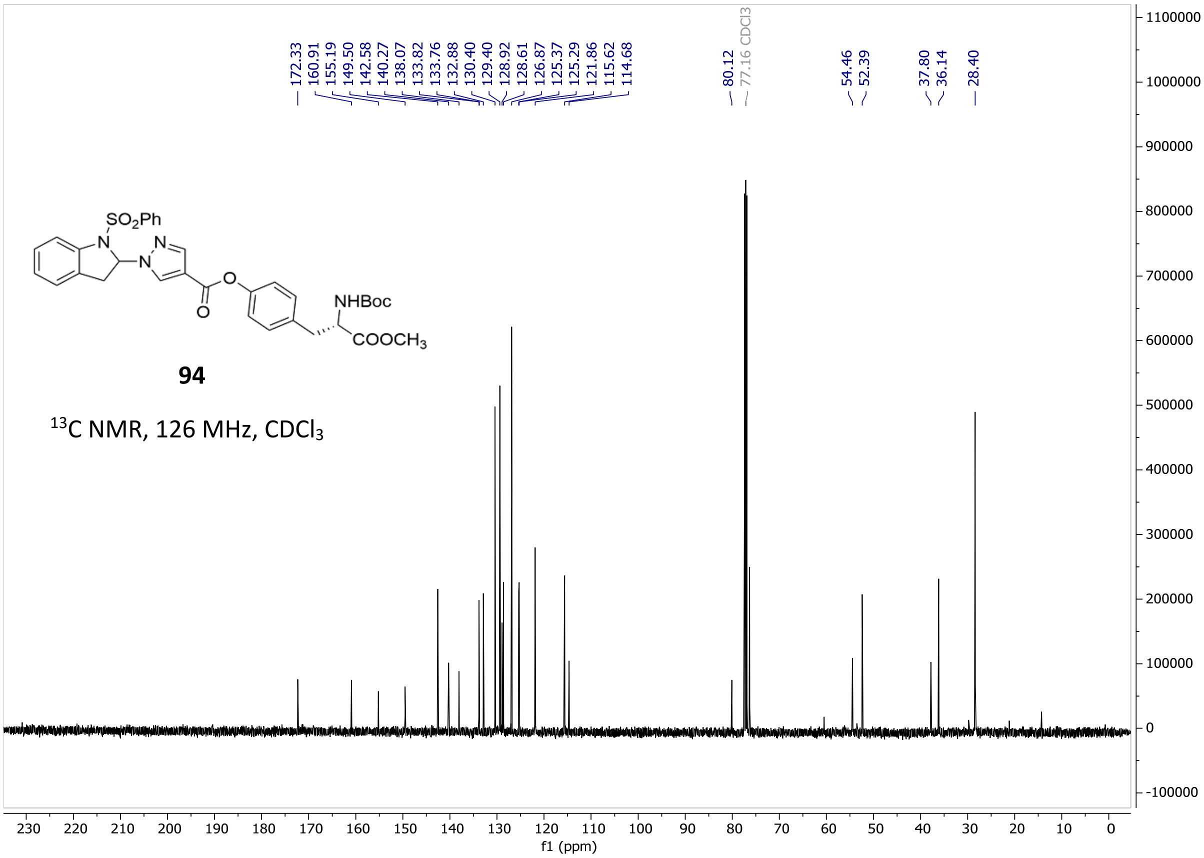

94

^{13}C NMR, 126 MHz, $CDCl_3$

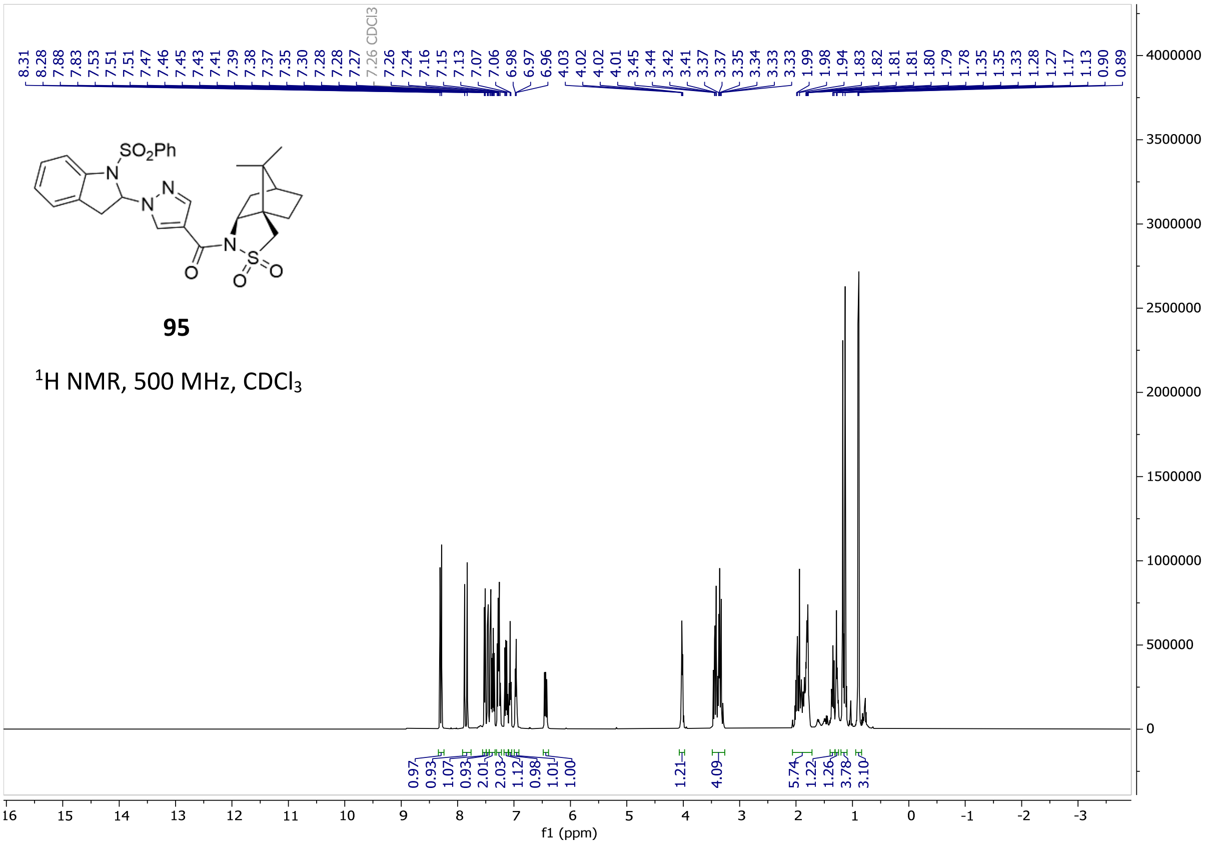
8.31
8.28
7.88
7.83
7.53
7.51
7.51
7.47
7.46
7.45
7.43
7.41
7.39
7.38
7.37
7.35
7.30
7.28
7.28
7.27
7.26 CDCl3
7.26
7.24
7.16
7.15
7.13
7.07
7.06
6.98
6.97
6.96
4.03
4.02
4.01
3.45
3.44
3.42
3.41
3.37
3.37
3.35
3.34
3.33
3.33
1.99
1.98
1.94
1.83
1.82
1.81
1.81
1.80
1.79
1.78
1.35
1.35
1.33
1.28
1.27
1.17
1.13
0.90
0.89
SO2Ph
N
N
N
N
S
O
O
O
95
1H NMR, 500 MHz, CDCl3
0.97
0.93
1.07
0.93
2.01
2.03
1.12
0.98
1.01
1.00
1.21
4.09
5.74
1.22
1.26
3.78
3.10
f1 (ppm)

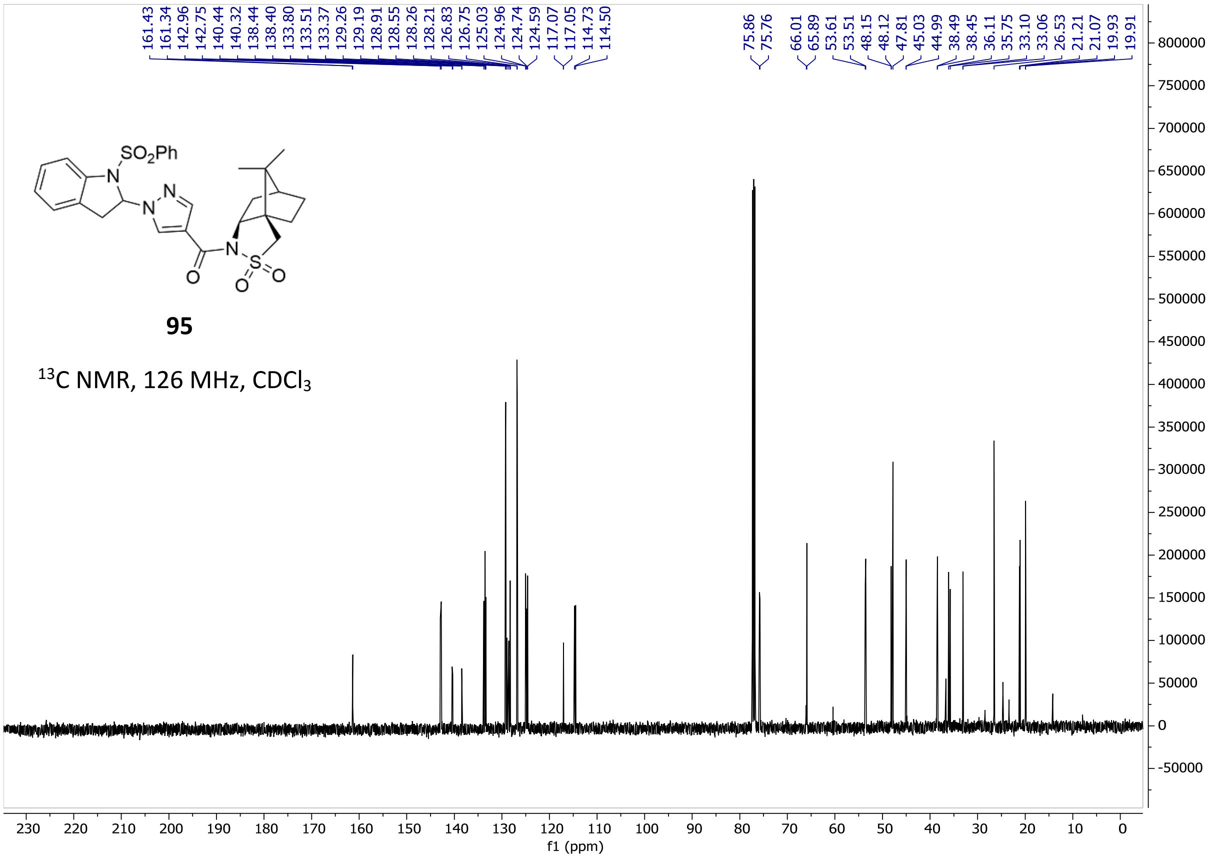
161.43
161.34
142.96
142.75
140.44
140.32
138.44
138.40
133.80
133.51
133.37
129.26
129.19
128.91
128.55
128.26
128.21
126.83
126.75
125.03
124.96
124.74
124.59
117.07
117.05
114.73
114.50
75.86
75.76
66.01
65.89
53.61
53.51
48.15
48.12
47.81
45.03
44.99
38.49
38.45
36.11
35.75
33.10
33.06
26.53
21.21
21.07
19.93
19.91
SO2Ph
N
N
N
N
S
O
O
O
95
13C NMR, 126 MHz, CDCl3
f1 (ppm)

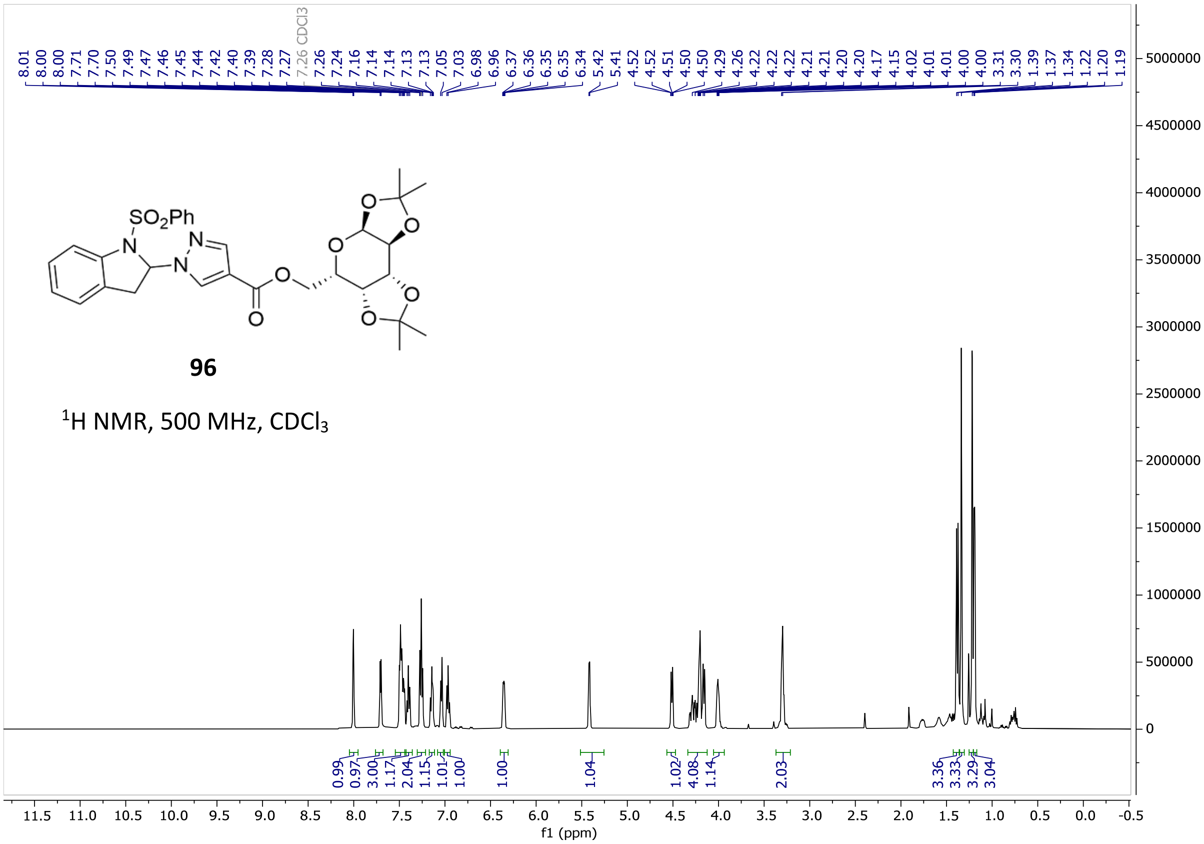
8.01 8.00 8.00 7.71 7.70 7.50 7.49 7.47 7.46 7.45 7.44 7.42 7.40 7.39 7.28 7.27 7.26 CDCl3 7.26 7.24 7.16 7.14 7.14 7.13 7.13 7.05 7.03 6.98 6.96 6.37 6.36 6.35 6.35 6.34 5.42 5.41 4.52 4.51 4.50 4.50 4.29 4.26 4.22 4.22 4.22 4.21 4.21 4.20 4.20 4.17 4.15 4.02 4.01 4.01 4.00 4.00 3.31 3.30 1.39 1.37 1.34 1.22 1.20 1.19
SO2Ph
N
N
N
O
O
O
O
O
O
O
O
96
1H NMR, 500 MHz, CDCl3
0.99 0.97 3.00 1.17 2.04 1.15 1.01 1.00 1.00 1.04 1.02 4.08 1.14 2.03 3.36 3.33 3.29 3.04
f1 (ppm)

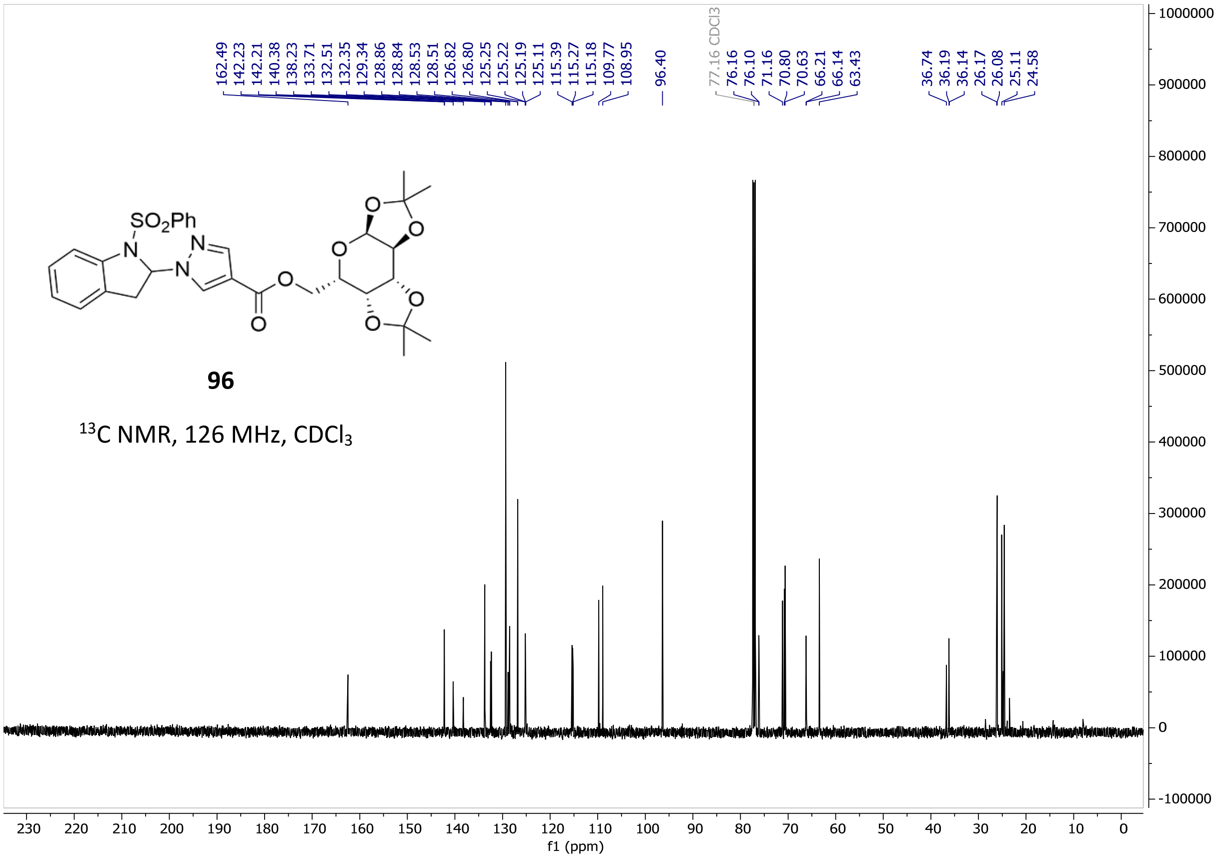
162.49 142.23 142.21 140.38 138.23 133.71 132.51 132.35 129.34 128.86 128.84 128.53 128.51 126.82 126.80 125.25 125.22 125.19 125.11 115.39 115.27 115.18 109.77 108.95 96.40 77.16 CDCl3 76.16 76.10 71.16 70.80 70.63 66.21 66.14 63.43 36.74 36.19 36.14 26.17 26.08 25.11 24.58
SO2Ph
N
N
N
O
O
O
O
O
O
O
O
96
13C NMR, 126 MHz, CDCl3
f1 (ppm)

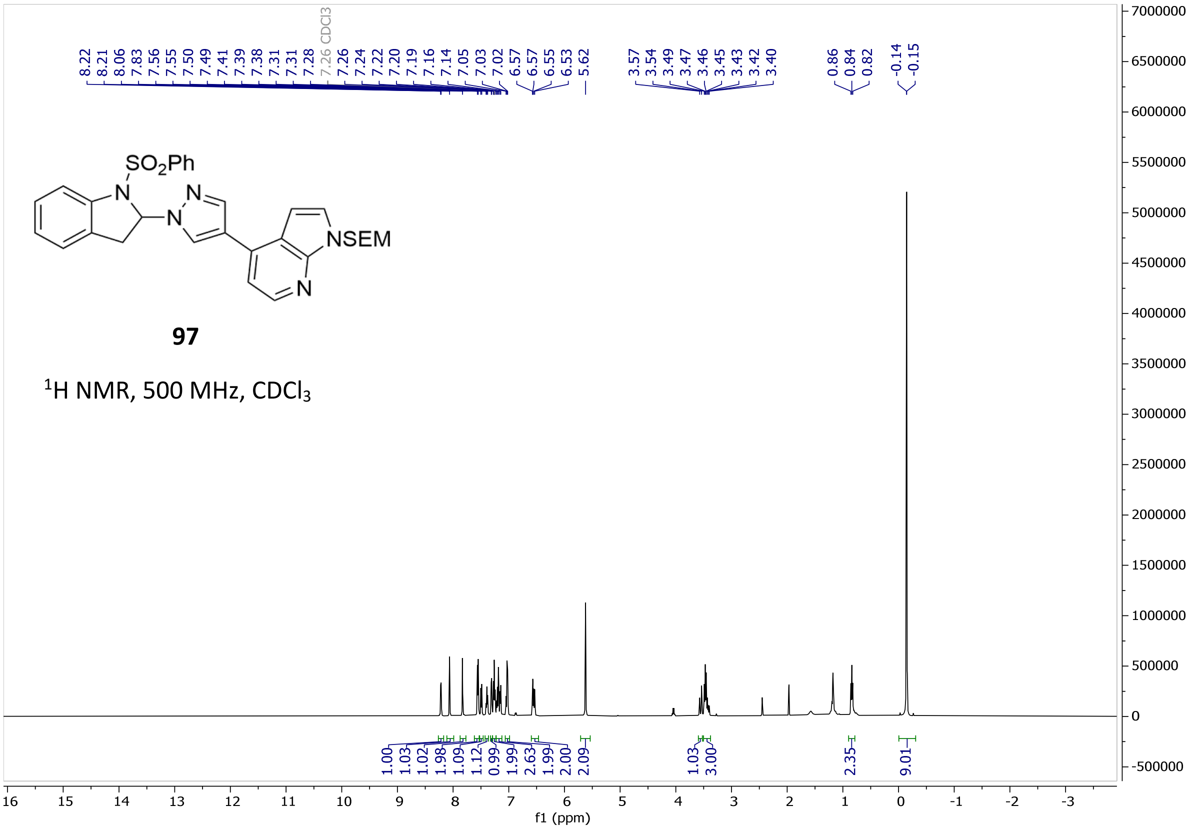
8.22 8.21 8.06 7.83 7.56 7.55 7.50 7.49 7.41 7.39 7.38 7.31 7.31 7.28 7.26 CDCl3 7.26 7.24 7.22 7.20 7.19 7.16 7.14 7.05 7.03 7.02 6.57 6.57 6.55 6.53 5.62
3.57 3.54 3.49 3.47 3.46 3.45 3.43 3.42 3.40
0.86 0.84 0.82 -0.14 -0.15
SO2Ph
N
N
N
NSEM
N
97
1H NMR, 500 MHz, CDCl3
1.00 1.03 1.02 1.98 1.09 1.12 0.99 1.99 2.63 1.99 2.00 2.09
1.03 3.00
2.35
9.01
f1 (ppm)

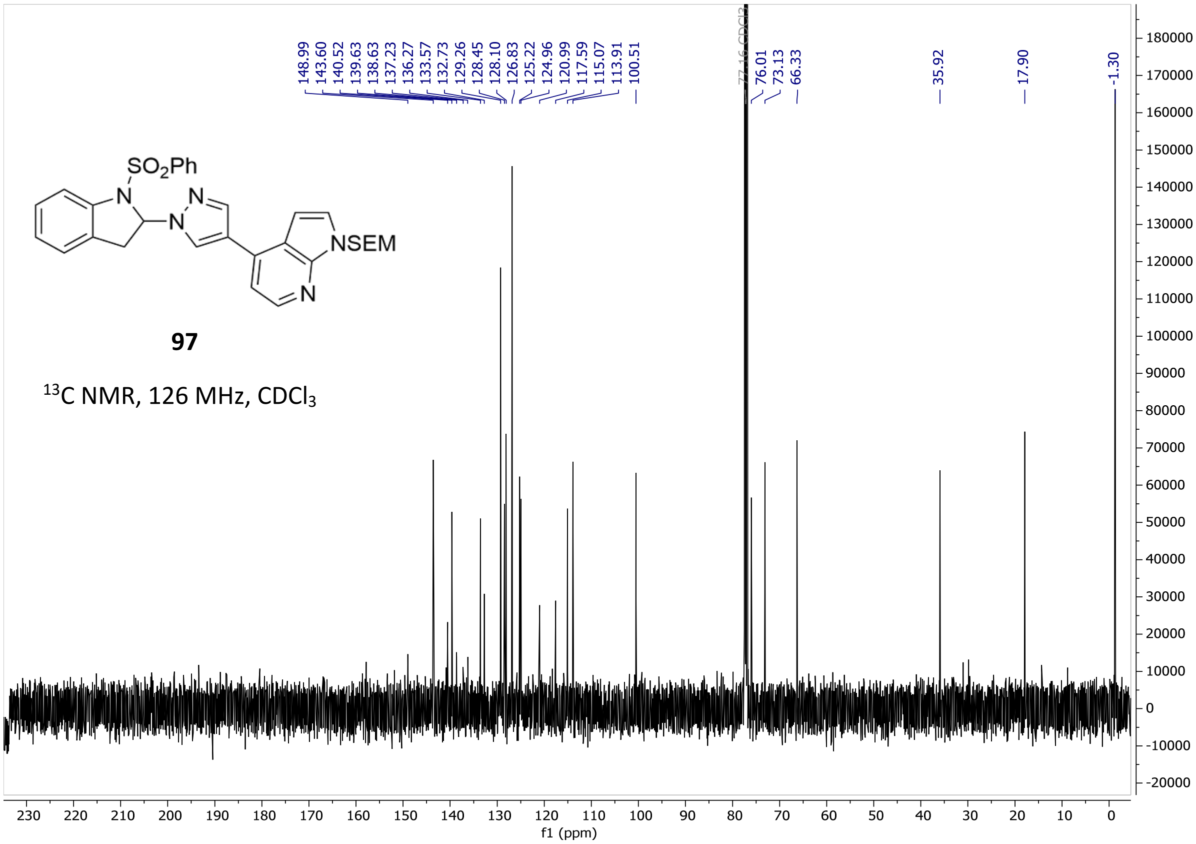
148.99 143.60 140.52 139.63 138.63 137.23 136.27 133.57 132.73 129.26 128.45 128.10 126.83 125.22 124.96 120.99 117.59 115.07 113.91 100.51
77.16 CDCl3
76.01 73.13 66.33
35.92
17.90
-1.30
SO2Ph
N
N
N
NSEM
N
97
13C NMR, 126 MHz, CDCl3
f1 (ppm)

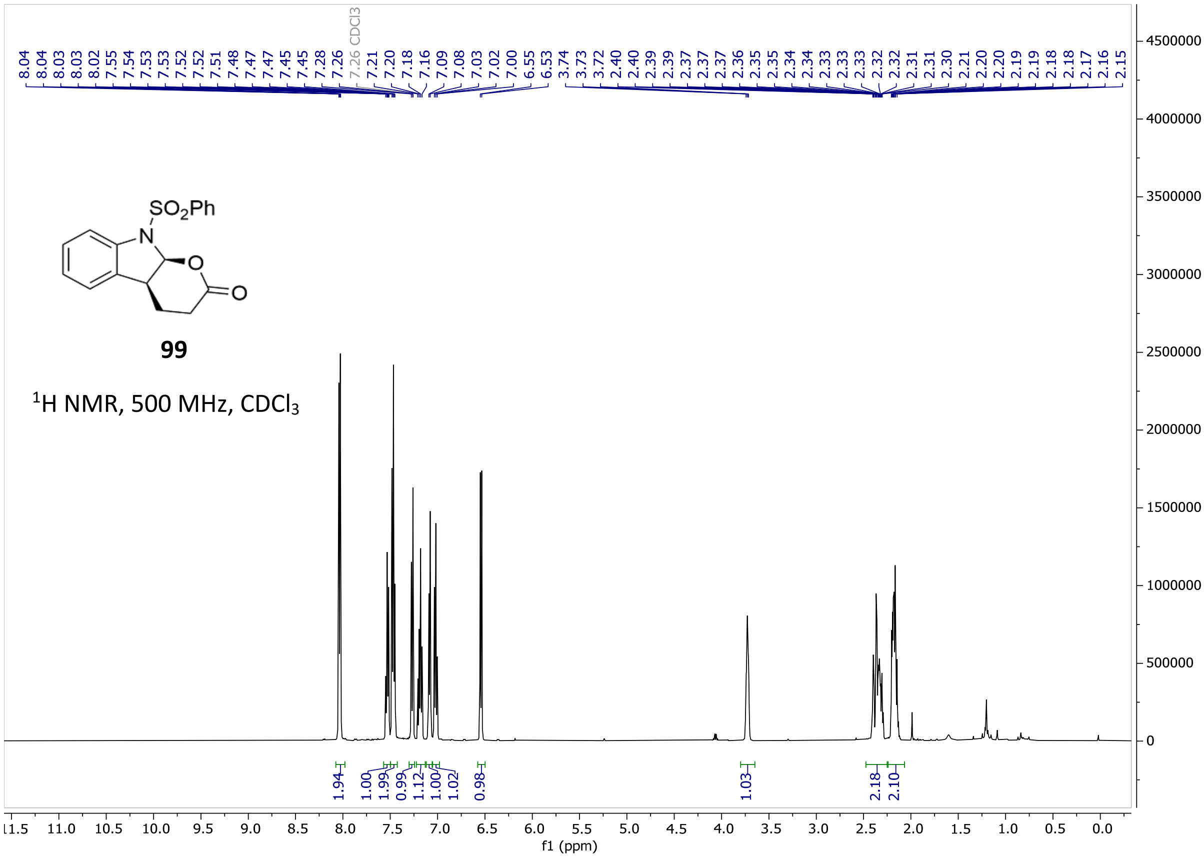
8.04 8.04 8.03 8.02 7.55 7.54 7.53 7.53 7.52 7.52 7.51 7.48 7.47 7.47 7.45 7.45 7.28 7.26 7.26 CDCl3 7.21 7.20 7.18 7.16 7.09 7.08 7.03 7.02 7.00 6.55 6.53 3.74 3.73 3.72 2.40 2.39 2.39 2.37 2.37 2.37 2.36 2.35 2.35 2.34 2.34 2.33 2.33 2.33 2.32 2.32 2.31 2.30 2.21 2.20 2.20 2.19 2.19 2.18 2.18 2.17 2.16 2.15
SO2Ph
N
O
O
99
1H NMR, 500 MHz, CDCl3
1.94 1.00 1.99 0.99 1.12 1.00 1.02 0.98 1.03 2.18 2.10
f1 (ppm)

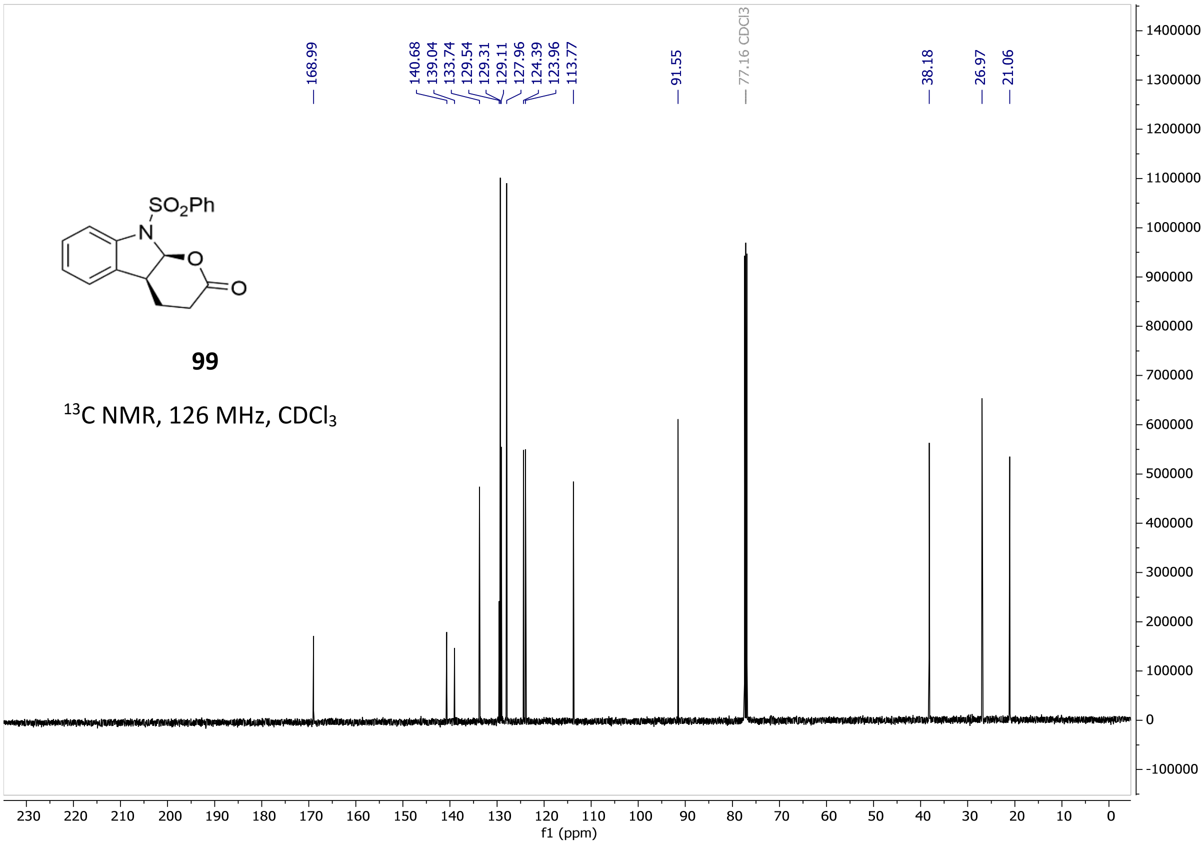
168.99 140.68 139.04 133.74 129.54 129.31 129.11 127.96 124.39 123.96 113.77 91.55 77.16 CDCl3 38.18 26.97 21.06
SO2Ph
N
O
O
99
13C NMR, 126 MHz, CDCl3
f1 (ppm)

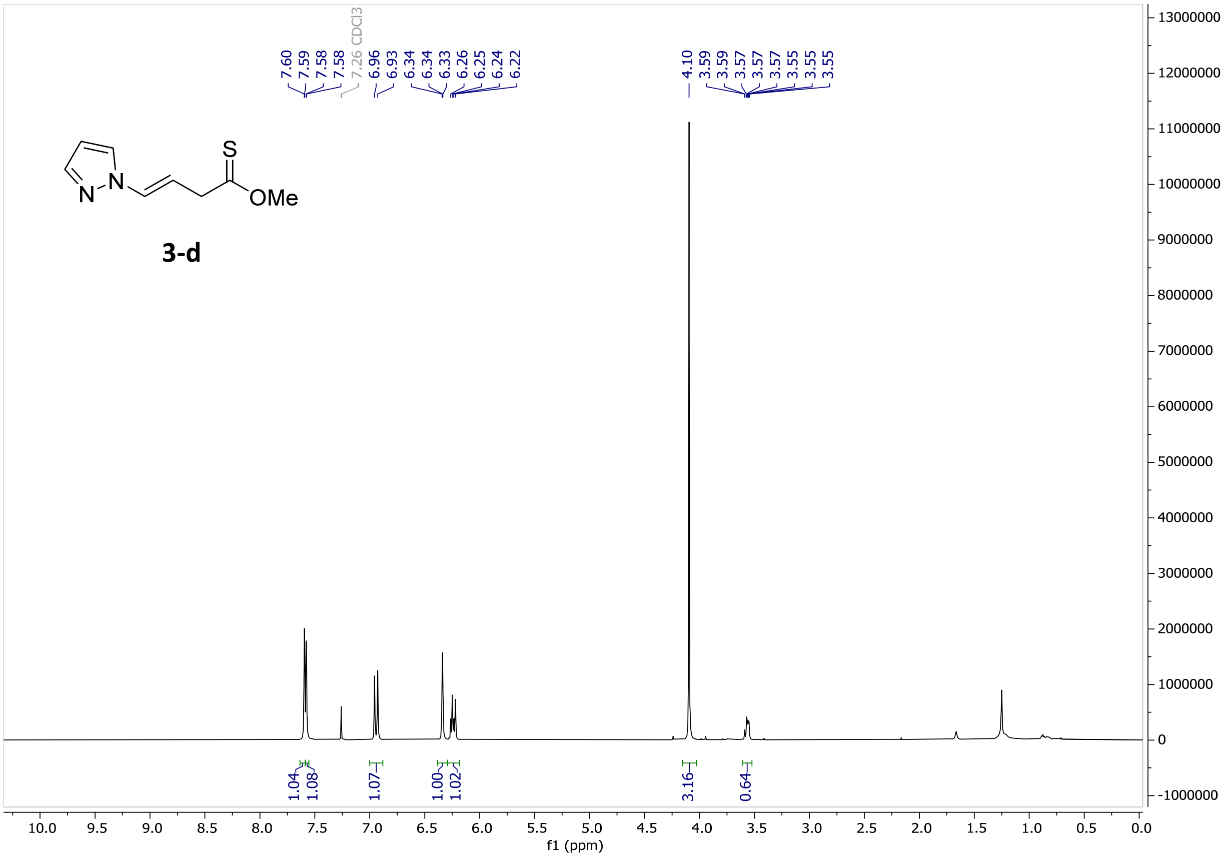
7.60
7.59
7.58
7.58
7.26 CDCl3
6.96
6.93
6.34
6.34
6.33
6.26
6.25
6.24
6.22
4.10
3.59
3.59
3.57
3.57
3.57
3.55
3.55
3.55
S
N
N
OMe
3-d
1.04
1.08
1.07
1.00
1.02
3.16
0.64
f1 (ppm)

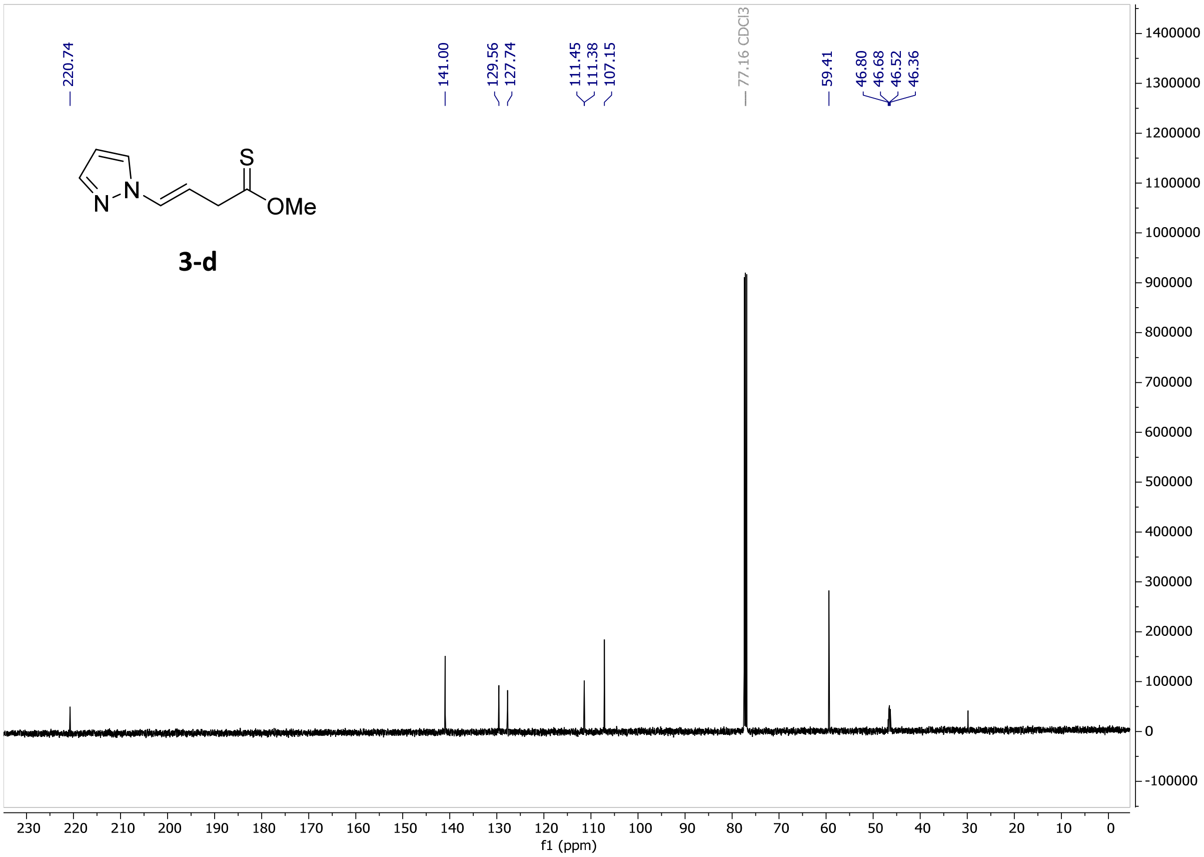
220.74
141.00
129.56
127.74
111.45
111.38
107.15
77.16 CDCl3
59.41
46.80
46.68
46.52
46.36
S
N
N
OMe
3-d
f1 (ppm)

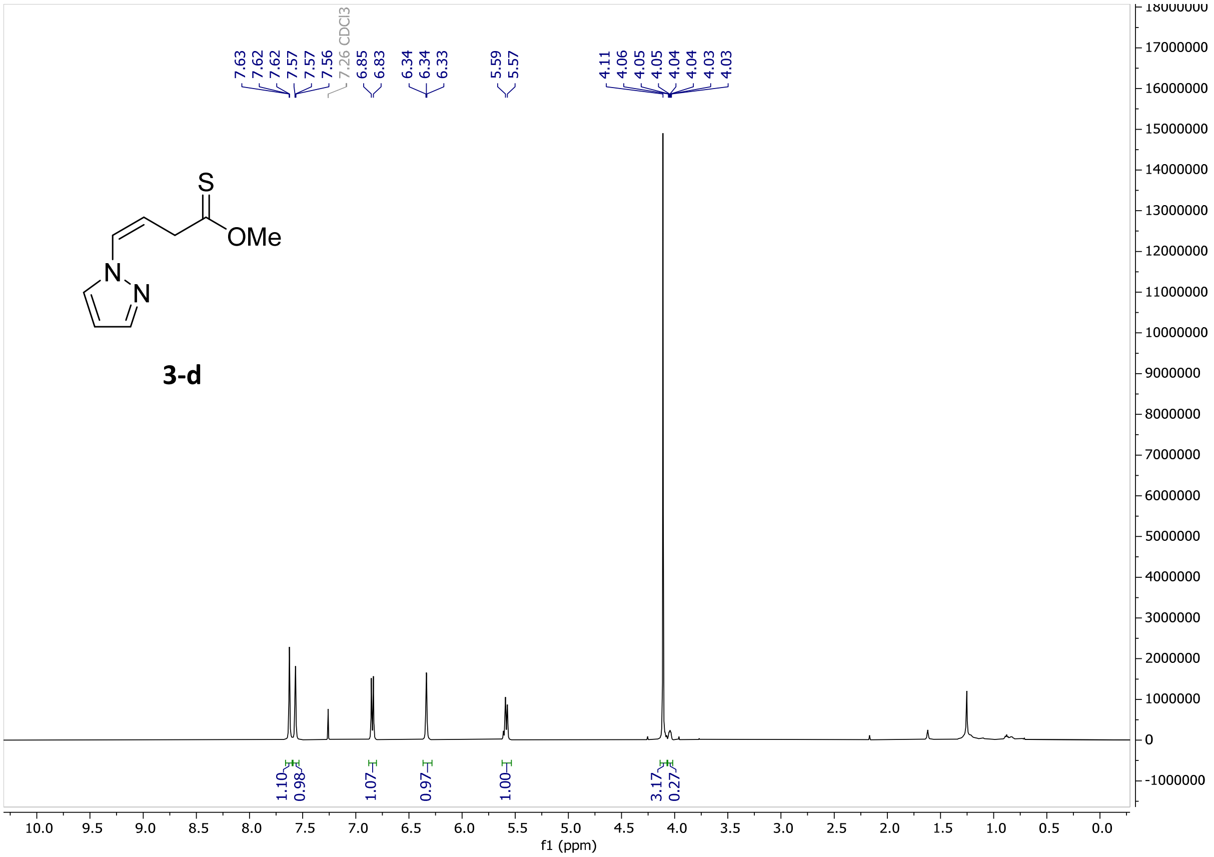
7.63
7.62
7.62
7.57
7.57
7.56
7.26 CDCl3
6.85
6.83
6.34
6.34
6.33
5.59
5.57
4.11
4.06
4.05
4.05
4.04
4.04
4.03
4.03
S
OMe
N
N
1.10
0.98
1.07
0.97
1.00
3.17
0.27
f1 (ppm)

3-d

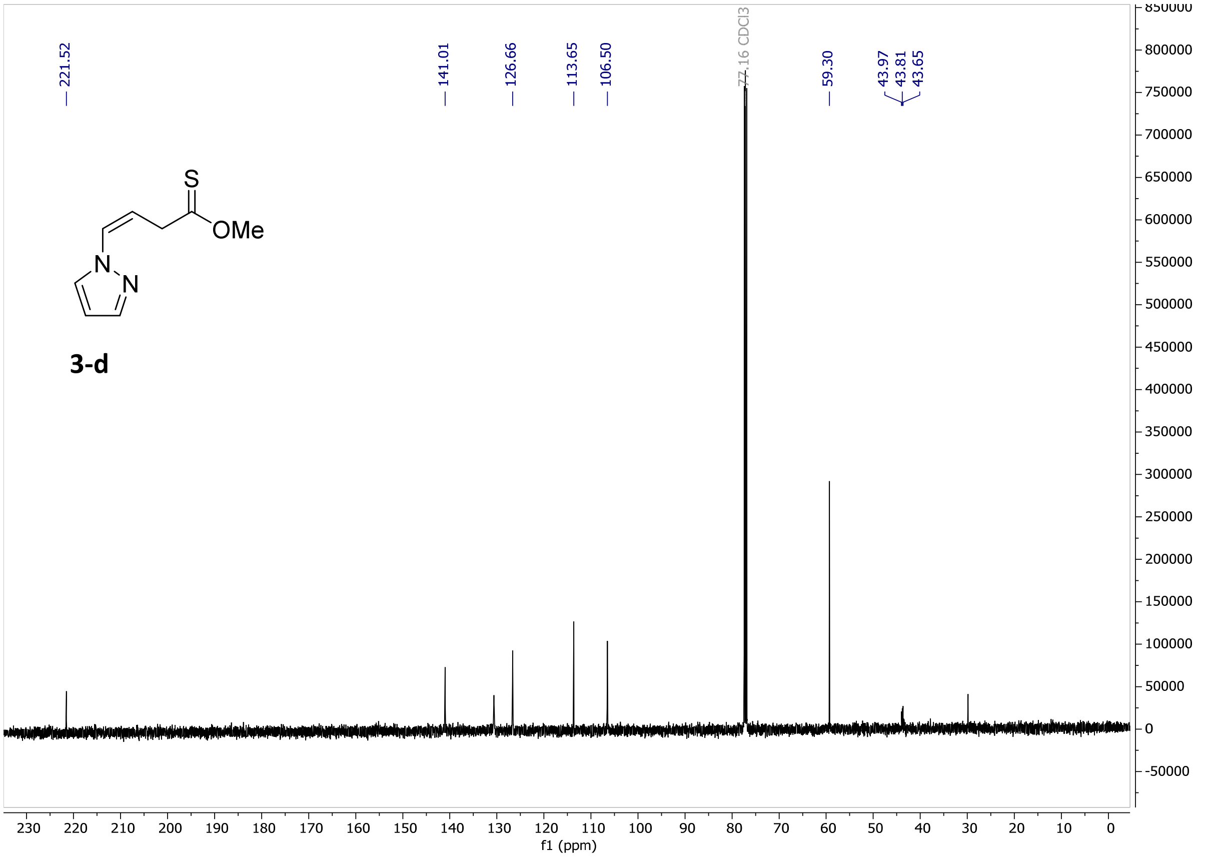
221.52
141.01
126.66
113.65
106.50
77.16 CDCl3
59.30
43.97
43.81
43.65
S
OMe
N
N
f1 (ppm)

3-d

6. Single crystal X-ray diffraction data of 30

Table S5 Crystal data and structure refinement for mo_220822Peng_0ma.

Identification code	mo_220822Peng_0ma
Empirical formula	$C_8H_9BrN_2O_2$
Formula weight	245.08
Temperature/K	100
Crystal system	orthorhombic
Space group	Pccn
a/Å	13.2630(13)
b/Å	24.954(2)
c/Å	5.6399(5)
α/°	90
β/°	90
γ/°	90
Volume/Å^3	1866.6(3)
Z	8
ρ_{calc}g/cm^3	1.744
μ/mm^{-1}	4.372
F(000)	976.0
Crystal size/mm^3	0.4 × 0.2 × 0.12
Radiation	MoKα (λ = 0.71073)
2Θ range for data collection/°	3.264 to 52.766
Index ranges	$-16 \leq h \leq 15$, $-31 \leq k \leq 31$, $-7 \leq l \leq 6$
Reflections collected	11450
Independent reflections	1899 [R_{int} = 0.0190, R_{sigma} = 0.0128]

Data/restraints/parameters	1899/0/119
Goodness-of-fit on F^2	1.042
Final R indexes [I>=2σ (I)]	R_1 = 0.0185, wR_2 = 0.0462
Final R indexes [all data]	R_1 = 0.0213, wR_2 = 0.0473
Largest diff. peak/hole / e Å^{-3}	0.42/-0.23

Molecular structure. There are 8 molecules per unit cell, 1 molecule per asymmetric unit. The thermal displacement ellipsoids are at a 50% probability level.

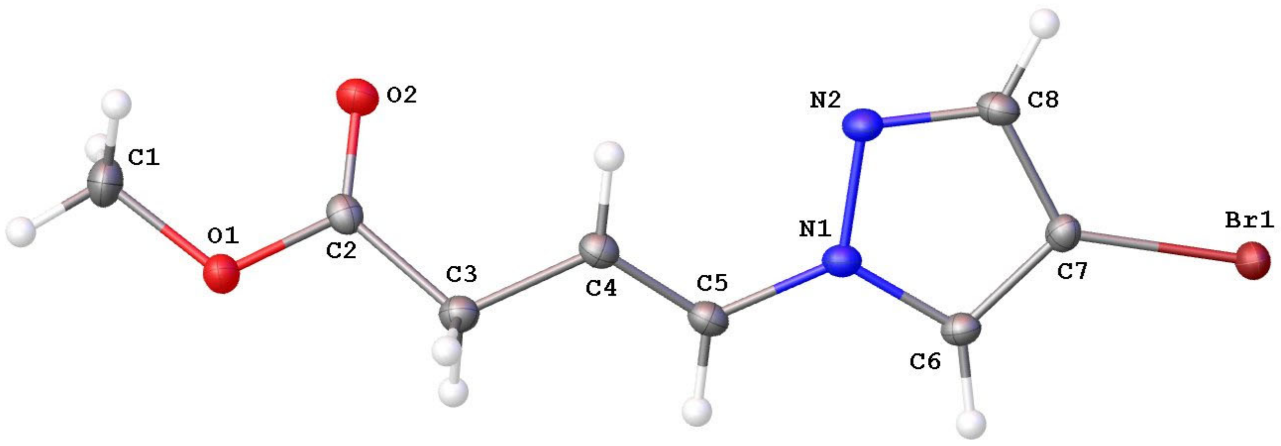

Unit cell and packing. View almost along axis c:

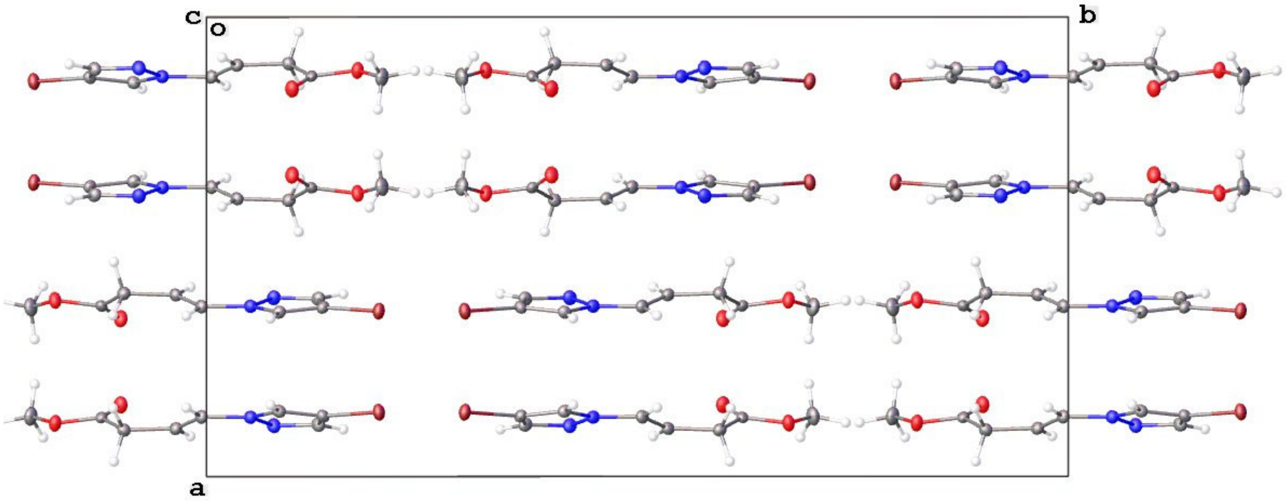

Table S6 Fractional Atomic Coordinates (×10^4) and Equivalent Isotropic Displacement Parameters (Å^2×10^3) for mo_220822Peng_0ma. U_{eq} is defined as 1/3 of of the trace of the orthogonalised U_{IJ} tensor.

**Atom**	***x***	***y***	***z***	**U(eq)**
Br1	3598.4(2)	7001.0(2)	-1184.7(3)	17.81(7)
O1	3838.3(9)	3244.7(5)	5371(2)	18.6(2)
O2	3437.3(8)	4006.0(5)	7275(2)	19.7(3)
N1	3707.8(9)	5511.8(6)	1510(2)	14.4(3)
N2	3891.7(10)	5782.8(6)	3556(2)	16.3(3)
C4	3953.1(11)	4643.0(6)	3285(3)	15.3(3)
C3	3985.8(12)	4041.9(6)	3180(3)	15.7(3)
C6	3576.9(11)	5844.0(7)	-372(3)	14.4(3)
C7	3682.3(11)	6353.9(6)	503(3)	14.3(3)
C5	3699.6(11)	4942.5(7)	1461(3)	15.6(3)
C2	3712.9(11)	3781.5(7)	5508(3)	14.4(3)
C8	3872.1(12)	6297.8(7)	2930(3)	16.3(3)
C1	3687.8(13)	2962.1(7)	7582(3)	23.3(4)

Table S7 Anisotropic Displacement Parameters (Å^2×10^3) for mo_220822Peng_0ma. The Anisotropic displacement factor exponent takes the form: $-2\pi^2[h^2a^{*2}U_{11}+2hka^*b^*U_{12}+\ldots]$.

**Atom**	**U_{11}**	**U_{22}**	**U_{33}**	**U_{23}**	**U_{13}**	**U_{12}**
Br1	25.59(11)	12.92(10)	14.92(11)	0.28(6)	-0.75(6)	1.71(6)
O1	25.8(6)	13.8(6)	16.3(6)	0.2(5)	2.7(5)	-1.4(5)
O2	26.1(6)	16.9(6)	16.2(6)	-2.5(5)	3.5(5)	0.7(5)

Table S7 Anisotropic Displacement Parameters (Å^2×10^3) for mo_220822Peng_0ma. The Anisotropic displacement factor exponent takes the form: $-2\pi^2[h^2a^{*2}U_{11}+2hka^*b^*U_{12}+\ldots]$.

**Atom**	**U_{11}**	**U_{22}**	**U_{33}**	**U_{23}**	**U_{13}**	**U_{12}**
N1	16.2(6)	15.4(7)	11.7(6)	-1.9(5)	-0.6(5)	0.0(5)
N2	20.0(6)	17.1(7)	11.8(6)	-1.9(5)	-2.9(5)	0.5(5)
C4	15.3(7)	15.2(8)	15.5(8)	-2.3(6)	-0.6(6)	-1.1(6)
C3	16.2(7)	16.6(8)	14.3(8)	-0.9(7)	0.5(6)	0.8(6)
C6	15.3(7)	16.7(8)	11.3(7)	-1.1(6)	-1.1(6)	1.0(6)
C7	14.8(7)	14.1(8)	14.0(7)	1.3(6)	0.7(6)	2.5(6)
C5	16.6(7)	14.8(8)	15.2(8)	-3.4(6)	-0.3(6)	-0.5(6)
C2	12.0(7)	13.3(8)	17.9(8)	-0.3(7)	-2.3(6)	-1.2(6)
C8	17.8(7)	17.0(8)	14.3(8)	-4.1(7)	-1.8(6)	1.2(6)
C1	31.3(10)	16.4(9)	22.2(10)	5.2(7)	1.3(7)	-2.5(7)

Table S8 Bond Lengths for mo_220822Peng_0ma.

**Atom**	**Atom**	**Length/Å**	**Atom**	**Atom**	**Length/Å**
Br1	C7	1.8778(16)	N2	C8	1.333(2)
O1	C2	1.3519(19)	C4	C3	1.502(2)
O1	C1	1.446(2)	C4	C5	1.315(2)
O2	C2	1.200(2)	C3	C2	1.509(2)
N1	N2	1.3597(18)	C6	C7	1.372(2)
N1	C6	1.358(2)	C7	C8	1.399(2)
N1	C5	1.421(2)			

Table S9 Bond Angles for mo_220822Peng_0ma.

Atom	Atom	Atom	Angle/°	Atom	Atom	Atom	Angle/°
C2	O1	C1	114.65(13)	C6	C7	Br1	127.53(13)
N2	N1	C5	121.00(13)	C6	C7	C8	106.11(15)
C6	N1	N2	112.50(13)	C8	C7	Br1	126.36(13)
C6	N1	C5	126.46(14)	C4	C5	N1	123.44(15)
C8	N2	N1	104.56(13)	O1	C2	C3	110.35(14)
C5	C4	C3	122.98(15)	O2	C2	O1	123.17(16)
C4	C3	C2	112.90(14)	O2	C2	C3	126.47(15)
N1	C6	C7	105.79(14)	N2	C8	C7	111.05(15)

Table S10 Torsion Angles for mo_220822Peng_0ma.

A	B	C	D	Angle/°	A	B	C	D	Angle/°
Br1	C7	C8	N2	-179.38(11)	C6	N1	N2	C8	0.16(17)
N1	N2	C8	C7	-0.30(18)	C6	N1	C5	C4	171.33(15)
N1	C6	C7	Br1	179.49(10)	C6	C7	C8	N2	0.33(18)
N1	C6	C7	C8	-0.21(16)	C5	N1	N2	C8	178.12(13)
N2	N1	C6	C7	0.04(17)	C5	N1	C6	C7	-177.78(13)
N2	N1	C5	C4	-6.3(2)	C5	C4	C3	C2	-147.55(15)
C4	C3	C2	O1	-174.75(12)	C1	O1	C2	O2	-4.5(2)
C4	C3	C2	O2	4.1(2)	C1	O1	C2	C3	174.43(12)
C3	C4	C5	N1	-177.21(14)					

Table S11 Hydrogen Atom Coordinates (Å×10^4) and Isotropic Displacement Parameters (Å^2×10^3) for mo_220822Peng_0ma.

Atom	*x*	*y*	*z*	U(eq)
H4	4123.15	4814.67	4733.85	18
H3B	4672.79	3927.27	2717.42	19
H3A	3512.87	3916.29	1941.22	19
H6	3440.66	5743.99	-1965.36	17
H5	3500.64	4769.14	36.01	19
H8	3973.65	6588.3	3992.5	20
H1A	3828.1	2580.13	7350.71	35
H1B	2988.51	3007.98	8105.37	35
H1C	4144.83	3106.83	8788.03	35

Experimental

The single crystal XRD of $C_8H_9BrN_2O_2$ [mo_220822Peng_0ma] was studied on a 'Bruker Kappa APEX-II' diffractometer. The crystal was kept at 100.0 K during data collection. Using the Olex2 [1] environment, the structure was solved with the ShelXT [2] structure solution program using Intrinsic Phasing and refined with the ShelXL [3] refinement package using Least Squares minimization.

1. Dolomanov, O.V., Bourhis, L.J., Gildea, R.J, Howard, J.A.K. & Puschmann, H. (2009), J. Appl. Cryst. 42, 339-341.
2. Sheldrick, G.M. (2015). Acta Cryst. A71, 3-8.
3. Sheldrick, G.M. (2015). Acta Cryst. C71, 3-8.

Crystal structure determination of [mo_220822Peng_0ma]

Crystal Data for $C_8H_9BrN_2O_2$ (M =245.08 g/mol): orthorhombic, space group Pccn (no. 56), a = 13.2630(13) Å, b = 24.954(2) Å, c = 5.6399(5) Å, V = 1866.6(3) Å^3, Z = 8, T = 100 K, μ(MoKα) = 4.372 mm^{-1}, $Dcalc$ = 1.744 g/cm^3, 11450 reflections measured (3.264° ≤ 2Θ ≤ 52.766°), 1899 unique (R_{int} = 0.0190, R_{sigma} = 0.0128) which were used in all calculations. The final R_1 was 0.0185 (I > 2σ(I)) and wR_2 was 0.0473 (all data).

Refinement model description

Number of restraints - 0, number of constraints - unknown.

Details:

```
1. Fixed Uiso
 At 1.2 times of:
  All C(H) groups, All C(H,H) groups
 At 1.5 times of:
  All C(H,H,H) groups
2.a Secondary CH2 refined with riding coordinates:
 C3(H3B,H3A)
2.b Aromatic/amide H refined with riding coordinates:
 C4(H4), C6(H6), C5(H5), C8(H8)
2.c Idealised Me refined as rotating group:
 C1(H1A,H1B,H1C)
```

References:

APEX2 (data collection)

Bruker (2007). *APEX2*. Bruker AXS Inc., Madison, Wisconsin, USA.

SAINT (integration and reduction)

Bruker (2007). *SAINT*. Bruker AXS Inc., Madison, Wisconsin, USA.

SADABS (absorption correction)

Sheldrick, G. M. (1996). SADABS. University of Göttingen, Germany.

SHELXTL (structure solution and refinement)

Sheldrick, G. M. (2008). *Acta Cryst.* **A64**, 112–122.

PLATON

Spek, A. L. (2003). *J. Appl. Cryst.* **36**, 7–13.

OLEX2

Dolomanov, O.V., Bourhis, L.J., Gildea, R.J., Howard, J.A.K., Puschmann H. (2008), *J. Appl. Cryst.* **42**, 339-341.
